# Leveraging viral genome sequences and machine learning models for identification of potentially selective antiviral agents

**DOI:** 10.1038/s42004-025-01583-2

**Published:** 2025-06-20

**Authors:** Tuan Xu, Miao Xu, Qi Zhang, Catherine Z. Chen, Wei Zheng, Ruili Huang

**Affiliations:** Division of Pre-clinical Innovation, National Center for Advancing Translational Sciences (NCATS), National Institutes of Health (NIH), Rockville, MD USA

**Keywords:** Virtual screening, Cheminformatics, SARS-CoV-2, Screening

## Abstract

Viral genome sequencing provides valuable information for antiviral development, yet its integration with machine learning for virtual screening remains underexplored. To bridge this gap, viral genome sequences were combined with structural data of approved and investigational antivirals to identify virus-selective agents. In parallel, quantitative structure-activity relationship (QSAR) models were built to predict pan-antivirals. Robust models were generated with the area under the receiver operating characteristic curve (AUC-ROC) >0.72 for virus-selective and >0.79 for pan-antiviral predictions. These models were applied to virtually screen ~360 K compounds for anti-SARS-CoV-2 activity. The 346 compounds identified by the models were tested using two in vitro assays, yielding hit rates of 9.4% (24/256) in the pseudotyped particle (PP) entry assay and 37% (47/128) in the RNA-dependent RNA polymerase (RdRp) assay. The top compounds showed potencies around 1 µM. This study provides a framework for virtual screening of virus-selective and pan- antivirals against emerging pathogens.

## Abstract (web summary)

Leveraging viral genome sequencing for antiviral drug development remains underexplored in machine learning applications. Here, the authors integrate viral genome sequences with drug structural data to create robust predictive models, identifying potential antivirals (e.g., anti-SARS-CoV-2 compounds).

## Introduction

Viral pandemics have posed significant challenges to global public health, with widespread consequences for healthcare systems and economies^1–3^. The COVID-19 pandemic, caused by SARS-CoV-2, fully exemplifies this threat. According to World Health Organization (WHO) records, as of May 30, 2024, COVID-19 has claimed over 7 million lives worldwide, with its financial impact amounting to 85.91% of global healthcare expenditures and 9.13% of gross domestic product (GDP)^4,5^. The continuous evolution of viruses, including the emergence of drug-resistant virus strains/variants and highly pathogenic new viruses, has raised concerns due to the diminished effectiveness of existing therapeutic regimens. A prominent example is the acquired immunodeficiency syndrome (AIDS), the most advanced stage of the human immunodeficiency virus (HIV) infection. Despite the remarkable success of the highly active antiretroviral therapy (HAART) in controlling HIV infection and improving patient outcomes, the widespread use of reverse transcriptase inhibitors has led to the development of drug-resistant HIV strains, thereby significantly reducing the clinical efficacy of current antiretroviral treatments^6^. Moreover, many viruses, such as Ebola and Nipah, still lack effective antiviral drugs, partly due to corporate strategic decisions in pharmaceutical development and technical challenges, continuing to pose significant threats to global health and safety^7,8^. These challenges underscore the urgent need for the rapid discovery of antiviral drugs capable of targeting specific pathogenic viruses and emerging strains/variants for potential outbreaks and future pandemics.

Antiviral drug discovery and development have traditionally been inefficient, costly, and time-consuming, often involving the experimental screening of millions of compounds for lead compound discovery^9,10^. Advances in computer-aided drug design (CADD) have provided a promising alternative by integrating bioinformatics, cheminformatics, and machine learning to streamline the screening of large antiviral compound libraries and optimization of lead compounds^11,12^. Among CADD techniques, machine learning-based virtual screening (VS) has emerged as a powerful tool, capable of modeling complex relationships and processing high-dimensional data to predict antiviral activity^13^. Machine learning algorithms such as support vector machines (SVM), random forests (RF), and eXtreme Gradient Boosting (XGB) are frequently employed to extract relevant features from known antiviral compounds and predict their efficacy against specific viral targets^14–16^. These methods efficiently prioritize compounds based on predicted antiviral activity, reducing the experimental search space and accelerating the identification of promising drug candidates.

Despite their immense potential, current machine learning-based VS methods for antiviral drug discovery still face significant challenges that hinder their full effectiveness and utility. Many existing models are often limited to specific targets against individual viruses, leading to high rates of false positives and inefficiencies in the drug discovery process^15,17^. Most models rely heavily on single-view data inputs, such as compound structures or molecular descriptors, without fully incorporating critical information from viral target protein sequences and structures, which are equally informative for accurately predicting compound activity^18^. For example, analyzing the relationships between variations in HIV-1 genomic sequences and drug molecular structures through machine learning could lead to the identification of novel lead compounds with unique mechanisms of action, potentially addressing drug-resistant HIV-1 infections^19^. Furthermore, many studies employ only a single machine learning algorithm, which limits their ability to capture diverse latent information. For instance, while SVMs may be sensitive to outliers, neural networks are typically more robust to noise. Additionally, many models are virus-specific and lack the flexibility to rapidly screen for antiviral compounds against different viral subtypes or emerging strains/variants^20^. This limitation is particularly concerning for rapidly evolving pathogens, such as influenza viruses, where new strains or variants frequently render existing vaccines and treatments ineffective^21^.

To address the challenges in machine learning-based VS for antiviral drug discovery, this study presents a novel ensemble framework. Unlike traditional target-specific approaches that rely on predefined viral proteins, our method leverages broader drug–virus interaction patterns without being restricted to a single target. By integrating compound structural data with viral genome sequences, our models can identify selective inhibitors of a single virus as well as pan-antiviral agents. The term “pan-antiviral” refers to broad-spectrum antiviral drugs in this study. The use of multiple algorithms (consensus models) allows for a more comprehensive representation of drug–virus interactions, addressing the limitations of single-algorithm approaches. We applied this framework to predict potential anti-SARS-CoV-2 compounds, followed by further testing using in vitro assays. Our findings demonstrate that this approach enables efficient antiviral discovery and can be extended to rapidly identify therapeutic candidates in response to emerging viral threats.

## Results

### Optimal predictive models for virus-selective antiviral drug candidates

**Fig. 1:**
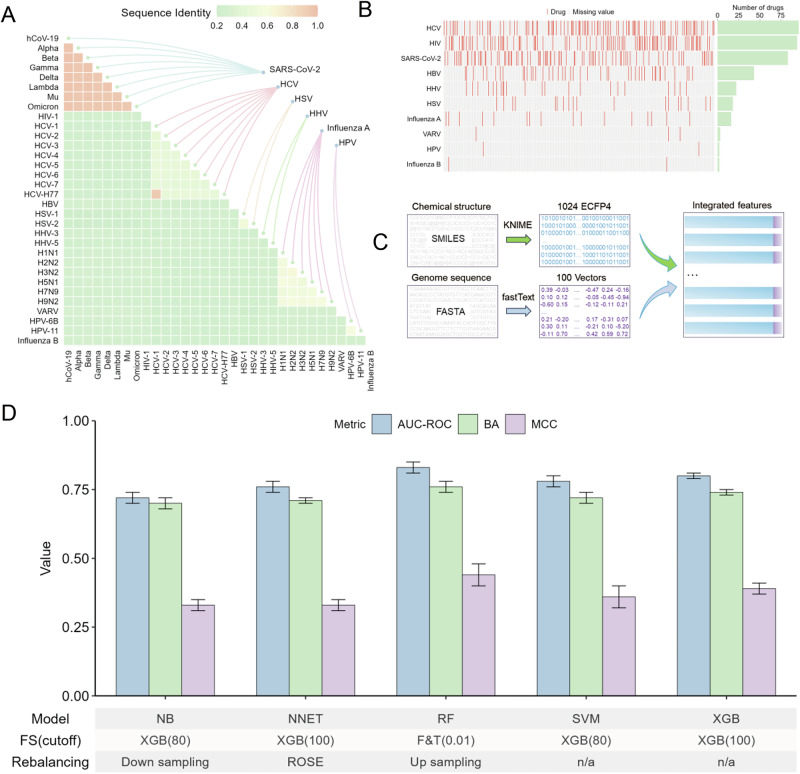
Comprehensive analysis and predictive modeling of virus-selective drugs. **A** Heatmap visualization of pairwise genomic similarity among common pathogenic viruses. Green indicates sequence dissimilarity, while orange denotes sequence similarity. **B** Distribution of approved and investigational antiviral drugs. The left panel displays the overall distribution of these drugs, while the right panel shows the number of drugs corresponding to each type of virus. **C** Flowchart outlining the feature components used for selective antiviral drug modeling. **D** Performance evaluation of the optimal predictive models and their corresponding parameter combinations. Results are presented as mean ± standard deviation (SD), with error bars representing the SD from 20 independent iterations. AUC-ROC area under the receiver operating characteristic curve, BA balanced accuracy, ECFP4 extended connectivity fingerprint 4, FASTA fast alignment search tool for DNA/RNA sequences; F&T Fisher’s exact test and *t*-test, FS feature selection, HBV hepatitis B virus, HCV hepatitis C virus, HHV human herpesvirus, HIV human immunodeficiency virus, HPV human papillomavirus, HSV herpes simplex virus, MCC Matthews correlation coefficient, NB naïve bayes, NNET neural network, RF random forest, ROSE random over-sampling examples, SARS-CoV-2 severe acute respiratory syndrome coronavirus 2, SMILES simplified molecular input line entry system, SVM support vector machine, VARV variola virus, XGB eXtreme gradient boosting.

Complete genome assemblies of 32 strains/variants from ten different viruses were retrieved as FASTA files from the GISAID, EBI, and NCBI databases (Supplementary Table S1). The viral genome sequences exhibited high conservation among strains/variants within the same virus (e.g., >94% sequence identity among eight SARS-CoV-2 strains/variants) but significant divergence across different viruses (e.g., <26% sequence identity between SARS-CoV-2 and HCV strains/variants) (Fig. 1A). A total of 303 approved and investigational antiviral drugs (AIADs), corresponding to 378 drug–virus pairs, were compiled from the NCATS in-house collection and DrugBank database (Supplementary Table S2). These drug–virus pairs encompass multiple virus types and mechanisms of action, reflecting the diverse ways in which antiviral compounds exert their effects. The number of AIADs available for each virus varied significantly, ranging from 2 (e.g., anti-influenza B drugs and anti-HPV drugs) to 96 (anti-HCV drugs), with a median of 20 drugs per virus (Fig. 1B). The 303 AIADs and the ten viruses form a total of 3030 possible drug–virus combinations. For modeling purposes, if a drug was reported to exhibit antiviral activity against a particular virus, this drug–virus pair was designated as 1 (positive), and other combinations were designated as 0 (negative). For example, entecavir has known anti-HBV activity and is not known to target SARS-CoV-2, as such, the entecavir-HBV combination was assigned an outcome of 1 and the entecavir-SARS-CoV-2 combination was assigned an outcome of 0. This resulted in 378 positive outcomes and 2652 negative outcomes that served as the input for the models. Compound structures (represented as 1024-bit ECFP4 fingerprints) and viral genome sequences (represented as 100-dimension vectors) were used as input features to construct and evaluate virus-selective models using five machine learning algorithms (Fig. 1C). The detailed model inputs are available on GitHub at https://github.com/TX-2017/antivirals_prediction. Data were split into training (70%) and test (30%) sets based on the number of unique compounds. Following parameter optimization, all five models achieved robust predictive performance, with AUC-ROC >0.72, BA >0.70, and MCC >0.33 (Fig. 1D and Supplementary Table S3). Among them, the top two models were RF (AUC-ROC = 0.83 ± 0.02, BA = 0.76 ± 0.02, and MCC = 0.44 ± 0.04), and XGB (AUC-ROC = 0.80 ± 0.01, BA = 0.74 ± 0.01, and MCC = 0.39 ± 0.02) (Fig. 1D). The RF model was optimized using feature selection based on Fisher’s exact test and t-test with a significance threshold of 0.01 (i.e., 160 ECFP4s and 62 viral genome sequence descriptors), followed by data rebalancing through the application of the up-sampling method. The XGB model was optimized using feature selection based on XGB with a threshold of 100 (i.e., 50 ECFP4s and 50 viral genome sequence descriptors), without any need for data rebalancing.

### Optimal predictive models for pan-antiviral drug candidates

**Fig. 2:**
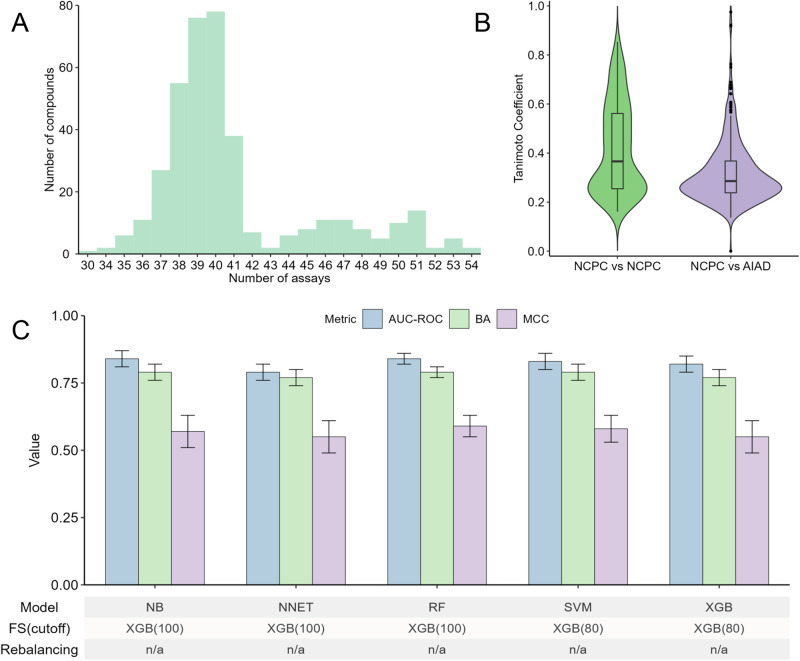
Comprehensive analysis and predictive modeling of pan-antiviral drugs. **A** Distribution of non-cytotoxic approved and investigational drugs in the Tox21 cell viability assays. Compounds were selected based on a stringent criterion of being inactive (non-cytotoxic) in at least 30 assays. **B** Structural similarity analysis of approved and investigational antiviral drugs compared to non-cytotoxic pharmaceutical compounds (NCPC) using the Tanimoto coefficient. **C** Performance evaluation of the optimal predictive models and their respective parameter combinations. Results are presented as mean ± standard deviation (SD), with error bars representing the SD from 20 independent iterations. AUC-ROC area under the receiver operating characteristic curve, BA balanced accuracy, FS feature selection, MCC Matthews correlation coefficient, NB naïve Bayes, NCPC non-cytotoxic pharmaceutical compounds, NNET neural network, RF random forest, SVM support vector machine, XGB eXtreme gradient boosting.

A total of 385 non-cytotoxic pharmaceutical compounds (NCPCs) were selected as negative controls based on their activity profiles in the cell viability count screens of the Tox21 assays, where each compound was classified as inactive (non-cytotoxic) in at least 30 out of 55 assays (Fig. 2A and Supplementary Table S4). None of these compounds were inactive across all 55 assays. For classification model development, 303 AIADs were labeled as 1 (active compounds), while 385 NCPCs were labeled as 0 (inactive compounds). Compound structures, represented as 1024-bit ECFP4 fingerprints, served as input features for QSAR model construction and evaluation using five machine learning algorithms (Fig. 2C). The detailed model inputs are available on GitHub at https://github.com/TX-2017/antivirals_prediction. Structural similarity analysis showed that the average maxTC value (AmaxTC) among the NCPCs was 0.42 ± 0.18, which was much larger than that between NCPCs and AIADs (AmaxTC = 0.31 ± 0.13) (Fig. 2B), indicating that the two groups of compounds are structurally distinct. QSAR models were developed to identify pan-antiviral drugs. All models built on the five machine learning algorithms showed good performance (AUC-ROC >0.79, BA >0.77, MCC >0.55) (Fig. 2C). The top two models were RF (AUC-ROC = 0.84 ± 0.02, BA = 0.79 ± 0.02, and MCC = 0.59 ± 0.04), and SVM (AUC-ROC = 0.83 ± 0.03, BA = 0.79 ± 0.03, and MCC = 0.58 ± 0.05) (Fig. 2C). The RF model was optimized through feature selection using XGB with a threshold of 100 ECFP4s, while the SVM model was optimized with a threshold of 80 ECFP4s. Since the number of NCPCs and AIADs (385 and 303, respectively) was comparable, data rebalancing was not required. A total of 85 chemical structural features were significantly enriched in AIADs compared to NCPCs, while nine features showed enrichment in NCPCs compared to AIADs (Fisher’s exact test with *p* < 0.05; Supplementary Table S5). These two groups of enriched structural features partially overlapped in certain categories but differed in specific details. For example, nitrogen-containing bonds were prominent in both classes, such as carbamate bonds (bond:C(=O)N_carbamate, *p* = 4.52 × 10^−5^) in AIADs and generic carboxamide bonds (bond:C(=O)N_carboxamide_generic, *p* = 0.01) in NCPCs. Similarly, halogen bonds were enriched in both groups, with alkyl dihalo bonds (bond:CX_halide_alkyl-X_dihalo(1_1-), *p* = 0.03) associated with AIADs and inorganic halide bonds (bond:X_halide_inorganic, *p* = 4.52 × 10^−13^) associated with NCPCs. Some structural features were specific to AIADs compared to NCPCs, including nucleoside/nucleotide analogs such as uracil (group:nucleobase_uracil, *p* = 0.001), guanine (group:nucleobase_guanine, *p* = 0.037), and adenine (group:nucleobase_adenine, *p* = 0.048). Other AIAD-specific features included heterocyclic systems such as benzimidazole (ring:hetero_[5_6]N_benzimidazole, *p* = 1.06 × 10^−4^), pyrrole (ring:hetero[5]N_pyrrole, *p* = 0.014), and quinoline (ring:hetero[6_6]_N_quinoline, *p* = 0.002), as well as functional groups like phosphate (bond:P = O_phosphate, *p* = 0.048) and nitrile (bond:C#N_nitrile, *p* = 0.027).

### Virtual screening of potential anti-SARS-CoV-2 drug candidates

**Fig. 3:**
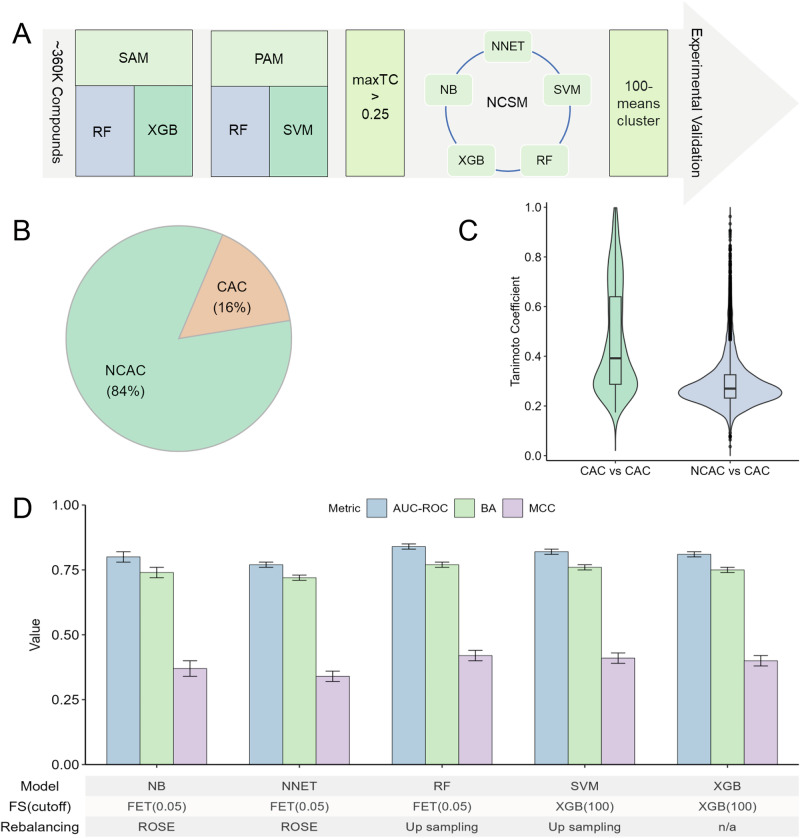
Virtual screening for potential anti-SARS-CoV-2 drugs. **A** Workflow outlining the identification process for potential anti-SARS-CoV-2 drugs. **B** Comparative distribution of non-cytotoxic anti-SARS-CoV-2 compounds (NCACs) and cytotoxic anti-SARS-CoV-2 compounds (CACs). **C** Structural similarity analysis of the NCACs and CACs using the Tanimoto coefficient. **D** Performance evaluation of the optimal predictive models and their respective parameter combinations. Results are presented as mean ± standard deviation (SD), with error bars representing the SD from 20 independent iterations. AUC-ROC area under the receiver operating characteristic curve, BA balanced accuracy, PAM pan-antiviral drug model, FET Fisher’s exact test, FS feature selection, MCC Matthews correlation coefficient, NB naïve Bayes, NNET neural network, SAM selective antiviral drug model, RF random forest, ROSE random over-sampling examples, NCSM non-cytotoxic anti-SARS-CoV-2 model, SVM support vector machine, XGB eXtreme gradient boosting.

A virtual screening of ~360 K compounds was conducted, followed by a five-step filtering process to identify potential anti-SARS-CoV-2 drug candidates (Fig. 3A). First, compounds predicted positive by both the RF and XGB models were identified as potential selective antiviral drugs. Virus-selective anti-SARS-CoV-2 drugs were defined as those predicted to be positive against at least four of the eight SARS-CoV-2 strains/variants (including the original strain and variants such as alpha, beta, delta, gamma, lambda, mu, and omicron). Second, compounds predicted positive by both the SVM and RF (pan-antiviral) models were identified as potential pan-antiviral drugs. Third, compounds exhibiting structural similarity (maxTC >0.25) to at least one of 83 known anti-SARS-CoV-2 drugs (Details in Supplementary Table S2) were selected for further analysis. The purpose of this approach was to ensure that the compounds selected fall within the applicability domain of the model, which includes compounds that share a certain level of structural similarity to the compounds used for model training. Predictions made on compounds outside of this domain are generally deemed less reliable. Fourth, QSAR models (based on chemical structure) were developed to predict compound cytotoxicity using ECFP4 fingerprints derived from the chemical structures. QSAR models, including NB, SVM, NNET, RF, and XGB, were applied to identify non-cytotoxic anti-SARS-CoV-2 candidates based on the outcomes from the cell viability counter screens of the PP and CPE assays. In total, 4595 compounds comprising 3857 non-cytotoxic anti-SARS-CoV-2 compounds (NCACs) and 738 cytotoxic anti-SARS-CoV-2 compounds (CACs), were identified (Fig. 3B and Supplementary Table S6). Structural similarity among the cytotoxic compounds (AmaxTC = 0.46 ± 0.21) was significantly higher than that between the cytotoxic and non-cytotoxic compounds (AmaxTC = 0.30 ± 0.11) (Fig. 3C). The QSAR models achieved strong predictive performance across five machine learning methods (AUC-ROC >0.77, BA >0.72, MCC >0.34) (Fig. 3D). Among them, the RF model performed the best, with an AUC-ROC of 0.84 ± 0.01, BA of 0.77 ± 0.01, and MCC of 0.42 ± 0.02. This model was optimized through feature selection using Fisher’s exact test with a significance threshold of 0.05, and data rebalancing was implemented using the up-sampling method. Detailed model inputs are available on GitHub at https://github.com/TX-2017/antivirals_prediction. Ultimately, compounds predicted positive by at least three of the five models were identified as potential non-cytotoxic anti-SARS-CoV-2 candidates. Finally, these compounds were clustered into 100 groups using the k-means algorithm to ensure structural diversity (Fig. 3A). After excluding compounds that had been previously tested or were unavailable in the NCATS in-house compound library, the remaining candidates were selected for further testing using in vitro assays.

### Testing of predicted anti-SARS-CoV-2 drug candidates using in vitro assays

**Fig. 4:**
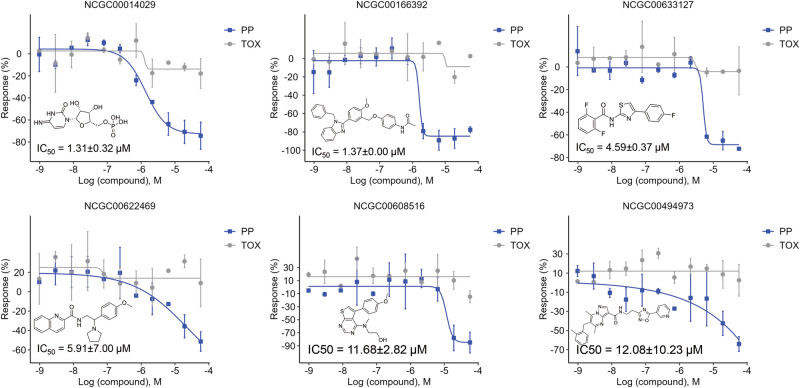
Concentration-response curves of representative SARS-CoV-2 PP entry inhibitors. Results are presented as mean ± standard deviation (SD), with error bars representing the SD of two independent experiments. PP pseudotyped particle, TOX cytotoxicity assay, IC_50_ half-maximal inhibitory concentration.

**Fig. 5:**
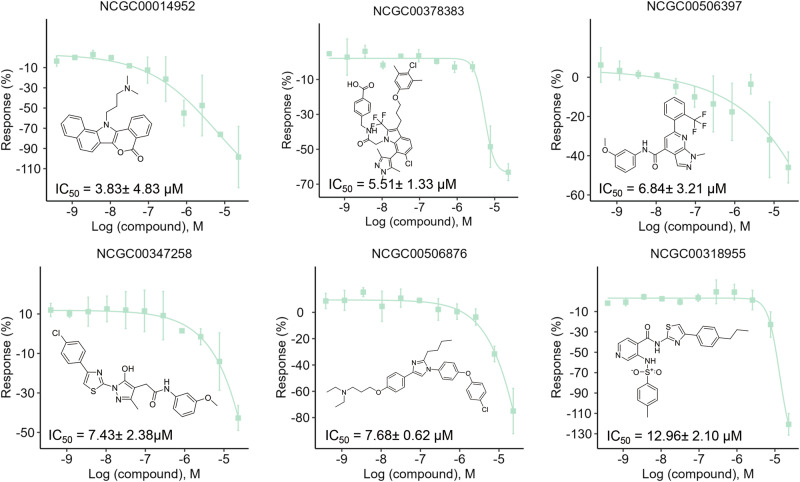
Concentration-response curves of representative SARS-CoV-2 RdRp inhibitors. Results are presented as mean ± standard deviation (SD), with error bars representing the SD of two independent experiments. RdRp, RND-dependent RNA polymerase, IC_50_ half-maximal inhibitory concentration.

A total of 346 predicted anti-SARS-CoV-2 compounds were subjected to experimental validation, including 256 tested for PP entry inhibition and 128 tested for RdRp inhibition, across 11 concentrations (Supplementary Tables S7, S8). In the PP entry assay, 24 of the 256 compounds were tested active, resulting in a hit rate of 9.4%. Of the active PP entry inhibitors, four were known compounds with documented biological activity, though not reported as antiviral drugs. The remaining 20 inhibitors were diverse compounds without previously reported biological activity (Supplementary Table S7). In the RdRp assay, 47 of the 128 compounds were confirmed as active, yielding a hit rate of 37%. Of the active RdRp inhibitors, six were known compounds with documented biological activity, one of which, azeliragon, was reported to exhibit anti-SARS-CoV-2 activity^22^, while the others were not known as antivirals. The remaining 41 inhibitors were diverse compounds without previously reported biological activity (Supplementary Table S8). Six representative PP entry inhibitors are highlighted in Fig. 4. Of these compounds, two showed high potency with IC₅₀ <2 μM: NCGC00014029 (IC₅₀ = 1.31 ± 0.32 μM, efficacy = −76.63 ± 14.92%) and NCGC00166392 (IC₅₀ = 1.37 ± 0.00 μM, efficacy = −83.25 ± 8.42%). One compound had moderate potency with IC₅₀s between 2 and 5 μM: NCGC00633127 (IC₅₀ = 4.59 ± 0.37 μM, efficacy = −67.42 ± 5.30%). Three compounds showed lower potency with IC₅₀ ≥5 μM: NCGC00622469 (IC₅₀ = 5.91 ± 7.00 μM, efficacy = −64.06 ± 9.68%), NCGC00608516 (IC₅₀ = 11.68 ± 2.82 μM, efficacy = −93.10 ± 32.33%), and NCGC00494973 (IC₅₀ = 12.08 ± 10.23 μM, efficacy = −79.39 ± 29.14%) (Fig. 4). Figure 5 illustrates six representative RdRp inhibitors, including one potent compound with IC₅₀ <5 μM: NCGC00014952 (IC₅₀ = 3.83 ± 4.83 μM, efficacy = −121.90 ± 50.78%), four moderately potent compounds with IC₅₀s between 5 and 10 μM: NCGC00378383 (IC₅₀ = 5.51 ± 1.33 μM, efficacy = −66.44 ± 6.99%), NCGC00506397 (IC₅₀ = 6.84 ± 3.21 μM, efficacy = −61.84 ± 11.65%), NCGC00347258 (IC₅₀ = 7.43 ± 2.38 μM, efficacy = −56.66 ± 16.43%), and NCGC00506876 (IC₅₀ = 7.68 ± 0.62 μM, efficacy = -88.14 ± 13.15%), and one less potent compound with IC₅₀ ≥10 μM: NCGC00318955 (IC₅₀ = 12.96 ± 2.10 μM, efficacy = −145.28 ± 8.39%) (Fig. 5). The representative compounds were selected based on a combination of factors including inhibitory potency, efficacy and quality of dose-response curve.

## Discussion

In this study, we combined viral genomic sequence information with chemical structure data to develop consensus machine learning classification models for the prediction of antiviral drugs against ten human pathogenic viruses across 32 strains/variants. Using these models, we conducted a large-scale virtual screening of ~360 K compounds from the NCATS in-house library to identify potential anti-SARS-CoV-2 candidates. The predicted anti-SARS-CoV-2 compounds were further stratified to prioritize a set of structurally diverse, non-cytotoxic compounds, which were experimentally validated using a PP entry assay to assess their ability to block SARS-CoV-2 entry into host cells and an RdRp activity assay to evaluate their effectiveness in inhibiting SARS-CoV-2 replication.

Selective antivirals target particular viral pathogens by disrupting key stages of their lifecycle or viral proteins, offering high efficacy with minimal off-target effects^23^. In this study, we compiled a dataset of 303 approved and investigational antiviral drugs (AIADs) against ten human pathogenic viruses (Fig. 1A, B) to construct virus-selective models for antiviral drug prediction. The selection of these 10 human pathogenic viruses was based on four key criteria: (1) their established or potential impact on public health, including their ability to cause human diseases and their pandemic potential, as indicated by historical evidence or risk assessment; (2) the availability of antiviral drugs or ongoing drug development programs targeting these viruses; (3) the completeness of genomic data necessary for computational modeling; and (4) their clinical significance in human infections. Our analysis of viral genome sequences revealed high similarity among strains/variants of the same virus but significant variations across different viral species (Fig. 1A), highlighting the value of viral genomic data in reflecting taxonomic characteristics and aiding in the construction of antiviral prediction models^24,25^. The collection of multiple viral genome sequences serves two main purposes. First, viruses exhibit high genetic variability, leading to multiple variants of the same virus. Collecting multiple sequences helps capture this natural diversity, enabling the development of more comprehensive feature vectors. Second, developing pan-antiviral models necessitates comprehensive access to antiviral drug information across multiple viral families, which enhances model robustness, adaptability, and predictive accuracy. Through parameter optimization, all five machine learning models achieved strong predictive performance (Fig. 1D), utilizing an integrated feature set combining compound structural fingerprints with viral genome sequence descriptors (Supplementary Table S3). This approach enabled more accurate predictions of compound efficacy against selective viral strains/variants. Consistent with our study, Deepthi et al. developed a deep learning ensemble model that combined chemical structures and viral genomic data for drug repurposing against SARS-CoV-2, achieving an AUC-ROC of 0.89 under five-fold cross-validation, underscoring the effectiveness of this integrative approach for antiviral drug discovery^25^. A limitation of our model is its reduced ability to predict selective antiviral drugs for viruses that are under-represented in the training dataset. For example, given that only two antiviral drugs are available for influenza B and HPV, the model’s accuracy in identifying selective antiviral drugs for these viruses may be constrained.

Pan-antiviral compounds, in contrast, target conserved viral components or essential host cell mechanisms shared across multiple virus families, offering a more versatile therapeutic option than selective antiviral compounds^26^. Our study sought to differentiate pan-antiviral compounds from non-cytotoxic pharmaceutical compounds (NCPCs), based on the hypothesis that antiviral drugs may induce cytotoxicity at concentrations effective against viruses^27^. We began with a set of 385 known NCPCs, each demonstrating non-cytotoxic profiles in at least 30 Tox21 cell viability assays (Fig. 2A)^28^. Structural analysis revealed that AIADs share structural features that are distinct from those of NCPCs (Fig. 2B), supporting the hypothesis that these structural features are crucial for antiviral efficacy and play a significant role in the model’s predictive power^15^. Our optimized pan-antiviral models, developed using chemical structure data, exhibited strong performance in predicting pan-antiviral compounds (Fig. 2C). In comparison, Speck-Planche and Kleandrova^29^ developed a multi-condition QSAR model using artificial neural networks (mtc-QSAR-ANN) that successfully predicted molecules with both pan-antiviral and anti-cytokine storm activities. Their model achieved >80% accuracy and identified key molecular fragments that could be assembled into novel compounds with predicted dual activity. By integrating diverse experimental conditions, including multiple biological targets and assay protocols, the mtc-QSAR-ANN model effectively predicted antiviral activities across different viral pathogens^29^. Many chemical features significantly enriched in AIADs compared to NCPCs in this study were known to be associated with antiviral properties. For example, nucleoside analogs represented the largest class of small molecule-based antivirals, forming the backbone of chemotherapy for infections caused by multiple viruses, such as ribavirin, a nucleoside analog effective against various RNA viruses^30^. Some heterocyclic compounds also played key roles in antiviral drug design, such as 2-substituted-5-amidino-benzimidazoles targeting adenovirus, herpesvirus, coxsackievirus B, and echovirus^31^, fostemsavir incorporating pyrrole effective against HIV^32^, and chloroquine containing quinoline for treatment against SARS coronavirus^33^. Specific functional groups have also demonstrated significant antiviral properties, such as PF-07304814 containing a nitrile group against SARS-CoV-2^34^, and cidofovir containing a phosphate group against varicella zoster virus (VZV), Epstein–Barr virus (EBV), human herpesvirus-6 (HHV-6), human herpesvirus-8 (HHV-8), HPV, polyomaviruses, and orthopoxviruses^35^.

To validate the practical application of our prediction models, we employed both the virus-selective and pan-antiviral models to identify potential anti-SARS-CoV-2 drugs from a library of ~360 K compounds (Fig. 3A). Consensus predictions were generated by combining multiple individual models to leverage their complementary strengths while minimizing their weaknesses. This approach captured diverse aspects of the data that might be overlooked by a single model, thereby improving overall performance^28,36^. In this study, RF combined with XGB for virus-selective models and RF with SVM for pan-antiviral models were used in consensus predictions to enhance accuracy. We further stratified the candidate compounds based on their anti-SARS-CoV-2 potency and structural diversity. First, structural analogs of known AIADs were prioritized, as they are recognized as important leads for anti-SARS-CoV-2 drug discovery^37^. Compounds exhibiting structural similarity (maxTC >0.25) to at least one of the 83 known anti-SARS-CoV-2 compounds in this study were selected for further screening. Second, the challenge of compound cytotoxicity in SARS-CoV-2-infected cells was addressed, as some compounds, such as ponatinib, remain harmless to normal, uninfected cells but become toxic in SARS-CoV-2-infected cells due to immunological changes and/or altered drug metabolism^38–40^. To address this, five robust machine learning classification models were developed to flag anti-SARS-CoV-2 compounds that may be cytotoxic. These models were based on compound outcomes from the cytotoxicity counter screens, conducted in parallel with the SARS-CoV-2 PP entry assay and the live virus CPE assay (Fig. 3B–D). Both the PP entry and CPE assays are cell-based with luminescence readouts^15,41^. The PP entry assay, using pseudotyped viral particles with SARS-CoV-2 Spike proteins, is used to identify viral cell entry inhibitors in biosafety level 2 laboratories. This assay primarily models key mechanisms of viral entry into host cells^42,43^, including: (1) inhibition of the ACE2-RBD interaction, preventing viral attachment; (2) inhibition of S protein processing, such as blocking TMPRSS2- or furin-mediated cleavage, thereby affecting membrane fusion or endocytic entry; (3) inhibition of membrane fusion by interfering with viral-host membrane interactions required for genome release; and (4) inhibition of endocytic entry, for example, by disrupting endosomal acidification or lysosomal protease activity, thereby preventing viral uncoating. The CPE assay is employed to measure the ability of compounds to prevent live SARS-CoV-2-induced cytopathic effects involving viral entry and replication in cells. The cytotoxicity counter screen data from both assays were used to minimize false positives in our cytotoxicity prediction models to enhance the model's robustness in identifying safe and effective anti-SARS-CoV-2 compounds. In the consensus model approach, non-cytotoxic anti-SARS-CoV-2 compounds were identified as compounds predicted to be non-toxic by at least three of the five models, thereby improving prediction reliability. Finally, k-means clustering was employed to ensure structural diversity among the selected compounds, balancing potency with variety to broaden therapeutic possibilities (Fig. 3A). This comprehensive strategy, integrating multiple analytical approaches, aimed to identify structurally diverse, potent, and safe anti-SARS-CoV-2 compounds.

Testing of the predicted anti-SARS-CoV-2 compounds using in vitro assays yielded 24 active PP entry inhibitors and 47 RdRp inhibitors (Supplementary Tables S7,S8). Among these compounds, azeliragon (NCGC00506876) was distinguished as the only compound previously reported to exhibit anti-SARS-CoV-2 activity. As a small molecule antagonist of the receptor for advanced glycation end products (RAGE), azeliragon has demonstrated promising antiviral effects in previous studies, where the internalization of infectious SARS-CoV-2 particles was significantly reduced in monocytes pretreated with 2 μM azeliragon. These results suggested that our method was useful for identifying antiviral compounds. In our study, azeliragon was tested active in the RdRp assay with an IC_50_ of 7.68 ± 0.62 μM (Fig. 5), further supporting its potential as an anti-SARS-CoV-2 agent. In addition, glaziovine (NCGC00408842), a compound not previously associated with SARS-CoV-2, has been reported to be an antiviral against the hepatitis B virus (HBV) by inhibiting hepatitis B surface antigen (HBsAg) secretion with an IC_50_ of 8.0 μM in the Hep G2.2.15 cell line^44^. The potential antiviral spectrum of glaziovine was expanded by our study, with it being identified as active in the RdRp assay with an IC_50_ of 10.49 ± 3.36 μM (Supplementary Table S8), indicating its potential effectiveness against SARS-CoV-2. The antiviral activities of the remaining active compounds identified in either the PP entry or RdRp assays have not been previously reported in the literature. As such, this novel dataset offers a valuable resource for the identification and development of new anti-SARS-CoV-2 drugs, presenting new opportunities for further investigation and drug development efforts.

Several key limitations should be considered when using in vitro assays like the PP entry assay and the RdRp assay to verify model predictions, since our models were trained on antivirals that do not necessarily act through these mechanisms. While the in vitro assays provide valuable tools for initial screening, they may not fully capture the complex interactions between the virus and host in a living organism, as they often target specific viral mechanisms in isolation. For example, the PP entry assay is effective in identifying viral entry inhibitors but may not simulate the complete dynamics of viral infection, as it lacks the full viral genome^41^. Similarly, the RdRp assay, which detects inhibitors of SARS-CoV-2 RNA-dependent RNA polymerase (RdRp) using fluorescent-labeled substrates, focuses solely on viral genome replication and may overlook compounds that act through alternative mechanisms, such as modulating host immune responses or disrupting viral assembly and release^45^. To address these limitations, future studies should include systematic assessments of in vivo antiviral efficacy, alongside comprehensive pharmacokinetic and pharmacodynamic characterizations. Additionally, thorough safety evaluations in relevant animal models are crucial to bridge the gap between model predictions and the development of clinically viable anti-SARS-CoV-2 therapies. Such a holistic approach will be essential for translating these promising in vitro results into effective therapeutic options for treating SARS-CoV-2 infections.

In summary, we developed robust machine learning models to identify virus-selective and pan-antiviral drugs by integrating compound structural information with viral genome sequence data. These models demonstrated robust predictive performance and were applied to a large-scale virtual screening of ~360 K compounds from the NCATS in-house library. Novel anti-SARS-CoV-2 drug candidates were identified using optimal consensus models that combined multiple algorithms, including RF, XGB, and SVM. A stratification process was further applied to the predicted compounds to minimize unwanted cytotoxicity and maximize structural diversity. A total of 24 novel PP entry inhibitors and 47 RdRp activity inhibitors were experimentally confirmed using in vitro assays. Among these, azeliragon demonstrated promising anti-SARS-CoV-2 activity, while others showed potential efficacy against SARS-CoV-2 for the first time. These findings demonstrated the predictive power of our computational models and provided a valuable set of lead compounds for further anti-SARS-CoV-2 drug development. Furthermore, the study’s integrative approach, combining chemical structure analysis, viral genomic data, and advanced machine learning techniques, offers a promising framework for discovering small molecule antivirals targeting both current and emerging viral threats.

## Materials and methods

### Feature vectors for viral genome sequences

Complete viral genome sequences were downloaded as FASTA files from three databases, including the Global Initiative on Sharing All Influenza Data database (GISAID, https://www.gisaid.org/, e.g., SARS-CoV-2 strains/variants), the European Bioinformatics Institute (EBI, https://www.ebi.ac.uk/genomes/virus.html, e.g., HPV-11), and the National Center for Biotechnology Information (NCBI, https://www.ncbi.nlm.nih.gov/genomes/GenomesGroup.cgi?opt=virus&taxid=10239&host=human, e.g., influenza A virus). The sequences were processed using R software with two key packages: “msa” (version 1.28.0) for sequence import and “seqinr” (version 4.2-23) for calculating pairwise alignment distances based on sequence identity. Recognizing the analogy between viral genomes and natural language, the genome sequences were treated as sentences, with k-monomeric units (where *k* = 6) serving as “words”. This analogy allowed us to apply natural language processing techniques to genomic data. Specifically, we employed the FastText embedding model to generate a “continuous bag of nucleobases” representation. This was implemented using the “fastText” package in R, with the following parameters: 100 epochs, a “softmax” loss function, a learning rate of 0.1 (default), and a word vector size of 100 (default). This approach converted viral genomes of varying lengths into uniform 100-dimensional feature vectors (real values), suitable for machine learning-based predictive analysis.

### Collection of approved and investigational antiviral drugs (AIADs)

The collection of AIADs was conducted in two main steps: initial identification and selection from databases, followed by systematic verification through literature review. First, compounds were sourced from the NCATS in-house collection of antivirals and the DrugBank database (https://go.drugbank.com/), ensuring a diverse and comprehensive representation of both approved and investigational antiviral compounds. Particular emphasis was placed on potential anti-SARS-CoV-2 compounds that had progressed to phase III clinical trials, reflecting their advanced status in the drug development pipeline. Other antiviral agents were included based on demonstrated efficacy against a range of viral pathogens across all clinical trial phases. Second, the antiviral activity of each compound was systematically verified through PubMed literature searches (https://pubmed.ncbi.nlm.nih.gov/) using a standardized keyword-based strategy, combining the compound name with terms such as “antiviral,” “antiviral activity,” and “viral inhibition.” Notably, the selected antiviral compounds target a wide spectrum of viral and host proteins involved in various stages of the viral lifecycle, including but not limited to viral entry, replication, assembly, and host immune modulation. Details of the AIADs collected were provided in Supplementary Table S2.

### Collection of non-cytotoxic pharmaceutical compounds (NCPCs) based on Tox21 cell viability assay data

The NCATS Pharmaceutical Collection (NPC)^46^, consisting of ~3000 approved and investigational drugs, was systematically evaluated for cytotoxicity using in vitro cell-based high-throughput screening (HTS) assays as part of the Tox21 program. Detailed data and descriptions of these assays were accessed through the NCATS Tox21 public data browser (https://tripod.nih.gov/pubdata/). Each compound was screened in triplicate at 15 distinct concentrations, with activity quantified using a curve rank ranging from −9 to 9^47^. Negative values (−9 to −1) indicated decreasing inhibitory activity, while positive values (1 to 9) indicated increasing activation activity. A curve rank of 0 signified inactivity. Compounds were classified as NCPCs if they exhibited a curve rank of 0 in at least 30 of the 55 cell viability assays. This threshold ensured the selection of compounds demonstrating minimal cytotoxic effects across a diverse range of cellular models. Details of NCPCs based on Tox21 cell viability assay data were provided in Supplementary Table S4.

### Collection of anti-SARS-CoV-2 pharmaceutical compounds based on the PP and cytopathic effect (CPE) assays

We identified potentially cytotoxic and non-cytotoxic anti-SARS-CoV-2 compounds using cell viability counter screen data for both the PP and CPE assays based on our previous study^41^. For modeling purposes, compounds were designated as cytotoxic (assigned a value of 1) if they met the following criteria: AC_50_ <10 µM, efficacy <−50%, and curve rank <−1 in the cell viability counter screen. Compounds that did not meet these criteria were designated as non-cytotoxic (assigned a value of 0). To ensure a conservative cytotoxicity assessment, an additional rule was applied: any compound exhibiting cytotoxicity in either the PP or CPE cell viability counter screen was classified as cytotoxic. Conversely, compounds that did not exhibit cytotoxicity in either assay were designated as non-cytotoxic. By prioritizing compounds with both efficacy against SARS-CoV-2 and minimal cytotoxicity, this approach enhances the potential for identifying potential anti-SARS-CoV-2 candidates with favorable safety profiles.

### Conversion of chemical structures to fingerprints and structural analysis

Chemical structures of compounds were converted to two types of commonly used structure fingerprints, i.e., extended connectivity fingerprints radius 4 (ECFP4) and ToxPrint. ECFP4 was used to build classification models and evaluate the structural similarities between compounds, while ToxPrint was used to identify chemical structural features significantly enriched in AIAD compounds. Molecular structures encoded in SMILES (simplified molecular input line entry system) were converted into ECFP4, which represents chemical compounds as 1024-bit binary vectors, where each bit indicates the presence (1) or absence (0) of a specific structural feature. This conversion was performed using the Chemistry Development Kit (CDK) integrated into the Konstanz Information Miner (KNIME) platform, version 4.7.1. Structural similarity between compounds was assessed by calculating the Tanimoto coefficient (TC) based on their ECFP4 fingerprints. The TC, which ranges from 0 to 1, measures similarity by dividing the number of shared structural features by the total number of features present in either compound. For each compound, its closest structural neighbor was identified by calculating the TC between it and all other compounds in the set, with the highest TC value defined as maxTC. This maxTC value was used to assess the structural similarity between compound sets. The ToxPrint fingerprints (729 bits) were generated using the publicly available ChemoTyper application (https://chemotyper.org/). Fisher’s exact test was used to determine the significance of structure features enriched in AIADs or NCPCs based on the ToxPrint fingerprints, and a *p* value <0.05 was considered statistically significant.

### Implementation and evaluation of machine learning classification models

The machine learning modeling process was performed following methodologies in our previous studies^48–53^. Virus-selective models were built using a combination of chemical structural features (ECFP4 fingerprints) and viral genome sequence descriptors. Pan-antiviral models were built using chemical structural features only. The dataset was randomly split into a training set (70%) and a testing set (30%), with this process repeated 20 times to ensure robustness and mitigate sampling bias. Here, “the dataset” refers to the data used to build each individual model. Five classification models were built: Naïve Bayes (NB) and SVM using the “e1071” package, neural networks (NNET) with the “nnet” package, RF via the “Random Forest” package, and XGB using the “xgboost” package. Laplace smoothing was applied to the NB classifier to address zero probability issues, while the SVM classifier employed a Gaussian radial basis function kernel. Default parameters were maintained for the RF and NNET classifiers. In the XGB model, parameters were set to include a maximum tree depth of 3, a learning rate of 0.01, and a subsample ratio of 0.5 for constructing each tree. Model performance was evaluated using area under the receiver operating characteristic curve (AUC-ROC) and balanced accuracy (BA) via the “pROC” package, and Matthews correlation coefficient (MCC) computed using the “mltools” package. The entire machine learning modeling procedure was executed in R version 4.2.1.

### Feature selection and data rebalance for machine learning model optimization

Feature selection was performed using four methods to identify the most informative features for machine learning model construction, with slight modifications to previously described approaches^48–53^. In Fisher’s exact test and *t*-test method, *p* value thresholds ranging from 0.01 to 0.05 in increments of 0.01 were utilized. The AUC-ROC method was implemented with cutoff thresholds from 0.52 to 0.56 in increments of 0.02, using the “pROC” package in R. For the Random Forest (RF) and eXtreme gradient boosting (XGB) methods, feature importance scores, such as Gini importance or Gain scores, were calculated using the “Random Forest” and “xgboost” packages, respectively. Features were selected at intervals from the top 20 to the top 100 ranked features. To address data imbalance, four sampling methods were employed: down-sampling, up-sampling, random over-sampling examples (ROSE), and synthetic minority over-sampling technique (SMOTE), utilizing the “ROSE” and “DMwR” packages in R. This comprehensive approach to feature selection and data balancing was designed to enhance the robustness and reliability of the machine learning models, improving their predictive power for identifying effective antiviral compounds.

### SARS-CoV-2 pseudotyped particle (PP) entry assay

The PP entry assay was performed according to previously described protocols^43^. In brief, SARS-CoV-2 Spike protein containing PPs, along with control PPs (vesicular stomatitis virus glycoprotein PP and bald PP), were custom-produced by Dexorgen (Rockville, MD). The assay was conducted in HEK293 cells expressing human angiotensin-converting enzyme 2 (HEK293-ACE2) under biosafety level 2 (BSL-2) conditions. Compounds were tested in 11-point, 1:3 serial dilutions starting from a concentration of 57.5 μM. After 48 h of incubation at 37 °C with 5% CO₂, luciferase activity was measured using the bright-glo luciferase assay (Promega) to assess the PP entry. Data were normalized to wells with SARS-CoV-2 spike PPs (100%) and bald PPs (0%). Cytotoxicity was evaluated in parallel using an intracellular ATP assay without the addition of PPs, with cells and media as 100 and 0% controls, respectively. The dual assessments provided a comprehensive evaluation of both compound efficacy in inhibiting viral entry and their potential cytotoxicity. All compound libraries used in the study were assembled by the National Center for Advancing Translational Sciences (NCATS), ensuring high consistency and quality control throughout the screening process.

### RNA-dependent RNA polymerase (RdRp) assay

The SARS-CoV-2 RdRp assay in the time-resolved fluorescence resonance energy transfer (TR-FRET) assay format was obtained from BPS Bioscience (San Diego, CA). The assay was optimized for high-throughput screening in a 1536-well plate format. Complete RdRp buffer was prepared by adding 10 µL of 0.5 M DTT to 5 mL of RdRp assay buffer component 1, followed by the addition of 20 µL of RdRp assay buffer component 2 (based on the manufacturer’s protocol). The RNAse inhibitor was then diluted 8-fold in the prepared complete RdRp buffer. The RdRp enzyme was diluted in the complete RdRp buffer to a final concentration of 60 ng/µL, ensuring that the enzyme was not refrozen after dilution. The RdRp reaction mixture was prepared by diluting the digoxigenin-labeled RNA duplex and biotinylated ATP 50-fold in the complete RdRp buffer. The enzyme mix was assembled for the 1536-well plate according to the following volumes per well: 0.5 µL of complete RdRp buffer, 1 µL of RdRp enzyme for test samples (no enzyme for blanks), 0.5 µL of RNAse inhibitor, and 0.5 µL of the RdRp reaction mixture, yielding a total volume of 2.5 µL per well. For each 1536-well plate, a 5 mL reaction mix needs to be prepared. Subsequently, 5 µL of the enzyme mix was dispensed into each well of the 1536-well plate. Test compounds were added by pintool, dispensing 23 nL of each compound into the respective wells, and the plate was incubated at 37 °C for 3 h. During the incubation period, the TR-FRET detection buffer was thawed on ice. Eu-labeled antibody was diluted to 1:600, and dye-labeled acceptor was diluted to 1:200, with 8.3 and 25 µL of each, respectively, added to 5 mL of the detection buffer. Following the incubation, 5 µL of the prepared detection solution was added to each well, and the plate was shaken on a rotator at room temperature for 20 minutes. Fluorescence intensity was measured using the BMG PHERAstar plate reader in the HTRF format with excitation at 317 nm and dual emissions at both 620 and 665 nm. For the 620 nm channel, a lag time of 60 µs and an integration time of 500 µs were set, and similar settings were applied for the 665 nm channel. Due to the difficulty in reading the 620 nm emission simultaneously on the 1536-well plate format, the primary readout was obtained at 665 nm. The focal height for optimal 665 nm reading was adjusted to 10 nm. This method enabled the high-throughput screening of RdRp inhibitors using a 1536-well plate format, facilitating the rapid and sensitive detection of fluorescence signals via TR-FRET.

### Virtual screening and validation using in vitro assays

The optimal machine learning models were applied to screen the NCATS in-house collection of ~360 K diverse compounds. These compounds included known bioactive compounds and new small molecules designed for drug discovery purposes. Consensus predictions from multiple individual models were utilized to enhance reliability and accuracy. The consensus score for each compound was calculated as the sum of its probability scores from multiple models, weighted by the respective AUC-ROC values of each model. To ensure the selection of structurally diverse candidate compounds for validation using in vitro assays, molecules predicted as positive hits by multiple models were further stratified through clustering based on structural similarity using the k-means algorithm. This approach partitioned compounds into k distinct clusters, facilitating the selection of representative candidates spanning diverse chemical scaffolds. Compounds achieving the highest consensus scores within their respective clusters were prioritized for testing using in vitro assays. To analyze the validation results, concentration-response curves were fitted using four-parameter logistic regression, where % assay activity was the response variable, and log10 compound concentration served as the independent variable. This analysis was conducted using the “drc” statistical package in R. Data visualizations were generated through the “ggplot2” package in R, and representative chemical structures were rendered using ChemDraw Professional software (version 23.1.1). The comprehensive workflow, including data collection, data processing, model building, model evaluation, virtual screening, and validation using in vitro assays, is illustrated in Fig. S1.

## Supplementary information

Supplementary Information

## Data Availability

All data supporting the findings of this study are included in the manuscript, supplementary information files, and the machine learning model input data are available on GitHub at https://github.com/TX-2017/antivirals_prediction.

## Code availability

The code is available on GitHub at https://github.com/TX-2017/machine-learning.

## References

[1] Morens, D. M. & Fauci, A. S. Emerging pandemic diseases: how we got to COVID-19. *Cell* **182**, 1077–1092 (2020). 32846157 10.1016/j.cell.2020.08.021 PMC7428724

[2] Gates, B. Responding to Covid-19—a once-in-a-century pandemic? *N. Engl. J. Med.* **382**, 1677–1679 (2020). 32109012 10.1056/NEJMp2003762

[3] Madhav, N. et al. *Disease Control Priorities: Improving Health and Reducing Poverty* (eds Mock, C. N. et al.) Ch. 17 (World Bank Publications, 2017). 30212058

[4] Faramarzi, A. et al. The global economic burden of COVID-19 disease: a comprehensive systematic review and meta-analysis. *Syst. Rev.* **13**, 68 (2024). 38365735 10.1186/s13643-024-02476-6 PMC10870589

[5] Organization, W. H. WHO coronavirus (COVID-19) dashboard. https://covid19.who.int (2024).

[6] Clutter, D. S., Jordan, M. R., Bertagnolio, S. & Shafer, R. W. HIV-1 drug resistance and resistance testing. *Infect. Genet. Evol.* **46**, 292–307 (2016). 27587334 10.1016/j.meegid.2016.08.031 PMC5136505

[7] Balmith, M., Faya, M. & Soliman, M. E. Ebola virus: a gap in drug design and discovery‐experimental and computational perspective. *Chem. Biol. Drug Des.* **89**, 297–308 (2017). 27637475 10.1111/cbdd.12870

[8] Shariff, M. Nipah virus infection: a review. *Epidemiol. Infect.* **147**, e95 (2019). 30869046 10.1017/S0950268819000086 PMC6518547

[9] Hughes, J. P., Rees, S., Kalindjian, S. B. & Philpott, K. L. Principles of early drug discovery. *Br. J. Pharmacol.* **162**, 1239–1249 (2011). 21091654 10.1111/j.1476-5381.2010.01127.x PMC3058157

[10] Lavecchia, A. Machine-learning approaches in drug discovery: methods and applications. *Drug Discov. Today* **20**, 318–331 (2015). 25448759 10.1016/j.drudis.2014.10.012

[11] Yasir, M., Tripathi, A. S., Tripathi, M. K., Shukla, P. & Maurya, R. K. in *CADD and Informatics in Drug Discov*ery (eds Rudrapal, M. & Khan, J.) (Springer, 2023).

[12] Onawole, A. T., Sulaiman, K. O., Kolapo, T. U., Akinde, F. O. & Adegoke, R. O. COVID-19: CADD to the rescue. *Virus Res.* **285**, 198022 (2020). 32417181 10.1016/j.virusres.2020.198022 PMC7228740

[13] Vamathevan, J. et al. Applications of machine learning in drug discovery and development. *Nat. Rev. Drug Discov.* **18**, 463–477 (2019). 30976107 10.1038/s41573-019-0024-5 PMC6552674

[14] Kc, G. B. et al. A machine learning platform to estimate anti-SARS-CoV-2 activities. *Nat. Mach. Intell.* **3**, 527–535 (2021).

[15] Xu, T. et al. Efficient identification of anti-SARS-CoV-2 compounds using chemical structure-and biological activity-based modeling. *J. Med. Chem.* **65**, 4590–4599 (2022). 35275639 10.1021/acs.jmedchem.1c01372 PMC8936051

[16] Huang, R. et al. Biological activity-based modeling identifies antiviral leads against SARS-CoV-2. *Nat. Biotechnol.* **39**, 747–753 (2021). 33623157 10.1038/s41587-021-00839-1 PMC9843700

[17] Adeshina, Y. O., Deeds, E. J. & Karanicolas, J. Machine learning classification can reduce false positives in structure-based virtual screening. *Proc. Natl Acad. Sci. USA.* **117**, 18477–18488 (2020). 32669436 10.1073/pnas.2000585117 PMC7414157

[18] Zhang, L., Tan, J., Han, D. & Zhu, H. From machine learning to deep learning: progress in machine intelligence for rational drug discovery. *Drug Discov. Today* **22**, 1680–1685 (2017). 28881183 10.1016/j.drudis.2017.08.010

[19] Blassel, L. et al. Using machine learning and big data to explore the drug resistance landscape in HIV. *PLoS Comput. Biol.* **17**, e1008873 (2021). 34437532 10.1371/journal.pcbi.1008873 PMC8425536

[20] Zeng, X. et al. deepDR: a network-based deep learning approach to in silico drug repositioning. *Bioinformatics* **35**, 5191–5198 (2019). 31116390 10.1093/bioinformatics/btz418 PMC6954645

[21] Petrova, V. N. & Russell, C. A. The evolution of seasonal influenza viruses. *Nat. Rev. Microbiol.* **16**, 47–60 (2018). 29081496 10.1038/nrmicro.2017.118

[22] Angioni, R. et al. RAGE engagement by SARS-CoV-2 enables monocyte infection and underlies COVID-19 severity. *Cell Rep. Med*. **4**, 101266 (2023). 10.1016/j.xcrm.2023.101266 PMC10694673 37944530

[23] Karim, M., Lo, C.-W. & Einav, S. Preparing for the next viral threat with broad-spectrum antivirals. *J. Clin. Invest*. **133**, e170236 (2023). 10.1172/JCI170236 PMC10232003 37259914

[24] Gorbalenya, A. E. & Lauber, C. Bioinformatics of virus taxonomy: foundations and tools for developing sequence-based hierarchical classification. *Curr. Opin. Virol.* **52**, 48–56 (2022). 34883443 10.1016/j.coviro.2021.11.003

[25] Deepthi, K., Jereesh, A. & Liu, Y. A deep learning ensemble approach to prioritize antiviral drugs against novel coronavirus SARS-CoV-2 for COVID-19 drug repurposing. *Appl. Soft Comput.* **113**, 107945 (2021). 34630000 10.1016/j.asoc.2021.107945 PMC8492370

[26] Vigant, F., Santos, N. C. & Lee, B. Broad-spectrum antivirals against viral fusion. *Nat. Rev. Microbiol.* **13**, 426–437 (2015). 26075364 10.1038/nrmicro3475 PMC4554337

[27] Strasfeld, L. & Chou, S. Antiviral drug resistance: mechanisms and clinical implications. *Infect. Dis. Clin. North Am.* **24**, 413–437 (2010). 20466277 10.1016/j.idc.2010.01.001 PMC2871161

[28] Xu, T., Xia, M. & Huang, R. *in Machine Learning and Deep Learning in Computational Toxicology* (ed. Hong, H.) Ch. 19 (Springer, 2023).

[29] Speck-Planche, A. & Kleandrova, V. V. Multi-condition QSAR model for the virtual design of chemicals with dual pan-antiviral and anti-cytokine storm profiles. *ACS omega* **7**, 32119–32130 (2022). 36120024 10.1021/acsomega.2c03363 PMC9476185

[30] Eyer, L. et al. Nucleoside analogs as a rich source of antiviral agents active against arthropod-borne flaviviruses. *Antivir. Chem. Chemother.* **26**, 2040206618761299 (2018). 29534608 10.1177/2040206618761299 PMC5890575

[31] Starčević, K. et al. Synthesis, antiviral and antitumor activity of 2-substituted-5-amidino-benzimidazoles. *Bioorg. Med. Chem.* **15**, 4419–4426 (2007). 17482821 10.1016/j.bmc.2007.04.032

[32] Bianco, M. D. C. A. D., Marinho, D. I. L. F., Hoelz, L. V. B., Bastos, M. M. & Boechat, N. Pyrroles as privileged scaffolds in the search for new potential HIV inhibitors. *Pharmaceuticals* **14**, 893 (2021). 34577593 10.3390/ph14090893 PMC8468532

[33] Vincent, M. J. et al. Chloroquine is a potent inhibitor of SARS coronavirus infection and spread. *Virol. J.* **2**, 1–10 (2005). 16115318 10.1186/1743-422X-2-69 PMC1232869

[34] Bai, B. et al. Peptidomimetic nitrile warheads as SARS-CoV-2 3CL protease inhibitors. *RSC Med. Chem.* **12**, 1722–1730 (2021). 34778773 10.1039/d1md00247c PMC8529539

[35] Paintsil, E. & Cheng, Y.-C. Antiviral agents. *Encyclopedia Microbiol.* 176–225 (2019).

[36] Xu, T., Ngan, D. K. & Huang, R. in *QSAR in Safety Evaluation and Risk Assessment* (ed. Hong, H.) Ch. 18 (Elsevier, 2024).

[37] Hasan, M. K. et al. Structural analogues of existing anti-viral drugs inhibit SARS-CoV-2 RNA dependent RNA polymerase: a computational hierarchical investigation. *Heliyon* **7**, e06435 (2021). 10.1016/j.heliyon.2021.e06435 PMC7934700 33693066

[38] Levy, M. Role of viral infections in the induction of adverse drug reactions. *Drug Saf.* **16**, 1–8 (1997). 9010640 10.2165/00002018-199716010-00001

[39] Carvalhal, F. et al. Evaluation of the cytotoxic and antiviral effects of small molecules selected by in silico studies as inhibitors of SARS-CoV-2 cell entry. *Molecules* **28**, 7204 (2023). 37894682 10.3390/molecules28207204 PMC10609270

[40] Yuan, C. et al. The role of cell death in SARS-CoV-2 infection. *Signal Transduct. Target. Ther.* **8**, 357 (2023). 37726282 10.1038/s41392-023-01580-8 PMC10509267

[41] Xu, T., Zheng, W. & Huang, R. High‐throughput screening assays for SARS‐CoV‐2 drug development: current status and future directions. *Drug Discov. Today* **26**, 2439–2444 (2021). 34048893 10.1016/j.drudis.2021.05.012 PMC8146264

[42] Nie, J. et al. Quantification of SARS-CoV-2 neutralizing antibody by a pseudotyped virus-based assay. *Nat. Protoc.* **15**, 3699–3715 (2020). 32978602 10.1038/s41596-020-0394-5

[43] Xu, M. et al. A high throughput screening assay for inhibitors of SARS-CoV-2 pseudotyped particle entry. *SLAS Discov.* **27**, 86–94 (2022). 35086793 10.1016/j.slasd.2021.12.005 PMC8720380

[44] Cheng, P. et al. Two new alkaloids and active anti-hepatitis B virus constituents from Hypserpa nitida. *Bioorg. Med. Chem. Lett.* **17**, 5316–5320 (2007). 17723297 10.1016/j.bmcl.2007.08.027

[45] Zhu, W. et al. RNA-dependent RNA polymerase as a target for COVID-19 drug discovery. *SLAS Discov.* **25**, 1141–1151 (2020). 32660307 10.1177/2472555220942123 PMC7684788

[46] Gorshkov, K. et al. Quantitative chemotherapeutic profiling of gynecologic cancer cell lines using approved drugs and bioactive compounds. *Transl. Oncol.* **12**, 441–452 (2019). 30576957 10.1016/j.tranon.2018.11.016 PMC6302136

[47] Huang, R. A quantitative high-throughput screening data analysis pipeline for activity profiling. *Methods Mol. Biol.* **2474**, 133–145 (2022). 35294762 10.1007/978-1-0716-2213-1_13 PMC9540341

[48] Xu, T. et al. Predictive models for human organ toxicity based on in vitro bioactivity data and chemical structure. *Chem. Res. Toxicol.* **33**, 731–741 (2020). 32077278 10.1021/acs.chemrestox.9b00305 PMC10926239

[49] Xu, T., Wu, L., Xia, M., Simeonov, A. & Huang, R. Systematic identification of molecular targets and pathways related to human organ level toxicity. *Chem. Res. Toxicol.* **34**, 412–421 (2020). 33251791 10.1021/acs.chemrestox.0c00305 PMC9460956

[50] Ye, L. et al. Prediction of drug-induced liver injury and cardiotoxicity using chemical structure and in vitro assay data. *Toxicol. Appl. Pharmacol.* **454**, 116250 (2022). 36150479 10.1016/j.taap.2022.116250 PMC9561045

[51] Xu, T. et al. Identification of potent and selective acetylcholinesterase/butyrylcholinesterase inhibitors by virtual screening. *J. Chem. Inf. Model.* **63**, 2321–2330 (2023). 37011147 10.1021/acs.jcim.3c00230 PMC10688023

[52] Xu, T. et al. Predictive models for human cytochrome P450 3A7 selective inhibitors and substrates. *J. Chem. Inf. Model.* **63**, 846–855 (2023). 36719788 10.1021/acs.jcim.2c01516 PMC10664139

[53] Wei, Z. et al. Use of in vitro methods combined with in silico analysis to identify potential skin sensitizers in the Tox21 10K compound library. *Front. Toxicol.* **6**, 1321857 (2024). 38482198 10.3389/ftox.2024.1321857 PMC10933113

