# Supplementary material for: Leveraging viral genome sequences and machine learning models for identification of potentially selective antiviral agents
Source: Commun Chem. 2025 Jun 20;8:189. doi: 10.1038/s42004-025-01583-2 (PMC12181400; doi:10.1038/s42004-025-01583-2)
Supplement: Supplementary file 1 — Supplementary Information [file 42004_2025_1583_MOESM1_ESM.pdf]

Supplementary Materials

Leveraging Viral Genome Sequences and Machine Learning Models for Identification of Potentially Selective Antiviral Agents

Tuan Xu, Miao Xu, Qi Zhang, Catherine Z. Chen, Wei Zheng, Ruili Huang*
Division of Pre-clinical Innovation, National Center for Advancing Translational Sciences (NCATS), National Institutes of Health (NIH), Rockville, Maryland 20850, United States

*Contact information for the corresponding author:

Email: huangru@mail.nih.gov

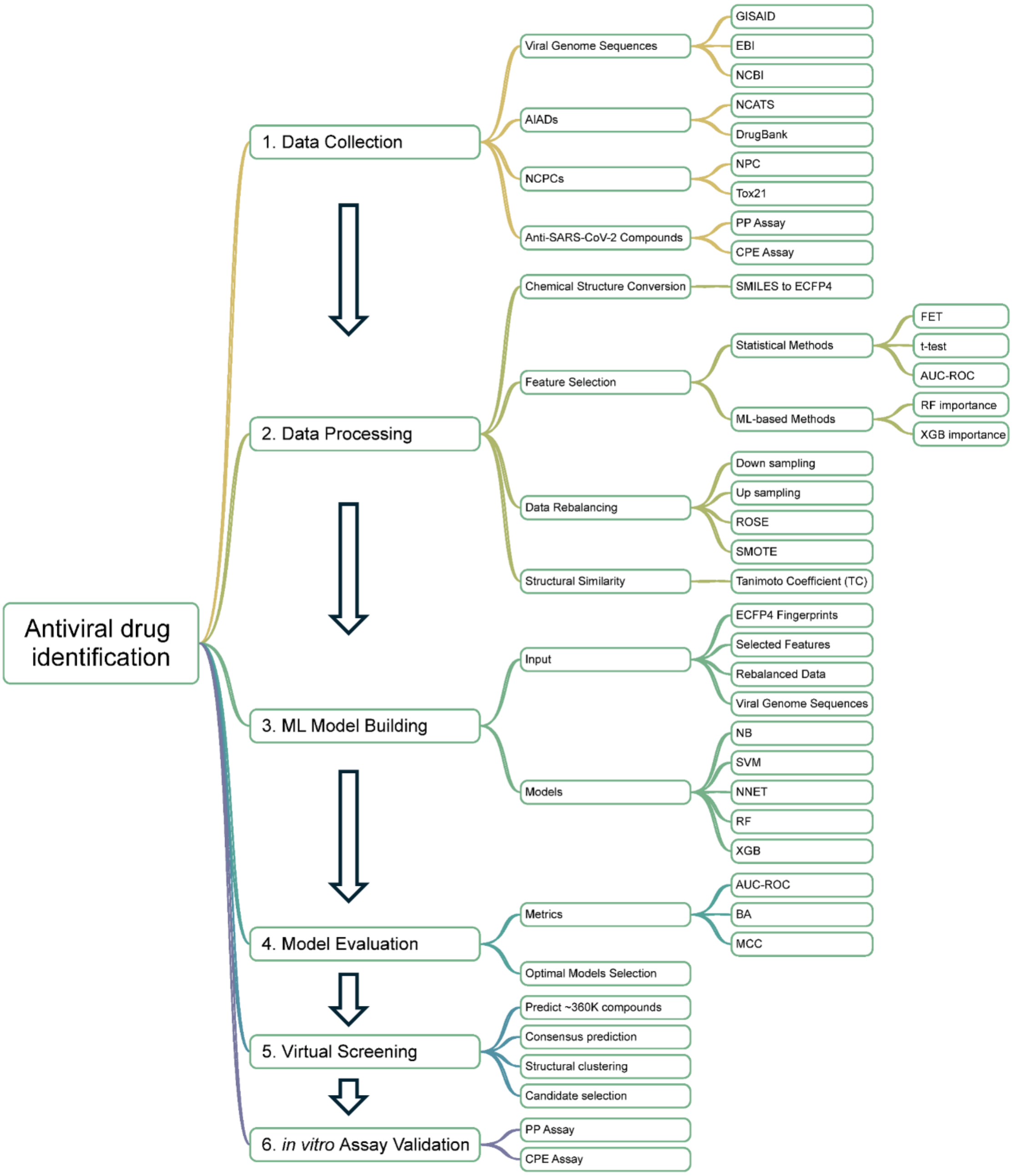

Figure S1. Comprehensive Workflow of the Study.

Table S1. Genomic information for 32 virus strains/variants

Virus strain	Abbreviation	Genome length (nt)	Accession	Database
Influenza A virus (A/California/07/2009(H1N1))	H1N1	13158	NC_026438	NCBI
Influenza A virus (A/New York/392/2004(H3N2))	H3N2	13627	NC_007373	NCBI
Influenza A virus (A/Goose/Guangdong/1/96(H5N1))	H5N1	13590	NC_007357	NCBI
Influenza A virus (A/Shanghai/02/2013(H7N9))	H7N9	13191	NC_026422	NCBI
Influenza A virus(A/Hong Kong/1073/99(H9N2))	H9N2	13498	NC_004910	NCBI
Hepatitis B virus	HBV	3182	NC_003977	NCBI
Severe acute respiratory syndrome coronavirus 2	hCoV-19	29903	NC_045512	NCBI
hCoV-19/USA/MT-MTPHL-3824373/2021	hCoV-19-Alpha	29763	EPI_ISL_3505398	GISAID
hCoV-19/Australia/QLD1892/2021	hCoV-19-Beta	29226	EPI_ISL_2274248	GISAID
hCoV-19/Brazil/SP-SEQ03805/2021	hCoV-19-Delta	29806	EPI_ISL_4273442	GISAID
hCoV-19/Chile/LL-UACH-00141/2021	hCoV-19-Gamma	29857	EPI_ISL_4261457	GISAID
hCoV-19/Peru/LIM-INS-2986/2021	hCoV-19-Lambda	29845	EPI_ISL_3670639	GISAID
hCoV-19/Chile/CO-PUC_MVL_1487/2021	hCoV-19-Mu	29781	EPI_ISL_4413327	GISAID
Hepatitis C virus genotype 1	HCV-1	9646	NC_004102	NCBI
Hepatitis C virus genotype 2	HCV-2	9711	NC_009823	NCBI
Hepatitis C virus genotype 3	HCV-3	9456	NC_009824	NCBI
Hepatitis C virus genotype 4	HCV-4	9355	NC_009825	NCBI
Hepatitis C virus genotype 5	HCV-5	9343	NC_009826	NCBI
Hepatitis C virus genotype 6	HCV-6	9628	NC_009827	NCBI
Hepatitis C virus genotype 7	HCV-7	9443	NC_030791	NCBI
Hepatitis C virus (isolate H77)	HCV-H77	9599	NC_038882	NCBI
Human alphaherpesvirus 3	HHV-3	124884	NC_001348	NCBI
Human herpesvirus 5	HHV-5	235646	NC_006273	NCBI
Human immunodeficiency virus 1	HIV-1	9181	NC_001802	NCBI
Human papillomavirus type 11	HPV-11	7931	M14119	EBI
Human papillomavirus type 6b	HPV-6B	7902	NC_001355	NCBI
Human alphaherpesvirus 1	HSV-1	152222	NC_001806	NCBI
Human alphaherpesvirus 2	HSV-2	154675	NC_001798	NCBI
Influenza B virus RNA 1	Influenza B	14452	NC_002204	NCBI
Variola virus	VARV-major	185578	NC_001611	NCBI
Influenza A virus (A/Korea/426/1968(H2N2))	H2N2	13460	NC_007378	NCBI
Coronavirus Omicron variant (hCoV-19/USA/MS-UMMC-M38E1-523206/2021)	Omicron	29784	EPI_ISL_8901818	GISAID

Table S2. Approved and investigational antiviral drugs targeting 32 virus strains/variants

Corresp onding virus strain	Dru gBa nk.I D	Name	Text	pm id
HIV	DB 004 95	3'-azido-3'-deoxythy midine	The antiviral activities of eight nucleoside analog antiviral drugs (ribavirin, acyclovir, lamivudine, 3'-azido-3'-deoxythymidine, emtricitabine, tenofovir, penciclovir and ganciclovir) against human parainfluenza virus type 2 (hPIV-2) were investigated.	251 544 65
HBV	DB 010 48	abacavir	Antiviral efficacy of abacavir in antiretroviral therapy-experienced adults harbouring HIV-1 with specific patterns of resistance to nucleoside reverse transcriptase inhibitors.	150 405 35
HIV	DB 010 48	abacavir	Antiviral efficacy of abacavir in antiretroviral therapy-experienced adults harbouring HIV-1 with specific patterns of resistance to nucleoside reverse transcriptase inhibitors.	150 405 35
HCV	DB 151 56	abt-072	ABT-072 is a non-nucleoside HCV NS5B polymerase inhibitor that was discovered as part of a program to identify new direct-acting antivirals (DAAs) for the treatment of HCV infection.	293 423 58
HCV	DB 002 84	acarbose	Furthermore, miglitol (a derivative of 1-deoxynojirimycin) and acarbose, approved type-II diabetes drugs that target alpha-glucosidase, have also shown antiviral properties to Filoviruses and Flaviviruses.	333 520 46
HBV	DB 003 16	acetamin ophen	It is noteworthy that several NSAIDs have antiviral activity, namely, aspirin, ibuprofen, naproxen, acetaminophen and lornoxicam, being able to potently inhibit the entry of Zika virus into the cells.	352 699 97
SARS-CoV-2	DB 008 19	acetazola mide	For example, co-administration of HCQ with several antiviral agents including lopinavir-ritonavir, darunavir-cobicistat, and acetazolamide, resulted in QT-interval prolongation, ventricular arrhythmias, and torsade de pointes.	358 890 04
SARS-CoV-2	DB 061 51	acetylcys teine	N-acetylcysteine as a potential treatment for COVID-19.	326 626 64
HIV	DB 009 45	acetylsali cylic acid	In addition, even if NSAIDs are not considered as antiviral agents, provocative evidence not yet verified by clinical scrutiny suggests antiviral properties for NSAID molecules, including ibuprofen, indomethacin and acetylsalicylic acid (ASA).	333 227 33
HHV	DB	acyclovir	[Acyclovir (antiviral)].	364

	007 87			574 0
HSV	DB 007 87	acyclovir	[Acyclovir (antiviral)].	364 574 0
HCV	DB 149 06	adafosbu vir	Adafosbuvir (also known as AL-335) is a pro-drug of a uridine-based nucleotide analog polymerase (NS5B) inhibitor with potent antiviral activity against HCV GTs 1-6.	320 653 30
HBV	DB 138 68	adefovir	A novel baseline hepatitis B virus sequencing-based strategy for predicting adefovir antiviral response.	259 830 54
HHV	DB 007 18	adefovir dipivoxil	Effect of Hepatitis B Virus Genotypes on the Efficacy of Adefovir Dipivoxil Antiviral Therapy	252 373 70
HBV	DB 007 18	adefovir dipivoxil	Effect of Hepatitis B Virus Genotypes on the Efficacy of Adefovir Dipivoxil Antiviral Therapy	252 373 70
HIV	DB 005 18	albendaz ole	Albendazole reduces viral loads and increases CD4 counts and hemoglobin levels in pregnant, HIV-1??????nfected women with helminth infections receiving antiretroviral therapy.	252 100 19
Influenz a A	DB 009 15	amantadi ne	Antiviral drugs like adamantane derivatives (amantadine and rimantadine) and neuraminidase inhibitors (oseltamivir and zanamivir) are used for treating influenza infection.	271 100 56
HCV	DB 009 15	amantadi ne	Antiviral drugs like adamantane derivatives (amantadine and rimantadine) and neuraminidase inhibitors (oseltamivir and zanamivir) are used for treating influenza infection.	271 100 56
HSV	DB 004 79	amikacin	Moreover, the possibility of exploiting the antiviral activity of these antibiotics against SARS-CoV-2 was evaluated, but the ability of fluoroquinolones (such as levofloxacin) to suppress SARS-CoV-2 and MERS-CoV replication in cultured cells has been proven limited, the effectiveness of aminoglycosides (such as gentamicin and amikacin) against SARS-CoV-2 due to production of retrocyclins, and the binding of meropenem to SARS-CoV-2 protease still need to be demonstrated.	345 716 99
SARS-CoV-2	DB 003 81	amlodipi ne	In this study, we tested a panel of antihypertensive drugs that are in clinical use and found that the CCBs benidipine HCI and amlodipine besylate have significant antiviral effect in vitro.	333 496 33
SARS-CoV-2	DB 010	amoxicill in	In addition to antiviral agents, antibiotics such as amoxicillin, azithromycin or fluoroquinolones are also being employed in an attempt to eradicate the SARS-CoV-2 virus.	341 438

	60			18
HIV	DB 004 15	ampicilli n	Lumbar puncture was performed and empiric intravenous antibiotics and an antiviral (vancomycin, ceftriaxone, ampicillin, and acyclovir) were initiated.	339 945 37
HIV	DB 007 01	amprena vir	No antiviral activity against SARS-CoV-2 was demonstrated for the HIV protease inhibitors darunavir, amprenavir, saquinavir, neither indinavir on Vero E6 and Vero E6/TMPRSS2, although indinavir has been predicting to bind with the SARS-CoV-2 PLpro.	347 555 38
HIV	DB 064 97	aplaviroc	Several CCR5-binding compounds including Maraviroc, Vicriviroc, Aplaviroc, TAK-779, SCH-C and CMPD 167 antagonize this process and have a strong antiviral activity against HIV-1 in vitro.	224 247 37
Influenz a A	DB 136 09	arbidol	Umifenovir (Arbidol) - antiviral drug against influenza viruses	291 624 76
SARS-CoV-2	DB 002 78	argatroba n	Argatroban also possesses anti-inflammatory and antiviral activities and has a well-established pharmacokinetics profile.	328 704 33
SARS-CoV-2	DB 066 97	artemeth er	At present, there is evidence demonstrating the antiviral activity of artemisinin and some of its derivatives such as artesunate, artemether, and dihydroartemisinin against SARS-CoV-2, from in vitro cell-based assays, to animal models and clinical trials, either alone or in combination with other therapeutic agents.	358 453 50
SARS-CoV-2	DB 116 38	artenimo l	Characterization of the antiviral effects of artenimol and omacetaxine mepesuccinate	347 124 00
HHV	DB 092 74	artesunat e	Since the antimalarial drug artesunate was recently demonstrated to have antiviral activity, the possible effects of artesunate on BKV replication in human primary renal proximal tubular epithelial cells (RPTECs), the host cells in PyVAN, were explored.	241 455 49
HCV	DB 115 86	asunapre vir	Asunaprevir (BMS-650032) is a potent hepatitis C virus (HCV) NS3 protease inhibitor demonstrating efficacy in alfa interferon-sparing, direct-acting antiviral dual-combination regimens (together with the NS5A replication complex inhibitor daclatasvir) in patients chronically infected with HCV genotype 1b.	225 082 97
HCV	DB 010 72	atazanavi r	Atazanavir (ATV) is a widely used HIV protease inhibitor (PI) both in treatment-experienced and treatment-naive patients due to its potent antiviral activity and good safety profile.	254 095 06
HIV	DB 010 72	atazanavi r	Atazanavir (ATV) is a widely used HIV protease inhibitor (PI) both in treatment-experienced and treatment-naive patients due to its potent antiviral activity and good safety profile.	254 095 06
HIV	DB	atorvasta	To investigate the efficacy of other commonly prescribed statins, we compared the antiviral activities of lovastatin, fluvastatin,	297

	010 76	tin	simvastatin, atorvastatin, rosuvastatin, and pitavastatin in Huh7 cells.	170 11
HBV	DB 010 76	atorvasta tin	To investigate the efficacy of other commonly prescribed statins, we compared the antiviral activities of lovastatin, fluvastatin, simvastatin, atorvastatin, rosuvastatin, and pitavastatin in Huh7 cells.	297 170 11
SARS-CoV-2	DB 002 07	azithrom ycin	Azithromycin antiviral properties in vitro	323 024 11
HCV	DB 122 83	balapirav ir	Balapiravir was originally developed as an antiviral agent against hepatitis C virus (HCV) and was evaluated in a phase 1 clinical trial as a short-course antiviral against DENV.	346 983 03
Influenz a A	DB 139 97	baloxavir marboxil	Baloxavir marboxil (BXM), an antiviral drug for influenza virus, inhibits RNA replication by binding to RNA replication cap-dependent endonuclease (CEN) of influenza A and B viruses.	317 679 49
HCV	DB 122 25	beclabuv ir	Daclatasvir, asunaprevir (ASV), and beclabuvir (BCV) are direct-acting antivirals (DAAs) for patients with hepatitis C virus genotype 1 infection.	288 922 35
HIV	DB 041 15	berberine	Antiviral activity of berberine	325 943 22
HBV	DB 050 20	besifovir dipivoxil	BACKGROUND/AIMS: Besifovir dipivoxil maleate (BSV), an acyclic nucleotide phosphonate, shows potent antiviral activity against hepatitis B virus.	334 933 93
HIV	DB 065 81	bevirima t	The HIV-1 maturation inhibitor, 3-O-(3',3'-dimethylsuccinyl) betulinic acid (bevirimat, PA-457) is a promising drug candidate with 10 nM in vitro antiviral activity against multiple wild-type (WT) and drug-resistant HIV-1 isolates.	180 437 58
HIV	DB 117 99	bictegrav ir	Antiviral activity of bictegravir against HIV clinical isolates	276 452 38
HBV	DB 117 99	bictegrav ir	Antiviral activity of bictegravir against HIV clinical isolates	276 452 38
HCV	DB 117 99	bictegrav ir	Antiviral activity of bictegravir against HIV clinical isolates	276 452 38

HBV	DB 160 14	bicyclol	After seeing the anti-HCV activity of bicyclol in vitro and in hepatitis C patients, we used bicyclol as a probe in an attempt to explore the antiviral molecular mechanism of bicyclol.	313 845 37
HCV	DB 160 14	bicyclol	After seeing the anti-HCV activity of bicyclol in vitro and in hepatitis C patients, we used bicyclol as a probe in an attempt to explore the antiviral molecular mechanism of bicyclol.	313 845 37
HCV	na	bifendate	Anti-HBV efficacy of bifendate in treatment of chronic hepatitis B, a primary study	121 335 00
HBV	na	bifendate	Anti-HBV efficacy of bifendate in treatment of chronic hepatitis B, a primary study	121 335 00
HBV	DB 117 82	birinapan t	Birinapant is an apoptosis inhibitor, and it has approved efficiency in controlling viral hepatitis in combination with other antiviral drugs.	351 864 96
HCV	DB 088 73	boceprev ir	Treatment with NS3 protease inhibitors (telaprevir and boceprevir):the first direct-acting antivirals (DAAs) for HCV:were associated with less severe side-effects.	301 272 85
HIV	DB 048 87	brecanav ir	CONCLUSIONS: Brecanavir/ritonavir was well tolerated and showed potent antiviral activity in HIV-1-infected patients harbouring both PI-sensitive and PI-resistant virus, following 24 weeks of dosing.	174 910 01
HHV	DB 121 51	brincidof ovir	As of November 2016, two leading antiviral drugs were in development, namely ST-246 (Tecovirimat) and CMX001 (Brincidofovir, derived from the licensed antiviral drug cidofovir).	302 708 59
VARV	DB 121 51	brincidof ovir	As of November 2016, two leading antiviral drugs were in development, namely ST-246 (Tecovirimat) and CMX001 (Brincidofovir, derived from the licensed antiviral drug cidofovir).	302 708 59
SARS-CoV-2	DB 090 19	bromhex ine	Bromhexine is a potent inhibitor of transmembrane serine protease 2 and has an antiviral effect.	337 229 99
SARS-CoV-2	DB 121 60	bucillami ne	The drug potentially has capability on the antiviral and prophylactic side as studied at the University of California, San Francisco, where they showed that thiol drugs like bucillamine may help prevent the binding of the virus to the ACE2 receptors, but most of the focus has been on its anti-oxidant and anti-inflammatory capabilities.	350 442 38
HIV	DB 117	cabotegr avir	Cabotegravir (formerly GSK1265744) is an integrase strand transfer inhibitor (INSTI) with potent antiviral activity.	316 444

	51			81
HCV	DB 002 01	caffeine	Although caffeine was reported to play a small role in antiviral activity of coffee extract against HSV, the coffee in our studies had a much greater antiviral effect than the energy drink, although these two beverages had similar caffeine content.	353 272 25
SARS-CoV-2	DB 137 29	camostat	These compounds are antiviral drugs used for the treatment of diseases caused by MERS-CoV (all the five compounds), SARS-CoV (trametinib) and another human coronavirus HCoV-229E (camostat).	340 641 63
SARS-CoV-2	DB 139 19	candesart an	Candesartan cilexetil also exhibited antiviral effects against Dengue virus serotype-2 (DENV2), Kunjin virus (KUNV) and Chikungunya virus (CHIKV), indicating that its antiviral properties may not be restricted to ZIKV.	316 693 33
HIV	DB 090 61	cannabid iol	Importance: Owing to its anti-inflammatory properties and antiviral "in vitro" effect against severe acute respiratory syndrome coronavirus 2 (SARS-CoV-2), cannabidiol (CBD) has been proposed as a potential treatment for coronavirus disease 2019 (COVID-19).	346 190 44
HBV	DB 005 64	carbama zepine	Rapamycin, along with other autophagy-inducing treatments such as metformin, statins, and carbamazepine, has been demonstrated to have antiviral properties in laboratory animals.	359 422 34
HSV	DB 014 13	cefepime	Broad-spectrum antibiotic and antiviral coverage (cefepime, linezolid, ampicillin, and acyclovir) as well as hydroxychloroquine were initiated for empiric treatment of COVID-19 and meningitis.	324 253 32
SARS-CoV-2	DB 065 90	ceftaroli ne fosamil	The RNA binding site with Ceftaroline fosamil binding can prevent viral assembly and can act as an antiviral for coronavirus.	340 224 85
HIV	DB 012 12	ceftriaxo ne	At the ICU, a 4-day course with empirical antibiotics and antivirals (ceftriaxone + azithromycin + oseltamivir) was used.	352 848 11
HCV	DB 065 80	celgosivi r	For antiviral applications, Bu-CAST (Celgosivir) was evaluated in clinical trials against HIV (NCT00002150 and NCT00002151) and HCV (NCT00157534) infections.	238 164 30
HIV	DB 117 58	cenicrivi roc	Cenicriviroc (CVC), a novel, oral, once-daily, dual CCR5/C-C chemokine receptor type 2 (CCR2) antagonist, demonstrated potent antiviral activity and was generally well tolerated in HIV-1-infected, antiretroviral-experienced, CCR5 antagonist-naive study participants.	266 369 29
SARS-CoV-2	DB 110 73	cetylpyri dinium	Oral care products containing cetylpyridinium chloride (CPC) as a bactericidal ingredient are known to exhibit antiviral activity against SARS-CoV-2 in vitro.	354 410 76
HCV	DB	chlorcycl	Surprisingly, several antihistamines, such as chlorcyclizine, chlorpheniramine, and diphenhydramine, have demonstrated antiviral	327

	089<br>36	izine	action against hepatitis C virus (HCV), filoviruses (consisting of Ebola virus, Marburg virus, and Cuevavirus), and Influenza A virus infection.	516<br>15
SARS-<br>CoV-2	DB<br>006<br>08	chloroqu<br>ine	Some potential drugs have shown antiviral effects on SARS-CoV-2 infections, such as chloroquine, hydroxychloroquine, favipiravir, lopinavir/ritonavir, arbidol, interferon alpha, and remedsivir.	328<br>016<br>40
SARS-<br>CoV-2	DB<br>004<br>77	chlorpro<br>mazine	It was expected because ribavirin, chloroquine and chlorpromazine had different antiviral mechanisms.	257<br>969<br>72
HHV	DB<br>003<br>69	cidofovir	Cidofovir (HPMPC) is a broad-spectrum antiviral agent, currently used to treat AIDS-related human cytomegalovirus retinitis.	199<br>690<br>24
VARV	DB<br>003<br>69	cidofovir	Cidofovir (HPMPC) is a broad-spectrum antiviral agent, currently used to treat AIDS-related human cytomegalovirus retinitis.	199<br>690<br>24
HIV	DB<br>003<br>69	cidofovir	Cidofovir (HPMPC) is a broad-spectrum antiviral agent, currently used to treat AIDS-related human cytomegalovirus retinitis.	199<br>690<br>24
HSV	DB<br>003<br>69	cidofovir	Cidofovir (HPMPC) is a broad-spectrum antiviral agent, currently used to treat AIDS-related human cytomegalovirus retinitis.	199<br>690<br>24
HCV	DB<br>058<br>68	ciluprevi<br>r	The clinical proof-of-concept for NS3/4A protease inhibitors was achieved with ciluprevir (BILN 2061), a macrocyclic protease inhibitor that showed substantial antiviral activity in patients with HCV genotype 1.	218<br>133<br>71
HSV	DB<br>005<br>37	ciproflox<br>acin	Ciprofloxacin has antiviral activity against BKV by inhibiting DNA topoisomerase activity.	306<br>196<br>88
Influenz<br>a A	DB<br>012<br>11	clarithro<br>mycin	The antiviral effect of macrolides is then tested in cell culture, and results suggest that azithromycin, clarithromycin, and lexithromycin display antiviral activity against SARS-CoV-2 by impeding viral entry.	337<br>347<br>04
HIV	DB<br>012<br>11	clarithro<br>mycin	The antiviral effect of macrolides is then tested in cell culture, and results suggest that azithromycin, clarithromycin, and lexithromycin display antiviral activity against SARS-CoV-2 by impeding viral entry.	337<br>347<br>04
SARS-<br>CoV-2	DB<br>007<br>66	clavulani<br>c acid	Regarding the SARS-CoV-2 helicase, the small molecules acetylcysteine, homovanillic acid, and clavulanic acid were identified, in our studies, as potential lead compounds for further examination to investigate specificity and in antiviral assays.	354<br>621<br>85

HBV	DB 066 83	clevudin e	Among the oral antiviral agents, including lamivudine, adefovir, entecavir, telbivudine, tenofovir, and clevudine, clevudine had a protective effect on renal function compared with lamivudine.	267 355 42
HIV	DB 007 58	clopidog rel	To determine if the antiviral activity of Clopidogrel was influenza type-specific it was evaluated against B/Yamagata/16/1988 for antiviral efficacy.	347 148 52
HSV	DB 003 63	clozapin e	In 2003, Baum et al showed that both Chlorpromazine and Clozapine, two antipsychotic medications which act as antagonists against dopamine and serotonin receptors, both possess antiviral activity against JCV, which led to the hypothesis that JCV may interact with these neurotransmitter receptors to enter permissive cells, perhaps explaining the high glial-tropism.	314 520 13
HIV	DB 090 65	cobicista t	the dual action of cobicistat as a direct antiviral and a drug booster can provide a new approach to design combination therapies and rescue the activity of compounds that are only partially effective in monotherapy.	352 296 34
HCV	DB 090 65	cobicista t	the dual action of cobicistat as a direct antiviral and a drug booster can provide a new approach to design combination therapies and rescue the activity of compounds that are only partially effective in monotherapy.	352 296 34
HCV	DB 153 15	coblopas vir	Coblopasvir (formerly coded as KW-136) is a pangenotypic inhibitor against HCV non-structural protein (NS) 5A with picomolar antiviral activities against HCV replicons or cell culture systems of genotypes 1a, 1b, 2a, 3a, 4a, 5a and 6a in vitro (data on file).	330 478 68
HCV	DB 013 94	colchicin e	CONCLUSION: These preliminary results suggest that colchicine might have an antiviral activity in HBV-DNA+ve chronic liver disease, and it could be regarded as an alternative therapy to interferon.	970 152 9
Influenz a A	DB 136 74	cridanim od	We found that the antiviral effect of Tilorone and Cridanimod in rats is not linked to the induction of circulating IFN-alpha or IFN-beta.	356 314 43
SARS-CoV-2	DB 004 34	cyprohep tadine	Antiviral activity of hit compounds in cells was confirmed for clomiphene (4, Table 2) and cyproheptadine (5) which inhibited HIV transcription and affected levels of spliced versus unspliced viral transcripts to an extent that was consistent with their ability to disrupt Rev-RRE complex function in vitro.	273 072 13
HIV	DB 091 02	daclatasv ir	Simeprevir (SMV), asunaprevir (ASV), daclatasvir (DCV), and sofosbuvir (SFV), which are newly developed direct-acting antiviral agents (DAAs) against hepatitis C virus (HCV) infection, are among the key components of anti-HCV regimens.	248 679 84
HCV	DB 091 02	daclatasv ir	Simeprevir (SMV), asunaprevir (ASV), daclatasvir (DCV), and sofosbuvir (SFV), which are newly developed direct-acting antiviral agents (DAAs) against hepatitis C virus (HCV) infection, are among the key components of anti-HCV regimens.	248 679 84
HCV	DB 117	danoprev ir	The results of one study suggested that danoprevir's antiviral effects may restore insulin sensitivity in patients with genotype 1 HCV.	272 939

	79			23
SARS-CoV-2	DB 062 92	dapaglifl ozin	Dapagliflozin can prevent the severe course of COVID-19 infection by preventing the lowering of cytosolic pH and reducing the viral load.	323 353 66
HIV	DB 086 39	dapivirin e	Antiviral mechanism of dapivirine	287 788 30
HHV	DB 012 64	darunavi r	In the present study, we compared the antiviral activity of PL-100 against HIV-1 subtype B with that of darunavir.	229 459 18
HIV	DB 012 64	darunavi r	In the present study, we compared the antiviral activity of PL-100 against HIV-1 subtype B with that of darunavir.	229 459 18
HCV	DB 091 83	dasabuvi r	The direct-acting antivirals including sofosbuvir, simeprevir, ledipasvir, ombitasvir, dasabuvir, and other drugs such as pegylated interferon-alpha, ribavirin are used for the treatment of HCV infection.	303 220 29
SARS-CoV-2	DB 007 46	deferoxa mine	Iron chelators, including deferoxamine, deferiprone and deferasirox possess antiviral actions against several viruses, including HIV, West Nile and HCV.	333 736 81
HIV	DB 007 05	delavirdi ne	The 12 FDA-approved antiviral drugs are paritaprevir, dolutegravir, raltegravir, efavirenz, elvitegravir, tipranavir, saquinavir, dasabuvir, delavirdine, maraviroc, trifluridine, and sodium tauroursodeoxycholate.	323 261 19
HCV	DB 148 50	deleobuv ir	Deleobuvir monotherapy was generally well tolerated and demonstrated dose-dependent antiviral activity against HCV genotype 1 over 5 days.	238 567 79
HSV	na	denotivir	Because the amide series of 5-benzoylamino-3-methyl-4-isothiazolecarboxylic acid 2 has been studied extensively and from this series denotivir (vratizolin) 4 became the antiviral drug.	168 382 83
HIV	DB 012 34	dexamet hasone	Recently, dexamethasone is also been widely accepted as an antiviral drug for the treatment of COVID-19 patients.	340 141 50
HBV	DB 012 34	dexamet hasone	Recently, dexamethasone is also been widely accepted as an antiviral drug for the treatment of COVID-19 patients.	340 141 50
HHV	DB	didanosi	Didanosine is an effective antiviral drug in untreated and antiretroviral therapy-experienced patients with Human Immunodeficiency	294

	009 00	ne	Virus (HIV).	037 17
HBV	DB 009 00	didanosi ne	Didanosine is an effective antiviral drug in untreated and antiretroviral therapy-experienced patients with Human Immunodeficiency Virus (HIV).	294 037 17
HIV	DB 009 00	didanosi ne	Didanosine is an effective antiviral drug in untreated and antiretroviral therapy-experienced patients with Human Immunodeficiency Virus (HIV).	294 037 17
SARS-CoV-2	DB 003 43	diltiazem	L-type calcium channels, which suggests that diltiazem could be a safe antiviral drug candidate for SARS-CoV-2 infection.	351 761 24
SARS-CoV-2	DB 009 75	dipyrida mole	THE ANTIVIRAL ACTIVITY OF DIPYRIDAMOLE	330 010 04
HIV	DB 008 22	disulfira m	Disulfiram (DS), known as an anti-alcoholism drug, has shown a potent antiviral activity.	360 803 68
HIV	DB 089 30	dolutegra vir	Phase III trials have assessed the antiviral activity of dolutegravir compared with efavirenz and raltegravir in antiretroviral (ARV)-naive patients and found dolutegravir to achieve more rapid and sustained virologic suppression in both instances.	238 246 75
HHV	DB 089 30	dolutegra vir	Phase III trials have assessed the antiviral activity of dolutegravir compared with efavirenz and raltegravir in antiretroviral (ARV)-naive patients and found dolutegravir to achieve more rapid and sustained virologic suppression in both instances.	238 246 75
HIV	DB 123 01	doravirin e	As part of a combined antiretroviral regimen, doravirine is safe and effective at suppressing viral replication in both treatment-naive and treatment-experienced adults living with human immunodeficiency virus (HIV)-1 who have no history of drug resistance against doravirine.	321 808 23
HSV	DB 134 21	edoxudin e	Since the first FDA-approved nucleoside analog "edoxudine" in 1969, there are currently 25 FDA-approved nucleoside/tide analogs and another 15 nucleoside analogs used as antiviral and anticancer agents, respectively.	358 894 02
HCV	DB 006 25	efavirenz	Efavirenz (EFV) is a non-nucleoside reverse transcriptase inhibitor which is recommended as an oral administration with other antiviral agents against HIV.	332 974 44
HIV	DB 006 25	efavirenz	Efavirenz (EFV) is a non-nucleoside reverse transcriptase inhibitor which is recommended as an oral administration with other antiviral agents against HIV.	332 974 44

HBV	DB 00625	efavirenz	Efavirenz (EFV) is a non-nucleoside reverse transcriptase inhibitor which is recommended as an oral administration with other antiviral agents against HIV.	33297444
HCV	DB 11574	elbasvir	Grazoprevir and elbasvir are second-generation inhibitors for NS3/4A protease and NS5A, respectively, with potent broad antiviral activity against HCV.	27840363
HIV	DB 14929	elsulfavirine	Elsulfavirine demonstrated excellent antiviral efficacy in treatment-naive patients during the clinical trials.	34000969
HCV	DB 09101	elvitegravir	Elvitegravir is a potent, boosted, once-daily, HIV integrase inhibitor with antiviral activity against wild-type and drug-resistant strains of HIV.	21348537
HIV	DB 09101	elvitegravir	Elvitegravir is a potent, boosted, once-daily, HIV integrase inhibitor with antiviral activity against wild-type and drug-resistant strains of HIV.	21348537
HIV	DB 06236	elvucitabine	Elvucitabine has also demonstrated potent antiviral activity in HIV-infected patients with resistance to 3TC and other NRTIs.	24278679
HCV	DB 05408	emricasan	The potential antiviral activity of emricasan was identified in a drug repurposing screen following the Zika virus outbreak in 2016.	32586380
HIV	DB 00879	emtricitabine	Emtricitabine, a new antiretroviral agent with activity against HIV and hepatitis B virus.	16323102
HBV	DB 00879	emtricitabine	Emtricitabine, a new antiretroviral agent with activity against HIV and hepatitis B virus.	16323102
HCV	DB 00879	emtricitabine	Emtricitabine, a new antiretroviral agent with activity against HIV and hepatitis B virus.	16323102
HCV	DB 00442	entecavir	Antiviral therapy included lamivudine, telbivudine, adefovir, tenofovir, or entecavir.	26595058
HBV	DB 004	entecavir	Antiviral therapy included lamivudine, telbivudine, adefovir, tenofovir, or entecavir.	265950

	42			58
SARS-CoV-2	DB04933	eritoran	We also briefly review the proposed use of TLR4 antagonists as antiviral treatments, including Eritoran, Resatorvid (CLI-095/TAK242), and glycyrrhizin, as well as another compound, nifuroxazide, that interrupts TLR4 signalling.	33505220
HCV	DB00530	erlotinib	Erlotinib, gefitinib, and lapatinib (anticancer drugs targeting EGFR and ERBb kinase family ERBB2) demonstrate in vitro and in vivo antiviral activities against hepatitis C virus (HCV) and human cytomegalovirus.	34951532
HCV	DB01175	escitalopram	Encouraged by the above pieces of evidence and in line with the global impetus of finding a new effective antiviral agent to treat the pandemic SARS-CoV-2, we assessed the anti-SARS-CoV-2 activity of eight commonly prescribed antidepressants (1-8) including Amitriptyline and Imipramine (TCAs class), Citalopram, Escitalopram, Paroxetine, Sertraline, Mirtazapine (SSRIs class), and Eszopiclone (MAOIs class) (Fig. 1).	35902647
HIV	DB00773	etoposide	The Etoposide-induced antiviral effect was associated with activation of p53, p21, increased expression of CDK1 and de-phosphorylation of SAMHD1.	35330617
HIV	DB06414	etravirine	However etravirine has been shown to have antiviral activity against HIV with resistance to efavirenz or nevirapine.	24715982
HCV	DB06414	etravirine	However etravirine has been shown to have antiviral activity against HIV with resistance to efavirenz or nevirapine.	24715982
HBV	DB06414	etravirine	However etravirine has been shown to have antiviral activity against HIV with resistance to efavirenz or nevirapine.	24715982
HCV	DB00973	ezetimibe	We identified novel antiviral activities for dalbavancin (against EV1), ezetimibe (against HIV-1 and ZIKV), azacitidine, cyclosporine, minocycline, oritavancin and ritonavir (against RVFV) (Fig. 4).	29698664
HCV	DB11808	faldaprevir	Faldaprevir (BI 201335) is a HCV NS3/4A protease inhibitor with pharmacokinetic properties supportive of QD dosing, with potent antiviral activity in HCV genotype 1-infected patients as well as HCV genotypes 4, 5, and 6.	26356295
HSV	DB00426	famciclovir	Famciclovir is an antiviral agent employed for the treatment of Herpes zoster infections.	35733311
HHV	DB00426	famciclovir	Famciclovir is an antiviral agent employed for the treatment of Herpes zoster infections.	35733311

HIV	DB00927	famotidine	Cimetidine and famotidine showed broad spectrum antiviral activities and have been proven to be effective in rotavirus gastroenteritis.	27679574
SARS-CoV-2	DB12466	favipiravir	Favipiravir antiviral efficacy against SARS-CoV-2 in a hamster model	33741945
SARS-CoV-2	DB01023	felodipine	Moreover, an array of calcium channel inhibitors, such as cilnidipine, felodipine, amlodipine, manidipine, nicardipine, and nisoldipine, exhibit similar antiviral properties.	36105208
HBV	DB15427	fialuridine	Fialuridine is an antiviral agent with potent activity against hepatitis B virus replication in vitro and in vivo.	9258627
HCV	DB11878	filibuvir	Moreover, NS5B substitutions M423T and I482L could affect the antiviral activity of filibuvir and VX-222.	28238877
SARS-CoV-2	DB00472	fluoxetine	An interesting study showed that the antidepressant drug fluoxetine exerts antiviral effects in an HCV model.	35053112
HCV	DB01095	fluvastatin	Fluvastatin or simvastatin has demonstrable antiviral activity against hepatitis C virus (HCV) as monotherapy.	23910646
SARS-CoV-2	DB00176	fluvoxamine	Furthermore, psychotropic drugs, such as mood stabilizers (i.e., lithium and valproate), antipsychotics (i.e., clozapine and chlorpromazine), and antidepressants (i.e., fluvoxamine), showed potential antiviral actions, inhibiting viruses infection and/or reactivation.	36101642
SARS-CoV-2	DB00983	formoterol	As discussed later, ciclesonide and formoterol are commonly used inhalers in asthma, and their antiviral properties could protect patients with asthma from COVID-19.	32624257
HIV	DB01319	fosamprenavir	As a result, anti-HIV or antiviral drugs (lopinavir, tenofovir disoproxil, fosamprenavir and ganciclovir), antiflu drugs (peramivir and zanamivir) and an anti-HCV drug (sofosbuvir) are predicted to bind to 3CLPro in SARS-CoV-2 with therapeutic potential for COVID-19 infection by our new protocol.	33078827
SARS-CoV-2	DB12010	fostamatinib	As a result, we identified cangrelor and fostamatinib as potential antiviral drugs against ASFVs.	34948216
HIV	DB117	fostemsavir	Notably, enfuvirtide, maraviroc, and fostemsavir, which are all PPI inhibitors, are approved for clinical treatment against HIV-1, showing that such strategies can be successful for antiviral drug discovery.	361453

	96			05
HHV	DB 010 04	ganciclo vir	Antiviral drugs such as foscarnet, valganciclovir, ganciclovir, letermovir, or brincidofovir are administered to prevent and control viral infection and reactivation.	308 978 43
HCV	DB 138 79	glecapre vir	Glecaprevir (GLE) is a potent NS3/4A protease inhibitor with nanomolar antiviral activity against HCV GT 1-6 and most known NS3 RASs.	294 274 84
HCV	DB 115 75	grazopre vir	Grazoprevir and elbasvir are second-generation inhibitors for NS3/4A protease and NS5A, respectively, with potent broad antiviral activity against HCV.	278 403 63
HCV	DB 152 22	gs-6620	Discovery of the first C-nucleoside HCV polymerase inhibitor (GS-6620) with demonstrated antiviral response in HCV infected patients.	235 477 94
HCV	DB 128 76	gs-9256	The NS3 protease inhibitor (PI) GS-9256 has demonstrated antiviral activity in a monotherapy study and in combination with other DAAs for treatment of chronic hepatitis C virus (HCV) infection.	281 328 67
HIV	DB 131 19	gsk-364735	GSK-364735 inhibited IN in an in vitro strand transfer assay with an IC50 of 8 nM, and it showed an antiviral EC90 value of 40 nM in MT-4 cells in the presence of 20% NHS.	192 655 12
SARS-CoV-2	DB 010 18	guanfaci ne	COVID-19 therapies included hydroxychloroquine (HCQ), antibiotics (azithromycin, imipenem, cotrimoxazole, vancomycin), antivirals (lopinavir/ritonavir (Lpv/r), remdesivir, oseltamivir, sofosbuvir, darunavir/cobicistat), immunomodulators (tocilizumab, rituximab, ruxolitinib, interferon, anakinra), steroids (irrespective of the type and mode of administration), anticoagulants (low molecular weight or unfractionated heparin), and other medications (fluconazole, guanfacine).	342 782 87
HSV	DB 005 02	haloperid ol	Antiviral effect of haloperidol on Rauscher murine leukemia virus	722 481 9
SARS-CoV-2	DB 007 41	hydrocor tisone	Other drugs in current use for the treatment of COVID-19, such as dexamethasone and hydrocortisone, are symptomatic immune modifiers that modulate the damaging effects of the virus-induced "cytokine storm" and are not antivirals.	335 461 85
SARS-CoV-2	DB 010 50	ibuprofe n	It is noteworthy that several NSAIDs have antiviral activity, namely, aspirin, ibuprofen, naproxen, acetaminophen and lornoxicam, being able to potently inhibit the entry of Zika virus into the cells.	352 699 97
HSV	DB 002 49	idoxuridi ne	Idoxuridine is a nucleoside analog and have been used as an antiviral drug for herpes.	359 909 01

SARS-CoV-2	DB 006 19	imatinib	Imatinib, a Bcr-Abl tyrosine kinase inhibitor, showed maximum antiviral activity in Vero cells.	352 209 26
HPV	DB 007 24	imiquim od	Imiquimod (IMQ), a synthetic TLR7 ligand, has both antiviral and antitumor activity against various skin malignancies in clinical treatment.	360 757 80
HBV	DB 007 24	imiquim od	Imiquimod (IMQ), a synthetic TLR7 ligand, has both antiviral and antitumor activity against various skin malignancies in clinical treatment.	360 757 80
HIV	DB 002 24	indinavir	Delavirdine, Dolutegravir and Indinavir are FDA approved antiviral drugs used for the treatment of Human Immunodeficiency virus (HIV).	331 795 68
SARS-CoV-2	DB 003 28	indometh acin	Another NSAID, indomethacin, reported to have direct antiviral effect on SARS-CoV by interfering with viral RNA synthesis, independent of cyclooxygenase inhibition in an in vitro study.	322 992 02
SARS-CoV-2	DB 119 44	ingavirin	[Experimental investigation of Ingavirin antiviral activity against human parainfluenza virus].	211 405 58
HCV	DB 010 29	irbesarta n	Additional small molecules, such as cyclosporin A, irbesartan, and vanitaracin A have demonstrated antiviral against HBV and HDV in NTCP-overexpressing cells.	344 523 97
SARS-CoV-2	DB 116 33	isavucon azole	Isavuconazole also displays antiviral activity to five New World (NW) mammarenaviruses that cause hemorrhagic fever.	318 773 48
HIV	DB 156 53	islatravir	Islatravir uniquely inhibits reverse transcriptase to suppress HIV-1 replication by multiple mechanisms of action, including inhibition of translocation, with potent antiviral activity against wild-type HIV-1 and common nucleotide reverse transcriptase inhibitor (NRTI)-resistant variants.	341 514 13
HIV	DB 009 51	isoniazid	The antiviral adenosine analog, 2',3'-dideoxyinosine (ddI), and the antitubercular nicotinic acid analogue, isoniazid, have recently received widespread clinical application in the treatment of acquired immunodeficiency syndrome (AIDS).	888 802 0
HIV	DB 009 82	isotretino in	Lopinavir and isotretinoin are examples of drugs repurposed for potential antiviral therapy, although mechanism of the drug's action is unclear.	341 112 71
HCV	DB 011	itraconaz ole	Another host factor-targeting compound, the antifungal agent Itraconazole (ITZ), was shown to have antiviral activity against PeV-A3 but not against PeV-A1.	317 396

	67			13
HBV	DB 011 67	itraconaz ole	Another host factor-targeting compound, the antifungal agent Itraconazole (ITZ), was shown to have antiviral activity against PeV-A3 but not against PeV-A1.	317 396 13
SARS-CoV-2	DB 006 02	ivermecti n	Ivermectin as an Antiviral	329 426 71
SARS-CoV-2	DB 009 20	ketotifen	In Vitro Assessment of the Antiviral Activity of Ketotifen, Indomethacin and Naproxen, Alone and in Combination, against SARS-CoV-2	338 103 56
HIV	DB 007 09	lamivudi ne	Lamivudine is an oral nucleoside analogue with strong antiviral activity against hepatitis B virus.	169 646 75
HBV	DB 007 09	lamivudi ne	Lamivudine is an oral nucleoside analogue with strong antiviral activity against hepatitis B virus.	169 646 75
HCV	DB 090 27	ledipasvi r	Ledipasvir is a new HCV-NS5A inhibitor with antiviral activity against HCV genotype 1.	267 709 24
HIV	DB 090 27	ledipasvi r	Ledipasvir is a new HCV-NS5A inhibitor with antiviral activity against HCV genotype 1.	267 709 24
HBV	DB 090 27	ledipasvi r	Ledipasvir is a new HCV-NS5A inhibitor with antiviral activity against HCV genotype 1.	267 709 24
HIV	DB 156 73	lenacapa vir	Lenacapavir (LEN, formerly GS-6207) is the first clinically validated HIV capsid inhibitor and displays picomolar antiviral activity against both wild-type virus and variants resistant to current ARVs.	348 756 75
HHV	DB 120 70	letermov ir	Letermovir's antiviral activity is highly specific to CMV and currently is the most active molecule against CMV, with a very low median effective concentration and preserved activity against CMV isolates that are resistant to other antiviral agents.	301 040 16
SARS-CoV-2	DB 008 48	levamiso le	Levamisole and inosiplex: antiviral agents with immunopotentiating action.	816 35
SARS-	DB	levofloxa	Therefore, empirical antiviral drugs (lopinavir/ritonavir, favipiravir, oseltamivir, zanamivir and peramivir, arbidol), antibiotic drugs	325

CoV-2	01137	cin	(moxifloxacin, levofloxacin, linezolid), chloroquine/hydroxychloroquine, glucocorticoids, monoclonal anti-inflammatory antibody (tocilizumab), have been used to treat SARS-CoV-2 infection.	24843
SARS-CoV-2	DB08882	linagliptin	Furthermore, DPP4i, such as gemigliptin, linagliptin, and evogliptin, reportedly exert antiviral properties, suggesting their potential as broad-spectrum antiviral agents.	35413782
HIV	DB00601	linezolid	Therefore, empirical antiviral drugs (lopinavir/ritonavir, favipiravir, oseltamivir, zanamivir and peramivir, arbidol), antibiotic drugs (moxifloxacin, levofloxacin, linezolid), chloroquine/hydroxychloroquine, glucocorticoids, monoclonal anti-inflammatory antibody (tocilizumab), have been used to treat SARS-CoV-2 infection.	32524843
SARS-CoV-2	DB00722	lisinopril	Very recently, captopril and lisinopril were evaluated as antiviral drugs in the worldwide fight against the COVID-19 pandemic caused by the SARS-CoV-2 virus.	34292730
HHV	DB12531	lobucavir	Successful cyclobutyl nucleosides have been developed as antiviral agents, such as Lobucavir against HBV.	22595174
HIV	DB01601	lopinavir	Lopinavir antiviral is a protease inhibitor of human immunodeficiency virus 1 (HIV1) and generally used as a booster with ritonavir.	35453248
HBV	DB01601	lopinavir	Lopinavir antiviral is a protease inhibitor of human immunodeficiency virus 1 (HIV1) and generally used as a booster with ritonavir.	35453248
HIV	DB00678	losartan	Therefore, the researchers tried to use losartan as an anti-SARS-CoV-2 candidate by combining antidiabetic, anti-inflammatory, and antiviral effects to help patients recover.	35889013
HCV	DB00678	losartan	Therefore, the researchers tried to use losartan as an anti-SARS-CoV-2 candidate by combining antidiabetic, anti-inflammatory, and antiviral effects to help patients recover.	35889013
SARS-CoV-2	DB12270	losmapimod	Interestingly, the p38 inhibitor losmapimod has been shown to impair LASV entry into the host cell, but the antiviral activity of this compound is not achieved through inhibition of the p38 pathway.	35133480
SARS-CoV-2	DB06708	lumefantrine	The antiviral effect of the aryl-aminoalcohol compounds quinine sulfate, mefloquine, halofantrine, and lumefantrine on both classical and emerging viruses has been studied.	31936284
HIV	DB04835	maraviroc	Maraviroc, the only CCR5 antagonist HIV inhibitor currently approved, has potent antiviral activity in treatment-experienced individuals infected with CCR5-using HIV-1 (R5 HIV-1).	19501260

HBV	DB 048 35	maraviro c	Maraviroc, the only CCR5 antagonist HIV inhibitor currently approved, has potent antiviral activity in treatment-experienced individuals infected with CCR5-using HIV-1 (R5 HIV-1).	195 012 60
HCV	DB 048 35	maraviro c	Maraviroc, the only CCR5 antagonist HIV inhibitor currently approved, has potent antiviral activity in treatment-experienced individuals infected with CCR5-using HIV-1 (R5 HIV-1).	195 012 60
HHV	DB 062 34	maribavi r	Maribavir is orally administered and has specific antiviral activity against CMV, including ganciclovir-resistant and cidofovir-resistant CMV strains.	301 040 16
HCV	DB 120 45	mericitab ine	Mericitabine (RG7128) is an oral cytidine nucleoside analog prodrug that exhibited strong antiviral effectiveness against the HCV polymerase across all HCV genotypes, with no evidence of resistance reported in patients treated with mericitabine monotherapy for 14 days.	220 953 98
HCV	DB 003 31	metformi n	Hence it is rational that we observed an antiviral action of metformin against JFH1, and indeed a trial in Spain showed that the addition of metformin to standard anti-HCV treatment improved SVR, which suggests a possible productive application of metformin and SNARK inhibitors to the anti-HCV armamentarium.	238 311 17
HIV	DB 003 31	metformi n	Hence it is rational that we observed an antiviral action of metformin against JFH1, and indeed a trial in Spain showed that the addition of metformin to standard anti-HCV treatment improved SVR, which suggests a possible productive application of metformin and SNARK inhibitors to the anti-HCV armamentarium.	238 311 17
HIV	DB 003 33	methado ne	These effects of methadone on HIV and the antiviral factors were antagonized by pretreatment of cells with naltrexone.	327 196 74
HCV	DB 003 33	methado ne	These effects of methadone on HIV and the antiviral factors were antagonized by pretreatment of cells with naltrexone.	327 196 74
Influenz a A	DB 002 11	midodrin e	The other molecules had comparable effects on the two H1N1 virus strains, with brinzolamide, midodrine and ribavirin being the most effective antivirals.	209 571 81
SARS-CoV-2	DB 008 34	mifeprist one	Antiviral effects of mifepristone on human immunodeficiency virus type-1 (HIV-1): targeting Vpr and its cellular partner, the glucocorticoid receptor (GR).	168 898 38
HCV	DB 050 63	mitoquin one	Herein, we show that the mitochondrial antioxidant mitoquinone/mitoquinol mesylate (Mito-MES), a dietary supplement, has potent antiviral activity against SARS-CoV-2 and its variants of concern in vitro and in vivo.	352 335 69
HIV	DB 129	mk-6186	Antiviral activity and in vitro mutation development pathways of MK-6186, a novel nonnucleoside reverse transcriptase inhibitor.	223 915

	99			31
SARS-CoV-2	DB15661	molnupiravir	Molnupiravir's antiviral efficacy and associated mechanism of action have been verified in vitro against both non-COVID and multiple coronaviruses.	35287311
Influenza A	na	moroxidine hydrochloride	Moroxydine hydrochloride (Mor) is known to have multi-antiviral activities against DNA and RNA viruses but very little information exists on its pharmacology	27188236
HHV	DB00688	mycophenolate mofetil	In fact, there are other immunosuppressants known with antiviral activities, including cyclosporine A, hydroxyurea, minocycline, mycophenolic acid, mycophenolate mofetil, leflunomide, tofacitinib, and thalidomide.	32952617
HIV	DB00688	mycophenolate mofetil	In fact, there are other immunosuppressants known with antiviral activities, including cyclosporine A, hydroxyurea, minocycline, mycophenolic acid, mycophenolate mofetil, leflunomide, tofacitinib, and thalidomide.	32952617
HHV	DB01024	mycophenolic acid	Mycophenolic acid, an immunomodulator, has potent and broad-spectrum in vitro antiviral activity against pandemic, seasonal and avian influenza viruses affecting humans	27259985
HIV	DB00704	naltrexone	Our data provide a proof-of-concept for the potential feasibility of repurposing of FDA approved non-peptide opioid antagonist; naltrexone as host-targeted broad-spectrum antiviral therapies to combat COVID-19 infections.	32930058
SARS-CoV-2	DB00788	naproxen	Antiviral Properties of the NSAID Drug Naproxen Targeting the Nucleoprotein of SARS-CoV-2 Coronavirus	33946802
HCV	DB14760	narlaprevir	Narlaprevir, a hepatitis C virus (HCV) NS3/4A serine protease inhibitor, has demonstrated robust antiviral activity in a placebo-controlled phase 1 study.	24168257
SARS-CoV-2	DB04861	nebivolol	Nebivolol, a cardioselective beta-blocker has also shown vasodilating properties, alongside antioxidative, anti-atherosclerotic activities, and antiviral properties.	35808843
HIV	DB00220	nelfinavir	HIV-1 protease inhibitors such as lopinavir/ritonavir and nelfinavir showed antiviral activity against SARS-CoV.	32585913
HIV	DB00238	nevirapine	The present study has demonstrated that an antiretroviral regimen of stavudine, lamivudine and nevirapine showed acceptable long-term antiviral effectiveness in advanced HIV-1 infected patients.	18851761

SARS-CoV-2	DB 068 03	niclosam ide	Antiviral activity of Niclosamide	346 641 62
SARS-CoV-2	DB 027 01	nicotina mide	Antiviral effect of nicotinamide on enterovirus-infected human islets in vitro: effect on virus replication and chemokine secretion.	193 822 75
HCV	DB 005 07	nitazoxa nide	The FDA-approved antiparasitic drug, nitazoxanide (NTZ), has been found to have antiviral activity against different viral infections such as coronaviruses, influenza, hepatitis C virus (HCV), hepatitis B virus (HBV), and other viruses signifying its potential as a broad spectrum antiviral drug.	327 252 86
HCV	DB 130 41	odalasvir	The doses chosen for evaluation in this study were either the highest approved dose (simeprevir) or doses found to have substantial antiviral effects in prior studies (AL-335 and odalasvir).	297 362 43
HCV	DB 092 96	ombitasv ir	The three-direct-acting antiviral (3D) regimen containing ombitasvir, paritaprevir, ritonavir, and dasabuvir with or without ribavirin (RBV) is approved for treatment of hepatitis C virus (HCV) genotype 1 (GT1)/human immunodeficiency virus type 1 (HIV-1) coinfection.	279 198 99
Influenz a B	DB 001 98	oseltami vir	Currently, four antivirals have proven efficacy in the treatment and prophylaxis of influenza A infections: two M2 inhibitors (amantadine and rimantadine) and two neuraminidase inhibitors (zanamivir and oseltamivir).	180 451 72
Influenz a A	DB 001 98	oseltami vir	Currently, four antivirals have proven efficacy in the treatment and prophylaxis of influenza A infections: two M2 inhibitors (amantadine and rimantadine) and two neuraminidase inhibitors (zanamivir and oseltamivir).	180 451 72
Influenz a A	DB 026 00	oseltami vir carboxyl ate	Oseltamivir has to be converted into oseltamivir carboxylate to have antiviral effects.	309 016 32
HIV	DB 012 29	paclitaxe l	Treatment with paclitaxel, a microtubule-stabilizing drug, has a strong antiviral effect, suggesting that microtubule flexibility is required for efficient ZIKV infection.	294 300 05
HCV	DB 007 15	paroxetin e	Also, Imipramine, Sertraline, and Paroxetine showed antiviral activity against MERS-CoV.	359 026 47
SARS-CoV-2	DB 008 06	pentoxif ylline	Antiviral activity of (A) pentoxifylline and (B) the standard antiviral agent ribavirin against Japanese encephalitis virus (JEV) evaluated using the plaque reduction assay.	188 043 47
Influenz	DB	peramivi	Oseltamivir, peramivir and zanamivir are three NA inhibitor antiviral interventions.	302

a A	066 14	r		108 00
SARS-CoV-2	DB 117 59	pevonedistat	Accordingly, we showed that the NAE inhibitor MLN4924, also known as pevonedistat, elicits broad and potent antiviral activity against mouse and human cytomegalovirus.	344 524 75
SARS-CoV-2	DB 165 14	pf-07304814	A series of antivirals being tested against SARS-CoV-2 such as PF-07304814, remdesivir, GC376, apilimod, nelfinavir and quinacrine, displayed high antiviral activity in vitro.	348 154 98
SARS-CoV-2	DB 166 91	pf-07321332	To make their antiviral candidate Paxlovid, the team combined PF-07321332 with the HIV antiviral ritonavir.	355 058 71
HCV	DB 138 78	pibrentasvir	Glecaprevir (formerly ABT-493; Fig. 1), a novel HCV NS3/4A PI with potent pangenotypic antiviral activity, is being developed for use in combination with the HCV NS5A inhibitor pibrentasvir (formerly ABT-530) for the treatment of HCV genotype 1 to 6 infection.	290 847 47
Influenza A	DB 149 74	pimodivir	Baloxavir is active against influenza A and B viruses, and pimodivir is only active against influenza A due to structural differences, whereas favipiravir has broad-spectrum antiviral activity against RNA viruses including filoviruses, arenaviruses, coronaviruses, and bunyaviruses.	355 513 01
HCV	DB 011 32	pioglitazone	Pioglitazone, a PPARgamma agonist, also exhibited antiviral activities against SARS-CoV-2, and both raloxifene and pioglitazone presented a synergistic antiviral effect.	338 224 89
HCV	DB 049 51	pirfenidone	Concomitantly, a strategy for repurposing existing approved small molecular drugs has been developed, with clinical trials being conducted on numerous existing antiviral drugs, such as remdesivir, hydroxychloroquine, favipiravir, pirfenidone, baricitinib, camostat, lopinavir/ritonavir and paritaprevir in search of an effective treatment for COVID-19.	345 755 89
HIV	DB 088 60	pitavastatin	To investigate the efficacy of other commonly prescribed statins, we compared the antiviral activities of lovastatin, fluvastatin, simvastatin, atorvastatin, rosuvastatin, and pitavastatin in Huh7 cells.	297 170 11
HPV	DB 011 79	podofilox	Podofilox, also termed podophyllotoxin, exhibits an antiviral function against herpes viruses, warts and influenza, as well as antitumor effects.	345 845 48
SARS-CoV-2	DB 062 09	prasugrel	The results suggest the effectiveness of vorapaxar, ticagrelor, cilostazol, cangrelor, and prasugrel in binding the main protease (Mpro) of SARS-CoV-2.	345 103 37
HIV	DB 001 75	pravastatin	Pravastatin, unlike the other tested statins, has no antiviral activity; hence, it inhibits HCV replication not by a direct action on HMG-CoA reductase, but by a specific antiviral mechanism.	228 798 25

HIV	DB 010 58	praziqua ntel	Remarkably, untreated Sm infection was associated with antiviral gene down-regulation, which was partially reversed by praziquantel therapy.	311 270 86
SARS-CoV-2	DB 008 60	prednisol one	The Food and Drug Administration (FDA) has approved antiviral drug (remdesivir), corticosteroid (dexamethasone, prednisolone, and methylprednisolone), and monoclonal antibodies, particularly IL-6 antibodies (tocilizumab, siltuximab, and anakinra), for the treatment of COVID-19.	347 215 73
HHV	DB 006 35	predniso ne	The patient was refractory to CHOP chemotherapy and immunosuppressive treatment in combination with antiviral drugs (prednisone, bortezomib, gancyclovir).	359 906 91
HCV	DB 006 35	predniso ne	The patient was refractory to CHOP chemotherapy and immunosuppressive treatment in combination with antiviral drugs (prednisone, bortezomib, gancyclovir).	359 906 91
HBV	DB 150 34	pri-724	We show the robust antiviral properties of PRI-724, iCRT14, and KYA1797k in multiple cell lines and against various laboratory and clinical strains of HSV-1.	347 495 29
HHV	DB 010 32	probenec id	Probenecid as an Antiviral	353 370 18
HIV	DB 003 96	progester one	Examples of this influence are the known antiviral effects of progesterone against HIV and the influenza virus, the immunomodulatory function of high doses of progesterone during pregnancy and the decrease of progesterone circulating levels in SARS patients.	347 613 29
SARS-CoV-2	DB 008 18	propofol	Putative antiviral effects of propofol in COVID-19.	336 856 35
SARS-CoV-2	DB 005 71	proprano lol	Propranolol also blocks the Warburg effect, disrupting the energy supply of both malignant and virus-infected cells, emphasizing the anticancer and antiviral properties of this drug.	347 768 71
HCV	DB 128 96	psi-352938	Since CYP3A4 is highly expressed in the liver, the CYP3A4-dependent metabolism of PSI-352938 makes it an effective liver-targeted prodrug, in part accounting for the potent antiviral activity observed clinically.	225 263 08
HIV	DB 002 05	pyrimeth amine	Pyrimethamine, another antiprotozoal medication, demonstrated in vitro antiviral activity against HIV.	352 999 94
SARS-CoV-2	DB 042	quercetin	ANTIVIRAL ACTIVITY OF QUERCETIN	347 096

	16			75
HBV	DB 009 08	quinidine	Primaquine, quinidine, quinine and tafenoquine only blocked coronaviruses replication at higher concentrations, while piperaquine completely lacked antiviral and cytotoxic effect.	342 177 52
HIV	DB 068 17	raltegrav ir	Raltegravir is a first-in-class HIV-1 integrase inhibitor with established antiviral efficacy in treatment-naive and treatment-experienced patients with multidrug-resistant HIV-1 infection.	220 982 78
HCV	DB 068 17	raltegrav ir	Raltegravir is a first-in-class HIV-1 integrase inhibitor with established antiviral efficacy in treatment-naive and treatment-experienced patients with multidrug-resistant HIV-1 infection.	220 982 78
HCV	DB 156 52	ravidasvi r	Antiviral treatments compared in the included studies were umifenovir (Arbidol), baloxavir marboxil, enisamium, favipiravir, lopinavir/ritonavir (LPV/r), remdesivir, ribavirin, sofosbuvir/daclatasvir, sofosbuvir/ledipasvir, sofosbuvir/ravidasvir, and sofosbuvir/velpatasvir.	351 009 85
SARS-CoV-2	DB 147 61	remdesiv ir	Anti-COVID-19 medicines such as Remdesivir (antiviral), Ivermectin (anti-parasitic), Favipiravir (anti-influenza), Hydroxychloroquine (anti-malarial and anti-rheumatic diseases), and arbidol are now being studied (anti-influenza) by various researchers.	356 671 52
HBV	DB 065 30	resiquim od	Oral resiquimod, a TLR7 and TLR8 agonist, showed promising antiviral effects in phase IIa safety and efficacy trials.	186 254 34
HBV	DB 027 09	resveratr ol	Antiviral Mechanisms of Resveratrol	359 900 40
Influenz a A	DB 008 11	ribavirin	Ribavirin antiviral effect partly reduced the rebound preceding the second IFN-alpha injection in patients receiving standard IFN-alpha 3 times per week plus ribavirin.	149 888 24
HIV	DB 008 11	ribavirin	Ribavirin antiviral effect partly reduced the rebound preceding the second IFN-alpha injection in patients receiving standard IFN-alpha 3 times per week plus ribavirin.	149 888 24
HCV	DB 008 11	ribavirin	Ribavirin antiviral effect partly reduced the rebound preceding the second IFN-alpha injection in patients receiving standard IFN-alpha 3 times per week plus ribavirin.	149 888 24
HBV	DB 008 11	ribavirin	Ribavirin antiviral effect partly reduced the rebound preceding the second IFN-alpha injection in patients receiving standard IFN-alpha 3 times per week plus ribavirin.	149 888 24
HIV	DB	rifabutin	Evaluation of the antiviral effect of rifabutin in AIDS-related complex.	254

	006 15			241 9
HIV	DB 010 45	rifampici n	Antiviral action of rifampicin.	418 969 7
HCV	DB 088 64	rilpivirin e	Rilpivirine, a new non-nucleoside reverse transcriptase inhibitor, has shown similar antiviral efficacy to efavirenz in a phase 2b trial with two nucleoside/nucleotide reverse transcriptase inhibitors.	217 639 36
HIV	DB 088 64	rilpivirin e	Rilpivirine, a new non-nucleoside reverse transcriptase inhibitor, has shown similar antiviral efficacy to efavirenz in a phase 2b trial with two nucleoside/nucleotide reverse transcriptase inhibitors.	217 639 36
Influenz a A	DB 004 78	rimantad ine	The antiviral drugs currently available against influenza viruses are amantadine, rimantadine, zanamivir, oseltamivir, peramivir, and baloxavir marboxil.	330 297 61
HCV	DB 005 03	ritonavir	Ritonavir, an HIV-1 protease inhibitor, has pharmacokinetic properties that enhance the activity of concomitantly administered direct acting antivirals against HCV.	258 463 01
HBV	DB 005 03	ritonavir	Ritonavir, an HIV-1 protease inhibitor, has pharmacokinetic properties that enhance the activity of concomitantly administered direct acting antivirals against HCV.	258 463 01
HHV	DB 005 03	ritonavir	Ritonavir, an HIV-1 protease inhibitor, has pharmacokinetic properties that enhance the activity of concomitantly administered direct acting antivirals against HCV.	258 463 01
HIV	DB 005 03	ritonavir	Ritonavir, an HIV-1 protease inhibitor, has pharmacokinetic properties that enhance the activity of concomitantly administered direct acting antivirals against HCV.	258 463 01
HCV	DB 004 12	rosiglitaz one	The reference drugs chosen for inhibiting TMPSSR2 were camostat mesylate and gabexate mesylate, and the drugs that could be repurposed as potential inhibitors of TMPSSR2 included danoprevir (antiviral), ezetimibe (cholesterol lowering drug), methyl prednisolone (corticosteroid), mevastatin (statin), pitavastatin (statin), rosiglitazone (antidiabetic), shikonin (natural compound), tideglusib (glycogen synthase kinase 3 inhibitor), pacritinib (janus kinase inhibitor), warfarin (anticoagulant), dipyridamole/aspirin (antiplatelet), cilostazol (antiplatelet) and natural molecules dithymoquinone, fucosterol, campesterol.	336 550 82
HIV	DB 010 98	rosuvasta tin	To investigate the efficacy of other commonly prescribed statins, we compared the antiviral activities of lovastatin, fluvastatin, simvastatin, atorvastatin, rosuvastatin, and pitavastatin in Huh7 cells.	297 170 11
Influenz	DB	rosuvasta	To investigate the efficacy of other commonly prescribed statins, we compared the antiviral activities of lovastatin, fluvastatin,	297

a A	010 98	tin	simvastatin, atorvastatin, rosuvastatin, and pitavastatin in Huh7 cells.	170 11
HCV	DB 126 60	samatasv ir	Samatasvir, a selective inhibitor of HCV replication in vitro with picomolar activity, was also one of the benzimidazole derivatives that combine with other antiviral drugs in the clinical therapy of HCV-infected patients.	347 075 97
HIV	DB 007 47	scopola mine	Thus, the present study was conducted to assess the antiviral effects of scopolamine and its immunomodulatory role against Japanese encephalitis virus (JEV) infections in embryonated chick.	336 206 66
SARS-CoV-2	DB 119 42	selinexor	Antiviral and Anti-Inflammatory Effects of Selinexor	355 592 57
HIV	DB 011 04	sertraline	Also, Imipramine, Sertraline, and Paroxetine showed antiviral activity against MERS-CoV.	359 026 47
HCV	DB 120 51	setrobuvi r	A recent study evaluated the antiviral activity of six HCV NNIs against the human norovirus RdRp132 including; Filibuvir, JTK-109, Lomibuvir, Nesbuvir, Setrobuvir, and Tegobuvir using a fluorescent polymerase assay.	305 848 00
SARS-CoV-2	DB 002 03	sildenafil	Having previously fast-tracked this paper to publication in summary form, we now expand on why cimetidine/famotidine (histamine type-2 receptor antagonists), dipyridamole (antiplatelet agent), fenofibrate/bezafibrate (cholesterol/triglyceride-lowering agents), and sildenafil (phosphodiesterase-5 inhibitor) are worth considering for patients with COVID-19 based on their antiviral, anti-inflammatory, renoprotective, cardioprotective, and anticoagulation properties.	337 242 65
HCV	DB 092 98	silibinin	Silybin (silibinin, silymarin) has shown antiviral activity against hepatitis C virus and chikungunya virus.	271 514 82
HCV	DB 062 90	simeprev ir	Simeprevir (SMV) is a second-generation oral HCV NS3/4A protease inhibitor with antiviral activity against HCV genotype 1, 2, 4, 5, and 6 infections.	285 398 15
HBV	DB 006 41	simvastat in	Fluvastatin or simvastatin has demonstrable antiviral activity against hepatitis C virus (HCV) as monotherapy.	239 106 46
HCV	DB 006 41	simvastat in	Fluvastatin or simvastatin has demonstrable antiviral activity against hepatitis C virus (HCV) as monotherapy.	239 106 46
HIV	DB 012	sitaglipti n	Significant antiviral activity of the sitagliptin and melittin-nanoconjugates complex (IC50 value-8.439 microM) was observed against SARS-CoV-2 in vitro.	352 369

	61			09
HCV	DB 089 34	sofosbuv ir	Simeprevir (SMV), asunaprevir (ASV), daclatasvir (DCV), and sofosbuvir (SFV), which are newly developed direct-acting antiviral agents (DAAs) against hepatitis C virus (HCV) infection, are among the key components of anti-HCV regimens.	248 679 84
HIV	DB 089 34	sofosbuv ir	Simeprevir (SMV), asunaprevir (ASV), daclatasvir (DCV), and sofosbuvir (SFV), which are newly developed direct-acting antiviral agents (DAAs) against hepatitis C virus (HCV) infection, are among the key components of anti-HCV regimens.	248 679 84
HBV	DB 089 34	sofosbuv ir	Simeprevir (SMV), asunaprevir (ASV), daclatasvir (DCV), and sofosbuvir (SFV), which are newly developed direct-acting antiviral agents (DAAs) against hepatitis C virus (HCV) infection, are among the key components of anti-HCV regimens.	248 679 84
HBV	DB 003 98	sorafenib	The antiviral effect of sorafenib in hepatitis c-related hepatocellular carcinoma	230 948 60
HCV	DB 120 69	sovaprev ir	According to our screening and selection criteria, three approved antivirals (elbasvir, grazoprevir, and sovaprevir) and 4 other drugs (hesperidin, pamaqueside, diosmin, and sitogluside) were identified as potent binders of the RBD.	331 302 79
HSV	DB 004 21	spironola ctone	Androgen receptor inhibitors like spironolactone, eplerenone are having extensive safety profile against hypertension and inhibits aldosterone actions, they also provide possible antiviral and anti-inflammatory actions that reduce lung injuries.	355 178 88
HIV	DB 006 49	stavudin e	Anti-HIV antiviral activity of stavudine in a thymidine kinase-deficient cellular line	106 821 38
SARS-CoV-2	DB 012 99	sulfadoxi ne	we analyze the anti-ZIKV effect of seven hybrid compounds derived from chloroquine and sulfadoxine antimalarial drugs, and, for the first time, we showed the high effectiveness of these quinolinic derivatives against ZIKV-BR in cervical and Vero cells, with 100% viral progeny inhibition.	333 836 19
HHV	DB 008 64	tacrolim us	Treatment with immunosuppressive agents, tacrolimus (Prograf), mycophenolate mofetil (CellCept), and prednisolone, and antiviral agents, entecavir (Baraclude) as prophylaxis therapy against hepatitis B virus reactivation, was started.	294 302 21
HCV	DB 008 64	tacrolim us	Treatment with immunosuppressive agents, tacrolimus (Prograf), mycophenolate mofetil (CellCept), and prednisolone, and antiviral agents, entecavir (Baraclude) as prophylaxis therapy against hepatitis B virus reactivation, was started.	294 302 21
HCV	DB 006 75	tamoxife n	Toremifene, a first generation SERM, exhibits potential antiviral effects against MERS-CoV, SARS-CoV, and Ebola virus in vitro, while other popular SERMs used for breast cancer, such as tamoxifen, also have potent anti-CoVs activities.	325 689 69
HCV	DB	taribaviri	Taribavirin (also known as viramidine) is an antiviral drug in Phase III human trials, but not yet approved for pharmaceutical use.	326

	064 08	n		351 56
VARV	DB 120 20	tecoviri mat	Tecovirimat, an antiviral drug, was found potentially to be beneficial in treating smallpox virus infection.	361 155 29
HCV	DB 118 52	tegobuvi r	Tegobuvir (GS-9190) by itself and in combination with other anti-HCV drugs were assessed in some clinical trials and showed high potency in viral load reduction with no significant cytotoxicity (Wyles et al.,).	335 991 80
HCV	DB 055 21	telaprevi r	Telaprevir antiviral resistance	231 880 91
HBV	DB 012 65	telbivudi ne	Antiviral therapy included lamivudine, telbivudine, adefovir, tenofovir, or entecavir.	265 950 58
HIV	DB 009 66	telmisart an	For some of the selected drugs clinical data for COVID-19 supports antiviral activity (salvianolic acid B, thymopentin, atorvastatin, montelukast, dipyridamole, lopinavir, ritonavir, telmisartan, daclatasavir, atazanavir, nintedanib, darunavir and ramipril).	337 870 66
HBV	DB 141 26	tenofovir	The most frequent antiviral drugs used for HBV are nucleoside or nucleotide analogs (NUCs), such as entecavir, tenofovir disoproxil or tenofovir alafenamide.	337 821 08
HIV	DB 141 26	tenofovir	The most frequent antiviral drugs used for HBV are nucleoside or nucleotide analogs (NUCs), such as entecavir, tenofovir disoproxil or tenofovir alafenamide.	337 821 08
HCV	DB 141 26	tenofovir	The most frequent antiviral drugs used for HBV are nucleoside or nucleotide analogs (NUCs), such as entecavir, tenofovir disoproxil or tenofovir alafenamide.	337 821 08
HBV	DB 092 99	tenofovir alafenam ide	Furthermore, tenofovir alafenamide fumarate (TAF), a tenofovir pro-drug that is easily incorporated into HIV-1-infected cells and shows similar antiviral efficacy as TDF with less adverse effects on the kidneys at a low dose, is currently used for treating both HIV-1- and HBV-infected patients.	301 743 10
HIV	DB 092 99	tenofovir alafenam ide	Furthermore, tenofovir alafenamide fumarate (TAF), a tenofovir pro-drug that is easily incorporated into HIV-1-infected cells and shows similar antiviral efficacy as TDF with less adverse effects on the kidneys at a low dose, is currently used for treating both HIV-1- and HBV-infected patients.	301 743 10
HCV	DB 092 99	tenofovir alafenam ide	Furthermore, tenofovir alafenamide fumarate (TAF), a tenofovir pro-drug that is easily incorporated into HIV-1-infected cells and shows similar antiviral efficacy as TDF with less adverse effects on the kidneys at a low dose, is currently used for treating both HIV-1- and HBV-infected patients.	301 743 10

SARS-CoV-2	DB 140 66	tetrandri ne	A previous study found that tetrandrine was a potential natural antiviral agent for the prevention and treatment of infection with HCoV-OC43, which is closely related to SARS-CoV and which shares several functional properties.	361 438 71
HBV	DB 049 00	thymalfa sin	Thymalfasin: clinical pharmacology and antiviral applications.	180 205 80
HCV	DB 049 00	thymalfa sin	Thymalfasin: clinical pharmacology and antiviral applications.	180 205 80
HBV	DB 179 65	tilorone	Tilorone, a Broad-Spectrum Antiviral for Emerging Viruses	322 053 50
HCV	DB 179 65	tilorone	Tilorone, a Broad-Spectrum Antiviral for Emerging Viruses	322 053 50
HSV	DB 179 65	tilorone	Tilorone, a Broad-Spectrum Antiviral for Emerging Viruses	322 053 50
Influenz a A	DB 179 65	tilorone	Tilorone, a Broad-Spectrum Antiviral for Emerging Viruses	322 053 50
HIV	DB 009 32	tipranavi r	Most importantly, the two most recently approved PIs, tipranavir (TPV) and darunavir (DRV), retain high antiviral potency against viral strains resistant to most other PIs.	209 580 50
HCV	DB 118 22	tmc-647055	Based on the reweighted virtual screening results (Table 3), obtained though the evaluation of the previous literature, we propose the concurrent administration of four compounds: phthalocyanine, hypericin, TMC-647055 and quarfloxin, to be used as a possible antiviral treatment against SARS-CoV-2.	325 651 26
HCV	DB 011 24	tolbutam ide	In contrast, non-immune-mediated DIT occurs through bone marrow suppression, usually from antineoplastic medications (alkylating agents, antimetabolites, and cytotoxic drugs), although several antiviral agents, tolbutamide, and thiazide diuretics have also been implicated.	204 696 10
SARS-CoV-2	DB 156 22	triazaviri n	[Investigation of triazavirin antiviral activity against influenza A virus (H5N1) in cell culture].	192 750 52
SARS-CoV-2	DB 086	triclosan	Triclosan is broad-spectrum antibacterial compound, with some antifungal and antiviral activity.	343 191

	04			68
HSV	DB 004 32	trifluridi ne	Currently, aciclovir, trifluridine and valaciclovir are commonly used as antiviral agents to treat herpesvirus infections.	213 198 70
HIV	DB 004 40	trimetho prim	Trimethoprim-sulfamethoxazole is advantageous as a broad spectrum antibacterial and antifungal agent, while some believe it also possesses direct antiviral and anti-inflammatory properties.	328 381 33
HCV	DB 152 06	uprifosb uvir	Ruzasvir (MK-8408, an NS5A inhibitor) and uprifosbuvir (MK-3682, a nonstructural protein 5B nucleotide inhibitor) are highly potent direct-acting antiviral agents for the treatment of hepatitis C virus (HCV) infection.	311 080 15
HCV	DB 015 86	ursodeox ycholic acid	Other chemoprevention strategies have been investigated to prevent development of HCC in patients after curative treatment with antiviral therapy, including glycyrrhizin and ursodeoxycholic acid.	260 793 99
HHV	DB 005 77	valaciclo vir	Herpetic reactivation can be prevented with oral antivirals (acyclovir, famciclovir or valaciclovir) [Figure 4].	266 447 36
HIV	DB 005 77	valaciclo vir	Herpetic reactivation can be prevented with oral antivirals (acyclovir, famciclovir or valaciclovir) [Figure 4].	266 447 36
HSV	DB 005 77	valaciclo vir	Herpetic reactivation can be prevented with oral antivirals (acyclovir, famciclovir or valaciclovir) [Figure 4].	266 447 36
HCV	DB 005 77	valaciclo vir	Herpetic reactivation can be prevented with oral antivirals (acyclovir, famciclovir or valaciclovir) [Figure 4].	266 447 36
HSV	DB 005 77	valacycl ovir hydrochl oride	Therapy for symptomatic HSV relies on antiviral drugs: acyclovir, valacyclovir hydrochloride, and famciclovir.	325 414 59
SARS-CoV-2	DB 003 13	valproic acid	Valproic acid (VPA) is a branched short-chain fatty acid clinically used as a broad-spectrum antiepileptic drug in the treatment of neurological disorders, which has shown promising antiviral activity against some herpesviruses.	332 561 72
SARS-CoV-2	DB 001 77	valsartan	Valsartan is a well-known antihypertensive drug that exhibits antiviral activity against CVB3 in association with its effect on downregulating CAR in human endothelial cells.	355 731 53

HBV	DB 061 87	valtorcitabine	In addition, several novel anti-HBV agents recently evaluated in phase II clinical trials, such as tenofovir, clevudine, telbivudine, pradefovir, and valtorcitabine, appear to be promising agents for the treatment of chronic hepatitis B. This article describes the clinical experience with new antiviral agents and the implications of the trial results for practice guidelines.	16103979
HCV	DB 119 29	vaniprevir	Characterization of vaniprevir, a hepatitis C virus NS3/4A protease inhibitor, in patients with HCV genotype 1 infection: safety, antiviral activity, resistance, and pharmacokinetics.	23747481
HCV	DB 120 37	vedroprevir	Vedroprevir was discovered among a new class of carboxylic acid HCV NS3 protease inhibitors, and was noted for its high antiviral activity, bioavailability, selectivity, and low toxicity.	29053394
HIV	DB 116 13	velpatasvir	Velpatasvir (VEL) is a second-generation HCV NS5A inhibitor with antiviral activity against HCV replicons in genotypes 1 through 6.	29427484
HCV	DB 116 13	velpatasvir	Velpatasvir (VEL) is a second-generation HCV NS5A inhibitor with antiviral activity against HCV replicons in genotypes 1 through 6.	29427484
SARS-CoV-2	DB 002 85	venlafaxine	In a new study, antidepressants (i.e., fluvoxamine, fluoxetine, citalopram, paroxetine, venlafaxine, reboxetine, clomipramine, and imipramine) had antiviral effects in SARS-CoV-2-infected cells.	34997196
SARS-CoV-2	DB 006 61	verapamil	We have found that Verapamil, a calcium channel blocker, inhibits influenza virus replication in Madin-Darby canine kidney cells and in murine pulmonary macrophages and that this antiviral effect occurs with drug addition late in the replication cycle.	6743023
HBV	DB 126 87	vesatolimod	GS-9620 (Vesatolimod) is currently being evaluated for safety, tolerability and antiviral activity in HIV-1 viremic controllers on ART and subsequently during ATI (NCT03060447).	34630386
HIV	DB 126 87	vesatolimod	GS-9620 (Vesatolimod) is currently being evaluated for safety, tolerability and antiviral activity in HIV-1 viremic controllers on ART and subsequently during ATI (NCT03060447).	34630386
HIV	DB 025 46	vorinostat	Recently Vorinostat has performed well as an antiviral therapy for HIV and West Nile Virus.	35341048
HCV	DB 120 26	voxilaprevir	The NS3 protease inhibitor voxilaprevir, NS5A second generation inhibitor velpatasvir, and NS5B nucleoside analog inhibitor sofosbuvir, had similar antiviral activities across the different genotypes/subtypes tested, while the NS5A first generation inhibitor, ledipasvir, had very good antiviral activity against GT1, 4, 5, and 6 in vitro.	29175627
HIV	DB 009	zalcitabine	Zalcitabine is an antiviral drug for human immunodeficiency virus (HIV)-infected patients.	356647

	43			78
Influenza B	DB00558	zanamivir	Influenza virus neuraminidase inhibitors (NAIs), including oseltamivir, zanamivir and peramivir, are important antivirals for the treatment and prophylaxis of influenza.	26400894
Influenza A	DB00558	zanamivir	Influenza virus neuraminidase inhibitors (NAIs), including oseltamivir, zanamivir and peramivir, are important antivirals for the treatment and prophylaxis of influenza.	26400894
HIV	DB00497	zidovudine	CONCLUSION: Long-term HIV therapy with efavirenz-containing regimens, particularly efavirenz, zidovudine, lamivudine, provides significantly greater antiviral activity and tolerability than a regimen of indinavir, zidovudine plus lamivudine.	18097230
HBV	DB00495	zidovudine	CONCLUSION: Long-term HIV therapy with efavirenz-containing regimens, particularly efavirenz, zidovudine, lamivudine, provides significantly greater antiviral activity and tolerability than a regimen of indinavir, zidovudine plus lamivudine.	18097230
HHV	DB00495	zidovudine	CONCLUSION: Long-term HIV therapy with efavirenz-containing regimens, particularly efavirenz, zidovudine, lamivudine, provides significantly greater antiviral activity and tolerability than a regimen of indinavir, zidovudine plus lamivudine.	18097230
SARS-CoV-2	DB16407	azvudine	Azvudine is a thymus-homing anti-SARS-CoV-2 drug effective in treating COVID-19 patients.	34873151
SARS-CoV-2	DB01294	bismuth subsalicylate	BSS has been shown to have antibacterial and antiviral activity	35370296
HCV	DB14950	bms-929075	Discovery of a Hepatitis C Virus NS5B Replicase Palm Site Allosteric Inhibitor (BMS-929075) Advanced to Phase 1 Clinical Studies	28430437
HIV	DB00921	buprenorphine	The Effect of Buprenorphine on Human Immunodeficiency Virus Viral Suppression.	34171087
HCV	DB00921	buprenorphine	The Effect of Buprenorphine on Human Immunodeficiency Virus Viral Suppression.	34171087
SARS-CoV-2	DB00490	buspirone	Buspirone docked with 3-chymotrypsin-like protease (3CLpro) of SARS-CoV-2	32834113
HIV	DB	censavud	TPN-101 (OBP-601, BMS-986001, festinavir, censavudine; Smith et al.,) is a thymidine analog NRTI (nucleoside reverse	357

	120 74	ine	transcriptase inhibitor) that shows major efficacy against HIV-2 and HIV-1 (Gupta et al.,).	549 52
SARS-CoV-2	DB 010 03	cromoglic acid	Scaffold Hopping of alpha-Rubromycin Enables Direct Access to FDA-Approved Cromoglicic Acid as a SARS-CoV-2 MPro Inhibitor.	341 989 33
SARS-CoV-2	DB 008 76	eprosartan	The antiviral activity of eprosartan (compound selected in silico) towards highly and low-virulent strains of tick-borne encephalitis virus was compared in vitro with activity of ribavirin.	332 220 83
SARS-CoV-2	DB 118 27	ertugliflozin	Identification of SGLT2 inhibitor Ertugliflozin as a treatment for COVID-19 using computational and experimental paradigm	235 497 799
HIV	DB 010 39	fenofibrate	The role of fenofibrate in the treatment of COVID-19	347 455 95
HHV	DB 124 38	filociclovir	Filociclovir (FCV; also known as cyclopropavir or MBX-400) is a methylenecyclopropane nucleoside analog with broad-spectrum antiviral activity, whose targets include HCMV, VZV, EBV, HHV-6A, HHV-6B, HHV-7 and HHV-8.	362 962 90
HIV	DB 061 66	fosdevirine	Fosdevirine (GSK2248761) is a non-nucleoside reverse transcriptase inhibitor with HIV-1 activity against common efavirenz-resistant strains	241 585 93
HBV	DB 150 63	inarigivir soproxil	SB9200 (inarigivir soproxil), a dinucleotide antiviral compound, can activate intracellular innate immunity via enabling DDX58/RIG-I.	360 044 88
SARS-CoV-2	DB 066 36	isavuconazonium	Targeting the SARS-CoV-2 main protease using FDA-approved Isavuconazonium, a P2??????3 ???-ketoamide derivative and Pentagastrin: An in-silico drug discovery approach	329 202 39
SARS-CoV-2	DB 010 97	leflunomide	A Small-Scale Medication of Leflunomide as a Treatment of COVID-19 in an Open-Label Blank-Controlled Clinical Trial	326 963 96
HCV	DB 119 54	lomibuvir	Lomibuvir is a non-nucleoside reverse transcriptase inhibitor that is also known to inhibit HCV RdRP.	352 177 18
HIV	DB 015 77	metamfetamine	Methamphetamine Reduces Human Influenza A Virus Replication	231 397 74

SARS-CoV-2	DB 006 22	nicardipi ne	Moreover, an array of calcium channel inhibitors, such as cilnidipine, felodipine, amlodipine, manidipine, nicardipine, and nisoldipine, exhibit similar antiviral properties.	361 052 08
HSV	DB 011 15	nifedipin e	Moreover, an array of calcium channel inhibitors, such as cilnidipine, felodipine, amlodipine, manidipine, nicardipine, and nisoldipine, exhibit similar antiviral properties.	361 052 08
SARS-CoV-2	DB 120 16	ponesim od	Ponesimod is a promising anti-HBV drug targeting the endosome maturation of HBV	333 460 55
SARS-CoV-2	DB 160 65	proxaluta mide	Proxalutamide Significantly Accelerates Viral Clearance and Reduces Time to Clinical Remission in Patients with Mild to Moderate COVID-19: Results from a Randomized, Double-Blinded, Placebo-Controlled Trial	336 339 20
HCV	DB 120 65	radalbuvi r	Radalbuvir is a reported inhibitor of hepatitis C NS5B polymerase and is currently in phase II clinical trials for oral use.	354 785 53
HIV	DB 061 76	romideps in	Pharmacokinetic/pharmacodynamic analysis of romidepsin used as an HIV latency reversing agent.	333 677 67
SARS-CoV-2	DB 048 47	roxadust at	Hypoxia and Roxadustat inhibit SARS-CoV-2 RNA replication, showing that post-entry steps in the viral life cycle are oxygen sensitive.	338 529 16
HCV	DB 117 13	ruzasvir	In Vitro Antiviral Profile of Ruzasvir, a Potent and Pangenotype Inhibitor of Hepatitis C Virus NS5A.	301 504 66
SARS-CoV-2	DB 088 16	ticagrelo r	he results suggest the effectiveness of vorapaxar, ticagrelor, cilostazol, cangrelor, and prasugrel in binding the main protease (Mpro) of SARS-CoV-2.	345 103 37
HCV	DB 139 20	valopicit abine	Here we demonstrate that the active component of the anti-HCV drug valopicitabine inhibits the replication of all 3 PV strains.	183 942 70

Table S3. Feature components used to construct the five optimal selective antiviral drug model (SAM)

Feature location	Feature selection method	Cutoff	note
X55	Fisher's exact test & t-test	0.01	viral genome sequence
X90	Fisher's exact test & t-test	0.01	viral genome sequence
X13	Fisher's exact test & t-test	0.01	viral genome sequence
X36	Fisher's exact test & t-test	0.01	viral genome sequence
X65	Fisher's exact test & t-test	0.01	viral genome sequence
X58	Fisher's exact test & t-test	0.01	viral genome sequence
X86	Fisher's exact test & t-test	0.01	viral genome sequence
X98	Fisher's exact test & t-test	0.01	viral genome sequence
X71	Fisher's exact test & t-test	0.01	viral genome sequence
X56	Fisher's exact test & t-test	0.01	viral genome sequence
X83	Fisher's exact test & t-test	0.01	viral genome sequence
X34	Fisher's exact test & t-test	0.01	viral genome sequence
X18	Fisher's exact test & t-test	0.01	viral genome sequence
X82	Fisher's exact test & t-test	0.01	viral genome sequence
X40	Fisher's exact test & t-test	0.01	viral genome sequence
X69	Fisher's exact test & t-test	0.01	viral genome sequence
X17	Fisher's exact test & t-test	0.01	viral genome sequence
X32	Fisher's exact test & t-test	0.01	viral genome sequence
X27	Fisher's exact test & t-test	0.01	viral genome sequence
X26	Fisher's exact test & t-test	0.01	viral genome sequence
X79	Fisher's exact test & t-test	0.01	viral genome sequence
X53	Fisher's exact test & t-test	0.01	viral genome sequence
X73	Fisher's exact test & t-test	0.01	viral genome sequence
X68	Fisher's exact test & t-test	0.01	viral genome sequence
X4	Fisher's exact test & t-test	0.01	viral genome sequence
V243	Fisher's exact test & t-test	0.01	ECFP4
X60	Fisher's exact test & t-test	0.01	viral genome sequence
X59	Fisher's exact test & t-test	0.01	viral genome sequence
X46	Fisher's exact test & t-test	0.01	viral genome sequence
X8	Fisher's exact test & t-test	0.01	viral genome sequence
V834	Fisher's exact test & t-test	0.01	ECFP4
X66	Fisher's exact test & t-test	0.01	viral genome sequence
V935	Fisher's exact test & t-test	0.01	ECFP4
V784	Fisher's exact test & t-test	0.01	ECFP4
X20	Fisher's exact test & t-test	0.01	viral genome sequence
V492	Fisher's exact test & t-test	0.01	ECFP4
X96	Fisher's exact test & t-test	0.01	viral genome sequence
V181	Fisher's exact test & t-test	0.01	ECFP4
V807	Fisher's exact test & t-test	0.01	ECFP4
X61	Fisher's exact test & t-test	0.01	viral genome sequence
V853	Fisher's exact test & t-test	0.01	ECFP4

V587	Fisher's exact test & t-test	0.01	ECFP4
V277	Fisher's exact test & t-test	0.01	ECFP4
V903	Fisher's exact test & t-test	0.01	ECFP4
X2	Fisher's exact test & t-test	0.01	viral genome sequence
V443	Fisher's exact test & t-test	0.01	ECFP4
X74	Fisher's exact test & t-test	0.01	viral genome sequence
V552	Fisher's exact test & t-test	0.01	ECFP4
X72	Fisher's exact test & t-test	0.01	viral genome sequence
V556	Fisher's exact test & t-test	0.01	ECFP4
V943	Fisher's exact test & t-test	0.01	ECFP4
V138	Fisher's exact test & t-test	0.01	ECFP4
X100	Fisher's exact test & t-test	0.01	viral genome sequence
X78	Fisher's exact test & t-test	0.01	viral genome sequence
V34	Fisher's exact test & t-test	0.01	ECFP4
V918	Fisher's exact test & t-test	0.01	ECFP4
X54	Fisher's exact test & t-test	0.01	viral genome sequence
V757	Fisher's exact test & t-test	0.01	ECFP4
V685	Fisher's exact test & t-test	0.01	ECFP4
V654	Fisher's exact test & t-test	0.01	ECFP4
X50	Fisher's exact test & t-test	0.01	viral genome sequence
V399	Fisher's exact test & t-test	0.01	ECFP4
V881	Fisher's exact test & t-test	0.01	ECFP4
X85	Fisher's exact test & t-test	0.01	viral genome sequence
V610	Fisher's exact test & t-test	0.01	ECFP4
V225	Fisher's exact test & t-test	0.01	ECFP4
V224	Fisher's exact test & t-test	0.01	ECFP4
V988	Fisher's exact test & t-test	0.01	ECFP4
X88	Fisher's exact test & t-test	0.01	viral genome sequence
X42	Fisher's exact test & t-test	0.01	viral genome sequence
V38	Fisher's exact test & t-test	0.01	ECFP4
V109	Fisher's exact test & t-test	0.01	ECFP4
X33	Fisher's exact test & t-test	0.01	viral genome sequence
V435	Fisher's exact test & t-test	0.01	ECFP4
X70	Fisher's exact test & t-test	0.01	viral genome sequence
V695	Fisher's exact test & t-test	0.01	ECFP4
X76	Fisher's exact test & t-test	0.01	viral genome sequence
V456	Fisher's exact test & t-test	0.01	ECFP4
V793	Fisher's exact test & t-test	0.01	ECFP4
X97	Fisher's exact test & t-test	0.01	viral genome sequence
V737	Fisher's exact test & t-test	0.01	ECFP4
V555	Fisher's exact test & t-test	0.01	ECFP4
V64	Fisher's exact test & t-test	0.01	ECFP4
V682	Fisher's exact test & t-test	0.01	ECFP4

V759	Fisher's exact test & t-test	0.01	ECFP4
V173	Fisher's exact test & t-test	0.01	ECFP4
V999	Fisher's exact test & t-test	0.01	ECFP4
V223	Fisher's exact test & t-test	0.01	ECFP4
V835	Fisher's exact test & t-test	0.01	ECFP4
V572	Fisher's exact test & t-test	0.01	ECFP4
V1010	Fisher's exact test & t-test	0.01	ECFP4
V211	Fisher's exact test & t-test	0.01	ECFP4
V331	Fisher's exact test & t-test	0.01	ECFP4
V493	Fisher's exact test & t-test	0.01	ECFP4
X19	Fisher's exact test & t-test	0.01	viral genome sequence
X39	Fisher's exact test & t-test	0.01	viral genome sequence
V479	Fisher's exact test & t-test	0.01	ECFP4
V596	Fisher's exact test & t-test	0.01	ECFP4
V623	Fisher's exact test & t-test	0.01	ECFP4
V636	Fisher's exact test & t-test	0.01	ECFP4
V797	Fisher's exact test & t-test	0.01	ECFP4
V275	Fisher's exact test & t-test	0.01	ECFP4
V513	Fisher's exact test & t-test	0.01	ECFP4
V450	Fisher's exact test & t-test	0.01	ECFP4
V655	Fisher's exact test & t-test	0.01	ECFP4
X93	Fisher's exact test & t-test	0.01	viral genome sequence
V543	Fisher's exact test & t-test	0.01	ECFP4
V197	Fisher's exact test & t-test	0.01	ECFP4
V657	Fisher's exact test & t-test	0.01	ECFP4
X94	Fisher's exact test & t-test	0.01	viral genome sequence
V1003	Fisher's exact test & t-test	0.01	ECFP4
V460	Fisher's exact test & t-test	0.01	ECFP4
V365	Fisher's exact test & t-test	0.01	ECFP4
V408	Fisher's exact test & t-test	0.01	ECFP4
V699	Fisher's exact test & t-test	0.01	ECFP4
X91	Fisher's exact test & t-test	0.01	viral genome sequence
X21	Fisher's exact test & t-test	0.01	viral genome sequence
V366	Fisher's exact test & t-test	0.01	ECFP4
V773	Fisher's exact test & t-test	0.01	ECFP4
V725	Fisher's exact test & t-test	0.01	ECFP4
V7	Fisher's exact test & t-test	0.01	ECFP4
V499	Fisher's exact test & t-test	0.01	ECFP4
V769	Fisher's exact test & t-test	0.01	ECFP4
V707	Fisher's exact test & t-test	0.01	ECFP4
V294	Fisher's exact test & t-test	0.01	ECFP4
V463	Fisher's exact test & t-test	0.01	ECFP4
V553	Fisher's exact test & t-test	0.01	ECFP4

V519	Fisher's exact test & t-test	0.01	ECFP4
V742	Fisher's exact test & t-test	0.01	ECFP4
V579	Fisher's exact test & t-test	0.01	ECFP4
X25	Fisher's exact test & t-test	0.01	viral genome sequence
V788	Fisher's exact test & t-test	0.01	ECFP4
V684	Fisher's exact test & t-test	0.01	ECFP4
V150	Fisher's exact test & t-test	0.01	ECFP4
V105	Fisher's exact test & t-test	0.01	ECFP4
V535	Fisher's exact test & t-test	0.01	ECFP4
V415	Fisher's exact test & t-test	0.01	ECFP4
V663	Fisher's exact test & t-test	0.01	ECFP4
X80	Fisher's exact test & t-test	0.01	viral genome sequence
V198	Fisher's exact test & t-test	0.01	ECFP4
V482	Fisher's exact test & t-test	0.01	ECFP4
V253	Fisher's exact test & t-test	0.01	ECFP4
V396	Fisher's exact test & t-test	0.01	ECFP4
V432	Fisher's exact test & t-test	0.01	ECFP4
V247	Fisher's exact test & t-test	0.01	ECFP4
V714	Fisher's exact test & t-test	0.01	ECFP4
V485	Fisher's exact test & t-test	0.01	ECFP4
V352	Fisher's exact test & t-test	0.01	ECFP4
V373	Fisher's exact test & t-test	0.01	ECFP4
V476	Fisher's exact test & t-test	0.01	ECFP4
V185	Fisher's exact test & t-test	0.01	ECFP4
V148	Fisher's exact test & t-test	0.01	ECFP4
V524	Fisher's exact test & t-test	0.01	ECFP4
V806	Fisher's exact test & t-test	0.01	ECFP4
V551	Fisher's exact test & t-test	0.01	ECFP4
V750	Fisher's exact test & t-test	0.01	ECFP4
V219	Fisher's exact test & t-test	0.01	ECFP4
V338	Fisher's exact test & t-test	0.01	ECFP4
V29	Fisher's exact test & t-test	0.01	ECFP4
V8	Fisher's exact test & t-test	0.01	ECFP4
V602	Fisher's exact test & t-test	0.01	ECFP4
X52	Fisher's exact test & t-test	0.01	viral genome sequence
V15	Fisher's exact test & t-test	0.01	ECFP4
V142	Fisher's exact test & t-test	0.01	ECFP4
V16	Fisher's exact test & t-test	0.01	ECFP4
V12	Fisher's exact test & t-test	0.01	ECFP4
V121	Fisher's exact test & t-test	0.01	ECFP4
V736	Fisher's exact test & t-test	0.01	ECFP4
V436	Fisher's exact test & t-test	0.01	ECFP4
V600	Fisher's exact test & t-test	0.01	ECFP4

V951	Fisher's exact test & t-test	0.01	ECFP4
V826	Fisher's exact test & t-test	0.01	ECFP4
V111	Fisher's exact test & t-test	0.01	ECFP4
V781	Fisher's exact test & t-test	0.01	ECFP4
V129	Fisher's exact test & t-test	0.01	ECFP4
V314	Fisher's exact test & t-test	0.01	ECFP4
V635	Fisher's exact test & t-test	0.01	ECFP4
V161	Fisher's exact test & t-test	0.01	ECFP4
V328	Fisher's exact test & t-test	0.01	ECFP4
V870	Fisher's exact test & t-test	0.01	ECFP4
V276	Fisher's exact test & t-test	0.01	ECFP4
V32	Fisher's exact test & t-test	0.01	ECFP4
V286	Fisher's exact test & t-test	0.01	ECFP4
X99	Fisher's exact test & t-test	0.01	viral genome sequence
X47	Fisher's exact test & t-test	0.01	viral genome sequence
V818	Fisher's exact test & t-test	0.01	ECFP4
V55	Fisher's exact test & t-test	0.01	ECFP4
V345	Fisher's exact test & t-test	0.01	ECFP4
V528	Fisher's exact test & t-test	0.01	ECFP4
V927	Fisher's exact test & t-test	0.01	ECFP4
V110	Fisher's exact test & t-test	0.01	ECFP4
V155	Fisher's exact test & t-test	0.01	ECFP4
V240	Fisher's exact test & t-test	0.01	ECFP4
V837	Fisher's exact test & t-test	0.01	ECFP4
V371	Fisher's exact test & t-test	0.01	ECFP4
X48	Fisher's exact test & t-test	0.01	viral genome sequence
V571	Fisher's exact test & t-test	0.01	ECFP4
V168	Fisher's exact test & t-test	0.01	ECFP4
V886	Fisher's exact test & t-test	0.01	ECFP4
V71	Fisher's exact test & t-test	0.01	ECFP4
V954	Fisher's exact test & t-test	0.01	ECFP4
V1011	Fisher's exact test & t-test	0.01	ECFP4
V680	Fisher's exact test & t-test	0.01	ECFP4
V845	Fisher's exact test & t-test	0.01	ECFP4
V509	Fisher's exact test & t-test	0.01	ECFP4
X5	Fisher's exact test & t-test	0.01	viral genome sequence
V42	Fisher's exact test & t-test	0.01	ECFP4
V730	Fisher's exact test & t-test	0.01	ECFP4
V263	Fisher's exact test & t-test	0.01	ECFP4
V239	Fisher's exact test & t-test	0.01	ECFP4
V183	Fisher's exact test & t-test	0.01	ECFP4
V431	Fisher's exact test & t-test	0.01	ECFP4
V902	Fisher's exact test & t-test	0.01	ECFP4

V347	Fisher's exact test & t-test	0.01	ECFP4
V141	Fisher's exact test & t-test	0.01	ECFP4
X84	Fisher's exact test & t-test	0.01	viral genome sequence
V376	Fisher's exact test & t-test	0.01	ECFP4
X95	Fisher's exact test & t-test	0.01	viral genome sequence
V213	Fisher's exact test & t-test	0.01	ECFP4
V252	Fisher's exact test & t-test	0.01	ECFP4
V77	Fisher's exact test & t-test	0.01	ECFP4
V916	Fisher's exact test & t-test	0.01	ECFP4
X34	XGboost	100	viral genome sequence
X65	XGboost	100	viral genome sequence
X55	XGboost	100	viral genome sequence
X54	XGboost	100	viral genome sequence
X81	XGboost	100	viral genome sequence
X20	XGboost	100	viral genome sequence
X13	XGboost	100	viral genome sequence
X6	XGboost	100	viral genome sequence
X79	XGboost	100	viral genome sequence
V243	XGboost	100	ECFP4
X17	XGboost	100	viral genome sequence
X7	XGboost	100	viral genome sequence
X95	XGboost	100	viral genome sequence
X49	XGboost	100	viral genome sequence
X46	XGboost	100	viral genome sequence
X15	XGboost	100	viral genome sequence
X25	XGboost	100	viral genome sequence
V552	XGboost	100	ECFP4
V757	XGboost	100	ECFP4
V438	XGboost	100	ECFP4
X53	XGboost	100	viral genome sequence
V834	XGboost	100	ECFP4
X90	XGboost	100	viral genome sequence
X31	XGboost	100	viral genome sequence
X59	XGboost	100	viral genome sequence
X83	XGboost	100	viral genome sequence
V918	XGboost	100	ECFP4
V784	XGboost	100	ECFP4
X36	XGboost	100	viral genome sequence
X16	XGboost	100	viral genome sequence
V138	XGboost	100	ECFP4
X26	XGboost	100	viral genome sequence
V935	XGboost	100	ECFP4
V854	XGboost	100	ECFP4

V556	XGboost	100	ECFP4
X44	XGboost	100	viral genome sequence
V807	XGboost	100	ECFP4
X27	XGboost	100	viral genome sequence
X2	XGboost	100	viral genome sequence
V893	XGboost	100	ECFP4
V34	XGboost	100	ECFP4
V475	XGboost	100	ECFP4
X8	XGboost	100	viral genome sequence
X45	XGboost	100	viral genome sequence
X77	XGboost	100	viral genome sequence
V988	XGboost	100	ECFP4
X69	XGboost	100	viral genome sequence
V225	XGboost	100	ECFP4
X86	XGboost	100	viral genome sequence
V881	XGboost	100	ECFP4
V853	XGboost	100	ECFP4
V684	XGboost	100	ECFP4
X11	XGboost	100	viral genome sequence
V443	XGboost	100	ECFP4
X4	XGboost	100	viral genome sequence
X93	XGboost	100	viral genome sequence
V1010	XGboost	100	ECFP4
V798	XGboost	100	ECFP4
V277	XGboost	100	ECFP4
X92	XGboost	100	viral genome sequence
V551	XGboost	100	ECFP4
X96	XGboost	100	viral genome sequence
V366	XGboost	100	ECFP4
X41	XGboost	100	viral genome sequence
V623	XGboost	100	ECFP4
X72	XGboost	100	viral genome sequence
X74	XGboost	100	viral genome sequence
X68	XGboost	100	viral genome sequence
X94	XGboost	100	viral genome sequence
V943	XGboost	100	ECFP4
V593	XGboost	100	ECFP4
V331	XGboost	100	ECFP4
X40	XGboost	100	viral genome sequence
X28	XGboost	100	viral genome sequence
V226	XGboost	100	ECFP4
V773	XGboost	100	ECFP4
X33	XGboost	100	viral genome sequence

V275	XGboost	100	ECFP4
V654	XGboost	100	ECFP4
V797	XGboost	100	ECFP4
V999	XGboost	100	ECFP4
V162	XGboost	100	ECFP4
X66	XGboost	100	viral genome sequence
V356	XGboost	100	ECFP4
X67	XGboost	100	viral genome sequence
V657	XGboost	100	ECFP4
X47	XGboost	100	viral genome sequence
V51	XGboost	100	ECFP4
V759	XGboost	100	ECFP4
V587	XGboost	100	ECFP4
V27	XGboost	100	ECFP4
V544	XGboost	100	ECFP4
V471	XGboost	100	ECFP4
V788	XGboost	100	ECFP4
X76	XGboost	100	viral genome sequence
V714	XGboost	100	ECFP4
X52	XGboost	100	viral genome sequence
V253	XGboost	100	ECFP4
V38	XGboost	100	ECFP4
V555	XGboost	100	ECFP4
X34	XGboost	80	viral genome sequence
X65	XGboost	80	viral genome sequence
X55	XGboost	80	viral genome sequence
X54	XGboost	80	viral genome sequence
X81	XGboost	80	viral genome sequence
X20	XGboost	80	viral genome sequence
X13	XGboost	80	viral genome sequence
X6	XGboost	80	viral genome sequence
X79	XGboost	80	viral genome sequence
V243	XGboost	80	ECFP4
X17	XGboost	80	viral genome sequence
X7	XGboost	80	viral genome sequence
X95	XGboost	80	viral genome sequence
X49	XGboost	80	viral genome sequence
X46	XGboost	80	viral genome sequence
X15	XGboost	80	viral genome sequence
X25	XGboost	80	viral genome sequence
V552	XGboost	80	ECFP4
V757	XGboost	80	ECFP4
V438	XGboost	80	ECFP4

X53	XGboost	80	viral genome sequence
V834	XGboost	80	ECFP4
X90	XGboost	80	viral genome sequence
X31	XGboost	80	viral genome sequence
X59	XGboost	80	viral genome sequence
X83	XGboost	80	viral genome sequence
V918	XGboost	80	ECFP4
V784	XGboost	80	ECFP4
X36	XGboost	80	viral genome sequence
X16	XGboost	80	viral genome sequence
V138	XGboost	80	ECFP4
X26	XGboost	80	viral genome sequence
V935	XGboost	80	ECFP4
V854	XGboost	80	ECFP4
V556	XGboost	80	ECFP4
X44	XGboost	80	viral genome sequence
V807	XGboost	80	ECFP4
X27	XGboost	80	viral genome sequence
X2	XGboost	80	viral genome sequence
V893	XGboost	80	ECFP4
V34	XGboost	80	ECFP4
V475	XGboost	80	ECFP4
X8	XGboost	80	viral genome sequence
X45	XGboost	80	viral genome sequence
X77	XGboost	80	viral genome sequence
V988	XGboost	80	ECFP4
X69	XGboost	80	viral genome sequence
V225	XGboost	80	ECFP4
X86	XGboost	80	viral genome sequence
V881	XGboost	80	ECFP4
V853	XGboost	80	ECFP4
V684	XGboost	80	ECFP4
X11	XGboost	80	viral genome sequence
V443	XGboost	80	ECFP4
X4	XGboost	80	viral genome sequence
X93	XGboost	80	viral genome sequence
V1010	XGboost	80	ECFP4
V798	XGboost	80	ECFP4
V277	XGboost	80	ECFP4
X92	XGboost	80	viral genome sequence
V551	XGboost	80	ECFP4
X96	XGboost	80	viral genome sequence
V366	XGboost	80	ECFP4

X41	XGboost	80	viral genome sequence
V623	XGboost	80	ECFP4
X72	XGboost	80	viral genome sequence
X74	XGboost	80	viral genome sequence
X68	XGboost	80	viral genome sequence
X94	XGboost	80	viral genome sequence
V943	XGboost	80	ECFP4
V593	XGboost	80	ECFP4
V331	XGboost	80	ECFP4
X40	XGboost	80	viral genome sequence
X28	XGboost	80	viral genome sequence
V226	XGboost	80	ECFP4
V773	XGboost	80	ECFP4
X33	XGboost	80	viral genome sequence
V275	XGboost	80	ECFP4
V654	XGboost	80	ECFP4
V797	XGboost	80	ECFP4

Table S4. Non-cytotoxic pharmaceutical compounds (NCPCS) in tox21 cell viability assays

SAMPLE_NAME	tox21-ahr-p1-viability	tox21-ap1-agonist-p1-viability	tox21-ar-bla-antagonist-p1-viability	tox21-ar-mda-kb2-luc-agonist-p3-viability	tox21-ar-mda-kb2-luc-antagonist-p1-viability	tox21-ar-mda-kb2-luc-antagonist-p2-viability	tox21-are-bla-p1-viability	tox21-aromatase-p1-viability	tox21-car-agonist-p1-viability	tox21-car-antagonist-p1-viability
Acetazolamide	NA	0	NA	0	NA	0	0	NA	0	0
Chlormezanone	NA	0	NA	0	NA	0	0	NA	0	0
Carbamazepine	NA	0	NA	0	NA	0	0	NA	0	0
Fenclonine	NA	0	NA	0	NA	0	0	NA	0	0
Clonidine	NA	0	NA	0	NA	0	0	NA	0	0
Cyclothiazide	NA	0	NA	0	NA	0	0	NA	0	0
Enoximone	NA	0	NA	0	NA	0	0	NA	0	0
Efaroxan	NA	0	NA	0	NA	0	0	NA	0	0
Felbamate	NA	0	NA	0	NA	0	0	NA	0	0
Flumazenil	NA	0	NA	0	NA	0	0	NA	0	0
Hydroxytacrine maleate	NA	0	NA	0	NA	0	0	NA	0	0
Lamotrigine	NA	0	NA	0	NA	0	0	NA	-0.67	0
Leflunomide	NA	0	NA	0	NA	0	0	NA	0	0
Molsidomine	NA	0	NA	0	NA	0	0	NA	0	0
Minoxidil	NA	0	NA	0	NA	0	0	NA	0	0
Nialamide	NA	0	NA	0	NA	0	0	NA	0	0
Pentoxifylline	NA	0	NA	0	NA	0	0	NA	0	0
Piroxicam	NA	0	NA	0	NA	0	0	NA	0	0
Primidone	NA	0	NA	0	NA	0	0	NA	0	0
Sulfaphenazole	NA	0	NA	0	NA	0	0	NA	0	0
Telenzepine	NA	0	NA	0	NA	0	0	NA	0	0
1-[2-(Trifluoromethyl)phenyl]-1H-imidazole	NA	0	NA	0	NA	0	0	NA	0	0
Tropicamide	NA	0	NA	0	NA	0	0	NA	0	0
Brimonidine	NA	0	NA	0	NA	0	0	NA	0	0
Zardaverine	NA	0	NA	0	NA	0	0	NA	0	0
Furosemide	NA	0	NA	0	NA	0	0	NA	0	0
Metyrapone	NA	0	NA	0	NA	0	0	NA	0	0
Sulfaguanidine	NA	0	NA	0	NA	0	0	NA	0	0
Sulfinpyrazone	NA	0	NA	0	NA	0	0	NA	0	0
Phenacetin	NA	0	NA	0	NA	0	0	NA	0	0
Bendroflumethiazide	NA	0	NA	0	NA	0	0	NA	0	0
Phensuximide	NA	0	NA	0	NA	0	0	NA	0	0

Benzthiazide	NA	0	NA	0	NA	0	0	NA	0	0
Azacyclonol	NA	0	NA	0	NA	0	0	NA	0	0
Sulfadimethoxine	NA	0	NA	0	NA	0	0	NA	0	0
Sulfapyridine	NA	0	NA	0	NA	0	0	NA	0	0
Metronidazole	NA	0	NA	0	NA	0	0	NA	0	0
alpha-Santonin	NA	0	NA	0	NA	0	0	NA	0	0
Tetrahydrozoline hydrochloride	NA	0	NA	0	NA	0	0	NA	0	0
Amylocaine hydrochloride	NA	0	NA	0	NA	0	0	NA	0	0
Trimethobenzamide hydrochloride	NA	0	NA	0	NA	0	0	NA	0	0
Proxyphylline	NA	0	NA	0	NA	0	0	NA	0	0
Clofibric acid	NA	0	NA	0	NA	0	0	NA	0	0
Meticrane	NA	0	NA	0	NA	0	0	NA	0	0
Midodrine hydrochloride	NA	0	NA	0	NA	0	0	NA	0	0
Bromopride	NA	0	NA	0	NA	0	0	NA	0	0
Cyclopentolate hydrochloride	NA	0	NA	0	NA	0	0	NA	0	0
Selegiline hydrochloride	NA	0	NA	0	NA	0	0	NA	0	0
Ornidazole	NA	0	NA	0	NA	0	0	NA	0	0
Tinidazole	NA	0	NA	0	NA	0	0	NA	0	0
Gliclazide	NA	0	NA	0	NA	0	0	NA	0	0
Benfotiamine	NA	0	NA	0	NA	0	0	NA	0	0
Etandazole	NA	0	NA	0	NA	0	0	NA	0	0
Probucol	NA	0	NA	0	NA	0	0	NA	0	0
Suxibuzone	NA	0	NA	0	NA	0	0	NA	0	0
Tiaprofenic acid	NA	0	NA	0	NA	0	0	NA	0	0
Etomidate	NA	0	NA	0	NA	0	0	NA	0	0
Pivampicillin	NA	0	NA	0	NA	0	0	NA	0	0
Acebutolol hydrochloride	NA	0	NA	0	NA	0	0	NA	0	0
Carteolol hydrochloride	NA	0	NA	0	NA	0	0	NA	0	0
Pipemidic acid	NA	0	NA	0	NA	0	0	NA	0	0
Meptazinol hydrochloride	NA	0	NA	0	NA	0	0	NA	0	0
Iopamidol	NA	0	NA	0	NA	0	0	NA	0	0
Rolipram	NA	0	NA	0	NA	0	0.67	NA	0	0
Fluticasone propionate	NA	0	NA	0	NA	0	0	NA	0.67	0
Brinzolamide	NA	0	NA	0	NA	0	0	NA	0	0
Disopyramide	NA	0	NA	0	NA	0	0	NA	0	0

Cefotiam hydrochloride	NA	0	NA	0	NA	0	0	NA	0	0
Indapamide	NA	0	NA	0	NA	0	0	NA	0	0
Metoprolol tartrate	NA	0	NA	0	NA	0	0	NA	0	0
Cefmetazole sodium	NA	-0.67	NA	0	NA	0	0	NA	0	0
Ketorolac	NA	0	NA	0	NA	0	0	NA	0	0
Dropropizine	NA	0	NA	0	NA	0	0	NA	0	0
Enalapril maleate	NA	0	NA	0	NA	0	0	NA	0	0
Hydrocortisone	NA	0	NA	0	NA	0	0	NA	0	0
Methysergide	NA	0	NA	0	NA	0	0	NA	0	0
Flurofamide	NA	0	NA	0	NA	0	0	NA	0	0
Methyldopa	NA	0	NA	0	NA	0	0	NA	0	0
Clomethiazole	NA	0	NA	0	NA	0	0	NA	0	0
Remoxipride	NA	0	NA	0	NA	0	0	NA	0	0
AF-DX 116	NA	0	NA	0	NA	0	0	NA	0	0
Levcromakalim	NA	0	NA	0	NA	0	0	NA	0	0
Roquinimex	NA	0	NA	0	NA	0	0	NA	0	0
Pinacidil monohydrate	NA	0	NA	0	NA	0	0	NA	0	0
Remacemide hydrochloride	NA	0	NA	0	NA	0	0	NA	0	0
Ipsapirone	NA	0	NA	0	NA	0	0	NA	0	0
Nicorandil	NA	0	NA	0	NA	0	0	NA	0	0
Capobenic acid	NA	0	NA	0	NA	0	0	NA	0	0
Sodium zomepirac	NA	0	NA	0	NA	0	0	NA	0	0
4-Chlorophenol	NA	0	NA	0	NA	0	0	NA	0	0
4-Aminobenzoic acid	NA	0	NA	0	NA	0	0	NA	0	0
Diazinon	NA	0	NA	0	NA	0	0	NA	0	0
Aspartame	NA	0	NA	0	NA	0	0	NA	0	0
Phenoxyacetic acid	NA	0	NA	0	NA	0	0	NA	0	0
Anthranilic acid	NA	0	NA	0	NA	0	0	NA	0	0
Hexanamide	NA	0	NA	0	NA	0	0	NA	0	0
Acetanilide	NA	0	NA	0	NA	0	0	NA	0	0
4-Chloro-3-methylphenol	NA	0	NA	0	NA	0	0	NA	0	0
Hexanedioic acid	NA	0	NA	0	NA	0	0	NA	0	0
Allylthiourea	NA	0	NA	0	NA	0	0	NA	0	0
Salicylamide	NA	0	NA	0	NA	0	0	NA	0	0
Fenthion	NA	0	NA	0	NA	0	0	NA	0	0
Cotinine	NA	0	NA	0	NA	0	0	NA	0	0

Fenspiride hydrochloride	NA	0	NA	0	NA	0	0	NA	0	0
Naphazoline hydrochloride	NA	0	NA	0	NA	0	0	NA	0	0
Nomifensine maleate	NA	0	NA	0	NA	0	0	NA	0	0
Bisoprolol fumarate	NA	0	NA	0	NA	0	0	NA	0	0
Diuron	NA	0	NA	0	NA	0	0	NA	0	0
2-Chloro-4-nitrobenzamide	NA	0	NA	0	NA	0	0	NA	0	0
Phenacemide	NA	0	NA	0	NA	0	0	NA	0	0
2-Aminoheptane sulfate	NA	0	NA	0	NA	0	0	NA	0	0
Acetarsol	NA	0	NA	0	NA	0	0	NA	0	0
Iproniazid sulfate	NA	0	NA	0	NA	0	0	NA	0	0
Vincamine	NA	0	NA	0	NA	0	0	NA	0	0
Mephenesin	NA	0	NA	0	NA	0	0	NA	0	0
Saccharin	NA	0	NA	0	NA	0	0	NA	0	0
Acetyl-L-leucine	NA	0	NA	0	NA	0	0	NA	0	0
Chromocarb	NA	0	NA	0	NA	0	0	NA	0	0
Acexamic acid	NA	0	NA	0	NA	0	0	NA	0	0
Pipenzolate bromide	NA	0	NA	0	NA	0	0	NA	0	0
Adiphenine hydrochloride	NA	0	NA	0	NA	0	0	NA	0	0
Diosmin	NA	0	NA	0	NA	0	0	NA	0	0
Pipobroman	NA	0	NA	0	NA	0	0	NA	0	0
Ramifenazone	NA	0	NA	0	NA	0	0	NA	0	0
Metaxalone	NA	0	NA	0	NA	0	0	NA	0	0
Secnidazole	NA	0	NA	0	NA	0	0	NA	0	0
Clopidol	NA	-0.67	NA	0	NA	0	0	NA	0	0
Modafinil	NA	0	NA	0	NA	0	0	NA	0	0
Arbutin	NA	0	NA	0	NA	0	0	NA	0	0
2-Hydroxy-3-methylbenzoic acid	NA	0	NA	0	NA	0	0	NA	0	0
Phenyl 4-aminosalicylate	NA	0	NA	0	NA	0	0	NA	0	0
Salsalate	NA	0	NA	0	NA	0	0	NA	0	0
Folic acid	NA	0	NA	0	NA	0	0	NA	0	0
Clopidogrel bisulfate	NA	0	NA	0	NA	0	0	NA	0	0
Benzathine penicillin G	NA	0	NA	0	NA	0	0	NA	0	0
Tramadol hydrochloride	NA	0	NA	0	NA	0	0	NA	0	0

Huperzine A	NA	0	NA	0	NA	0	0	NA	0	0
N-Acetyl-L-tyrosine	NA	0	NA	0	NA	0	0	NA	0	0
Phenicarbazide	NA	0	NA	0	NA	0	0	NA	0	0
Trimetozine	NA	0	NA	0	NA	0	0	NA	0	0
Iodoantipyrine	NA	0	NA	0	NA	0	0	NA	0	0
Warfarin sodium	NA	0	NA	0	NA	0	0	NA	0	0
Tegafur	NA	0	NA	0	NA	0	0	NA	0	0
Hexobarbital	NA	0	NA	0	NA	0	0	NA	0	0
Benactyzine	NA	0	NA	0	NA	0	0	NA	0	0
Sorbinil	NA	0	NA	0	NA	0	0	NA	0	0
Methyl nicotinate	NA	0	NA	0	NA	0	0	NA	0	0
Hippuric acid	NA	0	NA	0	NA	0	0	NA	0	0
Eszopiclone	NA	0	NA	0	NA	0	0	NA	0	0
Cephalexin	NA	0	NA	0	NA	0	0	NA	0	0
Pepstatin	NA	0	NA	0	NA	0	0	NA	0	0
6,7-Dimethoxyquinazoline-2,4(1H,3H)-dione	NA	0	NA	0	NA	0	0	NA	0	0
Tetridamine	NA	0	NA	0	NA	0	-0.67	NA	0	0
Loretin	NA	0	NA	0	NA	0	0	NA	0	0
Tetrabenazine	NA	0	NA	0	NA	0	0	NA	0	0
4-Acetylaminophenylacetic acid	NA	0	NA	0	NA	0	0	NA	0	0
Lozilurea	NA	0	NA	0	NA	0	0	NA	0	0
Amanozine	NA	0	NA	0	NA	0	0	NA	0	0
Zaleplon	NA	0	NA	0	NA	0	0	NA	0	0
Tizanidine hydrochloride	NA	0	NA	0	NA	0	0	NA	0	0
Romazarit	NA	0	NA	0	NA	0	0	NA	0	0
Diamfenetide	NA	0	NA	0	NA	0	0	NA	0	0
Ethopabate	NA	0	NA	0	NA	0	0	NA	0	0
Sulazepam	0	0	0	0	0.67	0	0	0	0	0
Sulfasuccinamide	NA	0	NA	0	NA	0	0	NA	0	0
Dazmegrel	NA	0	NA	0	NA	0	0	NA	0	0
Ambrisentan	NA	0	NA	0	NA	0	0	NA	0	0
Mecarbinate	NA	0	NA	0	NA	0	0	NA	0	0
Isocaine	NA	0	NA	0	NA	0	0	NA	0	0
Ftaxilide	NA	0	NA	0	NA	0	0	NA	0	0
Naloxone	NA	0	NA	0	NA	0	0	NA	0	0

Ambenonium chloride	NA	0	NA	0	NA	0	-0.67	NA	0	0
Butylscopolamine bromide	NA	0	NA	0	NA	0	0	NA	0	0
Tiotidine	NA	0	NA	0	NA	0	0	NA	0	0
Temephos	NA	0	NA	0	NA	0	0	NA	0	0
Olaquindox	NA	0	NA	0	NA	0	0	NA	0	0
Hexadecanoic acid	NA	0	NA	0	NA	0	0	NA	0	0
Amiphenazole	NA	0	NA	0	NA	0	0	NA	0	0
Nicothiazone	NA	0	NA	0	NA	0	0	NA	0	0
Glyoctamide	NA	0	NA	0	NA	0	0	NA	0	0
Cinromide	NA	0	NA	0	NA	0	0	NA	0	0
Parapropamol	NA	0	NA	0	NA	0	0	NA	0	0
Talinolol	NA	0	NA	0	NA	0	0	NA	0	0
Dofetilide	NA	0	NA	0	NA	0	0	NA	0	0
Eprosartan mesylate	NA	0	NA	0	NA	0	-0.67	NA	0	0
Pargeverine hydrochloride	NA	0	NA	0	NA	0	0	NA	0	0
Capecitabine	NA	0	NA	0	NA	0	0	NA	0	0
Etoricoxib	NA	0	NA	0	NA	0	0	NA	0	0
Cetraxate hydrochloride	NA	0	NA	0	NA	0	0	NA	0	0
Phenprobamate	NA	0	NA	0	NA	0	0	NA	0	0
Propyphenazone	NA	0	NA	0	NA	0	0	NA	0	0
Raltitrexed	0	0	0	0	0	0	0	0	0	0
Voriconazole	NA	0	NA	0	NA	0	0	NA	0	0
Tiagabine hydrochloride	NA	0	NA	0	NA	0	0	NA	0	0
Dexrazoxane hydrochloride	NA	0	NA	0	NA	0	0	NA	0	0
Rufinamide	NA	0	NA	0	NA	0	0	NA	-0.67	0
Iohexol	NA	0	NA	0	NA	0	0	NA	0	0
Flamenol	NA	0	NA	0	NA	0	0	NA	0	0
Ethyl nicotinate	NA	0	NA	0	NA	0	0	NA	0	0
alpha-Lipoic acid amide	NA	0	NA	0	NA	0	0	NA	0	0
Pyrithioxin dihydrochloride	NA	0	NA	0	NA	0	0	NA	0	0
Alovudine	NA	0	NA	0	NA	0	0	NA	0	0
Carsalam	NA	0	NA	0	NA	0	0	NA	0	0
Temozolomide	NA	0	NA	0	NA	0	0	NA	0	0
Pemirolast potassium	NA	0	NA	0	NA	0	0	NA	0	0
Trospium chloride	NA	0	NA	0	NA	0	0	NA	0	0

Eprazinone dihydrochloride	NA	0	NA	0	NA	0	0.67	NA	0	0
Clinofibrate	NA	0	NA	0	NA	0	0	NA	0	0
Zofenopril	NA	0	NA	0	NA	0	0	NA	0	0
Vecuronium bromide	NA	0	NA	0	NA	0	0	NA	0	0
Lenalidomide	NA	-0.67	NA	0	NA	0	0	NA	0	0
Pipethanate ethylbromide	NA	0	NA	0	NA	0	0	NA	0	0
Pemoline	NA	0	NA	0	NA	0	0	NA	0	0
Ipidacrine hydrochloride hydrate	NA	0	NA	0	NA	0	0	NA	0	0
Lazabemide hydrochloride	NA	0	NA	0	NA	0	0	NA	0	0
Loxoribine	NA	0	NA	0	NA	0	0	NA	0	0
Desonide	NA	0	NA	0	NA	0	0	NA	0	0
Fenobam	NA	0	NA	0	NA	0	0	NA	0	0
Suplatast tosylate	NA	0	NA	0	NA	0	0	NA	0	0
Timepidium bromide	NA	0	NA	-0.67	NA	0	0	NA	0	0
Nitarsone	NA	0	NA	0	NA	0	0	NA	0	0
Mebrofenin	0	0	0	0	0	0	0	0	0	0
Acetyl tributyl citrate	0	0	0	0	0	0	0	0	0	0
Imidapril hydrochloride	NA	0	NA	0	NA	0	0	NA	0	0
(-)- (S)-Cathinone hydrochloride	0	0	0	0	0	0	0	0	0	0
(4R,5S)-4-methyl-5-phenyl-4,5-dihydro-1,3-oxazol-2-amine	0	0	0	0	0	0	0	0	0	0
Mescaline hydrochloride	0	0	0	0	0	0	0	0	0	0
3,4,5-Trimethoxyamphetamine hydrochloride	0	0	0	0	-0.67	0	0	0	0	0
(+)-Metazocine fumarate (2:1)	0	0	0	0	0	0	0	0	0	0
1-Methyl-4-phenyl-4-propionoxypiperidine hydrochloride	0	0	0	0	0	0	0	0	0	0
N-(4-Fluorophenyl)-N-[1-(2-	-0.67	0	0	0	0	0	0	0	0	0

phenylethyl)-4-piperidyl] propanamide										
N-(1-Benzyl-4-piperidyl)-N-phenylpropanamide hydrochloride	0	0	0	0	0	0	0	0	0	0
1-(7-Methoxy-1,3-benzodioxol-5-yl)propan-2-amine hydrochloride	0	0	0	0	0	0	0	0	0	0
N-[1-(2-Hydroxy-2-phenylethyl)-4-piperidyl]-N-phenylpropanamide hydrochloride	0	0	0	0	0	0	0	0	-0.67	0
N-Phenyl-N-[1-(2-thienyl)methyl-4-piperidyl]propanamide hydrochloride	0	0	0	0	0	0	0	0	0	0
N-Phenyl-N-[1-[2-(2-thienyl)ethyl]-4-piperidyl]propanamide hydrochloride (1:1)	0	0	0	-1.33	-0.67	0	0	-0.67	-0.67	-1.33
R-(-)-2,5-Dimethoxy-4-methylamphetamine hydrochloride	0	0	0	0	-0.67	0	0	0	0	0
2,5-Dimethoxyamphetamine hydrochloride	0	0	-0.67	0	-0.67	0	0	0	0	0
1-(1,3-Benzodioxol-5-yl)-N-hydroxypropan-2-amine hydrochloride	0	0	0	0	0	0	-0.67	0	0	0
(S)-(+)-N-Ethylamphetamine hydrochloride	0	0	0	0	0	0	0	0	0	0
(-)-Fenfluramine hydrochloride	-0.67	0	0	0	0	0	0	0	0	-0.67
Amobarbital	0	0	0	0	0	0	1	0	0	0

S-(-)-Secobarbital	0	0	0	0	0	0	0	0	0	-0.67
Alfentanil hydrochloride	0	0	0	0	0	0	0	-1.33	0	0
1-Phenylcyclohexylamine hydrochloride	-0.67	0	0	0	0	0	0	-0.67	-2.67	-4
1-Benzylpiperazine difumarate	0	0	0	0	0	0	0	0	0	0
Dihydroisocodeine	0	0	0	0	0	0	0	0	0	0
Dihydromorphine	0	0	0	0	0	0	0	0	0	-0.67
1-(1,3-Benzodioxol-5-yl)-N-ethylpropan-2-amine hydrochloride	0	0	0	0	0	0	0	0	0	0
1-(1,3-Benzodioxol-5-yl)-N-methylpropan-2-amine hydrochloride	0	0	0	0	0	0	0	0	0	-1.33
Fenethylline hydrochloride	-4	0	0	0	0	0	0	-3.33	-0.67	-1.33
alpha-Methylfentanyl hydrochloride	0	0	0	0	0	0	0	0	0	0
Varenicline tartrate	NA	0	NA	0	NA	0	0	NA	0	0
Acifluorfen-sodium	0.67	0	0	0	0	0	0	0	0	0
Alacepril	0	0	0	0	0	0	0	0	0	0
Cefetamet pivoxil HCl	0	0	0	0	0	0	0	0	0	0
Cefozopran hydrochloride	0	0	0	0	0	0	0	0	0	0
Etizolam	0	0	0	0	0	0	0	0	0	0
Lobenzarit sodium	0	-0.67	0	0	0	0	0	0	0	0
Lysozyme hydrochloride	0	0	0	0	0	0	0	0	0	0
Sulbenicillin disodium	0	0	0	0	0	0	0	0	0	0
Xipamide	NA	0	NA	0	NA	0	0	NA	0	0
Tirofiban hydrochloride monohydrate	NA	0	NA	0	NA	0	0	NA	0	0
Landiolol hydrochloride	NA	0	NA	0	NA	0	0	NA	0	0

Biotin	NA	-0.67	NA	0	NA	0	0	NA	0	0
Propamidine	-4.33	0	0	0	0	0	0	0	-3	0
Cythioate	0.67	0	0	0	0	0	0	0	0	0
Nifenalol	NA	0	NA	0	NA	0	0	NA	0	0
Sulfaethoxypyridazine	0	0	0	0	0	0	0	0	0	0
Triamcinolone diacetate	NA	0	NA	0	NA	0	0	NA	0	0
Fexofenadine hydrochloride	NA	0	NA	0	NA	0	0	NA	0	0
Niceritrol	NA	0	NA	0	NA	0	0	NA	0	0
Ioxilan	NA	0	NA	0	NA	0	0.67	NA	0	0
Bamifylline Hydrochloride	NA	0	NA	0	NA	0	0	NA	0	0
Cadralazine	NA	0	NA	0	NA	0	0	NA	0	0
Oberadilol	0	0	0	0	0	0	0	0	0	0
Iomazenil	0	0	0	0	0	0	0	0	0	0
Depolipon	0	0	0	0	0	0	0	0	0	0
Phenyl 11-iodo-10-undecynoate	0	0	0	0	0	0	0	0	0	0
Tolvaptan	-1.33	0	0	0	0	0	0	0	0	0
Anacolin	0	0	0	0	0	0	0	0	0	0
Bimosiamose	0	0	-0.67	0	0	0	0	0	0	0
Betiatide	0	0	0	0	0	0	0	0	0	0
Acetylpheneturide	0	0	0	0	0	0	0	0	0	0
Blonanserin	0	0	0	0	0	0	0	0	0	0
Dicethiamine hydrochloride	0	0	0	0	0	0	0	0	0	0
Cloforex	0	0	0	0	0	0	0	0	0	0
Pelubiprofen	0	0	0	0	0	0	0	0	0	0
Risarestat	0	-0.67	-0.67	0	0	0	0	0	0	0
Diponium bromide	0	0	0	-3.67	-0.67	-1.33	0	0	0	0
Emiglitate	0	0	0	0	0	0	0	0	0	0
Etomidoline	0	0	0	0	0	0	0	0	0	0
Fezatione	0	0	-0.67	0	0	0	0	0	0	0
Itasetron	0	0	0	-2.67	-0.67	0	0	0	0	0
Pamicogrel	0	0	0	0	0	0	0	0	-0.67	-1.33
Esonarimod	0.67	0	0	0	0	0	0	0	0	0
Dalcotidine	0	0	0	0	0	0	0	0	0	0
Melinamide	0	-0.67	0	0	0	0	0	0	0	0
Meluadrine	0	0	0	0	0	0	0	-0.67	0	0
Oxendolone	0	0	0	0	0	0	0	0	0	0

Penflutizide	0	0	0	0	0	0	0	0	0	0
Doranidazole	0.67	-0.67	0	0	0	0	0	0	0	0
Prifinium bromide	0	-0.67	0	-2	-0.67	0	0	0	0	0
Sulfamethomidine	0	0	0	0	0	0	0	0	0	0
Tolindate	-0.67	0	-0.67	0	-0.67	0	0	0	0	0
Acreozast	0	0	0	0	0	0	0	0	0	0
Cefteram	0	0	0	0	0	0	0	0	0	0
Glyclopyramide	0	0	0	0	0	0	0	0	0	0
Rapacuronium bromide	0	0	0	0	0	0	-0.67	0	0	0
Temafloxacin	0	0	-0.67	0	0	0	0	0	0	0
Propanidid	0	0	0	0	0	0	0	0	0	0
Nitrefazole	0	0	0	0	0	0	0	0	0	0
Clometacine	0	0	0	0	0	0	0	0	0	0
Pifoxime	0	0	-1.33	0	0	0	0	0	0	0
4'-Piperidinylcarbonylmethoxyacetophenone	0	0	0	0	0	0	0	0	0	0
Fenclofenac	0.67	0	0	0	0	0	0	0	0	0
Amineptine	0	0	0	0	0	0	0	0	0	0
Flosequinan	0	0	0	0	0	0	0	0	0	0
Tolrestat	0	0	0	0	0	0	0	0.67	0	0
Tandospirone	0	0	0	0	0	0	0	0	0	0
Phenoxypropazine	0	0	0	0	0	0	0	0	0	0
Amoproxan	0	0	-1	0	0	0	0	0	0	0
Glybuzole	0	0	0	0	0	0	0	0	0	0
Safrazine hydrochloride	0	0	0	0	0	0	0	0	0	0
Ethyl 4-nitrophenyl ethylphosphonate	0	0	0	-4	-0.67	0	-0.67	0	0	0
Mefruside	0	0	0	0	0	0	0	0	0	0
Flomoxef	0	0	0	0	0	0	0	0	0	0
Sitafloxacin hydrate	0	0	0	0	0	0	0	0	0	0
Aceglatone	0	0	0	0	0	0	0	0	0	0
Sulcaine	0	0	0	0	0	0	0	0	0	0
N,N-Dimethyl-2-(2-phenylacetamido)acetamide	0	0	-0.67	0	0	0	0	0	0	0

Mepranoprofen arbamel	0	0	0	0	0	0	0	-1	0	0
Alloclamide	-1.33	0	0	0	0	0	0	-1.33	0	0
Bucolome	0	0	0	0	0	0	0	0	0	0
Fasoracetam	0	0	0	0	0	0	0	0	0	0
Lanperisone hydrochloride	0	0	0	0	0	0	0	0	0	-0.67
5alpha-Androstan-3beta-ol	0.67	0	0	0	0	0	0	0	0	0
Diloxanide furoate	NA	0	NA	0	NA	0	0	NA	0	0
cAMP	NA	0	NA	0	NA	0	0	NA	0	0
4-Hydroxy-3-methoxybenzaldehyde	NA	0	NA	0	NA	0	0	NA	0	0
Todralazine hydrochloride	NA	0	NA	0	NA	0	0	NA	0	0
N-Acetyl sulfamethoxazole	NA	0	NA	0	NA	0	0	NA	0	0
Methsuximide	0	0	0	0	0	0	0	0	0	0
Zaltoprofen	NA	0	NA	0	NA	0	0	NA	0	0
Mebutamate	NA	0	NA	0	NA	0	0	NA	0	0
Sodium 3, 4-dimethylphenyl-glyoxylate	NA	0	NA	0	NA	0	0	NA	0	0
Cloxazolam	NA	0	NA	0	NA	0	0	NA	0	0
Docarpamine	NA	0	NA	0	NA	0	0	NA	0	0
Emorfazone	NA	0	NA	0	NA	0	0	NA	0	0
Glymidine sodium	NA	0	NA	0	NA	0	0	NA	0	0
Talbutal	NA	0	NA	0	NA	0	0	NA	0	0
Embutramide	NA	0	NA	0	NA	0	0	NA	0	0
Arimoclomol	NA	0	NA	0	NA	0	0	NA	0	0
Sulfacytine	NA	0	NA	0	NA	0	0	NA	0	0
Thenyldiamine	NA	0	NA	0	NA	0	0	NA	0	0
Isoaminile	NA	0	NA	0	NA	0	0	NA	0	0
Methyl 5-sulfamoyl-o-anisate	NA	0	NA	0	NA	0	0	NA	0	0
Darusentan	NA	0	NA	0	NA	0	0	NA	0	0
1H-Tetrazol-1-acetic acid	NA	0	NA	0	NA	0	0	NA	0	0
Acephylline	NA	0	NA	0	NA	0	0	NA	0	0
3,5-Di-tert-butyl-4-	NA	0	NA	0	NA	0	0	NA	0	0

hydroxybenzaldehyde										
6-Methoxy-2-naphthalaldehyde	NA	0	NA	0	NA	0	0	NA	0	0
Brofoxine	NA	0	NA	0	NA	0	0	NA	0	0
5,6-Dimethoxy-1-indanone	NA	0	NA	0	NA	0	0	NA	0	0
Dibenzosuberone	NA	0	NA	0	NA	0	0	NA	0	0
3,4,5-Trimethoxyphenylacetonitrile	NA	-0.67	NA	0	NA	0	0	NA	0	0
Cintriamide	NA	0	NA	0	NA	0	0	NA	0	0
4-(4-Acetylpiperazin-4-yl)phenol	NA	0	NA	0	NA	0	0	NA	0	0
5-Acetylsalicylamide	NA	0	NA	0	NA	0	0	NA	0	0
Dimecrotic acid	NA	0	NA	0	NA	0	0	NA	0	0
3,3-Tetramethyleneglutarimide	NA	0	NA	0	NA	0	0	NA	0	0
Odiparcil	NA	0	NA	0	NA	0	0	NA	0	0
Tizoprolic acid	NA	0	NA	0	NA	0	0	NA	0	0
Methyl 5-(ethylsulfonyl)-o-anisate	NA	0	NA	0	NA	0	0	NA	0	0
Sulfaperin	NA	0	NA	0	NA	0	0	NA	0	0
(2Z)-{2-[(Chloroacetyl)amino]-1,3-thiazol-4-yl}(methoxyimino)acetic acid	NA	0	NA	0	NA	0	0	NA	0	0
Enciprazine	NA	0	NA	0	NA	0	0	NA	0	0
Propoxate	NA	0	NA	0	NA	0	0	NA	0	0
Prucalopride	NA	0	NA	0	NA	0	0	NA	0	0
Cycotiamine	NA	0	NA	0	NA	0	0	NA	0	0
Norfentanyl	NA	0	NA	0	NA	0	0	NA	0	0
Bravavir	NA	0	NA	0	NA	0	0	NA	0	0
Viloxazine hydrochloride	NA	0	NA	0	NA	0	0	NA	0	0
Furfenorex	NA	0	NA	0	NA	0	0	NA	0	0
Lotrafiban	NA	0	NA	0	NA	0	0	NA	0	0

SAMPLE_NAME	tox21-casp3-cho-p1-viability	tox21-casp3-hepg2-p1-viability	tox21-dt40-p1-100	tox21-dt40-p1-653	tox21-dt40-p1-657	tox21-elg1-luc-agonist-p1-viability	tox21-er-bla-antagonist-p1-viability	tox21-er-luc-bg1-4e2-agonist-p4-viability	tox21-er-luc-bg1-4e2-antagonist-p1-viability	tox21-er-luc-bg1-4e2-antagonist-p2-viability
Acetazolamide	0	0	NA	NA	NA	NA	NA	0	NA	0
Chlormezanone	0.67	0	NA	NA	NA	NA	NA	0	NA	0
Carbamazepine	0	0	NA	NA	NA	NA	NA	0	NA	0
Fenclonine	0	0	NA	NA	NA	NA	NA	0	NA	0
Clonidine	0	0	NA	NA	NA	NA	NA	0	NA	0
Cyclothiazide	0	0	NA	NA	NA	NA	NA	0	NA	0
Enoximone	0	0	NA	NA	NA	NA	NA	0	NA	0
Efaroxan	0	0	NA	NA	NA	NA	NA	0	NA	0
Felbamate	0	0	NA	NA	NA	NA	NA	0	NA	0
Flumazenil	0	0	NA	NA	NA	NA	NA	0	NA	0
Hydroxytacrine maleate	0	0	NA	NA	NA	NA	NA	0	NA	0
Lamotrigine	0	0	NA	NA	NA	NA	NA	0	NA	0
Leflunomide	0	0	NA	NA	NA	NA	NA	-0.67	NA	0
Molsidomine	0	0	NA	NA	NA	NA	NA	0	NA	0
Minoxidil	0	0	NA	NA	NA	NA	NA	0	NA	0
Nialamide	0.67	0	NA	NA	NA	NA	NA	0	NA	0
Pentoxifylline	0	0	NA	NA	NA	NA	NA	0	NA	0
Piroxicam	0	0	NA	NA	NA	NA	NA	0	NA	0
Primidone	0	0	NA	NA	NA	NA	NA	-0.67	NA	0
Sulfaphenazole	0	0	NA	NA	NA	NA	NA	0	NA	0
Telenzepine	0	0	NA	NA	NA	NA	NA	0	NA	0
1-[2-(Trifluoromethyl)phenyl]-1H-imidazole	0	0	NA	NA	NA	NA	NA	0	NA	0
Tropicamide	0	0	NA	NA	NA	NA	NA	0	NA	0
Brimonidine	0	0	NA	NA	NA	NA	NA	0	NA	0
Zardaverine	0	0	NA	NA	NA	NA	NA	0	NA	0
Furosemide	0	0	NA	NA	NA	NA	NA	0	NA	0
Metyrapone	0	0	NA	NA	NA	NA	NA	0	NA	0
Sulfaguanidine	0	0	NA	NA	NA	NA	NA	0	NA	0
Sulfinpyrazone	0	0	NA	NA	NA	NA	NA	0	NA	0
Phenacetin	0	0	NA	NA	NA	NA	NA	0	NA	0
Bendroflumethiazide	0	0	NA	NA	NA	NA	NA	0	NA	0
Phensuximide	0	0	NA	NA	NA	NA	NA	0	NA	0
Benzthiazide	0.67	0	NA	NA	NA	NA	NA	0	NA	0

Azacyclonol	0	0	NA	NA	NA	NA	NA	0	NA	0
Sulfadimethoxine	0.67	0	NA	NA	NA	NA	NA	0	NA	0
Sulfapyridine	0	0	NA	NA	NA	NA	NA	0	NA	0
Metronidazole	0.67	0	NA	NA	NA	NA	NA	0	NA	0
alpha-Santonin	-0.67	0	NA	NA	NA	NA	NA	0	NA	0
Tetrahydrozoline hydrochloride	0	0	NA	NA	NA	NA	NA	0	NA	0
Amylocaine hydrochloride	0	0	NA	NA	NA	NA	NA	0	NA	0
Trimethobenzamide hydrochloride	0	0	NA	NA	NA	NA	NA	0	NA	0
Proxyphylline	0	-0.67	NA	NA	NA	NA	NA	0	NA	0
Clofibric acid	0	0	NA	NA	NA	NA	NA	0	NA	0
Meticrane	0	0	NA	NA	NA	NA	NA	0	NA	0
Midodrine hydrochloride	0	0	NA	NA	NA	NA	NA	-0.67	NA	0
Bromopride	0	0	NA	NA	NA	NA	NA	0	NA	0
Cyclopentolate hydrochloride	0	0	NA	NA	NA	NA	NA	0	NA	0
Selegiline hydrochloride	0	0	NA	NA	NA	NA	NA	0	NA	0
Ornidazole	0	0	NA	NA	NA	NA	NA	0	NA	0
Tinidazole	0	0	NA	NA	NA	NA	NA	0	NA	0
Gliclazide	0	0	NA	NA	NA	NA	NA	0	NA	0
Benfotiamine	0	0	NA	NA	NA	NA	NA	0	NA	0
Etandazole	0	0	NA	NA	NA	NA	NA	0	NA	0
Probucol	0	0	NA	NA	NA	NA	NA	0	NA	0
Suxibuzone	0	0	NA	NA	NA	NA	NA	0	NA	0
Tiaprofenic acid	0	0	NA	NA	NA	NA	NA	0	NA	0
Etomidate	0	0	NA	NA	NA	NA	NA	0	NA	0
Pivampicillin	0	0	NA	NA	NA	NA	NA	0	NA	0
Acebutolol hydrochloride	0	0	NA	NA	NA	NA	NA	0	NA	0
Carteolol hydrochloride	0	0	NA	NA	NA	NA	NA	0	NA	0
Pipemidic acid	0	0.67	NA	NA	NA	NA	NA	0	NA	0
Meptazinol hydrochloride	0	0	NA	NA	NA	NA	NA	0	NA	0
Iopamidol	0.67	0	NA	NA	NA	NA	NA	0	NA	0
Rolipram	0	0	NA	NA	NA	NA	NA	0	NA	0
Fluticasone propionate	0	0	NA	NA	NA	NA	NA	0	NA	0
Brinzolamide	0	0	NA	NA	NA	NA	NA	0	NA	0
Disopyramide	0	0	NA	NA	NA	NA	NA	0	NA	0
Cefotiam hydrochloride	0	0	NA	NA	NA	NA	NA	0	NA	0
Indapamide	0	0	NA	NA	NA	NA	NA	0	NA	0
Metoprolol tartrate	0	0	NA	NA	NA	NA	NA	0	NA	0
Cefmetazole sodium	0	0	NA	NA	NA	NA	NA	0	NA	0
Ketorolac	0	0	NA	NA	NA	NA	NA	0	NA	0
Dropropizine	0	0	NA	NA	NA	NA	NA	0	NA	0

Enalapril maleate	0	0	NA	NA	NA	NA	NA	0	NA	0
Hydrocortisone	0.67	0	NA	NA	NA	NA	NA	0	NA	0
Methysergide	0	0	NA	NA	NA	NA	NA	0	NA	0
Flurofamide	0	0	NA	NA	NA	NA	NA	0	NA	0
Methyldopa	0	0	NA	NA	NA	NA	NA	0	NA	0
Clomethiazole	0	0	NA	NA	NA	NA	NA	0	NA	0
Remoxipride	0	0	NA	NA	NA	NA	NA	0	NA	0
AF-DX 116	0	0	NA	NA	NA	NA	NA	0	NA	0
Levcromakalim	0.67	0	NA	NA	NA	NA	NA	0	NA	0
Roquinimex	0	0	NA	NA	NA	NA	NA	0	NA	0
Pinacidil monohydrate	-0.67	0	NA	NA	NA	NA	NA	0	NA	0
Remacemide hydrochloride	0	0	NA	NA	NA	NA	NA	0	NA	0
Ipsapirone	0	0	NA	NA	NA	NA	NA	0	NA	0
Nicorandil	0	0	NA	NA	NA	NA	NA	0	NA	0
Capobenic acid	0	0	NA	NA	NA	NA	NA	0	NA	0
Sodium zomepirac	0	0	NA	NA	NA	NA	NA	0	NA	0
4-Chlorophenol	0	0	NA	NA	NA	NA	NA	0	NA	0
4-Aminobenzoic acid	0	0	NA	NA	NA	NA	NA	0	NA	0
Diazinon	0.67	0	NA	NA	NA	NA	NA	0	NA	0
Aspartame	0	0	NA	NA	NA	NA	NA	0	NA	0
Phenoxyacetic acid	0	0.67	NA	NA	NA	NA	NA	0	NA	0
Anthranilic acid	0	0	NA	NA	NA	NA	NA	0	NA	0
Hexanamide	-0.67	0	NA	NA	NA	NA	NA	0	NA	0
Acetanilide	0.67	0	NA	NA	NA	NA	NA	0	NA	0
4-Chloro-3-methylphenol	0	0	NA	NA	NA	NA	NA	0	NA	0
Hexanedioic acid	0	0	NA	NA	NA	NA	NA	0	NA	0
Allylthiourea	0	0	NA	NA	NA	NA	NA	0	NA	0
Salicylamide	0	0	NA	NA	NA	NA	NA	0	NA	0
Fenthion	0	0	NA	NA	NA	NA	NA	0	NA	0
Cotinine	0	0	NA	NA	NA	NA	NA	0	NA	0
Fenspiride hydrochloride	0	0	NA	NA	NA	NA	NA	0	NA	0
Naphazoline hydrochloride	0	0	NA	NA	NA	NA	NA	0	NA	0
Nomifensine maleate	0	0	NA	NA	NA	NA	NA	0	NA	0
Bisoprolol fumarate	0.67	0	NA	NA	NA	NA	NA	0	NA	0
Diuron	0	0	NA	NA	NA	NA	NA	0	NA	0
2-Chloro-4-nitrobenzamide	0	0	NA	NA	NA	NA	NA	0	NA	0
Phenacemide	0.67	0	NA	NA	NA	NA	NA	0	NA	0
2-Aminoheptane sulfate	0	0	NA	NA	NA	NA	NA	0	NA	0
Acetarsol	0	0	NA	NA	NA	NA	NA	0	NA	0
Iproniazid sulfate	0	0	NA	NA	NA	NA	NA	0	NA	0
Vincamine	0	0	NA	NA	NA	NA	NA	0	NA	0

Mephenesin	0	0	NA	NA	NA	NA	NA	0	NA	0
Saccharin	0.67	0	NA	NA	NA	NA	NA	0	NA	0
Acetyl-L-leucine	0	0	NA	NA	NA	NA	NA	0	NA	0
Chromocarb	0	0	NA	NA	NA	NA	NA	0	NA	0
Acexamic acid	0	0	NA	NA	NA	NA	NA	0	NA	0
Pipenzolate bromide	0	0	NA	NA	NA	NA	NA	0	NA	0
Adiphenine hydrochloride	0	0	NA	NA	NA	NA	NA	0	NA	0
Diosmin	0	0	NA	NA	NA	NA	NA	0	NA	0
Pipobroman	0	0	NA	NA	NA	NA	NA	0	NA	0
Ramifenazone	0	0	NA	NA	NA	NA	NA	0	NA	0
Metaxalone	0	0	NA	NA	NA	NA	NA	0	NA	0
Secnidazole	0	0	NA	NA	NA	NA	NA	0	NA	0
Clopidol	0.67	0	NA	NA	NA	NA	NA	0	NA	0
Modafinil	0.67	0	NA	NA	NA	NA	NA	0	NA	0
Arbutin	0.67	0	NA	NA	NA	NA	NA	0	NA	0
2-Hydroxy-3-methylbenzoic acid	0	0	NA	NA	NA	NA	NA	0	NA	0
Phenyl 4-aminosalicylate	0	0	NA	NA	NA	NA	NA	0	NA	0
Salsalate	0	0	NA	NA	NA	NA	NA	0	NA	0
Folic acid	0	0	NA	NA	NA	NA	NA	0	NA	0
Clopidogrel bisulfate	0	0	NA	NA	NA	NA	NA	0	NA	0
Benzathine penicillin G	0.67	0	NA	NA	NA	NA	NA	0	NA	0
Tramadol hydrochloride	0	0	NA	NA	NA	NA	NA	0	NA	0
Huperzine A	0	0	NA	NA	NA	NA	NA	0	NA	0
N-Acetyl-L-tyrosine	0	0	NA	NA	NA	NA	NA	0	NA	0
Phenicarbazide	0	0	NA	NA	NA	NA	NA	0	NA	0
Trimetozine	0.67	0	NA	NA	NA	NA	NA	0	NA	0
Iodoantipyrine	0	0	NA	NA	NA	NA	NA	0	NA	0
Warfarin sodium	0	0	NA	NA	NA	NA	NA	0	NA	0
Tegafur	0	0	NA	NA	NA	NA	NA	0	NA	0
Hexobarbital	0	0	NA	NA	NA	NA	NA	0	NA	0
Benactyzine	0	0	NA	NA	NA	NA	NA	0	NA	0
Sorbinil	0	0	NA	NA	NA	NA	NA	0	NA	0
Methyl nicotinate	0	0	NA	NA	NA	NA	NA	0	NA	0
Hippuric acid	0	0	NA	NA	NA	NA	NA	0	NA	0
Eszopiclone	0	0	NA	NA	NA	NA	NA	0	NA	0
Cephalexin	0	0	NA	NA	NA	NA	NA	0	NA	0
Pepstatin	0	0	NA	NA	NA	NA	NA	0	NA	0
6,7-Dimethoxyquinazoline-2,4(1H,3H)-dione	0.67	0	NA	NA	NA	NA	NA	0	NA	0
Tetridamine	0	0	NA	NA	NA	NA	NA	0	NA	0
Loretin	0	0	NA	NA	NA	NA	NA	0	NA	0

Tetrabenazine	0	0	NA	NA	NA	NA	NA	0	NA	0
4-Acetylaminophenylacetic acid	0	0	NA	NA	NA	NA	NA	0	NA	0
Lozilurea	0	0	NA	NA	NA	NA	NA	0	NA	0
Amanozine	0	0	NA	NA	NA	NA	NA	0	NA	0
Zaleplon	0	0	NA	NA	NA	NA	NA	0	NA	0
Tizanidine hydrochloride	0	0	NA	NA	NA	NA	NA	0	NA	0
Romazarit	0	0	NA	NA	NA	NA	NA	0	NA	0
Diamfenetide	0	0	NA	NA	NA	NA	NA	0	NA	0
Ethopabate	0	0	NA	NA	NA	NA	NA	0	NA	0
Sulazepam	0	0	-7	-5.67	-2	0	0	0	0	0
Sulfasuccinamide	0	0	NA	NA	NA	NA	NA	0	NA	0
Dazmegrel	0	0	NA	NA	NA	NA	NA	0	NA	0
Ambrisentan	0	0	NA	NA	NA	NA	NA	0	NA	0
Mecarbinate	0	0	NA	NA	NA	NA	NA	0	NA	0
Isocaine	0	0	NA	NA	NA	NA	NA	0	NA	0
Ftaxilide	0.67	0	NA	NA	NA	NA	NA	0	NA	0
Naloxone	0	0	NA	NA	NA	NA	NA	0	NA	0
Ambenonium chloride	0	0	NA	NA	NA	NA	NA	0	NA	0
Butylscopolamine bromide	0.67	0	NA	NA	NA	NA	NA	0	NA	0
Tiotidine	0	0	NA	NA	NA	NA	NA	0	NA	0
Temephos	0	0	NA	NA	NA	NA	NA	0	NA	0
Olaquindox	0	0	NA	NA	NA	NA	NA	0	NA	0
Hexadecanoic acid	-0.67	0	NA	NA	NA	NA	NA	0	NA	0
Amiphenazole	0.67	0	NA	NA	NA	NA	NA	0	NA	0
Nicothiazone	0	0	NA	NA	NA	NA	NA	0	NA	0
Glyoctamide	0.67	0	NA	NA	NA	NA	NA	0	NA	0
Cinromide	0	0	NA	NA	NA	NA	NA	0	NA	0
Parapropamol	0	0	NA	NA	NA	NA	NA	0	NA	0
Talinolol	0	0	NA	NA	NA	NA	NA	0	NA	0
Dofetilide	0.67	0	NA	NA	NA	NA	NA	0	NA	0
Eprosartan mesylate	0	0	NA	NA	NA	NA	NA	0	NA	0
Pargeverine hydrochloride	-0.67	0	NA	NA	NA	NA	NA	0	NA	0
Capecitabine	0	0	NA	NA	NA	NA	NA	0	NA	0
Etoricoxib	0	0	NA	NA	NA	NA	NA	0	NA	0
Cetraxate hydrochloride	0	0	NA	NA	NA	NA	NA	0	NA	0
Phenprobamate	0	0	NA	NA	NA	NA	NA	0	NA	0
Propyphenazone	0	0	NA	NA	NA	NA	NA	0	NA	0
Raltitrexed	0	0	-9	-9	-9	0	-7	0	0	0
Voriconazole	0	0	NA	NA	NA	NA	NA	0	NA	0
Tiagabine hydrochloride	0.67	0	NA	NA	NA	NA	NA	0	NA	0

Dexrazoxane hydrochloride	0	0	NA	NA	NA	NA	NA	0	NA	0
Rufinamide	0	0	NA	NA	NA	NA	NA	0	NA	0
Iohexol	0	0	NA	NA	NA	NA	NA	0	NA	0
Flamenol	0	0	NA	NA	NA	NA	NA	0	NA	0
Ethyl nicotinate	0	0	NA	NA	NA	NA	NA	0	NA	0
alpha-Lipoic acid amide	0.67	0.67	NA	NA	NA	NA	NA	0	NA	0
Pyrithioxin dihydrochloride	0	0	NA	NA	NA	NA	NA	0	NA	0
Alovudine	0	0	NA	NA	NA	NA	NA	0	NA	0
Carsalam	0.67	0	NA	NA	NA	NA	NA	0	NA	0
Temozolomide	0	0	NA	NA	NA	NA	NA	0	NA	0
Pemirolast potassium	0	0	NA	NA	NA	NA	NA	0	NA	0
Trospium chloride	0	0	NA	NA	NA	NA	NA	0	NA	0
Eprazinone dihydrochloride	0	0	NA	NA	NA	NA	NA	0	NA	0
Clinofibrate	0	0	NA	NA	NA	NA	NA	0	NA	0
Zofenopril	0	0	NA	NA	NA	NA	NA	0	NA	0
Vecuronium bromide	0	0	NA	NA	NA	NA	NA	0	NA	0
Lenalidomide	0	0	NA	NA	NA	NA	NA	0	NA	0
Pipethanate ethylbromide	0	0	NA	NA	NA	NA	NA	0	NA	0
Pemoline	0	0	NA	NA	NA	NA	NA	0	NA	0
Ipidacrine hydrochloride hydrate	0	0	NA	NA	NA	NA	NA	0	NA	0
Lazabemide hydrochloride	0	0	NA	NA	NA	NA	NA	0	NA	0
Loxoribine	0	0	NA	NA	NA	NA	NA	0	NA	0
Desonide	0	0	NA	NA	NA	NA	NA	0	NA	0
Fenobam	0	0	NA	NA	NA	NA	NA	0	NA	0
Suplatast tosylate	0	0	NA	NA	NA	NA	NA	0	NA	0
Timepidium bromide	0.67	0	NA	NA	NA	NA	NA	0	NA	0
Nitarsone	0	0	NA	NA	NA	NA	NA	0	NA	0
Mebrofenin	0	0	0	0.67	0.67	0	0	0	0	0
Acetyl tributyl citrate	0	0	-3.67	-5.33	-1.67	0.67	0	0	0	0
Imidapril hydrochloride	0	0	NA	NA	NA	NA	NA	0	NA	0
(-)- (S)-Cathinone hydrochloride	0	0	0	0	0	0	3	0	0	0
(4R,5S)-4-methyl-5-phenyl-4,5-dihydro-1,3-oxazol-2-amine	0	0	-1.33	0	0	0	-0.67	0	-0.67	0
Mescaline hydrochloride	0	0	0	0	0	0	0	0	0	0
3,4,5-Trimethoxyamphetamine hydrochloride	0	0	0	0	0	0	0.67	0	-0.67	0
(+)-Metazocine fumarate (2:1)	0	0	-0.67	-2.67	-5	0	0.67	-1.33	-1.67	0

1-Methyl-4-phenyl-4-propionoxypiperidine hydrochloride	0	0	-0.67	-2.67	-3.33	0	0	0	0	0
N-(4-Fluorophenyl)-N-[1-(2-phenylethyl)-4-piperidyl] propanamide	0	0	-7	-7.67	-7	0	0.67	-1.33	0	0
N-(1-Benzyl-4-piperidyl)-N-phenylpropanamide hydrochloride	0	0	-7	-7	-6.33	0	0	-1.33	0	0
1-(7-Methoxy-1,3-benzodioxol-5-yl)propan-2-amine hydrochloride	0	0	0	0	0	0	-0.67	-0.67	-1	0
N-[1-(2-Hydroxy-2-phenylethyl)-4-piperidyl]-N-phenylpropanamide hydrochloride	0	0	-5.33	-5.33	-5.33	0	0	-0.67	-2	0
N-Phenyl-N-[1-(2-thienyl)methyl-4-piperidyl]propanamide hydrochloride	-0.67	0	-7	-7.67	-7	0	0	-3.33	-1.33	0
N-Phenyl-N-[1-[2-(2-thienyl)ethyl]-4-piperidyl]propanamide hydrochloride (1:1)	0	0	-8.33	-8.33	-7	0	-1.33	-2.67	-1.33	0
R-(-)-2,5-Dimethoxy-4-methylamphetamine hydrochloride	0	0	-1.33	-2.67	-0.67	0	0	0	0	0
2,5-Dimethoxyamphetamine hydrochloride	0	0	0	0	0	0	-0.67	0	0	0
1-(1,3-Benzodioxol-5-yl)-N-hydroxypropan-2-amine hydrochloride	2	0	0	0	0	0	0	0	0	0
(S)-(+)-N-Ethylamphetamine hydrochloride	0	0	-0.67	0	-2	0	0	0	0	0
(-)-Fenfluramine hydrochloride	0	0	-7	-7	-6.33	0	0	-0.67	0	0
Amobarbital	0	0	0	0	0	0	0.67	0	-2	0
S-(-)-Secobarbital	0	0	0	0	-1.33	0	0	0	0	0
Alfentanil hydrochloride	0	0	0	-2	0	0	0	0	0	0
1-Phenylcyclohexylamine hydrochloride	0	0	-3.67	-5.33	-2	0	0	0	0	0
1-Benzylpiperazine difumarate	0	0	0	0	0	0	0	0	0	0
Dihydroisocodeine	0	0	0	0	0	0	0	0	0	0
Dihydromorphine	0	0	0	0	-0.67	0	0	0	0	0
1-(1,3-Benzodioxol-5-yl)-N-ethylpropan-2-amine hydrochloride	0	0	-5.33	-5.33	-6.67	0	0	-1.33	-0.67	0
1-(1,3-Benzodioxol-5-yl)-N-methylpropan-2-amine hydrochloride	-0.67	0	-0.67	-0.67	-2.67	0	0	-1.33	-0.67	-0.67

Fenethylline hydrochloride	-2.67	-0.67	-2	-4	-0.67	0	-0.67	-4	-0.67	-2
alpha-Methylfentanyl hydrochloride	0	0	-6.33	-7	-6.33	1	0	0	0	0
Varenicline tartrate	0	0	NA	NA	NA	NA	NA	0	NA	0
Acifluorfen-sodium	0.67	0	0	0	0	0	0	0	0.67	0
Alacepril	0	0	0	0	0	0	0	0	0	0
Cefetamet pivoxil HCl	0	0	0	0	0	0	-0.67	0	0	0
Cefozopran hydrochloride	0	0	0	0	0	0	0	0	0	0
Etizolam	0	0	-2	-1.33	-0.67	0	0	0	-0.67	0
Lobenzarit sodium	-0.67	0	0	0	0	0	0.67	0	0	0
Lysozyme hydrochloride	0	0	0	0	0	0	0	0	0	0
Sulbenicillin disodium	0	0	0	0	0	0	0	0	-0.67	0
Xipamide	0	0	NA	NA	NA	NA	NA	0	NA	0
Tirofiban hydrochloride monohydrate	0	0	NA	NA	NA	NA	NA	0	NA	0
Landiolol hydrochloride	0	0	NA	NA	NA	NA	NA	0	NA	0
Biotin	0	0	NA	NA	NA	NA	NA	0	NA	0
Propamidine	1.33	0	-9	-9	-9	0	-0.33	0	0	0
Cythioate	0	0	-1.33	-0.67	-4	0	0.67	0	0	0
Nifenalol	0	0	NA	NA	NA	NA	NA	0	NA	0
Sulfaethoxypyridazine	0	0	0	0	0	0	0.67	0	0.67	0
Triamcinolone diacetate	0	0	NA	NA	NA	NA	NA	0	NA	0
Fexofenadine hydrochloride	0	0	NA	NA	NA	NA	NA	0	NA	0
Niceritrol	0	0	NA	NA	NA	NA	NA	0	NA	0
Ioxilan	0	0	NA	NA	NA	NA	NA	0	NA	0
Bamifylline Hydrochloride	0.67	0	NA	NA	NA	NA	NA	0	NA	0
Cadralazine	0.67	0	NA	NA	NA	NA	NA	0	NA	0
Oberadilol	0	0	-9	-9	-9	0	0	-3.33	0	0
Iomazenil	-0.67	0	-2	-0.67	0	0	0	0	0	0
Depolipon	0	0	-9	-9	-9	0	-1.33	-0.67	-0.67	0
Phenyl 11-iodo-10-undecynoate	-0.67	0	-2	0	0	0	0.67	0	0	0
Tolvaptan	-2	0	-5.33	-7	-3.67	0	0	0	-0.67	0
Anacolin	0.67	0	-7	-7	-6.33	0	0	0	0	0
Bimosiamose	0	0	0	0	0	0	0.67	0	0	0
Betiatide	0.67	0	0.67	0.67	0	0	0	0	0	0
Acetylpheneturide	0	0	0	0	0	0	0	0	-0.67	0
Blonanserin	-0.67	0	0	-2	-4	0	0	0	-2.67	0
Dicethiamine hydrochloride	0	0	0	0	0	0	-0.67	0	0.67	0

Cloforex	0	0	-1.33	-3.33	-0.67	0	0.67	0	0	0
Pelubiprofen	0	0	0	0	0	0	0	0	0	0
Risarestat	0	0	-1.33	0	0.67	0	0	0	0	0
Diponium bromide	0	0	-9	-9	-9	0	0	0	0	0
Emiglitate	0	0	0	0	0	0	0.67	0	0	0
Etomidoline	0	0	-2	-4.67	-2	0	0	-2.67	-2.67	0
Fezatione	-2	0	-5.33	-3.67	-2	0	-1.33	-1.33	0	0
Itasetron	0	0	0	0	0	0	0	0	0	0
Pamicogrel	0	-2.67	0	0	0	0	0	0	0	0
Esonarimod	2	0	0	0	0	0	0	0	0	0
Dalcotidine	1.33	0	0	0	0	0.67	-0.67	0	0	0
Melinamide	-7	-2	0	0	0	0	0	-4	0	0
Meluadrine	0	0	0	0	0	0	0	0	0	0
Oxendolone	-1.33	0	-7	-7	-7	0	-0.67	0	1.33	0
Penflutizide	0	0.67	0	-0.67	0	0	0	0	0	0
Doranidazole	0	0	0	0	0	0	-1.33	0	0	0
Prifinium bromide	0	0	-9	-9	-7.67	0	1.67	0	1	0
Sulfamethomidine	0	0	0	0	0.67	0	0	0	0	0
Tolindate	0	0	-5.33	-3.67	-2	0	0	-0.67	-0.67	0
Acreozast	0	0	0	0	0	0	0	0	1	0
Cefteram	0	0	1	0.67	1.33	0	-0.67	0	0	0
Glyclopyramide	0	0	0	0	0	0	0	0	-0.67	0
Rapacuronium bromide	0	0	-1.33	-2.67	-0.67	0	0	0	0	0
Temafloxacin	0.67	0	0	0	0	0	0	0	0.67	0
Propanidid	0	0.67	-1.33	-3.67	-1.33	0	0	0	0.67	0
Nitrefazole	0	0	0	0	0	0	0	0	0	0
Clometacine	0	0	0	0	0	0	0	-1.33	0	0
Pifoxime	0	0	-1.33	-2	-1.33	0	0.67	0	0	0
4'-Piperidinylcarbonylmethoxy acetophenone	0	0	0	0	0	0.67	0	0	1	0
Fenclofenac	0	0	0	0	0	0	0	0	0	0
Amineptine	0	0	0	0	0	0	-1.33	0	0	0
Flosequinan	0	0	0	0	0	0	-0.67	0	-0.67	0
Tolrestat	0	0	0	0	0.67	0	0	0	0	0
Tandospirone	0	0	-2	-0.67	-0.67	0	0	0	0	0

Phenoxypropazine	0.67	0	0	0	0	0	0	0	0	0
Amoproxan	0	0	-1.33	-1.33	0	0	0	0	-0.67	0
Glybuzole	0	0	0	0	0	0	0	0	0	0
Safrazine hydrochloride	0	0	0	0	0	0	0.67	0	0	0
Ethyl 4-nitrophenyl ethylphosphonate	-0.67	-1.33	0	-2.67	-1.33	0	0	-3.67	0.67	0
Mefruside	0	0	0	0	0	0	0	0	0.67	0
Flomoxef	0.67	0	0	0	0	0	0	0	0	0
Sitafloxacin hydrate	0	0	0	0	0	0	0	0	-0.67	0
Aceglatone	-1.33	0	-7	-7	-3.67	0	0	0	0	0
Sulcaine	0	0	-9	-7.67	-9	0	0	-0.67	0	0
N,N-Dimethyl-2-(2-phenylacetamido)acetamide	1.33	0	0	0	0	0	0	0	-0.67	0
Mepranoprofen arbamel	0	0	-3.67	-5.33	-4	0	0	0	-0.67	0
Alloclamide	0	0	-5.33	-7	-4.67	0	0	0	-2.67	0
Bucolome	0	0	0	0	0	0	0	0	0	0
Fasoracetam	0	0	0	0	0	0	0.67	0	-2	0
Lanperisone hydrochloride	0	0	-9	-9	-9	0	0	0	-0.67	0
5alpha-Androstan-3beta-ol	-1.33	0	0	0	0	1.67	0.67	0	0	0
Diloxanide furoate	0	0	NA	NA	NA	NA	NA	0	NA	0
cAMP	0	0	NA	NA	NA	NA	NA	0	NA	0
4-Hydroxy-3-methoxybenzaldehyde	0	0	NA	NA	NA	NA	NA	0	NA	0
Todralazine hydrochloride	0	0	NA	NA	NA	NA	NA	0	NA	0
N-Acetyl sulfamethoxazole	0	0	NA	NA	NA	NA	NA	0	NA	0
Methsuximide	0	0	0	0	0	0	0	0	0	0
Zaltoprofen	0	0	NA	NA	NA	NA	NA	0	NA	0
Mebutamate	0	0	NA	NA	NA	NA	NA	0	NA	0
Sodium 3, 4-dimethylphenyl-glyoxylate	0	0	NA	NA	NA	NA	NA	0	NA	0
Cloxazolam	0	0	NA	NA	NA	NA	NA	0	NA	0
Docarpamine	0	0	NA	NA	NA	NA	NA	0	NA	0
Emorfazone	0	0	NA	NA	NA	NA	NA	0	NA	0
Glymidine sodium	0	0	NA	NA	NA	NA	NA	0	NA	0
Talbutal	0	0	NA	NA	NA	NA	NA	0	NA	0
Embutramide	0.67	0	NA	NA	NA	NA	NA	0	NA	0
Arimoclomol	0	0	NA	NA	NA	NA	NA	0	NA	0
Sulfacytine	0.67	0	NA	NA	NA	NA	NA	0	NA	0
Thenyldiamine	0.67	0	NA	NA	NA	NA	NA	0	NA	0
Isoaminile	0	0	NA	NA	NA	NA	NA	0	NA	0

Methyl 5-sulfamoyl-o-anisate	0	0	NA	NA	NA	NA	NA	0	NA	0
Darusentan	0	0	NA	NA	NA	NA	NA	0	NA	0
1H-Tetrazol-1-acetic acid	0	0.67	NA	NA	NA	NA	NA	0	NA	0
Acephylline	0	0	NA	NA	NA	NA	NA	0	NA	0
3,5-Di-tert-butyl-4-hydroxybenzaldehyde	-0.67	0	NA	NA	NA	NA	NA	0	NA	0
6-Methoxy-2-naphthalaldehyde	0	0	NA	NA	NA	NA	NA	0	NA	0
Brofoxine	0	0	NA	NA	NA	NA	NA	0	NA	0
5,6-Dimethoxy-1-indanone	0	0	NA	NA	NA	NA	NA	0	NA	0
Dibenzosuberone	0	0	NA	NA	NA	NA	NA	0	NA	0
3,4,5-Trimethoxyphenylacetonitrile	0	0	NA	NA	NA	NA	NA	0	NA	0
Cintriamide	0	0	NA	NA	NA	NA	NA	0	NA	0
4-(4-Acetylpiperazin-4-yl)phenol	0	0	NA	NA	NA	NA	NA	0	NA	0
5-Acetylsalicylamide	0	0	NA	NA	NA	NA	NA	0	NA	0
Dimecrotic acid	0	0	NA	NA	NA	NA	NA	0	NA	0
3,3-Tetramethyleneglutarimide	0	0	NA	NA	NA	NA	NA	0	NA	0
Odiparcil	0	0	NA	NA	NA	NA	NA	0	NA	0
Tizoprolic acid	0	0	NA	NA	NA	NA	NA	0	NA	0
Methyl 5-(ethylsulfonyl)-o-anisate	0	0	NA	NA	NA	NA	NA	0	NA	0
Sulfaperin	0	0	NA	NA	NA	NA	NA	0	NA	0
(2Z)-{2-[(Chloroacetyl)amino]-1,3-thiazol-4-yl}(methoxyimino)acetic acid	0	0	NA	NA	NA	NA	NA	0	NA	0
Enciprazine	0	0	NA	NA	NA	NA	NA	0	NA	0
Propoxate	0	0	NA	NA	NA	NA	NA	0	NA	0
Prucalopride	0	0	NA	NA	NA	NA	NA	0	NA	0
Cycotiamine	0	0	NA	NA	NA	NA	NA	0	NA	0
Norfentanyl	0	0	NA	NA	NA	NA	NA	0	NA	0
Bravavir	0	0	NA	NA	NA	NA	NA	0	NA	0
Viloxazine hydrochloride	0	0	NA	NA	NA	NA	NA	0	NA	0
Furfenorex	0	0	NA	NA	NA	NA	NA	0	NA	0
Lotrafiban	0.67	0	NA	NA	NA	NA	NA	0	NA	0

SAMPLE_NAME	tox21-erb-bla-antagonist-p1-viability	tox21-erb-bla-p1-viability	tox21-err-p1-viability	tox21-esre-bla-p1-viability	tox21-fxr-bla-agonist-p2-viability	tox21-fxr-bla-antagonist-p1-viability	tox21-gh3-tre-antagonist-p1-viability	tox21-gr-hela-bla-antagonist-p1-viability	tox21-h2ax-cho-p2-viability	tox21-hdac-p1-viability
Acetazolamide	0	0	0	0	0	0	NA	NA	0	0
Chlormezanone	0	0	0	-0.67	0	0	NA	NA	0	0
Carbamazepine	0	0	0	0	0	0	NA	NA	0	0
Fenclonine	0	0	0	0	0	0	NA	NA	0	0
Clonidine	0	0	0	0	0.67	0	NA	NA	0	0
Cyclothiazide	0	0	0	0	0	0	NA	NA	0	0
Enoximone	0	0	0	0	0	0	NA	NA	0	0
Efaroxan	0	0	0.67	0	0	-0.67	NA	NA	0	0
Felbamate	0	0	0	0	0	0	NA	NA	0	0
Flumazenil	0	0	0	0	0	-0.67	NA	NA	0	0
Hydroxytacrine maleate	0	0	0	0	0	0	NA	NA	0	0
Lamotrigine	0	0	0	0	0	0	NA	NA	0	0
Leflunomide	0	0	0	-0.67	0	0	NA	NA	0	0
Molsidomine	0	0	0	0	0	0	NA	NA	0	0
Minoxidil	0	0	0	0	0	0	NA	NA	0	0
Nialamide	0	0	0	0	0	0	NA	NA	0	0
Pentoxifylline	0	0	0	0	0	0	NA	NA	0	0
Piroxicam	0	0	0	0	0	0	NA	NA	0.67	0
Primidone	0	0	0	0	0	0	NA	NA	0	0
Sulfaphenazole	0	0	0	0	0	0	NA	NA	0	0
Telenzepine	0	0	0	0	0	0	NA	NA	0	0
1-[2-(Trifluoromethyl)phenyl]-1H-imidazole	0	0	0	0	-0.67	0	NA	NA	0	0
Tropicamide	0	-0.67	0	0	0	0	NA	NA	0	0
Brimonidine	0	-0.67	0	0	0	0	NA	NA	0	0
Zardaverine	0	0	0	0	-0.67	0	NA	NA	0	0
Furosemide	0	-0.67	0	0	0	0	NA	NA	0	0
Metyrapone	0	0	0	0.67	0	0.67	NA	NA	0	0
Sulfaguanidine	0	0	0	0	0	0	NA	NA	0	0
Sulfinpyrazone	0	0	0	0	0	0	NA	NA	0	0
Phenacetin	0	0	0	0	0.67	0	NA	NA	0	0
Bendroflumethiazide	0	0	0	0	0.67	0	NA	NA	0	0
Phensuximide	0	0	0	0	0	0	NA	NA	0	0
Benzthiazide	0	0	0	0	0	0	NA	NA	0	0

Azacyclonol	0	0	0	0	0	0	NA	NA	0	0
Sulfadimethoxine	0	0	0	0	0	0	NA	NA	0	0
Sulfapyridine	0	0	0	0	0	0	NA	NA	0	0
Metronidazole	0	0	0	0	0	0	NA	NA	0	0
alpha-Santonin	0	0	0	0	0.67	0	NA	NA	0	0
Tetrahydrozoline hydrochloride	0	0	0	0	0	0	NA	NA	0	0
Amylocaine hydrochloride	0	0	0	0	0.67	0	NA	NA	0	0
Trimethobenzamide hydrochloride	0	0	0	0	0	0	NA	NA	0	0
Proxyphylline	0	0	0	0	0	0	NA	NA	0	0
Clofibric acid	0	0	0	0	0	0	NA	NA	0	0
Meticrane	0	0	0	0	0	0	NA	NA	0	0
Midodrine hydrochloride	0	-0.67	0	0	0	0	NA	NA	0	0
Bromopride	0	0	0	0	0	0	NA	NA	0	0
Cyclopentolate hydrochloride	0	0	0	0	0	0	NA	NA	0	0
Selegiline hydrochloride	0	0	0	0	0	0	NA	NA	0	0
Ornidazole	0	0	0	0	0	0	NA	NA	0	0
Tinidazole	0	0	0	0	0	0	NA	NA	0	0
Gliclazide	0	0	0	0	0	0	NA	NA	0	0
Benfotiamine	0	0	0	0	0	0	NA	NA	0	0
Etandazole	0	0	0	-0.67	0.67	0	NA	NA	0	0
Probucol	0	0	0	0	0	0	NA	NA	0	0
Suxibuzone	0	0	0	0	0	0	NA	NA	0	0
Tiaprofenic acid	0	0	0	0	0	0	NA	NA	0	0
Etomidate	0	0	0	0	0	0	NA	NA	0	0
Pivampicillin	0	0	0	0	0	0	NA	NA	0	0
Acebutolol hydrochloride	0	0	0	0	0	0	NA	NA	0	0
Carteolol hydrochloride	0	0	0	0	0	0	NA	NA	0	0
Pipemidic acid	0	0	0	0	0	0	NA	NA	0	0
Meptazinol hydrochloride	0	0	0	-0.67	0.67	0	NA	NA	0	0
Iopamidol	0	0	0	0	0.67	0.67	NA	NA	0	0
Rolipram	0	0	0	0	0	0	NA	NA	0	0
Fluticasone propionate	0	0	0	0	0	0	NA	NA	0	0
Brinzolamide	0	0	0	0	0	0	NA	NA	0	0
Disopyramide	0	0	0	0	0	0	NA	NA	0	0
Cefotiam hydrochloride	0	0	0	0	0	0	NA	NA	0	0
Indapamide	0	0	0	0	0	0	NA	NA	0	0
Metoprolol tartrate	0	0	0	0	0	0.67	NA	NA	0	0
Cefmetazole sodium	0	0	0	0	0	0	NA	NA	0	0
Ketorolac	0	0	0	0	0	0	NA	NA	0	0
Dropropizine	0	0	0	0	0	0	NA	NA	0	0

Enalapril maleate	0	-0.67	0	0	0	0	NA	NA	0	0
Hydrocortisone	0	0	0	0	0	0	NA	NA	0	0
Methysergide	0	0	0	0	0	0	NA	NA	0	0
Flurofamide	0	0	0	0	0	0	NA	NA	0	0
Methyldopa	0	0	0	0	0.67	0	NA	NA	0	0
Clomethiazole	0	0	0	0	0.67	0	NA	NA	0	0
Remoxipride	0	0	0	0	0	0	NA	NA	0	0
AF-DX 116	0	0	0	0	0	0	NA	NA	0	0
Levcromakalim	0	0	0	0	0	0	NA	NA	0	0
Roquinimex	0	0	0	0	0	0	NA	NA	0	0
Pinacidil monohydrate	0	0	0	0.67	0.67	0	NA	NA	0	0
Remacemide hydrochloride	0	-0.67	0	0	0	0	NA	NA	0	0
Ipsapirone	0	0	0	0	0	0	NA	NA	0	0
Nicorandil	0	0	0	0	0.67	-0.67	NA	NA	0	0
Capobenic acid	0	0	-0.67	0	0	0	NA	NA	0	0
Sodium zomepirac	0	0	0	0	0	0	NA	NA	0	0
4-Chlorophenol	0	0	0	0	0	0	NA	NA	0	0
4-Aminobenzoic acid	0	0	0	-0.67	0	0	NA	NA	0	0
Diazinon	0	-0.67	0	0	0.67	0	NA	NA	0	0
Aspartame	0	0	0	0	0	0	NA	NA	0	0
Phenoxyacetic acid	0	-0.67	0	0	0	0	NA	NA	0	0
Anthranilic acid	0	0	0	0	0	0.67	NA	NA	0	0
Hexanamide	0	0	0	0	0	0	NA	NA	0	0
Acetanilide	0	0	0	0	0	0	NA	NA	0	0
4-Chloro-3-methylphenol	0	0	0	0	0	0	NA	NA	0	0
Hexanedioic acid	0	0	0	0	0	0	NA	NA	0	0
Allylthiourea	0	0	0	0	0	0	NA	NA	0	0
Salicylamide	0	0	0	0	0	0	NA	NA	0	0
Fenthion	0	0	0	0	0	0	NA	NA	0	0
Cotinine	0	0	0	0	0	0	NA	NA	0	0
Fenspiride hydrochloride	0	0	0	0	0	0	NA	NA	0	0
Naphazoline hydrochloride	0	0	0	0	0	0	NA	NA	0	0
Nomifensine maleate	0	0	0	0	0	0	NA	NA	0	0
Bisoprolol fumarate	0	0	0	0	0.67	0	NA	NA	0	0
Diuron	0	0	0	0	0	0	NA	NA	0	0
2-Chloro-4-nitrobenzamide	0	0	0	0	0	0	NA	NA	0	0
Phenacemide	0	0	-0.67	0	0	0	NA	NA	0	0
2-Aminoheptane sulfate	0	0	0	0	0.67	0	NA	NA	0	0
Acetarsol	0	0	0	0	0	0	NA	NA	0	0
Iproniazid sulfate	0	0	0	0	0	0	NA	NA	0	0

Vincamine	0	0	0	0	0	0	NA	NA	0	0
Mephenesin	0	0	0	0	0	0	NA	NA	0	0
Saccharin	0	0	0	0	0	0	NA	NA	0	0
Acetyl-L-leucine	0	0	0	0	0	0	NA	NA	0	0
Chromocarb	0	0	0	-0.67	0	0	NA	NA	0	0
Acexamic acid	0	0	0	0	0	0	NA	NA	0	0
Pipenzolate bromide	0	0	0	0	0	0	NA	NA	0	0
Adiphenine hydrochloride	0	-0.67	0	-0.67	0	0	NA	NA	0	0
Diosmin	0	0	-0.67	0	0	0	NA	NA	0	0
Pipobroman	0	0	0	0	0	0	NA	NA	0	0
Ramifenazone	0	0	0	0	0.67	0	NA	NA	0	0
Metaxalone	0	0	0	0	0	0	NA	NA	0	0
Secnidazole	0	0	0	0	0	0	NA	NA	0	0
Clopidol	0	0	0	0	0	0	NA	NA	0	0
Modafinil	0	0.67	0	0	0	0	NA	NA	0.67	0
Arbutin	0	0	0	0	0.67	0	NA	NA	0	0
2-Hydroxy-3-methylbenzoic acid	0	0	0	0	0.67	0	NA	NA	0	0
Phenyl 4-aminosalicylate	0	0	0	0	0	0	NA	NA	0	0
Salsalate	0	0	0	0	0	0	NA	NA	0	0
Folic acid	0	0	0	0	0	0	NA	NA	0	0
Clopidogrel bisulfate	0	0	0	0	0	0	NA	NA	0	0
Benzathine penicillin G	0	0	0	0	0	0	NA	NA	0	0
Tramadol hydrochloride	0	0	0	0	0	0	NA	NA	0	0
Huperzine A	0	0	0	0	0.67	0	NA	NA	0	0
N-Acetyl-L-tyrosine	0	0	0	-0.67	0.67	0	NA	NA	0	0
Phenicarbazide	0	0.67	0	0	0	0	NA	NA	0	0
Trimetozine	0	0	0	0	0	0	NA	NA	0	0
Iodoantipyrine	0	0	0	0	0	0	NA	NA	0	0
Warfarin sodium	0	0	0	0	0	0	NA	NA	0	0
Tegafur	0	-0.67	0	0	0.67	0	NA	NA	0	0
Hexobarbital	0	0	0	0	0	0	NA	NA	0	0
Benactyzine	0	0	0	0	0	0	NA	NA	0	0
Sorbinil	0	0	0	0	0	0	NA	NA	0	0
Methyl nicotinate	0	0	0	0	0	0	NA	NA	0	0
Hippuric acid	0	0	0	0	0	0	NA	NA	0	0
Eszopiclone	0	0	0	0	0	0	NA	NA	0	0
Cephalexin	0	0.67	0	0	0.67	0.67	NA	NA	0	0
Pepstatin	0	0	0	0	0	0	NA	NA	0	0
6,7-Dimethoxyquinazoline-2,4(1H,3H)-dione	0	0	0	-0.67	0	0	NA	NA	0	0
Tetridamine	0	0	0	0	0	0	NA	NA	0	0

Loretin	0	0	0	0	0	0	NA	NA	0	0
Tetrabenazine	0	0	0	0	0	0	NA	NA	0	0
4-Acetylaminophenylacetic acid	0	0	0	0	0	0	NA	NA	0	0
Lozilurea	0	0.67	0	0	0	0	NA	NA	0	0
Amanozine	0	0	0.67	0	0	0	NA	NA	0	0
Zaleplon	0	0	0	-0.67	0	0	NA	NA	0	0
Tizanidine hydrochloride	0	0	0	-0.67	0	0	NA	NA	0.67	0
Romazarit	0	0.67	0	0	0	0	NA	NA	0	0
Diamfenetide	0	0	0	0	0	0	NA	NA	0	0
Ethopabate	0	0	0	0	0.67	0	NA	NA	0	0
Sulazepam	0	0	0	0	0	0	-0.67	0	0	0
Sulfasuccinamide	0	0	0	0	0.67	0	NA	NA	0	0
Dazmegrel	0	0	0	0	0.67	0	NA	NA	0	0
Ambrisentan	0	0.67	0	0	0	0	NA	NA	0	0
Mecarbinate	0	-0.67	0	0	0	0	NA	NA	0	0
Isocaine	0	0	0	0	0	0	NA	NA	0	0
Ftaxilide	0	0	0	0	0	0	NA	NA	0	0
Naloxone	0	0	0	0.67	0	0	NA	NA	0	0
Ambenonium chloride	0	0	0	0	0.67	0	NA	NA	0	0
Butylscopolamine bromide	0	0	0	0	0	0	NA	NA	0	0
Tiotidine	0	0	0	0	0	0	NA	NA	0	0
Temephos	0	0	0	0	0	0	NA	NA	0	0
Olaquindox	0	0	0	0	0	0	NA	NA	0	0
Hexadecanoic acid	0	0	0	0	-0.67	0	NA	NA	0	0
Amiphenazole	0	0	0	0	0	0	NA	NA	0	0
Nicothiazone	0	0	0	0	0	0	NA	NA	0	0
Glyoctamide	0	0	0	0	0	0	NA	NA	0	0
Cinromide	0	0	0	0	0	0	NA	NA	0	0
Parapropamol	0	0	0	0	0	0	NA	NA	0	0
Talinolol	0	0	0	0	0	0	NA	NA	0	0
Dofetilide	0	0	0	0	0	0	NA	NA	0	0
Eprosartan mesylate	0	0	0	0	0	0	NA	NA	0	0
Pargeverine hydrochloride	0	0	0	0	0.67	0	NA	NA	0	0
Capecitabine	0	0	0	0	0	0	NA	NA	0	0
Etoricoxib	0	0	0	0	0	0	NA	NA	0	0
Cetraxate hydrochloride	0	0	0	0	0	0.67	NA	NA	0	0
Phenprobamate	0	0	0	0	0.67	0	NA	NA	0	0
Propyphenazone	0	0	0	0	0	0	NA	NA	0	0
Raltitrexed	-3	-6	0	0	-2	0	0.67	0	0	0
Voriconazole	0	0	0	0	0	0	NA	NA	0	0

Tiagabine hydrochloride	0	0	0	0	0	0	NA	NA	0	0
Dexrazoxane hydrochloride	0	0	0	0	0	0	NA	NA	0	0
Rufinamide	0	0	0	0	0	0	NA	NA	0	0
Iohexol	0	0	0	0	0	0	NA	NA	0	-0.67
Flamenol	0	0	0	0	0	0	NA	NA	0	0
Ethyl nicotinate	0	0	0	0	0	-0.67	NA	NA	0	0
alpha-Lipoic acid amide	0	0	0	0	0.67	0	NA	NA	0	0
Pyrithioxin dihydrochloride	0	0	0	0	0.67	0	NA	NA	0	0
Alovudine	0	0	0	-0.67	0	0	NA	NA	0	0
Carsalam	0	0	0	0	0	0.67	NA	NA	0	0
Temozolomide	0	0	0	0	0	0	NA	NA	0	0
Pemirolast potassium	0	0.67	0	0	0.67	0	NA	NA	0	0
Trospium chloride	0	0	0	0	0	-0.67	NA	NA	0	0
Eprazinone dihydrochloride	0	0	0	0	0	0	NA	NA	0	0
Clinofibrate	0	0	0	0	0	0	NA	NA	0	0
Zofenopril	0	0	0	0	0	0	NA	NA	0	0
Vecuronium bromide	0	0	0	0	0	0	NA	NA	0	0
Lenalidomide	0	0	0	0	0	0	NA	NA	0	0
Pipethanate ethylbromide	0	0	0	0	0	0	NA	NA	0	0
Pemoline	0	0	0	0	0	0	NA	NA	0	0
Ipidacrine hydrochloride hydrate	0	0	0	0	0	0	NA	NA	0	0
Lazabemide hydrochloride	0	0	0.67	0	0	0	NA	NA	0	0
Loxoribine	0	0	0	0	0.67	0	NA	NA	0	0
Desonide	0	0	0	0	0	0	NA	NA	0	0
Fenobam	0	0	0	0	0	0	NA	NA	0	0
Suplatast tosylate	0	0	0	0	0	0	NA	NA	0	0
Timepidium bromide	0	0	0	0	0	0	NA	NA	0	0
Nitarsone	0	0	0	0	0	0	NA	NA	0	0
Mebrofenin	0	0	0	0	0.67	0	0.67	1	0	0
Acetyl tributyl citrate	0	0	0	0	1	0	3.33	-1	-0.67	0
Imidapril hydrochloride	0	0	0	0	0.67	0	NA	NA	0	0
(-)- (S)-Cathinone hydrochloride	0	0	0	-0.67	1	-0.67	0	0	0	0
(4R,5S)-4-methyl-5-phenyl-4,5-dihydro-1,3-oxazol-2-amine	0	-0.67	0	0	0	0	0	0	0	0
Mescaline hydrochloride	0	0	0	0	0	0	0.67	0	0	0
3,4,5-Trimethoxyamphetamine hydrochloride	0	0	0	0	1.33	-0.67	0	0	0	0
(+)-Metazocine fumarate (2:1)	0	0	0	0	0	0	0	0	0	0

1-Methyl-4-phenyl-4-propionoxypiperidine hydrochloride	0	0	0	0	0	0	1.33	0	-0.67	0
N-(4-Fluorophenyl)-N-[1-(2-phenylethyl)-4-piperidyl] propanamide	0	0	0	0	0	0	-5	0	0	0
N-(1-Benzyl-4-piperidyl)-N-phenylpropanamide hydrochloride	0	0	0	0	0	0	0	0	0	0
1-(7-Methoxy-1,3-benzodioxol-5-yl)propan-2-amine hydrochloride	0	0	0	0	0	0	-1.67	0	0	0
N-[1-(2-Hydroxy-2-phenylethyl)-4-piperidyl]-N-phenylpropanamide hydrochloride	0	0	0	0	0.67	0	-1.67	0.67	0	0
N-Phenyl-N-[1-(2-thienyl)methyl-4-piperidyl]propanamide hydrochloride	0	0	-1.33	0	0	0	-0.67	0	0	0
N-Phenyl-N-[1-[2-(2-thienyl)ethyl]-4-piperidyl]propanamide hydrochloride (1:1)	0	0	-0.67	0	0	0	-2	0	0	0
R-(-)-2,5-Dimethoxy-4-methylamphetamine hydrochloride	0	0	0	0	0	-0.67	-2.67	0	0	0
2,5-Dimethoxyamphetamine hydrochloride	0	0	0	-0.67	1	0	-0.67	0	0	0
1-(1,3-Benzodioxol-5-yl)-N-hydroxypropan-2-amine hydrochloride	0	0	0	-0.67	2	0	0	0	0	0
(S)-(+)-N-Ethylamphetamine hydrochloride	0	0	0	-1	0.67	0	-0.67	0	0	0
(-)-Fenfluramine hydrochloride	0	0	0	0	1.33	0	-2	0	0	0
Amobarbital	0	0	0	0	-0.67	-0.67	1.67	0	0	0
S-(-)-Secobarbital	0	0	0	0	0.67	0	0	0	0	0
Alfentanil hydrochloride	0	0	0	0	0	0	0.67	0	0	0
1-Phenylcyclohexylamine hydrochloride	0	0	-0.67	0	0	0	-4.67	0	0	0
1-Benzylpiperazine difumarate	0	0	0	0	0	0	0	0	0	0
Dihydroisocodeine	0	0	0	-0.67	0	0.67	0	0.67	0	0
Dihydromorphine	0	0	0	0	0.67	0	0	0	0	0
1-(1,3-Benzodioxol-5-yl)-N-ethylpropan-2-amine hydrochloride	0	0	0	0	0	0	0	-0.67	0	0
1-(1,3-Benzodioxol-5-yl)-N-methylpropan-2-amine hydrochloride	0	0	0	-3.67	0	-0.67	0.67	0	0	0

Fenethylline hydrochloride	0	0	-1.33	0	-0.67	0	-0.67	0	0	0
alpha-Methylfentanyl hydrochloride	0	0	0	0	0	0	-1.33	0	0	0
Varenicline tartrate	0	0	0	0	0	0	NA	NA	0	0
Acifluorfen-sodium	0	0	0	0	0.67	0	0.67	0	0	0
Alacepril	0	0	0	0	0.67	0	0	0	0	0
Cefetamet pivoxil HCl	0	0	0	0	0	0.67	0	0	0	0
Cefozopran hydrochloride	0	0	0	0	0.67	0	0	-0.67	0	0
Etizolam	0	0	0	0	0	0	0	-0.67	0	0
Lobenzarit sodium	0	0	0	0	0.67	0	0	0	0.67	0
Lysozyme hydrochloride	0	0	0	0	0.67	0	0.67	0	0	0
Sulbenicillin disodium	0	0	0	0	0	0	0	0	0	0
Xipamide	0	0	0	0.67	0	0	NA	NA	0	0
Tirofiban hydrochloride monohydrate	0	0	0	0	0	0	NA	NA	0	0
Landiolol hydrochloride	0	0	0	0	0	0.67	NA	NA	0	0
Biotin	0	0	-0.67	0	0	0	NA	NA	0	0
Propamidine	0	0	0	0	0	0.67	0	0	0	0
Cythioate	0	0	0	0	0	0	0	-0.67	0	0
Nifenalol	0	0	0	0	0	0	NA	NA	0	0
Sulfaethoxypyridazine	0	0	0	0	0	0	0	0.67	0	0
Triamcinolone diacetate	0	0	0	0	0	0	NA	NA	0	0
Fexofenadine hydrochloride	0	0	0	0	0	0	NA	NA	0	0
Niceritrol	0	0	0	0	0	0	NA	NA	0	0
Ioxilan	0	0	0	0	0.67	0	NA	NA	0	0
Bamifylline Hydrochloride	0	0	0	0	0.67	0	NA	NA	0	0
Cadralazine	0	0	0	0	0	0	NA	NA	0	0
Oberadilol	0	-1.33	-0.67	0	0	0	0	0	0	0
Iomazenil	0	0	0	0	0	0	0	0	0	0
Depolipon	0	-1.33	0	0	0	0	-1.67	0	0	0
Phenyl 11-iodo-10-undecynoate	0	0	0	0	0	0	-0.67	0	0	0
Tolvaptan	-0.67	-3.67	-0.67	0	0	0	-0.67	0	0	0
Anacolin	0	0	0	0	0	0	0	0	0	0
Bimosiamose	0	0	0	0	1	0.67	1.33	0	0	0
Betiatide	0	0	0	0	0	0	0	0	0	0
Acetylpheneturide	0	0	0	0	0	0	0.67	0	0	0
Blonanserin	0	-0.67	0	0	0	0	-7	-2.33	0	0
Dicethiamine hydrochloride	0	0	0	0	0	0	0	0.67	0	0
Cloforex	0	0	0	0	0	0	-0.67	-0.67	0	0
Pelubiprofen	0	0	0	0	0	0	0.67	0	0	0

Risarestat	-2.67	-3.67	0	0	0	0	-0.67	0	0	0
Diponium bromide	0	0	0	0.33	0	0	0	0	0	0
Emiglitate	0	0	0	-0.67	0	0	0.67	0	0	0
Etomidoline	0	0	0	-0.67	0	0	-0.67	0.67	0	0
Fezatione	0	-0.67	0	0	0	0	-1.67	0	-0.67	0
Itasetron	0	0	0	0	0	0	0	0.67	0	0
Pamicogrel	0	-0.67	0	0	-2	0	-2.67	0	0	0
Esonarimod	0	0.67	0	0	1	0	0	-1	0	0
Dalcotidine	0	0	0	0	0	0	0.67	0	0	0
Melinamide	0	-1.33	-2	0	0	0	0	0	0	0
Meluadrine	0	0	0	0	0	0	0	0	0	0
Oxendolone	0	0	-2	0	0	0	0	0	0	0
Penflutizide	0	0	0	0	0	0	0	0	0	0
Doranidazole	0	0	0	0	0	0	1.67	0	0	0
Prifinium bromide	0	0	0	0	2.33	0	0	0	0	0
Sulfamethomidine	0	0	0	0	0	0	0	0.67	0	0
Tolindate	0	-2	0	0	0	-0.67	0	0	-2.67	0
Acreozast	0	0	0	0	0	0	0.67	0.67	0	0
Cefteram	0	0	0	0	-0.67	0	0.67	0	0	0
Glyclopyramide	0	0	0	0	0	0	0.67	0	0	0
Rapacuronium bromide	0	0	0	0	-0.67	0	1.67	0	0	0
Temafloxacin	0	0.67	0	0	0	0	0	0	0	0
Propanidid	0	0	0	0	0	0	0	0	0	0
Nitrefazole	0	0	0	0	0	0	0	0	0	0
Clometacine	0	0	0	0	2	0	1.33	0	0	0
Pifoxime	0	0	0	0	0	0	0	0	0	0
4'-Piperidinylcarbonylmethoxyacetophenone	0	0	0	0	0	0	-0.67	0	0	0
Fenclofenac	0	0	0	0	0	0	0.67	0.67	0	0
Amineptine	0	0	0	0	2	0	-1	-0.67	0	0
Flosequinan	0	0	0	0	1	0	0	0	0	0
Tolrestat	0	0	0	0	0	0	0	0	0	0
Tandospirone	0	0	0	-0.67	0	0	0	-1	0	0
Phenoxypropazine	0	0	0.67	0	0	0	0	0	0	0
Amoproxan	0	0	0	0	0	0	0	0	0	0
Glybuzole	0	0	0	0	0	0	0	0	0	0
Safrazine hydrochloride	0	0	0	0	0	0	0	0	0	0
Ethyl 4-nitrophenyl ethylphosphonate	0	0	-6	0	2	0	0	0	0	0
Mefruside	0	0	0	0	0	0	0	0	0	0
Flomoxef	0	0	0	-1.33	0	0	0	0	0	0
Sitafloxacin hydrate	0	0	0	0	0	0	0.67	0	0	0

Aceglatone	0	0	0	0	0	0	0	0	0	0
Sulcaine	0	-2	0	0	-1	0	0	0	0	0
N,N-Dimethyl-2-(2-phenylacetamido)acetamide	0	0	0	0	1	0	0	0	0	0
Mepranoprofen arbamel	0	0	0	0	0	0	0.67	0	0	0
Alloclamide	0	0	0	0	0	0	-4	0	0	0
Bucolome	0	0	0	0	0.67	0	0	0	0	0
Fasoracetam	0	0.67	0	0	0	0	-0.67	0	0	0
Lanperisone hydrochloride	0	-0.67	0	0	0	0	0	1.67	0	0
5alpha-Androstan-3beta-ol	0	0	0	0	0	-0.67	0	0	-0.67	0
Diloxanide furoate	0	0	0	0	0	0	NA	NA	0	0
cAMP	0	0	0	0	0	0	NA	NA	0	0
4-Hydroxy-3-methoxybenzaldehyde	0	0	0	0	0	0	NA	NA	0	0
Todralazine hydrochloride	0	0	0	0	0.67	-0.67	NA	NA	0	0
N-Acetyl sulfamethoxazole	0	0	0	0	0	0	NA	NA	0	0
Methsuximide	0	0	0	0	0	0.67	0.67	0	0	0
Zaltoprofen	0	0	0	0	0.67	0	NA	NA	0	0
Mebutamate	0	0	0	0	0	0	NA	NA	0	0
Sodium 3, 4-dimethylphenyl-glyoxylate	0	0	0	-0.67	0	0	NA	NA	0	0
Cloxazolam	0	0	0	0	0	0	NA	NA	0	0
Docarpamine	0	0	0	-0.67	0	0	NA	NA	0	0
Emorfazone	0	0	0	0	0	0	NA	NA	0	0
Glymidine sodium	0	0	0	0	0	0.67	NA	NA	0	0
Talbutal	0	0	0	0	0	0	NA	NA	0	0
Embutramide	0	0	0	0	0	0	NA	NA	0	0
Arimoclomol	0	0	0	0	0	0	NA	NA	0	0
Sulfacytine	0	0	0.67	0	0	0	NA	NA	0	0
Thenyldiamine	0	0	0	0	0.67	0	NA	NA	0	0
Isoaminile	0	0	0	0	0	0	NA	NA	0	0
Methyl 5-sulfamoyl-o-anisate	0	0	0	-0.67	0	0	NA	NA	0	0
Darusentan	0	0	0	0	0	0	NA	NA	-0.67	0
1H-Tetrazol-1-acetic acid	0	0	0	0	0	0	NA	NA	0	0
Acephylline	0	-0.67	0	0	0	0	NA	NA	0	0
3,5-Di-tert-butyl-4-hydroxybenzaldehyde	0	0	0	0	0.67	0	NA	NA	0	0
6-Methoxy-2-naphthalaldehyde	0	0	0	0	0	0.67	NA	NA	0	0
Brofoxine	0	0	0	0	0	0	NA	NA	0	0

5,6-Dimethoxy-1-indanone	0	0	0	0	0	0	NA	NA	0	0
Dibenzosuberone	0	0	0	0	0	0	NA	NA	0	0
3,4,5-Trimethoxyphenylacetonitrile	0	0	0	0	0.67	0.67	NA	NA	0	0
Cintriamide	0	0	0	0	0.67	0	NA	NA	0	0
4-(4-Acetylpiperazin-4-yl)phenol	0	0	0	0	0	0	NA	NA	0	0
5-Acetylsalicylamide	0	0	0	0	0	0	NA	NA	0	0
Dimecrotic acid	0	0	0	0	0	0	NA	NA	0	0
3,3-Tetramethyleneglutarimide	0	0	0	0	0	0.67	NA	NA	0	0
Odiparcil	0	0	0	0	0	0	NA	NA	0	0
Tizoprolic acid	0	0	0	0	0	0	NA	NA	0	0
Methyl 5-(ethylsulfonyl)-o-anisate	0	0	0	0	0	0.67	NA	NA	0	0
Sulfaperin	0	0	0	0	0	0	NA	NA	0	0
(2Z)-{2-[(Chloroacetyl)amino]-1,3-thiazol-4-yl}(methoxyimino)acetic acid	0	0	0	0	0	0	NA	NA	0	0
Enciprazine	0	0	0	0	0	0	NA	NA	0	0
Propoxate	0	0	0	0	0	0	NA	NA	0	0
Prucalopride	0	0	0	0	0	-0.67	NA	NA	0	0
Cycotiamine	0	0	0	0	-0.67	0	NA	NA	0	0
Norfentanyl	0	0	0	-0.67	0	0	NA	NA	0	0
Bravavir	0	0	0.67	0	0	0	NA	NA	0	0
Viloxazine hydrochloride	0	0	0	0	0	0	NA	NA	0	0
Furfenorex	0	0	0	0	0	-0.67	NA	NA	0	0
Lotrafiban	0	0	0	0	0	0	NA	NA	0	0

SAMPLE_NAME	tox21-hre-bla-agonist-p1-viability	tox21-hse-bla-p1-viability	tox21-mitotox-p1-viability	tox21-nfkb-bla-agonist-p1-viability	tox21-p53-bla-p1-viability	tox21-pgc-err-p1-viability	tox21-ppard-bla-agonist-p1-viability	tox21-ppard-bla-antagonist-p1-viability	tox21-pparg-bla-antagonist-p1-viability	tox21-pr-bla-agonist-p1-viability
Acetazolamide	0	-0.67	NA	0	NA	0	0	0	0	0
Chlormezanone	0	0	NA	0	NA	0	0	0	0	0
Carbamazepine	0	0	NA	0	NA	0	0	0	0	0
Fenclonine	0	-0.67	NA	0	NA	0	0	0	0	0
Clonidine	0	0	NA	0	NA	0	0	0	0	0.67
Cyclothiazide	0	0	NA	0	NA	0	-0.67	0	0	0
Enoximone	0	0	NA	0.67	NA	0	-0.67	0	0	0
Efaroxan	0	0	NA	0	NA	0	0	0	0	-0.67
Felbamate	0	-0.67	NA	0	NA	0	-0.67	0	0	0
Flumazenil	0	0	NA	0	NA	0	0	-0.67	0	0
Hydroxytacrine maleate	0	0	NA	0	NA	0	0	0	0	0
Lamotrigine	0	0	NA	0	NA	0	0	0	0	0
Leflunomide	0	0	NA	0	NA	0	0	0	0	-0.67
Molsidomine	0	0	NA	0	NA	0	0	0	0	0
Minoxidil	0	0	NA	0	NA	0	0	0	0	0
Nialamide	0	0	NA	0	NA	0	0	0	0	0
Pentoxifylline	0	0	NA	0	NA	0	0	0	0	0
Piroxicam	0	0	NA	0	NA	0	0	0	0	0
Primidone	0	0	NA	0	NA	0	0	0	0.67	0
Sulfaphenazole	0	0	NA	0	NA	0	0	0	0	-0.67
Telenzepine	0	0	NA	0	NA	0	0	0	0	-0.67
1-[2-(Trifluoromethyl)phenyl]-1H-imidazole	0	0	NA	0	NA	0	0	-0.67	0	0
Tropicamide	0	0	NA	0	NA	0	0	0	0	0
Brimonidine	0	0	NA	-0.67	NA	0	0	0	0	0
Zardaverine	0	0	NA	0	NA	0	0	0	0	-0.67
Furosemide	0	-0.67	NA	0	NA	0	0	0.67	0	0
Metyrapone	0	0	NA	0	NA	0	0	-0.67	0	0
Sulfaguanidine	0	0	NA	0	NA	0	0	0	0	0
Sulfinpyrazone	0	0	NA	0	NA	0	-0.67	-0.67	0	0
Phenacetin	0	0	NA	0	NA	0	0	0	0	0
Bendroflumethiazide	0	0	NA	0	NA	0	0	0	0	0
Phensuximide	0	0.67	NA	0	NA	0	0	-0.67	0	0
Benzthiazide	0	0	NA	0	NA	0	0	0	0.67	0

Azacyclonol	0	0	NA	0	NA	0	-0.67	-0.67	0	0
Sulfadimethoxine	0	0	NA	0	NA	0	0.67	0	0	-0.67
Sulfapyridine	0	0	NA	0	NA	0	0	0	-0.67	-0.67
Metronidazole	0	0	NA	0	NA	0	0	0	0	0
alpha-Santonin	0	0	NA	0	NA	0	0	0	0	0
Tetrahydrozoline hydrochloride	0	0	NA	-0.67	NA	0	0	0	0	0
Amylocaine hydrochloride	0	0	NA	0	NA	0	0	0	0	0
Trimethobenzamide hydrochloride	0	0	NA	0	NA	0	0	0	0	0
Proxyphylline	0	0	NA	0	NA	0.67	0	0	0	0
Clofibric acid	0	0	NA	0	NA	0	0	-0.67	0	0
Meticrane	0	0	NA	0	NA	0	0	0	0	0
Midodrine hydrochloride	0	0	NA	0	NA	0	0	-0.67	0.67	-0.67
Bromopride	0	0	NA	0	NA	0	0	0.67	0	0
Cyclopentolate hydrochloride	0	0.67	NA	-0.67	NA	0	0	0	0	0
Selegiline hydrochloride	0	0	NA	0	NA	0	0	0	0	0
Ornidazole	0	-0.67	NA	0	NA	0	0	0	0	0
Tinidazole	0	0	NA	0	NA	0	0	0.67	0	0
Gliclazide	0	0	NA	-0.67	NA	0	0	0	0.67	0
Benfotiamine	0	0	NA	0	NA	0	0	0	0.67	0
Etandazole	0	0	NA	0	NA	0	0	0	0	0
Probucol	0	0	NA	0	NA	0	0	0	0	-0.67
Suxibuzone	0	0	NA	0	NA	0	0	0	0	0
Tiaprofenic acid	0	0	NA	0	NA	0	0	0.67	0	0
Etomidate	0	0	NA	0	NA	0	0	0	0	0
Pivampicillin	0	0	NA	0	NA	0	-0.67	0	0	0
Acebutolol hydrochloride	0	0	NA	0	NA	0	-0.67	-0.67	0	0
Carteolol hydrochloride	0	0	NA	0	NA	0	0	0	0.67	0
Pipemidic acid	0	0	NA	0	NA	0	0	0	0	0
Meptazinol hydrochloride	0	0	NA	0.33	NA	0	0	0	0	0
Iopamidol	0	0	NA	0	NA	0	0	-0.67	0	0
Rolipram	0	0	NA	0	NA	0	0	0	0	0.67
Fluticasone propionate	0	0	NA	0	NA	0	0	-0.67	0	0
Brinzolamide	0	0	NA	0	NA	0	0	0	0	-0.67
Disopyramide	0	0.67	NA	-0.67	NA	0	0	0	0	0
Cefotiam hydrochloride	0	0	NA	0	NA	0	0	0	0	-0.67
Indapamide	0	0	NA	0	NA	0	0	-0.67	0	0
Metoprolol tartrate	0	0	NA	0	NA	0	-0.67	-0.67	0	0
Cefmetazole sodium	0	0	NA	-0.67	NA	0	0	-0.67	0.67	0.67
Ketorolac	0	0	NA	0	NA	0	0	0	0	-0.67
Dropropizine	0	0	NA	-0.67	NA	0	0	0	0	0

Enalapril maleate	0	0.67	NA	-0.67	NA	0	-0.67	-0.67	0	0
Hydrocortisone	0	0	NA	0	NA	0	0	0	0	0
Methysergide	0	0	NA	-0.67	NA	0	0	0	0.67	0
Flurofamide	0	0	NA	0	NA	0	0	-0.67	0	-0.67
Methyldopa	-0.67	0.67	NA	0	NA	0	0	0	0	-0.67
Clomethiazole	0	0	NA	0	NA	0	0	-0.67	-0.67	0
Remoxipride	0	0	NA	0	NA	0	0	0	0	-0.67
AF-DX 116	0	-0.67	NA	0	NA	0	0	0	0	-0.67
Levcromakalim	0	0	NA	0	NA	0	0.67	0	0	-0.67
Roquinimex	0	0.67	NA	0	NA	0	0	0	0.67	-0.67
Pinacidil monohydrate	0	0	NA	0	NA	0	0	0	0	-0.67
Remacemide hydrochloride	0	0	NA	0	NA	0	0	-0.67	0	-0.67
Ipsapirone	0	0	NA	0	NA	0	0	0	0	0.67
Nicorandil	0	-0.67	NA	0	NA	0	0	0	0	0
Capobenic acid	0	0	NA	-0.67	NA	0	0	0	0	0
Sodium zomepirac	0	0	NA	0	NA	0	0	0.67	0	0
4-Chlorophenol	0	0	NA	0	NA	0	0	0.67	0	-0.67
4-Aminobenzoic acid	0	0	NA	-0.67	NA	0	0	0	0	-0.67
Diazinon	0	0	NA	0	NA	0	0	0	-0.67	0
Aspartame	0	-0.67	NA	0	NA	0	0	0	0	0
Phenoxyacetic acid	0	0	NA	0	NA	0	0.67	0.67	0	0
Anthranilic acid	0	0	NA	0	NA	0	0	0	0	0
Hexanamide	0	0	NA	0	NA	0	0	-0.67	0	0
Acetanilide	0	0	NA	0	NA	0	0	0	0	0
4-Chloro-3-methylphenol	0	0	NA	0	NA	0	0	0	0	0
Hexanedioic acid	0	0	NA	0	NA	0	0	0	0	0
Allylthiourea	0	0	NA	0	NA	0	0	0	0	0
Salicylamide	0	0	NA	0	NA	0	0	0.67	0	0
Fenthion	0	-0.67	NA	0	NA	0	0	0	0	0.67
Cotinine	0	0	NA	-0.67	NA	0	0	0	0	0
Fenspiride hydrochloride	0	0	NA	0	NA	0	-0.67	0	0	0
Naphazoline hydrochloride	0	0	NA	0	NA	0	0	-0.67	0	0
Nomifensine maleate	0	0	NA	-0.67	NA	0	-0.67	0	0	-0.67
Bisoprolol fumarate	0	0	NA	0	NA	0	0	0	0	0
Diuron	0	0	NA	-0.67	NA	0	0	-0.67	0	0
2-Chloro-4-nitrobenzamide	0	0	NA	0	NA	0	0	0	0	0
Phenacemide	0	0.67	NA	0	NA	0	0	0	0	0
2-Aminoheptane sulfate	0	0	NA	0	NA	0	0	0	0	0
Acetarsol	0	0	NA	0	NA	0	0	0	0	0
Iproniazid sulfate	0	0	NA	0	NA	0	-0.67	-0.33	0	0
Vincamine	0	0	NA	0	NA	0	0	0	0	0

Mephenesin	0	0	NA	0	NA	0	0	0	-0.67	0
Saccharin	0	0	NA	0	NA	0	0	0	0	0
Acetyl-L-leucine	0	0	NA	0	NA	-0.67	0	-0.67	0	0
Chromocarb	0	0	NA	0	NA	0	0	0	0	0
Acexamic acid	0	0	NA	-0.67	NA	0	0	0	0	0
Pipenzolate bromide	0	0	NA	0	NA	0	0	0	0	0
Adiphenine hydrochloride	0	0	NA	-0.67	NA	0	0	-0.67	0.67	0
Diosmin	0	0	NA	0	NA	0	0	0	0	0
Pipobroman	0	0	NA	0	NA	0	0	0	0	0
Ramifenazone	0	0	NA	0	NA	0	0	0	0.67	-0.67
Metaxalone	0	0	NA	0	NA	0	0	0	0	0
Secnidazole	0	0	NA	0	NA	0.67	0	0	0	0
Clopidol	0	0	NA	0	NA	0	0	-0.67	0	0
Modafinil	0	0	NA	0	NA	0	0	0	0	0
Arbutin	0	0.67	NA	0	NA	0	0	0	0	0
2-Hydroxy-3-methylbenzoic acid	0	0.67	NA	0	NA	0	0	0	0	-0.67
Phenyl 4-aminosalicylate	0	0	NA	-0.67	NA	0	0	0	0	0.67
Salsalate	0	0	NA	0	NA	0	-0.67	0	0	0
Folic acid	0	0	NA	0	NA	0	0	0	0	0.67
Clopidogrel bisulfate	0	0.67	NA	0	NA	0	0	0	0	0
Benzathine penicillin G	0	0	NA	0	NA	0	0	0	0	0
Tramadol hydrochloride	0	0	NA	0	NA	0	0	0	0	0
Huperzine A	0	0	NA	0	NA	0	0	0	0	0
N-Acetyl-L-tyrosine	0	0	NA	0	NA	0	0	0	0	0
Phenicarbazide	0	0	NA	-0.67	NA	0	0	0	0	0
Trimetozine	0	0	NA	0	NA	0	0	0	0	0
Iodoantipyrine	0	0	NA	0	NA	0	0	0	0	0
Warfarin sodium	0	0	NA	0	NA	0	0	0	0	-0.67
Tegafur	0	0	NA	0	NA	0	0	0	0	0
Hexobarbital	0	0	NA	0	NA	0	0	-0.67	0	0
Benactyzine	0	0	NA	0	NA	0	0	0	0	0
Sorbinil	0	0.67	NA	0	NA	0	0	0	0	0
Methyl nicotinate	0	0	NA	0	NA	0	0	0	0	0
Hippuric acid	0	0	NA	0	NA	0	0.67	0	0	0
Eszopiclone	0	0	NA	0	NA	0	0	-0.67	0	0
Cephalexin	0	0	NA	0	NA	0	0	0	0	0
Pepstatin	0	0	NA	-0.67	NA	0	0	0	0	0
6,7-Dimethoxyquinazoline-2,4(1H,3H)-dione	0	0	NA	0	NA	0	0	0	0	-0.67
Tetridamine	0	0	NA	0	NA	0	0	-0.67	0	0
Loretin	0	0	NA	0	NA	0	0	0	0	-0.67

Tetrabenazine	0	0	NA	0	NA	0	0	0	0	0
4-Acetylaminophenylacetic acid	0	0	NA	0	NA	0	0	0	0	0
Lozilurea	0	0	NA	-0.67	NA	0	0	0.67	0	0
Amanozine	0	0	NA	0	NA	0	0	0	0	0
Zaleplon	0	0	NA	0	NA	0	0	0	0	0
Tizanidine hydrochloride	0	0	NA	0	NA	0	0	0	0	0
Romazarit	0	0.67	NA	0	NA	0	0	0	0	-0.67
Diamfenetide	0	0	NA	0	NA	0	0	-0.67	0	0
Ethopabate	0	0	NA	0	NA	0	0	0	-0.33	0
Sulazepam	0	0	0	0	0	0	0	0.67	0	4
Sulfasuccinamide	0	0	NA	0	NA	0	0.67	0	0.67	0
Dazmegrel	0	0	NA	0	NA	0	0	0	0.67	-0.67
Ambrisentan	0	0.67	NA	0	NA	0	0	0	0	0
Mecarbinate	0	0	NA	0	NA	0	0	0	0	0
Isocaine	0	0	NA	0	NA	0	0	-0.67	0.67	0
Ftaxilide	0	0	NA	0	NA	0	0	0	0	0
Naloxone	0	0	NA	0	NA	0	0	0	0	0
Ambenonium chloride	0	0.67	NA	0	NA	0	0	0	0.67	-0.67
Butylscopolamine bromide	0	0	NA	0	NA	0	0	0	0	0
Tiotidine	0	0	NA	0	NA	0	0	0	0	0.67
Temephos	0	0.67	NA	0	NA	0	0	0	-0.67	0.67
Olaquindox	0	0	NA	0	NA	0	0	0	0	-0.67
Hexadecanoic acid	0	0	NA	0	NA	0	0	0	0.67	0
Amiphenazole	0	0	NA	0	NA	0	0	0	0.67	0
Nicothiazone	0	0	NA	0	NA	0	0	0	0.67	-0.67
Glyoctamide	0	0	NA	-0.67	NA	0	0	-0.67	0	0
Cinromide	0	0	NA	0	NA	0	0	0	0	0
Parapropamol	0	0	NA	0	NA	0.67	0	0	0.67	0
Talinolol	0	0.67	NA	0	NA	0	0	0	0	-0.67
Dofetilide	0	0	NA	0	NA	0	0	0	-0.67	0
Eprosartan mesylate	0	0	NA	0	NA	0	0	0	0	0
Pargeverine hydrochloride	0	0	NA	-0.67	NA	0	0	0	0	0
Capecitabine	0	0	NA	0	NA	0	0	0	0	0
Etoricoxib	0	0	NA	0	NA	0	0	0	0	-0.67
Cetraxate hydrochloride	0	0	NA	0	NA	0	0	0	0.67	0
Phenprobamate	0	0	NA	0	NA	0	0	0	0	0
Propyphenazone	0	0	NA	-0.67	NA	0	0	0.67	0	0
Raltitrexed	0	0	0	0	-6.75	0	0	0	0	0
Voriconazole	0	0	NA	0	NA	0	0	0	0.67	0
Tiagabine hydrochloride	0	0	NA	0	NA	0	0	0	0.67	0
Dexrazoxane hydrochloride	0	0	NA	0	NA	0	0	0	0	0

Rufinamide	0	0	NA	0.67	NA	0	0	-0.67	0	0
Iohexol	0	0	NA	0	NA	0	-0.67	-0.67	0	0
Flamenol	0	0	NA	0	NA	0	0	0	0	0
Ethyl nicotinate	0	-0.67	NA	-0.67	NA	0	0	0	0.67	0
alpha-Lipoic acid amide	0	0	NA	0	NA	0	0	0	0	0
Pyrithioxin dihydrochloride	0	0.67	NA	-0.67	NA	0	0.67	-0.67	0	0
Alovudine	0	0	NA	0	NA	0	0	-0.67	0	0
Carsalam	0	0	NA	0	NA	0	0	0.67	0	0
Temozolomide	0	0	NA	-0.67	NA	0	0	0	0	0
Pemirolast potassium	0	0	NA	0	NA	0	0	0	0	0
Trospium chloride	0	0	NA	0	NA	0	0	0	0	0
Eprazinone dihydrochloride	0	0	NA	0	NA	0	0.67	0	0	0
Clinofibrate	0	0	NA	0	NA	0	0	0	0	0
Zofenopril	0	0	NA	0	NA	0	0	0	0	0
Vecuronium bromide	0	0.67	NA	0	NA	0	0	0	0	0
Lenalidomide	0	0	NA	0	NA	0	0	0	0	0
Pipethanate ethylbromide	0	0	NA	0	NA	0	0	0	0.67	0
Pemoline	0	0	NA	0	NA	0	0	0	0	-0.67
Ipidacrine hydrochloride hydrate	0	0	NA	0	NA	0	0	0	0	0
Lazabemide hydrochloride	0	0	NA	-0.67	NA	0	0	0	0	0
Loxoribine	0	0	NA	-0.67	NA	0	0	0	0.67	0.67
Desonide	0	0	NA	0	NA	-0.67	0	0	0	0.67
Fenobam	0	0	NA	-0.67	NA	0	0	0	0	0
Suplatast tosylate	0	0	NA	0	NA	0	0	-0.67	0.67	0
Timepidium bromide	0	0	NA	-0.67	NA	0	0	0	0	0
Nitarsone	0	0	NA	0	NA	0	0	0	0	0
Mebrofenin	0	0	0.67	0	0	0	0	0	0	0
Acetyl tributyl citrate	0	-0.67	0	-0.67	0	-0.67	0	0	1	0
Imidapril hydrochloride	0	-0.67	NA	0	NA	0	0	0	0	0
(-)- (S)-Cathinone hydrochloride	0	0	0	0	0	0	0.67	0	0.67	0
(4R,5S)-4-methyl-5-phenyl-4,5-dihydro-1,3-oxazol-2-amine	0	0	0	-0.67	0	0	0	0	0.67	-1.33
Mescaline hydrochloride	0	0	0	0	0	0	-0.67	0	0	0
3,4,5-Trimethoxyamphetamine hydrochloride	0	0	0	-0.67	0	0	-0.67	0	0	0
(+)-Metazocine fumarate (2:1)	0	0	0	0	0	0	0	0	0	0
1-Methyl-4-phenyl-4-propionoxypiperidine hydrochloride	0	0	0	0	0	-0.67	0	0	0	-1.67

N-(4-Fluorophenyl)-N-[1-(2-phenylethyl)-4-piperidyl] propanamide	0	-1	0	-0.67	0	0	0	0	1	0
N-(1-Benzyl-4-piperidyl)-N-phenylpropanamide hydrochloride	0	0	0	-0.67	0	0	0	0	1	0
1-(7-Methoxy-1,3-benzodioxol-5-yl)propan-2-amine hydrochloride	0	0	0	-0.67	0	0	0	0	0	-0.67
N-[1-(2-Hydroxy-2-phenylethyl)-4-piperidyl]-N-phenylpropanamide hydrochloride	0	0	0	0	0	0	0	0	0	0
N-Phenyl-N-[1-(2-thienyl)methyl-4-piperidyl]propanamide hydrochloride	0	0.67	0	-0.67	0	0	-1.33	0	1	0.67
N-Phenyl-N-[1-[2-(2-thienyl)ethyl]-4-piperidyl]propanamide hydrochloride (1:1)	0	0.67	0	-0.67	0	-1.33	-1.67	0	0	0
R-(-)-2,5-Dimethoxy-4-methylamphetamine hydrochloride	0	0	0	0	0	0	-2	0	1	-2
2,5-Dimethoxyamphetamine hydrochloride	0	0	0	0	0	-0.67	0	0	0	0
1-(1,3-Benzodioxol-5-yl)-N-hydroxypropan-2-amine hydrochloride	0	0.67	0	-1	0	0	0	0	0	0
(S)-(+)-N-Ethylamphetamine hydrochloride	0	0	0	-0.67	0	0	0	0	0	0
(-)-Fenfluramine hydrochloride	0	-0.67	0	-0.67	0	0	0	-0.67	0.67	-0.67
Amobarbital	0	0	0	0	0	0	-0.67	0	0	0
S-(-)-Secobarbital	0	-0.67	0	-0.67	0	0	0	0	2	-0.67
Alfentanil hydrochloride	0	0	0	-0.67	0	0	0	0	0	0
1-Phenylcyclohexylamine hydrochloride	0	0	0	-0.67	0	-1.33	0	0	0	0
1-Benzylpiperazine difumarate	0	0	0	-0.67	0	0	0	0	0	0
Dihydroisocodeine	0	0.67	0	0	0	0	0	0	0.67	-0.67
Dihydromorphine	0	0	0	-0.67	0	0	0	0	0	0.67
1-(1,3-Benzodioxol-5-yl)-N-ethylpropan-2-amine hydrochloride	0	0	0	0	0	0.67	0	0	-0.67	-0.67
1-(1,3-Benzodioxol-5-yl)-N-methylpropan-2-amine hydrochloride	-0.67	-0.67	0	-0.67	0	0	-1.33	-1	0	-2.33
Fenethylline hydrochloride	0	0	0	0	0	0	0	0	0	1.33
alpha-Methylfentanyl hydrochloride	0	0.67	0	0	0	-0.67	-1	-1	0	0

Varenicline tartrate	0	0	NA	0	NA	0	0	0	0	0
Acifluorfen-sodium	0	0	0	0	0	0	-0.67	1	0	0
Alacepril	0	0	0	-1	0	0	0	0	0	0
Cefetamet pivoxil HCl	0	0	0	0	0	0	0	0	0	0
Cefozopran hydrochloride	0	0	0	-0.67	0	0	0	-1.33	0	0
Etizolam	0	0.67	0	0	0	0	0	0	0.67	1.67
Lobenzarit sodium	0	0	0	0	0	0	-2	0	0	-3.67
Lysozyme hydrochloride	0	0	0	-0.67	0	0	0	-1.67	0	-0.33
Sulbenicillin disodium	0	0	0	0.67	0	0	0	-2	0	0
Xipamide	0	0	NA	0	NA	0	0	0	0	0
Tirofiban hydrochloride monohydrate	0	0	NA	0	NA	0	0	0	0	0
Landiolol hydrochloride	0	0	NA	0	NA	0	0	0	0	0
Biotin	0	0	NA	0	NA	0	0	0	0	0
Propamidine	0	0	0	0	0	0	0	-1	0	0
Cythioate	0	0.67	0	0	0	0	0	0	0	0
Nifenalol	0	0	NA	0	NA	0	-0.67	0	0	0
Sulfaethoxypyridazine	0	-0.67	0	0	0	0	0	0	0	0
Triamcinolone diacetate	0	0	NA	0	NA	0	0	-0.67	0	0
Fexofenadine hydrochloride	0	0	NA	0	NA	0	0	0	0.67	0
Niceritrol	0	0	NA	-0.67	NA	0	0	0	0	0
Ioxilan	0	0	NA	0	NA	0	0	0	0	0
Bamifylline Hydrochloride	0	0	NA	0	NA	0	0	-0.67	-0.67	0
Cadralazine	0	0	NA	0	NA	0	0	0	0	0
Oberadilol	-2	0	0	0	0	0	-0.67	-1.33	0	-0.67
Iomazenil	0	0	0	0	0	0	0	0	0	0
Depolipon	0	0	0	0	0	0	0	0.67	0	-2
Phenyl 11-iodo-10-undecynoate	0	0	0	0	0	0	0	0	-0.67	0
Tolvaptan	-0.67	0	0	0	0	0	-3.33	0.67	0	5
Anacolin	0	0	0	0	0	0	0	0	0	0
Bimosiamose	0	0	0	0	0	0	0	0	-0.67	0.67
Betiatide	0	0	0	0	0	-0.67	0	0	0	0
Acetylpheneturide	0	0	0	0	0	0	0	0	0	0
Blonanserin	0	0	0	-0.67	-2	-0.67	-1.33	-0.67	0	0
Dicethiamine hydrochloride	0	0.67	0	0	0	0	0	-1.67	0	0
Cloforex	0	1	0	0	0	0	0	0.67	0	0
Pelubiprofen	0	0	0	0	0	0	0	0	0	0
Risarestat	0	0	0	0	0	0	0	0	0	0
Diponium bromide	0	0	0	0	0	0	0	0	0	0
Emiglitate	0	0	0	0	0	0	0	0.67	0	0

Etomidoline	0	0	0	-0.67	0	0	0	0.67	0	-3.67
Fezatione	-1.33	1	-2.67	-0.67	0	0	0	1	0	0.67
Itasetron	0	0	0	0	0	0	0	-0.67	0	0
Pamicogrel	0	0	0	0	0	0	0	0	0	0
Esonarimod	0	0	0	0.33	0	0	0	0	0	0.67
Dalcotidine	0	0	0	-0.67	0	0	0	-1	0.67	0
Melinamide	0	0	0	0	0	-1.33	-1.33	-1.33	0	4.33
Meluadrine	0	0	0	0	0	0	0	0	0	0
Oxendolone	0	0	0	0	0	-1.33	-0.67	0	0	6.67
Penflutizide	0	0	0	-0.67	0	0	-0.67	-1	0	-0.67
Doranidazole	0	0	0.67	0	0	0	0	0	0	0
Prifinium bromide	0	0	0	0	0	0	0	0.67	0	0
Sulfamethomidine	0	0	0	0	0	0	-0.67	0	0	0
Tolindate	0	0	0	0	0	0	0	0	0	0
Acreozast	0	0	0	0	0	0	0	-1	0	0
Cefteram	0	0	0	0	0	0	0	-1	0	0
Glyclopyramide	0	0	0	0	0	0	0	0	0.67	0
Rapacuronium bromide	0	0	0	0	0	0	0	0	0	0
Temafloxacin	0	1	0	-1	0	0	-0.67	0	0	0
Propanidid	0	0	0	0	0	0	0	-0.67	0	0
Nitrefazole	0	0	0	0	0	0	0	0	0	-1.33
Clometacine	0	-0.67	0	0	0	0	-0.67	0	0.67	0
Pifoxime	0	0	0	0	0	0	0	0	0	0
4'-Piperidinylcarbonylmethoxyacetophenone	-0.67	0	0	0	0	0	0	0	0	0
Fenclofenac	0	0	0	0	0	0	0	0	0	0.67
Amineptine	0	0	0	0	1	0	0	0	0	0
Flosequinan	0	-1	0	0	0	0	0	0	0	0
Tolrestat	0	0	0	0	0	0	0	-0.67	0	-2.33
Tandospirone	0	0	0	0	0	0	0	0	0	-1.33
Phenoxypropazine	0	0	0	0	0	0	0	-0.67	0	0
Amoproxan	0	0	0	-0.67	0	0	0	-0.67	0	0
Glybuzole	0	0	0	0	0	0	0	0	0	0
Safrazine hydrochloride	0	0	0	0	0	0	0	0	0	0
Ethyl 4-nitrophenyl ethylphosphonate	0	1	0	0	0	-3.67	-0.67	-1.33	0	2.67
Mefruside	0	0	0	0	0	0	0	0	2	-0.67
Flomoxef	0	0.67	0	-0.67	0	0	0	0	0	0
Sitafloxacin hydrate	0	0	0	0	0	0	0	0	0.67	0
Aceglatone	0	0	0	0	0	0	0	-1	0	0
Sulcaine	0	0	0	0	0	0	0	0	0	-2.67

N,N-Dimethyl-2-(2-phenylacetamido)acetamide	0	-1	0	-0.67	0	0	0	-0.67	0	0
Mepranoprofen arbamel	0	0.67	0	0	0	0	-1	0	0	0
Alloclamide	0	0	0	0	0	0	0	-2.67	0	-1.33
Bucolome	0	0	0	0	0	0	0	-0.67	0	0
Fasoracetam	0	0	0	0	0	0	0	0	-0.67	0
Lanperisone hydrochloride	0	0	0	0	0	0	-0.67	0.67	0	0
5alpha-Androstan-3beta-ol	0	0	0	0	0	0	-1.33	0	0	0
Diloxanide furoate	0	0.67	NA	0	NA	0	0	0	0	0
cAMP	0	0	NA	0	NA	0	-0.67	0	0	0
4-Hydroxy-3-methoxybenzaldehyde	0	0	NA	0	NA	0	0	0	0	0
Todralazine hydrochloride	0	0.67	NA	0	NA	0	0	0	0.67	0
N-Acetyl sulfamethoxazole	0	0	NA	0	NA	0	0	0	0	0
Methsuximide	0	-2	0	-0.67	0	0	0	0	0	0
Zaltoprofen	0	0	NA	0	NA	0	0	-0.67	0	0
Mebutamate	0	0	NA	0	NA	0	0	0	0	0
Sodium 3, 4-dimethylphenyl-glyoxylate	0	0	NA	-0.67	NA	0	0	0	0	0
Cloxazolam	0	0	NA	0	NA	0	0.67	0	0	0
Docarpamine	0	0	NA	0	NA	0	0	0	0	0
Emorfazone	-0.67	0	NA	0	NA	0	0	0	0	0
Glymidine sodium	0	-0.67	NA	0	NA	0	0	0	0	0
Talbutal	0	0	NA	0	NA	0	0	0	0	-0.67
Embutramide	0	0	NA	0	NA	0	0.67	0	0	0
Arimoclomol	0	0	NA	0	NA	0	0	0	0	0
Sulfacytine	0	0	NA	0	NA	0	0	0	0	-0.67
Thenyldiamine	0	0	NA	-0.67	NA	0	0	0	0	-0.67
Isoaminile	0	0	NA	0	NA	0	0	0	0	0
Methyl 5-sulfamoyl-o-anisate	0	0	NA	0	NA	0	0.67	0	0	0
Darusentan	0	0	NA	-0.67	NA	0	0	0.67	0	0
1H-Tetrazol-1-acetic acid	0	0	NA	0	NA	0	-0.67	0	0.67	-0.67
Acephylline	0	0	NA	0	NA	0	0	0	0	0
3,5-Di-tert-butyl-4-hydroxybenzaldehyde	0	0	NA	-0.67	NA	0	0	0	0	0
6-Methoxy-2-naphthalaldehyde	0	0	NA	0	NA	0	0	0	0	0
Brofoxine	0	-0.67	NA	-0.67	NA	0	0	0	0	0
5,6-Dimethoxy-1-indanone	0	0	NA	0	NA	0	0	-0.67	0	0
Dibenzosuberone	0	0	NA	-0.67	NA	0	0	0	0	0
3,4,5-Trimethoxyphenylacetonitrile	0	0	NA	0	NA	0	0	0	0	0
Cintriamide	0	0	NA	0	NA	0	0	0	0.67	-0.67

4-(4-Acetylpiperazin-4-yl)phenol	0	0	NA	0	NA	0	0	0	0.67	0
5-Acetylsalicylamide	0	0	NA	0	NA	0	0	0	0.67	0
Dimecrotic acid	0	0	NA	0	NA	0	0	0	0	0
3,3-Tetramethyleneglutarimide	0	-0.67	NA	0	NA	0	0	0	0	0
Odiparcil	0	0	NA	0	NA	0	0	-0.67	0	-0.67
Tizoprolic acid	0	0.67	NA	0	NA	0	0	0	0	0
Methyl 5-(ethylsulfonyl)-o-anisate	0	0	NA	0	NA	0	0	0	-0.67	0
Sulfaperin	0	0	NA	0	NA	0	0	0	0	0
(2Z)-{2-[(Chloroacetyl)amino]-1,3-thiazol-4-yl}(methoxyimino)acetic acid	0	0	NA	0	NA	0	0.67	0.67	0	0
Enciprazine	0	0	NA	0	NA	0	0	0.67	0.67	0.67
Propoxate	0	0	NA	0	NA	0	0	0	0	0
Prucalopride	0	0	NA	0	NA	0	0	0	0.67	0
Cycotiamine	0	0	NA	0	NA	0	-0.67	0	0	0
Norfentanyl	0	0	NA	0	NA	0	0.67	0	0	0
Bravavir	0	0	NA	0	NA	0	0	0	-0.67	0
Viloxazine hydrochloride	0	0	NA	-0.67	NA	0	0	0	0.67	0
Furfenorex	0	0	NA	-0.67	NA	0	0.67	-0.67	0	0
Lotrafiban	0	0	NA	0	NA	0	0	0.67	0	0

SAMPLE_NAME	tox21-pr-bla-antagonist-p1-viability	tox21-pxr-p1-viability	tox21-rar-antagonist-p2-viability	tox21-ror-cho-antagonist-p1-viability	tox21-rt-viability-hek293-p1-flor.40.hr	tox21-rt-viability-hek293-p1-glo.40.hr	tox21-rt-viability-hepg2-p1-flor.40.hr	tox21-rt-viability-hepg2-p1-glo.40.hr	tox21-rxr-bla-agonist-p1-viability	tox21-sbe-bla-agonist-p1-viability
Acetazolamide	0	0	0	0	0	0	0	0	0	0
Chlormezanone	0	0	0	0	0	0	0	0	0	0
Carbamazepine	0	0	0	0	0	0	0	0	0	0
Fenclonine	0	0	0	0	0	0	0	0	0	0
Clonidine	0	0	0	0	0	0	0	0	0	0
Cyclothiazide	0	0	0	0.67	0	0	0	0	0	0
Enoximone	0	0	0	0.67	0	0	0	0	0	0
Efaroxan	0	0	0	0	0	0	0	0	0	0
Felbamate	0	0	0	0	0	0	0	0	0	0
Flumazenil	0	0	0	0	0	0	0	0	0	0
Hydroxytacrine maleate	0	0	0	0.67	0	0	0	0	0	0
Lamotrigine	0	0	0	0	0	0	0	0	0	0
Leflunomide	0	0	0	0	0	0	0	0	0	0
Molsidomine	0	0	0	0	0	0	0	0	0	0
Minoxidil	0	0	0	0	0	0	0	0	0	-0.67
Nialamide	0	0	0	0	0	0	0	0	0	0
Pentoxifylline	0	0	0	0	0	0	0	0	0	0
Piroxicam	0	0	0	0	0	0	0	0	0	0
Primidone	0	0	0	0	0	0	0	0	0	0
Sulfaphenazole	0	0	0	0	0	0	0	0	0	0
Telenzepine	0	0	0	0	0	0	0	0	0	0
1-[2-(Trifluoromethyl)phenyl]-1H-imidazole	-0.67	0	0	0	0	0	0	0	0.67	0
Tropicamide	0	0	0	0	0	0	0	0	0	0
Brimonidine	0	0.67	0	0.67	0	0	0	0	0	0
Zardaverine	0	0	0	0	0	0	0	0	0	0
Furosemide	0	0	0	0	0	0	0	0	0.67	0
Metyrapone	0	0	0	0	0	0	0	0	0	0
Sulfaguanidine	0	0	0	0	0	0	0	0	0	0
Sulfinpyrazone	0	0	0	0	0	0	0	0	0	0
Phenacetin	0	0	-0.67	0	0	0	0	0	0	0
Bendroflumethiazide	0	0	0	0	0	0	0	0	-0.67	0
Phensuximide	0	0	0	0	0	0	0	0	0	0

Benzthiazide	0	0	0	0	0	0	0	0	0	0
Azacyclonol	0	-0.67	0	0	0	0	0	0	0	0
Sulfadimethoxine	0	0	0	0	0	0	0	0	0	0
Sulfapyridine	0	0	0	0	0	0	0	0	0	0
Metronidazole	0	0	0	0	0	0	0	0	0	0
alpha-Santonin	0	-0.67	0	0	0	0	0	0	0	0
Tetrahydrozoline hydrochloride	0	0	0	0	0	0	0	0	0	0
Amylocaine hydrochloride	0	0	0	0.67	0	0	0	0	0	0
Trimethobenzamide hydrochloride	0	0	0	0	0	0	0	0	0	0
Proxyphylline	0	0	0	0	0	0	0	0	0	0
Clofibric acid	0	0	0	0	0	0	0	0	0	0
Meticrane	0	0	0	0	0	0	0	0	0	0
Midodrine hydrochloride	0	0	0	0	0	0	0	0	0	0
Bromopride	0	0	0	0	0	0	0	0	0	0
Cyclopentolate hydrochloride	0	0	0	0	0	0	0	0	0	0
Selegiline hydrochloride	0	0	0	0	0	0	0	0	0	0
Ornidazole	0	0	0	0	0	0	0	0	0	0
Tinidazole	0	0	0	0	0	0	0	0	0	0
Gliclazide	0	0	0	0	0	0	0	0	0	0
Benfotiamine	0	0	0	0	0	0	0	0	0.67	0
Etandazole	0	0	0	0	0	0	0	0	0	0
Probucol	0	0	0	0.67	0	0	0	0	0	0
Suxibuzone	0	0	0	0	0	0	0	0	0	0
Tiaprofenic acid	0	0	0	0	0	0	0	0	0	0
Etomidate	0	0	0	0	0	0	0	0	0	0
Pivampicillin	0	0	0	0	0	0	0	0	0	0
Acebutolol hydrochloride	0	0	0	0	0	0	0	0	0	0
Carteolol hydrochloride	0	0	0	0	0	0	0	0	0	0
Pipemidic acid	0	0	0	0	0	0	0	0	0	0
Meptazinol hydrochloride	0	0	0	0	0	0	0	0	0	0
Iopamidol	0	0	0	0	0	0	0	0	0	0
Rolipram	0	0	0	0	0	0	0	0	0	0
Fluticasone propionate	0	0	0	-0.67	0	0	0	0	0	0
Brinzolamide	0	0	0	0	0	0	0	0	0	0

Disopyramide	0	0	0	0	0	0	0	0	0	0
Cefotiam hydrochloride	0	0	0	0.67	0	0	0	0	0	0
Indapamide	0	0	0	0	0	0	0	0	0	0
Metoprolol tartrate	0	0	0	0	0	0	0	0	0	0
Cefmetazole sodium	0	0	0	0	0	0	0	0	0	0
Ketorolac	0	0	0	0	0	0	0	0	0	0
Dropropizine	0	0	0	0	0	0	0	0	0	0
Enalapril maleate	0	0	0	0	0	0	0	0	0	0
Hydrocortisone	0	0	0	0	0	0	0	0	0	0
Methysergide	0	0	0	0	0	0	0	0	0	0
Flurofamide	0	0	0	0	0	0	0	0	0	0
Methyldopa	0	0	0	0	0	0	0	0	0	0
Clomethiazole	0	0	0	0.67	0	0	0	0	0	0
Remoxipride	0	0	0	0	0	0	0	0	0	0
AF-DX 116	0	0	0	0	0	0	0	0	0	0
Levcromakalim	0	0	0	0	0	0	0	0	0	0
Roquinimex	0	0	0	0	0	0	0	0	0	0
Pinacidil monohydrate	-0.67	0	0	0	0	0	0	0	0	0
Remacemide hydrochloride	0	0	0	0	0	0	0	0	0	0
Ipsapirone	0	0	0	0	0	0	0	0	0	0
Nicorandil	0	0	0	0	0	0	0	0	0	0
Capobenic acid	0	0	0	0	0	0	0	0	0	0
Sodium zomepirac	0	0	0	0	0	0	0	0	0	0
4-Chlorophenol	0	0	0	0	0	0	0	0	0	0
4-Aminobenzoic acid	-0.67	0	0	0	0	0	0	0	0	0
Diazinon	0	0	0	0	0	0	0	0	0	0
Aspartame	0	0	0	0	0	0	0	0	0	0
Phenoxyacetic acid	0	0	0.67	0	0	0	0	0	0	0
Anthranilic acid	0	0	0	0	0	0	0	0	0	0
Hexanamide	0	0	0	0.67	0	0	0	0	0	0
Acetanilide	0	0	0	0	0	0	0	0	0	0
4-Chloro-3-methylphenol	0	-0.67	0	-0.67	0	0	0	0	0	0
Hexanedioic acid	0	0	0	0	0	0	0	0	0	0
Allylthiourea	0	0	0	0	0	0	0	0	0	0
Salicylamide	0	0	0	0	0	0	0	0	0	0
Fenthion	0	0	0	0	0	0	0	0	0	0

Cotinine	0	0	0	0	0	0	0	0	0	0
Fenspiride hydrochloride	0	0	0	0	0	0	0	0	0	0
Naphazoline hydrochloride	0	0	0	0	0	0	0	0	0	0
Nomifensine maleate	-0.67	0	0	0	0	0	0	0	0	0
Bisoprolol fumarate	0	0	0	0	0	0	0	0	0	0
Diuron	0	0	0	-0.67	0	0	0	0	0	0
2-Chloro-4-nitrobenzamide	0	0	0	0	0	0	0	0	0	0
Phenacemide	0	0	0	0	0	0	0	0	0	0
2-Aminoheptane sulfate	0	0	0	0	0	0	0	0	0	0
Acetarsol	0	0	0.67	0	0	0	0	0	0	0
Iproniazid sulfate	0	0	0.67	0	0	0	0	0	-0.67	0
Vincamine	0	0	0	0	0	0	0	0	0	0
Mephenesin	0	0	0	0	0	0	0	0	0	0
Saccharin	0	0	0	0	0	0	0	0	0	0
Acetyl-L-leucine	0	-0.67	0	0	0	0	0	0	0	0
Chromocarb	0	0	0	0	0	0	0	0	0	0
Acexamic acid	0	0	0	0	0	0	0	0	0	0
Pipenzolate bromide	0	0	0	0	0	0	0	0	0	0
Adiphenine hydrochloride	0	0.67	0	0	0	0	0	0	0	0
Diosmin	0	0	0	0	0	0	0	0	0	-0.67
Pipobroman	-0.67	0	0	0	0	0	0	0	0	0
Ramifenazone	0	0	0	0	0	0	0	0	0	0
Metaxalone	0	0	0	0	0	0	0	0	0	0
Secnidazole	0	0	0	0	0	0	0	0	0	0
Clopidol	0	0	0	0	0	0	0	0	0	0
Modafinil	0	0	0	0	0	0	0	0	0	0
Arbutin	0	0	0	0	0	0	0	0	0	0
2-Hydroxy-3-methylbenzoic acid	0	0	0	0	0	0	0	0	0	0
Phenyl 4-aminosalicylate	0	0	0	0	0	0	0	0	0	0
Salsalate	0	0	0	0	0	0	0	0	0	0
Folic acid	0	0	0	0	0	0	0	0	0	0
Clopidogrel bisulfate	0	0	0	0	0	0	0	0	0	0
Benzathine penicillin G	0	0	0	0	0	0	0	0	0	0

Tramadol hydrochloride	0	0	0	0	0	0	0	0	0	0
Huperzine A	0	0	0	0	0	0	0	0	0	0
N-Acetyl-L-tyrosine	0	0	0	0	0	0	0	0	0	0
Phenicarbazide	0	0	0	0	0	0	0	0	0	0
Trimetozine	0	0	0	0	0	0	0	0	0	0
Iodoantipyrine	0	0	0	0	0	0	0	0	0	0
Warfarin sodium	0	0	0	0	0	0	0	0	0	0
Tegafur	0	0	0	0	0	0	0	0	0	0
Hexobarbital	0	0	0	0	0	0	0	0	0	0
Benactyzine	0.67	0	0	0	0	0	0	0	0	0
Sorbinil	0	0	0	0	0	0	0	0	0	0
Methyl nicotinate	0	0	0	0	0	0	0	0	0	0
Hippuric acid	0	0	0	0	0	0	0	0	0	0
Eszopiclone	0	0	0	0	0	0	0	0	0	0
Cephalexin	0	0	0	0	0	0	0	0	0	0
Pepstatin	0	0	0	0	0	0	0	0	0	0
6,7-Dimethoxyquinazoline-2,4(1H,3H)-dione	0	0	0	0	0	0	0	0	0	0
Tetridamine	0	0	0	0	0	0	0	0	0	0
Loretin	0	0	0	0	0	0	0	0	0	0
Tetrabenazine	0	0	0	0	0	0	0	0	0	0
4-Acetylaminophenylacetic acid	0	0	0	0	0	0	0	0	0	0
Lozilurea	0	0	0	0	0	0	0	0	0	0
Amanozine	0	0	0	0	0	0	0	0	0	0
Zaleplon	0	0	0	0	0	0	0	0	0	0
Tizanidine hydrochloride	0	0	0	0	0	0	0	0	0	0
Romazarit	0	0	0	0	0	0	0	0	0	0
Diamfenetide	0	0	0	0	0	0	0	0	0	0
Ethopabate	0	0	0	0	0	0	0	0	0	0
Sulazepam	2	0	0	0	2	-2	0	0	0	0
Sulfasuccinamide	0	0	0	0	0	0	0	0	0	0
Dazmegrel	0	0	0	0.67	0	0	0	0	0	0
Ambrisentan	-0.67	0	0	0	0	0	0	0	0	0
Mecarbinate	0	0	0	0	0	0	0	0	0	0
Isocaine	0	0	0	0	0	0	0	0	0	0

Ftaxilide	0	0	0	0	0	0	0	0	0	0
Naloxone	0	0	0	0	0	0	0	0	0	0
Ambenonium chloride	0	0	0	0	0	0	0	0	0	0
Butylscopolamine bromide	0	0	0	0	0	0	0	0	0	0
Tiotidine	-0.67	0	0	0	0	0	0	0	0.67	0
Temephos	0	0	0	0	0	0	0	0	0	0
Olaquindox	0	0	0	0	0	0	0	0	0	0
Hexadecanoic acid	0	0	0	0	0	0	0	0	0	0
Amiphenazole	0	0	0	0	0	0	0	0	0	0
Nicothiazone	0	0	0	0	0	0	0	0	0	0
Glyoctamide	0	0	0	0	0	0	0	0	0	0
Cinromide	0	0	0	0	0	0	0	0	0	0
Parapropamol	0	0	0	0	0	0	0	0	0	0
Talinolol	0	0	0	0	0	0	0	0	-0.67	0
Dofetilide	0	0	0	0	0	0	0	0	0	0
Eprosartan mesylate	0	0	0	0.67	0	0	0	0	0	0
Pargeverine hydrochloride	0	0	0	0.67	0	0	0	0	0	0
Capecitabine	0	0	0	0	0	0	0	0	0	0
Etoricoxib	0	0	0	0	0	0	0	0	0	0
Cetraxate hydrochloride	0	0	0	0	0	0	0	0	0	0
Phenprobamate	0	0	0	0	0	0	0	0	0	0
Propyphenazone	0	0	0	0	0	0	0	0	0	0
Raltitrexed	0	0	0	0	0	-6	0	0	0	0
Voriconazole	0	0	0	0	0	0	0	0	0	0
Tiagabine hydrochloride	0	0	0	0	0	0	0	0	0	0
Dexrazoxane hydrochloride	0	0	0	0	0	0	0	0	0	0
Rufinamide	0	0	0	0	0	0	0	0	0	0
Iohexol	0	0	0	0	0	0	0	0	0	0
Flamenol	0	0	0	0	0	0	0	0	0	0
Ethyl nicotinate	0	0	0	0	0	0	0	0	0	0
alpha-Lipoic acid amide	0	0	0	0	0	0	0	0	0	0
Pyrithioxin dihydrochloride	0	0	0	0	0	0	0	0	0	-0.67
Alovudine	0	0	0	0	0	0	0	0	0	-0.67
Carsalam	0	0	0	0	0	0	0	0	0	0
Temozolomide	0	0	0	0	0	0	0	0	0	0
Pemirolast potassium	0	0	0	0	0	0	0	0	0	0

Trospium chloride	0	0	0	0	0	0	0	0	0	0
Eprazinone dihydrochloride	0	0	0	0	0	0	0	0	0	0
Clinofibrate	0	0	0	0	0	0	0	0	0	0
Zofenopril	0	0	0	0	0	0	0	0	0	0
Vecuronium bromide	0	0	0	0	0	0	0	0	0	0
Lenalidomide	0	0	0	0	0	0	0	0	0	0
Pipethanate ethylbromide	0	0	0	0	0	0	0	0	0	0
Pemoline	0	0	0	0	0	0	0	0	0	0
Ipidacrine hydrochloride hydrate	0	0	0	0	0	0	0	0	0	0
Lazabemide hydrochloride	0	0	0	0	0	0	0	0	0	0
Loxoribine	0	0	0	0	0	0	0	0	0	0
Desonide	0	0	-0.67	0	0	0	0	0	0	0
Fenobam	0	0	0	0	0	0	0	0	0	0
Suplatast tosylate	0	0	0	0	0	0	0	0	0	0
Timepidium bromide	0	0	0	0	0	0	0	0	-0.67	0
Nitarsone	0	0	0	0	0	0	0	0	0	0
Mebrofenin	0	0	0	0.67	0	0	0	0	0	0
Acetyl tributyl citrate	0	0	0	0	0	0	0	0	0	-0.67
Imidapril hydrochloride	0	0	0	0	0	0	0	0	0	0
(-)- (S)-Cathinone hydrochloride	0	0	0	0	0	0	3	0	0	0
(4R,5S)-4-methyl-5-phenyl-4,5-dihydro-1,3-oxazol-2-amine	-3	-0.67	0	0	0	0	0	0	0	0
Mescaline hydrochloride	0	0	0	0	0	0	0	0	0	0
3,4,5-Trimethoxyamphetamine hydrochloride	-1.33	0	0	0	0	0	0	0	0	0
(+)-Metazocine fumarate (2:1)	-0.67	-1.33	0	0	2	0	0	0	0	0
1-Methyl-4-phenyl-4-propionoxypiperidine hydrochloride	-0.67	0	0	0	0	0	0	0	0	0

N-(4-Fluorophenyl)-N-[1-(2-phenylethyl)-4-piperidyl] propanamide	0	-2	0	0	0	-4	2	0	0	0
N-(1-Benzyl-4-piperidyl)-N-phenylpropanamide hydrochloride	0	-1.33	0	0	2	-2	0	0	0	0
1-(7-Methoxy-1,3-benzodioxol-5-yl)propan-2-amine hydrochloride	-0.67	0	0	0	0	0	0	0	0	0
N-[1-(2-Hydroxy-2-phenylethyl)-4-piperidyl]-N-phenylpropanamide hydrochloride	0	-1.33	0	0	0	-4	0	0	0	0
N-Phenyl-N-[1-(2-thienyl)methyl-4-piperidyl]propanamide hydrochloride	0	-3.33	0	0	2	0	0	0	0	0
N-Phenyl-N-[1-[2-(2-thienyl)ethyl]-4-piperidyl]propanamide hydrochloride (1:1)	0	-2.67	0	0	0	-5	0	0	0	0
R-(-)-2,5-Dimethoxy-4-methylamphetamine hydrochloride	-0.67	-2	0	1	0	0	0	0	0	0
2,5-Dimethoxyamphetamine hydrochloride	0	0	0	0	0	0	0	0	0	0
1-(1,3-Benzodioxol-5-yl)-N-hydroxypropan-2-amine hydrochloride	0	0	0	1	0	0	0	0	0	0
(S)-(+)-N-Ethylamphetami	-1.67	0	0	3	0	0	5	0	0	0

ne hydrochloride										
(-)-Fenfluramine hydrochloride	-3	-1.33	0	2.33	2	0	0	0	0	0
Amobarbital	0	0	0	1	0	0	0	0	0	0
S-(-)-Secobarbital	-0.67	0	0	0	0	0	0	0	0	0
Alfentanil hydrochloride	-0.67	-0.67	0	1.33	0	0	0	0	0	0
1-Phenylcyclohexylamine hydrochloride	0	-3.67	0	0	0	0	0	0	0	0
1-Benzylpiperazine difumarate	0	0	0	1.67	0	0	0	0	0	0
Dihydroisocodeine	-2	0	0	1	0	0	0	0	0	0
Dihydromorphine	-0.67	0	0	0	0	0	0	0	0	0
1-(1,3-Benzodioxol-5-yl)-N-ethylpropan-2-amine hydrochloride	-0.67	0	0	0.67	0	0	0	0	0	0
1-(1,3-Benzodioxol-5-yl)-N-methylpropan-2-amine hydrochloride	-4.67	-2.33	0	1.67	0	0	0	0	-1.33	0
Fenethylline hydrochloride	0	0	0	0	0	-5	0	0	0	0
alpha-Methylfentanyl hydrochloride	0	0	0	0	0	0	0	0	0.67	0
Varenicline tartrate	0	0	0	0	0	0	0	0	0	0
Acifluorfen-sodium	0	0	0	0.67	0	0	0	0	0	0
Alacepril	0	0	0	0.67	0	0	0	0	0	0
Cefetamet pivoxil HCl	0	0	0	3	0	0	0	0	0	0
Cefozopran hydrochloride	0	0	0	2.33	0	0	0	0	0	0
Etizolam	0	0	0	0	2	0	0	0	1	0
Lobenzarit sodium	-6.67	-1.67	0	0	0	0	0	0	0	0
Lysozyme hydrochloride	0	0	0	0.67	0	0	0	0	0	0
Sulbenicillin disodium	-0.67	0	0	1.67	0	0	0	0	0	0
Xipamide	0	0	0	0	0	0	0	0	0	0

Tirofiban hydrochloride monohydrate	0	0	0	0	0	0	0	0	0	0
Landiolol hydrochloride	0	0	0	0	0	0	0	0	0	0
Biotin	0	0	0	0	0	0	0	0	0	0
Propamidine	0	0	0	0	0	0	0	0	0	0
Cythioate	0	0	0	0	0	0	-2	0	0	0
Nifenalol	-0.67	0	0	0	0	0	0	0	0	0
Sulfaethoxypyridazine	0	0	0	0	0	-2	0	0	0	0
Triamcinolone diacetate	0	0	0	0	0	0	0	0	0	0
Fexofenadine hydrochloride	0	0	0	0	0	0	0	0	0	0
Niceritrol	0	-0.67	0	0	0	0	0	0	0	0
Ioxilan	0	0	0	0	0	0	0	0	0	0
Bamifylline Hydrochloride	0	0	0	0	0	0	0	0	0	0
Cadralazine	0	0	0	0	0	0	0	0	0	0
Oberadilol	0	-1.33	0	0	0	-4	0	0	-1.33	0
Iomazenil	0	0	0	0	0	0	0	0	0	0
Depolipon	0	0	0	2.67	0	0	0	0	-1.33	0
Phenyl 11-iodo-10-undecynoate	0	0	0	0	0	-8	0	-9	0	0
Tolvaptan	0	0	0	0	0	-5	0	0	0	0
Anacolin	-0.67	0	0	0	0	0	0	-2	-1.33	0
Bimosiamose	0	0	0	0	0	0	0	0	0	0
Betiatide	0	0	0	0	0	0	0	0	0	0
Acetylpheneturide	0	0	0	0	0	0	0	0	0	0
Blonanserin	0	0	0	0	0	0	5	0	-3	0
Dicethiamine hydrochloride	0	0	0	0	0	0	0	0	0	0
Cloforex	0	0	0	0	2	0	0	0	0	0
Pelubiprofen	0	0	0	0	0	0	0	0	0	0
Risarestat	0	0	0	0	0	0	2	0	0	0
Diponium bromide	0	0	0	0	0	-5	0	0	-1.33	0
Emiglitate	0	0	0	0	0	0	0	0	0	0
Etomidoline	-0.67	-2	0	0	0	-9	0	0	0	0
Fezatione	0.67	0	0	0	0	0	0	0	-0.67	-2.67
Itasetron	0	0	0	0	0	0	0	0	0	0
Pamicogrel	0	0	0	0	0	0	0	-2	-2.67	0
Esonarimod	0	0	0	0	0	0	0	0	0	0

Dalcotidine	0	0	0	0	0	0	7	0	0	0
Melinamide	4	0	0	0	0	0	0	0	0	0
Meluadrine	0	0	0	0	0	0	0	0	0	0
Oxendolone	0.67	-0.67	0	0	2	-5	5	0	0	0
Penflutizide	0	0	0	0	0	0	0	0	0	0
Doranidazole	0	0	0	0	0	0	0	0	0	0
Prifinium bromide	0	0	0	0	0	-2	0	0	0	0
Sulfamethomidine	0	0	0	-0.67	0	0	0	0	0	0
Tolindate	-2	-0.67	0	0	0	0	0	0	-2	0
Acreozast	0	0	0	0	0	0	0	0	0	0
Cefteram	0	0	0	0	0	0	0	0	1	0
Glyclopyramide	0	0	0	0	0	0	0	0	1	0
Rapacuronium bromide	0	0	0	1.67	0	0	0	0	-2	0
Temafloxacin	0	0	0	5	0	0	0	0	0	0
Propanidid	0.67	0	0	0	-2	0	0	0	0	0
Nitrefazole	-3	0	0	0	0	0	0	0	0	0
Clometacine	0	0	0	0	0	0	0	0	0	0
Pifoxime	0	0	0	0	0	0	0	0	0	0
4'-Piperidinylcarbonylmethoxyacetophenone	0	0	0	0	0	0	0	0	0	0
Fenclofenac	0	0	0	0	0	0	0	0	0	0
Amineptine	0	0	0	0	0	0	0	0	0	0
Flosequinan	0	0	0	0	0	0	0	0	0	0
Tolrestat	0	0	0	0	0	0	0	0	0	0
Tandospirone	0	0	0	0	-2	0	2	0	-0.67	0
Phenoxypropazine	0	0	0	0	0	0	0	0	0	0
Amoproxan	0	0	0	0	0	0	0	0	0	0
Glybuzole	0	0	0	1	0	0	0	0	0	0
Safrazine hydrochloride	0	0	0	-0.67	0	0	0	0	0	0
Ethyl 4-nitrophenyl ethylphosphonate	-0.67	0	0	0	0	0	0	0	0	-2
Mefruside	0	0	0	0	0	0	0	0	0	0
Flomoxef	0	0	0	0	0	0	0	0	0	0
Sitafloxacin hydrate	0	0	0	6	0	0	0	0	0	0
Aceglatone	0	0	0.67	0	0	0	7	0	0	0

Sulcaine	0	0	0	0	0	0	0	0	-0.67	0
N,N-Dimethyl-2-(2-phenylacetamido)acetamide	0	0	-0.67	0	0	0	0	0	0	0
Mepranoprofen arbamel	0	0	0	0	0	0	0	0	-0.67	0
Alloclamide	-0.67	-0.67	0	2	2	0	0	0	-0.67	0
Bucolome	0	0	0	0	0	0	0	0	0	0
Fasoracetam	0	0	0	0	0	0	0	0	0	0
Lanperisone hydrochloride	0	0	0	2.67	2	-2	7	-2	-2	0
5alpha-Androstan-3beta-ol	-1	0	-0.67	0	0	0	0	0	-3	0
Diloxanide furoate	0	0	0	0	0	0	0	0	0	0
cAMP	0	0	0	0	0	0	0	0	0	0
4-Hydroxy-3-methoxybenzaldehyde	0	0	0	0	0	0	0	0	0	0
Todralazine hydrochloride	0	0	0	0	0	0	0	0	0	0
N-Acetyl sulfamethoxazole	0	0	0	0	0	0	0	0	0	0
Methsuximide	0	0	0	0	0	0	0	0	0	0
Zaltoprofen	0	0	0	0	0	0	0	0	0	0
Mebutamate	0	0	0	0	0	0	0	0	0	0
Sodium 3, 4-dimethylphenyl-glyoxylate	0	0	0	0	0	0	0	0	0	0
Cloxazolam	0	0	0	0	0	0	0	0	0	0
Docarpamine	0	0	0	0	0	0	0	0	0	0
Emorfazone	0	0	0	0	0	0	0	0	0	0
Glymidine sodium	0	0	0	0	0	0	0	0	0	0
Talbutal	0	0	0	0	0	0	0	0	0	0
Embutramide	0	0	0	0	0	0	0	0	0	0
Arimoclomol	0	0	0	0	0	0	0	0	0	0
Sulfacytine	0	0	0	0	0	0	0	0	0	0
Thenyldiamine	0	0	0	0	0	0	0	0	0	0
Isoaminile	0	0	0	0	0	0	0	0	0	0
Methyl 5-sulfamoyl-o-anisate	0	0	0	0	0	0	0	0	0	0
Darusentan	-0.67	0	0	0	0	0	0	0	0	0

1H-Tetrazol-1-acetic acid	0	0	0	0	0	0	0	0	0	0
Acephylline	0	0	0	0	0	0	0	0	0	0
3,5-Di-tert-butyl-4-hydroxybenzaldehyde	0	0	0	0	0	0	0	0	-0.67	0
6-Methoxy-2-naphthalaldehyde	0	0	0	0	0	0	0	0	0	0
Brofoxine	0	0	0	0	0	0	0	0	0	0
5,6-Dimethoxy-1-indanone	0	0	0	0	0	0	0	0	0	0
Dibenzosuberone	0	0	0	0	0	0	0	0	0	0
3,4,5-Trimethoxyphenylacetonitrile	0	0	0	0	0	0	0	0	0	0
Cintriamide	0	0	0	0	0	0	0	0	0	0
4-(4-Acetylpiperazin-4-yl)phenol	0	0	0	0	0	0	0	0	0	0
5-Acetylsalicylamide	0	0	0	0	0	0	0	0	0	0
Dimecrotic acid	0	0	0	0	0	0	0	0	0	0
3,3-Tetramethyleneglutarimide	0	0	0	0	0	0	0	0	0	0
Odiparcil	0	0	0	0	0	0	0	0	0	0
Tizoprolic acid	0	0	0	0	0	0	0	0	0	0
Methyl 5-(ethylsulfonyl)-o-anisate	0	0	0	0.67	0	0	0	0	0	0
Sulfaperin	0	0	0	-0.67	0	0	0	0	0	0
(2Z)-{2-[(Chloroacetyl)amino]-1,3-thiazol-4-yl}(methoxyimino)acetic acid	0	0	0	0	0	0	0	0	0	0
Enciprazine	0	0	0	0	0	0	0	0	0	0
Propoxate	0	0	0	0	0	0	0	0	0	0
Prucalopride	0	0	0	0	0	0	0	0	0	0
Cycotiamine	0	0	0	0	0	0	0	0	0	0
Norfentanyl	0.67	0	0	0	0	0	0	0	0	0
Bravavir	0	0	0	0	0	0	0	0	0	0
Viloxazine hydrochloride	0	0	0	0	0	0	0	0	0	0
Furfenorex	0	0	0	0	0	0	0	0	0	0

Lotrafiban	0	0	0	0	0	0	0	0	0	0

SAMPLE_NAME	tox21-sbe-bla-antagonist-p1-viability	tox21-shh-3t3-gli3-agonist-p1-viability	tox21-shh-3t3-gli3-antagonist-p1-viability	tox21-vdr-bla-agonist-p1-viability	tox21-vdr-bla-antagonist-p1-viability
Acetazolamide	0	0	0	0	0
Chlormezanone	0	0	0	-0.67	0
Carbamazepine	0	0	0	0	0
Fenclonine	0	0	0	0	0
Clonidine	0	0	0	0	0
Cyclothiazide	0	0	0	0	0
Enoximone	-0.67	0	0	0	0
Efaroxan	0	0	0	0	0
Felbamate	0	0	0	0	-0.67
Flumazenil	0	0	0	0	0
Hydroxytacrine maleate	0	0	0	0	0
Lamotrigine	0	0	0	0	0
Leflunomide	0	0	0	0	0
Molsidomine	0	0	0	0	0
Minoxidil	0	0	0	0	0
Nialamide	0	0	0	-0.67	0
Pentoxifylline	0	0	0	0	0
Piroxicam	0	0	0	0	0
Primidone	0	0	0	0	0
Sulfaphenazole	0	0	0	0	0.67
Telenzepine	0	0	0	0	0
1-[2-(Trifluoromethyl)phenyl]-1H-imidazole	0	0	0	0	-0.67
Tropicamide	0	0	0	0	0
Brimonidine	0	0	0	0	0
Zardaverine	0	0	0	0	0
Furosemide	0	0	0	0	0.67
Metyrapone	0	0	0	0	0
Sulfaguanidine	0	0	0	0	0
Sulfinpyrazone	0	0	0	0	0
Phenacetin	0	0	0	0	0
Bendroflumethiazide	0	0	0	0	0
Phensuximide	0	0	0	0	0
Benzthiazide	0	0	0	0	0
Azacyclonol	0	-0.67	-0.67	-0.67	0
Sulfadimethoxine	0	0	0	0	0
Sulfapyridine	0	0	0	0	0

Metronidazole	0	0	0	0	0
alpha-Santonin	0	0	0	0	0
Tetrahydrozoline hydrochloride	0	0	0	0	-0.67
Amylocaine hydrochloride	0	0	0	0	-0.67
Trimethobenzamide hydrochloride	0	0	0	0	0
Proxyphylline	0	0	0	0	0
Clofibric acid	0	0	0	0	0
Meticrane	0	0	0	0	0
Midodrine hydrochloride	0	0	0	0	0
Bromopride	0	0	0	0	0
Cyclopentolate hydrochloride	0	0	0	-0.67	0
Selegiline hydrochloride	0	0	0	0	0
Ornidazole	0	0	0	0	0
Tinidazole	0	0	0	0	0
Gliclazide	0	0	0	0	0
Benfotiamine	0	0	0	0	0
Etandazole	0	0	0	0	0
Probucol	0	0	0	0	0
Suxibuzone	0	0	0	0	0
Tiaprofenic acid	0	0	0	0	0
Etomidate	0	0	0	0	0
Pivampicillin	0	0	0	0	0
Acebutolol hydrochloride	0	0	0	0.67	0
Carteolol hydrochloride	0	0	0	0	0
Pipemidic acid	0	0	0	0	0
Meptazinol hydrochloride	0	0	-0.67	0	0
Iopamidol	0	0	0	0	0
Rolipram	0	0	0	0	0
Fluticasone propionate	0	0	0	0	0
Brinzolamide	0	0	0	0	0
Disopyramide	0	0	0	0	0
Cefotiam hydrochloride	0	0	0	0	0
Indapamide	0	0	0	0	0
Metoprolol tartrate	0	0	0	0	0
Cefmetazole sodium	0	-0.67	0	0	0
Ketorolac	0	0	-0.67	0	0
Dropropizine	0	0	0	0.67	0.67
Enalapril maleate	0	0	0	0	-0.67
Hydrocortisone	0	0	0	0	0
Methysergide	0	0	0	0	0
Flurofamide	0	0	0	0	0

Methyldopa	0	0	0	0	0
Clomethiazole	0	0	0	0	0
Remoxipride	0	0	-0.67	0	0
AF-DX 116	0	0	0	0	0
Levcromakalim	0	0	0	0	0
Roquinimex	0	0	0	0	0
Pinacidil monohydrate	0	0	0	0	0
Remacemide hydrochloride	0	0	0	0	0
Ipsapirone	0	0	0	0	0
Nicorandil	0	0	0	0	0
Capobenic acid	0	0	0	0.67	0
Sodium zomepirac	0	0	0	0	0
4-Chlorophenol	0	0	0	0	-0.67
4-Aminobenzoic acid	0	0	0	0	0
Diazinon	0	0	0	0	0
Aspartame	0	0	0	0	0
Phenoxyacetic acid	0	0	0	0	0
Anthranilic acid	0	0	0	0	0
Hexanamide	0	0	0	0	0
Acetanilide	0	0	0	0.67	0.67
4-Chloro-3-methylphenol	0	0	0	0	0
Hexanedioic acid	0	0	0	0	0
Allylthiourea	0	0	0	0	0
Salicylamide	0	0	0	0	0
Fenthion	0	0	0	0	0
Cotinine	0	0	0	0	0
Fenspiride hydrochloride	0	0	0	0	0
Naphazoline hydrochloride	0	0	0	-0.67	0
Nomifensine maleate	0	0	0	0	0
Bisoprolol fumarate	0	0	0	0	0
Diuron	0	0	0	0	0
2-Chloro-4-nitrobenzamide	0	0	0	0	0
Phenacemide	0	0	0	0	0
2-Aminoheptane sulfate	0	0	0	0	0
Acetarsol	0	0	0	0	0
Iproniazid sulfate	0	0	0	0	0
Vincamine	0	0	0	0	0
Mephenesin	0	0	0	0	0
Saccharin	0	0	0	0	0
Acetyl-L-leucine	0	0	0	0	0
Chromocarb	0	0	0	0	0

Acexamic acid	0	0	0	0	0.67
Pipenzolate bromide	0	0	0	0	0
Adiphenine hydrochloride	0	0	0	0	0
Diosmin	0	0	0	0	0
Pipobroman	0	0	0	0	0
Ramifenazone	0	0	0	0	0
Metaxalone	0	0	0	0	0
Secnidazole	0	0	0	0	0
Clopidol	0	0	0	0	0
Modafinil	0	0	0	0	0
Arbutin	0	0	0	0	0
2-Hydroxy-3-methylbenzoic acid	0	0	0	0	-0.67
Phenyl 4-aminosalicylate	0	0	0	0	0
Salsalate	0	0	0	0	0
Folic acid	0	0	0	0	0.67
Clopidogrel bisulfate	0	0	0	0	0
Benzathine penicillin G	0	0	0	0	0
Tramadol hydrochloride	0	0	0	0	0
Huperzine A	0	0	0	0	0
N-Acetyl-L-tyrosine	0	0	0	0.67	0
Phenicarbazide	0	0	0	0	0
Trimetozine	0	0	0	0	0
Iodoantipyrine	0	0	0	0	0
Warfarin sodium	0	0	-0.67	0	0.67
Tegafur	0	0	0	0	0
Hexobarbital	0	0	0	0	0
Benactyzine	0	0	0	0.67	0
Sorbinil	0	0	0	0	0
Methyl nicotinate	0	0	0	0	0
Hippuric acid	0	0	0	0	0
Eszopiclone	0	-0.67	0	0	-0.67
Cephalexin	0	0	0	0	0
Pepstatin	0	0	0	0	0
6,7-Dimethoxyquinazoline-2,4(1H,3H)-dione	-0.67	0	0	0	0
Tetridamine	0	0	0	0	0
Loretin	0	0	0	0	0
Tetrabenazine	0	0	0	0	0
4-Acetylaminophenylacetic acid	0	0	0	0	0
Lozilurea	0	0	0	0	0
Amanozine	0	0	0	0	0
Zaleplon	0	0	0	0	0

Tizanidine hydrochloride	0	0	0	0	0
Romazarit	0	0	0	0	0
Diamfenetide	0	0	0	0	0
Ethopabate	0	0	0	0	0
Sulazepam	0	0	0	0	0
Sulfasuccinamide	0	0	0	0	0
Dazmegrel	0	0	0	0	0
Ambrisentan	0	0	0	0	0
Mecarbinate	0	0	0	0	-0.67
Isocaine	0	0	0	0	0
Ftaxilide	0	0	0	0	0
Naloxone	0	0	0	0	0
Ambenonium chloride	0	0	0	0	0
Butylscopolamine bromide	0	0	-0.67	0	0
Tiotidine	0	0	0	0	0
Temephos	0	0	0	0	-0.67
Olaquindox	0	0	0	0	0
Hexadecanoic acid	0	0	0	0	-0.67
Amiphenazole	0	0	0	0	0
Nicothiazone	0	0	0	0	0
Glyoctamide	0	0	0	0	0
Cinromide	0	0	0	0	0
Parapropamol	0	0	0	0.67	0
Talinolol	0	-0.67	0	0	0.67
Dofetilide	0	0	0	0	0
Eprosartan mesylate	0	0	0	0	0
Pargeverine hydrochloride	-0.67	0	0	0	0
Capecitabine	0	0	0	0	0
Etoricoxib	0	0	0	0	0
Cetraxate hydrochloride	0	0	0	0	0
Phenprobamate	0	0	0	0	0
Propyphenazone	0	0	0	0	-0.67
Raltitrexed	0	0	0	0	0
Voriconazole	0	0	0	0	0
Tiagabine hydrochloride	0	0	0	0	0
Dexrazoxane hydrochloride	0	0	0	0	0
Rufinamide	0	0	0	0	0
Iohexol	0	0	0	0	0
Flamenol	0	0	0	0	0
Ethyl nicotinate	0	0	0	0	0
alpha-Lipoic acid amide	0	0	0	0	0

Pyrithioxin dihydrochloride	0	0	0	0	0
Alovudine	0	0	0	0	0
Carsalam	0	0	0	0	0.67
Temozolomide	0	0	0	0	0.67
Pemirolast potassium	0	0	0	0	0
Trospium chloride	0	0	0	0	0
Eprazinone dihydrochloride	0	0	0	0	0
Clinofibrate	0	0	0	0	0
Zofenopril	0	0	0	0	0
Vecuronium bromide	0	0	0	0	0
Lenalidomide	0	0	0	0	0
Pipethanate ethylbromide	0	0	0	0	0
Pemoline	-0.67	0	0	0	0
Ipidacrine hydrochloride hydrate	0	0	0	0	0
Lazabemide hydrochloride	0	0	0	0	0
Loxoribine	0	0	0	-0.67	0
Desonide	0	0	0	0	0.67
Fenobam	0	0	0	0	0.67
Suplatast tosylate	0	0	0	0	0
Timepidium bromide	0	0	0	0	0
Nitarsone	0.67	0	0	0	0
Mebrofenin	0	0	0	0	0.67
Acetyl tributyl citrate	0	0	0	0	0
Imidapril hydrochloride	0	0	0	0.67	0
(-)- (S)-Cathinone hydrochloride	0	0	-0.67	0	0
(4R,5S)-4-methyl-5-phenyl-4,5-dihydro-1,3-oxazol-2-amine	0	0	-0.67	0	0
Mescaline hydrochloride	0	-0.67	-2	0	0
3,4,5-Trimethoxyamphetamine hydrochloride	0	-3	-0.67	0	0
(+)-Metazocine fumarate (2:1)	0	0	0	0	0
1-Methyl-4-phenyl-4-propionoxypiperidine hydrochloride	0	0	-0.67	0	0
N-(4-Fluorophenyl)-N-[1-(2-phenylethyl)-4-piperidyl] propanamide	0	0	0	0	0
N-(1-Benzyl-4-piperidyl)-N-phenylpropanamide hydrochloride	0	0	-0.67	0	0
1-(7-Methoxy-1,3-benzodioxol-5-yl)propan-2-amine hydrochloride	0	0	0	0	0
N-[1-(2-Hydroxy-2-phenylethyl)-4-piperidyl]-N-phenylpropanamide hydrochloride	0	0	0	0	0
N-Phenyl-N-[1-(2-thienyl)methyl-4-piperidyl]propanamide hydrochloride	0	0	-2.33	-0.67	0

N-Phenyl-N-[1-[2-(2-thienyl)ethyl]-4-piperidyl]propanamide hydrochloride (1:1)	0	0	0	0	0
R-(-)-2,5-Dimethoxy-4-methylamphetamine hydrochloride	0	0	0	0	0
2,5-Dimethoxyamphetamine hydrochloride	0	0	0	0	0
1-(1,3-Benzodioxol-5-yl)-N-hydroxypropan-2-amine hydrochloride	0	0	0	0.67	0
(S)-(+)-N-Ethylamphetamine hydrochloride	0	0	0	0	0
(-)-Fenfluramine hydrochloride	0	-0.67	-2.33	-0.67	2
Amobarbital	0	0	-0.67	0	0
S-(-)-Secobarbital	0	0	-3	-0.67	0
Alfentanil hydrochloride	0	0	-1.33	0.67	0
1-Phenylcyclohexylamine hydrochloride	0	-6.33	-6.33	0	0
1-Benzylpiperazine difumarate	0	0	-0.67	0	1.67
Dihydroisocodeine	0	-0.67	-3	0	0.67
Dihydromorphine	0	-0.67	-4.67	-0.67	0
1-(1,3-Benzodioxol-5-yl)-N-ethylpropan-2-amine hydrochloride	0	0	0	0	0
1-(1,3-Benzodioxol-5-yl)-N-methylpropan-2-amine hydrochloride	0	-2	-3.67	-1.33	0.67
Fenethylline hydrochloride	0	-4.33	-6	0	0
alpha-Methylfentanyl hydrochloride	0	0	-0.67	0	0
Varenicline tartrate	0	0	0	0	0
Acifluorfen-sodium	0	0	0	-1.33	-0.67
Alacepril	0	0	0	0	0
Cefetamet pivoxil HCl	-0.67	0	0	-0.67	0
Cefozopran hydrochloride	0	0	0	0	0
Etizolam	0	-0.67	-2	0.67	0
Lobenzarit sodium	0	-0.67	-3	-2	0.67
Lysozyme hydrochloride	0	0	0	0	0
Sulbenicillin disodium	0	0	0	0	0.67
Xipamide	0	0	0	0	0
Tirofiban hydrochloride monohydrate	0	0	0	0	0
Landiolol hydrochloride	0	0	0	0	0
Biotin	0	0	0	0	0
Propamidine	0	0	0	0	0
Cythioate	0	0	0	0	0
Nifenalol	0	0	0	0.67	0
Sulfaethoxypyridazine	0	0	0	0	0
Triamcinolone diacetate	0	0	0	0	0
Fexofenadine hydrochloride	0	0	0	0	0
Niceritrol	0	0	0	0	0

Ioxilan	0	0	0	0	0
Bamifylline Hydrochloride	0	0	0	0	0
Cadralazine	0	0	0	0	0
Oberadilol	0	0	0	0	0
Iomazenil	0	0	0	0	0
Depolipon	0	0	0	0	0
Phenyl 11-iodo-10-undecynoate	0	0	0	0	0
Tolvaptan	0	0	0	0.67	0
Anacolin	0	0	0	0	0
Bimosiamose	0	0	0	0	0
Betiatide	0	0	0	0	0
Acetylpheneturide	0	0	0	0	0
Blonanserin	0	0	0	-0.67	0
Dicethiamine hydrochloride	0	0	0	0	-1.33
Cloforex	0	0	0	0	0
Pelubiprofen	0	0	0	0	0
Risarestat	0	0	0	0.67	0
Diponium bromide	0	0	0	0	0
Emiglitate	0	0	0	0	-0.67
Etomidoline	0	0	0	0	0
Fezatione	-0.67	0	0	0.67	0
Itasetron	0	0	0	0	0
Pamicogrel	0	0	0	0	0
Esonarimod	0	0	0	0	0
Dalcotidine	0	0	0	0	0
Melinamide	0	-1.33	-3	0	0
Meluadrine	0	0	0	0	-0.67
Oxendolone	0	0	0	0	0
Penflutizide	0	0	0	0	0
Doranidazole	0	0	0	0	0
Prifinium bromide	0	0	0	0.67	-1.67
Sulfamethomidine	0	0	0	0	0
Tolindate	0	0	0	0	-0.67
Acreozast	0	0	0	0	0
Cefteram	0	0.67	0	0	-2
Glyclopyramide	0	0	0	0	0
Rapacuronium bromide	0	0	0	0	0
Temafloxacin	0	0	0	0	0
Propanidid	0	0	0	0	0
Nitrefazole	0	0	0	0	0
Clometacine	0	-0.67	0	0	0

Pifoxime	0	0	0	0	0
4'-Piperidinylcarbonylmethoxyacetophenone	0	0	0	0	0
Fenclofenac	0	0	0	0	0.67
Amineptine	0	0	0	0	0
Flosequinan	0	0	0	0	0.67
Tolrestat	0	0	0	0	0
Tandospirone	-0.67	0	0	0	-1.33
Phenoxypropazine	0	0	0	0	-0.67
Amoproxan	0	0	0	0	0
Glybuzole	0	0	0	0	0
Safrazine hydrochloride	0	0	0	0	0
Ethyl 4-nitrophenyl ethylphosphonate	0	-0.67	0	0	0
Mefruside	0	0	0	0	0.67
Flomoxef	0	0	0	0	0
Sitafloxacin hydrate	0	0	0	0	0
Aceglatone	0	0	0	0	0
Sulcaine	0	0	0	0	0
N,N-Dimethyl-2-(2-phenylacetamido)acetamide	0	0	0	0	0
Mepranoprofen arbamel	0	0	0	0	0
Alloclamide	0	0	0	0	0
Bucolome	0	0	0	0	0
Fasoracetam	0	0	0	0	0
Lanperisone hydrochloride	0	0	0	-0.67	0
5alpha-Androstan-3beta-ol	0	0	0	-0.67	0
Diloxanide furoate	0	0	0	0	0
cAMP	0	0	0	0	0
4-Hydroxy-3-methoxybenzaldehyde	0	0	0	0	0
Todralazine hydrochloride	0	0	0	0	0
N-Acetyl sulfamethoxazole	0	0	0	0	0
Methsuximide	0	0	0	0	0
Zaltoprofen	0	0	0	0	0
Mebutamate	0	0	0	0	0
Sodium 3, 4-dimethylphenyl-glyoxylate	0	0	0	0	0
Cloxazolam	0	0	0	0	0
Docarpamine	0	0	0	0	0
Emorfazone	0	0	0	0	0
Glymidine sodium	0	0	0	0	0
Talbutal	0	0	0	0	0
Embutramide	0	0	0	0	0
Arimoclomol	0	0	0	0	0

Sulfacytine	0	0	0	0	0
Thenyldiamine	0	0	0	0	0
Isoaminile	0	0	0	0	0
Methyl 5-sulfamoyl-o-anisate	0	0	0	0	0
Darusentan	0	0	0	0	0
1H-Tetrazol-1-acetic acid	0	0	0	0	0
Acephylline	0	0	0	0	0
3,5-Di-tert-butyl-4-hydroxybenzaldehyde	0	0	0	0	0
6-Methoxy-2-naphthalaldehyde	0	0	0	0	0
Brofoxine	0	0	0	0	0
5,6-Dimethoxy-1-indanone	0	0	0	0	0
Dibenzosuberone	0	0	0	0	0
3,4,5-Trimethoxyphenylacetonitrile	0	0	0	0	0
Cintriamide	0	0	0	0	0
4-(4-Acetylpiperazin-4-yl)phenol	0	0	0	0	0
5-Acetylsalicylamide	0	0	0	0.67	0
Dimecrotic acid	0	0	0	0	0
3,3-Tetramethyleneglutarimide	0	0	0	0	0
Odiparcil	0	0	0	0	0
Tizoprolic acid	0	0	0	0	0
Methyl 5-(ethylsulfonyl)-o-anisate	0	0	0	0	0
Sulfaperin	0	0	0	0	0.67
(2Z)-{2-[(Chloroacetyl)amino]-1,3-thiazol-4-yl}(methoxyimino)acetic acid	-0.67	0	0	0	0
Enciprazine	0	0	0	0	0
Propoxate	0	0	-0.67	0	0.67
Prucalopride	0	0	0	0	-0.67
Cycotiamine	0	0	0	0	0.67
Norfentanyl	0	0	0	0	0.67
Bravavir	0	0	0	0	0
Viloxazine hydrochloride	0	0	0	0	0
Furfenorex	0	0	0	0	0
Lotrafiban	0	0	0	0	0

Table S5. Chemical features significantly enriched in AIADs or NCPCs based on the ToxPrint fingerprints

ToxPrint	Category	Subcategory	p avelue enriched in NCPCs	p avelue enriched in AIADs
bond:X[any_!C]_halide_inorganic	Functional Groups	Other	3.08E-13	NA
ring:aromatic_phenyl	Aromatic Systems	Single-Ring Aromatics	8.14E-04	NA
ring:aromatic_benzene	Aromatic Systems	Single-Ring Aromatics	4.71E-03	NA
atom:element_metal_group_I_II	Uncategorized	Other	5.81E-03	NA
bond:quatN_generic	Uncategorized	Other	8.73E-03	NA
bond:C(=O)N_carboxamide_generic	Functional Groups	Amide	1.10E-02	NA
bond:NC=O_aminocarbonyl_generic	Functional Groups	Carbonyl	1.36E-02	NA
group:ligand_path_4_bidentate_ethylenediamine	Functional Groups	Amine	1.56E-02	NA
ring:hetero_[6]_Z_1_2-	Uncategorized	Other	4.88E-02	NA
bond:C#N_nitrile	Uncategorized	Other	NA	2.74E-02
bond:C(=O)N_carbamate	Uncategorized	Other	NA	4.52E-05
bond:C(=O)N_carboxamide_(NHR)	Functional Groups	Amide	NA	5.71E-03
bond:C(=O)O_carboxylicEster_alkyl	Functional Groups	Carboxyl	NA	3.34E-02
bond:C=N_carboxamidine_generic	Uncategorized	Other	NA	1.05E-02
bond:C=N_guanidine_generic	Uncategorized	Other	NA	3.42E-02
bond:CC(=O)C_ketone_aliphatic_generic	Uncategorized	Other	NA	1.07E-03
bond:CC(=O)C_ketone_alkene_cyclic_2-en-1-one_generic	Uncategorized	Other	NA	2.99E-02
bond:COC_ether_aliphatic	Functional Groups	Ether	NA	1.32E-10
bond:COC_ether_alkenyl	Functional Groups	Ether	NA	2.17E-02
bond:COH_alcohol_aliphatic_generic	Uncategorized	Other	NA	1.56E-07
bond:COH_alcohol_alkene	Uncategorized	Other	NA	1.34E-02
bond:COH_alcohol_alkene_cyclic	Uncategorized	Other	NA	1.34E-02
bond:COH_alcohol_allyl	Uncategorized	Other	NA	3.04E-03
bond:COH_alcohol_diol_(1_3-)	Uncategorized	Other	NA	1.66E-04

bond:COH_alcohol_generic	Uncategorized	Other	NA	6.75E-07
bond:COH_alcohol_pri-alkyl	Uncategorized	Other	NA	3.71E-05
bond:COH_alcohol_sec-alkyl	Uncategorized	Other	NA	1.32E-04
bond:COH_alcohol_ter-alkyl	Uncategorized	Other	NA	2.95E-02
bond:CX_halide_alkyl-F_trifluoro_(1_1_1-)	Functional Groups	Halide	NA	3.42E-02
bond:CX_halide_alkyl-X_dihalo_(1_1-)	Functional Groups	Other	NA	2.50E-02
bond:CX_halide_alkyl-X_generic	Functional Groups	Other	NA	2.29E-02
bond:CX_halide_alkyl-X_trihalo_(1_1_1-)	Functional Groups	Other	NA	3.42E-02
bond:CX_halide_aromatic-X_dihalo_benzene_(1_2-)	Functional Groups	Other	NA	2.55E-02
bond:CX_halide_aromatic-X_dihalo_benzene_(1_4-)	Functional Groups	Other	NA	3.69E-02
bond:CX_halide_aromatic-X_generic	Functional Groups	Other	NA	5.19E-03
bond:CX_halide_generic-X_dihalo_(1_2-)	Functional Groups	Other	NA	2.55E-02
bond:P(=O)N_phosphoramide_monoamidophosphate	Functional Groups	Amide	NA	7.00E-03
bond:P~N_generic	Uncategorized	Other	NA	4.77E-02
bond:P=O_phosphate	Functional Groups	Phosphate	NA	4.76E-02
bond:P=O_phosphonate	Uncategorized	Other	NA	4.77E-02
bond:P=O_phosphorus_oxo	Uncategorized	Other	NA	2.15E-05
bond:PC_phosphorus_organo_generic	Uncategorized	Other	NA	6.40E-03
chain:alkaneBranch_isopropyl_C3	Uncategorized	Other	NA	7.34E-04
chain:alkaneBranch_t-butyl_C4	Uncategorized	Other	NA	5.53E-04
chain:alkaneCyclic_ethyl_C2_(connect_noZ)	Uncategorized	Other	NA	5.87E-05
chain:alkaneCyclic_hexyl_C6	Uncategorized	Other	NA	1.19E-03
chain:alkaneCyclic_pentyl_C5	Uncategorized	Other	NA	9.48E-03
chain:alkaneCyclic_propyl_C3	Uncategorized	Other	NA	1.19E-11
chain:alkaneLinear_ethyl_C2_(connect_noZ_CN=4)	Uncategorized	Other	NA	5.25E-06
chain:alkeneCyclic_diene_cyclohexene	Uncategorized	Other	NA	2.33E-02
chain:alkeneCyclic_ethene_C_(connect_noZ)	Uncategorized	Other	NA	2.61E-05

chain:alkeneCyclic_ethene_generic	Uncategorized	Other	NA	1.03E-08
chain:alkyne_ethyne_generic	Uncategorized	Other	NA	3.85E-02
group:aminoAcid_aminoAcid_generic	Uncategorized	Other	NA	5.43E-03
group:aminoAcid_valine	Uncategorized	Other	NA	1.65E-04
group:carbohydrate_hexopyranose_generic	Uncategorized	Other	NA	2.17E-02
group:carbohydrate_ketohexose	Uncategorized	Other	NA	3.42E-02
group:carbohydrate_pentofuranose_2-deoxy	Uncategorized	Other	NA	1.32E-03
group:ligand_path_4_bidentate_aminoacetaldehyde	Uncategorized	Other	NA	4.21E-03
group:ligand_path_4_tridentate	Uncategorized	Other	NA	1.61E-02
group:ligand_path_5_bidentate_aminopropanal	Uncategorized	Other	NA	2.45E-02
group:ligand_path_5_bidentate_propandiamine	Functional Groups	Amine	NA	1.65E-04
group:nucleobase_adenine	Nucleobase/Nucleotide Analogs	Purine	NA	4.77E-02
group:nucleobase_cytosine	Nucleobase/Nucleotide Analogs	Pyrimidine	NA	1.79E-03
group:nucleobase_guanine	Nucleobase/Nucleotide Analogs	Purine	NA	3.69E-02
group:nucleobase_hypoxanthine	Nucleobase/Nucleotide Analogs	Purine	NA	3.69E-02
group:nucleobase_thymine	Nucleobase/Nucleotide Analogs	Pyrimidine	NA	4.76E-02
group:nucleobase_uracil	Nucleobase/Nucleotide Analogs	Pyrimidine	NA	1.03E-03
ring:aromatic_biphenyl	Aromatic Systems	Single-Ring Aromatics	NA	3.16E-06
ring:hetero_[5]_N_imidazole	Heterocyclic Systems	Five-Membered Rings	NA	3.08E-04
ring:hetero_[5]_N_pyrrole	Uncategorized	Other	NA	1.41E-02
ring:hetero_[5]_N_pyrrole_generic	Uncategorized	Other	NA	1.65E-03
ring:hetero_[5]_N_triazole_(1_2_4-)	Uncategorized	Other	NA	3.54E-03
ring:hetero_[5]_N_triazole_(1_3_4-)	Uncategorized	Other	NA	1.79E-03
ring:hetero_[5]_O_oxolane	Uncategorized	Other	NA	1.01E-06
ring:hetero_[5]_Z_1-Z	Uncategorized	Other	NA	4.31E-07
ring:hetero_[5]_Z_1_2_4_1_3_4-Z	Uncategorized	Other	NA	5.47E-03
ring:hetero_[5]_Z_1_3-Z	Uncategorized	Other	NA	1.74E-03

ring:hetero_[5_6]_N_benzimidazole	Heterocyclic Systems	Five-Membered Rings	NA	1.06E-04
ring:hetero_[5_6]_N_indole	Heterocyclic Systems	Fused Systems	NA	4.45E-02
ring:hetero_[5_6]_N_purine	Nucleobase/Nucleotide Analogs	Purine	NA	6.40E-03
ring:hetero_[5_6]_Z_generic	Uncategorized	Other	NA	7.46E-09
ring:hetero_[6]_N_diazine_(1_3-)_generic	Uncategorized	Other	NA	4.12E-06
ring:hetero_[6]_N_pyridine	Heterocyclic Systems	Six-Membered Rings	NA	1.27E-02
ring:hetero_[6]_N_pyrimidine	Nucleobase/Nucleotide Analogs	Pyrimidine	NA	8.54E-03
ring:hetero_[6]_N_pyrimidine_2_4-dione	Nucleobase/Nucleotide Analogs	Pyrimidine	NA	1.76E-02
ring:hetero_[6]_O_pyran_generic	Uncategorized	Other	NA	2.58E-03
ring:hetero_[6]_Z_1-	Uncategorized	Other	NA	5.71E-03
ring:hetero_[6]_Z_1_3-	Uncategorized	Other	NA	1.04E-05
ring:hetero_[6]_Z_1_4-	Uncategorized	Other	NA	7.44E-03
ring:hetero_[6]_Z_generic	Uncategorized	Other	NA	3.51E-07
ring:hetero_[6_6]_N_quinoline	Heterocyclic Systems	Six-Membered Rings	NA	1.79E-03
ring:hetero_[6_6]_Z_generic	Uncategorized	Other	NA	3.70E-03
ring:hetero_[7]_O_oxepin	Uncategorized	Other	NA	3.69E-02

Table S6. Approved and investigational drugs evaluated in CPE and/or PP assays

Sample ID	Sample Name	Primary MOA	endpoint
NCGC00345173-01	GSK-525768	Bromodomain-Containing Protein 4 (Brd4) inhibitor	1
NCGC00160217-03	Sophocarpine	HERG Inhibitor	0
NCGC00164574-14	Erlotinib	Epidermal Growth Factor Receptor inhibitor	1
NCGC00168463-06	Bretylium tosylate	norepinephrine secretion Inhibitor	0
NCGC00250387-06	KU-0060648	DNA-Dependent Protein Kinase inhibitor	1
NCGC00347946-02	IDH-C227	Isocitrate Dehydrogenase [NADP] cytoplasmic inhibitor	0
NCGC00274078-01	Fludrocortisone		0
NCGC00346684-02	AG-1024	Insulin-like growth factor 1 receptor inhibitor	1
NCGC00387310-02	A-196	Histone-lysine N-methyltransferase KMT5C inhibitor	0
NCGC00159511-04	Butenafine	Trichophyton mentagrophytes Inhibitor	0
NCGC00485487-01	Samotolisib	Phosphatidylinositol 3-Kinase alpha isoform inhibitor	1
NCGC00262907-01	Bilastine		0
NCGC00249924-01	Ibuprofen piconol		0
NCGC00016272-05	Guanethidine sulfate		0
NCGC00091195-08	Hexachlorophene	Glucose-6-phosphate dehydrogenase Inhibitor	1
NCGC00015958-18	SP-600125	Mitogen-Activated protein kinase 8 (JNK) inhibitor	0
NCGC00181343-03	Ivabradine hydrochloride	HCN [I(f)] Blocker	0
NCGC00162143-03	Clodronate	ADP/ATP translocase 3 Inhibitor	0
NCGC00095322-05	Coenzyme Q10	mitochondrial electron transport	0
NCGC00263872-02	SPIRAMYCIN	Bacterial 70S ribosome Binding Agent	0
NCGC00264003-02	(2R,3R)-2,3-Dimercaptosuccinic acid	Lead Chelating Agent	0
NCGC00178027-08	VALSARTAN	Neprilysin Inhibitor	0
NCGC00345804-10	ARRY-162	Dual Specificity Mitogen-Activated Protein Kinase Kinase 1 inhibitor	0
NCGC00167537-02	Donepezil hydrochloride	Acetylcholinesterase Inhibitor	0
NCGC00185756-04	Estramustine	DNA Alkylating Drug	0
NCGC00510199-01	Bimiralisib	Phosphatidylinositol 3-Kinase alpha isoform inhibitor	1
NCGC00386432-07	BLZ945	Colony Stimulating Factor 1 Receptor inhibitor	0
NCGC00346461-02	NVP-AEW541	Insulin-like growth factor 1 receptor inhibitor	0
NCGC00015848-09	Tranylcypromine hydrochloride	Amine oxidase A inhibitor	0
NCGC00023902-09	4-Androstene-3,17-dione	Cytochrome P450 19A1 Substrate	0
NCGC00496851-01			0
NCGC00016497-13	Ethionamide	Cell Wall Biosynthesis Inhibitor	0
NCGC00263135-02	Piraxostat	Xanthine Dehydrogenase/Oxidase inhibitor	0
NCGC00178864-04	Ethambutol HCl		0
NCGC00167462-03	Acamprosate Calcium	NMDA Receptor antagonist	0

NCGC00159448-05	4-Methoxycinnamic acid		0
NCGC00183867-02	Aloin		0
NCGC00387879-02	Afuresertib (GSK2110183)	AKT serine/threonine kinase inhibitor	0
NCGC00263875-02	SELAMECTIN		0
NCGC00510491-01	Elafibranor	Peroxisome proliferator-activated receptor alpha agonist	0
NCGC00249933-01	Nitrogen mustard N-oxide		0
NCGC00167507-13	Lapatinib	Epidermal Growth Factor Receptor inhibitor	0
NCGC00018250-06	Sulconazole nitrate	Lanosterol 14-alpha demethylase Inhibitor	0
NCGC00017245-10	Ellagic acid	Aldose Reductase inhibitor	0
NCGC00095175-08	Cefditoren (Pivoxil)	Cephalosporin Antibiotic	0
NCGC00091238-03	2,2'-Dithiobisbenzothiazole		0
NCGC00188430-01	Moperone	Serotonin 2a (5-HT2a) receptor Inverse Agonist	0
NCGC00016032-11	alpha-Lipoic acid		0
NCGC00181135-02	Pentamycin		0
NCGC00179351-03	Quinethazone	Thiazide-sensitive sodium-chloride cotransporter Inhibitor	0
NCGC00178054-06	Clarithromycin	Macrolide Antibiotic	0
NCGC00274084-01	DL-Isoleucine		0
NCGC00380310-01		<MOA Unknown> | Class: Terpenoid | Genus: N/A | Family: N/A | Species: N/A	1
NCGC00346669-05	TAK-875	Free Fatty Acid Receptor 1 agonist	0
NCGC00510350-01	MPT-0B098	Tubulin polymerization inhibitor	1
NCGC00390670-01	BAY-356	Bromodomain-Containing Protein 4 (Brd4) inhibitor	0
NCGC00346706-07	VU0357121	Glutamate receptor metabotropic 5 Positive allosteric modulator	0
NCGC00390611-02	GSK2110183 (hydrochloride)		0
NCGC00378606-02	Ipragliflozin	Sodium/glucose cotransporter 2 Inhibitor	0
NCGC00345451-03	A-966492	Poly [ADP-ribose] polymerase 1 inhibitor	0
NCGC00263129-31	Palbociclib	Cyclin-Dependent Kinase 6 inhibitor	1
NCGC00508871-01	HM-30181	Multidrug resistance protein 1 inhibitor	0
NCGC00165856-03	Orlistat	Pancreatic Triacylglycerol Lipase inhibitor	0
NCGC00183365-02	ORNITHINE HYDROCHLORIDE	G-protein coupled receptor family C group 6 member A Agonist	0
NCGC00378669-02	Boldenone Undecylenate		0
NCGC00025355-12	Paroxetine	5-HT Reuptake Inhibitor	0
NCGC00345793-01	EMD-1214063	Hepatocyte Growth Factor Receptor inhibitor	0
NCGC00510033-02	Distigmine Bromide	Acetylcholinesterase Inhibitor	0
NCGC00390693-06	ELR-510444	Tubulin polymerization inhibitor	0
NCGC00346620-06	GSK-256066	Phosphodiesterase 4 inhibitor	0
NCGC00167527-03	Grepafloxacin hydrochloride	Topoisomerase IV Inhibitor	0
NCGC00483924-02	Brigatinib	ALK Tyrosine Kinase Receptor inhibitor	0
NCGC00263016-01	SA-13353	Transient receptor potential cation channel subfamily V member 1 agonist	0

NCGC00091687-01	Benzene sulfonic acid		0
NCGC00345819-01	Estetrol	Selective Estrogen Receptor modulator (SERM)	0
NCGC00025202-12	Mevastatin	HMG-CoA Reductase inhibitor	0
NCGC00180997-01	Ethylphenylacetylurea		0
NCGC00345492-06	PCI-34051	Histone deacetylase 8, class I inhibitor	1
NCGC00189077-01	Methsuximide	Voltage-gated T-type calcium channel Inhibitor	0
NCGC00178737-05	Trimeprazine tartrate	Histamine H1 receptor Antagonist	0
NCGC00263215-04	Torin-1	mTOR Complex 1 (mTORC1) inhibitor	0
NCGC00183012-01	Penflutizide	Thiazide-sensitive sodium-chloride cotransporter Inhibitor	0
NCGC00090752-04	Fenretinide	Retinoic Acid Receptor gamma agonist	0
NCGC00261968-01	Bufetolol	Adrenergic receptor beta Antagonist	0
NCGC00016388-04	Noscapine	Tubulin Polymerization Inhibitor	0
NCGC00346525-03	Aurora A Inhibitor I	Aurora kinase A inhibitor	0
NCGC00022662-05	Dexamethasone acetate	Mineralocorticoid receptor Agonist	0
NCGC00016612-14	Sulfadoxine	Dihydropteroate Synthase (DHPS) Inhibitor	0
NCGC00021277-04	Hydrocortisone acetate		0
NCGC00480828-04	ORY-1001	Lysine-Specific Histone Demethylase 1A inhibitor	0
NCGC00182710-02	Flurbiprofen axetil		0
NCGC00378978-02	XL-228	Insulin-like growth factor 1 receptor inhibitor	1
NCGC00095161-05	Rebamipide	Antioxidant	0
NCGC00346672-02	ARRY-380	Receptor tyrosine-protein kinase NEU (HER2; erbB2) inhibitor	0
NCGC00390366-01	Bosutinib(SKI-606)		1
NCGC00522020-01	Butropium Bromide	Muscarinic acetylcholine receptor Antagonist	0
NCGC00015730-11	Neostigmine bromide	Acetylcholinesterase Inhibitor	0
NCGC00091051-04	4-Aminobenzoic acid	Carbonic anhydrase II Inhibitor	0
NCGC00250393-01	CAP-232	Somatostatin srif2B (sst4) Agonist	0
NCGC00179364-03	Ioversol		0
NCGC00509991-01	Pipequaline	Gamma-aminobutyric acid receptor subunit alpha-1 modulator	0
NCGC00261971-01	Mazaticol		0
NCGC00386242-02	Cilengitide	Integrin alpha-V/beta-5 Antagonist	0
NCGC00521944-01			1
NCGC00180693-03		<MOA Unknown> | Class: Triterpen | Genus: Achras | Family: Sapotaceae | Species: sapota	1
NCGC00167493-04	Manidipine	Calcium Channel Blocker	0
NCGC00179456-04	Glycopyrrolate bromide		0
NCGC00182984-01	Tolindate		0
NCGC00016463-08	Cytisine	Nicotinic alpha3beta4 Receptor agonist	0
NCGC00522011-01	ER 819762	Prostaglandin EP4 Receptor antagonist	0
NCGC00253606-01	Mexazolam		0
NCGC00179546-05	Bambuterol hydrochloride	Butyrylcholinesterase Inhibitor	0

NCGC00183103-01	Tocophersolan		0
NCGC00095165-08	Carmofur	Thymidylate Synthase inhibitor	0
NCGC00263918-08	Epirubicin hydrochloride	DNA Topoisomerase II inhibitor	0
NCGC00522107-03	Relugolix	Gonadotropin Releasing Hormone Receptor antagonist	0
NCGC00498437-01	AZD-0156	ATM Kinase inhibitor	1
NCGC00346594-02	Oleanolic acid	Secretory Phospholipase A2 (sPLA2) inhibitor	0
NCGC00167514-02	Ethylestrenol	Androgen Receptor Agonist	0
NCGC00263897-02	PREDNISOLONE HEMISUCCINATE	0	
NCGC00346496-05	WZ-3146	Epidermal Growth Factor Receptor (Thr790Met Mutant) inhibitor	1
NCGC00090808-04	Amphotericin B	Anti-fungal	0
NCGC00249912-01	Floctafenine	Prostaglandin G/H synthase 2 Inhibitor	0
NCGC00249926-01	Pirmenol		0
NCGC00093985-11	Metolazone	Carbonic Anhydrase 7 inhibitor	0
NCGC00250379-21	CT-99021	Glycogen Synthase Kinase 3 (GSK-3) inhibitor	0
NCGC00015798-11	Dexpropranolol	Adrenergic receptor beta Antagonist	0
NCGC00475734-01	Patidegib	Smoothened Receptor antagonist	0
NCGC00185766-04	Esmolol hydrochloride	sodium ion transport Inhibitor	0
NCGC00181745-03	Bimatoprost	prostaglandin	0
NCGC00387475-01	GSK-2194069	Fatty Acid Synthase inhibitor	1
NCGC00242499-02	Pim 1 Inhibitor 2	Serine/threonine-protein kinase pim-1 inhibitor	0
NCGC00263227-02	BAY-41-8543	Soluble Guanylate Cyclase (sGC) activator	1
NCGC00164561-11	Zanamivir		0
NCGC00159507-02	Alclofenac	Cyclooxygenase-2 Inhibitor	0
NCGC00164528-04	GLYCERYL LAURATE	Toxic shock syndrome toxin-1 Inhibitor	0
NCGC00346888-03	UK-383367	Procollagen C-Endopeptidase inhibitor	0
NCGC00346617-03	AZD-5438	Cyclin-Dependent Kinase 1 inhibitor	1
NCGC00350511-01	PRP003-073	Succinate Receptor 1 inhibitor	0
NCGC00485481-02	ELN-441958	B1 Bradykinin Receptor antagonist	0
NCGC00509914-01	NMS-P118	Poly [ADP-ribose] polymerase 1 inhibitor	0
NCGC00386257-02	Aclidinium (Bromide)	Muscarinic acetylcholine receptor M3 Antagonist	0
NCGC00390666-01	Opicapone	Catechol O-methyltransferase Inhibitor	0
NCGC00380981-01		<MOA Unknown> | Class: N/A | Genus: N/A | Family: N/A | Species: N/A	1
NCGC00166055-04	SODIUM NITROPRUSSIDE		0
NCGC00402345-02	Decernotinib	Tyrosine-protein kinase JAK3 inhibitor	0
NCGC00017256-17	Galanthamine	Acetylcholinesterase Inhibitors	0
NCGC00018243-09	Sulfamethazine	Bacterial dihydropteroate synthase Inhibitor	0
NCGC00179611-05	DIHYDROSTREPTOMYCIN [5mM]	Large-conductance mechanosensitive channel Modulator	0

NCGC00160397-04	Irsogladine maleate	Muscarinic acetylcholine receptor M1 antagonist	0
NCGC00090762-11	Rifabutin	Cytochrome P450 3a4 Substrate	0
NCGC00015937-07	Spermidine	Autophagy Regulator	0
NCGC00181021-02	Bufogenin	Sodium/potassium-transporting ATPase Inhibitor	1
NCGC00165759-02	BAY-41-2272	Soluble Guanylate Cyclase (sGC) activator	0
NCGC00347068-02	KUC103669N	Rab7 GTPase inhibitor	0
NCGC00159497-02	Allylestrenol	Progesterone receptor Agonist	0
NCGC00166226-02	4-Propylbenzoic acid		0
NCGC00174736-02	Uridine triacetate	Thymidylate Synthase inhibitor	0
NCGC00160391-09	Auranofin	Thioredoxin Reductase inhibitor	1
NCGC00163548-03	Echinomycin	DNA Intercalating Drug	0
NCGC00370925-02	PF-4800567	Casein Kinase 1 epsilon inhibitor	0
NCGC00016332-04	Riboflavin	Vitamin	0
NCGC00024379-17	Emetine dihydrochloride hydrate	Anti-parasitic	1
NCGC00013556-15	Liothyronine Sodium	Thyroid hormone receptor beta-1 Agonist	0
NCGC00016000-18	Disulfiram	Aldehyde Dehydrogenase-2 inhibitor	1
NCGC00183010-01	Pelubiprofen	Prostaglandin G/H synthase 2 Inhibitor	0
NCGC00167510-02	Doripenem	Carbapenem Antibiotic	0
NCGC00346640-05	PF-04929113	Heat Shock Protein 90 (Hsp90) inhibitor	0
NCGC00023282-21	AMPICILLIN SODIUM		0
NCGC00016260-13	Ethacrynic acid	Sodium/potassium-transporting ATPase subunit alpha-1 Inhibitor	0
NCGC00025225-03	SCH-79797	Proteinase-activated receptor 1 antagonist	1
NCGC00347943-09	GSK-J1	Lysine-Specific Demethylase 6A inhibitor	0
NCGC00263555-02	Spautin-1	Ubiquitin Carboxy-Terminal Hydrolase 10/13 inhibitor	1
NCGC00164071-03	Isopropyl tetradecanoic acid		0
NCGC00165864-04	Pipamperone dihydrochloride	Serotonin 2c (5-HT2c) receptor Antagonist	0
NCGC00015971-05	Succinylcholine chloride	Muscle-type nicotinic acetylcholine receptor Agonist	0
NCGC00510691-01	3-BDO	Autophagy inhibitor	0
NCGC00507962-02	GSK2795039	NADPH Oxidase 2 inhibitor	0
NCGC00238452-05	Enzastaurin	Phosphatidylinositol 3-Kinase alpha isoform inhibitor	0
NCGC00386634-01	Compound 120	HIV Protease Inhibitors	0
NCGC00387870-04	GSK2141795	AKT serine/threonine kinase inhibitor	1
NCGC00181356-02	Oxapium iodide		0
NCGC00522465-01	GNF-6231	Protein Serine O-Palmitoleoyltransferase Porcupine inhibitor	0
NCGC00345784-11	PF-3758309	Serine/threonine-protein kinase PAK 4 inhibitor	0
NCGC00522534-01	Quinagolide (hydrochloride)		0
NCGC00160617-03	Thiamine pyrophosphate		0

NCGC00164520-02	L-Threonine		0
NCGC00179366-03	Florfenicol		0
NCGC00346568-05	GSK-1292263A	Glucose-Dependent Insulinotropic Receptor agonist	0
NCGC00475729-01	BMS-906024	gamma-Secretase inhibitor	0
NCGC00475726-01	Atuveciclib	Cyclin-Dependent Kinase 9 inhibitor	1
NCGC00387832-02	17-AAG (KOS953)	Heat Shock Protein 90 (hsp90) Inhibitor	1
NCGC00346738-05	Pregabalin	L-type Ca2+ channel Blocker	0
NCGC00346546-04	MK-3207	Calcitonin gene-related peptide type 1 Receptor antagonist	0
NCGC00346630-05	MP-10	Phosphodiesterase 10A inhibitor	0
NCGC00346502-04	Dienogest	Progesterone Receptor agonist	0
NCGC00094047-10	Ivermectin	Glutamate-gated Chloride Channel Activator	1
NCGC00167480-02	N-Hydroxymethylsuccinimide		0
NCGC00384196-02	UM 171	Hematopoietic Stem Cell Differentiation Agonist	1
NCGC00015774-06	Oxatomide	Histamine H1 receptor Antagonist	0
NCGC00481603-01	Fruquintinib	Vascular Endothelial Growth Factor Receptor 3 (VEGFR-3) inhibitor	0
NCGC00025092-02	GR-159897	Tachykinin receptor 2 antagonist	0
NCGC00379089-04	Genz-644282	DNA Topoisomerase I inhibitor	1
NCGC00249110-03	Sucralose		0
NCGC00179520-04	Metrizamide		0
NCGC00249909-01	Fenbutrazate	Monoamine transporters; Norepinephrine & dopamine Agonist	0
NCGC00378983-02	PF-04457845	Fatty Acid Amide Hydrolase inhibitor	0
NCGC00178839-08	NAD+		0
NCGC00346545-08	Telaprevir (VX-950)	HCV NS3 NS4A Protease Inhibitor	0
NCGC00263968-02	ANCITABINE HYDROCHLORIDE	antiviral	0
NCGC00386324-08	LDN-212854	ALK2 inhibitor	0
NCGC00179860-02		Staphylococcus aureus Inhibitor	1
NCGC00384220-02	AMG-925 (FLX925)	Cyclin-Dependent Kinase 4 inhibitor	0
NCGC00346696-07	PF-5274857	Smoothened Receptor antagonist	0
NCGC00164622-16	Voriconazole	Lanosterol 14alpha-demethylase Inhibitor	0
NCGC00167507-04	Lapatinib	Epidermal Growth Factor Receptor inhibitor	0
NCGC00507855-03	SGI-7079	AXL Kinase inhibitor	1
NCGC00015563-13	Imipramine	Serum paraoxonase/arylesterase 1 Inhibitor	0
NCGC00167454-02	Pilsicainide	Sodium channel alpha subunit Blocker	0
NCGC00090868-06	HALOTHANE	GABA-A receptor; anion channel Positive Allosteric Modulator	0
NCGC00402376-02	CEP-37440	ALK Inhibitor	0
NCGC00347372-02		<MOA Unknown> | Class: Steroid | Genus: Asparagus | Family: Liliaceae | Species: racemosus	1
NCGC00263163-11	SGX-523	Hepatocyte Growth Factor Receptor inhibitor	0

NCGC00487176-01	EAA-090		0
NCGC00345841-02	S-99	Mitogen-Activated Protein Kinase Kinase Kinase 5 inhibitor	0
NCGC00348365-01	INDOCYANINE GREEN		0
NCGC00263229-03	GSK-837149A	Fatty Acid Synthase inhibitor	0
NCGC00014993-12	Nonanedioic acid	reactive oxygen species biosynthetic process Inhibitor	0
NCGC00167451-04	Halobetasol Propionate		0
NCGC00263124-09	Nutlin-3	E3 Ubiquitin-Protein Ligase Mdm2 (Hdm2) inhibitor	0
NCGC00421916-03	PF-05175157	Acetyl-CoA Carboxylase inhibitor	0
NCGC00480912-02	GSK-484	Protein-Arginine Deiminase Type-4 inhibitor	0
NCGC00016637-10	Diloxanide furoate		0
NCGC00511352-01	BVT-14225	11-beta-Hydroxysteroid Dehydrogenase Type 1 (11beta-HSD1) Inhibitor	0
NCGC00017325-06	Kojic acid	Tyrosinase Inhibitor	0
NCGC00013109-03	Phenol red	amyloid fibril formation Inhibitor	0
NCGC00386433-04	CH-5183284	Fibroblast Growth Factor Receptor 3 inhibitor	0
NCGC00185754-01	Terlipressin	Vasopressin V1a Receptor Partial Agonist	0
NCGC00165898-11	SD-208	TGF-beta receptor type-1 inhibitor	0
NCGC00387039-04	Taselisib (GDC-0032)	Phosphatidylinositol 3-Kinase beta isoform inhibitor	1
NCGC00386236-02	TAK 438	Potassium-transporting ATPase Blocker	0
NCGC00263234-01	IDH-001	Isocitrate Dehydrogenase [NADP] cytoplasmic inhibitor	0
NCGC00016511-08	Chlorotrianisene	Estrogen receptor Modulator	0
NCGC00091533-10	Ethinylestradiol	Estrogen Receptor agonist	0
NCGC00386271-02	CNX-2006	Epidermal Growth Factor Receptor (Thr790Met Mutant) inhibitor	1
NCGC00507868-01	CPI-637	Histone Acetyltransferase p300 inhibitor	0
NCGC00271596-03	Exemestane	Aromatase inhibitor	0
NCGC00346881-02	APR-246	Mutant p53 activator	0
NCGC00016065-11	Tropicamide	Muscarinic acetylcholine receptor M3 Antagonist	0
NCGC00263553-13	StemRegenin 1	Aryl Hydrocarbon Receptor antagonist	0
NCGC00093828-12	PD-169316	Mitogen-Activated Protein Kinase p38 inhibitor	0
NCGC00015966-10	Levosulpiride	Dopamine D2 Antagonist	0
NCGC00167464-02	Hydrocortisone Sodium Phosphate	0	
NCGC00167467-03	Vecuronium bromide	Neuronal acetylcholine receptor subunit alpha-2 antagonist	0
NCGC00032387-14	TG-003	Dual Specificity protein kinase CLK4 inhibitor	0
NCGC00181352-01	Aspoxicillin		0
NCGC00181107-01	Arotinolol hydrochloride	Adrenergic receptor beta Antagonist	0
NCGC00509947-01	NVP-FGF-401	Fibroblast Growth Factor Receptor 4 inhibitor	0
NCGC00159478-04	Benzoxiquine		0

NCGC00346491-08	Varespladib	Secretory Phospholipase A2 (sPLA2) inhibitor	0
NCGC00142384-03	Cholic Acid	Bile acid receptor FXR Agonist	0
NCGC00390733-04	PF-06447475	Leucine-Rich Repeat Kinase 2 inhibitor	0
NCGC00390660-02	LDN-214117	ALK2 inhibitor	0
NCGC00346840-06	Tubacin	Histone deacetylase 6, class IIB inhibitor	0
NCGC00167432-05	Fosinopril??Na	Angiotensin-converting enzyme Inhibitor	0
NCGC00185750-01	Astromicin	Bacterial 70S ribosome Binding Agent	0
NCGC00250401-11	Flavopiridol	Cyclin-Dependent Kinase 1 inhibitor	1
NCGC00384607-01		<MOA Unknown> | Class: Terpenoid | Genus: N/A | Family: N/A | Species: N/A	1
NCGC00165727-10	AKT Inhibitor VIII	AKT serine/threonine kinase inhibitor	0
NCGC00389454-01	IMEXON	Ribonucleoside-Diphosphate Reductase Inhibitors	0
NCGC00378614-02	Flumatinib mesylate	Bcr-Abl Kinase inhibitor	0
NCGC00346590-02	Artenimol	Anti-malarial	1
NCGC00043225-04	Disodium 2-hydroxybutanedioate		0
NCGC00181743-02	Lypressin	Vasopressin receptor Agonist	0
NCGC00481108-02	BI-9564	Bromodomain-Containing Protein 9 (Brd9) inhibitor	0
NCGC00345847-02	JNJ-38158471	Proto-oncogene tyrosine-protein kinase receptor Ret inhibitor	0
NCGC00344523-01	L-(-)-Sorbose		0
NCGC00091470-05	Isoeugenol	Tyrosyl-DNA phosphodiesterase 2 Inhibitor	0
NCGC00167457-02	Gestrinone	Progesterone receptor Agonist	0
NCGC00522018-01	Echothiophate Iodide	Cholinesterases; ACHE & BCHE Inhibitor	0
NCGC00166076-07	Arbutin	Free Radical Scavenger	0
NCGC00253567-01	Talbutal	GABA-A receptor; anion channel Positive Allosteric Modulator	0
NCGC00378796-01	Tacalcitol	Vitamin D receptor Agonist	0
NCGC00160570-01	Butonate		0
NCGC00351478-06	A-366	Histone-lysine N-methyltransferase EHMT2 inhibitor	0
NCGC00183656-11	Elesclomol (STA-4783)	Heat Shock Protein 70 inducer	0
NCGC00488701-03	BAY-876	Solute carrier family 2, facilitated glucose transporter member 1 inhibitor	0
NCGC00351608-02	Erastin	Cystine/Glutamate Transporter inhibitor	0
NCGC00510200-01	MRT-83	Smoothened Receptor antagonist	0
NCGC00263183-02	MK-1775	Wee1-like protein kinase inhibitor	1
NCGC00179312-03	Cyclopenthiazide	Solute carrier family 12 member 3 Inhibitor	0
NCGC00390804-01	Goserelin (acetate)	LNCaP Inhibitor	0
NCGC00016352-07	Benzocaine	Sodium channel protein type 10 subunit alpha Blocker	0
NCGC00509943-03	Serabelisib	Phosphatidylinositol 3-Kinase alpha isoform inhibitor	0
NCGC00484078-02	Darolutamide (ODM-201)	Androgen Receptor antagonist	0
NCGC00016798-11	(S)-Timolol	Beta-2 adrenergic receptor Antagonist	0

NCGC00249613-10	Carfilzomib	Proteasome inhibitor	1
NCGC00345816-01	GM-1489	Matrix Metalloproteinase (MMP) inhibitor	0
NCGC00263178-03	ENMD-981693	Aurora kinase A inhibitor	1
NCGC00016912-06	Terconazole		1
NCGC00161634-09	Artemisinin	Anti-malarial	0
NCGC00016853-06	Nabumetone	Cyclooxygenase-2 Inhibitor	0
NCGC00274077-01	Hydrocortisone 17-butyrate		0
NCGC00241102-05	Midostaurin	Receptor-type tyrosine-protein kinase FLT3 inhibitor	1
NCGC00532513-01	ADRENOCHROME MONOAMINOGUANIDINE MESILATE ANHYDROUS	0	
NCGC00263544-04	Dexrazoxane hydrochloride	DNA Topoisomerase II inhibitor	0
NCGC00092289-01	DA-3003-1	Cell Division Cycle 25 Phosphatase inhibitor	1
NCGC00346681-07	NVP-BGT226	Phosphatidylinositol 3-Kinase alpha isoform inhibitor	1
NCGC00167453-02	Clinofibrate	HMG-CoA Reductase inhibitor	0
NCGC00346704-07	Cabazitaxel	Microtubule-Stabilizing agent	0
NCGC00018295-05	Isoxsuprine hydrochloride		0
NCGC00091455-13	Celecoxib	Cyclooxygenase 2(COX2) inhibitor	0
NCGC00481567-03	LY-2409881	inhibitor of nuclear factor kappa B kinase subunit beta inhibitor	0
NCGC00390260-01	Efaproxiral	Chemosensitizer	0
NCGC00015163-09	Benserazide	Aromatic-L-amino-acid decarboxylase inhibitor	0
NCGC00095099-05	Sildenafil citrate	Phosphodiesterase 5A inhibitor	0
NCGC00022003-06	Diflorasone diacetate		0
NCGC00015288-09	Cyclothiazide	renal sodium ion absorption Inhibitor	0
NCGC00161826-02	Phenylmercuric borate	platelet aggregation Inhibitor	1
NCGC00179556-03	Cefadroxil	Solute carrier family 15 member 2 Substrate	0
NCGC00263572-01	Deferasirox	Iron Chelating Agent	1
NCGC00345782-02	GMX-1778	Nicotinamide Phosphoribosyltransferase inhibitor	1
NCGC00095597-05	Andrographolide	NACHT, LRR and PYD domains-containing protein 3 Inflammasome inhibitor	0
NCGC00386776-01	AGN-194204	Retinoic Acid Receptor RXR-alpha agonist	0
NCGC00095107-09	Gliclazide	K(ATP) Channel Blocker	0
NCGC00188440-01	Zinc dibutyldithiocarbamate		0
NCGC00346522-06	A-922500	Diacylglycerol Acyltransferase Type 1 inhibitor	0
NCGC00346876-03	PX-12	Thioredoxin Reductase inhibitor	0
NCGC00167429-04	Temozolomide	DNA Alkylating Drug	0
NCGC00166310-02	Marbofloxacin	Antibiotic	0
NCGC00016098-11	Xylazine	Sigma opioid receptor Antagonist	0
NCGC00506868-01	PF-06273340	Neurotrophic Tyrosine Kinase Receptors (NTRK) inhibitor	0
NCGC00379044-02	Paricalcitol-D6 (ABT-358)	Vitamin D3 Receptor agonist	0

NCGC00164005-06	PENICILLIN V POTASSIUM		0
NCGC00263147-03	A-674563	AKT serine/threonine kinase inhibitor	1
NCGC00319018-01	Exametazime		0
NCGC00385195-01		<MOA Unknown> | Class: N/A | Genus: N/A | Family: N/A | Species: N/A	1
NCGC00025167-02	Formoterol hemifumarate	Voltage-Gated K(V) 7 (KCNQ) Channel Activator	0
NCGC00159498-03	Metipranolol	Beta-2 adrenergic receptor Antagonist	0
NCGC00346543-05	NCGC00346543	Mitogen-Activated Protein Kinase 7 inhibitor	0
NCGC00485941-02	Norimatinib	Leucine-Rich Repeat Kinase 2 inhibitor	0
NCGC00390588-01	CPI-169 racemate		0
NCGC00182059-03	Febuxostat	Xanthine Dehydrogenase/Oxidase inhibitor	0
NCGC00249903-01	Dexamethasone isonicotinate		0
NCGC00385326-01		<MOA Unknown> | Class: Saponin | Genus: N/A | Family: N/A | Species: N/A	1
NCGC00018234-07	Sulfaguanidine		0
NCGC00378862-01	ALK inhibitor 1	ALK tyrosine kinase receptor Inhibitor	1
NCGC00480830-01	Irosustat (STX-64)	Carbonic Anhydrase 2 inhibitor	0
NCGC00016605-08	Nifenazone		0
NCGC00489906-01	VU0661013	Induced Myeloid Leukemia Cell Differentiation Protein Mcl-1 inhibitor	0
NCGC00182551-01	Cythioate	Acetylcholinesterase and butyrylcholinesterase (AChE and BChE) Inhibitor	0
NCGC00182044-02	Toltrazuril (sulfone)		0
NCGC00167445-02	Eprazinone dihydrochloride	Neurokinin 1 receptor Antagonist	0
NCGC00481107-02	NVS-CECR2-1	Cat eye syndrome critical region protein 2 inhibitor	1
NCGC00346455-05	Cobimetinib	Dual Specificity Mitogen-Activated Protein Kinase Kinase inhibitor	0
NCGC00263196-01	S6K-18	Ribosomal Protein S6 Kinase beta-1 inhibitor	0
NCGC00016371-06	Diclofenamide	Carbonic Anhydrase 2 inhibitor	0
NCGC00015067-14	Aspirin	HMG-CoA Reductase Inhibitor	0
NCGC00015043-10	GABA	GABA-A receptor; anion channel Activator	0
NCGC00016642-04	Piperacetazine		0
NCGC00263570-01	Sivelestat sodium hydrate	Neutrophil Elastase inhibitor	0
NCGC00094374-12	Seliciclib	Cyclin-Dependent Kinase 9 inhibitor	0
NCGC00016764-04	Benfotiamine		0
NCGC00263127-03	PD-166793	Matrix Metalloproteinase-13 inhibitor	0
NCGC00015705-07	Memantine hydrochloride	NMDA Receptor antagonist	0
NCGC00162129-06	Tocainide hydrochloride	Sodium channel alpha subunit Inhibitor	0
NCGC00509938-02	Brilanestrant	Selective Estrogen Receptor downregulator (SERD)	0
NCGC00522513-01	EL-102	Hypoxia-inducible factor 1-alpha inhibitor	1
NCGC00482799-01	GSK-2256098	Focal Adhesion Kinase inhibitor	0
NCGC00510317-02	Elbasvir	Anti-viral	0

<table>
<tr><td>NCGC00016951-05</td><td>Clebopride maleate</td><td>Dopamine D2 receptor Antagonist</td><td>0</td></tr>
<tr><td>NCGC00345539-10</td><td>Dacinostat</td><td>Histone deacetylase 1, class I inhibitor</td><td>1</td></tr>
<tr><td>NCGC00499248-01</td><td>CDK8i-51</td><td>Cyclin-Dependent Kinase 8 inhibitor</td><td>0</td></tr>
<tr><td>NCGC00015851-10</td><td>Protriptyline hydrochloride</td><td>Norepinephrine transporter Inhibitor</td><td>0</td></tr>
<tr><td>NCGC00014617-01</td><td>Benzylhydrochlorothiazide</td><td></td><td>0</td></tr>
<tr><td>NCGC00164583-02</td><td>Amdinocillin</td><td></td><td>0</td></tr>
<tr><td>NCGC00167767-11</td><td>IKK-16</td><td>inhibitor of nuclear factor kappa B kinase subunit beta inhibitor</td><td>1</td></tr>
<tr><td>NCGC00371126-06</td><td>Y-320</td><td>Interleukin-15 Inhibitor</td><td>1</td></tr>
<tr><td>NCGC00094516-08</td><td>Coumaphos</td><td></td><td>0</td></tr>
<tr><td>NCGC00370784-05</td><td>Resiquimod</td><td>Toll-Like Receptor 7 agonist</td><td>1</td></tr>
<tr><td>NCGC00159387-03</td><td>Laureth 10s</td><td></td><td>0</td></tr>
<tr><td>NCGC00159368-05</td><td>1-Hexadecanol</td><td>Fatty aldehyde dehydrogenase Substrate</td><td>0</td></tr>
<tr><td>NCGC00386424-05</td><td>BMH-21</td><td></td><td>1</td></tr>
<tr><td>NCGC00090691-14</td><td>Didanosine</td><td>Human immunodeficiency virus type 1 reverse transcriptase Inhibitor</td><td>0</td></tr>
<tr><td>NCGC00018294-13</td><td>Miconazole nitrate</td><td>Anti-fungal</td><td>0</td></tr>
<tr><td>NCGC00263571-01</td><td>Bendazac sodium</td><td>Xanthine dehydrogenase/oxidase Inhibitor</td><td>0</td></tr>
<tr><td>NCGC00016018-11</td><td>Tetrahydrozoline HCl</td><td>Adrenergic receptor alpha Agonist</td><td>0</td></tr>
<tr><td>NCGC00183846-01</td><td>Toborinone</td><td>Phosphodiesterase 3 Inhibitor</td><td>0</td></tr>
<tr><td>NCGC00015801-12</td><td>Pentoxifylline</td><td>"3',5'-cyclic phosphodiesterase Inhibitor"</td><td>0</td></tr>
<tr><td>NCGC00510502-02</td><td>SF-2523</td><td>Phosphatidylinositol 3-Kinase beta isoform inhibitor</td><td>1</td></tr>
<tr><td>NCGC00167739-08</td><td>GW-4064X</td><td>Farnesoid X receptor agonist</td><td>0</td></tr>
<tr><td>NCGC00016498-08</td><td>Dyclonine hydrochloride</td><td>Sodium channel protein type II alpha subunit Inhibitor</td><td>0</td></tr>
<tr><td>NCGC00263092-02</td><td>Apratastat</td><td>ADAM metallopeptidase domain 17 inhibitor</td><td>0</td></tr>
<tr><td>NCGC00015074-14</td><td>Acetazolamide</td><td>Carbonic anhydrase XII Inhibitor</td><td>0</td></tr>
<tr><td>NCGC00380955-01</td><td></td><td><MOA Unknown> | Class: Terpenoid | Genus: N/A | Family: N/A | Species: N/A</td><td>1</td></tr>
<tr><td>NCGC00159393-03</td><td>N-Acetyl-L-tyrosine</td><td></td><td>0</td></tr>
<tr><td>NCGC00169804-02</td><td></td><td><MOA Unknown> | Class: Terpenoid | Genus: N/A | Family: N/A | Species: N/A</td><td>1</td></tr>
<tr><td>NCGC00016505-06</td><td>PROPOXYCAINE HYDROCHLORIDE</td><td>0</td><td></td></tr>
<tr><td>NCGC00164589-02</td><td>Phenprobamate</td><td></td><td>0</td></tr>
<tr><td>NCGC00249934-01</td><td>Tolciclate</td><td></td><td>0</td></tr>
<tr><td>NCGC00344625-13</td><td>IOX2</td><td>Hypoxia-Inducible Factor Prolyl Hydroxylase inhibitor</td><td>0</td></tr>
<tr><td>NCGC00346441-01</td><td>ABC-294640</td><td>Sphingosine Kinase 2 inhibitor</td><td>0</td></tr>
<tr><td>NCGC00169078-03</td><td></td><td><MOA Unknown> | Class: Terpenoid | Genus: Sphaeranthus | Family: N/A | Species: confertifolius</td><td>1</td></tr>
<tr><td>NCGC00345832-03</td><td>SD-06</td><td>p38 MAPK Inhibitor</td><td>0</td></tr>
<tr><td>NCGC00346443-02</td><td>Rupintrivir</td><td>HRV 3C Protease Inhibitor</td><td>0</td></tr>
<tr><td>NCGC00024714-11</td><td>Triprolidine</td><td>Histamine H1 receptor Antagonist</td><td>0</td></tr>
<tr><td>NCGC00023373-03</td><td>Clobetasol propionate</td><td>Glucocorticoid Receptor agonist</td><td>0</td></tr>
</table>

NCGC00351598-04	WHI-P154	EGFR Inhibitor	1
NCGC00345823-02	Akt-I-1,2	AKT serine/threonine kinase inhibitor	0
NCGC00167518-09	Temsirolimus	Rapamyin analog mTORC inhibitor	0
NCGC00263541-01	Reboxetine mesylate		0
NCGC00501213-01	PF-06651600	Tyrosine-protein kinase JAK3 inhibitor	0
NCGC00159460-01	INDINAVIR SULFATE	HIV Protease Inhibitors	0
NCGC00178580-03	myo-Inositol	Serotonin 2 (5-HT2) receptor Activator	0
NCGC00025035-05	SB-203580	Mitogen-Activated Protein Kinase p38 inhibitor	0
NCGC00390563-02	GDC-0834 racemate	Bruton's Tyrosine Kinase (BTK) inhibitor	0
NCGC00249389-22	LDN-193189	ALK2 inhibitor	1
NCGC00178860-06	Ethopropazine hydrochloride	Butyrylcholinesterase Inhibitor	0
NCGC00094226-13	Piceatannol	Syk Kinase Inhibitor	0
NCGC00241411-01	NCGC00241411	Interleukin-1 Receptor-Associated Kinase 1 inhibitor	0
NCGC00263824-02	GENTAMICIN SULFATE	Antibiotic	0
NCGC00510078-03	SR-3029	Cyclin-Dependent Kinase 6 inhibitor	1
NCGC00263171-03	Serdemetan	E3 Ubiquitin-Protein Ligase Mdm2 (Hdm2) inhibitor	0
NCGC00167746-09	PNU-120596	Neuronal acetylcholine receptor subunit alpha-7 Positive allosteric modulator	0
NCGC00182990-01	Tandospirone	Serotonin 1a (5-HT1a) receptor Partial Agonist	0
NCGC00507890-02	MX-69	E3 Ubiquitin-Protein Ligase Mdm2 (Hdm2) inhibitor	0
NCGC00179342-03	Oxprenolol hydrochloride	Adrenergic receptor beta Antagonist	0
NCGC00167446-02	Methyclothiazide	Sodium-(potassium)-chloride cotransporter 2 Inhibitor	0
NCGC00159510-18	Aripiprazole	5-HT1A Receptor Partial agonist	0
NCGC00253439-03	GSK-1904529A	Insulin-like growth factor 1 receptor inhibitor	0
NCGC00181319-01	Plicamycin	DNA Antagonist	0
NCGC00253581-01	Indomethacin farnesil		0
NCGC00024595-08	Carbetapentane	Sigma-1 receptor Agonist	0
NCGC00091338-06	4-Chloro-3-methylphenol		0
NCGC00014648-04	Spiclomazine hydrochloride	Adrenergic receptor Antagonist	0
NCGC00016760-08	Carbimazole		0
NCGC00522632-01	Tussilagone	Platelet-Activating Factor Receptor antagonist	0
NCGC00344534-01	Indigo carmine		0
NCGC00015562-25	Indomethacin	Prostaglandin G/H Synthase 2 (PTGS2; COX-2) Inhibitor	0
NCGC00379184-02	PF 06465469	Tyrosine-protein kinase ITK/TSK inhibitor	1
NCGC00483117-01	PF-CBP1	Histone Acetyltransferase p300 inhibitor	1
NCGC00510482-01	Litronesib	Kinesin-Like Protein KIF11 inhibitor	0
NCGC00165811-08	IMD-0354	NF-kappaB (NFKB) Activation inhibitor	1
NCGC00164613-08	Epalrestat	Aldose Reductase inhibitor	0
NCGC00095023-08	Ethisterone	Progesterone Receptor agonist	0

NCGC00179265-05	Netilmicin Sulfate	Lysosomal phospholipase A1 Inhibitor	0
NCGC00096056-02	Ethacridine lactate hydrate		0
NCGC00181029-01	1,2-Ditetradecanoyl-rac-glycerol-3-phosphocholine	Apolipoprotein A-I Binding Agent	0
NCGC00273564-01	Bropirimine	Toll-like receptor 7 Agonist	0
NCGC00159360-03	Betamethasone dipropionate	Glucocorticoid Receptor agonist	0
NCGC00091517-06	L-Ascorbic acid		0
NCGC00024667-14	Methyldopa	Aromatic-L-amino-acid decarboxylase inhibitor	0
NCGC00262549-02	Ajmaline	Voltage-gated potassium channel subunit Kv4.3 Inhibitor	0
NCGC00178357-03	BUCLADESINE		0
NCGC00018193-11	Fluvoxamine	5-HT Reuptake Inhibitor	0
NCGC00486904-01	MRT-68601 hydrochloride	MAP/microtubule affinity-regulating kinase 3 inhibitor	1
NCGC00015243-14	Clemizole hydrochloride	Histamine H1 Receptor antagonist	0
NCGC00180998-01	Amylmetacresol		0
NCGC00167456-02	Bisibutiamine		0
NCGC00024499-06	D-Aspartic acid	Isoaspartyl peptidase/L-asparaginase Interacts	0
NCGC00387263-05	Oltipraz	Nuclear Factor Erythroid 2-Related Factor 2 activator	0
NCGC00095049-04	Exalamide		0
NCGC00510490-01	MK-1064	Orexin Receptor Type 2 antagonist	0
NCGC00387786-01	[2-Chloro-4-[[4-(2-thienyl)-2-pyrimidinyl]amino]phenyl](4-methyl-1-piperazinyl)-methanone	Glycogen synthase kinase-3 beta Inhibitor	1
NCGC00094594-17	Bacitracin (Zinc)		0
NCGC00348110-01	PRP003-032	Activated CDC42 kinase 1 inhibitor	1
NCGC00485288-01	8-Chloroadenosine 3???????,5???????-cyclic-monophosphate	cAMP-dependent protein kinase (PKA) Agonist	1
NCGC00390223-01	Fadrozole	Aromatase inhibitor	0
NCGC00390552-02	CGM-097	E3 Ubiquitin-Protein Ligase Mdm2 (Hdm2) inhibitor	0
NCGC00160445-02	Bromisovalum		0
NCGC00249899-01	Cinoxate		0
NCGC00016667-04	Benoxinate hydrochloride	Sodium channel alpha subunit Blocker	0
NCGC00025099-02	CI-966	Sodium and chloride-dependent GABA transporter 1 inhibitor	0
NCGC00384523-01		cardiac muscle contraction Activator	1
NCGC00346802-02	Glycyl-H-1152	Rho-associated protein kinase 2 inhibitor	0
NCGC00167487-12	Bicalutamide	Androgen Receptor antagonist	0
NCGC00167439-03	Trenbolone (acetate)	Progesterone receptor Binding Agent	0
NCGC00346703-08	Torcetrapib	Cholesteryl Ester Transfer Protein inhibitor	0
NCGC00015209-11	Cyclophosphamide	DNA Alkylating Drug	0

NCGC00180686-03		<MOA Unknown> | Class: Steroid | Genus: Acocanthera | Family: Apocynaceae | Species: schimperii	1
NCGC00351595-02	PF-3644022	MAP kinase-activated protein kinase 5 inhibitor	0
NCGC00016759-04	Naproxen sodium	Cyclooxygenase 2(COX2) inhibitor	0
NCGC00480721-01	KG 5		1
NCGC00016397-04	Benzydamine hydrochloride	prostaglandin production involved in inflammatory response Inhibitor	0
NCGC00346695-02	MK-2461	Fibroblast Growth Factor Receptor 3 inhibitor	0
NCGC00274075-01	1-Octacosanol	PI3K-Akt signaling pathway Activator	0
NCGC00182549-04	Vicriviroc (SCH-417690)	C-C chemokine receptor type 5 antagonist	0
NCGC00166413-09	Argatroban	Thrombin inhibitor	0
NCGC00163355-05	Indigo		0
NCGC00263584-01	Tirofiban hydrochloride monohydrate	Integrin alpha-IIb/beta-3 Antagonist	0
NCGC00499351-01	GGTI-2418	Protein farnesyltransferase/geranylgeranyltransferase type-1 inhibitor	0
NCGC00163338-03	D-Phenylalanine		0
NCGC00160456-03	2-Iodohippuric acid	renal filtration Interacts	0
NCGC00522533-01	Olodaterol	Beta-2 adrenergic receptor Agonist	0
NCGC00090905-16	Busulfan	DNA Damaging Drug	0
NCGC00169210-02		<MOA Unknown> | Class: Sesquiterpen | Genus: Calea | Family: Compositae | Species: urticifolia	1
NCGC00024822-03	EVANS BLUE	Serum albumin Binding Agent	0
NCGC00386681-01	PF-4693627	Prostaglandin E Synthase inhibitor	0
NCGC00246966-11	Flubendazole	Tubulin polymerization inhibitor	0
NCGC00016281-11	Phenacetin	Prostaglandin G/H synthase 1 Inhibitor	0
NCGC00346495-14	TGX-221	Phosphatidylinositol 3-Kinase beta isoform inhibitor	0
NCGC00386415-05	Cerdulatinib	Tyrosine-protein kinase SYK inhibitor	0
NCGC00016438-04	Procarbazine hydrochloride	DNA Alkylating Drug	0
NCGC00166415-03	Palonosetron hydrochloride	Serotonin 3a (5-HT3a) receptor Antagonist	0
NCGC00347957-09	UNC-669	Lethal(3)malignant Brain Tumor-like Protein 3 inhibitor	0
NCGC00522475-03	Verubecestat (MK-8931)	Beta-Secretase 1 inhibitor	0
NCGC00261973-01	Butoctamide semisuccinate		0
NCGC00094082-08	2-Methoxyestradiol (2-MeOE2)	Hypoxia-inducible factor 1-alpha inhibitor	1
NCGC00094779-05	Salicyl alcohol		0
NCGC00386757-02	OTX-008	Galectin 1 (allosteric) inhibitor	0
NCGC00015884-15	Rilmenidine	Imidazoline I1 (Nischarin) Receptor agonist	0
NCGC00160392-03	LOMOFUGIN		1
NCGC00179302-06	Indirubin	Cyclin-Dependent Kinase 1 inhibitor	0
NCGC00262829-02	C-188-9	Signal Transducer and activator of Transcription 3 inhibitor	0

NCGC00183007-01	Risarestat	Aldose reductase Inhibitor	0
NCGC00166271-02	Pregnenolone acetate		0
NCGC00378630-01	BMS-690514	Vascular Endothelial Growth Factor Receptor 2 (VEGFR-2) inhibitor	1
NCGC00378895-01	Cebranopadol	Nociceptin Receptor agonist	0
NCGC00385325-01		<MOA Unknown> | Class: Steroide | Genus: N/A | Family: N/A | Species: N/A	1
NCGC00380400-01		<MOA Unknown> | Class: Terpenoid | Genus: N/A | Family: N/A | Species: N/A	1
NCGC00242225-02			1
NCGC00487123-01	SYUIQ-5		1
NCGC00250373-04	A-769662	AMP-Activated Protein Kinase (AMPK) activator	0
NCGC00181134-04	Haloprogin		0
NCGC00015233-07	Calmidazolium chloride	Calcium/calmodulin-dependent protein kinase kinase 2 inhibitor	0
NCGC00319019-01	Nemorubicin	DNA Topoisomerase II inhibitor	0
NCGC00379021-02	SU-014813	Vascular Endothelial Growth Factor Receptor 2 (VEGFR-2) inhibitor	0
NCGC00183097-01	Beraprost sodium	Thyroid hormone receptor Antagonist	0
NCGC00095303-04	Cloxyquin	Mycobacterium tuberculosis Inhibitor	0
NCGC00065934-05	Oxcarbazepine	Sodium channel inhibitor	0
NCGC00247665-04	Anagrelide hydrochloride	Phosphodiesterase III (PDE3) inhibitor	0
NCGC00378878-02	Lucitanib	Vascular Endothelial Growth Factor Receptor 3 (VEGFR-3) inhibitor	0
NCGC00371080-02	Dexmedetomidine	Adrenergic receptor alpha-2 Agonist	0
NCGC00188865-03	Pazopanib	Vascular Endothelial Growth Factor Receptor 3 (VEGFR-3) inhibitor	0
NCGC00510508-01	TPOP-146	Histone Acetyltransferase p300 inhibitor	0
NCGC00166238-02	Benznidazole	Pyruvate:ferredoxin oxidoreductase Substrate	0
NCGC00487207-01	Symadex		1
NCGC00263226-01	BAY-60-7550	Phosphodiesterase 2A inhibitor	0
NCGC00015368-11	Dicyclomine	Muscarinic acetylcholine receptor M3 Antagonist	0
NCGC00160502-01			0
NCGC00013917-09	NSC-87877	Tyrosine-protein phosphatase non-receptor type 11 inhibitor	0
NCGC00094918-07	Saccharin	Taste receptor type 1 member 3 Binding Agent	0
NCGC00242493-09	Quizartinib	Receptor-type tyrosine-protein kinase FLT3 inhibitor	0
NCGC00090680-06	Carbaryl	Acetylcholinesterase Inhibitor	0
NCGC00345022-04	IDE-2	lineage-specific differentiation enhancer	0
NCGC00345789-04	Venetoclax (ABT-199)	Apoptosis regulator Bcl-2 inhibitor	1
NCGC00387419-01	KP372-1		1
NCGC00262604-18	BKM-120	Phosphatidylinositol 3-Kinase beta isoform inhibitor	1
NCGC00371045-07	AMD-3465	Chemokine CXCR4 Receptor antagonist	0
NCGC00346875-02	BMS-299897	gamma-Secretase inhibitor	0

NCGC00018241-07	Betamipron	Solute carrier family 22 member 8 Inhibitor	0
NCGC00384474-01	Pralnacasan	Caspase-1 inhibitor	0
NCGC00182082-03	Tesaglitazar		0
NCGC00181012-01	Cefuzonam hydrochloride	Peptidoglycan biosynthesis Inhibitor	0
NCGC00345739-02	KH-CB19	Dual-Specificity Tyrosine-(Y)-Phosphorylation Regulated Kinase 1A inhibitor	0
NCGC00021139-11	5,5-Diphenylhydantoin		0
NCGC00380307-01		<MOA Unknown> | Class: Alkaloid | Genus: N/A | Family: N/A | Species: N/A	1
NCGC00263104-06	Foretinib	Vascular Endothelial Growth Factor Receptor 2 (VEGFR-2) inhibitor	1
NCGC00016024-05	(??)-Taxifolin	NF-kappaB (NFKB) modulator	0
NCGC00164100-01	Trisiloxane, octamethyl-		0
NCGC00159404-04	Sulisobenzone		0
NCGC00182076-03	Polythiazide	Thiazide-sensitive sodium-chloride cotransporter Inhibitor	0
NCGC00189073-04	Selumetinib (AZD6244)	Dual Specificity Mitogen-Activated Protein Kinase Kinase 1 inhibitor	0
NCGC00160451-02	Ibufenac		0
NCGC00018261-05	Perhexiline	Carnitine O-Palmitoyltransferase 1 inhibitor	0
NCGC00346896-03	MCOPPB	Nociceptin Receptor agonist	0
NCGC00160408-06	Benziodarone		0
NCGC00167356-07	Raltegravir	Anti-viral	0
NCGC00249905-01	Phthalofyne		0
NCGC00389655-01	Angiotensin II		0
NCGC00167505-03	Eptifibatide	Integrin alpha-IIb/beta-3 Inhibitor	0
NCGC00164248-05	Sucrose	Starch and sucrose metabolism Substrate	0
NCGC00015622-10	LY-294002	Phosphatidylinositol 3-Kinase alpha isoform inhibitor	0
NCGC00094956-04	Cephaloridine	Solute carrier family 22 member 7 Inhibitor	0
NCGC00162137-02	Dehydroepiandrosterone Sulfate (sodium salt)	Estrogen receptor alpha Partial Agonist	0
NCGC00370724-05	CHR-6494 TFA Salt	Serine/threonine-protein kinase haspin Inhibitor	1
NCGC00168771-04	Fosamprenavir Calcium Salt		0
NCGC00487085-02	CFI-400945	Serine/threonine-protein kinase PLK4 inhibitor	0
NCGC00506875-01	Vilanterol (GW642444; GW 642444X)	"adrenoceptor beta 2, surface Agonist"	0
NCGC00510142-01	Methylprednisolone aceponate		0
NCGC00182058-01	Doxercalciferol	Vitamin D3 Receptor agonist	0
NCGC00181788-01	Cefetamet pivoxil HCl		0
NCGC00016251-13	Probenecid	Solute carrier family 22 member 8 Inhibitor	0
NCGC00093560-19	Finasteride	3-Oxo-5-alpha-steroid 4-dehydrogenase 1 inhibitor	0
NCGC00181337-01	2,4-Dichlorobenzyl alcohol		0
NCGC00182974-01	Improsulfan tosylate		0

NCGC00180796-04	Withaferin A	NF-kappaB (NFKB) Activation inhibitor	1
NCGC00159352-14	ETIDRONATE DISODIUM		0
NCGC00346661-05	Cephalomannine	Tubulin polymerization inhibitor	0
NCGC00346540-03	FESOTERODINE FUMARATE	Muscarinic Antagonists	0
NCGC00021336-14	Diclofenac sodium	Cyclooxygenase-1/2/3 Inhibitor	0
NCGC00025262-02	Cisapride		0
NCGC00390646-02	Isavuconazole		0
NCGC00388506-02	Pinaverium bromide		0
NCGC00532501-01	ARIPIPRAZOLE LAUROXIL	Serotonin 1a (5-HT1a) receptor Partial Agonist	0
NCGC00347217-03	PRP003-013	Discoidin Domain-Containing Receptor 1 inhibitor	1
NCGC00094112-11	BIO	Arachidonate 5-Lipoxygenase inhibitor	1
NCGC00378723-01	CTS-1027	Matrix Metalloproteinase-12 inhibitor	0
NCGC00018204-13	Vinpocetine	Na channel blocker, also PDE1, IKK inhibitor	0
NCGC00522579-01	ERK5-IN-1	Mitogen-Activated Protein Kinase 3 (ERK1) inhibitor	1
NCGC00091120-13	Griseofulvin	Tubulin beta chain Inhibitor	0
NCGC00344542-01	Transfluthrin	Cytochrome P450 3A2 Activator	0
NCGC00016348-15	Ethoxyquin	Heat shock protein HSP 90-beta Inhibitor	0
NCGC00346718-02	CP-673451	Platelet-derived growth factor receptor beta inhibitor	0
NCGC00092318-12	NSC-23766	Rac1 GTPase inhibitor	0
NCGC00182066-02	Fluprednidene acetate		0
NCGC00347063-02	KUC110948N	Anti-malarial	0
NCGC00510231-01	GDC-0077	Poly [ADP-ribose] polymerase 1 inhibitor	0
NCGC00379009-01	Octreotide acetate	Growth Hormone Release Inhibitors	0
NCGC00159502-03	N-Cyclohexyl-2-benzothiazolesulfenamide	Aryl hydrocarbon receptor Agonist	0
NCGC00018187-09	Cinnarizine	Voltage-gated calcium channel Blocker	0
NCGC00507854-01	MI-503	Menin/MLL Interaction inhibitor	0
NCGC00386310-01	Selinexor	Exportin-1 antagonist	1
NCGC00025228-15	Rottlerin		1
NCGC00263179-09	AR-00341677	Serine/threonine-protein kinase B-raf inhibitor	0
NCGC00481319-01	AZD-9496 maleate	Selective Estrogen Receptor downregulator (SERD)	0
NCGC00163411-06	Triptolide	dCTP Pyrophosphatase 1 inhibitor	1
NCGC00167421-02	Sevoflurane	GABA-B receptor Modulator	0
NCGC00381237-01		<MOA Unknown> | Class: Terpenoid | Genus: Ajania | Family: N/A | Species: spp	1
NCGC00346573-02	Sapitinib	Epidermal Growth Factor Receptor inhibitor	1
NCGC00093354-13	Niacinamide	Poly [ADP-ribose] polymerase 1 Binder	0
NCGC00384836-01	Trinactin		1
NCGC00264052-02	Hyodeoxycholic acid	Glucocorticoid Receptor agonist	0
NCGC00179367-03	Iopromide		0

NCGC00167811-13	GW-9508	Free Fatty Acid Receptor 1 agonist	0
NCGC00015623-11	Proglumide	Cholecystokinin B Receptor antagonist	0
NCGC00247988-03	Cochineal	Solute carrier organic anion transporter family member 1B1 Inhibitor	0
NCGC00345082-02	KHS-101	Transforming acidic coiled-coil-containing protein 3 modulator	0
NCGC00511348-01	ML226	Protein ABDH11 inhibitor	0
NCGC00165844-06	NU-6027	Serine-protein kinase ATR inhibitor	0
NCGC00532510-01	PREDNISOLONE VALERATE ACETATE	0	
NCGC00166037-04	beta-Glycerol phosphate disodium salt pentahydrate	0	
NCGC00379255-03	FRAX-486	Serine/threonine-protein kinase PAK 1 inhibitor	1
NCGC00346707-05	Lonafarnib	Protein farnesyltransferase/geranylgeranyltransferase type-1 inhibitor	1
NCGC00181041-01	5-Chloro-2-methyl-3(2H)-isothiazolone	aerobic respiration Inhibitor	0
NCGC00389453-01	Chlortetracycline HCl	translation Inhibitor	0
NCGC00163160-04	Talipexole	Dopamine D4 receptor Agonist	0
NCGC00484844-01	Cilastatin	Renal dipeptidase Inhibitor	0
NCGC00389582-02	FRAX-597	Serine/threonine-protein kinase PAK 2 inhibitor	0
NCGC00486892-01	CPI-455	Lysine-Specific Demethylase 5B inhibitor	0
NCGC00025212-26	Cantharidin	Protein Phosphatase 2A inhibitor	0
NCGC00263111-11	GSK-3787	PPARdelta Antagonist	0
NCGC00485046-01	PF-06439015	ALK Tyrosine Kinase Receptor inhibitor	0
NCGC00015443-22	Flecainide acetate	Sodium channel protein type 5 subunit alpha Blocker	0
NCGC00346821-04	PD-150606	Calpain inhibitor	0
NCGC00167563-01	Mofezolac	Cyclooxygenase-1 Inhibitor	0
NCGC00390090-01	Alclometasone dipropionate	Glucocorticoid receptor Agonist	0
NCGC00347890-01			1
NCGC00241114-02	CPI-1189	Mitogen-Activated Protein Kinase p38 inhibitor	0
NCGC00263133-09	PF-573228	Focal Adhesion Kinase inhibitor	0
NCGC00386371-04	Ro3280	Serine/threonine-protein kinase PLK1 Inhibitor	1
NCGC00371263-02	OICR-9429	WD repeat-containing protein 5/Histone-lysine N-methyltransferase 2A Interaction inhibitor	0
NCGC00160519-13	Acipimox	Hydroxycarboxylic Acid Receptor 2 Partial antagonist	0
NCGC00181349-02	Strontium Ranelate		0
NCGC00181028-02	D-Pantethine	HMG-CoA reductase Inhibitor	0
NCGC00498399-01	Aderbasib	ADAM metallopeptidase domain 10 inhibitor	0
NCGC00378588-02	NCGC00378588	E3 Ubiquitin-Protein Ligase Mdm2 (Hdm2) inhibitor	1
NCGC00015964-17	Spiperone	Dopamine D4 receptor Antagonist	0

NCGC00181325-03	Menaquinone	bone resorption Inhibitor	0
NCGC00182026-02	Tiludronate disodium salt hydrate	Osteoclasts Inhibitor	0
NCGC00179382-03	Hexylcaine hydrochloride	Sodium channel alpha subunit Blocker	0
NCGC00167744-02	Methylperone		0
NCGC00386234-03	CEP-32496	Proto-oncogene tyrosine-protein kinase receptor Ret inhibitor	0
NCGC00015036-11	Amantadine hydrochloride	Dopamine Receptor D3 agonist	0
NCGC00186024-12	SB-525334	TGF-beta receptor type-1 inhibitor	0
NCGC00346723-07	PHA-767491A	Cell Division Cycle 7-Related Protein Kinase inhibitor	0
NCGC00015578-18	Ketoprofen	Cyclooxygenase 1(COX1) inhibitor	0
NCGC00386398-03	OTS-167	Maternal Embryonic Leucine Zipper Kinase inhibitor	1
NCGC00015204-09	Carmustine	DNA Alkylating Drug	0
NCGC00159541-03	S-Trityl-L-cysteine	Mitotic Kinesin Eg5 Inhibitor	0
NCGC00274057-01	Phenylpropanolamine hydrochloride	Adrenergic receptor alpha-1 Agonist	0
NCGC00016401-09	Hydroflumethiazide	Thiazide-sensitive sodium-chloride cotransporter Inhibitor	0
NCGC00263118-02	Lirimilast	Phosphodiesterase IIII (PDE4) Inhibitor	0
NCGC00384204-04	SGC-707	Protein arginine N-methyltransferase 3 inhibitor	0
NCGC00179259-06	Meropenem		0
NCGC00164594-02	Loteprednol etabonate	Glucocorticoid Receptor agonist	0
NCGC00346687-10	BX-912	3-Phosphoinositide Dependent Kinase 1 inhibitor	0
NCGC00262543-01	Midecamycin	Ribosome Inhibitor	0
NCGC00510779-01	BAY-876	Orally bioavailable GLUT1 Inhibitor for oncology	0
NCGC00165815-06	NSC-724771	Nitric Oxide Donor	0
NCGC00094571-10	Crotamiton	Transient receptor potential cation channel subfamily V member 4 antagonist	0
NCGC00389741-01	MK 0893	Glucagon receptor Antagonist	1
NCGC00095486-03	Ergosterol	Sterol (fungal cell wall)	0
NCGC00095933-04	Phenyl 4-aminosalicylate		0
NCGC00250412-21	JQ1	Bromodomain-Containing Protein 4 (Brd4) inhibitor	1
NCGC00023643-15	Estrone	Steroid hormone	0
NCGC00346553-10	BS-181	Cyclin-Dependent Kinase 7 inhibitor	0
NCGC00183277-01	Micronomicin sulfate		0
NCGC00346517-10	Daptomycin	Lipopeptide Antibiotic	0
NCGC00187983-01	Thiazovivin	Rho-associated protein kinase 1 inhibitor	0
NCGC00016296-07	Dicumarol	Vitamin k epoxide reductase complex subunit 1 isoform 1 Inhibitor	0
NCGC00408915-01	Acalisib	Phosphatidylinositol 3-Kinase delta isoform inhibitor	0
NCGC00510347-01	CC-885	Cereblon inhibitor	0

<table>
<tr><td>NCGC00263207-02</td><td>QS11</td><td>ADP-ribosylation factor GTPase activating protein 1 inhibitor</td><td>0</td></tr>
<tr><td>NCGC00373060-03</td><td></td><td></td><td>1</td></tr>
<tr><td>NCGC00021268-04</td><td>Hydroxyprogesterone caproate</td><td></td><td>1</td></tr>
<tr><td>NCGC00159346-04</td><td>Terbinafine</td><td>Fungal Squalene Monooxygenase Inhibitor</td><td>0</td></tr>
<tr><td>NCGC00390643-01</td><td>Prexasertib</td><td>Checkpoint kinase 1 inhibitor</td><td>0</td></tr>
<tr><td>NCGC00070736-03</td><td>Imiquimod</td><td>Toll-Like Receptor 7 agonist</td><td>0</td></tr>
<tr><td>NCGC00242498-14</td><td>D-4476</td><td>Casein Kinase 1 epsilon inhibitor</td><td>0</td></tr>
<tr><td>NCGC00168848-03</td><td></td><td><MOA Unknown> | Class: Steroid | Genus: Acocanthera | Family: Apocynaceae | Species: schimperii</td><td>1</td></tr>
<tr><td>NCGC00390475-03</td><td>Napabucasin</td><td>Signal Transducer and activator of Transcription 3 inhibitor</td><td>1</td></tr>
<tr><td>NCGC00351477-05</td><td>UNC-1999</td><td>Histone-lysine N-methyltransferase EZH2 inhibitor</td><td>0</td></tr>
<tr><td>NCGC00510682-01</td><td>GSK-2250665A</td><td>Tyrosine-protein kinase ITK/TSK inhibitor</td><td>0</td></tr>
<tr><td>NCGC00015841-11</td><td>Pargyline hydrochloride</td><td>Monoamine oxidase B Inhibitor</td><td>0</td></tr>
<tr><td>NCGC00024427-05</td><td>Naltrexone hydrochloride</td><td>Mu-Type Opioid Receptor antagonist</td><td>0</td></tr>
<tr><td>NCGC00346442-02</td><td>Phenprocoumon</td><td>Cytochrome P450 3A4 Substrate</td><td>0</td></tr>
<tr><td>NCGC00522030-01</td><td>Budralazine</td><td>adrenergic receptor signaling pathway Modulator</td><td>0</td></tr>
<tr><td>NCGC00347623-02</td><td></td><td><MOA Unknown> | Class: Terpenoid | Genus: N/A | Family: N/A | Species: N/A</td><td>1</td></tr>
<tr><td>NCGC00016529-07</td><td>Pramoxine hydrochloride</td><td>Sodium channel alpha subunit Antagonist</td><td>0</td></tr>
<tr><td>NCGC00015834-27</td><td>Primidone</td><td>Gamma-aminobutyric acid receptor subunit alpha-1 modulator</td><td>0</td></tr>
<tr><td>NCGC00262941-01</td><td>Ipenoxazone hydrochloride</td><td>Glutamate [NMDA] receptor Antagonist</td><td>0</td></tr>
<tr><td>NCGC00262009-03</td><td>Bisindolylmaleimide</td><td>Cyclin-Dependent Kinase 4 inhibitor</td><td>0</td></tr>
<tr><td>NCGC00345084-02</td><td>Robotnikinin</td><td>Sonic Hedgehog protein inhibitor</td><td>0</td></tr>
<tr><td>NCGC00501212-01</td><td>Peficitinib</td><td>Tyrosine-protein kinase JAK3 inhibitor</td><td>0</td></tr>
<tr><td>NCGC00167442-03</td><td>Tamsulosin hydrochloride</td><td>alpha1-Adrenoceptor Antagonists</td><td>0</td></tr>
<tr><td>NCGC00343505-01</td><td>Wr-301801</td><td>signaling/transport</td><td>1</td></tr>
<tr><td>NCGC00017312-04</td><td>Uridine</td><td>Anti-anxiety drug</td><td>0</td></tr>
<tr><td>NCGC00346839-01</td><td>4-Quinazolinamine</td><td>Epidermal Growth Factor Receptor inhibitor</td><td>0</td></tr>
<tr><td>NCGC00167751-05</td><td>SSR-69071</td><td>Neutrophil Elastase inhibitor</td><td>0</td></tr>
<tr><td>NCGC00015856-10</td><td>Prochlorperazine dimaleate</td><td>Dopamine D2 Antagonist</td><td>0</td></tr>
<tr><td>NCGC00344532-01</td><td>Mucic acid</td><td></td><td>0</td></tr>
<tr><td>NCGC00090730-03</td><td>D-Camphor</td><td></td><td>0</td></tr>
<tr><td>NCGC00249928-01</td><td>Eicosapentaenoic acid ethyl ester</td><td>0</td><td></td></tr>
<tr><td>NCGC00263517-03</td><td>BORNEOL</td><td></td><td>0</td></tr>
<tr><td>NCGC00188690-02</td><td>Prasugrel</td><td>P2Y Purinoceptor 12 antagonist</td><td>0</td></tr>
<tr><td>NCGC00180626-04</td><td>Cephaeline</td><td></td><td>1</td></tr>
<tr><td>NCGC00091058-11</td><td>Linolenic acid</td><td>Fatty acid desaturase 2 Substrate</td><td>0</td></tr>
<tr><td>NCGC00094819-07</td><td>Hexetidine</td><td></td><td>0</td></tr>
<tr><td>NCGC00379076-01</td><td>Maxacalcitol-D6</td><td>Vitamin D receptor Agonist</td><td>0</td></tr>
</table>

<table>
<tr><td>NCGC00378857-02</td><td>Preladenant</td><td>Adenosine Receptor A2A antagonist</td><td>0</td></tr>
<tr><td>NCGC00480972-03</td><td>GSK-864</td><td>Isocitrate Dehydrogenase [NADP] cytoplasmic (Arg132His Mutant) inhibitor</td><td>0</td></tr>
<tr><td>NCGC00018210-05</td><td>Oxyphencyclimine hydrochloride</td><td>Muscarinic acetylcholine receptor M4 Binding Agent</td><td>0</td></tr>
<tr><td>NCGC00248587-03</td><td>METHYLPHENIDATE HYDROCHLORIDE</td><td>0</td><td></td></tr>
<tr><td>NCGC00380850-01</td><td></td><td><MOA Unknown> | Class: Sesquiterpen | Genus: Tithonia | Family: Compositae | Species: diversifolia</td><td>1</td></tr>
<tr><td>NCGC00378622-10</td><td>Osimertinib mesylate</td><td>Epidermal Growth Factor Receptor (Thr790Met Mutant) inhibitor</td><td>0</td></tr>
<tr><td>NCGC00510909-01</td><td>Acolbifene</td><td>Selective Estrogen Receptor modulator (SERM)</td><td>0</td></tr>
<tr><td>NCGC00181911-01</td><td>Bucolome</td><td>Cytochrome P450 2C9 Inhibitor</td><td>0</td></tr>
<tr><td>NCGC00169803-03</td><td></td><td><MOA Unknown> | Class: Steroid | Genus: Nerium | Family: Apocynaceae | Species: oleander</td><td>1</td></tr>
<tr><td>NCGC00485421-01</td><td>8-Aminoadenosine</td><td>Generic Transcription Pathway Inhibitor</td><td>1</td></tr>
<tr><td>NCGC00263206-04</td><td>AMG-47a</td><td>Tyrosine-protein kinase Lck inhibitor</td><td>1</td></tr>
<tr><td>NCGC00167798-06</td><td>Oxaliplatin</td><td>DNA Alkylating Drug</td><td>0</td></tr>
<tr><td>NCGC00483923-01</td><td>AMG-337</td><td>Hepatocyte Growth Factor Receptor inhibitor</td><td>0</td></tr>
<tr><td>NCGC00167803-13</td><td>GSI-IX</td><td>gamma-Secretase inhibitor</td><td>0</td></tr>
<tr><td>NCGC00092384-05</td><td>Fenobam</td><td>Glutamate receptor metabotropic 5 antagonist</td><td>0</td></tr>
<tr><td>NCGC00274270-01</td><td></td><td></td><td>0</td></tr>
<tr><td>NCGC00184708-02</td><td></td><td>smooth muscle contraction Inhibitor</td><td>0</td></tr>
<tr><td>NCGC00370777-04</td><td>Gedatolisib</td><td>Phosphatidylinositol 3-Kinase alpha isoform inhibitor</td><td>1</td></tr>
<tr><td>NCGC00165863-16</td><td>PD-173074</td><td>Fibroblast Growth Factor Receptor 3 inhibitor</td><td>0</td></tr>
<tr><td>NCGC00090504-14</td><td>Isoliquiritigenin</td><td>NMDA Receptor antagonist</td><td>0</td></tr>
<tr><td>NCGC00350413-05</td><td>ESI-09</td><td></td><td>1</td></tr>
<tr><td>NCGC00016394-10</td><td>2-Hydroxy-4-methoxybenzophenone</td><td>0</td><td></td></tr>
<tr><td>NCGC00249910-01</td><td>Ibafloxacine</td><td>Bacterial DNA gyrase Inhibitor</td><td>0</td></tr>
<tr><td>NCGC00508900-01</td><td>VU-0364849</td><td>ALK2 inhibitor</td><td>0</td></tr>
<tr><td>NCGC00510931-01</td><td>Mavacoxib</td><td>Cyclooxygenase-2 Inhibitor</td><td>0</td></tr>
<tr><td>NCGC00348107-03</td><td>c-FMS Inhibitor</td><td>Mast/stem cell Growth Factor Receptor Kit inhibitor</td><td>0</td></tr>
<tr><td>NCGC00263825-02</td><td>HETACILLIN POTASSIUM</td><td>Bacterial penicillin-binding protein Inhibitor</td><td>0</td></tr>
<tr><td>NCGC00389552-01</td><td>CEFONICID SODIUM</td><td>Bacterial penicillin-binding protein Inhibitor</td><td>0</td></tr>
<tr><td>NCGC00390187-02</td><td>Delafloxacin (meglumine)</td><td>Staphylococcus Aureus Inhibitor</td><td>0</td></tr>
<tr><td>NCGC00016306-09</td><td>D-Cycloserine</td><td>Anti-tuberculosis</td><td>0</td></tr>
<tr><td>NCGC00390606-01</td><td>TNKS-656</td><td>Tankyrase-1 inhibitor</td><td>0</td></tr>
<tr><td>NCGC00178854-03</td><td>Minocycline hydrochloride</td><td>Tetracycline Antibiotic</td><td>0</td></tr>
<tr><td>NCGC00381559-06</td><td>Resminostat</td><td>Histone deacetylase 1, class I inhibitor</td><td>1</td></tr>
<tr><td>NCGC00167490-03</td><td>Lumefantrine</td><td>Anti-malarial</td><td>0</td></tr>
<tr><td>NCGC00242503-02</td><td>Meclinertant</td><td>Carboxypeptidase A inhibitor</td><td>0</td></tr>
</table>

NCGC00479249-03	Enasidenib	Isocitrate Dehydrogenase [NADP] mitochondrial inhibitor	0
NCGC00346658-03	Crenolanib	Receptor-type tyrosine-protein kinase FLT3 inhibitor	0
NCGC00345844-02	Bayer-18	Non-receptor tyrosine-protein kinase Tyk2 inhibitor	0
NCGC00386060-01		<MOA Unknown> | Class: N/A | Genus: N/A | Family: N/A | Species: N/A	1
NCGC00165972-02	1-Phenyl-1-propanol	Glycolysis / Gluconeogenesis Activator	0
NCGC00347280-04	IKK-2 Inhibitor VIII	inhibitor of nuclear factor kappa B kinase subunit beta inhibitor	1
NCGC00507976-02	Derquantel		0
NCGC00181000-02	Suplatast tosylate	Mediator Release inhibitor	0
NCGC00485933-01	TAK-593	Vascular Endothelial Growth Factor Receptor 2 (VEGFR-2) inhibitor	0
NCGC00378789-02	Etomoxir	Carnitine O-Palmitoyltransferase 1 inhibitor	0
NCGC00509861-02	Auristatin PE	microtubule polymerization Inhibitor	0
NCGC00182064-02	Ceftezole sodium	Peptidoglycan biosynthesis Inhibitor	0
NCGC00166294-08	Sparfloxacin	DNA Topoisomerase IV Inhibitor	0
NCGC00183881-01	Moxifloxacin hydrochloride	Anti-bacterial	0
NCGC00386697-01	Wnt agonist 1	tubulin complex assembly Inhibitor	1
NCGC00378665-02	Ixabepilone	Microtubule-Stabilizing agent	0
NCGC00016978-13	Repaglinide	Insulin Secretagogue	0
NCGC00090964-04	Tetraethylenepentamine	Oxidative Stress Inhibitor	0
NCGC00167435-07	Nilvadipine	Voltage-gated L-type calcium channel alpha-1C subunit Blocker	0
NCGC00023654-13	Captopril	Angiotensin-converting enzyme Inhibitor	0
NCGC00021575-05	Fluorometholone	Glucocorticoid receptor Agonist	0
NCGC00167441-02	Pramipexole hydrochloride	Dopamine Receptor D3 agonist	0
NCGC00510498-01	Adomeglivant	Glucagon Receptor antagonist	0
NCGC00182975-01	Amoebicon		0
NCGC00356069-06	GSK1324726A	Bromodomain-Containing Protein 4 (Brd4) inhibitor	1
NCGC00161670-08	Pepstatin	Aspartyl Protease Inhibitor	0
NCGC00242482-11	SNS-314	Aurora kinase A inhibitor	1
NCGC00263521-07	Deflazacort	Glucocorticoid Receptor agonist	0
NCGC00387463-02	MPS1-IN-1	ALK Tyrosine Kinase Receptor inhibitor	0
NCGC00263134-08	PF-4708671	Ribosomal Protein S6 Kinase beta-1 inhibitor	0
NCGC00168778-06	Solifenacin (hydrochloride)	Muscarinic acetylcholine receptor M5 Antagonist	0
NCGC00025060-22	Methotrexate	Dihydrofolate Reductase inhibitor	0
NCGC00164246-18	D-Sorbitol	Osmotic diuretic agent	0
NCGC00510921-01	Lucanthone	DNA Topoisomerase II inhibitor	0
NCGC00022019-03	Clobetasone butyrate	corticosteroid	0
NCGC00165855-05	Oxamflatin	Histone deacetylase 1, class I inhibitor	1
NCGC00166104-04	alpha-Lipoic acid amide	apoptotic process Inhibitor	0
NCGC00018199-09	Rhein	component of rhubarb	0

NCGC00186631-02	24, 25-Dihydroxy VD3	Vitamin D3 receptor Agonist	0
NCGC00183854-01	Oxendolone		0
NCGC00390644-02	CTX0294885	Casein kinase I Inhibitor	1
NCGC00522019-01	Polaprezinc		0
NCGC00016285-05	Sulfanilamide	Carbonic Anhydrase 1 inhibitor	0
NCGC00381113-01		<MOA Unknown> | Class: Terpenoid | Genus: N/A | Family: N/A | Species: N/A	1
NCGC00238451-09	Olaparib	Poly [ADP-ribose] polymerase 1 inhibitor	0
NCGC00498432-01	Olmutinib	Epidermal Growth Factor Receptor inhibitor	0
NCGC00370980-01	A-419259	Tyrosine-protein kinase HCK inhibitor	1
NCGC00021301-06	Fluocinolone acetonide	Glucocorticoid Receptor agonist	0
NCGC00016602-04	Benperidol	Dopamine D2 receptor Antagonist	0
NCGC00167468-02	Benorylate	prostaglandin biosynthetic process Inhibitor	0
NCGC00179341-04	Ondansetron	5-HT3 Antagonists	0
NCGC00346524-02	Nanchangmycin	Polyether Antibiotic	1
NCGC00164534-02	4-Phenolsulfonic acid		0
NCGC00347131-03	D(-)-Tartaric acid		0
NCGC00016509-08	TRIMETHOBENZAMIDE HYDROCHLORIDE	Dopamine D2 receptor Antagonist	0
NCGC00346547-03	PHT-427	3-Phosphoinositide Dependent Kinase 1 inhibitor	0
NCGC00532505-01	N,N-DIMETHYLGLYCINE HYDROCHLORIDE	Glutamate [NMDA] receptor Agonist	0
NCGC00160620-02	L-Methionine	S-adenosylmethionine synthetase (MAT 1 and MAT 2) Substrate	0
NCGC00091015-01	Phenolphthalin		0
NCGC00386668-01	M-024	Angiotensin AT2 Receptor Agonist	0
NCGC00015234-19	Carbamazepine	Sodium channel protein type 5 subunit alpha Blocker	0
NCGC00016523-05	Iodipamide	Complement factor B Inhibitor	0
NCGC00181916-01	Sulfaethoxypyridazine		0
NCGC00167489-03	Sarpogrelate hydrochloride	5HT(2A) and 5HT(2B) inhibitor	0
NCGC00181091-01	Bunamidine hydrochloride		1
NCGC00263159-10	OSI-930	Vascular Endothelial Growth Factor Receptor 2 (VEGFR-2) inhibitor	0
NCGC00014670-26	Domperidone	Dopamine Receptor D2 antagonist	0
NCGC00242057-03	GW-768505A	Angiopoietin-1 Receptor inhibitor	0
NCGC00346519-04	Teicoplanin	glycopeptide antibiotic	0
NCGC00522503-02	VELPATASVIR	Nonstructural protein 5A Inhibitor	0
NCGC00177986-12	Pefloxacin	Antibacterial	0
NCGC00073457-04	Metomidate hydrochloride	Cytochrome P450 2A5 Inhibitor	0
NCGC00384630-01		<MOA Unknown> | Class: Terpenoid | Genus: Castilla | Family: Moraceae | Species: elastica	1
NCGC00387769-02	Gardiquimod	Toll-Like Receptor 7 agonist	0
NCGC00164584-03	Cetraxate hydrochloride	Prostaglandin Synthesis and Regulation Modulator	0

NCGC00249885-01	Estradiol acetate		0
NCGC00025230-21	SB-431542	TGF-beta receptor type-1 inhibitor	0
NCGC00163169-05	Pizotifen	Antimigraine drugs	0
NCGC00164600-03	Artesunate	Anti-malarial	0
NCGC00183013-01	Doranidazole	Apoptosis Modulator	0
NCGC00510692-02	TPX-0005	Neurotrophic Tyrosine Kinase Receptors (TRK) inhibitor	1
NCGC00386195-06	Scopine	Metabolite of anisodine	0
NCGC00378595-03	RO-4987655	Dual Specificity Mitogen-Activated Protein Kinase Kinase 1 inhibitor	0
NCGC00090874-06	Piperonyl butoxide		0
NCGC00263583-01	Temocapril hydrochloride	Angiotensin-I Converting Enzyme inhibitor	0
NCGC00480777-01	Rolapitant	Tachykinin receptor 1 antagonist	0
NCGC00386413-01	Vactosertib	TGF-beta receptor type-1 inhibitor	0
NCGC00167831-05	SN-38	DNA Topoisomerase I inhibitor	1
NCGC00016242-05	Metyrapone	Cytochrome P450 11B1 Inhibitor	0
NCGC00164604-06	Fluvastatin	HMG-CoA Reductase inhibitor	1
NCGC00274272-01	Iobenzamic acid		0
NCGC00509905-01	Eliglustat (Tartrate)	Ceramide glucosyltransferase Antagonist	0
NCGC00242505-01	Deacetyl cortivazol	Glucocorticoid Receptor agonist	1
NCGC00509945-01	CEP-40783	Tyrosine-protein kinase receptor TYRO3 inhibitor	0
NCGC00345822-02	Akt-I-1	AKT serine/threonine kinase inhibitor	0
NCGC00159336-03	Sulbactam	beta-Lactamase Inhibitor	0
NCGC00521952-01	ROMURTIDE	TNF-alpha Activator	0
NCGC00018291-03	Procaterol	beta2-Adrenoceptor Agonists	0
NCGC00159505-03	Octadecanoic acid, monoester with 1,2-propanediol	0	
NCGC00370805-01	LY-2365109 hydrochloride	Sodium and chloride glycine transporter 1 inhibitor	0
NCGC00346533-09	Daclatasvir dihydrochloride	HCV NS5A Inhibitor	1
NCGC00347286-15	GSK-126	Histone-lysine N-methyltransferase EZH2 inhibitor	0
NCGC00345790-03	Laropiprant	Prostaglandin D2 Receptor antagonist	0
NCGC00481575-01	BI-847325	Aurora kinase B inhibitor	1
NCGC00090974-06	Diethyl phthalate	Androgen receptor Antagonist	0
NCGC00178069-03	Cloperastine hydrochloride		0
NCGC00178692-04	N-METHYL (-)EPHEDRINE [1R,2S]	Beta-1 adrenergic receptor Agonist	0
NCGC00505021-01	Indotecan	DNA Topoisomerase I inhibitor	0
NCGC00093802-06	Dehydroepiandrosterone	11-beta-Hydroxysteroid Dehydrogenase Type 1 Expression inhibitor	0
NCGC00016643-13	Trioxsalen	DNA Alkylating Drug	0
NCGC00091585-03	Octocrylene	Photosensitizer	0
NCGC00181039-02	Ethyl linoleate	regulation of prostaglandin secretion Inhibitor	0
NCGC00159324-04	2-Ethylhexyl salicylate		0

NCGC00162149-07	Demeclocycline hydrochloride	Bacterial 70S ribosome Inhibitor	0
NCGC00179310-03	Cefalonium	Bacterial penicillin-binding protein Inhibitor	0
NCGC00263138-10	Regorafenib	Proto-oncogene tyrosine-protein kinase receptor Ret inhibitor	1
NCGC00485328-01	(6R)-5,6,7,8-Tetrahydrobiopterin dihydrochloride	Phenylalanine-4-hydroxylase Substrate	0
NCGC00179357-05	PREDNICARBATE	Glucocorticoid Receptor Agonist	0
NCGC00016561-05	Meticrane	Translocator protein Inhibitor	0
NCGC00507860-01	PLX-7904	Serine/threonine-protein kinase B-raf inhibitor	0
NCGC00347934-09	AGI-5198	Isocitrate Dehydrogenase [NADP] cytoplasmic (Arg132His Mutant) inhibitor	0
NCGC00016016-28	Triamterene	Heart phosphodiesterase Inhibitor	1
NCGC00095042-08	ThioTEPA	DNA Alkylating Drug	0
NCGC00181756-01	gamma-Oryzanol	lipid oxidation Inhibitor	0
NCGC00263158-07	Amuvatinib	Proto-oncogene tyrosine-protein kinase receptor Ret inhibitor	1
NCGC00183276-01	Hydrocortisone buteprate		0
NCGC00179250-03	Halofantrine	Anti-malarial	0
NCGC00485484-02	THZ-2	Cyclin-Dependent Kinase 7 inhibitor	1
NCGC00021146-09	Ketanserin	5-HT2A Antagonists?	0
NCGC00346634-02	WAY-600	mTOR Complex 1 (mTORC1) inhibitor	1
NCGC00522017-01	rac Alminoprofen	Cyclooxygenase-2 Inhibitor	0
NCGC00017343-11	RETINOL	Retinol dehydrogenase 12 Substrate	0
NCGC00263117-22	Panobinostat	Histone deacetylase 1, class I inhibitor	1
NCGC00022579-05	Carbenicillin disodium	Bacterial penicillin-binding protein Inhibitor	0
NCGC00402377-02	PCI-27483	Coagulation Factor VIIa Inhibitor	0
NCGC00346666-01	Ginkgolide A	Platelet-Activating Factor Receptor antagonist	0
NCGC00182045-03	Pentostatin	Adenosine Deaminase inhibitor	0
NCGC00183095-01	Tigecycline	glycylcycline antibiotics	0
NCGC00016508-04	Methazolamide	Carbonic anhydrase XII Inhibitor	0
NCGC00347905-02	AZ-10606120	P2X Purinoceptor 7 antagonist	0
NCGC00085849-02	MEBHYDROLIN	Histamine H1 receptor Antagonist	0
NCGC00016634-04	Acetopromazine maleate		0
NCGC00378875-01	ALK inhibitor 2	ALK tyrosine kinase receptor Inhibitor	1
NCGC00167544-02	2,2'-Methylenebis[6-(2H-benzotriazol-2-yl)-4-(1,1,3,3-tetramethylbutyl)phenol]	0	
NCGC00181116-02	Carbomycin A	Bacterial 70S ribosome Inhibitor	0
NCGC00390548-02	Exherin	Cadherin-2 antagonist	0
NCGC00509915-01	GSK-6853	Bromodomain and PHD finger containing 1 inhibitor	0
NCGC00263233-01	IPFK2	6-phosphofructo-2-kinase/fructose-2,6-bisphosphatase 2 inhibitor	0

NCGC00181912-01	Iomazenil	GABAergic synapse Modulator	0
NCGC00164581-02	Estramustine	Estrogen receptor beta Modulator	0
NCGC00159425-09	ZINC UNDECYLENATE	Fatty Acid Biosynthesis Inhibitor	0
NCGC00023064-05	Medroxyprogesterone		0
NCGC00167473-02	Capreomycin IB		0
NCGC00345447-10	3-Methyladenine	Phosphatidylinositol 3-Kinase alpha isoform inhibitor	0
NCGC00178060-03	Tolperisone hydrochloride	Voltage-gated calcium channel Inhibitor	0
NCGC00274269-01			0
NCGC00016868-12	Acemetacin	Cyclooxygenase-1/2/3 Inhibitor	0
NCGC00015818-25	Praziquantel	Anti-helmenthic	0
NCGC00378801-04	BIX02188	Dual Specificity Mitogen-Activated Protein kinase kinase 5 inhibitor	0
NCGC00384618-01		<MOA Unknown> | Class: Steroide | Genus: N/A | Family: N/A | Species: N/A	1
NCGC00015882-15	Riluzole	Short transient receptor potential channel 5 agonist	0
NCGC00016382-09	Oxethazaine		0
NCGC00384169-08	WS6	Proliferation-associated protein 2G4 Binding Agent	1
NCGC00016457-02	Naringenin	Estrogen Receptor beta agonist	0
NCGC00521078-02	Cephaloglycin	Bacterial penicillin-binding protein Inhibitor	0
NCGC00016809-07	Moricizine hydrochloride	class 1c antiarrhythmic, Na(v)1.5 channel blocker	0
NCGC00262228-03	NCGC00262228	15-hydroxyprostaglandin dehydrogenase [NAD(+)] inhibitor	0
NCGC00389572-01	FIROCOXIB	Cyclooxygenase-2 Inhibitor	0
NCGC00183049-01	Zelandopam		0
NCGC00016610-12	Tolnaftate	Fungal Squalene Monooxygenase Inhibitor	0
NCGC00181154-03	Nitarsone		0
NCGC00242458-15	Selisistat	NAD-Dependent Protein Deacetylase Sirtuin-1 inhibitor	0
NCGC00510002-01	GLPG0187	Integrin Receptor Antagonist	0
NCGC00346874-02	PD-134308	Cholecystokinin B Receptor antagonist	0
NCGC00379236-08	ML-347	ALK2 inhibitor	0
NCGC00389431-01	RIBOFLAVIN 5-PHOSPHATE SODIUM	NADH oxidation Oxidative Enzyme	0
NCGC00379061-01	Umeclidinium bromide	Muscarinic acetylcholine receptor M3 Antagonist	0
NCGC00390657-02	Lesinurad (sodium)	Solute carrier family 22 member 12 Inhibitor	0
NCGC00263175-02	Tosedostat	Aminopeptidase N inhibitor	0
NCGC00249883-01	Hydrocortamate	Glucocorticoid receptor Agonist	0
NCGC00181148-01	Metharbital	GABA-A receptor; anion channel Binding Agent	0
NCGC00095836-05	Guaiazulene	lipid oxidation Inhibitor	0
NCGC00370856-01	ATC 0065	Melanin-concentrating hormone receptor 1 Antagonist	1
NCGC00485333-01	Febrifugine dihydrochloride		1

NCGC00378936-01	Letermovir	Tripartite terminase subunit UL28 homolog Inhibitor	0
NCGC00484069-01	JNJ-42041935	Hypoxia-Inducible Factor Prolyl Hydroxylase inhibitor	0
NCGC00017236-16	Baicalein	Androgen Receptor antagonist	0
NCGC00344053-04	Purmorphamine	Smoothened Receptor agonist	0
NCGC00492474-01	Compound 22d	CKs1-SKp2 protein-protein inhibitor	1
NCGC00249914-01	Bencyclane		0
NCGC00510315-02	Finafloxacin		0
NCGC00511383-01	RAF-709	Serine/threonine-protein kinase B-raf inhibitor	1
NCGC00016777-10	Probucol	Serine Protease Hepsin inhibitor	0
NCGC00389085-04	SR-9243	LiverX Receptor (LXR) Inverse agonist	0
NCGC00248275-02	S-Hydroprene		0
NCGC00249684-04	VX-702	Mitogen-Activated Protein Kinase p38 inhibitor	0
NCGC00507888-01	NCB-1026	TRAF2 and NCK-Interacting Protein Kinase inhibitor	1
NCGC00522520-01	GNE-495	Mitogen-Activated Protein Kinase Kinase Kinase 4 inhibitor	0
NCGC00386428-07	LY-2584702	Ribosomal Protein S6 Kinase beta-1 inhibitor	0
NCGC00181104-04	Dithiazanine chloride		1
NCGC00024016-03	Hydrocortisone valerate		0
NCGC00091326-06	Acetanilide		0
NCGC00378832-01	Eldecalcitol		0
NCGC00253593-01	Bitolterol	Beta-2 adrenergic receptor Agonist	0
NCGC00164595-02	Voglibose		0
NCGC00347303-01	Englerin A	Short transient receptor potential channel 5 agonist	0
NCGC00387037-01	PF-04979064	Phosphatidylinositol 3-Kinase alpha isoform inhibitor	1
NCGC00164569-02	Capecitabine	Dihydropyrimidine Dehydrogenase inhibitor	0
NCGC00250409-02	GSK-1016790A	Transient receptor potential cation channel subfamily V member 4 agonist	0
NCGC00163469-03	Monensin sodium salt	Anti-bacterial	1
NCGC00159515-03	Eszopiclone	GABAA Receptor Modulator	0
NCGC00263559-01	Carbocysteine	regulation of mucus secretion Modulator	0
NCGC00346716-20	Dabrafenib	Serine/threonine-protein kinase B-raf inhibitor	1
NCGC00378734-08	AZD1152	Aurora kinase B inhibitor	0
NCGC00182986-01	Acetylpheneturide		0
NCGC00485987-01	Tubeimoside II		1
NCGC00021569-20	Enalapril maleate	Angiotensin-I Converting Enzyme inhibitor	0
NCGC00179599-03	Cefoperazone sodium	Cephalosporin antibiotic	0
NCGC00168110-05	Tozasertib	Aurora kinase B inhibitor	0
NCGC00505020-01	Indimitecan	DNA Topoisomerase I inhibitor	0
NCGC00168777-04	Etonogestrel	contraceptive	0

NCGC00262958-01	Bravavir		0
NCGC00025379-01	SR 33805	Ca2+ channel Antagonist	0
NCGC00346947-02	PDPK1 Inhibitor	3-Phosphoinositide Dependent Kinase 1 inhibitor	0
NCGC00483135-03	CPI-0610	Bromodomain-Containing Protein 4 (Brd4) inhibitor	1
NCGC00351596-02	AZD-3463	Insulin-like growth factor 1 receptor inhibitor	1
NCGC00510708-01	NCGC00510708	Sphingosine Kinase 1 inhibitor	0
NCGC00022882-03	Diazoxide	"Sulfonylurea receptor 1, Kir6.2 Opener"	0
NCGC00015264-13	Clomipramine hydrochloride	Sodium-dependent Serotonin Transporter inhibitor	0
NCGC00183852-01	Diponium bromide	Muscarinic acetylcholine receptor Antagonist	0
NCGC00522014-01	Zolazepam Hydrochloride		0
NCGC00507842-01	BMS-582949	Mitogen-Activated Protein Kinase p38 inhibitor	0
NCGC00371153-09	RKI-1447	Rho-associated protein kinase 1 inhibitor	0
NCGC00182060-03	l-Oxyfedrine hydrochloride	Adrenergic receptor beta Partial Agonist	0
NCGC00015242-16	Chlorothiazide	Solute carrier family 12 member 3 inhibitor	0
NCGC00016408-09	Sulfapyridine	Antibiotic	0
NCGC00095143-07	Rosuvastatin calcium (Crestor)		0
NCGC00538143-01		surfactant homeostasis Activator	0
NCGC00016542-06	Levonordefrin	Adrenergic receptor alpha-2 Agonist	0
NCGC00015554-13	ML-7	Myosin Light Chain Kinase, smooth muscle inhibitor	0
NCGC00522306-02	Valbenazine		0
NCGC00356837-03	PF-04620110	Diacylglycerol Acyltransferase Type 1 inhibitor	0
NCGC00346444-07	VE-821	Serine-protein kinase ATR inhibitor	0
NCGC00095237-14	Gambogic acid		1
NCGC00015998-06	THIOTHIXENE	dopamine secretion Inhibitor	0
NCGC00181294-01	Thiamine disulfide	HIV Infection Inhibitor	0
NCGC00094557-06	2-Chloro-4-nitrobenzamide		0
NCGC00385241-01		<MOA Unknown> | Class: Saponin | Genus: N/A | Family: N/A | Species: N/A	1
NCGC00378621-01	Imeglimin	AMP-Activated Protein Kinase (AMPK) activator	0
NCGC00346676-04	CCT-137690	Aurora kinase A inhibitor	0
NCGC00189074-12	PD-184352	Dual Specificity Mitogen-Activated Protein Kinase Kinase inhibitor	0
NCGC00178404-03	Triamcinolone		0
NCGC00016017-20	AG-1478	Epidermal Growth Factor Receptor inhibitor	0
NCGC00018256-06	Oxyphenonium bromide	Muscarinic acetylcholine receptor Antagonist	0
NCGC00159481-03	Sofalcone	Prostaglandin Synthesis and Regulation Modulator	0
NCGC00507840-01	LTX-315	Bax/Bak-regulated mitochondrial membrane permeabilizer	0
NCGC00181093-01	Metrizoate sodium		0

NCGC00387089-01	YM-511	Aromatase inhibitor	0
NCGC00017134-08	Meclocycline sulfosalicylate		0
NCGC00378657-03	TMC435350/Simeprevir		1
NCGC00246969-06	Latanoprost	Prostanoid FP receptor Agonist	0
NCGC00095133-09	Famciclovir	antiviral	0
NCGC00015442-15	Fluorouracil	Dihydropyrimidine Dehydrogenase inhibitor	0
NCGC00166144-03	2,3-Dihydroxypropyl octanoate		0
NCGC00522470-01	OSU-T315	Integrin linked kinase inhibitor	1
NCGC00182029-01	Fosfluconazole	Cytochrome P450 51 Inhibitor	0
NCGC00091501-06	Resorcinol	leukotriene B4 biosynthetic process Inhibitor	0
NCGC00346610-02	Pantothenic acid	Vitamin	0
NCGC00095123-10	Candesartan cilexetil (Atacand)		0
NCGC00091320-05	Decanoic acid	Peroxisome proliferator-activated receptor gamma Modulator	0
NCGC00016246-21	Chlorhexidine HCl	Anti-bacterial	0
NCGC00015938-05	Salmeterol xinafoate	Beta-2 adrenergic receptor Agonist	0
NCGC00013495-01	Benztropine methylsulfonate	Dopamine transporter Inhibitor	0
NCGC00178433-02	HYDROXYAMPHETAMIN E HYDROBROMIDE	Adrenergic receptor Agonist	0
NCGC00242215-02	GSK 461364 analogue I		1
NCGC00345856-01	AZD-26	AKT serine/threonine kinase inhibitor	0
NCGC00510486-01	Tinostamustine	DNA Alkylating Drug	0
NCGC00182047-14	FludarabinePhosphate	DNA Polymerase inhibitor	0
NCGC00165825-02	Milnacipran hydrochloride	Serotonin transporter Inhibitor	0
NCGC00482546-01	Ganirelix		0
NCGC00167440-02	Bosentan	"Endothelin receptor, ET-A/ET-B Antagonist"	0
NCGC00179680-05	Clonidine	alpha1-Adrenoceptor Agonists	0
NCGC00016049-07	Tetracaine hydrochloride	Sodium channel alpha subunit Blocker	0
NCGC00386373-08	R788 (Fostamatinib)	Tyrosine-protein kinase SYK inhibitor	1
NCGC00378903-01	KD-025	Rho-associated protein kinase 2 inhibitor	0
NCGC00164385-05	Clomifene citrate	Estrogen Receptor antagonist	0
NCGC00346459-07	LY-2874455	Fibroblast Growth Factor Receptor 1 inhibitor	1
NCGC00178896-05	Mepenzolate bromide	Muscarinic acetylcholine receptor M3 Antagonist	0
NCGC00263529-01	Lacidipine	Ca channel blocker - DHP class	0
NCGC00163329-03	Clopidogrel bisulfate	P2Y Purinoceptor 12 antagonist	0
NCGC00346701-07	Laquinimod	Brain-derived Neurotrophic Factor (BDNF) activator	0
NCGC00346670-03	AMG-458	Hepatocyte Growth Factor Receptor inhibitor	0
NCGC00159369-04	1-Octadecanol		0
NCGC00510160-03	SGC-2085	Histone-Arginine Methyltransferase CARM1 inhibitor	0
NCGC00094762-08	Piperazine		0
NCGC00013095-10	Geraniol	Trichomonas vaginalis Inhibitor	0

NCGC00263223-02	CAY-10626	Phosphatidylinositol 3-Kinase alpha isoform inhibitor	1
NCGC00185916-05	DASA-58	Pyruvate kinase M2 activator	0
NCGC00165924-04	(S)-Mephenytoin	Sodium channel protein type V alpha subunit Inhibitor	0
NCGC00346653-06	SCH 900776	Checkpoint kinase 1 inhibitor	1
NCGC00189076-01	Dibromopropamidine dihydrochloride	0	
NCGC00346606-02	Cyclovirobuxin D	acute myocardial ischemia drug	0
NCGC00161327-10	Calcitriol	Vitamin D3 Receptor agonist	0
NCGC00347909-06	A-839977	Purinergic receptor P2X7 inhibitor	0
NCGC00016535-19	Prostaglandin E1	Prostaglandin E2 Receptor EP2 subtype agonist	0
NCGC00522580-01	CC-115	DNA-Dependent Protein Kinase inhibitor	1
NCGC00015821-10	Piracetam	"Glutamate receptor ionotropic, AMPA 3 Positive Allosteric Modulator"	0
NCGC00161643-05	Fluorescein sodium		0
NCGC00387876-01	Birabresib	Brd2 Inhibitor	1
NCGC00480913-01	Altiratinib	Vascular Endothelial Growth Factor Receptor 2 (VEGFR-2) inhibitor	0
NCGC00095105-05	Acetrizoic acid	Solute carrier organic anion transporter family member 1B1 Activator	0
NCGC00017251-06	alpha-MANGOSTIN		1
NCGC00168114-09	IC-87114	Phosphatidylinositol 3-Kinase delta isoform inhibitor	0
NCGC00018257-16	Methocarbamol	Muscle relaxant	0
NCGC00018215-06	Acebutolol hydrochloride	Beta-1 adrenergic receptor Antagonist	0
NCGC00356590-01	Arsenic trioxide		0
NCGC00167491-14	Lenalidomide	Cereblon inhibitor	0
NCGC00160624-04	Ethopabate	folic acid biosynthetic process Modulator	0
NCGC00263606-06	Tubastatin A	Histone deacetylase 6, class IIB inhibitor	0
NCGC00182992-01	Fasoracetam	GABA-B receptor Antagonist	0
NCGC00507966-01	AMG-511	PI3K alpha Inhibitor	1
NCGC00015874-05	Quinacrine	Secretory Phospholipase A2 (sPLA2) Inhibitor	1
NCGC00346468-03	17 alpha-propionate	Androgen Receptor antagonist	0
NCGC00093358-08	Cefotaxime sodium salt	Bacterial penicillin-binding protein Inhibitor	0
NCGC00378594-02	Btk inhibitor 1 (R enantiomer hydrochloride)	1	
NCGC00263204-09	CYC-116	Aurora kinase A inhibitor	1
NCGC00016530-07	Sulfameter	Antibiotic	0
NCGC00095795-06	5,7-Dichloro-8-hydroxy-2-methylquinoline	Botulinum neurotoxin type A Inhibitor	0
NCGC00273482-01	Nafcillin sodium monohydrate		0
NCGC00510489-01	BMS-986020	Lysophosphatidic Acid Receptor 1 antagonist	0
NCGC00378604-01	ABT-333		1
NCGC00025325-03	Vapiprost hydrochloride	Prostanoid TP Antagonist	0

NCGC00162142-06	SD-169	Mitogen-Activated Protein Kinase p38 inhibitor	0
NCGC00507775-01	Midecamycin Acetate	Ribosome Inhibitor	0
NCGC00167466-04	Lomustine	DNA Alkylating Drug	0
NCGC00181167-01	Isoniazid sodium methanesulfonate	0	
NCGC00166301-04	SODIUM TETRADECYL SULFATE	Vitamin K-dependent protein S Inhibitor	0
NCGC00188433-02	Hygromycin B	Ribosome-associated ATPase Inhibitor	0
NCGC00346721-04	GW-791343	P2X Purinoceptor 7 antagonist	0
NCGC00346714-02	Ipatasertib	AKT serine/threonine kinase inhibitor	1
NCGC00263231-01	PDHK RIKEN	Pyruvate dehydrogenase (PDH) inhibitor	0
NCGC00015034-21	INO-1001	Poly [ADP-ribose] polymerase inhibitor	0
NCGC00178529-07	Levofloxacin	DNA Topoisomerase IV Inhibitor	0
NCGC00159411-02	Triethanolamine		0
NCGC00384198-02	PX-478	Hypoxia-inducible factor 1-alpha inhibitor	0
NCGC00242513-02	Maraviroc	Chemokine CCR5 Antagonist	0
NCGC00016715-04	Trapidil	C-C motif chemokine 2 Inhibitor	0
NCGC00016028-08	Tyrphostin AG 555		1
NCGC00521082-01	Mebezonium Iodide		0
NCGC00179430-04	Iocetamic acid		0
NCGC00484907-01	(-)-Scopolamine N-oxide	Acetylcholinesterase Inhibitor	0
NCGC00346667-02	Lornoxicam	Cyclooxygenase 1(COX1) inhibitor	0
NCGC00510144-01	Gestonorone		0
NCGC00263041-01			1
NCGC00346489-03	BTZ-10526043	tuberculosis pathway Inhibitor	0
NCGC00013082-04	trans-Aconitic acid	Cytoplasmic aconitate hydratase Substrate	0
NCGC00389762-01	D-(+)-MALTOSE		0
NCGC00345853-03	JNJ-28312141	Colony Stimulating Factor 1 Receptor inhibitor	0
NCGC00016064-17	Terfenadine		1
NCGC00179573-03	Tiaprofenic acid	Cyclooxygenase Inhibitor	0
NCGC00538142-01		vasoconstriction Inhibitor	0
NCGC00178145-03	Thonzylamine hydrochloride		0
NCGC00508704-02	HALOPERIDOL DECANOATE	Dopamine D4 receptor Inverse Agonist	0
NCGC00263216-02	Torin-2	mTOR Complex 1 (mTORC1) inhibitor	1
NCGC00023037-06	Pancuronium bromide	Steroid non-depolarizing Blocker	0
NCGC00163492-01	Cerulenin	Anti-fungal	0
NCGC00168248-02	Benzoylecgonine		0
NCGC00485180-01	Chromeceptin	Peroxisomal multifunctional enzyme type 2 Binding Agent	1
NCGC00163650-04	Cryptotanshinone	STAT-3 Inhibitor	0
NCGC00379005-02	Silvestrol		1
NCGC00166257-03			0
NCGC00165767-04	Cyclofenil	Estrogen receptor beta Antagonist	0

NCGC00263088-02	BAG-956	Phosphatidylinositol 3-Kinase alpha isoform inhibitor	1
NCGC00378937-01	Setiptiline	serotonin receptor signaling pathway Modulator	0
NCGC00095045-05	Sulfanitran	Eimeria tenella Inhibitor	0
NCGC00263221-03	Ezatiostat	Glutathione-S-transferase P inhibitor	0
NCGC00094881-07	Glafenine hydrochloride		0
NCGC00262588-02	Merbromin		0
NCGC00387488-03	TAK-960	Serine/threonine-protein kinase PLK1 inhibitor	1
NCGC00181088-01	Melitracen hydrochloride		0
NCGC00263184-06	MK-0752	gamma-Secretase inhibitor	0
NCGC00378933-01	Pradigastat	Diacylglycerol Acyltransferase Type 1 inhibitor	0
NCGC00016913-14	Astemizole	Histamine H1 receptor Antagonist	0
NCGC00179246-05	VIOMYCIN SULFATE	70s Ribosome Inhibitor	0
NCGC00242506-06	Bortezomib	Proteasome inhibitor	0
NCGC00161599-13	Troglitazone	Peroxisome proliferator-activated receptor gamma agonist	0
NCGC00183871-04	N-Acetyl sulfamethoxazole		0
NCGC00390222-01	XL647		1
NCGC00249417-03	Ataciguat	Soluble Guanylate Cyclase (sGC) activator	0
NCGC00522629-01	ACC-789	Vascular Endothelial Growth Factor Receptor 2 (VEGFR-2) inhibitor	0
NCGC00015149-25	Bumetanide	Solute carrier family 12 member 1 inhibitor	0
NCGC00181161-03	Ethyl salicylate		0
NCGC00345803-02	AVN944	Inosine 5'-Monophosphate Dehydrogenase 2 inhibitor	1
NCGC00016569-09	TRANEXAMIC ACID	Tissue-Type Plasminogen activator inhibitor	0
NCGC00183001-01	Tolvaptan	Vasopressin V2 receptor antagonist	0
NCGC00346654-11	INK-128	mTOR Complex 1 (mTORC1) inhibitor	0
NCGC00481652-04	Gilteritinib	Receptor-type tyrosine-protein kinase FLT3 inhibitor	1
NCGC00380798-01		Tyrosine-protein kinase SRC Inhibitor	1
NCGC00386141-07	Rigosertib (ON-01910)	Cyclin-Dependent Kinase 1 inhibitor	0
NCGC00179460-03	Medrysone	Glucocorticoid receptor Agonist	0
NCGC00161703-03	SPC-839	inhibitor of nuclear factor kappa-B kinase subunit beta inhibitor	1
NCGC00016239-04	Methantheline bromide	Histamine H2 receptor Antagonist	0
NCGC00160622-04	D-delta-Tocopherol		0
NCGC00181782-03	Sertindole	Serotonin 2c (5-HT2c) receptor Antagonist	0
NCGC00263148-07	Abiraterone	Steroid 17-alpha-hydroxylase/17,20 lyase inhibitor	0
NCGC00481611-02	ACTB-1003	Ribosomal Protein S6 Kinase alpha-1 inhibitor	1
NCGC00344564-03	Diosmin	Aryl Hydrocarbon Receptor agonist	0
NCGC00015889-11	Raloxifene hydrochloride	Selective Estrogen Receptor modulator (SERM)	0

NCGC00181149-01	3-Anilinopropan-1-ol		0
NCGC00347398-04	Ginsenoside Rb1		0
NCGC00188863-06	Dronedarone hydrochloride	Alpha-1A Adrenergic Receptor antagonist	0
NCGC00381362-01		<MOA Unknown> | Class: Polyketide | Genus: N/A | Family: N/A | Species: N/A	1
NCGC00015418-09	Ethosuximide	Voltage-dependent T-type calcium channel subunit alpha-1G inhibitor	0
NCGC00163654-01	HARRINGTONINE		1
NCGC00091835-10	Chlormethine	DNA Alkylating Drug	0
NCGC00095986-04	10-hydroxycamptothecin	DNA Topoisomerase I inhibitor	1
NCGC00484067-01	Entrectinib	Neurotrophic Tyrosine Kinase Receptors (TRK) inhibitor	1
NCGC00169844-02		<MOA Unknown> | Class: N/A | Genus: N/A | Family: N/A | Species: N/A	1
NCGC00015599-10	L-165041	PPARdelta Agonist	0
NCGC00345840-03	Merck-22-6	Caspase-3 activator	1
NCGC00522015-01	Pirlimycin Hydrochloride	Bacterial 70S ribosome Inhibitor	0
NCGC00378919-01	ST-2825	Myeloid differentiation primary response protein MyD88 Dimerization inhibitor	0
NCGC00023945-12	3'-Azido-3'-deoxythymidine	Reverse Transcriptase Inhibitor	0
NCGC00346624-10	A-66	Phosphatidylinositol 3-Kinase alpha isoform inhibitor	0
NCGC00182047-03	Fludarabine phosphate	DNA Polymerase inhibitor	0
NCGC00021147-11	Doxylamine succinate	Histamine H1 receptor Antagonist	0
NCGC00166113-04	Diphenylcyclopropenone	immune system process Activator	0
NCGC00179542-03	Brinzolamide	Carbonic anhydrase II Inhibitor	0
NCGC00408813-01	GSK205	Transient receptor potential cation channel subfamily V member 4 Antagonist	1
NCGC00142599-02	Stigmasterol	Farnesoid X receptor agonist	0
NCGC00015340-14	Desipramine hydrochloride	Serotonin transporter Inhibitor	0
NCGC00094201-03	Masoprocol	Insulin-like growth factor 1 receptor inhibitor	0
NCGC00178127-11	Ramipril	Angiotensin-I Converting Enzyme inhibitor	0
NCGC00350780-13	SCH-772984	Mitogen-Activated Protein Kinase 3 (ERK1) inhibitor	0
NCGC00346804-01	EO-1428	Mitogen-Activated Protein Kinase p38 inhibitor	0
NCGC00379253-01	DBM 1285 dihydrochloride	MAP kinase p38 alpha Inhibitor	1
NCGC00178638-13	Valaciclovir	DNA Polymerase Inhibitor	0
NCGC00182081-04	Zotepine	Histamine H1 receptor Antagonist	0
NCGC00508824-01	Ralaniten (EPI-001)	Androgen Receptor antagonist	0
NCGC00018194-08	Bendroflumethiazide	Thiazide-sensitive sodium-chloride cotransporter Inhibitor	0
NCGC00378675-01	ONO-4817	Matrix Metalloproteinase (MMP) inhibitor	0
NCGC00509995-02	Tenalisib	Phosphatidylinositol 3-Kinase gamma isoform inhibitor	0
NCGC00182971-01	Hexafluorenium dibromide	Cholinesterase Inhibitor	0
NCGC00163638-01	TUBERCIDIN		1
NCGC00346507-07	AMG-208	Hepatocyte Growth Factor Receptor inhibitor	0

NCGC00522303-02	Elagolix	Gonadotropin Releasing Hormone Receptor antagonist	0
NCGC00348363-01	ARABITOL(D)	sugar alcohol	0
NCGC00015529-15	Ibuprofen lysine	Cyclooxygenase-1/2 Inhibitor	0
NCGC00522603-01	Resatorvid	Toll-Like Receptor 4 antagonist	0
NCGC00345834-05	TAK-715	Mitogen-Activated Protein Kinase p38 inhibitor	0
NCGC00344620-03	C-646	Tubulin polymerization inhibitor	0
NCGC00016794-13	Gemfibrozil	Peroxisome proliferator-activated receptor alpha agonist	0
NCGC00167419-03	ENZACAMENE		0
NCGC00346505-02	Epothilone A	Microtubule-Stabilizing agent	0
NCGC00094748-07	Oxyphenbutazone		0
NCGC00244253-18	Ruxolitinib	Tyrosine-protein kinase JAK2 inhibitor	0
NCGC00167794-02	Ryuvidine		1
NCGC00167559-01	Androstenone		0
NCGC00510944-01	Cyclopentamine hydrochloride		0
NCGC00499786-01	XL-784	ADAM metallopeptidase domain 10 inhibitor	0
NCGC00183020-01	Minopafant	Platelet activating factor receptor Antagonist	0
NCGC00378907-01	Edoxaban tosylate monohydrate	Coagulation Factor X Inhibitor	0
NCGC00346484-04	ZSTK-474	Phosphatidylinositol 3-Kinase alpha isoform inhibitor	1
NCGC00346474-01	NMP	Bacterial Efflux Pump Inhibitor	0
NCGC00481604-01	LY-3009120	Serine/threonine-protein kinase B-raf inhibitor	1
NCGC00346488-03	MLN-8054	Aurora kinase A inhibitor	0
NCGC00182039-02	Cilazapril	Angiotensin-converting enzyme Inhibitor	0
NCGC00263574-01	Diflucortolone valerate	Annexin A1 Activator	0
NCGC00160504-01	Testosterone 17-phenylpropionate	0	
NCGC00183285-15	Nilotinib	Bcr-Abl Kinase inhibitor	0
NCGC00181784-01	Iloprost	Prostanoid IP receptor Agonist	0
NCGC00165721-16	A-83-01	TGF-beta receptor type-1 inhibitor	0
NCGC00346646-02	CP-91149	Glycogen Phosphorylase inhibitor	0
NCGC00390587-03	G-007-LK	Tankyrase-1 inhibitor	0
NCGC00025064-17	A23187, free acid		1
NCGC00346725-06	Apalutamide	Androgen Receptor antagonist	0
NCGC00373220-03	CEFMENOXIME HYDROCHLORIDE	0	
NCGC00016741-09	Tinidazole		0
NCGC00015898-28	Rolipram	Phosphodiesterase 4 inhibitor	0
NCGC00179273-03	Rimexolone	Glucocorticoid receptor Agonist	0
NCGC00370768-04	Tolimidone	Tyrosine-protein kinase Lyn activator	0
NCGC00096060-07	Puerarin		0
NCGC00496841-01	MK-4101	Smoothened Receptor antagonist	0

NCGC00162400-09	Staurosporine	Checkpoint kinase 1 inhibitor	1
NCGC00492296-02	eFT508	MAP kinase-interacting serine/threonine-protein kinase 2 inhibitor	0
NCGC00167512-04	Everolimus	Rapamyin analog mTORC inhibitor	0
NCGC00182058-03	Doxercalciferol	Vitamin D3 Receptor agonist	0
NCGC00162225-04	L-tetramisol	Nicotinic Receptor Agonist	0
NCGC00160481-02	Tioxidazole	microtubule polymerization Inhibitor	0
NCGC00346886-09	YK-4-279	EWS-FLI1 inhibitor	0
NCGC00263173-09	Rucaparib	Poly [ADP-ribose] polymerase 1 inhibitor	0
NCGC00015981-12	SU6656	Tyrosine-protein kinase Lyn inhibitor	0
NCGC00181307-01	Febantel		0
NCGC00263906-02	Rolitetracycline		0
NCGC00249907-01	Sulthiame	Carbonic anhydrase XII Inhibitor	0
NCGC00025341-06	Isradipine	Calcium Channel Blocker	0
NCGC00142611-02	D-HISTIDINE	Histidine decarboxylase Substrate	0
NCGC00483033-01	Tildipirosin	Bacterial 70S ribosome Inhibitor	0
NCGC00507881-01	Miransertib	AKT serine/threonine kinase inhibitor	0
NCGC00015704-09	Methapyrilene hydrochloride		0
NCGC00263874-02	BLEOMYCIN (bleomycin B2 shown)	0	
NCGC00182989-01	Glyclopyramide		0
NCGC00164140-07	Dioctyl sulfosuccinate sodium salt	0	
NCGC00379144-01	S 32212 hydrochloride	Serotonin 2c (5-HT2c) receptor Inverse Agonist	1
NCGC00160673-01	Propicillin		0
NCGC00092314-15	Triciribine	AKT serine/threonine kinase inhibitor	0
NCGC00183046-03	Mequitazine	Histamine H1 receptor Antagonist	0
NCGC00179435-06	Ifomide	DNA Alkylating Drug	0
NCGC00346544-09	BIX-02189	Dual Specificity Mitogen-Activated Protein Kinase Kinase 5 inhibitor	0
NCGC00018171-06	Clidinium bromide		0
NCGC00179419-05	Guanadrel sulfate	Norepinephrine transporter Partial Agonist	0
NCGC00346736-01	Testosterone cypionate	Androgen Receptor agonist	0
NCGC00186028-04	Levetiracetam	Voltage-dependent N-type calcium channel subunit alpha-1B Blocker	0
NCGC00016973-08	Letrozole	Aromatase inhibitor	0
NCGC00499575-01	HDACi-02	Histone deacetylase 1, class I inhibitor	1
NCGC00521080-01	Digitoxin 3'''-Acetate	Sodium/potassium-transporting ATPase Inhibitor	0
NCGC00016786-15	Mycophenolic acid	Inosine 5'-Monophosphate Dehydrogenase 2 inhibitor	0
NCGC00507883-01	CPI-1205	Histone-lysine N-methyltransferase EZH2 inhibitor	0
NCGC00484808-01	CPI-703	Histone Acetyltransferase p300 inhibitor	0
NCGC00014891-14	RG-108	DNA Methyltransferase (DNMT) inhibitor	0

NCGC00165952-05	XCT-790	Estrogen-Related Receptor alpha Inverse agonist	0
NCGC00016411-10	Butacaine		0
NCGC00249915-01	Bupranolol	Beta-3 adrenergic receptor Antagonist	0
NCGC00167500-02	Etretinate	Retinoic acid receptor alpha Agonist	0
NCGC00166059-02	Aluminum acetate, basic		0
NCGC00181320-01	Mersalyl acid		0
NCGC00168459-04	NCGC00168459-01	Phosphodiesterase 4 inhibitor	1
NCGC00263116-09	ICG-001	Beta-catenin/TCF interaction inhibitor	0
NCGC00481574-01	GSK-503	EZH2 Inhibitor	0
NCGC00246835-02	Porfiromycin	DNA Damaging Drug	0
NCGC00378942-03	LY-2090314	Glycogen Synthase Kinase 3 (GSK-3) inhibitor	0
NCGC00263182-04	Mocetinostat	Histone deacetylase 1, class I inhibitor	1
NCGC00015718-24	Naftopidil	Alpha-1D Adrenergic Receptor antagonist	0
NCGC00167740-02	Costunolide	Telomerase reverse transcriptase inhibitor	0
NCGC00242477-02	AC-261066	Retinoic Acid Receptor beta agonist	0
NCGC00016345-14	4-Methylumbelliferone	MAO-A Inhibitor	0
NCGC00390575-03	CUDC-427	IAP inhibitor	0
NCGC00263093-14	Apilimod mesylate	Interleukin-12 Production inhibitor	0
NCGC00016279-05	Aminohippuric acid		0
NCGC00094689-06	Guaifenesin		0
NCGC00246971-04	Polydatin	Vascular cell adhesion protein 1 Expression inhibitor	0
NCGC00024309-20	Diltiazem hydrochloride	L-Type Calcium Channel Blocker	0
NCGC00386394-01	UNC2250	Tyrosine-protein kinase Mer inhibitor	0
NCGC00159544-04	Zaldaride maleate	Calmodulin antagonist	0
NCGC00183839-01	Rapacuronium bromide	Muscarinic acetylcholine receptor M5 Negative Allosteric Modulator	0
NCGC00165957-07	Amlodipine		0
NCGC00182978-01	Tiaramide hydrochloride		0
NCGC00091400-02	Allylthiourea		0
NCGC00014482-03	Methdilazine hydrochloride	ion channel	0
NCGC00386260-02	PD-168393	Epidermal Growth Factor Receptor inhibitor	1
NCGC00016101-17	Xylometazoline hydrochloride	Alpha-2A Adrenergic Receptor agonist	0
NCGC00182034-01	Teprenone	Heat shock 70 kDa protein 1 Modulator	0
NCGC00371137-09	PluriSln 1	SCD1 Inhibitor	1
NCGC00386293-01	Rociletinib	Epidermal Growth Factor Receptor (Thr790Met Mutant) inhibitor	1
NCGC00249886-01	Prednisolone tebutate		0
NCGC00346644-04	KX-01	Tubulin polymerization inhibitor	0
NCGC00016765-09	Diflunisal	Cyclooxygenase-2 Inhibitor	0
NCGC00532508-01	DROMETRIZOLE TRISILOXANE		0
NCGC00263168-03	PHA-793887	Cyclin-Dependent Kinase 4 inhibitor	0

NCGC00018239-11	ISOETHARINE MESYLATE		0
NCGC00017147-02	CEFEPIME HYDROCHLORIDE	Bacterial penicillin-binding protein Inhibitor	0
NCGC00263098-05	Combretastatin A-4	Tubulin polymerization inhibitor	1
NCGC00390607-01	Tesevatinib	EGFR Inhibitor	1
NCGC00159351-05	Cholesterol	Transient receptor potential cation channel subfamily V member 1 Inhibitor	0
NCGC00351600-04	BMN-673	Poly [ADP-ribose] polymerase 1 inhibitor	0
NCGC00016003-10	Trihexyphenidyl hydrochloride	Muscarinic acetylcholine receptor M5 Antagonist	0
NCGC00182985-01	Trolnitrate	Soluble guanylate cyclase Activator	0
NCGC00507884-01	WNK-463	Serine/threonine-protein kinase WNK1 inhibitor	0
NCGC00092344-05	GW-0742	PPARgamma Agonist	0
NCGC00159330-04	Doxofylline	Adenosine Receptor A1 antagonist	0
NCGC00390623-02	Pacritinib	Jak2 / FLT3 Inhibitor	1
NCGC00510266-01	M2698	AKT serine/threonine kinase inhibitor	1
NCGC00263922-02	Darifenacin hydrobromide	Muscarinic acetylcholine receptor M3 Antagonist	0
NCGC00263100-02	OSI-632	Vascular Endothelial Growth Factor Receptor 2 (VEGFR-2) inhibitor	0
NCGC00182040-01	Bevantolol hydrochloride	Adrenergic receptor beta Antagonist	0
NCGC00387040-01	VTX-27	Protein kinase C theta Inhibitor	1
NCGC00163128-17	Pioglitazone hydrochloride	Peroxisome proliferator-activated receptor gamma agonist	0
NCGC00025155-01	Omacetaxine mepesuccinate	Induced Myeloid Leukemia Cell Differentiation Protein Mcl-1 Expression inhibitor	1
NCGC00015748-17	Nifedipine	Voltage-gated L-type calcium channel alpha-1C subunit Inhibitor	0
NCGC00015997-12	Taurine	Antioxidant	0
NCGC00381558-06	AZD-2461	Poly [ADP-ribose] polymerase inhibitor	0
NCGC00185748-01	Paramethasone acetate	Glucocorticoid receptor Interacts	0
NCGC00390558-02	ALW-II-41-27	Proto-oncogene tyrosine-protein kinase receptor Ret inhibitor	1
NCGC00015725-15	Nimesulide	Cyclooxygenase 2(COX2) inhibitor	0
NCGC00095709-02	Tanshinone IIA	11-beta-Hydroxysteroid Dehydrogenase Type 1 inhibitor	0
NCGC00016278-10	Mefenamic acid	Cyclooxygenase-2 Inhibitor	0
NCGC00346436-04	CCT-241533	Checkpoint kinase 2 inhibitor	1
NCGC00167547-02	Agaric acid		0
NCGC00094087-07	Mizoribine	Inosine 5'-Monophosphate Dehydrogenase inhibitor	0
NCGC00249916-01	Dimorpholamine	skeletal muscle contraction Activator	0
NCGC00346647-03	GSK-1838705A	Insulin-like growth factor 1 receptor inhibitor	0
NCGC00180892-03	CLAVULANATE LITHIUM		0
NCGC00387038-01	GDC-046	Non-receptor tyrosine-protein kinase Tyk2 inhibitor	1

NCGC00167455-02	Zofenopril		0
NCGC00090686-11	Nitrofurazone	Antibiotic	0
NCGC00522492-01	PFK-158	6-phosphofructo-2-kinase/fructose-2,6-bisphosphatase 3 inhibitor	0
NCGC00025125-18	Colchicine	Tubulin polymerization inhibitor	1
NCGC00091104-10	Aspartame	Heterodimer G-protein coupled receptor activator	0
NCGC00015823-23	Piroxicam	Cyclooxygenase Inhibitor	0
NCGC00182052-15	SRT-1720	NAD-Dependent Protein Deacetylase Sirtuin-1 activator	1
NCGC00179244-03	Dorzolamide hydrochloride	Carbonic Anhydrase 2 inhibitor	0
NCGC00242481-05	AZD-7762	Checkpoint kinase 1 inhibitor	1
NCGC00270540-02	Miglitol	Alpha-Glucosidase inhibitor	0
NCGC00346960-01	HMSL10058	JNK Inhibitor	0
NCGC00164375-03	1-Phenoxy-2-propanol		0
NCGC00185769-02	Stiripentol	"Gaba Synthesis, Release, Reuptake And Degradation Modulator"	0
NCGC00263202-06	NVP-BSK805	Tyrosine-protein kinase JAK2 inhibitor	0
NCGC00016543-13	Phenformin hydrochloride	ATP synthase inhibitor	0
NCGC00016066-14	Urapidil hydrochloride	Adrenergic receptor alpha-1 Antagonist	0
NCGC00249919-01	Nandrolone furylpropionate		0
NCGC00094740-05	NORETHYNODREL	Progesterone receptor Agonist	0
NCGC00164034-05	Triclocarban		1
NCGC00521070-01	PF-06282999	Myeloperoxidase inhibitor	0
NCGC00510488-01	EPZ-031686	Sphingomyelin phosphodiesterase 3 inhibitor	0
NCGC00183869-01	Gusperimus trihydrochloride	Heat shock protein HSP90 Binding Agent	0
NCGC00378611-03	MI-773 (SAR405838)	p53-binding protein Mdm-2 Inhibitor	1
NCGC00160575-01	Uracil mustard	DNA Interacts	0
NCGC00346830-03	5-Fluorodeoxycytidine	DNA Methyltransferase (DNMT) inhibitor	0
NCGC00016927-11	Enoxacin	Quinoline Antibiotic	0
NCGC00017030-10	Fusidic Acid (sodium salt)		0
NCGC00016055-16	Trimethoprim	Bacterial dihydrofolate reductase Inhibitor	0
NCGC00015033-03	p-Aminoclonidine hydrochloride	Alpha-2A Adrenergic Receptor agonist	0
NCGC00167561-03	D-alpha-Tocopherol succinate		0
NCGC00508876-01	CCT-244747	Checkpoint kinase 1 inhibitor	0
NCGC00016740-18	Prazosin	alpha1-Adrenoceptor Antagonists	0
NCGC00347935-08	AGI-6780	Isocitrate Dehydrogenase [NADP] mitochondrial (Arg140Gln Mutant) inhibitor	0
NCGC00261969-01	Alatrofloxacin mesylate		0
NCGC00242490-05	PD-166285	Membrane-associated tyrosine-and threonine-specific cdc2-inhibitory kinase inhibitor	0
NCGC00346674-02	Onalespib	Heat Shock Protein 90 (Hsp90) inhibitor	0
NCGC00262603-04	Idelalisib	Phosphatidylinositol 3-Kinase delta isoform inhibitor	0
NCGC00253601-01	Calcium undecylenate		0

NCGC00345843-02	AMG-25	Mast/stem cell Growth Factor Receptor Kit inhibitor	1
NCGC00390649-02	Bay-65-1942 hydrochloride	inhibitor of nuclear factor kappa-B kinase subunit beta inhibitor	0
NCGC00356746-04	GNE-618	Nicotinamide phosphoribosyltransferase inhibitor	0
NCGC00344509-02	AC-265347	Calcium-Sensing Receptor agonist	0
NCGC00024631-03	EBPC	Aldose Reductase inhibitor	0
NCGC00386231-04	Evacetrapib (LY2484595)	Cholesteryl Ester Transfer Protein inhibitor	0
NCGC00242598-10	LY-2109761	TGF-beta receptor type-1 inhibitor	0
NCGC00522639-01	BVT-3498	11-beta-Hydroxysteroid Dehydrogenase Type 1 inhibitor	0
NCGC00274062-01	Zosuquidar trihydrochloride	Multidrug resistance protein 1 inhibitor	1
NCGC00163127-04	Acitretin	Retinoic Acid Receptor RXR-alpha agonist	0
NCGC00016850-15	Bezafibrate	Peroxisome proliferator-activated receptor gamma Agonist	0
NCGC00274056-01	Risedronate sodium	Farnesyl Diphosphate Synthase inhibitor	0
NCGC00182390-05			1
NCGC00347955-03	SR-1664	PPARgamma Modulator	0
NCGC00250388-04	KRP-203	Sphingosine 1-phosphate receptor 4 agonist	0
NCGC00386423-05	AT-13148	AKT serine/threonine kinase inhibitor	1
NCGC00179247-04	Nomegestrol acetate	Androgen Receptor Antagonist	0
NCGC00386417-15	PF-06463922	ALK Tyrosine Kinase Receptor inhibitor	0
NCGC00378431-08	BQU-57	Ras-Related Protein Ral-A inhibitor	0
NCGC00015831-06	Pheniramine maleate	Histamine H1 receptor Antagonist	0
NCGC00388364-06	Molidustat	Hypoxia-Inducible Factor Prolyl Hydroxylase inhibitor	0
NCGC00346950-03	NU-6102	Cyclin-Dependent Kinase 1 inhibitor	0
NCGC00387473-03	Smad3Inhibitor,SIS3		1
NCGC00345881-04	Conivaptan hydrochloride	Vasopressin V2 receptor antagonist	0
NCGC00160628-03	Rutin trihydrate	Aldose reductase Inhibitor	0
NCGC00015697-01	Myricetin	Androgen Receptor antagonist	0
NCGC00371120-06	Dapivirine?TMC-120	Human immunodeficiency virus type 1 reverse transcriptase Inhibitor	1
NCGC00263099-07	CP-466722	ATM Kinase inhibitor	0
NCGC00021193-03	AMCINONIDE		0
NCGC00185775-01	Salidroside	beta-Amyloid (Abeta) Protein Neurotoxicity Inhibitors	0
NCGC00167469-02			0
NCGC00166147-02	Dihydrotachysterol	Vitamin D receptor Agonist	0
NCGC00185763-03	6?-Methylprednisolone 21-hemisuccinate	0	
NCGC00249927-01	Simetride		0
NCGC00183366-07	NORETHINDRONE ACETATE	Progesterone Receptor Agonist	0
NCGC00483012-01	Phytic acid	lipid oxidation Inhibitor	0
NCGC00093347-12	Mestranol	Estrogen Receptor agonist	0

NCGC00095116-05	Metaxalone		0
NCGC00242484-09	WYE-354	mTOR Complex 1 (mTORC1) inhibitor	0
NCGC00015703-06	Mecamylamine	Neuronal acetylcholine receptor; alpha4/beta2 Antagonist	0
NCGC00022858-10	Sodium cromoglycate	Mediator Release Inhibitor	0
NCGC00371001-05	PFI-3	Transcription activator BRG1 inhibitor	0
NCGC00263105-06	Turofexorate isopropyl	Farnesoid X receptor agonist	0
NCGC00178754-06	Primaquine diphosphate	antimalarial	0
NCGC00015469-09	Guanfacine hydrochloride	Alpha-2a adrenergic receptor Agonist	0
NCGC00345024-01	GSK3 Inhibitor	Glycogen Synthase Kinase 3 (GSK-3) inhibitor	0
NCGC00015959-09	Sanguinarine	Serine/threonine-protein phosphatase PP1-alpha catalytic subunit Inhibitor	1
NCGC00165833-11	Entinostat	Histone deacetylase 2, class I inhibitor	1
NCGC00018284-06	MEPIVACAINE HYDROCHLORIDE	Sodium channel protein type X alpha subunit Inhibitor	0
NCGC00161641-05	Sinomenine	Mu-Type Opioid Receptor agonist	0
NCGC00091399-01	Benzothiazole		0
NCGC00510003-01	Vaborbactam		0
NCGC00142423-05	Phlorizin	Sodium/glucose cotransporter 2 inhibitor	0
NCGC00179566-03	Ritodrine hydrochloride	beta2-Adrenoceptor Agonists	0
NCGC00016507-05	Thonzonium bromide	osteoclast development Inhibitor	0
NCGC00015833-15	Pyrazinamide	Anti-tuberculosis agent	0
NCGC00492447-02	ARS-853	GTPase KRAS (Gly12Cys Mutant) inhibitor	0
NCGC00162222-04	Cibenzoline succinate	Voltage-sensitive sodium channel Blocker	0
NCGC00164634-07	Balsalazide disodium	Peroxisome proliferator-activated receptor gamma agonist	0
NCGC00385183-01		Potassium-transporting ATPase Inhibitor	0
NCGC00025001-03	Namodenoson	Adenosine Receptor A3 agonist	0
NCGC00159509-02	Perindopril	Angiotensin-I Converting Enzyme Inhibitor	0
NCGC00263460-01	Amprolium hydrochloride	Eimeria tenella Inhibitor	0
NCGC00389219-01	Oxeladin Citrate		0
NCGC00344622-15	SGC-0946	Histone-lysine N-methyltransferase, H3 Lysine-79 Specific inhibitor	0
NCGC00346560-03	Telbivudine	DNA Polymerase Inhibitor	0
NCGC00371144-02	AWD 131-138		0
NCGC00016467-10	Digitoflavone	Alpha-Glucosidase inhibitor	0
NCGC00253737-09	Istradefylline	Adenosine Receptor A2A antagonist	0
NCGC00187598-03	Amaranth		0
NCGC00378324-03	Bruceantin	Myc proto-oncogene protein Expression inhibitor	1
NCGC00015471-09	Ganciclovir	DNA Polymerase Inhibitor	0
NCGC00017248-18	Embelin	Anti-viral	1
NCGC00263154-08	AZD-6482	Phosphatidylinositol 3-Kinase beta isoform inhibitor	0
NCGC00510907-01	Etafedrine hydrochloride	Beta-2 adrenergic receptor Agonist	0

NCGC00016383-05	Trimethadione	Voltage-gated T-type calcium channel Blocker	0
NCGC00181030-02	Uracil	Dihydropyrimidine dehydrogenase Substrate	0
NCGC00091396-05	Benzoin		0
NCGC00164127-07	4-(1,1,3,3-Tetramethylbutyl)phenol	0	
NCGC00182996-01	Pamicogrel	platelet aggregation Inhibitor	0
NCGC00094048-01	Iofetamine hydrochloride		0
NCGC00532494-01	PHENINDAMINE	Histamine H1 receptor Antagonist	0
NCGC00485891-01	Norgestimate metabolite Norelgestromin	Progesterone receptor Agonist	0
NCGC00250392-01	AR-C155858	Monocarboxylate Transporter 2 inhibitor	0
NCGC00389508-01	XL-019	Jak2 Inhibitor	1
NCGC00249898-01	Sodium 3, 4-dimethylphenyl-glyoxylate	0	
NCGC00386704-01	Mitapivat	Pyruvate kinase M2 activator	0
NCGC00507799-01	Flumethasone acetate		0
NCGC00015806-06	Pirfenidone	TGF-beta receptor type-1 inhibitor	0
NCGC00091454-07	Phenol		0
NCGC00182995-01	Etomidoline		0
NCGC00168912-02		<MOA Unknown> | Class: Steroid | Genus: Nerium | Family: Apocynaceae | Species: oleander	1
NCGC00262608-11	TG-101209	Bromodomain-Containing Protein 4 (Brd4) inhibitor	1
NCGC00021191-15	Cortisone acetate	Glucocorticoid Receptor agonist	0
NCGC00263144-09	WZ-4002	Epidermal Growth Factor Receptor (Thr790Met Mutant) inhibitor	0
NCGC00385413-01		<MOA Unknown> | Class: Steroid | Genus: Nerium | Family: Apocynaceae | Species: oleander	1
NCGC00021148-08	Metoprolol tartrate		0
NCGC00509910-02	Umbralisib	Casein Kinase 1 epsilon inhibitor	0
NCGC00346628-05	LY-2608204	Glucokinase activator	0
NCGC00016353-08	Butyl 4-aminobenzoate		1
NCGC00263949-02	Piperacillin sodium salt	Penicillin-binding protein 2B Binding Agent	0

NCGC00386425-01	Defactinib	Focal Adhesion Kinase inhibitor	1
NCGC00387841-01	GSK-2830371	Protein phosphatase 1D (PP-2Cdelta; WIP1) Allosteric Inhibitor	0
NCGC00522025-01	Mepitiostane (Contains ~15% 3a,4a-Epithio isomer)	Estrogen receptor Antagonist	0
NCGC00093588-04	cAMP	cAMP-dependent protein kinase type II-alpha regulatory subunit Activator	0
NCGC00181082-01	Benzactyzine methobromide	Muscarinic acetylcholine receptor M4 Antagonist	0
NCGC00522584-01	MKC-3946	Serine/threonine-protein kinase/endoribonuclease IRE1 inhibitor	0
NCGC00384167-11	Galunisertib	TGF-beta receptor type-1 inhibitor	0
NCGC00229704-01	Raltitrexed	Thymidylate Synthase inhibitor	0
NCGC00263197-03	BMS-5	LIM domain kinase 2 inhibitor	0
NCGC00263094-05	AMG-900	Aurora kinase A inhibitor	0
NCGC00015787-09	PINACIDIL	"Sulfonylurea receptor 2, Kir6.2 Opener"	0
NCGC00181160-01	Ecabet	Urease subunit alpha Inhibitor	0
NCGC00091519-09	p-Cresol		0
NCGC00485883-01	Forodesine (hydrochloride)	Purine nucleoside phosphorylase Inhibitor	0
NCGC00346807-05	LRRK2-IN-1	Leucine-Rich Repeat Kinase 2 inhibitor	1
NCGC00090726-30	Ribavirin	Inosine 5'-Monophosphate Dehydrogenase inhibitor	0
NCGC00091268-11	Oxytetracycline	Anti-bacterial	0
NCGC00183879-01	Bisbentiamine		0
NCGC00166022-02	D-(-)-Mandelic acid		0
NCGC00378845-01	Trelagliptin succinate		0
NCGC00013037-01	Thanite		0
NCGC00016060-14	Thapsigargin	SERCA Calcium Channel inhibitor	0
NCGC00016801-05	Levobunolol hydrochloride	Beta-2 adrenergic receptor Antagonist	0
NCGC00274059-01	Nelarabine	Antimetabolite	0
NCGC00164549-02	Dofetilide	K(V)11.1 (erg1) Channel Blocker	0
NCGC00346937-01	Ombrabulin	Tubulin polymerization inhibitor	0
NCGC00509990-02	Iberdomide	Cereblon inhibitor	0
NCGC00531749-01	ACY-775	Histone deacetylase 6, class IIB inhibitor	0
NCGC00181308-01	Sorbitan monohexadecanoate		0
NCGC00015096-27	Amiodarone hydrochloride	Voltage gated potassium channel blocker	0
NCGC00168746-02	Methyldigoxin		0
NCGC00346940-03	CGP-60474	Cyclin-Dependent Kinase 1 inhibitor	1
NCGC00092276-11	Y-27632	Rho-associated protein kinase 1 inhibitor	0
NCGC00016480-20	Kaempferol	Signal Transducer and activator of Transcription 6 inhibitor	0
NCGC00094646-04	Dibenzothiophene	Cytochrome P450 2C9 Inhibitor	0
NCGC00015385-18	Dipyridamole	Phosphodiesterase PDE3 Inhibitor	0
NCGC00249906-01	Nicoboxil	positive regulation of vasodilation Activator	0
NCGC00021141-05	Phenyltoloxamine citrate	Histamine H1 receptor Blocker	0
NCGC00024929-02	ICI-D7288	HCN [I(h)] Blocker	0

NCGC00387224-01	Ampanel	Kainate Receptor Antagonist	0
NCGC00250377-09	NU-7441	DNA-Dependent Protein Kinase inhibitor	1
NCGC00166280-02	Bismuth(III) gallate basic hydrate		0
NCGC00510749-15	Loracarbef	Bacterial penicillin-binding protein Inhibitor	0
NCGC00159325-05	Desloratadine	Histamine H1 receptor Antagonist	0
NCGC00094932-06	LANATOSIDE C		0
NCGC00161344-07	Eicosapentaenoic Acid	lipoprotein oxidation Inhibitor	0
NCGC00016781-08	Ambroxol hydrochloride	surfactant homeostasis Modulator	0
NCGC00015919-17	Sotalol	Beta-1 Adrenergic Receptor antagonist	0
NCGC00181774-02	Chrysarobin		0
NCGC00509934-01	BAY-1125976	AKT serine/threonine kinase inhibitor	0
NCGC00263532-10	Olopatadine hydrochloride	Mediator Release Inhibitor	0
NCGC00161602-06	Doxycycline	Antibiotic	0
NCGC00485035-01	IC 261		1
NCGC00178286-05	Pralidoxime chloride	Butyrylcholinesterase Activator	0
NCGC00015242-13	Chlorothiazide	Solute carrier family 12 member 3 inhibitor	0
NCGC00253586-01	Isalon		0
NCGC00015619-13	Loratadine	Histamine H1 Receptor Antagonists	0
NCGC00386295-01	KPT-276	Exportin-1 antagonist	1
NCGC00345798-03	BMS-911543	Tyrosine-protein kinase JAK2 inhibitor	0
NCGC00015199-24	Chlorambucil	DNA Damaging Drug	0
NCGC00142624-06	Lactulose	Disaccharide	0
NCGC00182042-04	Propylhexedrine (hydrochloride)	Trace amine-associated receptor 1 Agonist	0
NCGC00378991-02	Efatutazone	Peroxisome proliferator-activated receptor gamma agonist	0
NCGC00347908-01	A-740003	P2X7 Receptor Antagonist	0
NCGC00181085-01	Miripirium chloride		1
NCGC00388369-01	Prednisolone Phosphate (sodium salt)	Glucocorticoid receptor Agonist	0
NCGC00345795-04	AZD-5363	AKT serine/threonine kinase inhibitor	0
NCGC00345854-06	CEP-33779	Tyrosine-protein kinase JAK2 inhibitor	1
NCGC00482810-01	BMS-202	Drug Targeting Programmed Cell Death 1 ligand 1	0
NCGC00182030-01	Sulbenicillin disodium		0
NCGC00187911-06	PLX-4720	Serine/threonine-protein kinase B-raf (Val600Glu Mutant) inhibitor	1
NCGC00346536-06	PIK-93	Phosphatidylinositol 3-Kinase alpha isoform inhibitor	0
NCGC00166218-02	4,4'-Dinitrodiphenylurea		0
NCGC00178090-03	Pristimerin		1
NCGC00253579-01	Bolandiol dipropionate	Androgen Receptor Agonist	0
NCGC00508985-02	Eltanexor	Exportin-1 antagonist	0
NCGC00260337-02	Sodium bithionolate		1
NCGC00178480-03	(+)-Tubocurarine chloride hydrochloride	Small conductance calcium-activated potassium channel protein 2 Blocker	0

NCGC00249884-01	Sulfisoxazole acetal	Bacterial dihydropteroate synthase Inhibitor	0
NCGC00183876-03	Tenofovir disoproxil fumarate	Reverse Transcriptase Inhibitor	0
NCGC00263444-01	Metocurine iodide		0
NCGC00385704-01		<MOA Unknown> | Class: Terpenoid | Genus: N/A | Family: N/A | Species: N/A	1
NCGC00179445-03	Dydrogesterone	signaling/transport	0
NCGC00092377-04	Apafant	Platelet activating factor receptor Inhibitor	0
NCGC00160514-14	Idebenone	Calcium Channel modulator	0
NCGC00183784-18	IOX-1	Lysine-Specific Demethylase 4A inhibitor	0
NCGC00509994-02	Vadadustat	Hypoxia-Inducible Factor Prolyl Hydroxylase inhibitor	0
NCGC00389455-01	Penicillin G Sodium	Bacterial penicillin-binding protein Inhibitor	0
NCGC00183599-01			0
NCGC00480774-04	Pexidartinib	Colony Stimulating Factor 1 Receptor inhibitor	0
NCGC00181109-03	Decoquinate	Antiprotozoal agent	0
NCGC00263136-07	Pracinostat	Histone deacetylase 2, class I inhibitor	1
NCGC00262959-01	Difethialone		1
NCGC00163886-05	Fenchlorphos	Cholinesterases; ACHE & BCHE Inhibitor	0
NCGC00025012-06	AM-630	cannabinoid CB2 Antagonist	0
NCGC00263130-02	cPEPCK Inhibitor	Phosphoenolpyruvate Carboxykinase, cytosolic [GTP] inhibitor	0
NCGC00087387-05	Droxinostat	Histone deacetylase 6, class IIB inhibitor	0
NCGC00379082-01	Anidulafungin		0
NCGC00183868-02	Fentiazac		0
NCGC00345857-05	CGI-1746	Bruton's Tyrosine Kinase (BTK) inhibitor	0
NCGC00263570-06	Sivelestat sodium salt hydrate	Neutrophil Elastase inhibitor	0
NCGC00181917-01	Carbenicillin indanyl	Bacterial penicillin-binding protein Inhibitor	0
NCGC00160631-02	Famphur	Trichostrongylus axei Inhibitor	0
NCGC00094616-07	Chlorthalidone	renal sodium excretion Activator	0
NCGC00016271-05	Tolazoline hydrochloride	Adrenergic receptor alpha-1 Antagonist	0
NCGC00015252-12	Cyproheptadine hydrochloride	5-HT2B Receptor antagonist	0
NCGC00389765-01	DIBEKACIN		0
NCGC00346879-04	PNU-74654	Beta-catenin/TCF interaction inhibitor	0
NCGC00018248-24		Cyclooxygenase 2(COX2) inhibitor	0
NCGC00378617-04	XL 413 hydrochloride	Cell Division Cycle 7-Related Protein Kinase inhibitor	0
NCGC00509937-01	CBL-0137	Histone chaperone FACT inhibitor	1
NCGC00510500-01	PF-06650833	Interleukin-1 Receptor-Associated Kinase 4 inhibitor	0
NCGC00532496-01	P-T-BUTYL-M-CRESOL		0
NCGC00379003-05	WEHI-539	Apoptosis regulator Bcl-2-like protein 1 (Bcl-xL) inhibitor	0
NCGC00024728-02	L-690330	Inositol monophosphatase 1 inhibitor	0

NCGC00015073-05	Amifostine	Alkaline phosphatase placental-like Substrate	0
NCGC00181020-01	Zeaxanthin	osteoclast development Inhibitor	0
NCGC00159403-03	Nandrolone decanoate		0
NCGC00510264-02	GSK-3326595	Protein arginine N-methyltransferase 5 inhibitor	0
NCGC00390724-02	E-7449	Poly [ADP-ribose] polymerase 1 inhibitor	0
NCGC00179295-03	Meptazinol hydrochloride	Mu opioid receptor Partial Agonist	0
NCGC00092290-11	SL-327	Dual Specificity Mitogen-Activated Protein Kinase Kinase 1 inhibitor	0
NCGC00344505-01	Calindol	Calcium-Sensing Receptor agonist	0
NCGC00015999-19	Tolbutamide	"Sulfonylurea receptor 1, Kir6.2 Inhibitor"	0
NCGC00249923-01	Cholesolvin	Triglyceride Biosynthesis Inhibitor	0
NCGC00166409-02	Sodium 2,3-dimercaptopropanesulfonate monohydrate	0	
NCGC00348372-01	LINDANE	GABA-A receptor Binding Agent	0
NCGC00346954-01	TCS-2312	Histone deacetylase 1, class I inhibitor	1
NCGC00386284-05	AZD-3514	Androgen Receptor antagonist	1
NCGC00249921-01	Omoconazole		0
NCGC00378840-01	TG-100801	Proto-oncogene tyrosine-protein kinase Src inhibitor	0
NCGC00166312-02	Closantel		1
NCGC00346939-07	PF-8380	Ectonucleotide Pyrophosphatase/Phosphodiesterase Family Member 2 inhibitor	0
NCGC00250404-15	ABT-888	Poly [ADP-ribose] polymerase 1 inhibitor	0
NCGC00346878-04	SU-5402	Fibroblast Growth Factor Receptor 1 inhibitor	0
NCGC00346499-02	Ostarine	Selective Androgen Receptor modulator (SARM)	0
NCGC00507846-01	BFH-772	Vascular Endothelial Growth Factor Receptor 2 (VEGFR-2) inhibitor	0
NCGC00016309-06	Sulfathiazole	Escherichia coli Inhibitor	0
NCGC00510704-01	LY-3214996	Mitogen-Activated Protein Kinase 3 (ERK1) inhibitor	0
NCGC00016305-11	Silver sulfadiazine	Antibiotic	0
NCGC00263533-01	Penciclovir	Human herpesvirus 1 DNA polymerase Inhibitor	0
NCGC00508850-01	L-778123	Protein farnesyltransferase/geranylgeranyltransferase type-1 inhibitor	0
NCGC00344562-01	Celiprolol hydrochloride	Beta-2 adrenergic receptor Partial Agonist	0
NCGC00510494-01	RRx-001	Nitric Oxide Donor	0
NCGC00187481-08	BEZ-235	mTOR Complex 1 (mTORC1) inhibitor	0
NCGC00016866-11	Pipemidic acid		0
NCGC00249052-02	Calcium levulinate		0
NCGC00346692-03	CUDC-907	Phosphatidylinositol 3-Kinase beta isoform inhibitor	0

NCGC00162385-04	Olprinone	Phosphodiesterase III (PDE3) Inhibitor	0
NCGC00167422-01	Enflurane	Glycine receptor Positive Allosteric Modulator	0
NCGC00263137-03	RD-162	Androgen Receptor antagonist	0
NCGC00387472-01	R-268712	TGF-beta receptor type-1 inhibitor	0
NCGC00344540-01	17-BETA-ESTRADIOL 3,17-DIPROPIONATE	0	
NCGC00183106-01		Aeromonas salmonicida Inhibitor	0
NCGC00183003-01	Depolipon		0
NCGC00164543-02	Lomerizine dihydrochloride	Ca channel blocker - benzhydryl class	0
NCGC00247735-02	Varenicline tartrate	Neuronal acetylcholine receptor subunit alpha-4 partial agonist	0
NCGC00241342-03	KUC107550N	Dual-Specificity Tyrosine-(Y)-Phosphorylation Regulated Kinase 1A inhibitor	0
NCGC00179536-02	Rifampicin	DNA-Directed RNA Polymerase Inhibitor	0
NCGC00159461-08	Amprenavir	HIV Protease Inhibitors	0
NCGC00386179-02	OSI-027	mTOR Complex 1 (mTORC1) inhibitor	1
NCGC00164555-10	Entacapone	NCI-H1299 Inhibitor	0
NCGC00263189-06	JNJ-38877605	Hepatocyte Growth Factor Receptor inhibitor	0
NCGC00015321-11	Dobutamine	beta1-Adrenoceptor Antagonists	0
NCGC00016107-09	ZM-39923	Tyrosine-protein kinase JAK3 inhibitor	0
NCGC00167962-06	Quinapril hydrochloride	Angiotensin-converting enzyme Inhibitor	0
NCGC00181749-01	Cefminox sodium	Peptidoglycan biosynthesis Inhibitor	0
NCGC00181010-02	Mabuterol hydrochloride	Beta-2 adrenergic receptor Agonist	0
NCGC00242217-09	GW-843682X	Serine/threonine-protein kinase PLK1 inhibitor	1
NCGC00274274-01	Benzquinamide	Dopamine D2 receptor Modulator	0
NCGC00025330-09	SB-408124	Orexin Receptor Type 1 antagonist	0
NCGC00159564-05	Scriptaid		1
NCGC00164296-05	3-indolebutyric acid	Peroxisomal bifunctional enzyme modulator	0
NCGC00159341-18	Lamivudine	Anti-viral	0
NCGC00179291-04	Fursultiamine Hydrochloride		0
NCGC00016308-06	Isopropamide	Muscarinic acetylcholine receptor Antagonist	0
NCGC00348375-01	TILMICOSIN	Bacterial 70S ribosome Binding Agent	0
NCGC00346884-02	Eeyarestatin I	Endoplasmic reticulum associated protein degradation (ERAD) inhibitor	0
NCGC00346944-02	Y-39983	Rho-associated protein kinase 1 inhibitor	1
NCGC00015996-10	Tiapride hydrochloride	Dopamine D3 receptor Antagonist	0
NCGC00178554-03	AZLOCILLIN SODIUM	Bacterial penicillin-binding protein Inhibitor	0
NCGC00166222-04	N,N'-Dibenzylethane-1,2-diamine dihydrochloride	F-box only protein 3 Inhibitor	0
NCGC00345496-08	Lomeguatrib	Methylated-DNA--protein-cysteine methyltransferase inhibitor	0

NCGC00387725-02	A-1210477	Induced Myeloid Leukemia Cell Differentiation Protein Mcl-1 inhibitor	0
NCGC00159559-03	Eplerenone	Mineralocorticoid Receptor antagonist	0
NCGC00408796-01	EMPM	Serine/threonine-protein phosphatase 2A inhibitor	0
NCGC00013841-09	Cyclobenzaprine hydrochloride	5-HT2A Receptor Ligands	0
NCGC00346698-10	Vistusertib	mTOR Complex 1 (mTORC1) inhibitor	0
NCGC00015451-11	Flupirtine maleate	NMDA Receptor antagonist	0
NCGC00017358-15	Rotenone	NADH-Ubiquinone Oxidoreductase (Complex I) inhibitor	1
NCGC00387409-02	Ertapenem	Peptidoglycan synthase FtsI Inhibitor	0
NCGC00346565-04	Elvitegravir	Anti-viral	0
NCGC00510495-01	Amiselimod hydrochloride	Sphingosine 1-phosphate receptor 1 ligand	0
NCGC00345786-03	Compound 7	Histone deacetylase 6, class IIB inhibitor	0
NCGC00274268-01			0
NCGC00250402-05	Dapagliflozin	Sodium/glucose cotransporter 2 inhibitor	0
NCGC00378877-01	Methylnaltrexone Bromide		0
NCGC00167584-01	Nicametate citrate	cell growth Inhibitor	0
NCGC00015226-17	Mitotane	Cytochrome P450 11B1 inducer	0
NCGC00178851-03	Moxalactam disodium	Bacterial penicillin-binding protein Inhibitor	0
NCGC00094908-06	Mephenesin	Glutamate NMDA receptor Antagonist	0
NCGC00015819-17	Propafenone hydrochloride	Potassium Channel Subfamily K Member 3 Blocker	0
NCGC00389726-01	Dutasteride	Steroid 5alpha-Reductase Inhibitors	0
NCGC00241101-09	Neratinib	Epidermal Growth Factor Receptor inhibitor	1
NCGC00165733-10	Apicidin		1
NCGC00346945-06	BAY61-3606	Tyrosine-protein kinase SYK inhibitor	0
NCGC00022408-04	Flumethasone pivalate		0
NCGC00161326-08	Calcifediol (monohydrate)		0
NCGC00015558-14	Isoprenaline hydrochloride	Beta-1 Adrenergic Receptor agonist	0
NCGC00183025-01	Biliscopin		0
NCGC00346598-02	Shikimic acid	Plant biochemical intermediate	0
NCGC00091902-11	Malathion	Acetylcholinesterase Inhibitor	0
NCGC00346584-02	Asiatic acid	Glycogen Phosphorylase inhibitor	0
NCGC00387023-02	AG-13958	Vascular Endothelial Growth Factor Receptor 2 (VEGFR-2) inhibitor	0
NCGC00387776-01	GSK-3 inhibitor XIII	Glycogen synthase kinase-3 beta Inhibitor	1
NCGC00022848-25	Hydrocortisone	Glucocorticoid Receptor agonist	0
NCGC00600469-01			1
NCGC00386357-01	Caspofungin Acetate	"1,3-beta-glucan synthase Inhibitor"	0
NCGC00346690-04	SB-705498	Transient receptor potential cation channel subfamily V member 1 agonist	0
NCGC00509941-01	Mavelertinib	Epidermal Growth Factor Receptor (Thr790Met Mutant) inhibitor	0
NCGC00164620-02	Nadifloxacin	DNA gyrase Inhibitor	0

NCGC00420895-01	PU-WS-13	Endoplasmin inhibitor	0
NCGC00179532-03	Lactobionic acid	Matrix Metalloproteinase (MMP) inhibitor	0
NCGC00164572-02	Rimonabant	Cannabinoid CB1 Antagonist	0
NCGC00379156-01	YM 244769	Sodium/calcium exchanger 3 Inhibitor	1
NCGC00345851-06	GNE-477	Phosphatidylinositol 3-Kinase alpha isoform inhibitor	1
NCGC00263019-01	WAY-204688	Estrogen Receptor agonist	0
NCGC00165873-03	PS-1145	inhibitor of nuclear factor kappa B kinase subunit beta inhibitor	0
NCGC00017013-06	Thiamine chloride		0
NCGC00015508-16	Hydrochlorothiazide	Solute carrier family 12 member 3 inhibitor	0
NCGC00017326-05	(1S,9R)-beta-Hydrastine	GABA-A receptor; anion channel Antagonist	0
NCGC00482953-01	LP-533401	Tryptophan Hydroxylase 2 (TPH2) Inhibitor	0
NCGC00167737-02	Lazabemide hydrochloride	Monoamine oxidase B Inhibitor	0
NCGC00160387-04	Clothiapine	GABA-A receptor; benzodiazepine site Antagonist	0
NCGC00498438-01	RX-5902 (SUPINOXIN)	Probable ATP-dependent RNA Helicase DDX5 inhibitor	1
NCGC00167427-02	Epoprostenol sodium	Prostanoid IP receptor Agonist	0
NCGC00167506-04	Pimecrolimus	Macrolide Antibiotic	0
NCGC00344081-06	STF-62247	Autophagy inducer	0
NCGC00178213-05	Nafronyl oxalate	Serotonin 2 (5-HT2) receptor Antagonist	0
NCGC00178483-14	Limonin		0
NCGC00182065-02	Dimemorfan phosphate		0
NCGC00165909-10	TRAM-34	Intermediate Conductance Channel Blocker	0
NCGC00249937-01	Amidoflumet		0
NCGC00016344-07	5-Aminosalicylic acid	reactive oxygen species biosynthetic process Inhibitor	0
NCGC00482960-01	Xanthophyll		0
NCGC00181341-02	Emedastine difumarate	Histamine H1 receptor Antagonist	0
NCGC00263165-02	PH-797804	Mitogen-Activated Protein Kinase p38 inhibitor	0
NCGC00246387-06	Arbidol	antiinfluenza drug	0
NCGC00510884-01	Tylvalosin	Bacterial 70S ribosome Inhibitor	0
NCGC00016991-05	Proparacaine	Sodium channel protein type 10 subunit alpha Blocker	0
NCGC00024717-10	L-NAME	Nitric Oxide Synthase inhibitor	0
NCGC00249759-02	NCGC00249759	Phosphodiesterase IIII (PDE4) Inhibitor	0
NCGC00346688-04	Alectinib (CH5424802)	ALK Tyrosine Kinase Receptor inhibitor	0
NCGC00179636-04	Clindamycin hydrochloride	Antibacterial	0
NCGC00532499-01	ROMIFIDINE	Alpha-2B adrenergic receptor Agonist	0
NCGC00165782-07	GW-4869	Sphingomyelin phosphodiesterase 2 inhibitor	0
NCGC00166296-02	L-(+)-Lysine monohydrochloride	Serotonin 4 (5-HT4) receptor Antagonist	0
NCGC00159372-04	Butanedioic acid		0

NCGC00167523-03	Ormetoprim	Folate biosynthesis Inhibitor	0
NCGC00185751-06	Cefuroxime Axetil		0
NCGC00249935-01	Phenylacetylglycine dimethylamide	0	
NCGC00167795-06	PD158780		1
NCGC00168748-02	Rotigotine	Dopamine D4 receptor Agonist	0
NCGC00159384-04	6-Chlorothymol	Estrogen receptor Binding Agent	0
NCGC00346935-09	CX-6258	Serine/threonine-protein kinase pim-1 inhibitor	1
NCGC00159354-06	BORNYL ACETATE		0
NCGC00521941-01	BMS-986158	Bromodomain-Containing Protein 2 (Brd2) inhibitor	0
NCGC00262945-03	Rivaroxaban	Coagulation Factor Xa inhibitor	0
NCGC00345839-10	Baricitinib	Tyrosine-protein kinase JAK1 inhibitor	0
NCGC00346463-01	Nesbuvir	RNA-Directed RNA Polymerase Inhibitor	0
NCGC00263181-10	GSK-690693	AKT serine/threonine kinase inhibitor	0
NCGC00420878-02	Dalbavancin		0
NCGC00386206-04	Azilsartan	Type-1 angiotensin II receptor Antagonist	0
NCGC00181156-01	Oxabolone cipioncate	Androgen Receptor Agonist	0
NCGC00016522-10	Bisacodyl		1
NCGC00181795-02	Lumiracoxib	Solute carrier family 22 member 8 Inhibitor	0
NCGC00356119-03	GSK-2801	Bromodomain adjacent to zinc finger domain protein 2B inhibitor	0
NCGC00263085-12	Wnt-C59	Protein Serine O-Palmitoleoyltransferase Porcupine inhibitor	0
NCGC00347938-05	BAF-312	Sphingosine 1-phosphate receptor 5 ligand	0
NCGC00346497-03	WZ-8040	Epidermal Growth Factor Receptor (Thr790Met Mutant) inhibitor	1
NCGC00347958-03	WS-3	Receptor tyrosine-protein kinase HER3 (erbB3) inhibitor	1
NCGC00164556-02	Fudosteine	Mucin-5AC Inhibitor	0
NCGC00344561-01	Norgestimate	Progesterone receptor Agonist	0
NCGC00178714-04	Topiramate	Na channel blocker	0
NCGC00261107-01	Cilnidipine	Voltage-gated N-type calcium channel alpha-1B subunit Inhibitor	0
NCGC00018200-12	Phenolphthalein	store-operated calcium entry Blocker	0
NCGC00164576-04	Lopinavir	HIV Protease Inhibitors	0
NCGC00091512-04	(2E)-3-Phenylprop-2-enal	Peroxisome proliferator-activated receptor gamma Agonist	0
NCGC00188399-06	Fingolimod hydrochloride	Sphingosine 1-phosphate receptor 5 agonist	1
NCGC00262195-03	R-1487	Mitogen-Activated Protein Kinase p38 inhibitor	0
NCGC00386332-03	Losmapimod	Mitogen-Activated Protein Kinase p38 inhibitor	0
NCGC00015693-13	Mitoxantrone	DNA Topoisomerase II inhibitor	1
NCGC00016059-13	Thioridazine	Serotonin 2c (5-HT2c) receptor Antagonist	0
NCGC00160508-03	Isosorbide		0
NCGC00250397-04	NVP-XAV-939	Tankyrase-1 inhibitor	0

NCGC00510701-01	CCT251236	Heat Shock Factor Protein 1 inhibitor	1
NCGC00182973-01	Chromonar hydrochloride	Platelet Aggregation (Plug Formation) Inhibitor	0
NCGC00386301-06	BMS-833923	Smoothened Receptor antagonist	0
NCGC00378575-03	Dofequidar	Multidrug resistance protein 1 inhibitor	0
NCGC00181342-02	Imidapril hydrochloride		0
NCGC00346541-04	XL-765	mTOR Complex 1 (mTORC1) inhibitor	0
NCGC00346825-02	10074-G5	Myc proto-oncogene protein Expression inhibitor	0
NCGC00262542-10		Anti-malarial	0
NCGC00167982-04	Pirarubicin	DNA Topoisomerase II inhibitor	0
NCGC00263530-01	Lafutidine	Histamine H2 Receptor Antagonists	0
NCGC00016229-14	Propylthiouracil	Thyroid peroxidase inhibitor	0
NCGC00181776-02	Dicyclopentamethylenethiuram disulfide	1	
NCGC00015257-14	Clofibrate	Peroxisome proliferator-activated receptor alpha agonist	0
NCGC00499577-01	JAK3i	Tyrosine-protein kinase JAK3 inhibitor	0
NCGC00498439-01	LY-3177833	Cell Division Cycle 7-Related Protein Kinase inhibitor	0
NCGC00093644-07	Amsacrine	DNA Intercalating Drug	1
NCGC00370833-02	NS-304	platelet aggregation Inhibitor	0
NCGC00499427-02	GSK-481	Receptor-interacting serine/threonine-protein kinase 1 inhibitor	0
NCGC00159518-02	Rabeprazole	H+/K+-ATPase inhibitor	0
NCGC00347939-08	BAM-7	Apoptosis regulator BAX activator	0
NCGC00263579-01	rac Nebivolol hydrochloride	beta1-Adrenoceptor Antagonists	0
NCGC00263225-01	AG-041R	gastrin/CCKB receptor Antagonist	0
NCGC00346578-08	OSI-420	Epidermal Growth Factor Receptor inhibitor	0
NCGC00345800-03	CVT-6883	Adenosine Receptor A2B antagonist	0
NCGC00182868-16	Daporinad	Nicotinamide phosphoribosyltransferase inhibitor	0
NCGC00262928-01	Clevidipine	Voltage-gated L-type calcium channel Blocker	0
NCGC00509932-01	Derazantinib	Fibroblast Growth Factor Receptor 1 inhibitor	0
NCGC00024027-09	Methysergide	Serotonin 7 (5-HT7) receptor Antagonist	0
NCGC00095158-04	Secnidazole	antiprotozoal agent	0
NCGC00164447-01	Ciprofibrate	PPARalpha Agonist	0
NCGC00379014-01	Cariprazine	Serotonin 1a (5-HT1a) receptor Partial Agonist	0
NCGC00015156-15	Baclofen	GABA-B receptor Agonist	0
NCGC00346579-03	PIK-293	Phosphatidylinositol 3-Kinase delta isoform inhibitor	0
NCGC00178913-14	Celastrol	Heat Shock Protein 90 (Hsp90) inhibitor	1
NCGC00017254-05	Cyanein	Golgi-Specific Brefeldin A-Resistance Guanine Nucleotide Exchange Factor 1 inhibitor	0

NCGC00510526-01	CHZ868	Tyrosine-protein kinase JAK2 inhibitor	0
NCGC00389735-01	SALINOMYCIN, SODIUM	Anticoccidial/Antibacterial	1
NCGC00179359-04	Penbutolol sulfate		0
NCGC00345852-03	BS-194	Cyclin-Dependent Kinase 1 inhibitor	1
NCGC00164253-04	LITHIUM CITRATE		0
NCGC00015095-15	Amitriptyline hydrochloride	Sodium-dependent serotonin transporter Inhibitor	0
NCGC00510233-01	BMS-986205	Indoleamine 2,3-dioxygenase 1 inhibitor	0
NCGC00015144-05	O6-Benzylguanine	Methylated-DNA--protein-cysteine methyltransferase inhibitor	0
NCGC00262943-01	Tocanfil		0
NCGC00018099-17	Dyphylline	Phosphodiesterase 4B inhibitor	0
NCGC00378692-06	Ulixertinib	Mitogen-Activated Protein Kinase 3 (ERK1) inhibitor	0
NCGC00508865-03	Ezutromid	Utrophin Expression Enhancer	0
NCGC00262929-01	BIBR-1048	Thrombin Inhibitor	0
NCGC00271513-02	Lofexidine	Adrenergic receptor alpha-2 Agonist	0
NCGC00344533-01	Nalpha-Acetyl-L-glutamine		0
NCGC00510492-02	Anlotinib	Vascular Endothelial Growth Factor Receptor 3 (VEGFR-3) inhibitor	1
NCGC00182980-01	Fominoben hydrochloride		0
NCGC00480771-01	ONO-4059 analog	BTK Inhibitor	0
NCGC00511388-01	Itacitinib	Tyrosine-protein kinase JAK1 inhibitor	0
NCGC00250400-05	Crizotinib	ALK Tyrosine Kinase Receptor inhibitor	1
NCGC00024683-23	Parthenolide	NF-kappaB (NFKB) Activation inhibitor	0
NCGC00178395-04	Piperidolate hydrochloride		0
NCGC00182048-01	Pipothiazine palmitate	Dopamine D2 receptor Antagonist	0
NCGC00159456-30	Imatinib	Bcr-Abl Kinase inhibitor	0
NCGC00091563-12	Thiram		1
NCGC00016961-06	Atovaquone	Synergistic with proguanil, mitochondrial sensitizer (cytochrome bc1 complex)	1
NCGC00378390-01	Compound 2	LIM domain kinase 2 inhibitor	0
NCGC00185747-02	Niceritrol	Hydroxycarboxylic acid receptor 2 Agonist	0
NCGC00242478-04	Alvespimycin hydrochloride	Heat Shock Protein 90 (Hsp90) inhibitor	1
NCGC00167801-02	CL-82198	Matrix Metalloproteinase-13 inhibitor	0
NCGC00160546-02	Haloxon	Acetylcholinesterase Inhibitor	0
NCGC00522052-01	Ciforadenant	Adenosine Receptor A2A antagonist	0
NCGC00485412-01	Ro 41-5253	Retinoic acid receptor gamma Antagonist	1
NCGC00159437-03	Clobutinol hydrochloride	HERG Inhibitor	0
NCGC00379220-07	SR-2211	Nuclear receptor ROR-gamma Inverse agonist	0
NCGC00319021-01		DNA synthesis involved in DNA replication Activator	0
NCGC00274061-01	Trilostane	Estrogen Receptor beta modulator	0
NCGC00250405-15	AZD-8055	mTOR Complex 1 (mTORC1) inhibitor	1
NCGC00482904-01	Capromorelin	Ghrelin receptor Agonist	0

NCGC00014352-10	GANT-61	Sonic Hedgehog protein inhibitor	0
NCGC00159476-09	Tribromsalan		1
NCGC00096077-06	Olanzapine	5-HT2A Receptor antagonist	0
NCGC00166105-04	BENZYL NICOTINATE		0
NCGC00015408-05	RACEPHEDRINE HYDROCHLORIDE	Norepinephrine Transporter Inhibitor	0
NCGC00015150-07	Betaine	Nitric Oxide Donor	0
NCGC00081778-05	SID-7969543	Steroidogenic factor 1 antagonist	0
NCGC00264001-02	CALCIUM GLUCEPTATE		0
NCGC00481653-02	AMG-319	Phosphatidylinositol 3-Kinase delta isoform inhibitor	0
NCGC00181118-01	Sulfabromomethazine		0
NCGC00386306-06	Spebrutinib (AVL-292)	Bruton's Tyrosine Kinase (BTK) inhibitor	1
NCGC00015147-04	Benzamil	Epithelial Sodium Channels (ENaC) Blocker	0
NCGC00389841-01	LINACLOTIDE (1 mg/ml)	Heat-stable enterotoxin receptor Agonist	0
NCGC00091038-04	Sodium deoxycholate monohydrate	0	
NCGC00386665-07	Bemcentinib	AXL Kinase inhibitor	1
NCGC00166413-02	Argatroban	Thrombin inhibitor	0
NCGC00160482-02	Sulfalene	Bacterial dihydropteroate synthase Inhibitor	0
NCGC00510505-01	WT-161	Histone deacetylase 6, class IIB inhibitor	0
NCGC00378997-07	Micafunginsodium		0
NCGC00181794-01	Dimethyl(2-phenoxyethyl)-2-thenylammonium iodide	acetylcholine metabolic process Modulator	0
NCGC00018121-07	Proxyphylline	"3',5'-cyclic phosphodiesterase Inhibitor"	0
NCGC00167471-02	Anethole trithione		0
NCGC00510354-01	RUSKI-201	Protein-cysteine N-palmitoyltransferase HHAT inhibitor	0
NCGC00390655-01	PTC-691	Polycomb Complex Protein BMI-1 inhibitor	0
NCGC00163532-06	Daidzin	Aldehyde Dehydrogenase-2 inhibitor	0
NCGC00015647-31	Nocodazole	Tubulin polymerization inhibitor	0
NCGC00345883-07	DESVENLAFAXINE SUCCINATE	Serotonin transporter Inhibitor	0
NCGC00159570-14	RITA	Thioredoxin inhibitor	0
NCGC00142598-07	beta-Sitosterol	Neutrophil Elastase (Leukocyte Elastase) Release inhibitor	0
NCGC00015049-24	Apigenin	Ornithine Decarboxylase inhibitor	0
NCGC00344520-01	L-Valine	tryptophan transport Inhibitor	0
NCGC00345842-03	GSK-25	Rho-associated protein kinase 1 inhibitor	1
NCGC00482940-01	CEP28122	ALK Inhibitor	0
NCGC00380287-01	Cytochalasin E from Aspergillus clavatus	<MOA Unknown> | Class: polyketide/AS | Genus: N/A | Family: N/A | Species: N/A	1
NCGC00263153-07	AR-42	Histone deacetylase 2, class I inhibitor	1
NCGC00379071-02	SRT-2104	NAD-Dependent Protein Deacetylase Sirtuin-1 activator	0
NCGC00160513-01	Testosterone decanoate	Androgen Receptor Agonist	0

NCGC00509936-02	CCT-251545	Cyclin-Dependent Kinase 19 inhibitor	0
NCGC00025357-04	Nicorandil	K(ATP) Channel Activator	0
NCGC00532506-01	SODIUM LAURYL SULFOACETATE	0	
NCGC00242054-15	SJN-2511	TGF-beta receptor type-1 inhibitor	0
NCGC00250381-02	AZ-23	Neurotrophic Tyrosine Kinase Receptors (NTRK) inhibitor	1
NCGC00384664-01		<MOA Unknown> | Class: macrolide | Genus: N/A | Family: N/A | Species: N/A	1
NCGC00091151-04	Phenoxyacetic acid	cell proliferation Activator	0
NCGC00016283-04	Diphemanil methylsulfate	antimitotic	0
NCGC00346657-02	MK-5108	Aurora A Inhibitor	0
NCGC00248247-02	Dimethyl maleate	NFkappaB-inducing kinase Inhibitor	0
NCGC00390564-04	GS7340		0
NCGC00092284-04	AHPN	Retinoic Acid Receptor gamma agonist	1
NCGC00242056-04	IKK-3 Inhibitor IX	inhibitor of nuclear factor kappa-B kinase subunit epsilon inhibitor	0
NCGC00091034-10	Captan		0
NCGC00167519-02	Ramatroban	Thromboxane A2 Receptor antagonist	0
NCGC00166213-02	2-Octyl-1-dodecanol		0
NCGC00511392-01	WEHI-345	Receptor-interacting serine/threonine-protein kinase 2 inhibitor	0
NCGC00178579-04	Levobupivacaine hydrochloride	Sodium channel protein type IV alpha subunit Blocker	0
NCGC00387999-01	AD57 (hydrochloride)		1
NCGC00274079-01	Sodium urate		0
NCGC00159412-04	Tromethamine	calcium ion transport Inhibitor	0
NCGC00379039-02	Tiotropium (Bromide)	Muscarinic M3 Antagonists	0
NCGC00249890-01	Mebutamate		0
NCGC00166006-02	L-Cystine	Cystine/glutamate transporter Substrate	0
NCGC00345838-04	GNE-493	Phosphatidylinositol 3-Kinase alpha isoform inhibitor	1
NCGC00018258-10	Econazole nitrate	Anti-fungal	0
NCGC00379246-01	Phortress (NSC-710305)	Aryl Hydrocarbon Receptor agonist	0
NCGC00167768-11	KI-8751	Vascular Endothelial Growth Factor Receptor 2 (VEGFR-2) inhibitor	0
NCGC00345855-06	Filgotinib	Tyrosine-protein kinase JAK1 inhibitor	0
NCGC00264110-02	AN-2690		0
NCGC00160580-02	2-Hydroxy-N-(4-hydroxyphenyl)-benzamide	Ribonucleoside-diphosphate reductase M2 chain Inhibitor	0
NCGC00346448-03	Tariquidar	Multidrug resistance protein 1 inhibitor	0
NCGC00091112-15	Methylrosaniline chloride	Cyclin-Dependent Kinase inhibitor 1B Degradation inhibitor	1
NCGC00507875-01	XMD8-87	Activated CDC42 kinase 1 inhibitor	0
NCGC00249902-01	Epsiprantel	calcium ion homeostasis Inhibitor	0
NCGC00507864-01	GDC-0084	Phosphatidylinositol 3-Kinase alpha isoform inhibitor	1
NCGC00263236-02	SM-164	IAP inhibitor	0

NCGC00510940-01	Kebuzone	Cyclooxygenase Inhibitor	0
NCGC00090827-07	Guaiacol	intestine smooth muscle contraction Inhibitor	0
NCGC00386261-01	XL-888	Heat Shock Protein 90 (Hsp90) inhibitor	0
NCGC00378631-01	Boceprevir	"Hepatitis C virus serine protease, NS3/NS4A Inhibitor"	0
NCGC00390634-03	LY-2857785	Cyclin-Dependent Kinase 9 inhibitor	1
NCGC00346503-01	Perifosine	PI3K kinase Activator	0
NCGC00166139-03	3-Hydroxy-4-butyrophenetidide		0
NCGC00263102-06	Degrasyn	Signal Transducer and activator of Transcription 3 inhibitor	1
NCGC00511370-02	ACY-738	Histone deacetylase 6, class IIB inhibitor	1
NCGC00180572-02		<MOA Unknown> | Class: trichothecene | Genus: Fusarium ? | Family: N/A | Species: N/A	1
NCGC00346702-05	Capmatinib	Hepatocyte Growth Factor Receptor inhibitor	0
NCGC00185753-04	Etoposide Phosphate		0
NCGC00094619-06	Chloramphenicol sodium succinate	Bacterial 70S ribosome Inhibitor	0
NCGC00263180-14	Trametinib	Dual Specificity Mitogen-Activated Protein Kinase Kinase 1 inhibitor	0
NCGC00351549-05	GSK-J2	Lysine-Specific Demethylase 6A inhibitor	0
NCGC00343773-03	YW3-56	Protein-Arginine Deiminase Type-4 inhibitor	0
NCGC00181771-02	Ibutilide fumarate	Voltage dependent L-type calcium channel Activator	0
NCGC00180461-11	HYDROQUINIDINE		0
NCGC00164575-06	Telithromycin		0
NCGC00344550-03	Stanozolol	Androgen Receptor agonist	0
NCGC00165770-04	Cardiogenol	Cardiomyocytes Differentiation Initiator	0
NCGC00093350-05	Amikacin	30S ribosomal protein S12 Inhibitor	0
NCGC00095239-05	1,3,5-Trimethoxybenzene		0
NCGC00480922-01	SKLB4771	Receptor-type tyrosine-protein kinase FLT3 inhibitor	0
NCGC00253613-01	Anileridine		0
NCGC00371151-06	Balicatib	Cathepsin K inhibitor	0
NCGC00015225-08	Chelerythrine chloride	P2X Purinoceptor 7 antagonist	0
NCGC00091595-07	Sodium 2-phenylphenate tetrahydrate	0	
NCGC00522278-01	Oglufanide	VEGF Inhibitor	0
NCGC00165816-04	KI-16425	Lysophosphatidic Acid Receptor 1 antagonist	0
NCGC00262939-01	Cycotiamine		0
NCGC00016819-05	Gliquidone	Cytochrome P450 3A4 Substrate	0
NCGC00242495-18	PF-562271	Focal Adhesion Kinase inhibitor	0
NCGC00163505-09	Go-6983	Protein Kinase C inhibitor	0
NCGC00162399-13	KN-93	Calcium/calmodulin-dependent protein kinase kinase 2 inhibitor	1

NCGC00263101-07	CP-724714	Receptor tyrosine-protein kinase NEU (HER2; erbB2) inhibitor	0
NCGC00381751-04	Filanesib	Kinesin-Like Protein KIF11 inhibitor	0
NCGC00253572-01	Embutramide		0
NCGC00485671-01	TAPI-2 acetate salt	ADAM17 Inhibitor	1
NCGC00475728-01	AZD-8186	Phosphatidylinositol 3-Kinase beta isoform inhibitor	0
NCGC00018302-07	Trichlormethiazide	Solute carrier family 12 member 3 inhibitor	0
NCGC00080392-05	Phosmet	Acetylcholinesterase Inhibitor	0
NCGC00015478-17	GW-5074	Raf Kinase C Inhibitor	0
NCGC00261966-01			0
NCGC00015500-20	Haloperidol	Dopamine D2 Antagonist	0
NCGC00263200-02	RO495	Non-receptor tyrosine-protein kinase Tyk2 inhibitor	1
NCGC00506803-01	Belotecan	DNA Topoisomerase I inhibitor	0
NCGC00509999-02	Seletalisib	Phosphatidylinositol 3-Kinase delta isoform inhibitor	0
NCGC00481573-01	Pexmetinib	Mitogen-Activated Protein Kinase p38 inhibitor	0
NCGC00095129-06	Valdecoxib	Cyclooxygenase-2 Inhibitor	0
NCGC00014873-05	HLI-373989	E3 Ubiquitin-Protein Ligase Mdm2 (Hdm2) inhibitor	0
NCGC00091125-07	Gallic acid	intrinsic apoptotic signaling pathway in response to oxidative stress Inhibitor	0
NCGC00346467-01	VCH-916		0
NCGC00025103-05	Monastrol	Kinesin-Like Protein KIF11 inhibitor	0
NCGC00346493-03	AT-9283	Aurora kinase B inhibitor	0
NCGC00018269-05	Trimebutine maleate		0
NCGC00091231-04	Quinidine hydrochloride monohydrate	Antimalarial Agents; Cytochrome P450 Cyp2d6 Inhibitors	0
NCGC00016490-12	Flufenamic acid	Cyclooxygenase-2 Inhibitor	0
NCGC00386427-01	Poziotinib	Receptor tyrosine-protein kinase HER4 (erbB4) inhibitor	1
NCGC00386162-01	Ammonium Glycyrrhizinate	Thrombin Inhibitors	0
NCGC00015769-14	Strophantine octahydrate	Na+/K+ ATPase inhibitor	0
NCGC00159340-05	Tazobactam sodium	Bacterial beta-lactamase TEM Inhibitor	0
NCGC00016981-05	Troleandomycin	70S ribosome Inhibitor	0
NCGC00091823-09	Chrysophanol	Vascular Endothelial Growth Factor Receptor 2 (VEGFR-2) inhibitor	0
NCGC00386723-01	GSK-2256294A	Bifunctional epoxide hydrolase 2 inhibitor	0
NCGC00091398-01	Nitrobenzene	Glutathione reductase Inhibitor	0
NCGC00178840-12	Cloxacillin sodium	beta-lactam antibiotic	0
NCGC00346453-03	BMS-754807	Insulin-like growth factor 1 receptor inhibitor	0
NCGC00381723-02	Taltirelin	Thyrotropin-Releasing Hormone (TRH) Analogs	0
NCGC00186054-01	Tannic acid	Beta-secretase 1 Inhibitor	1
NCGC00167553-02	Norphenylephrine hydrochloride	Adrenergic receptor alpha-1 Agonist	0

NCGC00021925-03	Prednisolone acetate	Glucocorticoid steroid	0
NCGC00124338-01			1
NCGC00345850-02	Bentamapimod	Mitogen-Activated protein kinase 8 (JNK) inhibitor	1
NCGC00387878-04	GSK-2879552	Lysine-Specific Histone Demethylase 1A inhibitor	0
NCGC00160424-02	Clobenzorex hydrochloride		0
NCGC00386129-03	ADW-742	Insulin-like growth factor 1 receptor inhibitor	0
NCGC00507862-01	LDC4297 (LDC044297)		1
NCGC00015430-06	Fenoterol hydrobromide		0
NCGC00164605-02	Ampiroxicam	Cyclooxygenase Inhibitor	0
NCGC00346932-04	SB-3CT	Matrix Metalloproteinase-2 inhibitor	0
NCGC00024956-17	PD-153035	Epidermal Growth Factor Receptor inhibitor	0
NCGC00016797-11	Amoxicillin sodium	Anti-bacterial	0
NCGC00346675-02	AT-406	IAP inhibitor	0
NCGC00386131-05	Brivanib Alaninate (BMS-582664)		0
NCGC00164598-03	CEFPODOXIME PROXETIL		0
NCGC00090797-28	Digoxin	Nuclear receptor ROR-gamma antagonist	1
NCGC00522516-01	MIR96-IN-1	microRNA-96 inhibitor	0
NCGC00263122-02	MK-0354	Hydroxycarboxylic Acid Receptor 2 Partial agonist	0
NCGC00107508-04	GSK-5959	Bromodomain and PHD finger containing 1 inhibitor	0
NCGC00274171-10	RUC-4	Integrin alphaIIbbeta3 (Fibrinogen gpIIb/IIIa) antagonist	0
NCGC00346655-02	Linagliptin	Dipeptidyl Peptidase IV inhibitor	0
NCGC00387826-01	Deltarasin	Retinal rod rhodopsin-sensitive cGMP 3',5'-cyclic phosphodiesterase subunit delta inhibitor	0
NCGC00095086-04	PERICIAZINE	Dopamine D2 receptor Antagonist	0
NCGC00183862-01	Tolrestat	Aldose Reductase inhibitor	0
NCGC00344481-01	PRP01-012	Tyrosine-protein kinase Yes inhibitor	0
NCGC00025091-11	U-73122	Phospholipase C inhibitor	0
NCGC00346483-02	AC-480	Receptor tyrosine-protein kinase HER4 (erbB4) inhibitor	0
NCGC00017352-22	Resveratrol	NF-kappaB (NFKB) modulator	0
NCGC00386545-01	Ciclesonide	Glucocorticoid receptor Agonist	0
NCGC00274276-01		Beta-2 adrenergic receptor Agonist	0
NCGC00386347-02	Ospemifene	Selective Estrogen Receptor modulator (SERM)	0
NCGC00508823-01	BG-15a		0
NCGC00345085-02	(-)-Indolactam V	Protein Kinase C activator	1
NCGC00181132-01	Halofuginone hydrochloride		0
NCGC00164057-03	2-Bromo-2-nitro-1,3-propanediol	reactive oxygen species biosynthetic process Activator	0
NCGC00274271-01	Dimethisteron		0

NCGC00378634-01	Seocalcitol	Vitamin D Analog	0
NCGC00509984-01	Omapatrilat	Neprilysin Inhibitor	0
NCGC00167562-01	Buquinolate		0
NCGC00178220-05	Dipyrone	Prostaglandin G/H synthase 1 Inhibitor	0
NCGC00263185-09	Dacomitinib	Epidermal Growth Factor Receptor inhibitor	1
NCGC00159379-04	BETA-HYDROXYETHYL SALICYLATE	0	
NCGC00179541-03	Carteolol hydrochloride	Adrenergic receptor beta Partial Agonist	0
NCGC00370879-01	(R)-DRF053 dihydrochloride		1
NCGC00371019-02	PF-04418948	Prostaglandin E2 Receptor EP2 subtype antagonist	0
NCGC00167465-02	Calcipotriene	Vitamin D receptor Activator	0
NCGC00015383-07			0
NCGC00159376-04	Methylparaben	canonical glycolysis Inhibitor	0
NCGC00347949-11	KY-02111	Beta-catenin/TCF interaction inhibitor	0
NCGC00351597-01	EPZ-5676	Histone-lysine N-methyltransferase, H3 Lysine-79 Specific inhibitor	0
NCGC00015948-10	Spironolactone		0
NCGC00016261-08	Pyridoxine	Oxidative Stress Inhibitor	0
NCGC00386313-08	Berzosertib	Serine-protein kinase ATR inhibitor	0
NCGC00402250-03	AS-1940477	Mitogen-Activated Protein Kinase p38 inhibitor	0
NCGC00510527-02	GSK-2982772	Receptor-interacting serine/threonine-protein kinase 1 inhibitor	0
NCGC00092288-05	(-)-Blebbistatin	Myosin II ATPase Activity inhibitor	0
NCGC00164617-14	Adapalene	Retinoic Acid Receptor beta agonist	0
NCGC00013724-07	(-)-Scopolamine methyl bromide		0
NCGC00164593-02	Enalapril acid		0
NCGC00508861-01	GSK-2981278	Nuclear receptor ROR-gamma Inverse agonist	0
NCGC00346549-08	AT-7867	AKT serine/threonine kinase inhibitor	0
NCGC00016889-06	Tenoxicam	non-steroidal antiinflammatory	0
NCGC00249892-01	Lexiscan	Adenosine A2a receptor Agonist	0
NCGC00183015-01	Temoporfin	cell proliferation Inhibitor	0
NCGC00015149-16	Bumetanide	Solute carrier family 12 member 1 inhibitor	0
NCGC00016362-05	Benzonatate	Slowly adapting pulmonary stretch receptor Inhibitor	0
NCGC00522550-01	EMD-534085	Kinesin-Like Protein KIF11 inhibitor	0
NCGC00510683-01	PF-04937319	Glucokinase activator	0
NCGC00346486-03	Abexinostat	Histone deacetylase 1, class I inhibitor	1
NCGC00386364-01	Lomitapide	Microsomal triglyceride transfer protein large subunit Inhibitor	1
NCGC00166125-02	Meglumine	Amino sugar	0
NCGC00273984-04	SB-747651-A	Ribosomal Protein S6 Kinase alpha-5 inhibitor	0
NCGC00016255-10	SULFINPYRAZONE	Canalicular Multispecific Organic Anion Transporter 1 Modulator	0

NCGC00344513-02	J113397	ORL1 (OP4, NOP) Antagonist	0
NCGC00037856-01	Medronic acid		0
NCGC00388389-01	N-acetyl-D-Glucosamine		0
NCGC00161345-07	DOCONEXENT	Voltage-gated L-type calcium channel Modulator	0
NCGC00163540-01	Hesperetin	Transient receptor potential cation channel subfamily M member 3 antagonist	0
NCGC00522453-01	MLN9708	Proteasome subunit beta type-5 Inhibitor	1
NCGC00177979-05	Azelastine hydrochloride	Histamine H1 Receptor Antagonists	0
NCGC00510235-01	H3B-6527	Fibroblast Growth Factor Receptor 4 inhibitor	0
NCGC00159423-03	Phytonadione	Vitamin K epoxide reductase complex subunit 1 Substrate	0
NCGC00164560-05	ABACAVIR SULFATE		1
NCGC00378889-01	PF-04971729	Sodium/glucose cotransporter 2 Inhibitor	0
NCGC00090708-11	OXYQUINOLINE HEMISULFATE	Trichophyton interdigitale Inhibitor	0
NCGC00485986-01	Tubeimoside I	Wnt signaling pathway Inhibitor	1
NCGC00387042-02	AZD-1208	Serine/threonine-protein kinase pim-1 inhibitor	0
NCGC00166005-02	L-Cysteine methyl ester hydrochloride	0	
NCGC00261977-03	Nimorazole	Radiosensitizer	0
NCGC00420698-02	BPTES	Glutaminase inhibitor	0
NCGC00094916-06	Suprofen	Prostaglandin G/H synthase 2 Inhibitor	0
NCGC00165865-03	Penfluridol	Dopamine receptor Blocker	1
NCGC00091325-09	Dichlorophen	"Indoleamine 2,3-dioxygenase Inhibitor"	0
NCGC00263096-16	Iniparib	Poly [ADP-ribose] polymerase 1 inhibitor	0
NCGC00345858-03	Takeda-6d	Serine/threonine-protein kinase B-raf inhibitor	0
NCGC00390241-07	LX1606 (Hippurate)	Tryptophan 5-Hydroxylase 1 inhibitor	0
NCGC00346677-02	CT-98014	Glycogen Synthase Kinase 3 (GSK-3) inhibitor	0
NCGC00390255-01	Maslinic acid	Glycogen Phosphorylase inhibitor	0
NCGC00166307-07	Calciferol	Vitamin D receptor Agonist	0
NCGC00161827-01		Xanthine dehydrogenase Binding Agent	0
NCGC00167546-03	S-(5 -Adenosyl)-L-methionine p-toluenesulfonate salt	S-adenosylmethionine synthase isoform type-2 Binding Agent	0
NCGC00346883-02	6-Bromoindirubin-3'-acetoxime	GSK-3 Inhibitor	1
NCGC00378563-01	LY-2334737	Ribonucleoside-Diphosphate Reductase inhibitor	0
NCGC00015508-23	Hydrochlorothiazide	Solute carrier family 12 member 3 inhibitor	0
NCGC00015007-14	R-(+)-Atenolol	Beta-2 adrenergic receptor Antagonist	0
NCGC00164590-02	Drospirenone	Mineralocorticoid Receptor antagonist	0
NCGC00160396-04	Pravadoline	Cannabinoid Receptor 1 agonist	0
NCGC00024994-12	Selegiline hydrochloride	Amine oxidase B inhibitor	0
NCGC00247954-03	SSR-504734	Sodium and chloride glycine transporter 1 inhibitor	0

NCGC00016393-07	2,2'-Dihydroxy-4-methoxybenzophenone	0	
NCGC00188346-18	PP242	mTOR Complex 1 (mTORC1) inhibitor	1
NCGC00532512-01	HYDROCORTISONE ACEPONATE	0	
NCGC00178734-10	Sitagliptin	Dipeptidyl Peptidase IV inhibitor	0
NCGC00183873-05	Granisetron hydrochloride		0
NCGC00532507-01	INOSITOL NIACINATE		0
NCGC00261972-01			0
NCGC00262957-01	Tobicillin		0
NCGC00344538-01	1,3-Propanedisulfonic acid disodium salt	0	
NCGC00167532-02	Balofloxacin	Anti-bacterial	0
NCGC00159373-04	Thymol	Aldose reductase Inhibitor	0
NCGC00024901-04	BD-1047	Sigma non-opioid intracellular receptor 1 antagonist	0
NCGC00509912-01	BLU-554	Fibroblast Growth Factor Receptor 4 inhibitor	0
NCGC00096095-02	8-Cl-Ado	Telomerase reverse transcriptase inhibitor	0
NCGC00179358-04	Methacycline hydrochloride	Tetracycline Antibiotic	0
NCGC00346806-06	Endoxifen	Selective Estrogen Receptor modulator (SERM)	0
NCGC00042523-03	N-Oxydiethylenebenzothiazole-2-sulfenamide	0	
NCGC00016889-15	Tenoxicam	non-steroidal antiinflammatory	0
NCGC00181297-01	Mebrofenin		0
NCGC00018181-12	Nalidixic acid	Quinoline Antibiotic	0
NCGC00263186-04	SGI-1776	Serine/threonine-protein kinase pim-1 inhibitor	0
NCGC00188866-01	NCGC00188866	Rac1 GTPase inhibitor	0
NCGC00249925-01	Tilmacoxib	Cyclooxygenase-2 Inhibitor	0
NCGC00164554-06	Mupirocin		0
NCGC00181767-12	Tolcapone	Catechol-O-Methyltransferase inhibitor	0
NCGC00273481-01	Metixene hydrochloride	Muscarinic acetylcholine receptor Antagonist	0
NCGC00510693-01	TAK-659	Tyrosine-protein kinase SYK inhibitor	0
NCGC00386267-03	(R)-Lansoprazole	H+/K+-ATPase Inhibitor	0
NCGC00164603-04	Ebastine	Histamine H1 receptor Antagonist	0
NCGC00181300-01	Riboflavin tetrabutyrate	platelet aggregation Inhibitor	0
NCGC00346719-03	LY-2811376	beta-Secretase Inhibitor	0
NCGC00390618-03	UNC2025 (hydrochloride)	Tyrosine-protein kinase Mer inhibitor	1
NCGC00016258-06	Menadione	Fatty Acid Synthase inhibitor	0
NCGC00015462-22	Glipizide	K(ATP) Channel activator	0
NCGC00092321-03	Tetrabenazine Mesylate	Vesicular Monoamine Transporter 2 (VMAT2) Inhibitor	0
NCGC00480770-01	MK-3102	Dipeptidyl peptidase IV Inhibitor	0
NCGC00346741-02	Travoprost	Prostanoid TP Agonist	0

NCGC00015061-29	Acyclovir		0
NCGC00370903-02	PD 161570	Fibroblast growth factor receptor 1 Antagonist	1
NCGC00015452-20	Flutamide	Androgen Receptor antagonist	0
NCGC00509862-02	Fluralaner		0
NCGC00167488-03	Sorafenib	Vascular Endothelial Growth Factor Receptor 3 (VEGFR-3) inhibitor	1
NCGC00372530-03	NCGC00372530	L-Lactate Dehydrogenase A chain inhibitor	0
NCGC00015970-10	Sulindac	Prostaglandin G/H synthase 2 inhibitor	0
NCGC00242249-05	SB-590885	Serine/threonine-protein kinase B-raf inhibitor	0
NCGC00185859-01			1
NCGC00022653-09	Cefazolin	Bacterial penicillin-binding protein Inhibitor	0
NCGC00380921-01		<MOA Unknown> | Class: Saponin | Genus: N/A | Family: N/A | Species: N/A	1
NCGC00390546-03	ATN-161	Integrin alphavbeta3 (Vitronectin) antagonist	0
NCGC00091219-08	DEET		0
NCGC00475723-01	AM-5992	Cyclin-Dependent Kinase 4 inhibitor	0
NCGC00346566-10	Roflumilast	Phosphodiesterase 4D inhibitor	0
NCGC00016957-05	Aceclofenac	Cyclooxygenase-2 Inhibitor	0
NCGC00164578-06	Etoricoxib	Cyclooxygenase 2(COX2) inhibitor	0
NCGC00345805-01	STX-0119	Signal Transducer and activator of Transcription 3 inhibitor	1
NCGC00372956-01	Roniciclib	Cyclin-Dependent Kinase 1 inhibitor	0
NCGC00346531-02	Oligomycin A	ATP synthase inhibitor	0
NCGC00181026-02	Robenidine	Eimeria tenella Inhibitor	0
NCGC00378853-02	Atrasentan	Endothelin-1 Receptor antagonist	0
NCGC00263128-02	PD-0220245	Chemokine CXCR1/CXCR2 Receptor (IL-8 Receptor) antagonist	0
NCGC00274082-01	Sodium dodecyl sulfate		0
NCGC00346626-04	CHIR-124	Checkpoint kinase 1 inhibitor	1
NCGC00274038-02	AZ-TAK1 Inhibitor	Mitogen-Activated Protein Kinase Kinase Kinase 7 inhibitor	0
NCGC00386387-02	Buclizine HCl	Histamine H1 receptor Antagonist	0
NCGC00178830-05	Isosorbide dinitrate		0
NCGC00164155-02	Benzylparaben	Estrogen receptor alpha Agonist	0
NCGC00186037-02	Ibandronate sodium hydrate	Farnesyl Pyrophosphate Synthase inhibitor	0
NCGC00178566-03	ENOXOLONE	15-hydroxyprostaglandin dehydrogenase inhibitor	0
NCGC00179297-04	Azapropazone	interleukin-1 alpha production Inhibitor	0
NCGC00179642-05	Isoconazole nitrate	Antifungal Agent	0
NCGC00387775-03	D-942	AMP-Activated Protein Kinase (AMPK) activator	0
NCGC00508901-01	GLPG-0974	Free Fatty Acid Receptor 2 antagonist	0
NCGC00250403-11	Linifanib	Vascular Endothelial Growth Factor Receptor 2 (VEGFR-2) inhibitor	0

NCGC00484062-02	Solcitinib	Tyrosine-protein kinase JAK1 inhibitor	0
NCGC00345083-01	Stauprimide	Protein kinase C inhibitor	1
NCGC00167524-02	Amorolfine hydrochloride	Antifungal Agent	0
NCGC00370919-02	Prinaberel	Estrogen Receptor beta agonist	0
NCGC00016322-13	Dapsone	Bacterial dihydropteroate synthase Inhibitor	0
NCGC00389778-03	NafamostatMesylate	Tryptase Inhibitors	0
NCGC00090753-06	Azithromycin	Anti-bacterial	0
NCGC00168853-03	Lupeol	Tumor necrosis factor alpha Production inhibitor	0
NCGC00016311-32	Phentolamine mesilate	Alpha-1A Adrenergic Receptor antagonist	0
NCGC00379060-01	NVP-LCQ195	Cyclin-Dependent Kinase 1 inhibitor	0
NCGC00484085-01	KPT-9274	Nicotinamide Phosphoribosyltransferase inhibitor	1
NCGC00164551-05	Biapenem		0
NCGC00160450-03	4-Acetylaminophenylacetic acid		0
NCGC00387877-01	Voruciclib	Cyclin-Dependent Kinase 4 inhibitor	0
NCGC00181168-01	Trifluomeprazine 2-butenedioate		0
NCGC00483118-01	SRT-3025	NAD-Dependent Protein Deacetylase Sirtuin-1 activator	0
NCGC00164499-03	DL-alpha Tocopheryl nicotinate	platelet aggregation Inhibitor	0
NCGC00186046-02	Caroverine	NMDA Receptor antagonist	0
NCGC00178826-03	Glucosamine hydrochloride	Solute carrier organic anion transporter family member 1B3 Inhibitor	0
NCGC00390779-02	Brivaracetam	Synaptic vesicle glycoprotein 2A Modulator	0
NCGC00094363-07	D-SERINE	Serine palmitoyltransferase 1 Substrate	0
NCGC00015323-11	Decamethonium bromide	Muscle-type nicotinic acetylcholine receptor Partial Agonist	0
NCGC00346710-03	Bindarit	NF-kappaB (NFKB) modulator	0
NCGC00346574-04	Lexibulin hydrochloride	Tubulin polymerization inhibitor	0
NCGC00378765-02	MK-0812	C-C chemokine receptor type 2 antagonist	0
NCGC00164549-12	Dofetilide	K(V)11.1 (erg1) Channel Blocker	0
NCGC00182543-02	Xipamide	renal sodium excretion Activator	0
NCGC00168783-01	Diisopropyl fluorophosphate	Acetylcholinesterase Inhibitor	0
NCGC00475739-01	PF-4989216	Phosphatidylinositol 3-Kinase alpha isoform inhibitor	1
NCGC00488783-01	Lemborexant	Orexin Receptor Type 1 antagonist	0
NCGC00485261-01	Methylstat	Lysine-specific demethylase 4E inhibitor	0
NCGC00509864-02	ORNIPRESSIN		0
NCGC00263192-04	Silmitasertib	Casein Kinase 2 inhibitor	1
NCGC00179475-03	Halcinonide		0
NCGC00179245-04	Alcuronium chloride		0
NCGC00511386-01	RO8994	E3 Ubiquitin-Protein Ligase Mdm2 (Hdm2) inhibitor	1
NCGC00181111-05	Tamibarotene	Retinoic Acid Receptor alpha antagonist	0
NCGC00390261-01	Indisulam	Carbonic Anhydrase 9 inhibitor	0
NCGC00183878-01	Zaltoprofen	Cyclooxygenase Inhibitor	0

NCGC00241104-09	Doramapimod	Mitogen-Activated Protein Kinase 11 inhibitor	0
NCGC00408859-01	Cetilistat	Pancreatic lipase Inhibitor	0
NCGC00015860-06	Prilocaine hydrochloride	reactive oxygen species biosynthetic process Inhibitor	0
NCGC00522578-01	Etrasimod	Sphingosine 1-phosphate receptor 5 agonist	0
NCGC00402337-02	BGP-15	Insulin Sensitizers	0
NCGC00025158-02	(S)-(+)-Dimethindene maleate	Histamine H1 receptor Antagonist	0
NCGC00347937-02	AR-7	Retinoic Acid Receptor alpha antagonist	0
NCGC00263539-01	Ziprasidone	5-HT2A Antagonists?	0
NCGC00346478-06	AST-1306	Epidermal Growth Factor Receptor inhibitor	1
NCGC00246958-03	NSC-207895	E3 Ubiquitin-Protein Ligase Mdm4 (Hdm4) inhibitor	0
NCGC00159453-14	Zileuton	Arachidonate 5-Lipoxygenase inhibitor	0
NCGC00167805-03	Ivachtin	Caspase-3 inhibitor	0
NCGC00249611-05	Ixazomib	Proteasome inhibitor	1
NCGC00483922-01	AZD-3759	Epidermal Growth Factor Receptor inhibitor	0
NCGC00016611-10	Bufexamac	non-steroidal antiinflammatory	0
NCGC00509942-01	EOS-200271	Indoleamine 2,3-dioxygenase 1 inhibitor	0
NCGC00160584-01	Chlorphenesin		0
NCGC00510878-01	Apaziquone	DNA Alkylating Drug	0
NCGC00263281-01	GSK-1995010	Fatty Acid Synthase inhibitor	0
NCGC00263617-13	M-344	Histone deacetylase 1, class I inhibitor	1
NCGC00094872-13	Piperine	Transient receptor potential cation channel subfamily V member 1 agonist	0
NCGC00015606-06	LOBELINE HYDROCHLORIDE	Synaptic vesicular amine transporter Inhibitor	0
NCGC00344555-01	Proscillaridin	Steroid	0
NCGC00162383-05	Vancomycin hydrochloride	Anti-bacterial	0
NCGC00242051-10	GW-2580	Colony Stimulating Factor 1 Receptor inhibitor	0
NCGC00016959-06	Ciprofloxacin	DNA Topoisomerase IV Inhibitor	0
NCGC00159474-03	Fluprednisolone	Glucocorticoid receptor Agonist	0
NCGC00015992-01	Tyrphostin A9		1
NCGC00508859-01	NAMPT-IN-1	Nicotinamide Phosphoribosyltransferase inhibitor	1
NCGC00388284-07	Darunavir (Ethanolate)	Hiv Protease Inhibitors	0
NCGC00386177-01	Esomeprazole Sodium	H+/K+-ATPase inhibitor	0
NCGC00346671-06	Anacetrapib	Cholesteryl Ester Transfer Protein inhibitor	0
NCGC00485293-01	Cytochalasin H from Phomopsis sp.	cytoskeleton organization Inhibitor	1
NCGC00538148-01			0
NCGC00249372-02			1
NCGC00263910-02	Aminopentamide sulfate	Muscarinic acetylcholine receptor Antagonist	0
NCGC00015300-14	Vanoxeamine	Sodium-dependent Dopamine Transporter inhibitor	1

NCGC00181016-19	Bexarotene	Retinoic Acid Receptor RXR-alpha agonist	1
NCGC00161825-08	GSK-3965	LiverX Receptor (LXR) agonist	0
NCGC00179392-03	Beclomethasone		0
NCGC00263131-05	PF-3845	Fatty Acid Amide Hydrolase inhibitor	0
NCGC00018107-05	2-Thiosalicylic acid	prostaglandin biosynthetic process Inhibitor	0
NCGC00179408-03	Iodixanol		0
NCGC00510443-01	Incyclinide	Matrix Metalloproteinase-9 inhibitor	1
NCGC00346959-11	HMSL10038	Tyrosine-protein kinase Lck inhibitor	0
NCGC00167403-06	Pluripotin	Mitogen-Activated Protein Kinase Kinase Kinase Kinase 2 inhibitor	1
NCGC00159462-20	Ritonavir		0
NCGC00090903-01	Sulfasalazine	Arachidonate 5-Lipoxygenase inhibitor	0
NCGC00091532-06	Clorophene		0
NCGC00182054-03	Aranidipine	Voltage-gated calcium channel Antagonist	0
NCGC00016686-07	Natamycin	Antifungal Agent	0
NCGC00263214-02	HC-067047	Transient receptor potential cation channel subfamily V member 4 antagonist	0
NCGC00015955-16	Salbutamol sulfate	Beta-2 Adrenergic Receptor agonist	0
NCGC00247878-08	NVP-AUY922	Heat Shock Protein 90 (Hsp90) inhibitor	0
NCGC00390740-04	Avibactam sodium		0
NCGC00018172-09	Indapamide	Carbonic Anhydrase 7 inhibitor	0
NCGC00263132-03	PF-477736	Checkpoint kinase 1 inhibitor	0
NCGC00181040-02	dl-Coniine	Nicotinic acetylcholine receptor alpha1/beta1/gamma/delta Agonist	0
NCGC00016347-07	Benzthiazide	Thiazide-sensitive sodium-chloride cotransporter Inhibitor	0
NCGC00183864-01	Adesulfone sodium	Dihydropteroate synthase 1 Inhibitor	0
NCGC00346529-03	Safinamide	Sodium channel inhibitor	0
NCGC00160444-02	Clofenamide	Carbonic anhydrase Inhibitor	0
NCGC00356073-13	RVX-208	Bromodomain-Containing Protein 2 (Brd2) inhibitor	0
NCGC00179652-04	Nalbuphine hydrochloride	Kappa opioid receptor Partial Agonist	0
NCGC00091023-07	CETYLPYRIDINIUM CHLORIDE		1
NCGC00481542-04			1
NCGC00163489-05	Shikonin	Tumor necrosis factor Expression inhibitor	1
NCGC00018237-09	Trichlorfon	Butyrylcholinesterase Inhibitor	0
NCGC00016329-07	Phenindione	Vitamin k epoxide reductase complex subunit 1 isoform 1 Inhibitor	0
NCGC00018153-10	Dienestrol	Estrogen Receptor agonist	0
NCGC00390702-02	Efinaconazole	Lanosterol 14-alpha demethylase Inhibitor	0
NCGC00263123-02	MK-0767	Peroxisome proliferator-activated receptor alpha agonist	0
NCGC00024762-06	Tesmilifene hydrochloride	Estrogen Receptor antagonist	0
NCGC00095606-02	(2S,5R)-5-Methyl-2-(propan-2-yl)cyclohexanone	TNF-alpha Inhibitor	0
NCGC00379053-02	Desmopressin (Acetate)	Vasopressin receptor Agonist	0
NCGC00263191-02	PHA-690509	Cyclin-Dependent Kinase 2 inhibitor	1

NCGC00016879-06	Torsemide	Loop Diuretics	0
NCGC00142605-05	Salicin	Antiinflammatory agent	0
NCGC00163699-10	Fumagillin		0
NCGC00166124-03	Benzyl cinnamate		0
NCGC00186006-10	Nefiracetam	Gamma-aminobutyric acid receptor subunit alpha-1 modulator	0
NCGC00263560-01	6-Aminopenicillanic acid		0
NCGC00262961-01	Cromoglicate lisetil	mast cell activation Inhibitor	0
NCGC00345836-13	SMI-4a	Serine/threonine-protein kinase pim-1 inhibitor	0
NCGC00018100-16	Fasudil hydrochloride	Rho-associated protein kinase 1 inhibitor	0
NCGC00166300-02	Pyridoxal phosphate hydrate	P2X purinoceptor 2 Antagonist	0
NCGC00344630-02	Marizomib	Proteasome inhibitor	1
NCGC00347064-06	KUC107871N	Valosin-containing protein (VCP) p97 / Transitional endoplasmic reticulum ATPase inhibitor	1
NCGC00164462-01	Pyriproxyfen	juvenile hormone metabolic process Modulator	0
NCGC00241113-04	Orantinib	Vascular Endothelial Growth Factor Receptor 2 (VEGFR-2) inhibitor	0
NCGC00182051-05	R-406	Tyrosine-protein kinase SYK inhibitor	1
NCGC00346963-01	HMSL10083	CSF1R (c-FMS) Inhibitor	0
NCGC00015701-06	Metyrosine	Tyrosine 3-hydroxylase Inhibitor	0
NCGC00016384-15	Sulfisoxazole	Antibiotic	0
NCGC00522572-01	GDC-0339	Serine/threonine-protein kinase pim-1 inhibitor	0
NCGC00178376-05	Sulfaquinoxaline		0
NCGC00346506-05	LY-2886721	Beta-Secretase 1 inhibitor	0
NCGC00162288-07	Valproic acid sodium salt	GSK-3 Inhibitor	0
NCGC00484061-01	APTO-253	Myc proto-oncogene protein Expression inhibitor	0
NCGC00014911-03	Cucurbitacin I	Signal Transducer and activator of Transcription 3 inhibitor	1
NCGC00163450-06	Ilomastat (GM6001, Galardin)	Matrix Metalloproteinase (MMP) inhibitor	0
NCGC00183050-01	Bethanidine sulfate (2:1)	adrenergic receptor signaling pathway involved in heart process Inhibitor	0
NCGC00095048-06	Pipobroman	DNA Crosslinking Agent	0
NCGC00167420-01	Perflenapent		0
NCGC00181770-02	Garenoxacin mesylate hydrate	Topoisomerase IV Inhibitor	0
MLS000543798-03			1
NCGC00183283-01	Iomeprol		0
NCGC00162179-08	cis-Flupentixol		0
NCGC00496921-02	GSK-963	Receptor-interacting serine/threonine-protein kinase 1 inhibitor	0
NCGC00380855-01		<MOA Unknown> | Class: steroid | Genus: Thevetia | Family: Apocynaceae | Species: plumeriaefolia	1
NCGC00370899-04	Golgicide A		1

NCGC00024928-23	Tamoxifen	Selective Estrogen Receptor modulator (SERM)	0
NCGC00346709-02	20-hydroxyecdysone	reduces expression of cPEPCK	0
NCGC00096122-05	Thioinosine	Serine/threonine-protein kinase N1 Inhibitor	0
NCGC00160594-03	IPROHEPTINE	Histamine H1 receptor Antagonist	0
NCGC00408838-01	Piroheptine Hydrochloride	Muscarinic acetylcholine receptor Antagonist	0
NCGC00181335-02	Fosfestrol tetrasodium	estrogen-related receptor gamma Inverse Agonist	0
NCGC00178852-04	Tobramycin	30S Ribosomal Protein Inhibitor	0
NCGC00346580-04	Vinflunine (Tartrate)	Tubulin polymerization inhibitor	1
NCGC00481403-01	AZD-3264	inhibitor of nuclear factor kappa B kinase subunit beta inhibitor	0
NCGC00077915-02	ML-119	Tyrosine-protein phosphatase non-receptor type 11 inhibitor	0
NCGC00386908-01			1
NCGC00090851-14	5-Azacitidine	DNA Methyltransferase (DNMT) inhibitor	1
NCGC00182991-01	Glybuzole	insulin secretion Activator	0
NCGC00370823-03	Pitolisant (hydrochloride)	Histamine H3 receptor Inverse Agonist	0
NCGC00345846-01	TG-46	Tyrosine-protein kinase JAK2 inhibitor	1
NCGC00166035-05	Demecolcine	Tubulin polymerization inhibitor	0
NCGC00015611-18	Lidocaine	Free Fatty Acid Receptor 1 antagonist	0
NCGC00386818-01	ML327	CDH1 Expression Enhancers	0
NCGC00181762-01	Nequinate	Eimeria tenella Inhibitor	0
NCGC00167460-03	Ceftizoxim??Na	Bacterial Penicillin-binding Protein Inhibitor	0
NCGC00166308-02	Difloxacin hydrochloride	Bacterial DNA gyrase Inhibitor	0
NCGC00481596-01	PLX-647	Mast/stem cell Growth Factor Receptor Kit inhibitor	0
NCGC00179009-06	Phenethicillin potassium salt	Bacterial penicillin-binding protein Inhibitor	0
NCGC00180889-03	Flunixin meglumine	Prostaglandin G/H synthase 2 Inhibitor	0
NCGC00248905-02	(1S,2R,5S)-(+)-Menthol	Transient receptor potential cation channel subfamily A member 1 Antagonist	0
NCGC00159370-10	Docosanol	Anti-Herpes Simplex Virus Drug	0
NCGC00091281-10	Daminozide	Lysine-specific Demethylase 2A inhibitor	0
NCGC00507798-01	Drostanolone propionate	Androgen Receptor Agonist	0
NCGC00161419-06	Trimetrexate	Dihydrofolate Reductase inhibitor	1
NCGC00522464-01	Lusutrombopag	Thrombopoietin receptor Agonist	0
NCGC00179344-04	Pivmecillinam		0
NCGC00346805-02	XRP-44X	Tubulin polymerization inhibitor	0
NCGC00160454-01	Felbinac ethyl		0
NCGC00179360-05	Tridihexethyl chloride	Muscarinic acetylcholine receptor M3 Antagonist	0
NCGC00025176-02	Etomidate	GABAA Receptor Modulator	0
NCGC00346972-08	PF-543	Sphingosine Kinase 1 inhibitor	0
NCGC00065890-14	Nevirapine	Reverse Transcriptase Inhibitor	0

NCGC00090782-06	Nelfinavir mesylate	HIV Protease Inhibitors	0
NCGC00183281-03	Valeric acid		0
NCGC00379012-01	CVT-313	Cyclin-Dependent Kinase 1 inhibitor	0
NCGC00164126-01	Octyl gallate		1
NCGC00384525-01		<MOA Unknown> | Class: Terpenoid | Genus: N/A | Family: N/A | Species: N/A	1
NCGC00178065-03	TENIPOSIDE	DNA Topoisomerase II Inhibitors	1
NCGC00178255-02	QUININE ETHYL CARBONATE	Ferriprotoporphyrin IX Antagonist	0
NCGC00096010-07	Thiostrepton from Streptomyces azureus	0	
NCGC00178417-03	Ribostamycin sulfate	Protein disulfide-isomerase Inhibitor	0
NCGC00381562-02	Tazemetostat	Histone-lysine N-methyltransferase EZH2 inhibitor	0
NCGC00371105-01	Efonidipine hydrochloride monoethanolate	Voltage-gated T-type calcium channel Blocker	0
NCGC00021382-07	ERYTHROMYCIN ETHYLSUCCINATE	Bacterial 70S ribosome Binding Agent	0
NCGC00387801-02	AMG-232	E3 Ubiquitin-Protein Ligase Mdm2 (Hdm2) inhibitor	0
NCGC00510528-01	MT-DADMe-ImmA	S-methyl-5'-thioadenosine phosphorylase inhibitor	0
NCGC00166000-04	Iohexol		0
NCGC00092310-06	Amisulpride	Dopamine Receptor D2 antagonist	0
NCGC00095129-15	Valdecoxib	Cyclooxygenase-2 Inhibitor	0
NCGC00481606-01	AZD-5597		1
NCGC00015009-23	4-Aminopyridine	Voltage-gated potassium channel subunit Kv1.3 Blocker	0
NCGC00249891-01	Bromodiphenhydramine	Histamine H1 receptor Antagonist	0
NCGC00402296-03	NDMC101	Dipeptidyl peptidase IV Inhibitor	1
NCGC00160654-06	Ethylparaben		0
NCGC00091330-07	Anthralin		0
NCGC00178575-05	Morantel tartrate	Neuronal acetylcholine receptor; alpha3/beta2 Agonist	0
NCGC00263232-01	GLS-968	Glutaminase inhibitor	0
NCGC00522037-01	Oxitropium Bromide	Muscarinic acetylcholine receptor Antagonist	0
NCGC00346619-03	PP-121	Platelet-derived growth factor receptor inhibitor	1
NCGC00475742-01	TAS-116	Heat Shock Protein 90 (Hsp90) inhibitor	0
NCGC00346602-01	Lappaconitine	Sodium channel protein type 1 subunit alpha Blocker	0
NCGC00163468-12	Mitomycin C	DNA Alkylating Drug	0
NCGC00015159-13	Betaxolol HCl	Beta-1 Adrenergic Receptor antagonist	0
NCGC00347801-02		<MOA Unknown> | Class: Terpenoid | Genus: N/A | Family: N/A | Species: N/A	1
NCGC00522040-01	Carpipramine Dihydrochloride		1
NCGC00091390-04	4-Methoxyphenol	Tyrosinase Substrate	0
NCGC00183836-01	Emiglitate	Maltase-glucoamylase Inhibitor	0
NCGC00253587-02	Sitaxentan	Endothelin-1 Receptor antagonist	0

NCGC00091505-07	Nitrofurantoin	Probable pyruvate-flavodoxin oxidoreductase Substrate	0
NCGC00521067-01	BMS-587101	Integrin alphaLbeta2 (LFA-1) antagonist	0
NCGC00161398-06	GW-441756	Leucine-Rich Repeat Kinase 2 inhibitor	0
NCGC00486932-01	TM-5275	PAI Inhibitor	0
NCGC00262960-01	Resocortol butyrate	Glucocorticoid receptor Agonist	0
NCGC00507859-01	Larotrectinib	Neurotrophic Tyrosine Kinase Receptors (NTRK) inhibitor	0
NCGC00388375-02	HhSignalingPathwayAntagonist	Sonic hedgehog protein Antagonist	1
NCGC00345791-06	ASP-3026	ALK Tyrosine Kinase Receptor inhibitor	0
NCGC00271415-05	Rifaximin	Anti-bacterial	0
NCGC00347858-02		<MOA Unknown> | Class: Terpenoid | Genus: N/A | Family: N/A | Species: N/A	1
NCGC00018185-23	Tranilast	Interleukin-10 Production Enhancer	0
NCGC00167576-02	Aprindine hydrochloride	Calmodulin Inhibitor	0
NCGC00253909-02	PRT-060318	Tyrosine-protein kinase SYK inhibitor	0
NCGC00379068-03	MK591	Leukotriene Synthesis inhibitor	0
NCGC00015245-10	Bethanechol chloride	Muscarinic receptor Agonist	0
NCGC00015045-09	Methacholine chloride	Muscarinic acetylcholine receptor M2 Binding Agent	0
NCGC00166291-04	Lycopene	response to oxidative stress Modulator	0
NCGC00346438-02	Retaspimycin Hydrochloride	Heat Shock Protein 90 (Hsp90) inhibitor	1
NCGC00166249-03	Erythrosin B		0
NCGC00182548-02	Flutropium bromide	Muscarinic acetylcholine receptor Antagonist	0
NCGC00238621-08	Sotrastaurin	ProteIn kinase C epsilon type inhibitor	0
NCGC00159441-03	Deslanoside	Sodium/potassium-transporting ATPase Inhibitor	1
NCGC00389590-05	THZ1	Cyclin-Dependent Kinase 7 inhibitor	1
NCGC00018113-18	Sodium Phenylbutyrate		0
NCGC00390536-02	Aldoxorubicin hydrochlorid	DNA Topoisomerase II inhibitor	1
NCGC00344556-01	Epitiostanol		0
NCGC00345824-02	BMS-2	HGFR Inhibitor	0
NCGC00389663-01	Empagliflozin (BI 10773)	Sodium/glucose cotransporter 2 Antagonist	0
NCGC00346526-03	PHA-680632	Aurora kinase A inhibitor	0
NCGC00346949-01	ICRF-193	DNA Topoisomerase II Inhibitor	0
NCGC00179431-03	Carbenoxolone sodium	gap junction assembly Inhibitor	0
NCGC00018254-08	Bifonazole	Cytochrome P450 51 Inhibitor	0
NCGC00263905-04	TOLTRAZURIL		0
NCGC00018296-07	Meclizine hydrochloride	Constitutive Androstane Receptor Agonist	0
NCGC00346535-02	IVX-214	Polo-like Kinase-1 (Plk-1) Inhibitor	0
NCGC00015954-04	Sobuzoxane	DNA topoisomerase II Inhibitor	0
NCGC00371093-04	Marimastat	Matrix Metalloproteinase (MMP) inhibitor	0
NCGC00179499-05	Dipivefrin hydrochloride	Beta-2 adrenergic receptor Agonist	0
NCGC00094812-05	Acetarsol		0
NCGC00263467-03	2-Pyrrolidone-5-carboxylic acid		0

NCGC00178719-02	STROPHANTHIDIN		1
NCGC00023722-08	Floxuridine	Thymidylate Synthase inhibitor	0
NCGC00024715-05	L-Arginine		0
NCGC00354729-02	AST-487	Receptor-type tyrosine-protein kinase FLT3 inhibitor	0
NCGC00024623-04	Doxepin hydrochloride	unknown	0
NCGC00510162-01	MSC2530818	Cyclin-Dependent Kinase 8 inhibitor	0
NCGC00179511-08	Racecadotril	Neprilysin inhibitor	0
NCGC00263142-04	Tiplasinin	Plasminogen activator inhibitor 1 inhibitor	0
NCGC00164637-02	Nifekalant	K(ATP) Channel Blocker	0
NCGC00090997-11	Dichlorvos	Butyrylcholinesterase Inhibitor	0
NCGC00651745-01	ABBV-744	Bromodomain-Containing Protein 2 (BD2 domain) inhibitor	1
NCGC00249888-03	Vitamin B12	Coenzyme A Stimulant	0
NCGC00160484-02	Etafenone	vascular smooth muscle contraction Inhibitor	0
NCGC00159469-04	Methoxyphenamine	Adrenergic receptor beta Agonist	0
NCGC00181913-02	Difeterol	Histamine H1 receptor Antagonist	0
NCGC00379052-01	Ticagrelor	Purinergic receptor P2Y12 Antagonist	1
NCGC00095121-16	NATEGLINIDE		0
NCGC00096081-05	beta-Carotene	Antioxidant	0
NCGC00507851-01	OTS-514	Lymphokine activated Killer T-cell originated Protein Kinase inhibitor	1
NCGC00022819-04	Ipratropium bromide	Muscarinic Antagonists	0
NCGC00015219-07	Choline Chloride		0
NCGC00167426-03	Caramiphen edisylate		0
NCGC00015439-20	Flumazenil	Gamma-aminobutyric acid receptor subunit alpha-1 modulator	0
NCGC00094580-10	Allopurinol	Xanthine Oxidase Inhibitor	0
NCGC00378623-01	Dolastatin 10	Tubulin polymerization inhibitor	0
NCGC00346642-04	PHA-665752	Hepatocyte Growth Factor Receptor inhibitor	0
NCGC00092360-02	Cordycepin	Hypoxanthine-guanine phosphoribosyltransferase (HGPRTase) inhibitor	0
NCGC00379045-01	Alfacalcidol-D6	Increase blood calcium level via a variety of mechanisms	0
NCGC00387244-01	NIBR-189	G-protein Coupled Receptor 183 modulator	0
NCGC00014711-14	Oxaprozin	Cyclooxygenase-2 Inhibitor	0
NCGC00183865-01	Flugestone		0
NCGC00179802-02		<MOA Unknown> | Class: Steroid | Genus: Tephrosia | Family: Fabaceae | Species: nana	1
NCGC00017323-08	Tetracycline hydrochloride	Anti-bacterial	0
NCGC00016624-05	Diphenidol	Muscarinic acetylcholine receptor M5 Antagonist	0
NCGC00095748-04	3-(Octadecyloxy)-1,2-propanediol		0
NCGC00168251-01	Ethylmorphine	Opioid receptors; mu/kappa/delta Agonist	0

NCGC00386692-01	UNC-569	Tyrosine-protein kinase Mer inhibitor	0
NCGC00485940-01	FAS-IN-1	Fatty acid synthase Inhibitor	1
NCGC00094558-09	CACODYLIC ACID		0
NCGC00384574-01		<MOA Unknown> | Class: Terpenoid | Genus: N/A | Family: N/A | Species: N/A	1
NCGC00016916-11	Norfloxacin	DNA Topoisomerase IV Inhibitor	0
NCGC00164524-02	D-Glucuronolactone		0
NCGC00159574-16	ZM-447439	Aurora kinase A inhibitor	0
NCGC00167525-02	Tazarotene	Retinoic Acid Receptor gamma agonist	0
NCGC00386226-01	Pralatrexate	Dihydrofolate Reductase (DHFR) Inhibitors	0
NCGC00165719-16	Alendronate (Fosamax)	Farnesyl Pyrophosphate Synthase inhibitor	0
NCGC00163700-12	Vincristine sulfate	Tubulin polymerization inhibitor	1
NCGC00167509-02	Pipethanate ethylbromide	Muscarinic acetylcholine receptor Antagonist	0
NCGC00388518-02	Ticarcillin sodium		0
NCGC00263220-07	Romidepsin	Histone deacetylase 1, class I inhibitor	1
NCGC00159391-03	Sulfobromophthalein	Solute carrier organic anion transporter family member 1B1 Substrate	0
NCGC00024025-05	Dehydrocholic acid	bile acid secretion Inhibitor	0
NCGC00179649-06	Prednisolone	Glucocorticoid Receptor agonist	0
NCGC00486944-01	NVS-PAK1-1	Serine/threonine-protein kinase PAK 1 inhibitor	0
NCGC00018123-07	Acetylcholine chloride	Acetylcholinesterase Substrate	0
NCGC00167586-03	Piroctone Olamine		1
NCGC00538320-01		Prostamide/prostaglandin F synthase Inhibitor	0
NCGC00485994-01	Bivalirudin (Trifluoroacetate)		0
NCGC00023673-18	Adenosine	Adenosine Receptor A1 agonist	0
NCGC00095082-06	Iopanoic acid		0
NCGC00166141-06	Carbinoxamine maleate	Histamine H1 receptor Antagonist	0
NCGC00178520-13	Bromhexine hydrochloride	Mucolytic Agent	0
NCGC00162445-05	Misoprostol	Prostanoid EP3 receptor Agonist	0
NCGC00159326-03	Lincomycin hydrochloride	Antibiotic	0
NCGC00263162-08	RO-4929097	Notch Signaling inhibitor	0
NCGC00344134-02	Limaprost		0
NCGC00179521-03	Cefixime	Bacterial penicillin-binding protein Inhibitor	0
NCGC00346747-02	NCGC00346747	Mitogen-Activated Protein Kinase Kinase Kinase 14 (NIK) inhibitor	0
NCGC00346623-04	BMS-378806	HIV Attachment Inhibitor	0
NCGC00167452-03	Pirenoxine Sodium	maintenance of lens transparency Activator	0
NCGC00531794-01	Cimicoxib		0
NCGC00475735-01	Erdafitinib	Fibroblast Growth Factor Receptor 3 inhibitor	0
NCGC00015389-07	Propofol	Transient receptor potential cation channel subfamily V member 1 agonist	0
NCGC00346096-02	GSK-429286A	Rho-associated protein kinase 1 inhibitor	0

NCGC00263575-01	Ezetimibe	NPC1-like intracellular cholesterol transporter 1 inhibitor	0
NCGC00182981-01	Proxazole citrate		0
NCGC00390459-02	Alsterpaullone		1
NCGC00263855-02	CEFAMANDOLE SODIUM		0
NCGC00167816-03	JX-401	Mitogen-Activated Protein Kinase p38 inhibitor	0
NCGC00024567-03	Ambenonium chloride	Acetylcholinesterase Inhibitor	0
NCGC00263603-10	3-Deazaneplanocin A	Histone-lysine N-methyltransferase Ezh2 inhibitor	1
NCGC00378873-04	Reparixin	Chemokine CXCR1/CXCR2 Receptor (IL-8 Receptor) antagonist	0
NCGC00390625-02	Maropitant	Substance-P receptor Antagonist	0
NCGC00159380-05	Benzoyl peroxide		0
NCGC00182713-18	Canertinib	Epidermal Growth Factor Receptor inhibitor	1
NCGC00387306-01	PT-2385	Hypoxia-inducible factor 2-alpha inhibitor	0
NCGC00379032-06	Tasquinimod	Protein S100-A9 inhibitor	0
NCGC00014017-03	L-Proline		0
NCGC00263164-17	Cabozantinib	Proto-oncogene tyrosine-protein kinase receptor Ret inhibitor	0
NCGC00166290-03	Polyoxyethylene (9) lauryl ether		0
NCGC00166138-04	Ethynodiol diacetate	Progesterone receptor Agonist	0
NCGC00249918-01	Estriol tripropionate		0
NCGC00510479-01	Ensartinib	ALK Tyrosine Kinase Receptor inhibitor	0
NCGC00263201-04	AMG-51	Hepatocyte Growth Factor Receptor inhibitor	0
NCGC00016799-08	Suxibuzone	Prostaglandin G/H synthase 2 Inhibitor	0
NCGC00166097-02	Cinnamyl alcohol	prostaglandin secretion Inhibitor	0
NCGC00094575-01	Tolterodine tartrate	Muscarinic M3 Antagonist	0
NCGC00263149-06	AEE-788	Epidermal Growth Factor Receptor inhibitor	1
NCGC00168759-05	PTC-124	Nonsense Mutation Suppressor	1
NCGC00386421-08	Verdinexor	Exportin-1 antagonist	1
NCGC00346641-07	ADL-5859	Delta-type Opioid Receptor agonist	0
NCGC00014650-04	Ansamitocin P3	Tubulin polymerization inhibitor	0
NCGC00247668-03	LDN-57444	Ubiquitin Carboxyl-Terminal Hydrolase isozyme L1 inhibitor	0
NCGC00188437-01	Sultamicillin tosylate	Staphylococcus Aureus Inhibitor	0
NCGC00079434-04	KL001	Cryptochrome modulator	0
NCGC00181101-01	Isothipendyl hydrochloride	Histamine H1 receptor Antagonist	0
NCGC00016103-16	Lificiguat	Hypoxia-inducible factor 1-alpha inhibitor	0
NCGC00345833-01	SR-3677	Rho-associated protein kinase 2 inhibitor	0
NCGC00238624-07	NCGC00238624	GALK Inhibitor	0
NCGC00025288-07	Deguelin	NADH-Ubiquinone Oxidoreductase (Complex I) inhibitor	1
NCGC00179584-05	Ceftazidime	Penicillin-binding protein 4 Inhibitor	0

NCGC00163125-06	Pregnenolone	Transient receptor potential cation channel subfamily M member 3 agonist	0
NCGC00164557-11	Eprosartan mesylate	urate transport Inhibitor	0
NCGC00346832-02	Terameprocol	Antimitotic Agent	0
NCGC00016288-06	Bemegride	GABA-A receptor; anion channel Antagonist	0
NCGC00164512-02	Guaiacol carbonate		0
NCGC00390788-02	Tirapazamine	DNA Damaging Drug	0
NCGC00159392-05	1,1,1-Trichloro-2-methyl-2-propanol	0	
NCGC00094614-06	4-Chloro-3,5-dimethylphenol		0
NCGC00263219-03	Elacridar	Multidrug resistance protein 1 inhibitor	0
NCGC00347708-02		<MOA Unknown> | Class: N/A | Genus: N/A | Family: N/A | Species: N/A	1
NCGC00181783-05	Iguratimod	NF-kappaB (NFKB) Activation inhibitor	0
NCGC00164380-01	Formestane	Aromatase inhibitor	0
NCGC00091106-05	Methyl salicylate		0
NCGC00185850-16	BIX-01294	DNA Methyltransferase (DNMT) inhibitor	0
NCGC00161408-07	Anagrelide	Phosphodiesterase 3A inhibitor	0
NCGC00159365-04	m-Cresyl acetate		0
NCGC00183121-01	Zinc pyrithione		1
NCGC00242514-13	Sepantronium bromide	Survivin inhibitor	0
NCGC00253607-01	Watanidipine	calcium ion transmembrane transport Blocker	0
NCGC00178319-06	Thiamphenicol	Antibiotic	0
NCGC00185778-06	Nitisinone	4-Hydroxyphenylpyruvate Dioxygenase inhibitor	0
NCGC00016009-19	Tolazamide	ATP-sensitive inward rectifier potassium channel 1 Inhibitor	0
NCGC00160662-02	Ambrisentan	Endothelin ETA Receptor Antagonist	0
NCGC00189144-02	UNC-0646	Histone-lysine N-methyltransferase EHMT2 inhibitor	0
NCGC00241099-04	Saracatinib	Proto-oncogene tyrosine-protein kinase Src inhibitor	1
NCGC00351603-15	Ceritinib	ALK Tyrosine Kinase Receptor inhibitor	0
NCGC00480817-01	FG-2216	Hypoxia-Inducible Factor Prolyl Hydroxylase inhibitor	0
NCGC00507845-01	Netarsudil	Rho-associated protein kinase 1 inhibitor	1
NCGC00263167-10	SNS-032	Cyclin-Dependent Kinase 9 inhibitor	0
NCGC00018249-05	Ethoxzolamide	Carbonic anhydrase Inhibitor	0
NCGC00016265-08	Folic acid	folic acid-containing compound metabolic process Substrate	0
NCGC00017063-13	Amodiaquine		0
NCGC00351602-09	AP-26113	Insulin-like growth factor 1 receptor inhibitor	0
NCGC00532504-01	CARNIDAZOLE	Trichomonas Inhibitor	0
NCGC00095156-06	Carbadox		0
NCGC00344546-01	Fenipentol		0

NCGC00386327-03	MPI-0479605	Dual Specificity protein kinase TTK inhibitor	0
NCGC00263091-15	AT-7519	Cyclin-Dependent Kinase 9 inhibitor	0
NCGC00381605-01	MKC-1	Protein Kinase C alpha type inhibitor	1
NCGC00166153-02	Climbazole		0
NCGC00091066-01	3-Methylbutyl nitrite	vasodilation Activator	0
NCGC00508855-01	Danirixin	Chemokine CXCR2 Receptor (IL-8 beta Receptor) antagonist	0
NCGC00161410-03	CCT-018159	Heat Shock Protein 90 (Hsp90) inhibitor	0
NCGC00016395-01	Diphenylpyraline hydrochloride	Histamine H1 receptor Agonist	0
NCGC00090698-06	1,3-Diphenylguanidine		0
NCGC00522552-01	PI4KIIIbeta-IN-10		1
NCGC00182708-01	Pipecuronium bromide	Muscle-type nicotinic acetylcholine receptor Antagonist	0
NCGC00159450-04	Parethoxycaine		0
NCGC00016831-09	Buflomedil hydrochloride	Vasodilator	0
NCGC00510440-01	CPI-4203	Lysine-specific demethylase 5A inhibitor	0
NCGC00181006-02	1-(4-Hydroxyphenyl)-2-methyl-2-methylaminoethanol hydrochloride	0	
NCGC00167522-02	Hexoprenaline sulfate	Beta-2 adrenergic receptor Agonist	0
NCGC00347910-01	CK-59	Calcium/calmodulin-dependent protein kinase kinase 2 inhibitor	0
NCGC00510705-01	ASP-9521	Aldo-Keto Reductase family 1 member C3 inhibitor	0
NCGC00344326-02	9(E),12(E)-Octadecadienoic acid	Streptococcus mutans Inhibitor	0
NCGC00185773-02	Clindamycin palmitate	Antibacterial	0
NCGC00346501-07	Bleomycin sulfate	DNA strand break inducer	0
NCGC00167438-02	Trospium chloride	Muscarinic Antagonists	0
NCGC00185997-11	AS-252424	Phosphatidylinositol 3-Kinase gamma isoform inhibitor	0
NCGC00167565-02	Carbazochrome sodium sulfonate	Secretory Phospholipase A2 (sPLA2) inhibitor	0
NCGC00179332-03	Naftifine hydrochloride	Squalene monooxygenase Inhibitor	0
NCGC00015482-15	Gallamine triethiodide	Muscarinic acetylcholine receptor M2 antagonist	0
NCGC00024622-05	Dantrolene sodium	Ryanodine receptor 1 Antagonist	0
NCGC00346631-02	Orteronel	Steroid 17-alpha-hydroxylase/17,20 lyase inhibitor	0
NCGC00386673-01	AS 1517499	Signal transducer and activator of transcription 6 Inhibitor	1
NCGC00024584-17	BROMOCRIPTINE		0
NCGC00370832-01	JNJ-28871063 hydrochloride	Receptor tyrosine-protein kinase HER4 (erbB4) inhibitor	0
NCGC00185990-14	Ketorolac	non-steroidal antiinflammatory	0
NCGC00162247-06	Levallorphan	Mu-type opioid receptor Antagonist	0
NCGC00480787-02	Cangrelor (AR-C69931)		0
NCGC00346711-02	Galeterone	Steroid 17-alpha-hydroxylase/17,20 lyase inhibitor	0
NCGC00346605-03	Sodium Danshensu	Pregnane X Receptor (PXR) Regulator	0

NCGC00319020-01		Prostanoid IP receptor Agonist	0
NCGC00024976-06	CGS-21680	Adenosine Receptor A2A agonist	0
NCGC00346487-03	Quisinostat	Histone deacetylase 1, class I inhibitor	1
NCGC00347951-03	LSD1-C76	Lysine-Specific Histone Demethylase 1A inhibitor	0
NCGC00347903-02	Ro-51	P2X Purinoceptor 3 antagonist	0
NCGC00091297-05	Anilinium nitrate		0
NCGC00015099-12	Alprenolol hydrochloride	Beta-adrenergic receptor Antagonist	0
NCGC00386239-01	Mirabegron	Beta-3 Adrenergic Receptor agonist	0
NCGC00094818-09	Hexestrol	Aldo-Keto Reductase family 1 member C1 inhibitor	0
NCGC00016385-08	Sulfabenzamide		0
NCGC00346827-02	7-Hydroxystaurosporine	Checkpoint kinase 1 inhibitor	1
NCGC00164626-02	Tiagabine hydrochloride	Sodium and chloride-dependent GABA transporter 1 inhibitor	0
NCGC00179626-05		Mineralocorticoid Receptor Antagonist	0
NCGC00390734-01	Nedaplatin	DNA Crosslinking Agent	0
NCGC00346435-07	Niraparib	Poly [ADP-ribose] polymerase 1 inhibitor	0
NCGC00024842-05	Clomethiazole		0
NCGC00346739-06	Retigabine	Potassium Voltage-Gated Channel Subfamily KQT Member 2 activator	0
NCGC00015339-07	Dequalinium dichloride	Protein kinase C beta Inhibitor	0
NCGC00023509-18	Lovastatin	HMG-CoA Reductase inhibitor	0
NCGC00263015-01	NCGC00263015	Toll-Like Receptor 4 antagonist	0
NCGC00179602-03	Testosterone propionate	Androgen Receptor Agonist	0
NCGC00263161-11	PF-04217903	Hepatocyte Growth Factor Receptor inhibitor	0
NCGC00346885-02	Sipatrigine	Sodium channel protein type 2 subunit alpha Blocker	0
NCGC00242486-04	AZD-1480	Tyrosine-protein kinase JAK2 inhibitor	1
NCGC00250408-10	GSK-2126458	Phosphatidylinositol 3-Kinase beta isoform inhibitor	1
NCGC00345831-03	SB-242235	Mitogen-Activated Protein Kinase p38 inhibitor	0
NCGC00015572-06	MIBG		0
NCGC00181009-02	Timepidium bromide	Muscarinic acetylcholine receptor M5 Antagonist	0
NCGC00263176-03	BMS-794833	Vascular Endothelial Growth Factor Receptor 2 (VEGFR-2) inhibitor	0
NCGC00532324-01	VX-984	DNA-Dependent Protein Kinase inhibitor	0
NCGC00018172-18	Indapamide	Carbonic Anhydrase 7 inhibitor	0
NCGC00274280-01	Pixantronemaleate	DNA Modulator	0
NCGC00159451-06	Tinoridine	lipid oxidation Inhibitor	0
NCGC00093985-19	Metolazone	Carbonic Anhydrase 7 inhibitor	0
NCGC00389228-01	SAR-131675	VEGFR-3 Inhibitor	0
NCGC00179582-03	Bacampicillin hydrochloride		0
NCGC00390645-03	CPI-360	Histone-lysine N-methyltransferase EZH2 inhibitor	0

NCGC00017331-08	(-)-Epicatechin	superoxide anion generation Inhibitor	0
NCGC00484057-01	Methenolone (acetate)		0
NCGC00179612-03	Neomycin sulfate	30S Ribosomal Protein Inhibitors	0
NCGC00242492-04	BIBR-1532	Telomerase reverse transcriptase inhibitor	0
NCGC00163432-05	Calpeptin	Calpain inhibitor	0
NCGC00162463-03	Ionomycin(Calciumsalt)	Potassium channel subfamily K member 2 Inhibitor	1
NCGC00165902-08	SU-MI-2	Hepatocyte Growth Factor Receptor inhibitor	0
NCGC00346571-07	SB-743921	Kinesin-Like Protein KIF11 inhibitor	0
NCGC00480786-05	Brequinar sodium	Dihydroorotate Dehydrogenase inhibitor	0
NCGC00178874-03	DL-Carnitine HCl	Mitochondrial carnitine/acylcarnitine carrier protein Substrate	0
NCGC00016035-13	Trazodone hydrochloride	alpha2-Adrenoceptor Antagonists	0
NCGC00273985-04	Wyeth Cot Inhibitor 2	Tumor necrosis factor alpha Production inhibitor	0
NCGC00344522-01	Gluconolactone	Calcium Chelating Agent	0
NCGC00165875-04	PB-28	Sigma non-opioid intracellular receptor 1 antagonist	0
NCGC00389674-01	Apremilast (CC-10004)	Phosphodiesterase 4 Inhibitor	0
NCGC00016549-05	Dosulepin hydrochloride	Serotonin transporter Inhibitor	0
NCGC00014483-16	Pamelor	Norepinephrine transporter Inhibitor	0
NCGC00016872-12	Ticlopidine hydrochloride	P2Y Purinoceptor 12 antagonist	0
NCGC00253587-01	Sitaxentan	Endothelin-1 Receptor antagonist	0
NCGC00263152-15	Ponatinib	Proto-oncogene tyrosine-protein kinase receptor Ret inhibitor	1
NCGC00182851-02	Meprednisone	Glucocorticoid Receptor agonist	0
NCGC00510316-02	Ombitasvir		0
NCGC00093889-12	Physostigmine		0
NCGC00346656-03	Dinaciclib (SCH727965)	Cyclin-Dependent Kinase 12 inhibitor	1
NCGC00015080-08	Methylatropine nitrate	Muscarinic acetylcholine receptor Antagonist	0
NCGC00249685-09	Dovitinib	Vascular Endothelial Growth Factor Receptor 2 (VEGFR-2) inhibitor	1
NCGC00181037-03	Isoflurane	Glycine receptor Positive Allosteric Modulator	0
NCGC00263139-03	Rolofylline	Adenosine Receptor A1 antagonist	0
NCGC00508820-02	Dalfopristin	Bacterial 70S ribosome Inhibitor	0
NCGC00016087-11	Vigabatrin	Gamma-amino-N-butyrate transaminase Inhibitor	0
NCGC00371029-01	MJ 15	Cannabinoid receptor Antagonist	1
NCGC00167470-05	Mezlocillin Sodium	Bacterial penicillin-binding protein Inhibitor	0
NCGC00482751-01	Casticin		1
NCGC00016612-05	Sulfadoxine	Dihydropteroate Synthase (DHPS) Inhibitor	0
NCGC00094810-08	Warfarin sodium	Vitamin K Epoxide Reductase inhibitor	0
NCGC00185765-02	Encainide hydrochloride	Sodium channel protein type V alpha subunit Inhibitor	0

NCGC00507893-01	UK-371804	Urokinase-type plasminogen activator inhibitor	0
NCGC00250375-10	Linsitinib	Insulin-like growth factor 1 receptor inhibitor	0
NCGC00390402-02	NSC-23925	Multidrug resistance protein 1 inhibitor	0
NCGC00380658-01		<MOA Unknown> | Class: Steroide | Genus: N/A | Family: N/A | Species: N/A	1
NCGC00015480-05	GW-7647	PPARalpha Agonist	0
NCGC00346612-06	LY-2140023	Glutamate receptor metabotropic 2 agonist	0
NCGC00181159-03	Trabectedin	DNA Damaging Drug	0
NCGC00346475-02	Birinapant	IAP inhibitor	0
NCGC00346556-03	Semagacestat	gamma-Secretase inhibitor	0
NCGC00385189-01		<MOA Unknown> | Class: Steroid | Genus: Nerium | Family: Apocynaceae | Species: oleander	1
NCGC00481082-01	BMS-595	Casein Kinase 2 alpha prime inhibitor	1
NCGC00159503-02	Methylprednisolone acetate		0
NCGC00015436-11	Fluphenazine hydrochloride	Dopamine Receptor D2 antagonist	1
NCGC00250406-02	Tipifarnib	Protein farnesyltransferase/geranylgeranyltransferase type-1 inhibitor	1
NCGC00095304-05	Furazolidone	DNA Crosslinking Agent	0
NCGC00263177-04	CUDC-101	Epidermal Growth Factor Receptor inhibitor	1
NCGC00093704-43	Cyclosporin A	Serine/threonine-protein phosphatase 2B inhibitor	0
NCGC00183005-01	Zanapezil fumerate	Acetylcholinesterase Inhibitor	0
NCGC00185981-05			1
NCGC00387711-02	AZD-6738	Serine-protein kinase ATR inhibitor	0
NCGC00386380-02	Tideglusib	Glycogen Synthase Kinase 3 (GSK-3) inhibitor	0
NCGC00247029-02	2-Deoxyglucose	Glucose-6-phosphate isomerase inhibitor	0
NCGC00015725-27	Nimesulide	Cyclooxygenase 2(COX2) inhibitor	0
NCGC00510518-01	CFI-402257	Dual Specificity protein kinase TTK inhibitor	0
NCGC00167578-04	Mitiglinide calcium	Insulin Secretagogue	0
NCGC00094721-07	Methimazole	Thyroid peroxidase Inhibitor	0
NCGC00402372-02	AZ-82	Kinesin-Like Protein KIFC1 inhibitor	0
NCGC00025219-10	Purvalanol A	Cyclin-Dependent Kinase 4 inhibitor	1
NCGC00186014-02	Paliperidone	Serotonin 2a (5-HT2a) receptor Antagonist	0
NCGC00345882-03	Silodosin	alpha1-Adrenoceptor Antagonists	0
NCGC00380750-01		<MOA Unknown> | Class: Polyketide | Genus: N/A | Family: N/A | Species: N/A	1
NCGC00384192-02	TH588	7,8-Dihydro-8-Oxoguanine Triphosphatase inhibitor	1
NCGC00344521-01	1-(cis-3-Chloroallyl)-3,5,7-triaza-1-azoniaadamantane chloride	0	
NCGC00183844-04	Sitafloxacin hydrate	Anti-bacterial	0
NCGC00091635-09	Fenthion	Acetylcholinesterase Inhibitor	0

NCGC00095109-08	Venlafaxine	5-HT Reuptake Inhibitor	0
NCGC00356417-05	LLY-507	Sphingomyelin phosphodiesterase 2 inhibitor	0
NCGC00247866-05	NCGC00247866	Bloom Helicase inhibitor	0
NCGC00165883-04	Rufinamide	Sodium channel protein type IX alpha subunit Modulator	0
NCGC00346456-07	Macitentan	Endothelin-1 Receptor antagonist	0
NCGC00094854-07	Allantoin	Allantoinase Substrate	0
NCGC00023234-05	Flurandrenolide		0
NCGC00179665-04	Danazol	Androgen Receptor Agonist	0
NCGC00091056-05	Styrene		0
NCGC00250383-02	Irestatin 9389	Serine/threonine-protein kinase/endoribonuclease IRE1 inhibitor	1
NCGC00386567-02	FLIBANSERIN	5-HT1A Receptor agonist	0
NCGC00347956-02	SR-9011	Nuclear Receptor Subfamily 1 Group D Member 2 agonist	1
NCGC00181019-01	Testosterone isocaproate		0
NCGC00015950-05	SB-415286	Glycogen Synthase Kinase 3 (GSK-3) inhibitor	0
NCGC00095169-07	Miltefosine		1
NCGC00263115-03	Ibutamoren mesilate	Growth Hormone Secretagogue	0
NCGC00510478-01	ARV-771	Bromodomain-Containing Protein 4 (Brd4) inhibitor	1
NCGC00344623-17	UNC-1215	Lethal(3)malignant Brain Tumor-like Protein 3 inhibitor	0
NCGC00386382-03	Lurasidone (Hydrochloride)		0
NCGC00356145-10	CB-839	Glutaminase inhibitor	0
NCGC00165976-02	Squalane		0
NCGC00415059-02	AZD-3965	Monocarboxylate Transporter 2 inhibitor	0
NCGC00166111-06	Estriol	Estrogen Receptor agonist	0
NCGC00090987-05	1-Naphthaleneacetic acid	Fusarium oxysporum Inhibitor	0
NCGC00386314-03	GSK-2656157	Protein kinase R (PKR)-like endoplasmic reticulum kinase (PERK)	0
NCGC00180843-03		<MOA Unknown> | Class: Iridoid | Genus: Kickxia | Family: Apocynaceae | Species: lanigera	1
NCGC00262398-01	NCGC00262398	Ubiquitin Carboxyl-Terminal Hydrolase 2 inhibitor	1
NCGC00022037-07	Medroxyprogesterone acetate	Progesterone Receptor agonist	0
NCGC00165761-07	BMS-345541	inhibitor of nuclear factor kappa B kinase subunit beta inhibitor	1
NCGC00263230-01	FASN MRK	Fatty Acid Synthase inhibitor	0
NCGC00015716-09	Nylidrin	Glutamate NMDA receptor; Grin1/Grin2c Antagonist	0
NCGC00094817-05	Flopropione	Catechol O-methyltransferase Inhibitor	0
NCGC00346891-02	Pyroxamide	Histone deacetylase 1, class I inhibitor	0
NCGC00178238-05	Midodrine hydrochloride	Alpha-1b adrenergic receptor Agonist	0
NCGC00507848-01	GSK-583	Receptor-interacting serine/threonine-protein kinase 2 inhibitor	0

NCGC00018167-05	Cladribine	Adenosine Deaminase Inhibitor	0
NCGC00522560-01	Tasimelteon	Melatonin receptor type 1B Agonist	0
NCGC00253603-01	Mibolerone	Progesterone receptor Agonist	0
NCGC00510442-01	Abametapir	Matrix Metalloproteinase-9 inhibitor	0
NCGC00183024-01	Proglumetacin	Cyclooxygenase-2 Inhibitor	0
NCGC00346838-01	Secoisolariciresinol	Phenylpropanoid	0
NCGC00016338-10	Dibucaine	Sodium channel protein type X alpha subunit Blocker	0
NCGC00509987-01	Avacopan	C5a Anaphylatoxin Chemotactic Receptor 1 antagonist	0
NCGC00242480-03	Ivacaftor	CFTR Channel Potentiator	0
NCGC00386367-02	GNE-9605	Leucine-Rich Repeat Kinase 2 inhibitor	0
NCGC00160682-02	Isocaine		0
NCGC00015713-13	Nitrendipine	Intermediate conductance calcium-activated potassium channel protein 4 Inhibitor	0
NCGC00344565-01	Mizolastine	Histamine H1 Receptor Antagonists	0
NCGC00016268-21	Niacin	Hydroxycarboxylic Acid Receptor 3 agonist	0
NCGC00182053-03	Acrivastine	Histamine H1 receptor Antagonist	0
NCGC00167444-04	Cefpiramide (sodium)	Bacterial penicillin-binding protein Inhibitor	0
NCGC00378691-05	Asunaprevir (Prop INN; USAN) BMS-650032 Sunvepra	0	
NCGC00022304-21	ACETYLCYSTEINE	NF-kappaB (NFKB) Activation inhibitor	0
NCGC00164615-05	Prulifloxacin	Antibacterial	0
NCGC00095978-06	Lufenuron		0
NCGC00484080-02	LX-7101	LIM domain kinase 2 inhibitor	0
NCGC00182979-02	Perlapine	Histamine H1 receptor Inverse Agonist	0
NCGC00015817-14	Promethazine		0
NCGC00166146-02	Quinestrol	Estrogen receptor Agonist	0
NCGC00344182-03	5-trans Prostaglandin F2?	Prostanoid FP receptor Agonist	0
NCGC00348371-01	Chloramphenicol palmitate	Antibiotic	0
NCGC00188943-09	Orotic acid	Dihydroorotate dehydrogenase metabolite	0
NCGC00183853-01	Mosapramine	Dopamine D4 receptor Antagonist	0
NCGC00164612-02	Propyphenazone	Cyclooxygenase-2 Inhibitor	0
NCGC00025109-13	ZM-336372	RAF Kinase inhibitor	0
NCGC00345885-04	Etravirine	P-glycoprotein 1 Inhibitor	0
NCGC00402358-02	Epacadostat	Indoleamine 2,3-dioxygenase 1 inhibitor	0
NCGC00345845-02	TG-89	Tyrosine-protein kinase JAK2 inhibitor	1
NCGC00387800-02	AHU-377	Neprilysin inhibitor	0
NCGC00015605-09	Lamotrigine	Sodium channel protein type 1 subunit alpha Blocker	0
NCGC00387780-01	Cdk1/2 inhibitor III	Cyclin-dependent kinase 2/cyclin A Inhibitor	1
NCGC00346457-03	Copanlisib	Phosphatidylinositol 3-Kinase alpha isoform inhibitor	0
NCGC00163778-09	Imazalil sulfate	O75469|||Q9UJ26 REC20 - 20% relative effective concentration; the concentration of	0

		the test compound showing 20% of the agonistic activity of 1 _ 10_5 M rifampicin via hPXR	
NCGC00015675-22	Milrinone	Phosphodiesterase III (PDE3) Inhibitor	0
NCGC00344510-06	NPS-2143	Calcium-Sensing Receptor antagonist	0
NCGC00274030-16	Infigratinib	Fibroblast Growth Factor Receptor 1 inhibitor	0
NCGC00389300-01	Nelfinavir	HIV Protease Inhibitor	1
NCGC00347066-11	KUC105555N	Valosin-containing protein (VCP) p97 / Transitional endoplasmic reticulum ATPase inhibitor	0
NCGC00016488-15	Kinetin	Acidic mammalian chitinase inhibitor	0
NCGC00162249-09	MK-886	Arachidonate 5-Lipoxygenase-Activating Protein inhibitor	0
NCGC00161290-05	16,16-dimethyl PGE2	prostaglandin	0
NCGC00250385-11	XMD8-92	Leucine-Rich Repeat Kinase 2 inhibitor	1
NCGC00161605-02	L-Ascorbyl 6-palmitate		0
NCGC00163474-07	Cyclopamine	Smoothened Receptor antagonist	0
NCGC00381227-01		<MOA Unknown> | Class: Triterpen | Genus: trichilia | Family: Meliaceae | Species: americana	1
NCGC00346492-03	XL-147	Phosphatidylinositol 3-Kinase alpha isoform inhibitor	0
NCGC00179264-03	Tiletamine Hydrochloride	Glutamate [NMDA] receptor subunit epsilon 2 Antagonist	0
NCGC00253595-01	Clocapramine	Serotonin 2a (5-HT2a) receptor Antagonist	0
NCGC00389745-01	RG7112	p53-binding protein Mdm-2 Binding Agent	1
NCGC00016423-15	(-)-Gossypol	Apoptosis regulator Bcl-2 inhibitor	1
NCGC00091367-05	Danthron	DNA topoisomerase II alpha Inhibitor	0
NCGC00531790-01	JTT-501	Peroxisome proliferator-activated receptor gamma Agonist	0
NCGC00016391-13	Clioquinol		1
NCGC00090731-08	2-Phenoxyethanol	Vanilloid receptor Agonist	0
NCGC00166270-02	1,3,5-Trihydroxybenzene dihydrate	Voltage-gated calcium channel Inhibitor	0
NCGC00023339-16	Pilocarpine	Muscarinic acetylcholine receptor M3 Agonist	0
NCGC00476204-01	PFI-4	Bromodomain and PHD finger containing 1 inhibitor	0
NCGC00168883-03		<MOA Unknown> | Class: Terpenoid | Genus: Lasiolena | Family: N/A | Species: santosii	1
NCGC00505019-01	LMP-744	DNA Topoisomerase I inhibitor	1
NCGC00090820-03	4-Acetamidobenzoic acid	immune response Modulator	0
NCGC00509935-01	AG-881	Isocitrate Dehydrogenase [NADP] cytoplasmic (Arg132His Mutant) inhibitor	0
NCGC00183808-18	ML324	Lysine-Specific Demethylase 4E inhibitor	0
NCGC00164563-04	Entecavir (monohydrate)	DNA Polymerase Inhibitors	0
NCGC00346509-08	GSK-615	Phosphatidylinositol 3-Kinase alpha isoform inhibitor	1
NCGC00016094-10	17_-hydroxy Wortmannin	PI3K Inhibitor	0

NCGC00263146-04	SR-3306	Mitogen-Activated protein kinase 8 (JNK) inhibitor	1
NCGC00160431-02	2-[(4-Chloro-3-methylphenyl)amino]benzoic acid	0	
NCGC00181750-01	Cefozopran hydrochloride	Bacterial penicillin-binding protein Binding Agent	0
NCGC00510483-02	Amcasertib	Platelet-derived growth factor receptor alpha inhibitor	0
NCGC00344524-01	Benzylhexadecyldimethylammonium chloride	0	
NCGC00356071-11	CPI-203	Bromodomain-Containing Protein 4 (Brd4) Degradation inducer	1
NCGC00185741-02	Nepafenac	Cyclooxygenase-2 Inhibitor	0
NCGC00166248-03	Green S		0
NCGC00346471-03	Rebeccamycin	DNA Topoisomerase I inhibitor	1
NCGC00345815-02	MG-115	Proteasome inhibitor	1
NCGC00090965-07	Propyl-4-hydroxybenzoate		0
NCGC00018218-13	Edaravone	5'-Nucleotidase inhibitor	0
NCGC00346895-01	Amastatin	Cytosolic leucine aminopeptidase inhibitor	0
NCGC00249392-05	Telatinib	Vascular Endothelial Growth Factor Receptor 3 (VEGFR-3) inhibitor	0
NCGC00386381-04	Besifloxacin Hydrochloride	Topoisomerase IV Inhibitor	0
NCGC00021305-08	Sirolimus	Rapamyin analog mTORC inhibitor	0
NCGC00485958-01	Eluxadoline		0
NCGC00016354-11	Butylparaben		0
NCGC00263145-02	BI-78D3	Mitogen-Activated protein kinase 8 (JNK) inhibitor	0
NCGC00160606-01	Sulbentine		0
NCGC00015790-18	PD-098059	Dual Specificity Mitogen-Activated Protein Kinase Kinase 1 inhibitor	0
NCGC00185774-06	Erdosteine	Mucolytic Agent	0
NCGC00242597-10	Saxagliptin	Dipeptidyl Peptidase IV inhibitor	0
NCGC00163409-07	Betulinic acid	Caspase 3/8 Activator	0
NCGC00351594-01	BCL6 Inhibitor	B-Cell Lymphoma 6 Protein inhibitor	0
NCGC00016527-06	Clopamine		0
NCGC00408930-02	NCGC00408930	Nuclear Factor Erythroid 2-Related Factor 2 inhibitor	1
NCGC00345802-01	ACY-1215	Histone deacetylase 6, class IIB inhibitor	0
NCGC00378921-05	Merestinib	Hepatocyte Growth Factor Receptor inhibitor	0
NCGC00015760-12	Octopamine	Dopamine transporter Binding Agent	0
NCGC00015962-22	SB-202190	Mitogen-Activated protein kinase 8 (JNK) inhibitor	0
NCGC00386713-03	WAY-262611	Dickkopf WNT Signaling Pathway inhibitor 1 inhibitor	0
NCGC00386361-02	NMS-P937	Serine/threonine-protein kinase PLK1 inhibitor	0
NCGC00386518-01	Cannabidiol	Cannabinoid Receptor 1 agonist	0
NCGC00378943-01	DB-07268	Mitogen-Activated protein kinase 8 (JNK) inhibitor	0

NCGC00159377-04	4-tert-Butylphenol	UDP-glucuronosyltransferase 1-6 Substrate	0
NCGC00384172-01	WDR5-C47	Inhibitor of WDR5-MLL interaction. Not the mest WDR5-MLL Inhibitor available but this one offers a different chemotype.	0
NCGC00159486-06	Hippuric acid	Solute carrier family 22 member 8 Inhibitor	0
NCGC00165752-04	Eslicarbazepine (acetate)	Sodium channel alpha subunit Antagonist	0
NCGC00385002-01		<MOA Unknown> | Class: N/A | Genus: N/A | Family: N/A | Species: N/A	1
NCGC00387273-01	TA02	Mitogen-Activated Protein Kinase p38 inhibitor	0
NCGC00015523-13	Hydroquinone		0
NCGC00160518-03	Orbifloxacin	DNA gyrase Binding Agent	0
NCGC00091596-05	Octadecanoic acid		0
NCGC00387792-03	PX-866	Phosphatidylinositol 3-Kinase alpha isoform inhibitor	0
NCGC00390559-05	Azilsartan Medoxomil	Type-1 angiotensin II receptor Antagonist	0
NCGC00163470-22	FK-506	Serine/threonine-protein phosphatase 2B inhibitor	0
NCGC00015917-09	SB-206553	5-HT2B Receptor Antagonist	0
NCGC00263209-09	ML-141	Cell Division Control Protein 42 Homolog inhibitor	0
NCGC00248146-03	4-Methylbenzenesulfonic acid		0
NCGC00180839-02	Penta-O-galloyl-????-D-glucose hydrate	1	
NCGC00263174-02	Ispinesib	Kinesin-Like Protein KIF11 inhibitor	0
NCGC00507838-01	Naquotinib mesylate	Epidermal Growth Factor Receptor (Thr790Met Mutant) inhibitor	1
NCGC00179767-02		lipid metabolic process Modulator	0
NCGC00160603-02	4-Styrylpyridine		0
NCGC00183014-01	Sulfamethomidine	Bacterial dihydrofolate reductase Inhibitor	0
NCGC00249661-03	NCGC00249661	15-hydroxyprostaglandin dehydrogenase [NAD(+)] inhibitor	0
NCGC00389554-01	DORAMECTIN	chloride transmembrane transport Activator	1
NCGC00181909-01	Sulcaine		0
NCGC00346635-10	WYE-125132	mTOR Complex 1 (mTORC1) inhibitor	1
NCGC00485875-01	Fluorocyclopentenylcytosine	Cyclin-Dependent Kinase 1 inhibitor	0
NCGC00346431-02	Omigapil	Glyceraldehyde 3-phosphate Dehydrogenase inhibitor	0
NCGC00346836-01	GSK-2334470	3-Phosphoinositide Dependent Kinase 1 inhibitor	0
NCGC00182063-03	Cadralazine		0
NCGC00389231-01	Riviciclib (P276-00)	Cyclin-Dependent Kinase 4 inhibitor	1
NCGC00022680-05	Chlormadinone acetate	Androgen receptor Antagonist	0
NCGC00482383-01	EPZ-020411	Protein arginine N-methyltransferase 6 inhibitor	0
NCGC00186632-01	Strychnine	Glycine receptor subunit alpha-1 Antagonist	0
NCGC00164548-02	Danofloxacin	Bacterial DNA gyrase Inhibitor	0
NCGC00346608-02	Astragaloside A	Telomerase reverse transcriptase activator	0
NCGC00024494-02	D-Alanine	Alanine aminotransferase 1 Substrate	0

NCGC00510689-01	YU-238259	Homology-dependent DNA repair inhibitor	0
NCGC00188344-07	Navitoclax (ABT-263)	Apoptosis regulator Bcl-2 inhibitor	1
NCGC00274083-01	Loxiglumide	Cholecystokinin A receptor Antagonist	0
NCGC00379161-04	ML-228	Hypoxia-inducbile factor 1-alpha activator	1
NCGC00346837-02	JZL-195	Fatty Acid Amide Hydrolase inhibitor	0
NCGC00346555-05	Apixaban	Coagulation Factor Xa inhibitor	0
NCGC00189220-12	UNC-0638	Histone-lysine N-methyltransferase EHMT2 inhibitor	0
NCGC00092375-02	JNJ-16259685	Glutamate receptor metabotropic 1 antagonist	0
NCGC00182084-01	Clorsulon	antiparasitic agent	0
NCGC00015360-08	Pentetic acid	Radioactive metals Chelating Agent	0
NCGC00181103-02	Propiverine hydrochloride	Muscarinic acetylcholine receptor Blocker	0
NCGC00018130-08	PHENAZOPYRIDINE HYDROCHLORIDE	0	
NCGC00160395-04	Dibenzepin hydrochloride	Monoamine transporter Inhibitor	0
NCGC00168109-05	Secin H3	Cytohesin inhibitor	0
NCGC00179625-03	Thioproperazine dimethanesulfonate	Serotonin 2a (5-HT2a) receptor Antagonist	0
NCGC00016386-06	Sulfamerazine	Bacterial dihydropteroate synthase Inhibitor	0
NCGC00181008-02	Olsalazine sodium	Cyclooxygenase Inhibitor	0
NCGC00183004-01	Alilusem potassium	renal sodium ion transport Inhibitor	0
NCGC00390417-03	GSK-872	Receptor-interacting serine/threonine-protein kinase 3 inhibitor	0
NCGC00389632-06	Tipiracil	Thymidine Phosphorylase inhibitor	0
NCGC00262061-03	Ro-31-8425	Protein Kinase C inhibitor	0
NCGC00386224-06	Icotinib hydrochloride	Epidermal Growth Factor Receptor inhibitor	0
NCGC00167529-02	Itopride hydrochloride	Acetylcholinesterase Inhibitor	0
NCGC00346534-03	MC-1568	Histone deacetylase 2, class I inhibitor	0
NCGC00177980-03	Oxiconazole nitrate	Steroid biosynthesis Inhibitor	0
NCGC00346607-02	Curcumol	Glutathione-S-transferase P inhibitor	0
NCGC00522029-01	Zuclopenthixol Decanoate		0
NCGC00016244-15	Isoniazid	Anti-bacterial	0
NCGC00263188-03	YO-01027	gamma-Secretase inhibitor	0
NCGC00496843-02	Ripasudil	Rho-associated protein kinase 1 inhibitor	0
NCGC00344549-05	Oxandrolone	Androgen Receptor agonist	0
NCGC00015708-15	Maprotiline hydrochloride	Sodium-dependent Noradrenaline Transporter inhibitor	0
NCGC00263151-07	JNJ-7706621	Aurora kinase A inhibitor	1
NCGC00475743-01	Motolimod	Toll-Like Receptor 8 agonist	0
NCGC00024778-02	Bifemelane	Monoamine oxidase B Inhibitor	0
NCGC00241112-04	RWJ-67657	Tumor necrosis factor alpha Production inhibitor	0
NCGC00347846-02		<MOA Unknown> | Class: polyketide/AS | Genus: N/A | Family: N/A | Species: N/A	1
NCGC00346648-05	GSK-1070916A	Aurora kinase B inhibitor	0

NCGC00186035-08	IRAK-1-4 Inhibitor I	Interleukin-1 Receptor-Associated Kinase 1 inhibitor	0
NCGC00386712-02	TMP195	Histone deacetylase 9, class IIA inhibitor	0
NCGC00179248-03	Articaine hydrochloride	Sodium channel alpha subunit Blocker	0
NCGC00346500-08	PIK-75	Phosphatidylinositol 3-Kinase alpha isoform inhibitor	0
NCGC00162413-14	Geldanamycin	Heat Shock Protein 90 (Hsp90) inhibitor	0
NCGC00025251-08	GW-1929	PAPRgamma Agonist	0
NCGC00488781-01	Empesertib	Dual Specificity protein kinase TTK inhibitor	0
NCGC00348215-03	Bedaquiline (TMC-207)	ATP synthase subunit c Inhibitor	0
NCGC00510507-01	Evobrutinib	Bruton's Tyrosine Kinase (BTK) inhibitor	0
NCGC00247877-09	CNF-2024	Heat Shock Protein 90 (Hsp90) inhibitor	1
NCGC00179501-03	Aurantiamarin (Methyl Hesperidin)	Free Radical Scavenger	0
NCGC00390805-02	Icatibant	Bradykinin B2 receptor Antagonist	0
NCGC00386871-03	ML340	Zinc finger protein GLI1 Inhibitor	1
NCGC00380162-01		<MOA Unknown> | Class: Sugar | Genus: N/A | Family: N/A | Species: N/A	1
NCGC00015771-13	Orphenadrine dihydrogen citrate	Muscarinic Antagonists	0
NCGC00016747-07	Dilazep	Equilibrative nucleoside transporter 2 Inhibitor	0
NCGC00091469-17	Saquinavir Mesylate		0
NCGC00015925-12	Sulfaphenazole	Antibiotic	0
NCGC00015162-10	Buspirone	Serotonin 1a (5-HT1a) receptor Partial Agonist	0
NCGC00015004-13	Amoxapine	5-HT2A Antagonists?	0
NCGC00346938-02	E5555	Prothrombin inhibitor	0
NCGC00091534-02	o-Cresol		0
NCGC00094572-12	Amitraz		0
NCGC00181080-01	Phenacaine hydrochloride	"Calcium/calmodulin-dependent 3',5'-cyclic nucleotide phosphodiesterase 1A Inhibitor"	0
NCGC00167430-04	Tioconazole	Anti-fungal	0
NCGC00016418-23	Methoxsalen	DNA Intercalating Drug	0
NCGC00510519-01	MI-538	Menin/MLL Interaction inhibitor	0
NCGC00188380-05	Refametinib	Dual Specificity Mitogen-Activated Protein Kinase Kinase 1 inhibitor	0
NCGC00385658-01		<MOA Unknown> | Class: Steroid | Genus: Asparagus | Family: Liliaceae | Species: racemosus	1
NCGC00181131-04	Epothilone B	Microtubule-Stabilizing agent	0
NCGC00344531-01	Droxidopa	Adrenergic receptor Agonist	0
NCGC00386370-01	VS-5584	mTOR Complex 1 (mTORC1) inhibitor	1
NCGC00344535-01	5alpha-Cholestan-3beta-ol	Sterol regulatory element-binding protein cleavage-activating protein Binding Agent	0
NCGC00015260-22	CEFACLOR		0
NCGC00346539-05	CCT-129202	Aurora kinase A inhibitor	0
NCGC00510946-01	Modufolin	Tetrahydrofolate mimetic	1

NCGC00179594-03	Cefotiam hydrochloride	Escherichia coli Inhibitor	0
NCGC00015823-17	Piroxicam	Cyclooxygenase Inhibitor	0
NCGC00159331-07	CHOLECALCIFEROL	Vitamin D receptor Binding Agent	0
NCGC00263089-07	Barasertib	Aurora kinase B inhibitor	0
NCGC00182994-01	Anacolin		0
NCGC00509913-01	LF3	Beta-catenin/TCF interaction inhibitor	0
NCGC00390557-01	IRAK inhibitor 4 (trans)	Interleukin-1 receptor-associated kinase 4 Inhibitor	1
NCGC00015598-16	beta-Lapachone	Tryptophan 2,3-Dioxygenase inhibitor	0
NCGC00015254-20	Clozapine	Dopamine D4 Antagonist	0
NCGC00015121-17	Phenoxybenzamine hydrochloride	Alpha-1A Adrenergic Receptor antagonist	0
NCGC00181110-02	Cambendazole	glucose transport Inhibitor	1
NCGC00389282-01	Indacaterol Maleate	Beta-2 adrenergic receptor Agonist	0
NCGC00185736-03	Posaconazole	Lanosterol 14alpha-demethylase Inhibitor	1
NCGC00263205-02	Motesanib	Vascular Endothelial Growth Factor Receptor 2 (VEGFR-2) inhibitor	0
NCGC00390592-01	BMS-983970	Notch Signaling inhibitor	0
NCGC00015883-11	Risperidone	Dopamine D2 Antagonist	0
NCGC00018097-06	Paroxypropione	Succinate semialdehyde dehydrogenase Inhibitor	0
NCGC00015205-10	PK 11195	Translocator protein (TSPO) inhibitor	0
NCGC00164582-02	Feprazone		0
NCGC00378593-08	Alvelestat (AZD9668????	Neutrophil Elastase inhibitor	0
NCGC00013364-03	Vitamin B6		0
NCGC00345811-02	LLL-12	Signal Transducer and activator of Transcription 3 inhibitor	1
NCGC00159414-01	p-Menthane-1,8-diol monohydrate		0
NCGC00015861-09	Propentofylline	Adenosine receptor Modulator	0
NCGC00522633-01	Xanthatin	NF-kappaB (NFKB) Activation inhibitor	0
NCGC00167496-01	Methylene blue	Amine oxidase A inhibitor	1
NCGC00345830-02	PD-173955 Analogue 1	Proto-oncogene tyrosine-protein kinase Src inhibitor	0
NCGC00496840-01	BI-7273	Bromodomain-Containing Protein 7 (Brd7) inhibitor	0
NCGC00384181-09	SP-2509	Lysine-Specific Histone Demethylase 1A inhibitor	1
NCGC00253602-01	Thenyldiamine		0
NCGC00016743-08	Piromidic acid		0
NCGC00024995-43	Paclitaxel	Microtubule-Stabilizing agent	1
NCGC00178778-07	DIETHYLCARBAMAZINE CITRATE	0	
NCGC00263187-07	TAK-733	Dual Specificity Mitogen-Activated Protein Kinase Kinase 1 inhibitor	0
NCGC00166079-02	C.I. Solvent Red 24		0
NCGC00378736-01	Cinepazide Maleate	Voltage-gated calcium channel Blocker	0
NCGC00346953-01	JNK Inhibitor V	JNK Inhibitor	0

NCGC00481317-01	CB-5083	Valosin-containing protein (VCP) p97 / Transitional endoplasmic reticulum ATPase inhibitor	1
NCGC00510481-01	GSK-682753A	G-protein Coupled Receptor 183 Inverse agonist	0
NCGC00094887-05	Fenoprofen calcium salt dihydrate	Cyclooxygenase-2 Inhibitor	0
NCGC00263951-01	CEPHRADINE	Cephalosporin antibiotic	0
NCGC00185745-01	Cefroxadine		0
NCGC00015608-22	Loperamide hydrochloride	Mu-opiod receptor agonist	0
NCGC00241410-02	NCGC00241410	Interleukin-1 Receptor-Associated Kinase 1 inhibitor	0
NCGC00160423-02	Bamethan		0
NCGC00015870-27	Quercetin	Catechol-O-Methyltransferase inhibitor	0
NCGC00015859-14	Procainamide	Sodium channel alpha subunit Blocker	0
NCGC00159424-04	Tetramethylthiuram monosulfide		1
NCGC00016613-10	Antazoline hydrochloride	Histamine H1 receptor Antagonist	0
NCGC00186047-02	IPA-3	Serine/threonine-protein kinase PAK 1 inhibitor	0
NCGC00095089-10	Fluconazole	Anti-fungal	0
NCGC00168747-01	Methoxyflurane	synaptic transmission Inhibitor	0
NCGC00389668-02	mTORInhibitor,Ku-0063794	mTORC1/2 Inhibitor	1
NCGC00263166-10	Obatoclax	Apoptosis regulator Bcl-2 inhibitor	1
NCGC00346618-07	Omecamtiv mecarbil	Cardiac Myosin activator	0
NCGC00188435-01	2-Hydroxyethyl octadecanoate		0
NCGC00188442-01	Estradiol enanthate		0
NCGC00351593-05	GDC-0623	Dual Specificity Mitogen-Activated Protein Kinase Kinase inhibitor	0
NCGC00188443-01	Dapiprazole hydrochloride	Adrenergic Receptor Alpha-1 Antagonist	0
NCGC00346882-02	AS-1949490	Phosphatidylinositol 3,4,5-Trisphosphate 5-Phosphatase 2 inhibitor	0
NCGC00481564-01	NPS-1034	Hepatocyte Growth Factor Receptor inhibitor	0
NCGC00095227-06	Alizarin	Calcium Binding Agent	0
NCGC00015826-13	Perphenazine	Muscarinic acetylcholine receptor M1 Antagonist	0
NCGC00164076-03	SODIUM GLUCONATE		0
NCGC00387875-03	Voxtalisib	mTOR Complex 1 (mTORC1) inhibitor	1
NCGC00346465-06	GDC-0349	Serine/threonine-protein kinase pim-3 inhibitor	0
NCGC00181290-01	Furazabol		0
NCGC00090749-12	Diethylstilbestrol	Estrogen Receptor agonist	0
NCGC00016089-06	Nonivamide	Vanilloid receptor Agonist	0
NCGC00094906-06	Nefopam hydrochloride	Serotonin transporter Inhibitor	0
NCGC00182977-01	Trimethoquinol	Beta-3 adrenergic receptor Agonist	0
NCGC00263610-05	CAY10398		1
NCGC00378460-08	GKT-137831	NADPH Oxidase 4 inhibitor	0
NCGC00016737-04	Epirizole		0

NCGC00384250-02	GNE-3511	Mitogen-Activated Protein Kinase Kinase Kinase 12 inhibitor	1
NCGC00015420-15	Emodin	Bifunctional epoxide hydrolase 2 inhibitor	0
NCGC00246799-02			1
NCGC00346629-09	GSK1349572	HIV Integrase Inhibitors	0
NCGC00386317-04	Ribociclib (LEE011)	Cyclin-Dependent Kinase 4 inhibitor	0
NCGC00167434-02	Pemirolast potassium		0
NCGC00347423-03		Sodium/potassium-transporting ATPase Inhibitor	1
NCGC00181354-02	Tocoretinate	Retinoic acid receptor gamma Agonist	0
NCGC00480876-01	T-00127-HEV1	Phosphatidylinositol 4-Kinase beta inhibitor	0
NCGC00390577-02	Omaveloxolone	Nuclear Factor Erythroid 2-Related Factor 2 activator	1
NCGC00159438-03	L-selenomethionine	Antioxidant	0
NCGC00387133-01	5 -Fluoroindirubinoxime		1
NCGC00160664-04	Nitroxoline	Methionine Aminopeptidase-2 inhibitor	0
NCGC00345018-01	D-159687	Phosphodiesterase IIII D (PDE4D) Inhibitor	0
NCGC00015785-10	Progesterone	Progesterone Receptor agonist	0
NCGC00015735-11	Niclosamide	ATP synthase inhibitor	1
NCGC00178877-03	Methicillin sodium hydrate	peptidoglycan biosynthetic process Inhibitor	0
NCGC00271789-11	ActinomycinD	Anti-bacterial	0
NCGC00163381-01	KN-62	CaMK-II Inhibitor	0
NCGC00390656-02	Pimavanserin		0
NCGC00242504-01	Cortivazol	Glucocorticoid Receptor (GR) Agonist	0
NCGC00263212-05	SJ-172550	E3 Ubiquitin-Protein Ligase Mdm4 (Hdm4) inhibitor	0
NCGC00016692-05	Bromperidol	Dopamine D2 receptor Antagonist	0
NCGC00384274-01	Indirubin_E8		1
NCGC00015026-16	Atropine (sulfate monohydrate)		0
NCGC00015216-17	Chlorpropamide	"Sulfonylurea receptor 1, Kir6.2 Inhibitor"	0
NCGC00346440-02	Sumanirole maleate	Dopamine Receptor D2 agonist	0
NCGC00179623-04	Lisinopril dihydrate	Angiotensin-converting enzyme Inhibitor	0
NCGC00510355-01	Mavacamten	Myosin-binding protein C modulator	0
NCGC00164283-07	Hexadecyltrimethylammonium bromide	Cell membrane Interacts	0
NCGC00159390-14	PERMETHRIN	Voltage-sensitive sodium channel Inhibitor	0
NCGC00159366-02	m-Cresol		0
NCGC00016504-09	Droperidol	HERG Inhibitor	0
NCGC00015836-12	Pirenzepine	Muscarinic acetylcholine receptor M1 Antagonist	0
NCGC00095150-16	Telmisartan	Angiotensin AT1 Receptor antagonist	0
NCGC00016257-10	4-Dimethylaminoantipyrine		0
NCGC00249888-01	Vitamin B12	Coenzyme A Stimulant	0
NCGC00183008-01	Israpafant	Interleukin-5 Inhibitor	0

NCGC00015830-08	Phenelzine	Amine oxidase A inhibitor	0
NCGC00509989-01	MK-0557	Neuropeptide Y Receptor Type 5 antagonist	0
NCGC00347065-03	KUC107887N	Valosin-containing protein (VCP) p97 / Transitional endoplasmic reticulum ATPase inhibitor	0
NCGC00346689-06	NVP-BVU-972	Hepatocyte Growth Factor Receptor inhibitor	0
NCGC00179867-02			0
NCGC00016896-06	Amrinone	platelet aggregation Inhibitor	0
NCGC00488771-02	BAY-1436032	Isocitrate Dehydrogenase [NADP] cytoplasmic (Arg132His Mutant) inhibitor	0
NCGC00510501-01	Zanubrutinib	Bruton's Tyrosine Kinase (BTK) inhibitor	0
NCGC00379214-02	WIKI4	Tankyrase-1 inhibitor	0
NCGC00015110-09	Aminoglutethimide	Aromatase inhibitor	0
NCGC00165915-05	KDS-4103	Fatty Acid Amide Hydrolase inhibitor	0
NCGC00013565-03	D-Leucine	Mammalian target of Rapamycin (mTORC1) Activator	0
NCGC00386721-01	PF-956980	Tyrosine-protein kinase JAK3 inhibitor	0
NCGC00025346-08	Mirtazapine	5-HT2A Receptor antagonist	0
NCGC00522039-01	Pyrethrin 1	Trypanosoma brucei rhodesiense Inhibitor	0
NCGC00263211-04	JK-184	Smoothened Receptor antagonist	0
NCGC00167449-02	Desogestrel	Progesterone receptor Agonist	0
NCGC00159549-16	Zebularine	Cytidine Deaminase inhibitor	0
NCGC00015501-05	Apresoline	Myeloperoxidase inhibitor	0
NCGC00092381-04	ABT-702	Adenosine Kinase inhibitor	0
NCGC00346946-03	CDK9 Inhibitor	Cyclin-Dependent Kinase 9 inhibitor	0
NCGC00181775-02	Toluidine blue		0
NCGC00182073-03	Nedocromil	mast cell degranulation Inhibitor	0
NCGC00370979-01	AMG-548	Tumor necrosis factor alpha Production inhibitor	0
NCGC00013289-09	Pridinol	Muscarinic acetylcholine receptor Antagonist	0
NCGC00024196-12	Megestrol acetate	Progesterone Receptor agonist	0
NCGC00091449-10	Eugenol	reactive oxygen species biosynthetic process Modulator	0
NCGC00181785-01	Aprepitant	Tachykinin receptor 1 antagonist	0
NCGC00015207-12	Cilostazol	Phosphodiesterase 3A inhibitor	0
NCGC00346941-01	ITK Inhibitor	ITK Inhibitor	0
NCGC00344529-02	Cefamandole (nafate)	Bacterial Penicillin-binding Protein Inhibitor	0
NCGC00263445-02	Brucine-N-oxide	Vascular endothelial growth factor receptor 2 Inhibitor	0
NCGC00510891-01	Cefacetrile		0
NCGC00168290-05	Avermectin B1a		0
NCGC00018166-02	Estradiol valerate		0
NCGC00380410-01		<MOA Unknown> | Class: trichothecene | Genus: N/A | Family: N/A | Species: N/A	1

NCGC00510269-04	AZD-5153 6-Hydroxy-2-naphthoic acid	Bromodomain-Containing Protein 4 (Brd4) inhibitor	0
NCGC00509909-01	Avadomide	Cereblon inhibitor	0
NCGC00420892-01	NKH477	Adenylate cyclase Activator	1
NCGC00345809-03	RGB-286147	Cyclin-Dependent Kinase 7 inhibitor	0
NCGC00532509-01	ISAVUCONAZONIUM SULFATE	Lanosterol 14-alpha demethylase Inhibitor	0
NCGC00159459-04	Mycophenolate mofetil	Inosine 5'-Monophosphate Dehydrogenase inhibitor	0
NCGC00015428-15	(+/-) -Fluoxetine	Serotonin transporter Inhibitor	0
NCGC00016456-20	Chrysine	Antiinflammatory Drug	0
NCGC00162453-19	Trichostatin A	Cyclin-Dependent Kinase inhibitor 1 Expression Enhancer	1
NCGC00346962-03	HMSL10077	Bruton's Tyrosine Kinase (BTK) inhibitor	1
NCGC00165736-04	Acadesine	AMP-Activated Protein Kinase (AMPK) activator	0
NCGC00178499-08	Cefdinir	Cephalosporin antibiotic	0
NCGC00510167-01	THZ531	Cyclin-Dependent Kinase 12 inhibitor	0
NCGC00142400-07	Chenodeoxycholic acid	11-beta-Hydroxysteroid Dehydrogenase Type 1 inhibitor	0
NCGC00496910-01	Daprodustat	Hypoxia-Inducible Factor Prolyl Hydroxylase inhibitor	0
NCGC00165912-03	Tofisopam		0
NCGC00168774-02	Rasagiline mesylate	Amine oxidase B inhibitor	0
NCGC00091493-09	Anethole	TNF signaling pathway Inhibitor	0
NCGC00507889-01	Mivebresib (ABBV-075)	Bromodomain-Containing Protein 4 (Brd4) inhibitor	0
NCGC00018158-08	Doxazosin mesylate	alpha1-Adrenoceptor Antagonists	0
NCGC00346678-15	Ganetespib (STA-9090)	Heat Shock Protein 90 (Hsp90) inhibitor	0
NCGC00249893-01	Lodoxamide	type I hypersensitivity Inhibitor	0
NCGC00387824-01	CUDC-305	Heat Shock Protein 90 (hsp90) inhibitor	1
NCGC00015862-07	Pyridostigmine bromide	Acetylcholinesterase Inhibitor	0
NCGC00345806-02	Cathepsin S Inhibitor	Cathepsin S inhibitor	0
NCGC00090787-08	Benzophenone		0
NCGC00346567-12	AZD-8330	Dual Specificity Mitogen-Activated Protein Kinase Kinase inhibitor	0
NCGC00182997-01	Melinamide	Acyl coenzyme A:cholesterol acyltransferase Inhibitor	0
NCGC00091575-03	2-Ethyl-1,3-hexanediol		0
NCGC00483927-02	ARV-825	Bromodomain-Containing Protein 4 (Brd4) Degradation inducer	1
NCGC00378847-01	B-Raf inhibitor	Serine/threonine-protein kinase B-raf Inhibitor	1
NCGC00264046-02	Glutathione	"Glutathione reductase, mitochondrial Substrate"	0
NCGC00015453-08	Fexofenadine hydrochloride	Histamine H1 receptor Antagonist	0
NCGC00183011-01	Itasetron	Serotonin 3 (5-HT3) receptor Antagonist	0
NCGC00522455-01	SJG-136	DNA Alkylating Drug	0
NCGC00179526-05	Demecarium bromide	Acetylcholinesterase Inhibitor	0

NCGC00510267-01	PF-06821497	Histone-lysine N-methyltransferase EZH2 inhibitor	0
NCGC00384179-01	NMS-859	Valosin-containing protein (VCP) p97 / Transitional endoplasmic reticulum ATPase inhibitor	0
NCGC00347941-04	CASIN	Cell Division Control Protein 42 Homolog inhibitor	0
NCGC00486911-01	Tilfrinib		1
NCGC00016570-06	Mephentermine hemisulfate		0
NCGC00183848-01	Troxacitabine	DNA-Directed DNA Polymerase inhibitor	0
NCGC00178510-09	Roxithromycin	Bacterial 70S ribosome Inhibitor	0
NCGC00187482-21	Pictilisib	Phosphatidylinositol 3-Kinase alpha isoform inhibitor	1
NCGC00263084-02	Hh-Ag1.5	Smoothened Receptor agonist	0
NCGC00346673-02	Milciclib	Cyclin-Dependent Kinase 1 inhibitor	1
NCGC00521942-01	Milademetan	E3 Ubiquitin-Protein Ligase Mdm2 (Hdm2) inhibitor	1
NCGC00184998-02	Dopexamine	D(2) dopamine receptor Agonist	0
NCGC00507861-01	FRAX-1036	Serine/threonine-protein kinase PAK 2 inhibitor	1
NCGC00017246-10	PICROTIN		0
NCGC00017324-07	Simvastatin	HMG-CoA Reductase inhibitor	1
NCGC00164526-02	Camylofin	Muscarinic acetylcholine receptor Antagonist	0
NCGC00183833-01	Bimosiamose	Selectin E Inhibitor	0
NCGC00160567-01	Acetomenaphthone		0
NCGC00263566-03	Oteracil potassium	Orotate phosphoribosyltransferase Inhibitor	0
NCGC00346652-08	PF-03814735	Aurora kinase A inhibitor	1
NCGC00015208-19	Caffeine	Chitinase Inhibitor	0
NCGC00183000-01	Lanperisone hydrochloride	Sodium channel alpha subunit Binding Agent	0
NCGC00091455-09	Celecoxib	Cyclooxygenase 2(COX2) inhibitor	0
NCGC00346645-06	TAK-901	Aurora kinase B inhibitor	1
NCGC00346621-05	CCT-128930	AKT serine/threonine kinase inhibitor	0
NCGC00095118-08	Rofecoxib	Cyclooxygenase-2 Inhibitor	0
NCGC00167479-01	Bicisate dihydrochloride		0
NCGC00015455-10	Felodipine	Voltage-gated calcium channel alpha2/delta subunit 1 Inhibitor	0
NCGC00386401-01	Ravoxertinib	Mitogen-Activated Protein Kinase 3 (ERK1) inhibitor	0
NCGC00016361-21	Acetaminophen	Cyclooxygenase-3 Inhibitor	0
NCGC00094960-06	Anthraquinone	Beta amyloid A4 protein Binding Agent	0
NCGC00181083-01	Piperilate hydrochloride		0
NCGC00178032-03	CITICOLINE		0
NCGC00346679-10	GW-788388	TGF-beta receptor type-1 inhibitor	0
NCGC00185770-01	Sucrose octasulfate, sodium salt		0
NCGC00167515-03	Enocitabine	DNA Replication inhibitor	0

NCGC00166397-04	Lofepramine hydrochloride	norepinephrine uptake Inhibitor	0
NCGC00346930-01	Picoplatin	DNA Alkylating Drug	0
NCGC00167773-13	TPCA-1	inhibitor of nuclear factor kappa B kinase subunit beta inhibitor	1
NCGC00167476-02	Calcium hopantenate		0
NCGC00023217-08	Meclofenamate sodium	Cyclooxygenase Inhibitor	0
NCGC00094561-06	Phenothrin		0
NCGC00091616-03	2-Ethoxybenzamide		0
NCGC00481544-01	Cetrorelix	Gonadotropin-releasing hormone receptor Antagonist	0
NCGC00016092-09	WAY-100635	5-HT1A Receptor Antagonist	0
NCGC00261967-01	Prednisolone farnesylate	Glucocorticoid and meneralocorticoid receptor agonists/antagonists Agonist	0
NCGC00346504-07	Avagacestat	gamma-Secretase inhibitor	0
NCGC00179506-05	Kanamycin (sulfate)	30S Ribosomal Protein Inhibitors	0
NCGC00229735-09	KU-60019	ATM Kinase inhibitor	0
NCGC00174046-02	Imidocarb dipropionate	Adenosine deaminase Inhibitor	0
NCGC00346693-02	BMS-265246	Cyclin-Dependent Kinase 1 inhibitor	1
NCGC00387217-01	CRT 0066101		1
NCGC00510496-01	VLX-1570	Ubiquitin Carboxyl-Terminal Hydrolase isozyme L5 inhibitor	1
NCGC00017112-14	Ciclopirox		1
NCGC00346477-02	Emricasan	Caspase (Pan) inhibitor	0
NCGC00482879-02	Tucatinib	Receptor tyrosine-protein kinase NEU (HER2; erbB2) inhibitor	0
NCGC00386309-03	WZ-4003	NUAK family SNF1-like kinase 1 inhibitor	0
NCGC00346595-02	Oridonin	Apoptosis regulator Bcl-2 Expression inhibitor	0
NCGC00168784-03	Gemcitabine	DNA Polymerase inhibitor	0
NCGC00163440-02	CYTOCHALASIN D	actin polymerization or depolymerization Inhibitor	1
NCGC00091057-08	Silybin		0
NCGC00160471-02	Crocin?	Tyrosinase Inhibitor	0
NCGC00182705-02	Azatadine	Histamine H1 receptor Antagonist	0
NCGC00167533-02	Vardenafil	Phosphodiesterase V (PDE5) Inhibitor	0
NCGC00248225-03	9(Z)-Hexadecenoic acid	Gap junction Modulator	0
NCGC00242491-03	SB-265610	Chemokine CXCR2 Receptor (IL-8 beta Receptor) antagonist	0
NCGC00164625-04	Cerivastatin sodium	HMG-CoA reductase Inhibitor	1
NCGC00263237-09	JZL-184	Monoglyceride Lipase inhibitor	0
NCGC00507865-01	ONO-4059 (GS-4059)	Bruton's Tyrosine Kinase (BTK) inhibitor	0
NCGC00168108-05	AG-14361	Poly [ADP-ribose] polymerase 1 inhibitor	0
NCGC00378788-02	SB 699551 dihydrochloride	Serotonin 5a (5-HT5a) receptor Antagonist	1
NCGC00379163-01	Atiprimod	Vascular endothelial growth factor secretory inhibitor	1
NCGC00094981-07	Benzyl benzoate		0

<table>
<tr><td>NCGC00384881-02</td><td></td><td><MOA Unknown> | Class: Saponin | Genus: N/A | Family: N/A | Species: N/A</td><td>1</td></tr>
<tr><td>NCGC00018139-05</td><td>Ipriflavone</td><td>Free Radical Scavenger</td><td>0</td></tr>
<tr><td>NCGC00182033-01</td><td>Felypressin</td><td>Vasopressin V1a receptor Agonist</td><td>0</td></tr>
<tr><td>NCGC00159406-15</td><td>Noradrenaline bitartrate monohydrate</td><td>Alpha-1A Adrenergic Receptor agonist</td><td>0</td></tr>
<tr><td>NCGC00346542-10</td><td>Hesperadin</td><td>Aurora kinase A inhibitor</td><td>1</td></tr>
<tr><td>NCGC00274074-01</td><td>2-(Dodecyloxy)ethanol</td><td></td><td>0</td></tr>
<tr><td>NCGC00378600-02</td><td>Glasdegib</td><td>Smoothened Receptor antagonist</td><td>0</td></tr>
<tr><td>NCGC00345837-03</td><td>GNE-490</td><td>Phosphatidylinositol 3-Kinase beta isoform inhibitor</td><td>1</td></tr>
<tr><td>NCGC00090757-12</td><td>Melphalan</td><td>DNA Alkylating Drug</td><td>0</td></tr>
<tr><td>NCGC00371130-03</td><td>Vilazodone Hydrochloride</td><td>Sodium-dependent serotonin transporter Inhibitor</td><td>0</td></tr>
<tr><td>NCGC00181773-05</td><td>Parecoxib</td><td>Cyclooxygenase-2 Inhibitor</td><td>0</td></tr>
<tr><td>NCGC00253575-01</td><td>Arimoclomol</td><td>Heat shock 70 kDa protein 1 Activator</td><td>0</td></tr>
<tr><td>NCGC00507887-01</td><td>ML-390</td><td>Dihydroorotate Dehydrogenase inhibitor</td><td>0</td></tr>
<tr><td>NCGC00025172-03</td><td>Roquinimex</td><td>Tumor necrosis factor Secretion modulator</td><td>0</td></tr>
<tr><td>NCGC00167472-03</td><td>Amlexanox</td><td>Mediator Release inhibitor</td><td>1</td></tr>
<tr><td>NCGC00686699-01</td><td>S(-)-Verapamil hydrochloride hydrate</td><td>0</td><td></td></tr>
<tr><td>NCGC00015595-14</td><td>Labetalol HCl</td><td>Adrenergic receptor Antagonist</td><td>0</td></tr>
<tr><td>NCGC00016934-06</td><td>Nizatidine</td><td>Histamine H2 receptor Antagonist</td><td>0</td></tr>
<tr><td>NCGC00249416-01</td><td>Oberadilol</td><td>Adrenergic receptor beta Antagonist</td><td>0</td></tr>
<tr><td>NCGC00094358-38</td><td>Isotretinoin</td><td>Retinoic Acid Receptor beta agonist</td><td>0</td></tr>
<tr><td>NCGC00181140-02</td><td>Carboquone</td><td>cell growth Inhibitor</td><td>0</td></tr>
<tr><td>NCGC00159504-04</td><td>Thymol iodide</td><td></td><td>0</td></tr>
<tr><td>NCGC00263193-02</td><td>KI-20227</td><td>Colony Stimulating Factor 1 Receptor inhibitor</td><td>0</td></tr>
<tr><td>NCGC00025195-06</td><td>Ozagrel hydrochloride</td><td>Thromboxane Synthase inhibitor</td><td>0</td></tr>
<tr><td>NCGC00253598-01</td><td>Sulfacytine</td><td>Dihydropteroate synthetase Inhibitor</td><td>0</td></tr>
<tr><td>NCGC00507698-01</td><td>Talaporfin sodium</td><td></td><td>0</td></tr>
<tr><td>NCGC00510503-01</td><td>Elacestrant</td><td>Selective Estrogen Receptor modulator (SERM)</td><td>0</td></tr>
<tr><td>NCGC00263565-06</td><td>Gestodene</td><td>Progesterone Receptor agonist</td><td>0</td></tr>
<tr><td>NCGC00510690-01</td><td>GDC-0326</td><td>Phosphatidylinositol 3-Kinase alpha isoform inhibitor</td><td>0</td></tr>
<tr><td>NCGC00386151-01</td><td>Tie2 kinase inhibitor</td><td>Angiopoietin-1 Receptor inhibitor</td><td>0</td></tr>
<tr><td>NCGC00165993-02</td><td>Picolinic acid</td><td></td><td>0</td></tr>
<tr><td>NCGC00166140-02</td><td>Pyrithioxin dihydrochloride</td><td>acetylcholine biosynthetic process Activator</td><td>0</td></tr>
<tr><td>NCGC00346070-02</td><td>GSK-2606414</td><td>Protein kinase R (PKR)-like endoplasmic reticulum kinase (PERK)</td><td>0</td></tr>
<tr><td>NCGC00016373-08</td><td>Benzethonium chloride</td><td></td><td>0</td></tr>
<tr><td>NCGC00183682-14</td><td>VX-765</td><td>Caspase-1 inhibitor</td><td>0</td></tr>
<tr><td>NCGC00346469-03</td><td>Delanzomib</td><td>Proteasome inhibitor</td><td>1</td></tr>
<tr><td>NCGC00242501-03</td><td>ITX3</td><td>Triple Functional Domain Protein inhibitor</td><td>0</td></tr>
<tr><td>NCGC00181042-02</td><td>Menthyl salicylate</td><td></td><td>0</td></tr>
</table>

NCGC00387848-03	IPI-145	PI3-kinase p110-gamma subunit Inhibitor	0
NCGC00018242-07	Sulfacetamide	Bacterial dihydropteroate synthase Inhibitor	0
NCGC00347822-02		<MOA Unknown> | Class: Iridoid | Genus: Veronica | Family: Scrophulariaceae | Species: anagallis-aquatica	1
NCGC00179607-03	Chlorphensin carbamate		0
NCGC00016367-08	Succinylsulfathiazole		0
NCGC00018213-06	Cyclopentolate hydrochloride	Muscarinic acetylcholine receptor Antagonist	0
NCGC00091414-07	Salicylamide		0
NCGC00179514-05	Ceforanide	Bacterial penicillin-binding protein Inhibitor	0
NCGC00181124-02	Sparteine	sodium ion transport Inhibitor	0
NCGC00263694-01	Kartogenin	Chondrocyte Differentiation Initiator	0
NCGC00163157-05	Mesoridazine	D(2) dopamine receptor Antagonist	0
NCGC00015092-10	Aminocaproic acid	Plasminogen inhibitor	0
NCGC00242489-02	LG-100268	Retinoic Acid Receptor RXR-alpha agonist	0
NCGC00095003-07	Tioxolone	Carbonic Anhydrase 1 inhibitor	0
NCGC00018102-16	Flunarizine dihydrochloride	Calcium Channel Blocker	0
NCGC00263113-03	GYKI 53655	AMPA receptor antagonist	1
NCGC00183107-01			0
NCGC00183860-01	Mefruside	Carbonic anhydrase Inhibitor	0
NCGC00249936-01	Iocarmic acid		0
NCGC00507774-01	Cefbuperazone	Peptidoglycan biosynthesis Inhibitor	0
NCGC00167499-02	Roxatidine acetate hydrochloride		0
NCGC00023016-07	Cefmetazole sodium	Bacterial penicillin-binding protein Inhibitor	0
NCGC00378894-01	Talarozole (R enantiomer)	Steroid 17-alpha-hydroxylase/17,20 lyase inhibitor	0
NCGC00387248-01	Azixa	Tubulin polymerization inhibitor	1
NCGC00274064-01	Betamethasone valerate	Glucocorticoid steroid	0
NCGC00090693-11	D-Nicotine	Neuronal acetylcholine receptor; alpha4/beta2 Agonist	0
NCGC00346498-01	Danoprevir	HCV Protease Inhibitor	0
NCGC00186658-01	Dexpanthenol		0
NCGC00015122-11	Bupropion hydrochloride	Sodium-dependent Dopamine Transporter inhibitor	0
NCGC00346570-04	Tebipenem pivoxil	Carbapenem Antibiotic	0
NCGC00160428-03	Indibulin	Tubulin polymerization inhibitor	0
NCGC00242479-03	Zibotentan	Endothelin-1 Receptor antagonist	0
NCGC00024257-10	Phenylephrine hydrochloride	Alpha-1A Adrenergic Receptor ligand	0
NCGC00183040-01			0
NCGC00159361-06	Menthyl Anthranilate		0
NCGC00184993-03	Amfenac Sodium	Cyclooxygenase-2 Inhibitor	0
NCGC00015227-08	Dexchlorpheniramine hydrochloride	Histamine H1 Receptor Antagonists; SNRI	0
NCGC00390707-01	Oclacitinib	Tyrosine-protein kinase JAK3 Inhibitor	0

NCGC00386839-01			1
NCGC00381477-05	SAR-405	Phosphatidylinositol 3-Kinase Type 3 inhibitor	0
NCGC00018143-05	Enrofloxacin	Topoisomerase IV Inhibitor	0
NCGC00016636-05	Flavoxate hydrochloride	Muscarinic acetylcholine receptors; M1 & M2 Antagonist	0
NCGC00249390-02	Tivozanib (AV-951)	Vascular Endothelial Growth Factor Receptor 2 (VEGFR-2) inhibitor	1
NCGC00384920-01		<MOA Unknown> | Class: Steroid | Genus: N/A | Family: N/A | Species: N/A	1
NCGC00164607-01	Bentiromide sodium		0
NCGC00378564-01	Ro-28-1675	Glucokinase activator	0
NCGC00485430-01	PF-06250112	Bruton's Tyrosine Kinase (BTK) inhibitor	0
NCGC00183110-02	Imidafenacin	potassium ion transport Inhibitor	0
NCGC00025245-05	Ginkgolide B	Platelet-Activating Factor Receptor antagonist	0
NCGC00345461-06	Gandotinib	Tyrosine-protein kinase JAK2 inhibitor	1
NCGC00167973-02	Eperisone hydrochloride	skeletal muscle contraction Inhibitor	0
NCGC00179276-04	Etilefrine hydrochloride		0
NCGC00186030-12	AS-605240	Phosphatidylinositol 3-Kinase gamma isoform inhibitor	0
NCGC00182983-01	Fezatione		0
NCGC00160355-01			0
NCGC00387035-02	SAR-020106	Checkpoint kinase 1 inhibitor	1
NCGC00186009-04	AS-604850	Phosphatidylinositol 3-Kinase gamma isoform inhibitor	0
NCGC00385253-01		<MOA Unknown> | Class: Terpenoid | Genus: Begonia | Family: Begoniaceae | Species: keniensis	1
NCGC00378902-02	Toceranib	Vascular Endothelial Growth Factor Receptor 2 (VEGFR-2) inhibitor	0
NCGC00167328-03	Aloxistatin	Calpain inhibitor	0
NCGC00016914-06	CEFOTETAN	Bacterial Penicillin-binding Protein Inhibitor	0
NCGC00167538-02	Clorprenaline hydrochloride	Beta-2 adrenergic receptor Agonist	0
NCGC00390695-02	EMD-638683	Serine/threonine-protein kinase Sgk1 inhibitor	0
NCGC00183677-03	Hypothemycin	Dual Specificity Mitogen-Activated Protein Kinase Kinase 1 inhibitor	0
NCGC00016528-05	Proguanil hydrochloride	DHFR inhibitor	0
NCGC00476094-01	Dexanabinol	NMDA Receptor antagonist	0
NCGC00499183-01	XMU-MP-1	Serine/threonine-protein kinase 4 inhibitor	1
NCGC00386015-01		<MOA Unknown> | Class: Triterpen | Genus: Iberis | Family: N/A | Species: contracta	1
NCGC00475765-01	Compound 36	IAP inhibitor	0
NCGC00024357-07	Scopolamine hydrobromide	Muscarinic Antagonists	0
NCGC00163150-07	Clenbuterol	Beta-2 adrenergic receptor Agonist	0
NCGC00024246-16	Daunorubicin	DNA Topoisomerase II inhibitor	0

NCGC00389618-01	BYL-719	PI3K alpha Inhibitor	0
NCGC00379007-02	Retapamulin	70S ribosome Inhibitor	0
NCGC00189075-02	PD-0325901	Dual Specificity Mitogen-Activated Protein Kinase Kinase 1 inhibitor	0
NCGC00483210-07			1
NCGC00346494-06	E-7010	Tubulin polymerization inhibitor	1
NCGC00483921-01	PLX-8394	Serine/threonine-protein kinase B-raf inhibitor	0
NCGC00346581-05	AZ-960	Tyrosine-protein kinase JAK3 inhibitor	1
NCGC00182982-01	Dalcotidine	Histamine H2 receptor Antagonist	0
NCGC00379102-02	UA62784	microtubule polymerization Inhibitor	1
NCGC00163135-04	Niguldipine	L-type Ca2+ channel Blocker	0
NCGC00182049-02	Nitroscanate	ATP biosynthetic process Inhibitor	0
NCGC00185994-09	10058-F4	Myc proto-oncogene protein inhibitor	0
NCGC00350264-01	PRP003-062	Succinate Receptor 1 inhibitor	0
NCGC00179596-11	Butoconazole nitrate	Anti-fungal	0
NCGC00386077-01		<MOA Unknown> | Class: N/A | Genus: N/A | Family: N/A | Species: N/A	1
NCGC00378635-03	Triptonide	dCTP Pyrophosphatase 1 inhibitor	1
NCGC00521081-01	Cycrimine Hydrochloride		0
NCGC00510476-01	Sulfatinib	Vascular Endothelial Growth Factor Receptor 2 (VEGFR-2) inhibitor	0
NCGC00496795-01	WO2016044770		1
NCGC00345799-06	Oprozomib	Proteasome inhibitor	1
NCGC00025207-15	HA-14-1	Apoptosis regulator Bcl-2 inhibitor	0
NCGC00263087-10	Volasertib	Serine/threonine-protein kinase PLK1 inhibitor	1
NCGC00346893-01	SL-0101-1	RSK Inhibitor	0
NCGC00370759-04	Pyrintegrin	BMP Signalling and Regulation Inhibitor	1
NCGC00091110-01	Glutaraldehyde		0
NCGC00386412-04	G749	Receptor-type tyrosine-protein kinase FLT3 Inhibitor	1
NCGC00164591-04	Artemether	Anti-malarial	0
NCGC00179239-05	Clocortolone pivalate		0
NCGC00162106-17	Cefsulodin Sodium Salt	Bacterial penicillin-binding protein Inhibitor	0
NCGC00386200-01	Vildagliptin (LAF-237)	Dipeptidyl peptidase IV Inhibitor	0
NCGC00018157-13	Flurbiprofen	non-steroidal antiinflammatory	0
NCGC00343768-05	Rabusertib	Checkpoint kinase 1 inhibitor	0
NCGC00263170-02	Taladegib	Smoothened Receptor antagonist	0
NCGC00025065-10	Cyclopiazonic acid from Penicillium cyclopium	Sarcoplasmic/endoplasmic reticulum calcium ATPase 3 Inhibitor	1
NCGC00179585-04	Rescinnamin	Serotonergic synapse Inhibitor	0
NCGC00476210-01	GNF-7	Bcr-Abl Kinase inhibitor	1
NCGC00179401-03	Ethotoin	Sodium channel protein type V alpha subunit Inhibitor	0
NCGC00346572-02	Avasimibe	Sterol O-acyltransferase inhibitor	1

NCGC00015643-11	Metoclopramide	Muscarinic acetylcholine receptor M3 Antagonist	0
NCGC00486045-01	Sofosbuvir D6	Lysosomal protective protein Substrate	0
NCGC00016014-09	Tyrphostin B42	Signal Transducer and activator of Transcription 3 inhibitor	0
NCGC00166289-02	Hydroxystilbamidine bis(methanesulfonate)	0	
NCGC00538141-01		Microsomal triglyceride transfer protein Inhibitor	0
NCGC00263017-01	CPG-52364	Toll-Like Receptor 9 antagonist	0
NCGC00345813-03	Icaritin	Estrogen Receptor agonist	0
NCGC00187906-15	PI-103	Phosphatidylinositol 3-Kinase beta isoform inhibitor	0
NCGC00509904-02	RO-5126766	Serine/threonine-protein kinase B-raf inhibitor	0
NCGC00188962-02	Pravastatin sodium	HMG-CoA Reductase inhibitor	0
NCGC00167980-02	Pranoprofen	prostaglandin biosynthetic process Inhibitor	0
NCGC00378976-03	Idasanutlin	E3 Ubiquitin-Protein Ligase Mdm2 (Hdm2) inhibitor	0
NCGC00015444-10	Fenoldopam	Dopamine Receptor D1 agonist	0
NCGC00015038-11	Aminopterin	Dihydrofolate Reductase inhibitor	0
NCGC00169326-03		<MOA Unknown> | Class: trichothecene | Genus: ct Fusarium | Family: N/A | Species: N/A	1
NCGC00386078-01		"Acetyl-CoA acetyltransferase, mitochondrial Inhibitor"	1
NCGC00506872-01	Acotiamide hydrochlorid	Acetylcholinesterase Inhibitor	0
NCGC00347285-06	MI-2	Menin/MLL Interaction inhibitor	0
NCGC00483168-02	EPZ-015866	Protein arginine N-methyltransferase 5 inhibitor	0
NCGC00018302-12	Trichlormethiazide	Solute carrier family 12 member 3 inhibitor	0
NCGC00371149-01	BX795	Serine/threonine-protein kinase TBK1 Inhibitor	1
NCGC00167502-01			0
NCGC00346697-02	842166X	Cannabinoid Receptor 2 agonist	0
NCGC00538146-01		Adrenergic receptor beta Blocker	0
NCGC00249350-02			1
NCGC00379254-06	KRCA-0008	ALK Tyrosine Kinase Receptor inhibitor	0
NCGC00163553-03	Evodiamine	Transient receptor potential cation channel subfamily V member 1 agonist	0
NCGC00480785-05	BLU-9931	Fibroblast Growth Factor Receptor 4 inhibitor	0
NCGC00390731-01	Tafamidis	Transthyretin Modulator	0
NCGC00351606-08	PRT-062607	Tyrosine-protein kinase SYK inhibitor	0
NCGC00507863-01	RSL3	Phospholipid hydroperoxide glutathione peroxidase inhibitor	0
NCGC00249920-01	Nandrolone cyclohexylpropionate	Androgen Receptor Agonist	0
NCGC00015977-06	SKF-89976A	Sodium and chloride-dependent GABA transporter 1 inhibitor	0

NCGC00167979-01	Methenolone enanthate		0
NCGC00092372-12	Necrostatin-1	Receptor-interacting serine/threonine-protein kinase 1 inhibitor	0
NCGC00347528-02	Rhodomyrtone A	<MOA Unknown> | Class: Polyketide | Genus: N/A | Family: N/A | Species: N/A	1
NCGC00165799-03	GW-6471	PPARalpha Antagonist	0
NCGC00016403-06	Mafenide hydrochloride	Carbonic anhydrase II Inhibitor	0
NCGC00179619-06	Erythromycin propionate	Anti-bacterial	0
NCGC00016886-04	Ioxaglic Acid	Thrombin Inhibitor	0
NCGC00263531-10	Linezolid	Anti-bacterial	0
NCGC00015479-22	Genistein	Short transient receptor potential channel 5 agonist	0
NCGC00532500-01	GABAPENTIN ENACARBIL		0
NCGC00024596-08	Carbidopa	decarboxylase inhibitor	0
NCGC00015238-13	Chlorzoxazone	Intermediate conductance calcium-activated potassium channel protein 4 Activator	0
NCGC00182544-01	Triamcinolone hexacetonide		0
NCGC00346592-02	Icariin	Phosphodiesterase V (PDE5) Inhibitor	0
NCGC00351607-02	GZD-824	Bcr-Abl Kinase inhibitor	1
NCGC00181773-01	Parecoxib sodium	Cyclooxygenase-2 Inhibitor	0
NCGC00159388-05	Pentaerythritol tetranitrate	Soluble guanylate cyclase Activator	0
NCGC00090548-04	Deferiprone	Iron Chelating Agent	0
NCGC00161679-02	MG-132	Proteasome inhibitor	1
NCGC00263020-01	NCGC00263020	Mitogen-Activated Protein Kinase Kinase Kinase 14 (NIK) inhibitor	0
NCGC00181765-03	Pranlukast hydrate	Cysteinyl leukotriene receptor 1 antagonist	0
NCGC00091070-02	Diisopropylamine	Pyruvate dehydrogenase kinase isoform 4 Inhibitor	0
NCGC00250374-02	Licofelone	Arachidonate 5-Lipoxygenase inhibitor	0
NCGC00508867-01	SAR-260301	Phosphatidylinositol 3-Kinase beta isoform inhibitor	0
NCGC00159491-08	Rimantadine	M2 Channel Inhibitor	0
NCGC00159435-03	Amiloxate		0
NCGC00241107-12	Bosutinib	Bcr-Abl Kinase inhibitor	1
NCGC00159319-04	Zonisamide	Na channel blocker, T-type Ca channel blocker	0
NCGC00178802-11	Deferoxamine mesylate	Metal Chelating Agent	0
NCGC00378610-02	Topiroxostat	Xanthine dehydrogenase Inhibitor	0
NCGC00159356-02	Nandrolone phenylpropionate	Steroid hormone	0
NCGC00346686-10	Tivantinib	Hepatocyte Growth Factor Receptor inhibitor	1
NCGC00249887-01	Indecainide	Sodium channel alpha subunit Blocker	0
NCGC00014925-06	Topotecan hydrochloride	DNA Topoisomerase I inhibitor	0
NCGC00164568-04	Montelukast sodium	Cysteinyl leukotriene receptor 1 Antagonist	0
NCGC00182055-03	Bazedoxifene	Selective Estrogen Receptor modulator (SERM)	0

NCGC00345810-01	Tetrahydrouridine	Cytidine Deaminase inhibitor	0
NCGC00509985-02	Sufugolix	Gonadotropin Releasing Hormone Receptor antagonist	0
NCGC00344515-02	Piperaquine	Anti-malarial	0
NCGC00159339-10	Streptomycin sulfate	30s Ribosomal Protein Inhibitors	0
NCGC00142615-10	Epinephrine	Alpha-1A Adrenergic Receptor agonist	0
NCGC00250391-05	AZD-7545	Pyruvate dehydrogenase (PDH) inhibitor	0
NCGC00167447-02	Prosultiamine	Apoptosis Activator	0
NCGC00159383-04	Propyl benzoate		0
NCGC00390238-01	Isepamicin	30S Ribosomal Protein Inhibitors	0
NCGC00166298-02	Melengestrol acetate		0
NCGC00249930-01	Tolycaine		0
NCGC00346577-02	RAF-265 derivative	Raf kinase B/C Inhibitor	0
NCGC00484056-01	Testosterone (undecanoate)		0
NCGC00263217-05	NVP-231	Ceramide Kinase inhibitor	1
NCGC00263114-01	SCH-900435	Sodium and chloride glycine transporter 1 inhibitor	0
NCGC00249897-01	Nitromersol		0
NCGC00015989-22	Thalidomide	Cereblon inhibitor	0
NCGC00183137-01	Sulfadiazine silver		0
NCGC00181137-01	Sulfathiourea		0
NCGC00015656-12	Mianserin	5-HT2A Antagonists?	0
NCGC00346625-03	CAY10505	Phosphatidylinositol 3-Kinase gamma isoform inhibitor	0
NCGC00179244-11	Dorzolamide hydrochloride	Carbonic Anhydrase 2 inhibitor	0
NCGC00346510-06	MGCD-265	Hepatocyte Growth Factor Receptor inhibitor	0
NCGC00496836-02	CC-223	mTOR Complex 1 (mTORC1) inhibitor	1
NCGC00018125-05	Tripelennamine citrate	Histamine H1 receptor Antagonist	0
NCGC00510497-02	Eribulin mesylate	Tubulin polymerization inhibitor	0
NCGC00378579-06	LCL-161	IAP inhibitor	0
NCGC00346550-03	Lumacaftor	CFTR Channel (DeltaF508 Mutant) Corrector	0
NCGC00015691-06	Cysteamine hydrochloride	Protein-glutamine gamma-glutamyltransferase 2 Inhibitor	0
NCGC00389304-01	Clindamycin Phosphate	Bacterial 70S ribosome Inhibitor	0
NCGC00090793-05	NAPHTHALENE, ZONE REFINED	Metabolism of xenobiotics by cytochrome P450 Substrate	0
NCGC00181760-01	Lobenzarit sodium	response to oxidative stress Modulator	0
NCGC00181099-01	Siccanin		0
NCGC00015466-11	Gabapentin	Voltage-dependent calcium channel subunit alpha-2/delta-1 ligand	0
NCGC00390633-02	Evofosfamide	DNA Alkylating Drug	0
NCGC00160596-03	Aluminum monostearate		0
NCGC00095787-05	(-)-Inosine		0
NCGC00167793-02	PD-198306	Mitogen-Activated Protein Kinase 3 (ERK1) inhibitor	1

NCGC00093356-10	Cytarabine	DNA-Directed DNA Polymerase inhibitor	0
NCGC00274275-01	Tripamide	Solute carrier family 12 member 2 Inhibitor	0
NCGC00182999-01	Flomoxef	Streptococcus pneumoniae Inhibitor	0
NCGC00015893-06	Ropinirole	Dopamine D3 receptor Partial Agonist	0
NCGC00167513-04	Vandetanib	Proto-oncogene tyrosine-protein kinase receptor Ret inhibitor	0
NCGC00167423-01	Bevonium metilsulfate	Muscarinic acetylcholine receptor Antagonist	0
NCGC00181914-01	Alloclamide		0
NCGC00016697-04	Trimetazidine dihydrochloride	"Trifunctional enzyme subunit beta, mitochondrial Inhibitor"	0
NCGC00015714-16	Nimodipine	Voltage-gated L-type calcium channel Antagonist	0
NCGC00244256-04	Lestaurtinib	Jak/Tyk/Flt Inhibitor	1
NCGC00015155-11	Bepridil	Voltage-dependent P/Q-type calcium channel subunit alpha-1A Blocker	0
NCGC00017338-03	Antimycin A	Cytochrome C reductase inhibitor	0
NCGC00532514-01	FLUPENTIXOL DECANOATE	Serotonin 2 (5-HT2) receptor Antagonist	0
NCGC00262578-03	Flucloxacillin sodium	Penicillin-binding protein Inhibitor	0
NCGC00183030-02	Terodiline hydrochloride	HERG Inhibitor	0
NCGC00253588-01	Gimatecan		1
NCGC00345781-01	NSC-678515	DNA Intercalating Drug	0
NCGC00346680-05	MK-8245	Acyl-CoA desaturase inhibitor	1
NCGC00016878-04	Piretanide	Cystic fibrosis transmembrane conductance regulator Antagonist	0
NCGC00371131-03	NMS-873	Valosin-containing protein (VCP) p97 / Transitional endoplasmic reticulum ATPase inhibitor	1
NCGC00346508-12	TG-100115	Phosphatidylinositol 3-Kinase alpha isoform inhibitor	0
NCGC00510190-02	BMS-687453	PPARalpha Agonist	0
NCGC00182711-02	Picosulfate sodium		0
NCGC00229511-10	Tofacitinib	Tyrosine-protein kinase JAK3 inhibitor	0
NCGC00475744-02	AZD-3147	mTOR Complex 1 (mTORC1) inhibitor	0
NCGC00378780-02	MRT-67307	Serine/threonine-protein kinase ULK1 inhibitor	0
NCGC00166115-02	Dibutyltin dilaurate		0
NCGC00181048-02	Dolasetron mesylate hydrate	Serotonin 3a (5-HT3a) receptor Antagonist	0
NCGC00347940-04	Bioymifi	Drug Acting on TRAIL Receptor	0
NCGC00186465-06	MK-2206	AKT serine/threonine kinase inhibitor	0
NCGC00095899-09	Rizatriptan Benzoate	5-HT1B Agonist	0
NCGC00347947-04	ITD-1	TGF-beta receptor type-1 inhibitor	1
NCGC00179619-17	Erythromycin	Anti-bacterial	0
NCGC00179274-03	Tribenoside		0
NCGC00351480-16	GSK-343	Histone-lysine N-methyltransferase EZH2 inhibitor	0
NCGC00510493-01	Briciclib	Eukaryotic translation initiation factor 4E inhibitor	0

NCGC00346841-07	LY-411575	gamma-Secretase inhibitor	0
NCGC00387813-02	Afatinib (BIBW2992)	EGFR (HER1; erbB1) inhibitor	1
NCGC00390570-02	HG-6-64-1	Serine/threonine-protein kinase B-raf inhibitor	1
NCGC00507885-01	UNC-3866	Chromodomain Y-like protein 2 inhibitor	0
NCGC00183872-01	Brotizolam		0
NCGC00092357-04	Sibutramine hydrochloride	Sodium-dependent Serotonin Transporter inhibitor	0
NCGC00351601-07	Otenabant	Cannabinoid Receptor 1 antagonist	0
NCGC00017354-07	Spectinomycin dihydrochloride	Antibiotic	0
NCGC00346615-02	TAME	Anaphase Promoting Complex (apc) inhibitor	0
NCGC00178501-04	CEFTIBUTEN	Peptidoglycan synthase FtsI Inhibitor	0
NCGC00263169-06	Plinabulin	Tubulin polymerization inhibitor	1
NCGC00184999-01	Sultopride hydrochloride	Dopamine D3 receptor Antagonist	0
NCGC00166134-02	Estradiol cypionate	Androgen Receptor Antagonist	0
NCGC00185755-03	Cefatrizine		0
NCGC00510234-01	Avitinib	Epidermal Growth Factor Receptor inhibitor	1
NCGC00166207-01	Cridanimod	Interferon inducer	0
NCGC00347067-04	KUC111774N	Microphthalmia-associated transcription factor Expression inhibitor	0
NCGC00390699-04	R-1530	Vascular Endothelial Growth Factor Receptor 3 (VEGFR-3) inhibitor	0
NCGC00166210-02	PAROMOMYCIN SULFATE	Bacterial 70S ribosome Inhibitor	0
NCGC00246113-02			0
NCGC00346513-02	Amonafide	DNA Topoisomerase II inhibitor	0
NCGC00163547-01	Ecdysone	Mas-Related G-protein Coupled Receptor MRG ligand	0
NCGC00386362-01	ARQ-621	Kinesin-Like Protein KIF11 inhibitor	0
NCGC00378640-02	Alvimopan	Mu-Type Opioid Receptor antagonist	0
NCGC00185757-01	Diethylmethyl(2-3-methyl-2-phenylvaleryloxy)-ethyl)ammonium bromide	Muscarinic acetylcholine receptor M3 Antagonist	0
NCGC00178815-03	Tolmetin sodium dihydrate	Cyclooxygenase-2 Inhibitor	0
NCGC00263239-03	FMK	Ribosomal Protein S6 Kinase alpha-3 inhibitor	0
NCGC00253562-10	ABT-737	Apoptosis regulator Bcl-2 inhibitor	1
NCGC00347566-02		MAPK signaling pathway Inhibitor	1
NCGC00095136-09	Olmesartan medoxomil		0
NCGC00162116-04	Cephapirin sodium	Bacterial penicillin-binding protein Inhibitor	0
NCGC00510111-01	VLX-600	Autophagy inducer	1
NCGC00386210-03	Golvatinib	Vascular Endothelial Growth Factor Receptor 2 (VEGFR-2) inhibitor	0
NCGC00024452-04	Miglustat	Maltase-glucoamylase Inhibitor	0
NCGC00091612-09	Triacetin		0
NCGC00091019-19	Dexamethasone	Glucocorticoid Receptor agonist	0

NCGC00249932-01	Emorfazone		0
NCGC00095911-06	Quetiapine	Serotonin 2a (5-HT2a) receptor Antagonist	0
NCGC00346450-01	Triciribine phosphate	AKT serine/threonine kinase inhibitor	0
NCGC00263839-02	Pentisomicin	Staphylococcus aureus Inhibitor	0
NCGC00253658-01	Dapoxetine	Serotonin Transporter (SERT) Inhibitor	0
NCGC00013822-04	NSC-75503	Sonic Hedgehog protein inhibitor	0
NCGC00385743-01		<MOA Unknown> | Class: Saponin | Genus: N/A | Family: N/A | Species: N/A	1
NCGC00345792-02	AN-2728	Phosphodiesterase IIII (PDE4) Inhibitor	0
NCGC00167503-02	Aclarubicin	DNA Topoisomerase II inhibitor	0
NCGC00263218-08	Teriflunomide	Dihydroorotate Dehydrogenase inhibitor	1
NCGC00094736-05	Nitromide	Eimeria tenella Inhibitor	0
NCGC00182070-03	Ioxilan		0
NCGC00093362-05	Betahistine mesylate	Histamine H3 receptor Antagonist	0
NCGC00167542-01	Tiamulin	Bacterial 70S ribosome Inhibitor	0
NCGC00015100-15	Altretamine	DNA Alkylating Drug	0
NCGC00166241-02	2,6-DIIODO-4-NITROPHENOL		0
NCGC00389390-01	Abiraterone acetate	Cytochrome P450 17A1 Inhibitor	1
NCGC00390770-02	Arglabin	NACHT, LRR and PYD domains-containing protein 3 Inflammasome inhibitor	0
NCGC00016421-12	CYCLIZINE	Histamine H1 receptor Antagonist	0
NCGC00346551-03	Pomalidomide	Cereblon inhibitor	0
NCGC00025100-17	LFM-A13	Bruton's Tyrosine Kinase (BTK) inhibitor	0
NCGC00263126-04	Cutamesine hydrochloride	sigma1 Receptor Agonist	0
NCGC00263194-02	SB-216763	Glycogen Synthase Kinase 3 (GSK-3) inhibitor	0
NCGC00015358-18	Disopyramide	Na channel blocker	0
NCGC00386638-01	2-(6-((E)-2-((3R,3aS,4S,5R,6S,7aR)-3,5,6-trimethyl-1-oxooctahydroisobenzofuran-4-yl)vinyl)pyridin-3-yl)Benzonitrile	Proteinase-activated receptor 1 Antagonist	1
NCGC00091032-09	Cyproterone acetate	Androgen Receptor antagonist	0
NCGC00178697-07	Irinotecan	DNA Topoisomerase I inhibitor	0
NCGC00167510-09	Doripenem Hydrate		0
NCGC00263546-03	Moexipril HCl	Angiotensin-converting enzyme Inhibitor	0
NCGC00346724-02	Varlitinib tosylate	Epidermal Growth Factor Receptor inhibitor	0
NCGC00022787-04	Oxacillin sodium	beta-lactam antibiotic	0
NCGC00370731-05	OXA-01	mTOR Complex 1 (mTORC1) inhibitor	0
NCGC00165768-05	BML-277	Checkpoint kinase 2 inhibitor	0
NCGC00253600-01	Iothalamate sodium		0
NCGC00351483-01	XL-388	mTOR Complex 1 (mTORC1) inhibitor	0
NCGC00186464-03	Aliskiren hemifumarate	Renin inhibitor	0
NCGC00241097-06	Tandutinib	Platelet-derived growth factor receptor alpha inhibitor	0
NCGC00018098-07	Iodoquinol		1

NCGC00021274-04	ESTRADIOL BENZOATE		0
NCGC00346931-02	NVP-TAE226	Insulin-like growth factor 1 receptor inhibitor	1
NCGC00345848-02	AMG-1	Hepatocyte Growth Factor Receptor inhibitor	0
NCGC00092310-14	Amisulpride	Dopamine Receptor D2 antagonist	0
NCGC00024643-10	Ifenprodil	Kir3.1/Kir3.4 Inhibitor	0
NCGC00378591-01	HG-9-91-01	Serine/threonine-protein kinase SIK2 inhibitor	1
NCGC00159430-04	Hexobarbital	GABA-A receptor; benzodiazepine site Activator	0
NCGC00522008-01	BI09	Serine/threonine-protein kinase/endoribonuclease IRE1 inhibitor	0
NCGC00485903-01	2,4-Pyrimidinediamine with linker		1
NCGC00248196-04	Tempol	Antioxidant	0
NCGC00274058-01	Naloxone	Mu opioid receptor Inverse Agonist	0
NCGC00378567-02	MK-8033	Hepatocyte Growth Factor Receptor inhibitor	0
NCGC00378916-02	MK-5172		0
NCGC00351599-03	Abemaciclib	Cyclin-Dependent Kinase 4 inhibitor	1
NCGC00345829-03	PD-173955	Tyrosine-protein kinase CSK Inhibitior	0
NCGC00263961-02	Ceftriaxone disodium trihydrate	Solute carrier family 22 member 8 Inhibitor	0
NCGC00179658-04	Acenocoumarol	Vitamin k epoxide reductase complex subunit 1 isoform 1 Inhibitor	0
NCGC00096028-06	PIPLARTINE		1
NCGC00016407-08	Sulfamethizole	Anti-bacterial	0
NCGC00496839-01	ON-123300	Proto-oncogene tyrosine-protein kinase receptor Ret inhibitor	0
NCGC00016446-17	Metronidazole	Antibiotic	0
NCGC00182849-01	Epristeride	3-oxo-5-alpha-steroid 4-dehydrogenase 2 Inhibitor	0
NCGC00379087-02	Tipranavir		0
NCGC00250386-10	BX-795	Serine/threonine-protein kinase ULK1 inhibitor	0
NCGC00263639-15	Tacedinaline	Histone deacetylase 1, class I inhibitor	1
NCGC00164529-04	1-Stearoyl-rac-glycerol	Phospholipase A2 group IIA Inhibitor	0
NCGC00166266-03	1,3-Bis(4-carboxyphenoxy)propane	0	
NCGC00494684-01	Basmisanil	Gamma-aminobutyric acid receptor subunit alpha-5 Inverse agonist	0
NCGC00016026-10	Terazosin hydrochloride	Alpha1-adrenoceptor Antagonists	0
NCGC00532503-01	BUNITROLOL	Beta-2 adrenergic receptor Antagonist	0
NCGC00165772-06	C75	Carnitine O-Palmitoyltransferase 1 activator	0
NCGC00183273-04	Chloroprocaine HCl	"Sodium Channel Alpha Subunits; Brain (types I, Ii, Iii) Inhibitor"	0
NCGC00181002-10	Cinacalcet hydrochloride	Calcium-Sensing Receptor agonist	0
NCGC00345812-02	Ingenol mebutate	Protein Kinase C delta type ligand	0
NCGC00159508-05	Rose Bengal disodium	Free Radical Stimulator	0

NCGC00484800-01	FX-9847	Dual-Specificity Tyrosine-(Y)-Phosphorylation Regulated Kinase 1A inhibitor	1
NCGC00346636-03	WYE-687	mTOR Complex 1 (mTORC1) inhibitor	1
NCGC00167443-02	Afloqualone		0
NCGC00182993-01	Betiatide		0
NCGC00164547-18	Zafirlukast	Cysteinyl leukotriene receptor 1 antagonist	0
NCGC00015277-17	Cinoxacin	Topoisomerase IV Inhibitor	0
NCGC00241111-12	VX-745	Mitogen-Activated Protein Kinase p38 inhibitor	0
NCGC00013058-02	Malachite green oxalate		1
NCGC00378713-01	Nirogacestat	gamma-Secretase inhibitor	0
NCGC00015755-10	Nimustine	DNA Crosslinking Agent	0
NCGC00091099-01	Dibromomannitol	DNA Crosslinking Agent	0
NCGC00017159-23	Curcumin	G1/S-specific cyclin-D1 Expression inhibitor	0
NCGC00390283-02	Vesatolimod	Toll-Like Receptor 7 agonist	0
NCGC00384168-11	SAG	Smoothened Receptor agonist	0
NCGC00095348-11	Diindolylmethane	Androgen Receptor Antagonist	0
NCGC00262642-10	WP-1066	Signal Transducer and activator of Transcription 3 inhibitor	0
NCGC00346685-03	KW-2478	Heat Shock Protein 90 (Hsp90) inhibitor	0
NCGC00015840-09	Phloretin	Vascular cell adhesion protein 1 Expression inhibitor	0
NCGC00025323-08	Ubenimex	Aminopeptidase N inhibitor	0
NCGC00357427-09	Pyridostatin	G-quadraplex binding ligand	0
NCGC00092386-26	Sertraline hydrochloride	Sodium-dependent Serotonin Transporter inhibitor	0
NCGC00381753-02	Pitavastatin Calcium	HMG-CoA Reductase Inhibitor	1
NCGC00015014-11	Acetohexamide	"Sulfonylurea receptor 1, Kir6.2 Inhibitor"	0
NCGC00025345-02	Atomoxetine hydrochloride	Serotonin transporter Inhibitor	0
NCGC00095099-11	Sildenafil citrate	Phosphodiesterase 5A inhibitor	0
NCGC00346700-13	A-803467	Nav1.8 (SNS/PN3) Sodium Channel Blocker	0
NCGC00167550-08	Narasin	Nf-kappa B Signaling Pathway Inhibitor	1
NCGC00015610-17	Leflunomide	Dihydroorotate Dehydrogenase inhibitor	1
NCGC00022735-16	Methylprednisolone	Glucocorticoid Receptor agonist	0
NCGC00274068-01	Itraconazole	cytochrome Inhibitor	1
NCGC00249900-01	Glyceryl 1-(2-aminobenzoate)		0
NCGC00167969-02	Rubitecan		1
NCGC00024868-09	Bisoprolol fumarate	beta1-Adrenoceptor Antagonists	0
NCGC00346824-01	3-Bromopyruvate	Hexokinase 2 inhibitor	0
NCGC00485486-02	Nastorazepide	Cholecystokinin B Receptor antagonist	0
NCGC00346965-01	HMSL10087	FLT4	1
NCGC00179669-04	Norethindrone		0
NCGC00091695-03	Triethylenetetramine	Copper Chelating Agent	0

NCGC00384864-01		<MOA Unknown> | Class: Saponin | Genus: N/A | Family: N/A | Species: N/A	1
NCGC00386699-01	Fluphenazine decanoate dihydrochloride	0	
NCGC00346530-09	Pimasertib	Dual Specificity Mitogen-Activated Protein Kinase Kinase 1 inhibitor	0
NCGC00016506-07	Naphazoline hydrochloride	Alpha-1b adrenergic receptor Agonist	0
NCGC00531792-01	PYRETHRIN II	Plasmodium falciparum Inhibitor	0
NCGC00165774-05	Cloprostenol (sodium salt)		0
NCGC00179504-05	Etoposide	DNA Topoisomerase II inhibitor	0
NCGC00319022-01	Octotiamine		0
NCGC00390270-02	Amrubicin	DNA Topoisomerase II inhibitor	0
NCGC00015082-09	AMINOGUANIDINE HYDROCHLORIDE	Nitric Oxide Synthase, inducible inhibitor	0
NCGC00025179-12	Mifepristone	Progesterone Receptor antagonist	0
NCGC00164633-02	Nisoldipine	Ca2+ channel blocker (dihydropyridine)	1
NCGC00094719-08	Methenamine		0
NCGC00015316-05	Eflornithine Hydrochloride	Ornithine Decarboxylase inhibitor	0
NCGC00481588-01	ACY-241	Histone deacetylase 6, class IIB inhibitor	0
NCGC00091285-03	Tricaprylin		0
NCGC00015659-14	Mexiletene hydrochloride		0
NCGC00024504-09	Kainic acid	Glutamate receptor ionotropic kainate 5 Agonist	0
NCGC00386747-06	TIC-10	Caseinolytic mitochondrial matrix peptidase inhibitor	0
NCGC00016324-09	Sulfamethoxypyridazine		0
NCGC00420882-01	LY-341495	Glutamate receptor metabotropic 3 antagonist	0
NCGC00183829-01	Minodronic acid	Geranylgeranyl pyrophosphate synthetase Inhibitor	0
NCGC00510356-01	AX-024	T Cell Receptor/Cytoplasmic Protein NCK1 Interaction inhibitor	0
NCGC00025349-05	Apomorphine	D2-like dopamine receptor Agonist	0
NCGC00015876-11	Ranitidine	Histamine H2 receptor Antagonist	0
NCGC00378682-03	Pasireotide (ditrifluoroacetate)	Somatostatin receptor 5 Agonist	0
NCGC00182031-05	Etizolam	GABA-A receptor Agonist	0
NCGC00488967-01	Senexin-B	Cyclin-Dependent Kinase 19 inhibitor	1
NCGC00346482-01	Mesna disulfide	chemoprotective agent	0
NCGC00263157-02	BMS-777607	Tyrosine-protein kinase Mer inhibitor	0
NCGC00345828-07	Pyridone 6	Tyrosine-protein kinase JAK1 inhibitor	0
NCGC00015251-13	Clotrimazole	Anti-fungal	0
NCGC00346600-02	Dioscin	ROS inducer	1
NCGC00182988-01	Acreozast		0
NCGC00485024-02	PF-06454589	Leucine-Rich Repeat Kinase 2 inhibitor	0
NCGC00160614-01	Glucuronamide		0
NCGC00247716-01	(+)-Pentazocine succinate		0
NCGC00522023-01	Paliperidone Palmitate		0

NCGC00161834-01	Budesonide	Glucocorticoid steroid	0
NCGC00509944-01	Asciminib	Bcr-Abl Kinase inhibitor	1
NCGC00091544-18	Estradiol	Estrogen Receptor agonist	0
NCGC00346548-07	KRN-633	Vascular Endothelial Growth Factor Receptor 2 (VEGFR-2) inhibitor	0
NCGC00022043-06	Deoxycorticosterone acetate	Mineralocorticoid receptor Agonist	0
NCGC00164601-02	Liranaftate	Fungal Squalene Monooxygenase Inhibitor	0
NCGC00489873-03	CCT-251921	Cyclin-Dependent Kinase 8 inhibitor	0
NCGC00015237-07	Carbachol	Acetylcholinesterase Substrate	0
NCGC00253608-01	Chlorexolone		0
NCGC00253740-06	Lacosamide	NMDA Glycine-Site Receptor antagonist	0
NCGC00346638-03	TWS-119	Glycogen Synthase Kinase 3 (GSK-3) inhibitor	1
NCGC00183600-04	PARAMETHADIONE	Voltage-gated T-type calcium channel Blocker	0
NCGC00014810-01	Pirbuterol hydrochloride	Beta-2 adrenergic receptor Agonist	0
NCGC00347904-03	AZ-11645373	P2X Purinoceptor 7 antagonist	1
NCGC00249922-01	Estriol benzoate diacetate		0
NCGC00263893-02	Lactitol	Maltase-glucoamylase Inhibitor	0
NCGC00510480-01	YKL-05-099	Serine/threonine-protein kinase SIK2 inhibitor	1
NCGC00021226-04	Dexamethasone 21-phosphate disodium salt	Glucocorticoid receptor Agonist	0
NCGC00263210-04	SANT-2	Smoothened Receptor antagonist	0
NCGC00183039-03	Nystatin	Anti-fungal	0
NCGC00378854-02	Anamorelin	Growth Hormone Secretagogue Receptor Type 1 agonist	0
NCGC00182074-03	Nicomolol	Cholesterol biosynthesis Modulator	0
NCGC00165846-04	Nefazodone hydrochloride	Serotonin transporter Antagonist	0
NCGC00095164-04	Protionamide	tuberculosis treatment	0
NCGC00346713-02	AZD-4547	Fibroblast Growth Factor Receptor 1 inhibitor	0
NCGC00179370-05	Methotrimeprazine maleat salt	Serotonin 2 (5-HT2) receptor Antagonist	0
NCGC00510688-01	LGH-447	Serine/threonine-protein kinase pim-1 inhibitor	0
NCGC00378990-07	Ledipasvir (GS5885)	Nonstructural protein 5A Inhibitor	0
NCGC00015678-10	Moxisylyte hydrochloride	Adrenergic receptor alpha-1 Antagonist	0
NCGC00379210-02	LMK235	Histone deacetylase 6 Inhibitor	1
NCGC00016994-20	Camptothecin	DNA Topoisomerase I inhibitor	1
NCGC00015520-11	Hydroxyurea	Ribonucleoside-Diphosphate Reductase inhibitor	0
NCGC00166323-16	Trifluorothymidine	Thymidylate Synthase inhibitor	0
NCGC00188689-09	Asenapine maleate	Dopamine D1/D2 Antagonist	1
NCGC00095191-14	Oseltamivir (phosphate)		0
NCGC00161418-13	JTC-801	Nociceptin Receptor antagonist	0
NCGC00274273-01		Mu opioid receptor Partial Agonist	0

NCGC00387145-01	HA-130	Ectonucleotide Pyrophosphatase/Phosphodiesterase Family Member 2 inhibitor	1
NCGC00246968-07	Olmesartan	Angiotensin AT1 Receptor antagonist	0
NCGC00183654-01	Etidocaine	Sodium channel protein type II alpha subunit Blocker	0
NCGC00183271-01	Bucillamine		0
NCGC00346976-02	ML-323	Ubiquitin Carboxyl-Terminal Hydrolase 2 inhibitor	0
NCGC00344588-04	I-BET151	Bromodomain-Containing Protein 2 (Brd2) inhibitor	1
NCGC00094851-05	Iodamide		0
NCGC00483046-01	SCE 2174		0
NCGC00185071-01	Calcium folinate	Antineoplastic Enhancing Agents	0
NCGC00386312-01	CNX-774	Bruton's Tyrosine Kinase (BTK) inhibitor	0
NCGC00024359-10	Penicillamine	Metal Chelating Agent	0
NCGC00160511-01	Desoxycorticosterone Pivalate		0
NCGC00015854-10	Propantheline bromide	Muscarinic acetylcholine receptor M4 Antagonist	0
NCGC00178214-04	BENZALKONIUM CHLORIDE		1
NCGC00347945-09	ID-8	Dual-Specificity Tyrosine-(Y)-Phosphorylation Regulated Kinase 1A inhibitor	0
NCGC00483049-02	Gamithromycin		0
NCGC00166309-02	Altrenogest		0
NCGC00184994-02	Cidofovir	DNA Polymerase Inhibitor	0
NCGC00015409-11	Edrophonium chloride	Butyrylcholinesterase Inhibitor	0
NCGC00346458-02	MLN 2480	RAF Kinase inhibitor	0
NCGC00249414-01	Fonazine	Serotonin (5-HT) receptor Antagonist	0
NCGC00249929-01	Neticonazole	ergosterol biosynthetic process Inhibitor	0
NCGC00021623-05	Nadolol	Beta-2 adrenergic receptor Antagonist	0
NCGC00022248-04	Hydrocortisone 21-hemisuccinate sodium salt	0	
NCGC00164789-04	Fulvestrant	Estrogen Receptor Antagonist	0
NCGC00485920-01	Tofogliflozin (hydrate)	Sodium/glucose cotransporter 2 Inhibitor	0
NCGC00023648-04	Desoximetasone	Glucocorticoid receptor Agonist	0
NCGC00163451-04	Go-6976	Checkpoint kinase 1 inhibitor	0
NCGC00344578-01	Flavin adenine dinucleotide disodium salt hydrate	oxidative phosphorylation Modulator	0
NCGC00387803-01	Atosiban	Oxytocin receptor Antagonist	0
NCGC00378999-05	SB-1317	Cyclin-Dependent Kinase 9 inhibitor	0
NCGC00386882-01	ML264	Krueppel-like factor 5 Expression inhibitor	0
NCGC00386449-03	MLN-7243	NEDD8-Activating Enzyme E1 inhibitor	1
NCGC00182079-09	TRANDOLAPRIL		0
NCGC00016551-04	Meprylcaine hydrochloride	"Sodium channel alpha subunits; brain (Types I, II, III) Inhibitor"	0
NCGC00167301-02	Triptorelin	Gonadotropin-releasing hormone receptor Agonist	0

NCGC00160540-03	Ethiazide		0
NCGC00183597-01	Betamethasone butyrate propionate	0	
NCGC00346722-06	ADX-47273	Glutamate receptor metabotropic 5 modulator	0
NCGC00510028-01	Amezinium methylsulfate	Amine oxidase [flavin-containing] B Inhibitor	0
NCGC00253760-02	Albendazole oxide	antiparasitic agent	0
NCGC00507844-01	BQ-123	Endothelin-1 Receptor antagonist	0
NCGC00015519-11	Dopamine hydrochloride	Dopamine Receptor D2 agonist	0
NCGC00095056-04	Retinyl palmitate		0
NCGC00094419-03	TPCK	Serine Protease inhibitor	0
NCGC00016360-07	Monobenzone	Tyrosinase inhibitor	0
NCGC00018277-06	Mebeverine hydrochloride		0
NCGC00160469-01	Gamolenic acid	prostanoid metabolic process Interacts	0
NCGC00090970-03	Maleic acid	"NADP-dependent malic enzyme, mitochondrial Inhibitor"	0
NCGC00015496-08	N,N,N,N',N',N'-Hexamethylhexane-1,6-diaminium	0	
NCGC00263235-01	ACC1 BMS	Acetyl-CoA Carboxylase inhibitor	0
NCGC00183658-01	Quadrisol	Prostaglandin G/H synthase 2 Inhibitor	0
NCGC00346632-02	R-547	Cyclin-Dependent Kinase 4 inhibitor	1
NCGC00250382-07	Sonidegib	Smoothened Receptor antagonist	0
NCGC00167477-01	Fidarestat	Aldose reductase Inhibitor	0
NCGC00168085-26	Vorinostat (SAHA)	Histone deacetylase 1, class I inhibitor	1
NCGC00390569-01	Netupitant		0
NCGC00095312-04	Deracoxib	Prostaglandin G/H synthase 2 Inhibitor	0
NCGC00169779-03		<MOA Unknown> | Class: Iridoid | Genus: Plumeria | Family: Apocynaceae | Species: rubra	1
NCGC00386329-06			1
NCGC00181334-04	DIBUNATE SODIUM		0
NCGC00274065-01	Desonide	Glucocorticoid Receptor agonist	0
NCGC00161336-01	Potassium ricinoleate	Prostanoid EP3 receptor Binding Agent	0
NCGC00263121-03	MG-149	Histone Acetyltransferase KAT5 inhibitor	0
NCGC00344626-14	GSK-J4	Lysine-Specific Demethylase 6A inhibitor	0
NCGC00346966-01	HMSL10088	Receptor-interacting serine/threonine-protein kinase 1 (RIPK1)	0
NCGC00263955-02	TYLOSIN TARTRATE		0
NCGC00263208-09	TW-37	Apoptosis regulator Bcl-2 inhibitor	1
NCGC00166414-05	Pemetrexed	Thymidylate Synthase inhibitor	0
NCGC00165979-06	CINNAMIC ACID		0
NCGC00017223-05	Ursolic acid	11-beta-Hydroxysteroid Dehydrogenase Type 1 inhibitor	0
NCGC00160383-02	Lenperone Hydrochloride	Serotonin 2 (5-HT2) receptor Antagonist	0
NCGC00370783-06	GSK-2578215A	Leucine-Rich Repeat Kinase 2 inhibitor	0
NCGC00371443-05	KDM5-C70	Lysine-Specific Demethylase 5B inhibitor	0

NCGC00249911-01	Normethadone	Mu-type opioid receptor Agonist	0
NCGC00250389-02	CDIBA	Cytosolic Phospholipase A2 inhibitor	0
NCGC00390715-04	UNBS-5162	pan-CXCL expression inhibitor	0
NCGC00380159-01		<MOA Unknown> | Class: Steroide | Genus: N/A | Family: N/A | Species: N/A	1
NCGC00263160-06	Brivanib	Vascular Endothelial Growth Factor Receptor 2 (VEGFR-2) inhibitor	0
NCGC00091351-01	m-Dinitrobenzene		0
NCGC00164553-07	Clofarabine	DNA Polymerase inhibitor	0
NCGC00479074-01	Acalabrutinib	Bruton's Tyrosine Kinase (BTK) inhibitor	0
NCGC00095264-04	Chloroxine	Antibacterial	1
NCGC00015148-12	DL-Buthionine-(S,R)-sulfoximine	Glutamate-Cysteine Ligase catalytic subunit inhibitor	0
NCGC00094652-06	DIGITOXIN		0
NCGC00159513-03	Cefprozil	Cephalosporin antibiotic	0
NCGC00249895-01	Bornelone		0
NCGC00522021-01	Dexamethasone 21-Palmitate	Glucocorticoid receptor Agonist	0
NCGC00532495-01	Sizofilan		1
NCGC00016600-08	Clofazimine	DNA Binding Agent	0
NCGC00015609-15	Lonidamine	Mitochondrial pyruvate carrier 1 (MPC1) inhibitor	0
NCGC00167533-04	Vardenafil	Phosphodiesterase V (PDE5) Inhibitor	0
NCGC00166245-04	Proflavin hemisulfate	RNA Binding Agent	0
NCGC00386298-01	Rifapentine	DNA-Directed RNA Polymerase Inhibitor	0
NCGC00024846-08	Guanabenz	Adrenergic receptor alpha-2 Agonist	0
NCGC00386241-01	Avanafil	Phosphodiesterase 5A Inhibitor	0
NCGC00092364-11	PNU-282987	Neuronal acetylcholine receptor subunit alpha-7 agonist	0
NCGC00091666-04	1,8-Cineol	tumor necrosis factor production Inhibitor	0
NCGC00386761-02	Mps1-IN-2	Dual specificity protein kinase TTK Inhibitor	1
NCGC00015191-21	Chlormezanone	Gamma-aminobutyric acid receptor subunit alpha-1 modulator	0
NCGC00481577-01	TP-0903	AXL Kinase inhibitor	1
NCGC00018271-07	Homochlorcyclizine		0
NCGC00346808-03	GNF-2	Bcr-Abl Kinase inhibitor	0
NCGC00159492-03	Perazine	Cytochrome P450 1A2 Inhibitor	0
NCGC00091208-11	HEXYLRESORCINOL	Tyrosinase Inhibitor	0
NCGC00253611-01	Isoaminile		0
NCGC00179384-10	Chlorcyclizine	Hepatitis C virus Inhibitor	0
NCGC00183837-01	Monatepil	Calcium Channel Blocker	0
NCGC00179306-04	Zuclopenthixol (-)-10-Camphorsulfonic Acid Salt	Serotonin 2 (5-HT2) receptor Antagonist	0
NCGC00346614-07	PIK-294	Phosphatidylinositol 3-Kinase delta isoform inhibitor	0
NCGC00159418-05	Tegafur	Thymidylate Synthase inhibitor	0

NCGC00510699-01	GSK9311	Bromodomain and PHD finger containing 1 inhibitor	0
NCGC00183105-01	Lubiprostone	Chloride Channel Protein 2 activator	0
NCGC00183870-01	Ethoheptazine hydrochloride	Opioid receptors; mu/kappa/delta Modulator	0
NCGC00160485-01	Diamthazole		0
NCGC00378896-04	MP-513 Tenelia Tenglyn Teneligliptin hydrobromide hydrate (Prop INNM)	0	
NCGC00016640-06	Todralazine hydrochloride	histone acetylation Inhibitor	0
NCGC00165889-13	SB-505124	TGF-beta receptor type-1 inhibitor	0
NCGC00346943-03	GSK-923295	Centromere Associated Protein E inhibitor	1
NCGC00013895-08	Benzbromarone	Xanthine dehydrogenase Inhibitor	0
NCGC00506889-01	Sugammadex		0
NCGC00096117-01	5-Iodotubercidin		1
NCGC00015256-29	Chloroquine	Anti-malarial	0
NCGC00024501-04	D-Glutamic acid	Metabotropic glutamate receptor 1 Agonist	0
NCGC00165791-04	Epinastine hydrochloride	Histamine H1 receptor Antagonist	0
NCGC00263564-04	Levosimendan	Troponin C potentiator	0
NCGC00381306-01		<MOA Unknown> | Class: N/A | Genus: N/A | Family: N/A | Species: N/A	1
NCGC00016960-07	Glimepiride	K(ATP) Channel activator	0
NCGC00091011-20	CHLORAMPHENICOL		0
NCGC00181165-01	Sodium decanehydroxamate		0
NCGC00421877-04	AG-120		0
NCGC00390744-01	Necrostatin 2	Receptor-interacting serine/threonine-protein kinase 1 inhibitor	0
NCGC00164631-19	Sunitinib malate	Proto-oncogene tyrosine-protein kinase receptor Ret inhibitor	1
NCGC00160478-02	Menadione sodium bisulfite	NQO1 protein Substrate	0
NCGC00015692-08	Methoxamine hydrochloride		0
NCGC00021666-04	Nalmefene	Kappa opioid receptor Partial Agonist	0
NCGC00241103-01	Masitinib	Fibroblast Growth Factor Receptor 3 inhibitor	0
NCGC00091146-08	Phenothiazine	Trichostrongylus colubriformis Inhibitor	0
NCGC00167574-03	Xaliproden hydrochloride	Serotonin 1a (5-HT1a) receptor Agonist	0
NCGC00379217-03	APY29	Serine/threonine-protein kinase/endoribonuclease IRE1 inhibitor	0
NCGC00022398-05	Fluocinonide	Glucocorticoid Receptor agonist	0
NCGC00345794-11	MLN-4924	NEDD8-Activating Enzyme E1 inhibitor	1
NCGC00247879-05	VER-82576	Heat Shock Protein 90 (Hsp90) inhibitor	1
NCGC00507869-01	Lanabecestat	Beta-Secretase 1 inhibitor	0
NCGC00015429-18	Felbamate	Gamma-aminobutyric acid receptor subunit alpha-1 modulator	0
NCGC00263222-02	CAY-10581	Indoleamine 2,3-dioxygenase 1 inhibitor	0
NCGC00016256-20	Pyrimethamine	Malarial Dihydrofolate Reductase inhibitor	1
NCGC00178803-03	HOMATROPINE METHYLBROMIDE	0	

NCGC00483926-01	Sitravatinib	Proto-oncogene tyrosine-protein kinase receptor Ret inhibitor	1
NCGC00013369-07	Pamoic acid disodium salt		0
NCGC00159477-05	Phenylacetic acid		0
NCGC00389470-01	Dirithromycin	Bacterial 70S ribosome Inhibitor	0
NCGC00179305-04	Lymecycline		0
NCGC00179333-03	Phensuximide		0
NCGC00189393-09	NCGC00189393	Bloom Helicase inhibitor	0
NCGC00182709-03	Landiolol hydrochloride	Beta-1 adrenergic receptor Antagonist	0
NCGC00016572-12	Sulfamonomethoxine		0
NCGC00179308-04	Fluticasone propionate	Glucocorticoid Receptor agonist	0
NCGC00386720-01	CP-346086	Apolipoprotein B Secretion inhibitor	0
NCGC00507871-01	BAY-1217389	Dual Specificity protein kinase TTK inhibitor	0
NCGC00510506-01	RG-7845 (GDC-0853)	Bruton's Tyrosine Kinase (BTK) inhibitor	0
NCGC00532294-01	Martinostat	Histone deacetylase 1, class I inhibitor	1
NCGC00016902-04	Etifenin		0
NCGC00262942-01	Etifelmine hydrochloride		0
NCGC00091345-04	Hexanedioic acid		0
NCGC00095152-09	Alfuzosin hydrochloride	alpha1-Adrenoceptor Antagonists	0
NCGC00181120-02	Acetophenazine Maleate	Sigma opioid receptor Antagonist	0
NCGC00179578-03	Mometasone furoate		0
NCGC00263198-04	Lenvatinib	Proto-oncogene tyrosine-protein kinase receptor Ret inhibitor	0
NCGC00181755-01	Delapril hydrochloride		0
NCGC00090770-09	5-Bromo-2'-deoxyuridine	base conversion or substitution editing Activator	0
NCGC00164624-02	Adefovir dipivoxil	DNA Polymerase Inhibitor	0
NCGC00249393-08	Apatinib	Proto-oncogene tyrosine-protein kinase receptor Ret inhibitor	0
NCGC00181031-03	Hydroxycitronellal	Transient receptor potential cation channel subfamily M member 8 Agonist	0
NCGC00346532-04	VX-222		0
NCGC00015649-08	Moxonidine hydrochloride hydrate	Imidazoline I1 (Nischarin) Receptor agonist	0
NCGC00384513-01		Sodium/potassium-transporting ATPase Inhibitor	1
NCGC00370984-01	M-475271	Proto-oncogene tyrosine-protein kinase Src inhibitor	1
NCGC00186460-03	Bardoxolone methyl	Nuclear Factor Erythroid 2-Related Factor 2 activator	1
NCGC00389459-01	Cymeval	DNA Polymerase Inhibitor	0
NCGC00378941-04	Cinaciguat	Soluble Guanylate Cyclase (sGC) activator	0
NCGC00242227-11	TCS-JNK-5a	Mitogen-Activated Protein Kinase 9 inhibitor	0
NCGC00160163-01	Diosgenin	Cyclooxygenase up-regulator	0
NCGC00386008-01		<MOA Unknown> | Class: Steroid | Genus: N/A | Family: N/A | Species: N/A	1

NCGC00510463-03	STK16-IN-1	Phosphatidylinositol 3-Kinase alpha isoform inhibitor	0
NCGC00178768-03	POLYMYXIN B SULFATE	Peptide Antibiotic	0
NCGC00160621-05	DEXTROSE		0
NCGC00181096-01	Chlophedianol		0
NCGC00090705-24	Zalcitabine	Reverse Transcriptase Inhibitor	0
NCGC00347948-15	IWP-2	Protein Serine O-Palmitoleoyltransferase Porcupine inhibitor	0
NCGC00166215-05	2-Phenylethanol		0
NCGC00165862-04	Pamidronate Disodium	Farnesyl diphosphate synthase Inhibitor	0
NCGC00015267-11	Citalopram hydrobromide	5-HT Reuptake Inhibitor	0
NCGC00389197-01	HSP-990	Heat Shock Protein 90 (Hsp90) Inhibitor	0
NCGC00247785-11		Perinuclear compartment (PNC) assembly inhibitor	0
NCGC00013683-06	Chlorprothixene	Serotonin (5-HT) receptor Antagonist	0
NCGC00091888-09	3,3,5-Trimethylcyclohexyl salicylate	0	
NCGC00510695-01	Belizatinib	ALK Tyrosine Kinase Receptor inhibitor	0
NCGC00091737-05	Sorbic acid		0
NCGC00346650-02	CH-5132799	Phosphatidylinositol 3-Kinase beta isoform inhibitor	1
NCGC00016270-09	Levodopa	Dopamine Receptor D1 agonist	0
NCGC00522551-01	Monepantel		0
NCGC00183108-01	Propamidine		0
NCGC00183830-01	Lemildipine	Voltage-gated calcium channel Blocker	0
NCGC00166217-02	1-Bromoheptadecafluorooctane		0
NCGC00017369-07	Biochanin A	3-Oxo-5-alpha-steroid 4-dehydrogenase 1 inhibitor	0
NCGC00372509-03	BMS-4	LIM domain kinase 2 inhibitor	0
NCGC00370842-02	PF-670462	Casein Kinase 1 delta inhibitor	0
NCGC00015448-06	Salirasib	mTOR Complex 1 (mTORC1) inhibitor	0
NCGC00024563-11	Azasetron hydrochloride	5-HT3 Receptor Antagonist	0
NCGC00164431-01	Terpineol	Acetylcholinesterase Inhibitor	0
NCGC00387872-01	VS-4718	Focal Adhesion Kinase inhibitor	0
NCGC00371104-01	Cevimeline hydrochloride		0
NCGC00094381-09	Semaxanib	Vascular Endothelial Growth Factor Receptor 2 (VEGFR-2) inhibitor	0
NCGC00167536-03	Tosufloxacin toluenesulfonic acid	Topoisomerase IV Inhibitor	0
NCGC00386213-04	Rupatadine (Fumarate)	Platelet activating factor receptor Antagonist	0
NCGC00091865-02	Benzyl alcohol		0
NCGC00522027-01	Predocol		0
NCGC00183096-01	Cefcapene pivoxil hydrochloride	Penicillin-binding protein Inhibitor	0
NCGC00263538-01	Trovafloxacin mesylate	Streptococcus pneumoniae Inhibitor	0
NCGC00250410-11	GSK-650394	Serine/threonine-protein kinase Sgk1 inhibitor	0
NCGC00263103-09	Pelitinib	Epidermal Growth Factor Receptor inhibitor	1

NCGC00017253-03	Ergobasine	Serotonin (5-HT) receptor Partial Agonist	0
NCGC00263172-11	DCC-2036	Bcr-Abl Kinase inhibitor	1
NCGC00096014-03	Salsalate	prostaglandin biosynthetic process Inhibitor	0
NCGC00166015-02	C.I. Basic Violet 14		1
NCGC00250390-10	ISOX	Histone deacetylase 6, class IIB inhibitor	1
NCGC00378974-02	DCC-2618	Mast/stem cell Growth Factor Receptor Kit inhibitor	0
NCGC00346835-01	2-Iminobiotin	Nitric Oxide Synthase, inducible inhibitor	0
NCGC00262940-01	Iodoxamic Acid		0
NCGC00016374-05	Carbarsone		0
NCGC00159349-15	Levonorgestrel	Progesterone receptor Agonist	0
NCGC00090957-05	Octanoic acid	Solute carrier family 22 member 8 Substrate	0
NCGC00263120-06	Enzalutamide	Androgen Receptor antagonist	0
NCGC00178024-05	Metaraminol bitartrate	Adrenergic receptor alpha-1 Agonist	0
NCGC00483929-01	KI-696	Nuclear Factor Erythroid 2-Related Factor 2 inhibitor	0
NCGC00263598-06	Chidamide	Histone deacetylase 11, class IV inhibitor	0
NCGC00686671-01	Brincidofovir		0
NCGC00346304-02	L-779450	Serine/threonine-protein kinase B-raf inhibitor	0
NCGC00090814-07	4-Chlorophenol	macrophage activation involved in immune response Inhibitor	0
NCGC00356803-11	EPZ004777	Histone-lysine N-methyltransferase, H3 Lysine-79 Specific inhibitor	0
NCGC00021761-05	Flumethasone	Glucocorticoid receptor Agonist	0
NCGC00379065-03	Riociguat	Soluble Guanylate Cyclase (sGC) activator	0
NCGC00249901-01	Thiocyanoacetic acid sodium salt		0
NCGC00090714-01	Sodium xylenesulfonate		0
NCGC00018186-07	Procyclidine hydrochloride	Muscarinic acetylcholine receptor M4 Antagonist	0
NCGC00346951-01	Fascaplysin	Cyclin-Dependent Kinase 4 inhibitor	1
NCGC00386547-01	Difluprednate	Glucocorticoid receptor Agonist	0
NCGC00242476-03	GSK-4112	Nuclear Receptor Subfamily 1 Group D Member 1 agonist	0
NCGC00532497-01	CEFPODOXIME		0
NCGC00370891-01	C 021 dihydrochloride	C-C chemokine receptor type 4 antagonist	1
NCGC00094792-17	Tioguanine	Hypoxanthine-guanine phosphoribosyltransferase (HGPRTase) inhibitor	1
NCGC00015766-16	Oxymetazoline hydrochloride	Alpha-1A Adrenergic Receptor agonist	0
NCGC00090764-04	2-Ethylhexyl 4-(dimethylamino)benzoate	0	
NCGC00164632-07	TIBOLONE	Estrogen Receptor agonist	0
NCGC00183104-02	Nizofenone		0
NCGC00159506-06	Cyclandelate	Voltage-gated calcium channel Blocker	0
NCGC00164631-05	Sunitinib malate	Proto-oncogene tyrosine-protein kinase receptor Ret inhibitor	1

NCGC00093351-06	Cefoxitin	Penicillin-binding protein 2B Inhibitor	0
NCGC00016936-04	Aztreonam	beta-lactam antibiotic	0
NCGC00386277-03	GDC-0152	IAP inhibitor	0
NCGC00095460-08	Hematoxylin	Polyphenol and histologic stain	0
NCGC00344624-17	PFI-1	Bromodomain-containing protein 4 Inhibitor	1
NCGC00182606-01	Physalien		0
NCGC00167534-02	Pazufloxacin	Topoisomerase IV Inhibitor	0
NCGC00167526-06	Camostat	Trypsin inhibitor	0
NCGC00166088-02	Decitabine	DNA Methyltransferase (DNMT) inhibitor	0
NCGC00177995-10	Sarafloxacin hydrochloride	Antibacterial	0
NCGC00167558-02	Fleroxacin	Quinoline Antibiotic	0
NCGC00090955-06	Dicyclohexylamine	spermidine biosynthetic process Inhibitor	0
NCGC00484799-01	OTS964	Lymphokine-activated killer T-cell-originated protein kinase inhibitor	1
NCGC00356592-02	Ergotamine tartrate		0
NCGC00017115-04	Thiethylperazine Malate		0
NCGC00390365-01	Delamanid		0
NCGC00347952-11	NSC-319726	Mutant p53 activator	1
NCGC00162409-08	Bafilomycin A1	Vacuolar H+ ATPase (V-ATPase) inhibitor	1
NCGC00345826-04	CC-401	Mitogen-Activated protein kinase 8 (JNK) inhibitor	0
NCGC00346659-04	PF-04691502	Phosphatidylinositol 3-Kinase alpha isoform inhibitor	1
NCGC00355875-04	GSK-2837808A	L-Lactate Dehydrogenase A chain inhibitor	0
NCGC00179034-15	Pentamidine isethionate	Protein Tyrosine Phosphatase Type IVA inhibitor	0
NCGC00510263-01	GS-9901	Phosphatidylinositol 3-Kinase delta isoform inhibitor	0
NCGC00093976-15	Idarubicin hydrochloride	DNA Topoisomerase II inhibitor	1
NCGC00274067-09	Eltrombopag olamine	Thrombopoietin Receptor agonist	1
NCGC00091050-09	Phenylmercuric acetate		0
NCGC00161831-08	Mefloquine (hydrochloride)	Anti-malarial	1
NCGC00159417-10	TRICLOSAN	Bacterial enoyl-[acyl-carrier-protein] reductase Binding Agent	0
NCGC00092361-06	Prostaglandin E2	Prostaglandin E2 Receptor EP2 subtype agonist	0
NCGC00378911-02	(S)-Tedizolid	Bacterial 70S ribosome Inhibitor	0
NCGC00189130-07	UNC-0631	Histone-lysine N-methyltransferase EHMT2 inhibitor	0
NCGC00160530-13	Toremifene citrate	Selective Estrogen Receptor modulator (SERM)	0
NCGC00167459-02	Doxapram hydrochloride	Potassium channel subfamily K member 9 Inhibitor	0
NCGC00346877-07	Batimastat	Matrix Metalloproteinase (MMP) inhibitor	0
NCGC00384249-01	CXC-195	NADPH Oxidase inhibitor	0
NCGC00017360-04	Aconitine		0
NCGC00016407-16	Sulfamethizole	Anti-bacterial	0

NCGC00160529-06	Tizanidine hydrochloride	Alpha-2 Adrenergic Receptor agonist	0
NCGC00159455-27	Gefitinib	Epidermal Growth Factor Receptor inhibitor	0
NCGC00021394-04	Anisotropine methylbromide	Muscarinic acetylcholine receptor M3 Antagonist	0
NCGC00159320-04	Triamcinolone diacetate		0
NCGC00165783-03	Vadimezan	Stimulator of Interferon Genes Protein agonist	0
NCGC00346627-08	CX-5461	DNA-Directed RNA Polymerase inhibitor	0
NCGC00163413-07	Lycorine hydrochloride		1
NCGC00521943-01	TAK-931	Cell Division Cycle 7-Related Protein Kinase inhibitor	0
NCGC00163431-06	CA-074 Me	Cathepsin B inhibitor	0
NCGC00390266-01	Nalfurafine hydrochloride	Kappa opioid receptor Agonist	0
NCGC00165869-22	Dorsomorphin	Bone morphogenetic protein receptor type-1A inhibitor	1
NCGC00094874-05	Esculin hydrate	tumor necrosis factor production Inhibitor	0
NCGC00017344-11	Fisetin	NAD-Dependent Protein Deacetylase Sirtuin-1 activator	0
NCGC00387136-01	TC-A 2317 hydrochloride		1
NCGC00371122-02	Betrixaban	Coagulation factor X Inhibitor	0
NCGC00181023-03	NOLATREXED HCL		0
NCGC00507938-02	EED-226	Polycomb protein EED inhibitor	0
NCGC00346557-02	Erteberel	Estrogen Receptor beta agonist	0
NCGC00485951-02	VBY-825	Cathepsin S inhibitor	0
NCGC00420704-02	AT-IAP	IAP inhibitor	0
NCGC00015767-10	Oxybutynin chloride	Muscarinic Antagonists	0
NCGC00023699-09	Cephalothin	Solute carrier family 22 member 7 Inhibitor	0
NCGC00185956-04	UNC-0224	Histone-lysine N-methyltransferase EHMT2 inhibitor	0
NCGC00094857-06	AMINACRINE		1
NCGC00021145-11	Loxapine succinate	Muscarinic acetylcholine receptor M1 Antagonist	0
NCGC00263447-01	alpha-Santonin		0
NCGC00506887-01	Sodium aurothiomalate(I)	Prostaglandin E synthase Inhibitor	0
NCGC00262995-02	ML-364	Ubiquitin Carboxyl-Terminal Hydrolase 2 inhibitor	1
NCGC00507892-01	VPS34 Inhibitor 1 (Compound 19, PIK-III analogue)	Phosphatidylinositol 3-Kinase Type 3 inhibitor	0
NCGC00507850-01	RO-9021	Tyrosine-protein kinase SYK inhibitor	0
NCGC00183838-01	Perisoxal citrate		0
NCGC00016267-06	Isocarboxazid	Monoamine oxidase Inhibitor	0
NCGC00263203-06	Danusertib	Aurora kinase A inhibitor	0
NCGC00378918-01	CDK9-IN-2	CDK9/cyclin T1 Inhibitor	1
NCGC00015897-08	Ranolazine	Sodium channel protein type 5 subunit alpha Blocker	0
NCGC00093366-05	(4-Aminophenyl)arsonic acid	Apoptosis Activator	0

NCGC00387092-01	PF 429242	cholesterol biosynthetic process Inhibitor	1
NCGC00509886-03	BMS-214662	Protein farnesyltransferase/geranylgeranyltransferase type-1 inhibitor	1
NCGC00015661-12	Molsidomine		0
NCGC00345783-02	17-hydroxy Wortmannin	Phosphatidylinositol 3-Kinase alpha isoform inhibitor	0
NCGC00017048-03	Levopropoxyphene napsylate	Kappa Opioid Receptor Antagonist	0
NCGC00390582-04	RGB-286638	Cyclin-Dependent Kinase 1 inhibitor	1
NCGC00485948-01	WIN 55,212-2 (Mesylate)	Cannabinoid CB2 receptor Agonist	1
NCGC00389662-01	Luteinizing hormone releasing hormone human acetate salt (LHRH)	Putative gonadotropin-releasing hormone II receptor Agonist	0
NCGC00025000-22	Ketoconazole	Anti-fungal	1
NCGC00183845-01	Pibutidine	Histamine H2 receptor Binding Agent	0
NCGC00025307-02	Demethylasterriquinone B1		1
NCGC00163742-05	d-LIMONENE	response to oxidative stress Modulator	0
NCGC00378793-02	NBI-74330	Chemokine CXCR3 Receptor antagonist	0
NCGC00016372-07	4-Hydroxy-3-nitrophenylarsonic acid	0	
NCGC00188864-02	Iloperidone	Dopamine D2 Antagonist	0
NCGC00386321-01	Triapine	Ribonucleoside-Diphosphate Reductase inhibitor	0
NCGC00095112-07	Avobenzone	Free Radical Scavenger	0
NCGC00347279-01	AIM-100	Activated CDC42 kinase 1 inhibitor	0
NCGC00509863-01	Naloxegol (oxalate)	Mu-type opioid receptor Antagonist	0
NCGC00023212-14	Stavudine	Reverse Transcriptase Inhibitor	0
NCGC00016618-04	Dimethisoquin hydrochloride	Neuronal acetylcholine receptor; alpha4/beta4 Inhibitor	0
NCGC00390811-01	Oxytocin	Oxytocin receptor Agonist	0
NCGC00510750-02	JPH203	Large neutral amino acid transporter small subunit 1 inhibitor	0
NCGC00166322-02	PROTIRELIN	Thyrotropin-releasing hormone receptor Agonist	0
NCGC00015997-10	Taurine	Antioxidant	0
NCGC00090766-07	Prednisone	Glucocorticoid Receptor agonist	0
NCGC00384199-05	4SC-202	Histone deacetylase 2, class I inhibitor	1
NCGC00163394-01	BML-265		1
NCGC00016323-09	Sulfachloropyridazine	Salmonella Inhibitor	0
NCGC00379024-02	Epothilone D	Tubulin polymerization inhibitor	0
NCGC00481572-02	GNE-317	Phosphatidylinositol 3-Kinase alpha isoform inhibitor	1
NCGC00165740-05	Benazepril	Angiotensin-I Converting Enzyme inhibitor	0
NCGC00094754-05	Phenacemide	Sodium channel alpha subunit Blocker	0
NCGC00532517-01	OZENOXACIN	Topoisomerase IV Inhibitor	0
NCGC00159337-04	Efavirenz	Reverse Transcriptase Inhibitor	0
NCGC00346537-04	LY-2228820	Mitogen-Activated Protein Kinase p38 inhibitor	0
NCGC00013687-03	Pyrophosphoric acid		0

NCGC00345056-07	Vidofludimus	Dihydroorotate Dehydrogenase inhibitor	1
NCGC00344111-03	BTP2	ORAI 1/2/3 Inhibitor	1
NCGC00408835-01	Frovatriptan succinate	Serotonin 1d (5-HT1d) receptor Agonist	0
NCGC00188865-05	Pazopanib	Vascular Endothelial Growth Factor Receptor 3 (VEGFR-3) inhibitor	0
NCGC00522007-01	PF-5006739	Casein Kinase 1 delta inhibitor	0
NCGC00378920-01	Falecalcitriol		0
NCGC00384304-02	EPZ011989	Histone-lysine N-methyltransferase EZH2 (Try641Phe Mutant) inhibitor	0
NCGC00179673-09	Idoxuridine	Anti-Herpes Simplex Virus Drug	0
NCGC00015686-11	Gallopamil hydrochloride	Voltage-gated L-type calcium channel Blocker	0
NCGC00408918-01	Ridaifen-B	PERQ amino acid-rich with GYF domain-containing protein 2 Binding Agent	1
NCGC00181355-02	Carpronium chloride	Muscarinic acetylcholine receptor Agonist	0
NCGC00164564-09	Emtricitabine	Reverse Transcriptase Inhibitor	0
NCGC00253438-12	BI-2536	Serine/threonine-protein kinase PLK1 inhibitor	1
NCGC00370909-03	BX-471	C-C chemokine receptor type 1 antagonist	1
NCGC00015810-26	Papaverine hydrochloride	Phosphodiesterase X (PDE10) Inhibitor	1
NCGC00016892-04	Iopamidol		0
NCGC00159521-09	Zoledronic acid monohydrate	Farnesyl Pyrophosphate Synthase inhibitor	0
NCGC00094969-05	Anisindione	Vitamin K-dependent gamma-carboxylase Inhibitor	0
NCGC00016599-10	Flucytosine	Thymidylate Synthase inhibitor	0
NCGC00263190-11	KU-0064	ATM Kinase inhibitor	0
NCGC00511354-01	TP-3654	Serine/threonine-protein kinase pim-1 inhibitor	0
NCGC00346433-06	Peramivir	Influenza Infection Inhibitor	0
NCGC00507847-01	Ozanimod	Sphingosine 1-phosphate receptor 5 agonist	0
NCGC00387215-01	PKI-166	Epidermal Growth Factor Receptor inhibitor	0
NCGC00094207-05	Pentolinium bitartrate		0
NCGC00016012-12	Triflupromazine hydrochloride	Muscarinic acetylcholine receptor M2 Antagonist	0
NCGC00015089-17	Amiloride hydrochloride	Epithelial Sodium Channels (ENaC) Blocker	0
NCGC00164533-02	Buformin	"AMP-activated protein kinase, AMPK Activator"	0
NCGC00179492-08	FLUNISOLIDE	Glucocorticoid Receptor agonist	0
NCGC00178831-03	Acriflavine hydrochloride	Hypoxia-inducible factor 1 alpha Interacts	0
NCGC00378596-08	Entospletinib	Tyrosine-protein kinase SYK inhibitor	1
NCGC00183016-01	Cefteram		0
NCGC00167531-02	Rivastigmine tartrate	Acetylcholinesterase Inhibitors	0
NCGC00344541-01	Adrenochrome semicarbazone		0
NCGC00016590-06	Azaperone		0
NCGC00345796-09	GSK-2636771	Phosphatidylinositol 3-Kinase beta isoform inhibitor	0

NCGC00346691-02	Canagliflozin	Sodium/glucose cotransporter 2 inhibitor	0
NCGC00386509-01	Fluticasone furoate	Glucocorticoid Receptor agonist	0
NCGC00068236-07	Gatifloxacin	DNA Topoisomerase IV Inhibitor	0
NCGC00178377-07	Amygdalin	Caspase-3 activator	0
NCGC00021202-03	Famotidine	Histamine H2 Receptor Antagonists	0
NCGC00160515-01	Acarbose	Alpha-Glucosidase inhibitor	0
NCGC00164128-02	Hexadecanoic acid, 1-methylethyl ester	N-acylsphingosine-amidohydrolase Inhibitor	0
NCGC00094852-04	L-Thyroxine	synthetic hormone	0
NCGC00248803-03	DL-Cysteine		0
NCGC00159458-11	Atorvastatin calcium	HMG-CoA Reductase inhibitor	0
NCGC00509939-01	IPI-549	Phosphatidylinositol 3-Kinase gamma isoform inhibitor	0
NCGC00263224-03	D-NMAPPD	Ceramidase inhibitor	0
NCGC00261970-01	BEMOTRIZINOL		0
NCGC00378965-05	XMD17-109	Mitogen-Activated Protein Kinase 7 inhibitor	1
NCGC00183912-01	FR-180204	Mitogen-Activated Protein Kinase 3 (ERK1) inhibitor	0
NCGC00183858-01	Blonanserin	Serotonin 2a (5-HT2a) receptor Antagonist	0
NCGC00385478-01	9-Methoxycamptothecin	<MOA Unknown> | Class: Alkaloid | Genus: N/A | Family: N/A | Species: N/A	1
NCGC00346596-02	Oxymatrine	mu/kappa-Opioid Agonist	0
NCGC00249938-01	Glymidine sodium		0
NCGC00346732-02	Hydroxocobalamin	Methionine synthase cofactor	0
NCGC00378599-01	Encorafenib	Serine/threonine-protein kinase B-raf (Val600Glu Mutant) inhibitor	0
NCGC00249894-01	Fosphenytoin	Sodium channel protein type VIII alpha subunit Inhibitor	0
NCGC00016010-09	Terbutaline hemisulfate	cAMP biosynthetic process Partial Agonist	0
NCGC00015754-17	Nilutamide	Androgen Receptor antagonist	0
NCGC00347358-02		<MOA Unknown> | Class: Terpenoid | Genus: N/A | Family: N/A | Species: N/A	1
NCGC00248064-08	CP-456773	NACHT, LRR and PYD domains-containing protein 3 Inflammasome inhibitor	0
NCGC00016666-06	Hydrocotarnine hydrobromide	Cytochrome P450 3A4 Inhibitor	0
NCGC00090991-02	N,N-Dimethylbenzylamine		0
NCGC00263097-03	Cediranib	Vascular Endothelial Growth Factor Receptor 2 (VEGFR-2) inhibitor	1
NCGC00510232-02	LXS-196	Protein Kinase C alpha type inhibitor	0
NCGC00344069-03	PI3-Kinase ? Inhibitor 2		1
NCGC00159483-06	Hydroxychloroquine sulfate	Anti-malarial	0
NCGC00346552-08	PD-318088	Dual Specificity Mitogen-Activated Protein Kinase Kinase 1 inhibitor	0
NCGC00183017-01	Ethyl 4-nitrophenyl ethylphosphonate	0	
NCGC00183877-01	Unoprostone isopropyl ester	Prostanoid FP receptor Agonist	0
NCGC00249908-01	Mefenorex		0

NCGC00347950-07	LGK-974	Protein Serine O-Palmitoleoyltransferase Porcupine inhibitor	1
NCGC00241036-03	NCGC00241036	Protein Jumonji inhibitor	0
NCGC00475727-01	Savolitinib	Hepatocyte Growth Factor Receptor inhibitor	0
NCGC00187912-13	Ibrutinib (PCI-32765)	Bruton's Tyrosine Kinase (BTK) inhibitor	1
NCGC00253573-01	Tepoxalin	platelet aggregation Inhibitor	0
NCGC00016069-13	Brimonidine	Alpha-2C adrenergic receptor Agonist	0
NCGC00379063-05	Voreloxin	DNA Topoisomerase II inhibitor	1
NCGC00181100-01	Ormeloxifene	Estrogen receptor Partial Agonist	0
NCGC00178526-03	APRAMYCIN SULFATE		0
NCGC00384854-01		Sodium/potassium-transporting ATPase Inhibitor	1
NCGC00015279-08	Meclofenoxate hydrochloride		0
NCGC00345849-02	NIBR-17	Phosphatidylinositol 3-Kinase alpha isoform inhibitor	1
NCGC00016564-17	Metformin	ATP synthase inhibitor	0
NCGC00263156-14	Nintedanib	Vascular Endothelial Growth Factor Receptor 3 (VEGFR-3) inhibitor	0
NCGC00250407-05	CHIR-265	Serine/threonine-protein kinase B-raf inhibitor	1
NCGC00162440-05	Leptomycin B	Exportin-1 antagonist	0
NCGC00371125-09	AR-A014418	Glycogen Synthase Kinase 3 (GSK-3) inhibitor	0
NCGC00346829-02	Fotemustine	DNA Damaging Drug	0
NCGC00346743-03	Givinostat hydrochloride	Histone deacetylase 1, class I inhibitor	1
NCGC00016023-18	Theobromine	Phosphodiesterase Inhibitor	0
NCGC00095124-31	Rosiglitazone	Peroxisome proliferator-activated receptor gamma agonist	0
NCGC00351609-09	STF-118804	Nicotinamide Phosphoribosyltransferase inhibitor	0
NCGC00164619-02	Anastrozole	Aromatase inhibitor	0
NCGC00186628-01	Butylscopolamine bromide	Muscarinic acetylcholine receptor Antagonist	0
NCGC00345825-02	BMS-3	LIM domain kinase 2 inhibitor	1
NCGC00160623-04	Octinoxate		0
NCGC00016526-07	Berberine	AMP-Activated Protein Kinase (AMPK) activator	0
NCGC00346889-03	PF-03716556	H+/K+-ATPase inhibitor	0
NCGC00179580-04	Biotin		0
NCGC00167498-02	Thiamphenicol glycinate		0
NCGC00346683-02	YM-201636	1-Phophatidylinositol 3-phosphate 5-kinase inhibitor	1
NCGC00183026-01	Dicethiamine hydrochloride		0
NCGC00346575-02	Dabigatran	Thrombin inhibitor	0
NCGC00510268-01	BAY-1895344	Serine-protein kinase ATR inhibitor	1
NCGC00386040-01		<MOA Unknown> | Class: Saponin | Genus: Anagallis | Family: Primulaceae | Species: monelli	1

NCGC00346705-02	PMSF	Serine Protease inhibitor	0
NCGC00507841-02	Crenigacestat	Notch Signaling inhibitor	0
NCGC00181350-14	Vatalanib	Vascular Endothelial Growth Factor Receptor 2 (VEGFR-2) inhibitor	0
NCGC00181153-01	Treosulfan	cell growth Inhibitor	0
NCGC00183861-01	(2R)-2-Propyloctanoic acid	S100 beta biosynthetic process Inhibitor	0
NCGC00241455-04	GW-501516	Peroxisome proliferator-activated receptor delta agonist	0
NCGC00167557-01	DL-Methionine methylsulfonium chloride	ERK1 and ERK2 cascade Activator	0
NCGC00510477-01	Lurbinectedin	DNA-Directed RNA Polymerase inhibitor	1
NCGC00387794-01	PX-13-17OH		1
NCGC00387301-01	Timiperone	serotonin receptor signaling pathway Modulator	0
NCGC00386429-01	PI-3065	Phosphatidylinositol 3-Kinase alpha isoform inhibitor	1
NCGC00163510-01	Aloe-emodin	Caspase-3 activator	0
NCGC00263125-03	SG-00529	mTOR Complex 1 (mTORC1) inhibitor	0
NCGC00015822-15	Pyrilamine	Histamine H1 receptor Antagonist	0
NCGC00164547-06	Zafirlukast	Cysteinyl leukotriene receptor 1 antagonist	0
NCGC00015590-08	Linopirdine	Potassium Voltage-Gated Channel Subfamily KQT Member 2 inhibitor	0
NCGC00074022-02	Pyricarbate	Bradykinin B2 receptor Antagonist	0
NCGC00262376-01	NCGC00262376	Flt3/IRAK Inhibitor	0
NCGC00347944-06	Hg-10-102-01	Leucine-Rich Repeat Kinase 2 inhibitor	0
NCGC00178782-04	Dicloxacillin sodium salt monohydrate	peptidoglycan biosynthetic process Inhibitor	0
NCGC00163683-02	Metenkephalin	Delta-type Opioid Receptor agonist	0
NCGC00509993-01	Ibiglustat	Ceramide Glucosyltransferase inhibitor	0
NCGC00253597-01	Isosulfan blue		0
NCGC00246187-06	Pyronaridine Tetraphosphate	Anti-malarial	1
NCGC00510487-01	Navoximod	Indoleamine 2,3-dioxygenase 1 inhibitor	0
NCGC00510504-01	TD-139	Galectin 3 inhibitor	0
NCGC00262641-04	S31-201	Signal Transducer and activator of Transcription 3 inhibitor	0
NCGC00498442-01	SHP-099	Tyrosine-protein phosphatase non-receptor type 11 inhibitor	0
NCGC00027930-07	Moclobemide	Monoamine oxidase A Inhibitor	0
NCGC00319175-03	Rilpivirine		1
NCGC00479176-02	Selonsertib	Mitogen-Activated Protein Kinase Kinase Kinase 5 inhibitor	0
NCGC00263086-08	BI-D1870	Ribosomal Protein S6 Kinase alpha-1 inhibitor	1
NCGC00165722-03	Plerixafor hydrochloride	Chemokine CXCR4 Receptor antagonist	0
NCGC00095107-18	Gliclazide	K(ATP) Channel Blocker	0
NCGC00167519-07	Ramatroban	Thromboxane A2 Receptor antagonist	0
NCGC00346894-04	Valspodar	Multidrug resistance protein 1 inhibitor	0

NCGC00161923-31	Picropodophyllin	Insulin-like growth factor 1 receptor inhibitor	1
NCGC00346449-02	Parogrelil	Phosphodiesterase 3A inhibitor	0
NCGC00178810-03	Novobiocin sodium	Heat Shock Protein 90 (hsp90) Inhibitor	0
NCGC00164602-03	RACTOPAMINE HYDROCHLORIDE	Beta-2 adrenergic receptor Agonist	0
NCGC00253646-08	Agomelatine	5-HT2C Receptor antagonist	0
NCGC00025297-04	Gabexate mesilate	NF-kappaB (NFKB) Activation inhibitor	0
NCGC00345818-01	Ximelagatran	Thrombin Inhibitor	0
NCGC00017337-17	(E)-Capsaicin	Transient receptor potential cation channel subfamily V member 1 Agonist	0
NCGC00244252-03	TG-101348	Tyrosine-protein kinase JAK2 inhibitor	1
NCGC00015054-12	Tacrine hydrochloride	Acetylcholinesterase Inhibitor	0
NCGC00510464-01	P21d hydrochloride	Protein-tyrosine kinase 6 inhibitor	0
NCGC00532516-01	IOBITRIDOL		0
NCGC00253585-01	Cyclomethycaine		0
NCGC00178707-05	Ramelteon	Melatonin MT1/MT2 Agonist	0
NCGC00015594-09	LOXOPROFEN SODIUM		0
NCGC00348378-01	FOSFOMYCIN	UDP-N-acetylglucosamine 1-carboxyvinyltransferase Inhibitor	0
NCGC00249896-01	Cinnamedrine		0
NCGC00181094-01	Sodium tyropanoate		0
NCGC00178241-04	Dichloro(ethylenediamine)platinum(II)	DNA Alkylating Drug	0
NCGC00015437-22	Fenofibrate	Peroxisome proliferator-activated receptor alpha agonist	0
NCGC00159479-05	Methyl nicotinate		0
NCGC00023193-06	Triamcinolone acetonide	Glucocorticoid Receptor agonist	0
NCGC00263228-01	BI-99179	Fatty Acid Synthase inhibitor	0
NCGC00091021-01	Trichloroacetic acid		0
NCGC00016710-05	Clemastine fumarate	Histamine H1 Receptor Antagonists	0
NCGC00179676-03	Lynestrenol		0
NCGC00162068-06	Opipramol dihydrochloride	Serotonin 2 (5-HT2) receptor Antagonist	0
NCGC00167954-03	EGFR Inhibitor		1
NCGC00025072-08	PD-128907	Dopamine Receptor D3 agonist	0
NCGC00241982-07	GSK-269962A	Rho-associated protein kinase 1 inhibitor	1
NCGC00263109-15	GDC-0980	Phosphatidylinositol 3-Kinase alpha isoform inhibitor	1
NCGC00346527-03	Roxadustat	Hypoxia-Inducible Factor Prolyl Hydroxylase inhibitor	0
NCGC00160527-03	Mosapride citrate	5-HT4 Antagonist	0
NCGC00017363-32		Protien Synthesis inhibitor	1
NCGC00164588-06	Malotilate	Liver Protein Metabolism modulator	0
NCGC00164507-02	Nifuroxime		0
NCGC00480730-01	AMG PERK 44	Protein kinase R (PKR)-like endoplasmic reticulum kinase (PERK)	0
NCGC00091014-06	Hydroxyflutamide	Androgen Receptor antagonist	0

NCGC00167448-02	Trepibutone	Catechol O-methyltransferase Inhibitor	0
NCGC00384608-01		<MOA Unknown> | Class: Polyketide | Genus: N/A | Family: N/A | Species: N/A	1
NCGC00344537-01	L-Glutathione oxidized disodium salt	Glutathione reductase Substrate	0
NCGC00167552-01	Piperocaine hydrochloride	Nicotinic acetylcholine receptor Blocker	0
NCGC00181092-01	Ipodate sodium		0
NCGC00090774-05	Nitazoxanide	antiprotozoal agent	0
NCGC00167785-11	PAC-1	Caspase-3 activator	1
NCGC00386237-01	Brintellix	5-HT1A Receptor agonist	1
NCGC00483925-02	Glesatinib	Hepatocyte Growth Factor Receptor inhibitor	0
NCGC00015924-24	Synephrine	alpha1-Adrenoceptor Agonist	0
NCGC00016034-05	Tulobuterol hydrochloride	Beta-2 adrenergic receptor Agonist	0
NCGC00373228-02	Fluorometholone Acetate		0
NCGC00164344-04	Symclosene		0
NCGC00182987-01	Esonarimod	cytokine production involved in inflammatory response Inhibitor	0
NCGC00016706-10	Parbendazole	Tubulin polymerization inhibitor	0
NCGC00174007-04	Pyrantel tartrate	Antiparasitic Agent	0
NCGC00510794-01	KIN1408		1
NCGC00183029-02	Troxipide	reactive oxygen species biosynthetic process Inhibitor	0
NCGC00021137-04	ESTROPIPATE		0
NCGC00182965-01	Biperiden	Anticholinergic	0
NCGC00182704-02	Luliconazole	Lanosterol 14-alpha demethylase Inhibitor	0
NCGC00160670-08	Mecarbinate	antiinfluenza drug	0
NCGC00344507-02	Calhex-231	Calcium-Sensing Receptor (CaSR) Agonist	0
NCGC00159422-10	Tiopronin	Myeloperoxidase inhibitor	0
NCGC00386667-01	P2Y14 Antagonist Prodrug 7j hydrochloride	Purinergic receptor P2Y14 Antagonist	1
NCGC00181170-02	Bendamustine	DNA Alkylating Drug	0
NCGC00538144-01		Taenia pisiformis Inhibitor	0
NCGC00507873-02	EAI045	allosteric EGFR inhibitor (L858R/T790M-specific)	0
NCGC00249612-02	Ixazomib citrate	Proteasome inhibitor	1
NCGC00344558-01	Verteporfin	photosensitizer	1
NCGC00166142-04	Diperodon hydrochloride	sodium ion transmembrane transport Inhibitor	0
NCGC00162073-12	H-89	Protein Kinase A inhibitor	0
NCGC00510188-01	ABT-450		0
NCGC00263090-09	AZ-3146	Dual Specificity protein kinase TTK inhibitor	0
NCGC00346490-06	OSU-03012	3-Phosphoinositide Dependent Kinase 1 inhibitor	0
NCGC00378973-06	Pilaralisib	Phosphatidylinositol 3-Kinase alpha isoform inhibitor	1
NCGC00018251-05	Methyl delta-aminolevulinate hydrochloride	Porphyrin and chlorophyll metabolism Modulator	0

<table>
<tr><td>NCGC00168809-03</td><td></td><td><MOA Unknown> | Class: Steroid | Genus: Nerium | Family: Apocynaceae | Species: oleander</td><td>1</td></tr>
<tr><td>NCGC00161927-04</td><td>DMEP</td><td>Antimitotic Agent</td><td>0</td></tr>
<tr><td>NCGC00346942-01</td><td>WHI-P97</td><td>Epidermal Growth Factor Receptor inhibitor</td><td>0</td></tr>
<tr><td>NCGC00091071-08</td><td>p,p'-DDT</td><td></td><td>0</td></tr>
<tr><td>NCGC00166061-02</td><td>L-Cysteine ethyl ester hydrochloride</td><td>0</td><td></td></tr>
<tr><td>NCGC00161621-13</td><td>Cepharanthine</td><td></td><td>0</td></tr>
<tr><td>NCGC00021123-03</td><td>Diatrizoic acid dihydrate</td><td></td><td>0</td></tr>
<tr><td>NCGC00015802-18</td><td>Pimozide</td><td>HERG Inhibitor</td><td>0</td></tr>
<tr><td>NCGC00346952-01</td><td>Cdk4/6 Inhibitor IV</td><td>CDK4/6 Inhibitor</td><td>0</td></tr>
<tr><td>NCGC00263150-04</td><td>Andarine</td><td>Selective Androgen Receptor modulator (SARM)</td><td>0</td></tr>
<tr><td>NCGC00249904-01</td><td>Butamisole</td><td>Nicotinic acetylcholine receptor alpha subunit Agonist</td><td>0</td></tr>
<tr><td>NCGC00384193-03</td><td>SCH-51344</td><td>DNA Damaging Drug</td><td>0</td></tr>
<tr><td>NCGC00386356-05</td><td>Bepotastine (Beslilate)</td><td>Histamine H1 receptor Antagonist</td><td>0</td></tr>
<tr><td>NCGC00522488-01</td><td>JNJ-42165279</td><td>Fatty Acid Amide Hydrolase inhibitor</td><td>0</td></tr>
<tr><td>NCGC00388392-01</td><td>Bergenin monohydrate</td><td>Protein Tyrosine Phosphatase (PTP) Inhibitors</td><td>0</td></tr>
<tr><td>NCGC00346660-03</td><td>10-Deacetylbaccatin</td><td>antineoplastic intermediate</td><td>0</td></tr>
<tr><td>NCGC00347278-03</td><td>A-443654</td><td>AKT serine/threonine kinase inhibitor</td><td>1</td></tr>
<tr><td>NCGC00163736-04</td><td>Imidacloprid</td><td></td><td>0</td></tr>
<tr><td>NCGC00167981-03</td><td>Isosorbide mononitrate</td><td></td><td>0</td></tr>
<tr><td>NCGC00160543-01</td><td>Pregnandiol</td><td>G-protein coupled bile acid receptor 1 Agonist</td><td>0</td></tr>
<tr><td>NCGC00091643-10</td><td>2-Mercaptobenzothiazole</td><td>Thyroid peroxidase Inhibitor</td><td>0</td></tr>
<tr><td>NCGC00015672-15</td><td>Morin hydrate</td><td>DNA-3-Methyladenine Glycosylase inhibitor</td><td>0</td></tr>
<tr><td>NCGC00167433-05</td><td>Rocuronium bromide</td><td>Neuronal acetylcholine receptor subunit alpha-1 antagonist</td><td>0</td></tr>
<tr><td>NCGC00262598-02</td><td>Atracurium besylate</td><td></td><td>0</td></tr>
<tr><td>NCGC00091010-03</td><td>Domiphen bromide</td><td>HERG Inhibitor</td><td>0</td></tr>
<tr><td>NCGC00183856-01</td><td>Phenyl 11-iodo-10-undecynoate</td><td></td><td>0</td></tr>
<tr><td>NCGC00346936-13</td><td>GNF-5837</td><td>Neurotrophic Tyrosine Kinase Receptors (NTRK) inhibitor</td><td>1</td></tr>
<tr><td>NCGC00181323-02</td><td>Squalene</td><td>Steroid biosynthesis Modulator</td><td>0</td></tr>
<tr><td>NCGC00183832-01</td><td>Ro 22-9194</td><td>Thromboxane A2 receptor Inhibitor</td><td>0</td></tr>
<tr><td>NCGC00346569-03</td><td>KW-2449</td><td>Aurora kinase A inhibitor</td><td>0</td></tr>
<tr><td>NCGC00346890-02</td><td>Banoxantrone</td><td>DNA Topoisomerase II inhibitor</td><td>0</td></tr>
<tr><td>NCGC00163732-03</td><td>Moxidectin</td><td></td><td>1</td></tr>
<tr><td>NCGC00164492-02</td><td>Sulfisomidine</td><td></td><td>0</td></tr>
<tr><td>NCGC00386198-07</td><td>Dalcetrapib</td><td>Cholesteryl Ester Transfer Protein inhibitor</td><td>0</td></tr>
<tr><td>NCGC00346651-08</td><td>Nepicastat hydrochloride</td><td>Dopamine beta-hydroxylase inhibitor</td><td>0</td></tr>
<tr><td>NCGC00016285-09</td><td>Sulfanilamide</td><td>Carbonic Anhydrase 1 inhibitor</td><td>0</td></tr>
<tr><td>NCGC00346842-04</td><td>Indoximod</td><td>Indoleamine 2,3-dioxygenase 1 inhibitor</td><td>0</td></tr>
</table>

NCGC00475741-01	TAK-441	Smoothened Receptor antagonist	0
NCGC00159522-05	Cephalexin	Cephalosporin antibiotic	0
NCGC00263195-04	AV-412	Epidermal Growth Factor Receptor inhibitor	0
NCGC00095255-08	dl-alpha-Tocopheryl acetate		0
NCGC00181915-01	Mepranoprofen arbamel		0
NCGC00346649-04	PKI-402	Phosphatidylinositol 3-Kinase alpha isoform inhibitor	0
NCGC00510073-01	AQX-MN-100	Phosphatidylinositol 3,4,5-Trisphosphate 5-Phosphatase 1 inhibitor	0
NCGC00015747-09	Nicardipine	Ca channel blocker - DHP class	0
NCGC00384729-01		<MOA Unknown> | Class: Polyketide | Genus: N/A | Family: N/A | Species: N/A	1
NCGC00263108-02	Vorapaxar	Proteinase-activated receptor 1 antagonist	0
NCGC00167543-05	CEFTIOFUR HYDROCHLORIDE	Cephalosporin antibiotic	0
NCGC00344528-01	TRYPAN BLUE		0
NCGC00181129-22	Dasatinib	Bcr-Abl Kinase inhibitor	1
NCGC00507856-01	PD-1/PD-L1 Inhibitor 1	Programmed cell death protein 1/Programmed cell death protein ligand 1 inhibitor	0
NCGC00164552-06	Bromfenac Na		0
NCGC00390672-01	Seviteronel	Steroid 17-alpha-hydroxylase/17,20 lyase inhibitor	0
NCGC00181296-02	Seratrodast	Thromboxane A2 Receptor antagonist	0
NCGC00499576-01	HDACi-01	Histone deacetylase 1, class I inhibitor	1
NCGC00182998-01	Bisoxatin acetate		0
NCGC00263119-07	Ruboxistaurin mesylate	Protein Kinase C beta type inhibitor	0
NCGC00249391-11	NVP-BHG712	Serine/threonine-protein kinase B-raf inhibitor	1
NCGC00483038-01	TAK-599		0
NCGC00179590-04	Syrosingopine		0
NCGC00185003-01			0
NCGC00373041-03	Favipiravir	Anti-viral	0
NCGC00179240-04	Levocabastine hydrochloride	Neurotensin receptor type 2 Inverse Agonist	0
NCGC00386235-04	Cobicistat	Cytochrome P450 3A Inhibitor	0
NCGC00253713-01	Fenticonazole nitrate	Antifungal Agent	0
NCGC00179313-05	Imidazolidinyl urea	T-lymphocyte activation antigen CD86 Activator	0
NCGC00163953-03	Allethrin	Testis-specific androgen-binding protein Binding Agent	0
NCGC00015580-22	Ketotifen	Histamine H1 Receptor antagonist	0
NCGC00508883-01	AZ-9482	Poly [ADP-ribose] polymerase 1 inhibitor	0
NCGC00345808-01	BP-1-102	Signal Transducer and activator of Transcription 3 inhibitor	0
NCGC00015146-17	Brompheniramine (maleate)	Histamine H1 receptor Antagonist	0
NCGC00343986-02	PDGF receptor tyrosine kinase inhibitor IV	Platelet-derived growth factor receptor beta Inhibitor	1
NCGC00095166-04	Clopidol		0

NCGC00093936-08	D-Glutamine	Protein-glutamine gamma-glutamyltransferase Substrate	0
NCGC00167492-03	Lercanidipine hydrochloride	Voltage-gated L-type calcium channel Blocker	0
NCGC00181306-04	Docetaxel	Microtubule-Stabilizing agent	1
NCGC00094985-05	Zoalene		0
NCGC00253596-01	Ethchlorvynol	GABA-A receptor; anion channel Positive Allosteric Modulator	0
NCGC00015603-08	Lomefloxacin hydrochloride	Quinoline Antibiotic	0
NCGC00179290-03	Pivampicillin		0
NCGC00164559-03	Duloxetine hydrochloride	Serotonin Transporter (SERT) Inhibitor	0
NCGC00013226-32		Dopamine Receptor D2 antagonist	0
NCGC00532515-01	ROBENACOXIB	Cyclooxygenase-2 Inhibitor	0
NCGC00250411-03	Veliflapon	Arachidonate 5-Lipoxygenase-Activating Protein inhibitor	0
NCGC00505919-02	S63845	Induced Myeloid Leukemia Cell Differentiation Protein Mcl-1 inhibitor	0
NCGC00181122-01	2-Dodecylisoquinolinium tosylate		1
NCGC00092309-06	SKI-II	Valosin-containing protein (VCP) p97 / Transitional endoplasmic reticulum ATPase inhibitor	0
NCGC00165880-14	Reversine	Adenosine Receptor A3 antagonist	1
NCGC00015673-19	Minoxidil	K(ATP) Channel Activator	0
NCGC00160611-03	1-Ethyl-2-pyrrolidinone		0
NCGC00091886-04	Denatonium benzoate	Glycolysis / Gluconeogenesis Inhibitor	0
NCGC00185743-02	Cloflucarban		1
NCGC00346633-03	SNX-2112	Heat Shock Protein 90 (Hsp90) inhibitor	0
NCGC00094772-04	PYRVINIUM PAMOATE	Cryptosporidium parvum Inhibitor	1
NCGC00250380-10	AZ-628	Serine/threonine-protein kinase B-raf inhibitor	0
NCGC00250399-02	Vemurafenib	Serine/threonine-protein kinase B-raf inhibitor	0
NCGC00244250-02	NCGC00244250	Tyk 2 Inhibitor	0
NCGC00249913-01	Isoniazid pyruvate	Isocitrate lyase Inhibitor	0
NCGC00484075-02	AZD-8835	Phosphatidylinositol 3-Kinase alpha isoform inhibitor	0
NCGC00167984-01	Tiquizium bromide		0
NCGC00015829-14	PROMAZINE HYDROCHLORIDE	Serotonin 2a (5-HT2a) receptor Antagonist	0
NCGC00179493-03	Solanine alpha	Cholinesterase Inhibitor	0
NCGC00178086-03	Aminolevulinic acid hydrochloride	DNA Inhibitor	0
NCGC00351592-01	Manassantin A	Hypoxia-inducible factor 1-alpha inhibitor	0
NCGC00164493-02	Dipyrithione		1
NCGC00263112-02	GSK-461364A	Serine/threonine-protein kinase PLK1 inhibitor	0
NCGC00510484-01	Pemafibrate	Peroxisome proliferator-activated receptor alpha modulator	0
NCGC00379091-01	BIX-02565	Ribosomal Protein S6 Kinase alpha-3 inhibitor	0

NCGC00381292-01		<MOA Unknown> | Class: Steroid | Genus: Thevetia | Family: Apocynaceae | Species: plumeriaefolia	1
NCGC00346637-05	Odanacatib	Cathepsin K inhibitor	0
NCGC00179596-03	Butoconazole nitrate	Anti-fungal	0
NCGC00229512-05	BMS-509744	Tyrosine-protein kinase ITK/TSK inhibitor	0
NCGC00163798-06	Triethylene glycol		0
NCGC00347770-02		<MOA Unknown> | Class: Terpenoid | Genus: N/A | Family: N/A | Species: N/A	1
NCGC00160615-03	Sucrose octaacetate		0
NCGC00379079-03	Cefoselis (sulfate)	Antibiotic	0
NCGC00249917-01	Cloxazolam	GABA-A receptor; benzodiazepine site Agonist	0
NCGC00351479-01	(R)-PFI-2	Histone-lysine N-methyltransferase SETD7 inhibitor	0
NCGC00178317-04	Foscarnet Sodium	Human immunodeficiency virus type 1 reverse transcriptase Inhibitor	0
NCGC00238454-10	PIK-90	Phosphatidylinositol 3-Kinase alpha isoform inhibitor	0
NCGC00346663-04	Geniposidic acid	Antioxidant	0
NCGC00166122-01	Perflexane		0
NCGC00015462-14	Glipizide	K(ATP) Channel activator	0
NCGC00015400-05	Enoximone	Phosphodiesterase 3 Inhibitor	0
NCGC00253592-01	Menadiol	regulation of blood coagulation Activator	0
NCGC00015542-07	Ibudilast	Phosphodiesterase 4A Inhibitor	0
NCGC00025220-11	Purvalanol B	Cyclin-Dependent Kinase 1 inhibitor	0
NCGC00167436-02	Azelnidipine	Ca channel blocker - DHP class	0
NCGC00186629-04	ATP (disodium salt)		0
NCGC00378574-01	Brexpiprazole	Dopamine D3 receptor Partial Agonist	0
NCGC00167781-12	CETIRIZINE HYDROCHLORIDE	Histamine H1 Receptor Antagonists	0
NCGC00164597-02	Ropivacaine hydrochloride	Voltage-gated potassium channel subunit Kv1.5 Inhibitor	0
NCGC00253577-01	Lenampicillin hydrochloride		0
NCGC00386436-01	EPZ-015666	Protein arginine N-methyltransferase 5 inhibitor	0
NCGC00250384-02	PHA-408	inhibitor of nuclear factor kappa B kinase subunit beta inhibitor	0
NCGC00263271-04	Alisertib	Aurora kinase A inhibitor	0
NCGC00346682-08	PA-824	Nitric Oxide Donor	0
NCGC00249931-01	Docarpamine	Dopamine D1 receptor Agonist	0
NCGC00388356-01	Cytochalasin A		1
NCGC00166020-03	Ethyl 3-aminobenzoate methanesulfonic acid salt	Sodium channel alpha subunit Blocker	0
NCGC00263140-03	Talmapimod	Mitogen-Activated Protein Kinase p38 inhibitor	0
NCGC00181045-01	Pyridoxamine phosphate		0
NCGC00346955-02	A-770041	Tyrosine-protein kinase Lck inhibitor	0
NCGC00507682-01	Emodepside	synaptic transmission Modulator	0

NCGC00386735-01	AZD-8797	Chemokine CX3CR1 Receptor antagonist	0
NCGC00253576-01	Amelometasone		0
NCGC00345807-02	CAA-0225	Cathepsin L inhibitor	0
NCGC00178318-04	Sodium 2-mercaptoethanesulfonate	Antioxidant	0
NCGC00015467-20	Glybenclamide	Sulfonylurea receptor 2 Modulator	0
NCGC00263021-04	MLN-120B	inhibitor of nuclear factor kappa B kinase subunit beta inhibitor	0
NCGC00378841-01	Levomefolate calcium		0
NCGC00475733-01	E-7820	Integrin Receptor antagonist	0
NCGC00480775-01	INCB8761	C-C chemokine receptor type 2 antagonist	0
NCGC00390545-02	Valrubicin	DNA Topoisomerase II inhibitor	0
NCGC00094574-15	Fipronil		0
NCGC00179685-03	Isoflupredone acetate		0
NCGC00351604-03	EPZ-005687	Histone-lysine N-methyltransferase EZH2 inhibitor	0
NCGC00346514-07	Bafetinib	Bcr-Abl Kinase inhibitor	0
NCGC00386948-01	AMG-837	Free Fatty Acid Receptor 1 agonist	0
NCGC00016557-06	Ethaverine hydrochloride	Voltage-gated L-type calcium channel Blocker	0
NCGC00238453-16	TAE-684	ALK Tyrosine Kinase Receptor inhibitor	1
NCGC00183831-01	Aceglatone		0
NCGC00182546-02	Gefarnate	positive regulation of mucus secretion Interacts	0
NCGC00016834-07	Fenbufen	Prostaglandin G/H synthase 2 Inhibitor	0
NCGC00185767-01	Spiramycin II	Bacterial 70S ribosome Binding Agent	0
NCGC00015273-15	Chlorpromazine hydrochloride	Histamine H1 receptor Antagonist	0
NCGC00021122-08	Alverine citrate		0
NCGC00181011-02	Gimeracil	Dihydropyrimidine Dehydrogenase inhibitor	0
NCGC00253867-02	Prucalopride	5-HT4 Agonist	0
NCGC00522022-01	Befunolol	Adrenergic receptor beta Antagonist	0
NCGC00385432-01		<MOA Unknown> | Class: Shikimate | Genus: N/A | Family: N/A | Species: N/A	1
NCGC00159467-03	(-)-Cocaine hydrochloride	Serotonin transporter Inhibitor	0
NCGC00090887-04	Phenyl salicylate	Estrogen receptor alpha Agonist	0
NCGC00021152-06	Hydroxyzine	antiparasitic agent	0
NCGC00385764-01		<MOA Unknown> | Class: N/A | Genus: N/A | Family: N/A | Species: N/A	1
NCGC00344330-02	Elaidic Acid	SiHa Inhibitor	0
NCGC00015365-22	Daidzein	Estrogen Receptor beta agonist	0
NCGC00188863-02	Dronedarone hydrochloride	Alpha-1A Adrenergic Receptor antagonist	1
NCGC00015838-06	Propionylpromazine hydrochloride	Trypanothione reductase Inhibitor	0
NCGC00025329-02	SB-239063	Tumor necrosis factor alpha Production inhibitor	0
NCGC00163107-07	CITCO	Nuclear Receptor Subfamily 1 Group I Member 3 agonist	0
NCGC00178711-03	Gemifloxacin mesylate	Topoisomerase IV subunit A Inhibitor	0

<table>
<tr><td>NCGC00166299-03</td><td>BISMUTH SUBSALICYLATE</td><td></td><td>0</td></tr>
<tr><td>NCGC00263655-01</td><td>Huperzine A</td><td>Acetylcholinesterase Inhibitors</td><td>0</td></tr>
<tr><td>NCGC00385528-01</td><td></td><td><MOA Unknown> | Class: Steroid | Genus: Tephrosia | Family: Fabaceae | Species: nana</td><td>1</td></tr>
<tr><td>NCGC00347936-06</td><td>Ak-7</td><td>Histone Deacetylase SIRT2 Inhibitor</td><td>0</td></tr>
<tr><td>NCGC00164544-02</td><td>Lanoconazole</td><td>Lanosterol 14-alpha demethylase Inhibitor</td><td>0</td></tr>
<tr><td>NCGC00182972-01</td><td>Hexocyclium</td><td>Muscarinic acetylcholine receptor M5 Antagonist</td><td>0</td></tr>
<tr><td>NCGC00381297-01</td><td></td><td><MOA Unknown> | Class: Terpenoid | Genus: N/A | Family: N/A | Species: N/A</td><td>1</td></tr>
<tr><td>NCGC00386215-05</td><td>Alogliptin (SYR-322)</td><td>Dipeptidyl Peptidase IV inhibitor</td><td>0</td></tr>
<tr><td>NCGC00016013-06</td><td>Trimipramine maleate</td><td>Serotonin 2 (5-HT2) receptor Antagonist</td><td>0</td></tr>
<tr><td>NCGC00178500-19</td><td>Streptozocin</td><td>DNA Alkylating Drug</td><td>0</td></tr>
<tr><td>NCGC00167958-05</td><td>Imipenem</td><td>Penicillin-binding protein 2 Inhibitor</td><td>0</td></tr>
<tr><td>NCGC00346470-02</td><td>Zilpaterol??HCl</td><td>beta1-Adrenoceptor Antagonists</td><td>0</td></tr>
<tr><td>NCGC00168785-05</td><td>Diclazuril</td><td>antiprotozoal agent</td><td>0</td></tr>
<tr><td>NCGC00346582-03</td><td>Mubritinib</td><td>Receptor tyrosine-protein kinase NEU (HER2; erbB2) inhibitor</td><td>0</td></tr>
<tr><td>NCGC00179630-03</td><td>Homatropine hydrobromide</td><td>Muscarinic acetylcholine receptor Antagonist</td><td>0</td></tr>
<tr><td>NCGC00390775-03</td><td>Atractylenolide I</td><td>TNF-alpha Production Inhibitor</td><td>0</td></tr>
<tr><td>NCGC00485206-01</td><td>Lometrexol hydrate</td><td>GAR transformylase Inhibitor</td><td>1</td></tr>
<tr><td>NCGC00480898-01</td><td>Cevipabulin</td><td>Microtubule-Stabilizing agent</td><td>1</td></tr>
<tr><td>NCGC00387189-01</td><td>AL-8697</td><td>Proto-oncogene tyrosine-protein kinase receptor Ret inhibitor</td><td>0</td></tr>
<tr><td>NCGC00386281-05</td><td>Fidaxomicin</td><td></td><td>0</td></tr>
<tr><td>NCGC00244257-02</td><td>Momelotinib</td><td>Tyrosine-protein kinase JAK1 inhibitor</td><td>1</td></tr>
<tr><td>NCGC00263199-04</td><td>AMG-Tie2-1</td><td>Angiopoietin-1 Receptor inhibitor</td><td>1</td></tr>
<tr><td>NCGC00510509-01</td><td>Vesnarinone</td><td>Valosin-containing protein (VCP) p97 / Transitional endoplasmic reticulum ATPase inhibitor</td><td>0</td></tr>
<tr><td>NCGC00522012-01</td><td>CEFTOBIPROLE MEDOCARIL SODIUM</td><td>0</td><td></td></tr>
<tr><td>NCGC00163376-14</td><td>PP1</td><td></td><td>1</td></tr>
<tr><td>NCGC00344508-02</td><td>NPS-R568</td><td>Calcium-Sensing Receptor agonist</td><td>0</td></tr>
<tr><td>NCGC00182622-03</td><td>EFLOXATE</td><td></td><td>0</td></tr>
<tr><td>NCGC00390584-01</td><td>Exatecan</td><td>DNA Topoisomerase I inhibitor</td><td>1</td></tr>
<tr><td>NCGC00093926-10</td><td>Doxifluridine</td><td>Thymidylate Synthase inhibitor</td><td>0</td></tr>
<tr><td>NCGC00186528-19</td><td>TEPP-46</td><td>Pyruvate kinase M2 activator</td><td>0</td></tr>
<tr><td>NCGC00166206-04</td><td>1,2-Benzenedicarboxaldehyde</td><td></td><td>0</td></tr>
<tr><td>NCGC00510444-01</td><td>Epiblastin A</td><td>Casein Kinase 1 alpha inhibitor</td><td>1</td></tr>
<tr><td>NCGC00347676-02</td><td>Schisandrol B</td><td>MAPK signaling pathway Modulator</td><td>0</td></tr>
<tr><td>NCGC00346460-05</td><td>BMS-536924</td><td>Insulin-like growth factor 1 receptor inhibitor</td><td>1</td></tr>
<tr><td>NCGC00263280-01</td><td>Ac-SAH-p53-8</td><td>HDM2/HDMX Inhibitor</td><td>0</td></tr>
<tr><td>NCGC00480885-01</td><td>INT-747 (Obeticholic acid)</td><td>Farnesoid X receptor agonist</td><td>0</td></tr>
<tr><td>NCGC00181097-01</td><td>Dimefline hydrochloride</td><td></td><td>0</td></tr>
</table>

NCGC00263143-07	VER-155008	Heat Shock Protein 70 inhibitor	0
NCGC00183598-02	Ximelagatran	Thrombin Inhibitor	0
NCGC00263107-03	GB-83	Proteinase-Activated Receptor 2 antagonist	0
NCGC00015116-20	Aniracetam	AMPA receptor modulator	0
NCGC00371134-08	PR-619	Ubiquitin carboxyl-terminal hydrolase isozyme L3 Inhibitor	1
NCGC00346826-01	HPI-1	Smoothened Receptor antagonist	0
NCGC00242497-14	Vismodegib	Smoothened Receptor antagonist	0
NCGC00183859-01	Prifinium bromide	Muscarinic acetylcholine receptor Antagonist	0
NCGC00091502-11	Coumarin	Vitamin k epoxide reductase complex subunit 1 isoform 1 Inhibitor	0
NCGC00263155-04	Belinostat	Histone deacetylase 1, class I inhibitor	1
NCGC00253590-01	Variotin		0
NCGC00346699-02	TAK-285	Epidermal Growth Factor Receptor inhibitor	1
NCGC00344764-12	CPI-613	Pyruvate dehydrogenase (PDH) inhibitor	0
NCGC00186637-01	Chromomycin A₃ from Streptomyces griseus	0	
NCGC00389251-02	AZ-191	Dual-Specificity Tyrosine-(Y)-Phosphorylation Regulated Kinase 1A inhibitor	0
NCGC00159397-03	Iodoantipyrine		0
NCGC00016375-09	Sulfadimethoxine	Antibiotic	0
NCGC00095054-05	Resorcinol monoacetate		0
NCGC00538147-01			0
NCGC00346822-02	Idronoxil	IAP inhibitor	1
NCGC00016241-10	Furosemide	Loop Diuretics	0
NCGC00015335-11	Diphenhydramine hydrochloride	Histamine H1 Receptor Antagonist	0
NCGC00165841-02	NH125		1

Table S7. Testing of the predicted anti-SARS-CoV-2 compounds using in vitro PP assay

Sample.ID	Sample.Name	IC50 µM_activity	IC50 µM_viability	efficacy_activity	efficacy_viability	Curve rank_activity	Curve rank_viability	class
NCGC00166392-01		1.37±0.00	NaN±NA	-83.25±8.42	-17.57±24.85	-9.00±0.00	0.00±0.00	active
NCGC00416125-01		NaN±NA	45.35±24.53	7.75±10.96	-54.95±2.08	0.00±0.00	-3.50±2.12	inactive
NCGC00601147-01		7.74±1.25	5.48±0.89	-110.41±37.27	-122.42±2.06	-9.00±0.00	-9.00±0.00	inactive
NCGC00634974-01		12.92±6.07	8.63±NA	-54.22±29.31	-17.50±52.52	-2.00±0.00	-1.50±2.12	inactive
NCGC00240619-01		NaN±NA	NaN±NA	-30.16±12.91	7.72±28.42	0.00±0.00	0.00±0.00	inactive
NCGC00188918-01		8.63±NA	12.35±16.92	22.62±14.18	-46.92±28.88	1.00±1.41	-2.50±0.71	inactive
NCGC00378438-01		21.62±30.57	19.88±26.39	-61.46±18.14	49.38±12.14	-2.00±0.00	2.50±0.71	active
NCGC00537529-01		2.05±0.17	22.99±1.87	-72.78±1.07	-112.63±1.45	-9.00±0.00	-7.00±0.00	inactive
NCGC00455477-01		34.57±5.60	14.51±1.18	-87.86±12.62	-92.48±14.11	-4.50±3.54	-7.00±0.00	inactive
NCGC00681477-01		0.00±NA	NaN±NA	-31.84±45.02	0.00±0.00	-1.50±2.12	0.00±0.00	inconclusive
NCGC00625590-01		19.31±0.00	19.31±0.00	-68.68±12.40	-63.56±14.00	-5.00±0.00	-3.50±2.12	inactive
NCGC00645468-01		NaN±NA	18.26±1.49	8.94±12.64	-53.35±25.20	0.00±0.00	-3.50±2.12	inactive
NCGC00609658-01		NaN±NA	NaN±NA	8.45±11.96	-12.19±51.63	0.00±0.00	0.00±0.00	inactive
NCGC00249682-01		26.14±6.32	NaN±NA	-82.10±1.97	-18.13±25.64	-4.50±3.54	0.00±0.00	active
NCGC00639653-01		12.93±1.05	5.77±2.71	-110.31±0.78	-100.98±2.63	-9.00±0.00	-9.00±0.00	inactive
NCGC00437819-01		NaN±NA	NaN±NA	-24.55±NA	0.00±0.00	0.00±0.00	0.00±0.00	inactive
NCGC00455519-01		8.63±NA	19.44±3.15	37.55±13.51	-64.72±4.23	1.00±1.41	-3.50±2.12	inactive
NCGC00181471-02		24.48±3.97	22.99±1.87	-86.07±6.12	-132.09±31.74	-4.50±3.54	-7.00±0.00	inactive
NCGC00529416-01		43.24±0.00	8.63±0.00	-66.77±7.80	-72.78±14.15	-2.00±0.00	-8.50±0.71	inactive

NCGC00506297-01		31.28±16.92	14.72±17.76	-100.07±17.48	-51.40±14.93	-2.00±0.00	-2.00±0.00	inactive
NCGC00606028-01		43.24±0.00	8.64±9.48	-107.85±26.15	-83.69±20.54	-2.00±0.00	-7.50±0.71	inactive
NCGC00597009-01		NaN±NA	30.61±NA	18.65±26.37	-43.37±7.56	0.00±0.00	-1.00±1.41	inactive
NCGC00602667-01		24.32±0.00	24.32±0.00	-96.93±0.36	-98.73±20.33	-7.00±0.00	-7.00±0.00	inactive
NCGC00437228-01		NaN±NA	NaN±NA	-12.16±17.20	-7.70±10.89	0.00±0.00	0.00±0.00	inactive
NCGC00621424-01		15.34±0.00	20.49±1.67	-83.62±9.47	-83.51±7.80	-9.00±0.00	-7.00±0.00	inactive
NCGC00608597-01		17.21±NA	13.00±12.27	-23.22±32.83	-61.84±3.53	-1.00±1.41	-2.00±0.00	inconclusive
NCGC00183299-01		NaN±NA	NaN±NA	0.00±0.00	-10.57±1.91	0.00±0.00	0.00±0.00	inactive
NCGC00371363-02		NaN±NA	NaN±NA	4.31±40.45	0.00±0.00	0.00±0.00	0.00±0.00	inactive
NCGC00346750-02		4.65±1.12	15.34±NA	60.89±12.37	-39.49±55.84	3.50±2.12	-3.50±4.95	inactive
NCGC00645255-01		0.02±NA	15.09±5.98	29.85±19.23	-94.43±0.34	1.50±2.12	-4.50±3.54	inactive
NCGC00639010-01		22.14±11.98	7.86±4.25	-62.27±8.24	-41.20±0.82	-4.50±3.54	-6.00±0.00	inactive
NCGC00371387-01		24.84±13.44	22.98±10.80	-90.59±22.01	-56.38±15.15	-2.00±0.00	-2.50±0.71	inactive
NCGC00621935-01		4.85±0.00	2.08±0.50	-105.29±11.41	-113.68±0.65	-9.00±0.00	-9.00±0.00	inactive
NCGC00524754-01		22.99±1.87	0.09±NA	-59.37±3.48	-14.19±63.59	-5.00±0.00	-1.00±1.41	active
NCGC00239715-01		NaN±NA	25.80±2.10	-9.00±12.73	-66.27±9.64	0.00±0.00	-6.00±1.41	inactive
NCGC00498996-01		28.01±8.96	7.04±2.25	65.53±15.77	-51.37±18.11	3.50±2.12	-6.50±0.71	inactive
NCGC00487825-01		NaN±NA	NaN±NA	-31.39±0.10	-30.19±7.50	0.00±0.00	0.00±0.00	inactive
NCGC00682315-01		16.49±3.99	NaN±NA	-39.50±23.75	-10.97±15.51	-4.00±1.41	0.00±0.00	inconclusive
NCGC00273549-01		24.67±26.26	17.21±NA	-4.98±100.47	-31.61±44.71	1.50±4.95	-1.00±1.41	inactive
NCGC00490325-01		NaN±NA	22.24±0.00	-9.93±14.05	-76.93±1.42	0.00±0.00	-4.50±3.54	inactive
NCGC00676382-01		5.15±0.42	4.85±NA	-71.41±13.90	-28.34±40.08	-9.00±0.00	-1.00±1.41	inactive

NCGC00384396-01		27.28±0.00	24.32±NA	-59.16±11.73	-41.67±3.32	-2.00±0.00	-2.00±2.83	inactive
NCGC00524720-01		48.52±0.00	4.32±NA	-69.01±23.41	-17.84±25.23	-2.00±0.00	-1.50±2.12	active
NCGC00639530-01		36.93±8.93	12.19±0.00	-76.46±30.96	-52.53±3.76	-2.00±0.00	-4.00±1.41	inactive
NCGC00637665-01		34.57±5.60	10.68±4.23	-96.25±19.83	-52.68±4.37	-2.00±0.00	-7.00±1.41	inactive
NCGC00519292-01		NaN±NA	24.48±3.97	-18.14±10.17	-47.53±9.16	0.00±0.00	-3.00±1.41	inactive
NCGC00239760-01		32.48±2.64	22.99±1.87	-82.34±11.27	-40.21±11.57	-2.00±0.00	-2.00±0.00	inactive
NCGC00238724-01		NaN±NA	18.26±1.49	16.91±13.16	-72.06±22.06	0.00±0.00	-6.00±1.41	inactive
NCGC00181439-02		30.81±5.00	10.86±NA	-91.11±15.70	-14.97±49.55	-4.50±3.54	-4.00±5.66	inactive
NCGC00621381-01		6.29±6.46	14.68±9.89	121.91±85.92	-104.23±8.52	7.50±0.71	-7.00±0.00	inactive
NCGC00380445-01		NaN±NA	NaN±NA	-29.18±2.81	0.00±0.00	0.00±0.00	0.00±0.00	inactive
NCGC00417289-01		NaN±NA	0.01±NA	-21.65±NA	21.92±10.85	0.00±0.00	1.50±2.12	inactive
NCGC00500321-01		12.71±9.34	NaN±NA	-54.40±11.88	-39.40±26.50	-4.50±0.71	0.00±0.00	active
NCGC00344074-02	CAY10589	NaN±NA	19.31±NA	39.27±11.80	-27.61±9.31	0.00±0.00	-1.00±1.41	inactive
NCGC00639263-01		7.69±NA	11.33±8.32	9.73±59.81	-76.90±28.02	2.50±3.54	-2.00±0.00	inactive
NCGC00186247-01		18.26±1.49	24.32±0.00	-82.27±10.11	-49.20±2.64	-7.00±0.00	-2.00±0.00	inactive
NCGC00438361-01		6.11±NA	27.28±0.00	36.96±9.43	-81.89±19.43	2.00±2.83	-2.00±0.00	inactive
NCGC00494973-01		12.08±10.23	0.02±NA	-79.39±29.14	8.49±40.19	-4.50±3.54	1.50±2.12	active
NCGC00506278-01		NaN±NA	NaN±NA	-11.67±2.60	-40.38±17.57	0.00±0.00	0.00±0.00	inactive
NCGC00603824-01		13.10±3.17	11.03±8.74	-95.62±7.43	-93.62±30.44	-2.00±0.00	-8.00±1.41	inactive
NCGC00411900-01		31.63±5.13	6.50±8.49	-78.41±4.31	-87.90±22.14	-2.00±0.00	-5.50±4.95	inactive
NCGC00424972-01		45.88±3.73	6.64±7.85	-74.57±7.91	-56.27±4.76	-2.00±0.00	-3.00±0.00	inactive

NCGC00375031-01		53.81± NA	13.35± 8.13	-37.21± 25.42	-31.83± 2.77	-1.00±1.41	-3.00±1.41	inconclusive
NCGC00529399-01		43.24± 0.00	8.63± NA	-75.07± 15.64	-38.15±53.95	-2.00±0.00	-4.50±6.36	inactive
NCGC00253514-01		3.79± 1.50	17.21± NA	-56.17± 1.14	-28.41±10.03	-5.50±3.54	-1.00±1.41	inactive
NCGC00239726-01		33.78±13.38	NaN± NA	-48.80± 0.18	-24.89±13.11	-2.00±0.00	0.00±0.00	inconclusive
NCGC00505848-01		7.69± NA	17.67± 5.66	21.18± 29.95	-47.47± 6.72	1.50±2.12	-2.00±0.00	inactive
NCGC00641276-01		27.28± NA	43.24± NA	17.69± 25.02	-49.31±25.36	1.00±1.41	-1.00±1.41	inactive
NCGC00455481-01		48.52± NA	9.74± 1.58	-30.27± 42.81	-52.86± 8.86	-1.00±1.41	-4.00±2.83	inconclusive
NCGC00506285-01		38.79± 6.29	0.14± NA	-108.05± 7.53	43.70±41.35	-4.50±3.54	1.00±1.41	active
NCGC00387593-01	SR 1824	21.63±30.56	12.19± NA	-40.24± 11.11	-21.28±10.87	-2.00±0.00	-1.00±1.41	inconclusive
NCGC00594158-01		13.67± NA	NaN± NA	32.64± 46.16	-18.94±26.78	1.00±1.41	0.00±0.00	inactive
NCGC00516612-01		43.24± 0.00	19.31± 0.00	-107.67± 21.13	-106.40±13.45	-2.00±0.00	-7.00±0.00	inactive
NCGC00471107-01		NaN± NA	NaN± NA	-9.62± 13.61	0.00± 0.00	0.00±0.00	0.00±0.00	inactive
NCGC00418071-01		8.37± 3.93	21.02± 9.88	46.57± 2.02	-50.84± 1.32	3.00±1.41	-4.50±0.71	inactive
NCGC00682196-01		NaN± NA	NaN± NA	-33.65± 5.78	-20.04±16.27	0.00±0.00	0.00±0.00	inactive
NCGC00455548-01		32.48± 2.64	19.44± 3.15	-99.63± 4.03	-119.20±15.01	-2.00±0.00	-7.00±0.00	inactive
NCGC00429389-01		5.44± NA	0.41± 0.28	9.93± 43.68	-44.75±26.90	1.50±2.12	-5.50±3.54	inactive
NCGC00185837-02		25.80± 2.10	25.80± 2.10	-121.00± 0.54	-116.13± 6.25	-7.00±0.00	-7.00±0.00	inactive
NCGC00506539-01		NaN± NA	NaN± NA	41.15± 39.75	10.99±15.55	0.00±0.00	0.00±0.00	inactive
NCGC00635855-01		3.33± 2.99	6.77± 4.12	-93.89± 0.23	-101.12± 4.33	-3.00±0.00	-8.00±1.41	inactive
NCGC00244218-01		NaN± NA	13.69±15.03	0.46± 31.67	-65.11±10.77	0.00±0.00	-6.00±1.41	inactive
NCGC00640481-01		21.67± 0.00	3.29± 2.21	-34.00± 5.85	-59.58± 0.93	-2.00±0.00	-5.00±4.24	inconclusive

NCGC00632502-01		NaN±NA	NaN±NA	-3.21±26.24	12.69±41.31	0.00±0.00	0.00±0.00	inactive
NCGC00636020-01		24.32±NA	24.32±NA	48.44±31.22	-29.53±41.77	3.50±4.95	-1.00±1.41	inactive
NCGC00165829-03	Myoseverin	10.86±0.00	NaN±NA	-59.79±5.77	-29.98±4.82	-6.00±4.24	0.00±0.00	active
NCGC00411739-01		70.34±NA	0.11±NA	20.32±28.74	9.17±38.07	1.00±1.41	1.50±2.12	inactive
NCGC00014029-01		1.31±0.32	NaN±NA	-76.63±14.92	-16.35±2.15	-9.00±0.00	0.00±0.00	active
NCGC00487820-01		30.61±NA	43.24±0.00	-41.63±18.58	-75.11±20.20	-1.00±1.41	-2.00±0.00	inconclusive
NCGC00437102-01		NaN±NA	NaN±NA	-17.27±9.25	-29.14±3.92	0.00±0.00	0.00±0.00	inactive
NCGC00253491-01		34.35±NA	NaN±NA	29.87±12.20	-22.77±10.64	1.00±1.41	0.00±0.00	inactive
NCGC00506397-01		45.88±3.73	16.93±6.71	-109.40±6.87	-89.71±10.39	-2.00±0.00	-7.00±0.00	inactive
NCGC00424324-01		21.67±NA	17.21±NA	-13.81±55.97	-21.19±29.96	-1.00±1.41	-1.00±1.41	inconclusive
NCGC00493074-01		18.51±4.48	NaN±NA	-104.35±7.10	-24.46±NA	-7.00±0.00	0.00±0.00	active
NCGC00432419-01		NaN±NA	NaN±NA	-5.84±8.26	9.22±13.03	0.00±0.00	0.00±0.00	inactive
NCGC00379148-01	TC-I 15	NaN±NA	NaN±NA	-17.09±5.81	-18.34±12.04	0.00±0.00	0.00±0.00	inactive
NCGC00184181-03		19.31±0.00	6.11±0.00	-90.11±9.49	-90.28±16.00	-7.00±0.00	-9.00±0.00	inactive
NCGC00025071-05	SB 216641 hydrochloride	11.68±2.82	32.48±2.64	-87.26±20.52	-114.12±14.63	-4.50±3.54	-2.00±0.00	inactive
NCGC00347251-01		18.99±7.52	34.35±0.00	-40.74±1.35	-52.41±28.21	-4.00±0.00	-2.00±0.00	inconclusive
NCGC00682787-01		43.24±NA	39.56±12.66	-62.15±87.89	-55.00±21.79	-1.00±1.41	-2.00±0.00	inactive
NCGC00505951-01		43.24±NA	26.83±10.63	31.36±44.35	-52.15±3.83	1.00±1.41	-3.00±1.41	inactive
NCGC00517060-01		NaN±NA	18.99±7.52	7.44±10.53	-65.64±3.59	0.00±0.00	-5.00±0.00	inactive
NCGC00411496-01		33.78±8.17	22.91±23.54	-89.32±9.26	-57.43±25.57	-7.00±0.00	-2.50±0.71	inactive

NCGC00592206-01	Dodecanoyl-D-sucrose	27.46±4.45	NaN±NA	-45.56±1.41	-8.45±11.95	-2.00±0.00	0.00±0.00	inconclusive
NCGC00617910-01		NaN±NA	NaN±NA	10.06±30.06	-10.55±14.93	0.00±0.00	0.00±0.00	inactive
NCGC00469833-01		NaN±NA	24.32±NA	2.60±34.49	-43.37±26.31	0.00±0.00	-1.00±1.41	inactive
NCGC00639072-01		34.35±NA	24.32±NA	0.72±51.44	-40.70±0.39	-1.00±1.41	-1.00±1.41	inconclusive
NCGC00645161-01		NaN±NA	NaN±NA	4.24±6.00	-7.00±9.90	0.00±0.00	0.00±0.00	inactive
NCGC00676390-01		36.93±8.93	15.09±5.98	-108.22±28.07	-108.05±0.99	-4.50±3.54	-7.00±0.00	inactive
NCGC00601146-01		NaN±NA	7.92±8.14	38.58±17.68	-56.99±11.11	0.00±0.00	-4.00±2.83	inactive
NCGC00590939-01		12.93±1.05	43.24±NA	-70.32±8.19	-34.81±14.24	-9.00±0.00	-1.00±1.41	inactive
NCGC00351121-01		22.99±1.87	35.26±11.28	-88.09±10.78	-107.38±16.14	-7.00±0.00	-4.50±3.54	inactive
NCGC00350274-01		27.28±0.00	13.50±8.22	-94.33±13.51	-86.50±38.61	-4.50±3.54	-7.50±0.71	inactive
NCGC00496798-01		22.99±1.87	20.76±5.02	-89.88±2.71	-111.84±6.31	-2.00±0.00	-2.00±0.00	inactive
NCGC00097089-01		9.28±2.24	6.85±NA	-76.70±3.55	-40.60±10.24	-7.00±0.00	-2.00±2.83	inactive
NCGC00023726-01		45.88±3.73	22.99±1.87	-105.96±8.60	-112.11±23.38	-2.00±0.00	-4.50±3.54	inactive
NCGC00596185-01		43.24±0.00	20.49±1.67	-77.97±0.73	-56.75±7.83	-2.00±0.00	-4.50±3.54	inactive
NCGC00496045-01		45.88±3.73	12.40±16.85	-68.85±32.92	-48.68±20.15	-2.00±0.00	-2.50±0.71	inactive
NCGC00476390-01		NaN±NA	17.21±NA	-2.20±38.50	-22.60±31.97	0.00±0.00	-1.00±1.41	inactive
NCGC00639519-01		5.15±0.42	3.64±0.30	-85.84±16.49	-103.25±1.00	-6.00±4.24	-9.00±0.00	inactive
NCGC00417217-01		37.38±17.56	41.97±3.41	-101.93±0.03	-97.21±2.47	-4.50±3.54	-7.00±0.00	inactive
NCGC00181430-02		14.51±1.18	21.82±3.54	-99.49±19.29	-115.40±13.33	-7.00±0.00	-7.00±0.00	inactive
NCGC00483886-01		68.53±NA	2.65±2.37	-70.65±57.13	-33.34±14.94	-1.00±1.41	-3.00±0.00	active

NCGC00452893-01		NaN±NA	NaN±NA	-3.27±33.69	-10.16±14.37	0.00±0.00	0.00±0.00	inactive
NCGC00483869-01		3.29±0.80	15.34±NA	153.33±8.46	-28.97±13.58	9.00±0.00	-1.00±1.41	inactive
NCGC00356615-01		14.51±1.18	14.04±4.49	-114.65±22.10	-78.10±13.84	-7.00±0.00	-8.00±1.41	inactive
NCGC00608711-01		NaN±NA	15.15±9.22	-6.63±49.64	-60.67±40.77	0.00±0.00	-2.00±0.00	inactive
NCGC00537422-01		NaN±NA	NaN±NA	0.00±0.00	-16.48±23.31	0.00±0.00	0.00±0.00	inactive
NCGC00386996-02		12.38±9.81	26.83±10.63	61.10±21.00	-83.50±0.14	6.50±0.71	-4.50±3.54	inactive
NCGC00244887-01		19.16±0.00	34.11±37.46	-72.39±1.55	-47.77±14.26	-7.00±0.00	-4.00±2.83	inactive
NCGC00433541-01		29.33±7.09	35.26±11.28	-90.92±14.37	-55.72±3.10	-4.50±3.54	-2.00±0.00	inactive
NCGC00682322-01		3.96±1.27	43.24±NA	-36.84±3.09	-19.03±47.37	-4.50±2.12	-1.00±1.41	inconclusive
NCGC00622469-01		5.91±7.00	NaN±NA	-64.06±9.68	-4.58±45.68	-6.50±2.12	0.00±0.00	active
NCGC00633127-01		4.59±0.37	NaN±NA	-67.42±5.30	-16.30±23.05	-9.00±0.00	0.00±0.00	active
NCGC00638959-01		35.26±11.28	14.51±1.18	-127.71±1.53	-111.22±14.42	-4.50±3.54	-7.00±0.00	inactive
NCGC00499010-01		NaN±NA	21.67±0.00	24.82±13.34	-68.40±0.39	0.00±0.00	-2.00±0.00	inactive
NCGC00636938-01		NaN±NA	NaN±NA	11.24±15.89	-6.49±9.18	0.00±0.00	0.00±0.00	inactive
NCGC00447367-01		43.24±NA	27.88±15.08	-33.09±74.34	-93.47±4.93	-1.00±1.41	-2.00±0.00	inconclusive
NCGC00347258-01		14.51±1.18	35.26±11.28	-115.48±26.10	-81.90±3.14	-2.00±0.00	-2.00±0.00	inactive
NCGC00487746-01		NaN±NA	0.02±NA	36.86±0.23	11.89±16.82	0.00±0.00	1.50±2.12	inactive
NCGC00455552-01		15.34±0.00	5.35±2.12	-69.93±8.26	-88.64±0.34	-3.00±0.00	-7.00±0.00	inactive
NCGC00505072-01		48.52±0.00	7.74±1.25	-98.00±10.64	-75.43±7.09	-2.00±0.00	-7.00±0.00	inactive
NCGC00274239-03		21.82±3.54	30.81±5.00	-101.49±0.93	-96.34±1.22	-7.00±0.00	-7.00±0.00	inactive
NCGC00634590-01		4.59±0.37	1.85±0.45	-94.60±19.49	-95.74±9.88	-9.00±0.00	-9.00±0.00	inactive
NCGC00488005-01		4.40±3.72	12.59±2.04	241.33±45.38	-87.57±4.44	8.00±1.41	-7.00±0.00	inactive

NCGC00241082-02		18.26±1.49	21.67±0.00	-88.90±7.94	-84.19±0.82	-7.00±0.00	-7.00±0.00	inactive
NCGC00417848-01		37.90±9.17	NaN±NA	-46.38±7.91	19.18±9.02	-2.00±0.00	0.00±0.00	inconclusive
NCGC00181461-02		28.95±2.35	23.30±5.63	-84.55±3.80	-51.96±4.66	-7.00±0.00	-2.00±0.00	inactive
NCGC00090154-01		22.99±1.87	NaN±NA	-66.67±23.98	-38.63±27.39	-3.00±1.41	0.00±0.00	active
NCGC00455538-01		22.05±29.97	6.57±1.59	-13.01±94.00	-61.14±10.47	1.50±4.95	-5.00±4.24	inactive
NCGC00635159-01		36.44±2.96	25.80±2.10	-59.44±24.00	-70.80±6.30	-2.00±0.00	-4.50±3.54	inactive
NCGC00492522-01		19.31±NA	9.94±3.18	-48.18±23.67	-41.58±13.53	-1.00±1.41	-2.50±0.71	inconclusive
NCGC00484582-01		NaN±NA	NaN±NA	-14.42±1.95	11.40±16.13	0.00±0.00	0.00±0.00	inactive
NCGC00343704-01		24.32±0.00	6.15±1.00	-111.31±7.75	-65.63±9.15	-4.50±3.54	-7.00±2.83	inactive
NCGC00524715-01		27.28±0.00	11.15±3.57	-44.72±2.79	-61.54±7.61	-2.00±0.00	-2.00±0.00	inconclusive
NCGC00437272-01		22.25±7.12	1.37±NA	-45.01±3.69	-34.61±3.92	-2.00±0.00	-1.00±1.41	inconclusive
NCGC00350803-01		18.51±4.48	8.16±0.66	-76.79±1.96	-48.29±12.94	-7.00±0.00	-5.50±3.54	inactive
NCGC00253553-01		NaN±NA	15.75±5.04	2.93±30.77	-44.57±5.12	0.00±0.00	-5.00±1.41	inactive
NCGC00240451-01		NaN±NA	NaN±NA	33.24±8.32	0.00±0.00	0.00±0.00	0.00±0.00	inactive
NCGC00633293-01		NaN±NA	20.76±5.02	14.83±20.98	-44.89±6.74	0.00±0.00	-4.00±0.00	inactive
NCGC00350815-01		45.88±3.73	6.57±1.59	-101.39±19.93	-70.63±19.47	-2.00±0.00	-7.50±0.71	inactive
NCGC00640599-01		22.99±1.87	2.07±1.40	-78.25±7.58	-52.96±4.70	-2.00±0.00	-5.50±3.54	inactive
NCGC00250276-01		43.24±0.00	14.51±1.18	-96.38±2.56	-51.61±6.44	-2.00±0.00	-4.50±0.71	inactive
NCGC00500296-01		NaN±NA	NaN±NA	-28.78±NA	-8.12±11.49	0.00±0.00	0.00±0.00	inactive
NCGC00263683-01		NaN±NA	0.61±NA	-39.78±14.98	21.48±30.38	0.00±0.00	1.00±1.41	inactive
NCGC00181478-01		43.24±0.00	38.79±6.29	-114.39±12.10	-114.10±4.72	-2.00±0.00	-2.00±0.00	inactive
NCGC00530810-01		28.95±2.35	2.20±1.74	-76.96±3.36	-84.79±18.55	-4.50±3.54	-8.00±1.41	inactive

NCGC00378383-01		43.24±NA	32.46±15.25	-48.91±17.42	-56.51±15.74	-1.00±1.41	-2.00±0.00	inconclusive
NCGC00410870-01		21.87±8.67	15.49±6.14	-98.87±12.81	-95.08±13.71	-4.50±3.54	-7.00±0.00	inactive
NCGC00524748-01		25.80±2.10	5.57±7.49	-70.07±3.30	-52.08±1.82	-7.00±0.00	-2.00±0.00	inactive
NCGC00424246-01		6.85±NA	4.53±5.79	3.33±53.01	-48.43±2.53	2.00±2.83	-3.00±0.00	inactive
NCGC00492875-01		34.57±5.60	2.14±1.30	-100.20±1.43	-66.85±6.78	-2.00±0.00	-5.50±4.95	inactive
NCGC00350796-01		30.61±0.00	11.52±0.94	-105.36±2.14	-76.52±1.46	-4.50±3.54	-5.50±4.95	inactive
NCGC00384478-01		33.78±13.38	4.71±4.22	-74.39±14.39	-41.73±0.89	-3.50±2.12	-6.00±0.00	inactive
NCGC00011359-01		NaN±NA	NaN±NA	0.00±0.00	14.66±20.73	0.00±0.00	0.00±0.00	inactive
NCGC00418445-01		12.84±4.11	19.82±0.00	-91.15±3.08	-66.36±25.05	-9.00±0.00	-5.00±2.83	inactive
NCGC00162037-01		19.31±0.00	30.61±NA	-83.77±11.17	-63.15±52.87	-4.50±3.54	-1.00±1.41	inactive
NCGC00411677-01		20.26±10.96	NaN±NA	83.58±24.63	-29.03±23.79	8.00±1.41	0.00±0.00	inactive
NCGC00658403-01		32.46±15.25	37.90±15.01	-80.48±7.15	-72.06±28.21	-4.50±3.54	-2.00±0.00	inactive
NCGC00681565-01		NaN±NA	34.35±NA	-21.38±30.24	-42.53±12.60	0.00±0.00	-1.00±1.41	inactive
NCGC00496797-01		32.48±2.64	15.75±5.04	-113.10±1.24	-81.77±15.46	-7.00±0.00	-7.00±0.00	inactive
NCGC00676409-01		33.78±13.38	13.24±15.66	-55.87±13.10	-46.20±17.34	-2.00±0.00	-2.50±0.71	inactive
NCGC00637900-01		3.29±0.80	24.32±NA	-77.78±38.66	-33.74±47.71	-5.00±4.24	-1.00±1.41	inactive
NCGC00680633-01		NaN±NA	NaN±NA	-15.17±21.46	0.00±0.00	0.00±0.00	0.00±0.00	inactive
NCGC00318955-01		43.24±0.00	12.19±0.00	-117.21±9.28	-68.86±11.59	-2.00±0.00	-5.00±2.83	inactive
NCGC00263695-01		24.05±27.14	8.74±11.98	-20.48±111.60	-58.90±17.87	0.00±2.83	-5.50±4.95	inactive
NCGC00447285-01		43.24±0.00	15.75±5.04	-126.65±62.65	-101.68±11.40	-2.00±0.00	-7.00±0.00	inactive

NCGC00466408-01		NaN±NA	27.46±4.45	19.42±5.50	-43.35±12.42	0.00±0.00	-2.00±0.00	inactive
NCGC00635188-01		13.69±15.03	19.31±0.00	-130.02±53.53	-59.61±1.55	-4.50±3.54	-5.00±0.00	inactive
NCGC00262313-01		43.24±0.00	7.27±3.41	-97.32±7.59	-61.43±21.95	-2.00±0.00	-6.50±0.71	inactive
NCGC00634115-01		NaN±NA	43.24±0.00	10.92±15.45	-50.19±2.33	0.00±0.00	-2.00±0.00	inactive
NCGC00620572-01		43.24±NA	2.36±2.11	-33.13±0.94	-31.76±9.45	-1.00±1.41	-4.50±2.12	inconclusive
NCGC00346961-01	HMSL10076	21.82±3.54	NaN±NA	-123.25±15.55	-25.10±NA	-7.00±0.00	0.00±0.00	active
NCGC00500228-01		NaN±NA	15.34±NA	2.77±29.38	-19.26±27.24	0.00±0.00	-1.50±2.12	inactive
NCGC00092351-01		43.24±0.00	5.84±3.94	-66.32±2.15	-71.92±37.17	-2.00±0.00	-6.50±0.71	inactive
NCGC00521797-01		0.02±NA	NaN±NA	26.16±15.15	-22.43±6.81	1.50±2.12	0.00±0.00	inactive
NCGC00455535-01		34.35±0.00	21.67±0.00	-100.59±2.39	-111.96±7.65	-2.00±0.00	-7.00±0.00	inactive
NCGC00386581-01	RU 42698	24.32±0.00	NaN±NA	-69.50±7.95	0.00±0.00	-3.50±2.12	0.00±0.00	active
NCGC00476420-01		35.25±NA	NaN±NA	-49.67±NA	-2.58±29.93	-1.00±1.41	0.00±0.00	inconclusive
NCGC00501143-01		NaN±NA	NaN±NA	-22.06±14.54	-10.68±15.10	0.00±0.00	0.00±0.00	inactive
NCGC00244483-01		40.89±3.33	43.24±NA	-93.56±13.12	-24.89±55.72	-2.00±0.00	-1.00±1.41	inactive
NCGC00636897-01		NaN±NA	48.52±NA	0.00±0.00	-29.25±41.37	0.00±0.00	-1.00±1.41	inactive
NCGC00411834-01		NaN±NA	26.62±25.12	-3.50±4.94	-49.17±1.52	0.00±0.00	-4.00±2.83	inactive
NCGC00632378-01		NaN±NA	NaN±NA	-11.52±NA	0.00±0.00	0.00±0.00	0.00±0.00	inactive
NCGC00241703-02		43.24±0.00	10.41±2.52	-58.21±5.64	-67.06±11.43	-2.00±0.00	-6.00±1.41	inactive
NCGC00506827-01	SPL 334	22.99±1.87	NaN±NA	-80.23±23.79	-41.67±NA	-2.00±0.00	0.00±0.00	active
NCGC00640462-01		0.00±NA	5.56±3.01	8.07±42.35	-51.69±24.43	-1.50±2.12	-3.50±2.12	inconclusive
NCGC00632198-01		40.89±3.33	4.87±6.80	-65.50±6.63	-34.45±10.19	-2.00±0.00	-3.00±0.00	active

NCGC00375147-01		NaN±NA	NaN±NA	-7.82±11.06	-10.29±50.73	0.00±0.00	0.00±0.00	inactive
NCGC00431023-01		NaN±NA	7.73±10.76	-32.73±8.55	-46.12±3.89	0.00±0.00	-2.50±0.71	inactive
NCGC00489047-01	Oxacillin (sodium salt)	NaN±NA	NaN±NA	11.93±16.86	0.00±0.00	0.00±0.00	0.00±0.00	inactive
NCGC00355903-01		16.27±7.64	4.93±6.72	-99.02±32.08	-29.69±7.34	-2.00±0.00	-2.50±0.71	active
NCGC00493076-01		26.46±23.73	9.28±2.24	-5.70±74.93	-42.50±1.82	1.00±4.24	-4.50±2.12	inactive
NCGC00432247-01		12.19±0.00	6.48±0.53	-97.14±6.90	-91.79±0.26	-9.00±0.00	-9.00±0.00	inactive
NCGC00025314-01	MRS 1845	13.64±19.29	NaN±NA	-45.01±24.18	-15.65±56.47	-2.00±0.00	0.00±0.00	inconclusive
NCGC00426420-01		41.43±10.02	13.10±3.17	-69.48±1.78	-54.69±12.07	-2.00±0.00	-5.50±0.71	inactive
NCGC00516584-01		NaN±NA	6.86±9.63	-14.58±33.19	-42.21±13.99	0.00±0.00	-4.00±2.83	inactive
NCGC00510093-01	Takeda103A	NaN±NA	6.11±NA	-10.58±NA	53.61±7.75	0.00±0.00	1.00±1.41	inactive
NCGC00618634-01		6.85±NA	10.86±NA	39.48±15.82	-24.84±2.60	1.00±1.41	-1.50±2.12	inactive
NCGC00476458-01		NaN±NA	NaN±NA	17.14±4.85	-19.48±27.55	0.00±0.00	0.00±0.00	inactive
NCGC00244507-01		43.24±0.00	24.48±3.97	-85.63±11.67	-70.49±6.08	-2.00±0.00	-4.50±3.54	inactive
NCGC00641177-01		48.52±0.00	12.19±0.00	-127.89±5.69	-85.21±13.54	-2.00±0.00	-7.00±0.00	inactive
NCGC00244891-02		16.28±1.32	39.56±12.66	-98.00±3.45	-53.35±3.92	-7.00±0.00	-2.00±0.00	inactive
NCGC00434959-01		7.69±NA	0.02±NA	1.40±49.71	20.10±28.43	1.00±1.41	1.50±2.12	inactive
NCGC00684826-01		43.24±0.00	6.48±0.53	-69.56±7.95	-56.41±2.21	-2.00±0.00	-8.00±0.00	inactive
NCGC00476475-01		20.49±1.67	4.32±NA	-83.87±1.60	-26.80±NA	-7.00±0.00	-1.50±2.12	inactive
NCGC00345537-01		43.24±0.00	9.52±3.77	-91.80±5.82	-48.63±0.99	-2.00±0.00	-5.00±1.41	inactive
NCGC00637519-01		NaN±NA	NaN±NA	11.82±16.72	4.26±6.03	0.00±0.00	0.00±0.00	inactive
NCGC00608406-01		27.28±NA	NaN±NA	-19.80±28.00	0.00±0.00	-1.00±1.41	0.00±0.00	inconclusive
NCGC00635125-01		8.63±0.00	3.38±1.34	-100.32±7.76	-108.43±4.34	-9.00±0.00	-9.00±0.00	inactive
NCGC00626932-01		NaN±NA	NaN±NA	-9.97±14.10	-9.77±13.82	0.00±0.00	0.00±0.00	inactive

NCGC00524716-01		16.28±1.32	10.86±NA	-72.51±10.69	-20.04±28.34	-7.00±0.00	-1.00±1.41	inactive
NCGC00011271-01		NaN±NA	NaN±NA	-7.35±10.40	-16.98±24.02	0.00±0.00	0.00±0.00	inactive
NCGC00608516-01		11.68±2.82	NaN±NA	-93.10±32.33	-22.17±31.35	-9.00±0.00	0.00±0.00	active
NCGC00650745-01		9.94±NA	14.03±0.00	46.45±16.42	-69.28±6.57	1.00±1.41	-5.00±0.00	inactive
NCGC00355981-01		45.88±3.73	NaN±NA	-56.33±1.73	-15.51±10.98	-2.00±0.00	0.00±0.00	active
NCGC00496825-01		5.18±6.36	24.32±NA	42.16±2.13	-17.32±65.20	2.50±0.71	-3.50±4.95	inactive
NCGC00384300-01		17.49±13.85	30.61±0.00	-54.13±8.91	-56.88±7.84	-2.00±0.00	-2.00±0.00	inactive
NCGC00676379-01		17.21±0.00	4.35±0.71	-86.77±10.62	-58.22±9.06	-7.00±0.00	-5.00±4.24	inactive
NCGC00641658-01		5.85±1.42	37.90±15.01	108.99±55.57	-75.28±5.79	4.50±3.54	-2.00±0.00	inactive
NCGC00506292-01		43.24±0.00	0.00±NA	-60.08±4.59	14.22±20.11	-2.00±0.00	1.50±2.12	active
NCGC00379268-01		5.48±0.89	7.69±NA	-35.23±3.14	-47.13±8.27	-4.50±2.12	-2.50±3.54	inconclusive
NCGC00506405-01		38.79±6.29	24.48±3.97	-115.79±5.33	-58.01±3.38	-2.00±0.00	-5.00±0.00	inactive
NCGC00676377-01		43.24±0.00	4.71±4.22	-95.56±7.06	-90.47±26.59	-2.00±0.00	-7.00±0.00	inactive
NCGC00633179-01		43.24±0.00	22.99±1.87	-99.28±2.28	-87.65±1.96	-2.00±0.00	-7.00±0.00	inactive
NCGC00624145-01		30.61±NA	NaN±NA	-13.03±37.07	-19.10±27.01	-1.00±1.41	0.00±0.00	inconclusive
NCGC00455487-01		43.24±NA	14.68±9.89	-50.02±12.96	-70.09±16.66	-1.00±1.41	-4.50±3.54	inactive
NCGC00443091-01		NaN±NA	6.77±4.12	-16.26±12.33	-57.93±12.11	0.00±0.00	-2.00±0.00	inactive
NCGC00506307-01		NaN±NA	NaN±NA	9.69±13.71	-8.43±35.25	0.00±0.00	0.00±0.00	inactive
NCGC00412201-01		21.96±13.37	NaN±NA	-103.65±9.72	-9.71±13.73	-7.00±0.00	0.00±0.00	active
NCGC00183800-01		1.54±0.25	0.99±0.53	-99.26±3.21	-98.12±14.62	-9.00±0.00	-9.00±0.00	inactive
NCGC00371362-01		31.28±16.92	34.57±5.60	-106.14±13.83	-100.64±8.10	-4.50±3.54	-4.50±3.54	inactive

NCGC00505080-01		24.32±0.00	15.09±5.98	-75.24±8.12	-71.02±18.42	-4.50±3.54	-5.00±2.83	inactive
NCGC00528988-01		36.42±17.11	12.27±1.99	-81.55±33.71	-76.60±22.25	-2.00±0.00	-5.00±2.83	inactive
NCGC00622267-01		9.68±0.00	8.68±1.41	-91.43±11.47	-113.56±3.31	-9.00±0.00	-7.00±0.00	inactive
NCGC00620363-01		18.51±4.48	8.63±0.00	-39.38±6.88	-79.34±18.18	-3.00±1.41	-6.00±1.41	inconclusive

Table S8. Testing of the predicted anti- SARS-CoV-2 compounds using in vitro RdRp assay

Sample.ID	Sample.Name	IC50 µM	Efficacy	Curve rank	class
NCGC00355977-01		12.87± 0.00	-42.60± 7.72	-2.00±0.00	active
NCGC00537529-01		19.88± 6.36	-117.60± 21.14	-7.00±0.00	active
NCGC00390330-01	Caspase-3InhibitorQ-DEVD-OPh	NaN± NA	-20.42± 14.03	0.00±0.00	inactive
NCGC00244886-01		NaN± NA	-25.01± 15.18	0.00±0.00	inactive
NCGC00378433-01		13.65± 1.11	-88.37± 7.79	-7.00±0.00	active
NCGC00411620-01		NaN± NA	-9.27± 13.11	0.00±0.00	inactive
NCGC00476335-01		NaN± NA	-25.63± 15.85	0.00±0.00	inactive
NCGC00411630-01		NaN± NA	5.66± 8.00	0.00±0.00	inactive
NCGC00500211-01		NaN± NA	12.91± 1.62	0.00±0.00	inactive
NCGC00505662-01		NaN± NA	-21.76± 2.92	0.00±0.00	inactive
NCGC00451966-01		5.75± NA	-30.57± 43.23	-1.00±1.41	active
NCGC00674791-01		4.07± NA	-23.76± 18.76	-1.50±2.12	active
NCGC00433546-01		NaN± NA	7.71± 10.91	0.00±0.00	inactive
NCGC00351816-01		4.43± 3.97	-71.47± 18.25	-4.50±3.54	active
NCGC00384396-01		NaN± NA	-16.22± 8.09	0.00±0.00	inactive
NCGC00483855-01		NaN± NA	-9.77± 3.61	0.00±0.00	inactive
NCGC00666300-01		NaN± NA	-11.71± 16.56	0.00±0.00	inactive
NCGC00186247-01		9.11± NA	-39.57± 0.06	-2.00±2.83	active
NCGC00413956-01		NaN± NA	11.15± 3.32	0.00±0.00	inactive
NCGC00506278-01		NaN± NA	12.67± 2.17	0.00±0.00	inactive
NCGC00424972-01		NaN± NA	8.94± 12.65	0.00±0.00	inactive
NCGC00375031-01		NaN± NA	15.57± 2.43	0.00±0.00	inactive
NCGC00417202-01		26.36± NA	-33.32± 47.12	-1.00±1.41	active
NCGC00413954-01		NaN± NA	13.35± 3.27	0.00±0.00	inactive
NCGC00165849-03	NS3694	7.24± NA	-68.74± 68.46	-3.50±4.95	active
NCGC00483359-01		NaN± NA	-6.01± 36.14	0.00±0.00	inactive
NCGC00387593-01	SR 1824	10.05± 3.98	-50.36± 7.64	-3.50±2.12	active
NCGC00418071-01		17.07± 5.46	-85.21± 31.35	-3.50±2.12	active
NCGC00538118-01	Losartan Related Compound B (2-	NaN± NA	14.86± 6.60	0.00±0.00	inactive

	Butyl-4-chloro-(1-{[2 -(1H-tetrazol-5-yl)biphenyl-4-yl]methyl}-1H-imidazol-5-yl)methyl acetate)				
NCGC00412159-01		NaN± NA	7.87± 11.13	0.00±0.00	inactive
NCGC00379263-01		NaN± NA	5.75± 8.13	0.00±0.00	inactive
NCGC00471124-01		NaN± NA	22.22± 14.71	0.00±0.00	inactive
NCGC00500357-01		NaN± NA	15.37± 21.74	0.00±0.00	inactive
NCGC00506397-01		6.84± 3.21	-61.84± 11.64	-3.50±2.12	active
NCGC00424324-01		NaN± NA	10.74± 1.66	0.00±0.00	inactive
NCGC00517634-01		NaN± NA	5.69± 8.04	0.00±0.00	inactive
NCGC00420893-01	SR1664	18.18± NA	-31.56± 24.08	-1.00±1.41	active
NCGC00483901-01		NaN± NA	-20.67± 5.29	0.00±0.00	inactive
NCGC00483913-01		12.87± NA	-42.88± 42.25	-1.00±1.41	active
NCGC00521795-01		NaN± NA	16.96± 11.26	0.00±0.00	inactive
NCGC00506876-03	AZELIRAGON	7.68± 0.62	-88.13± 13.14	-7.00±0.00	active
NCGC00257326-01	PharmaGSID_48507	NaN± NA	13.53± 7.09	0.00±0.00	inactive
NCGC00496824-01		NaN± NA	-5.69± 8.05	0.00±0.00	inactive
NCGC00345157-01		NaN± NA	14.20± 7.27	0.00±0.00	inactive
NCGC00505664-01		NaN± NA	-8.71± 12.31	0.00±0.00	inactive
NCGC00504961-01		16.20± NA	-29.64± 13.70	-1.00±1.41	active
NCGC00678299-01		4.57± NA	-32.44± 45.88	-1.00±1.41	active
NCGC00496798-01		NaN± NA	-4.35± 31.27	0.00±0.00	inactive
NCGC00411627-01		NaN± NA	9.93± 14.05	0.00±0.00	inactive
NCGC00476390-01		NaN± NA	-15.42± 1.05	0.00±0.00	inactive
NCGC00253474-01		NaN± NA	-15.12± 4.04	0.00±0.00	inactive
NCGC00483480-01		0.06± NA	-21.51± 30.41	-1.00±1.41	active
NCGC00417217-01		NaN± NA	10.97± 1.09	0.00±0.00	inactive
NCGC00483886-01		NaN± NA	-3.59± 20.40	0.00±0.00	inactive
NCGC00483869-01		5.12± NA	-20.22± 14.19	-1.00±1.41	active
NCGC00504962-01		NaN± NA	7.24± 10.24	0.00±0.00	inactive

NCGC00387556-02	HZ52	12.87± 0.00	-87.34± 9.92	-7.00±0.00	active
NCGC00500203-01		NaN± NA	3.14± 4.44	0.00±0.00	inactive
NCGC00417872-01		NaN± NA	20.06± 10.39	0.00±0.00	inactive
NCGC00676413-01		2.57± NA	-10.26± 14.51	-1.50±2.12	active
NCGC00483899-01		6.62± NA	-47.78± 18.60	-2.50±3.54	active
NCGC00621616-01		NaN± NA	-18.22± 4.48	0.00±0.00	inactive
NCGC00347258-01		7.43± 2.38	-56.66± 16.44	-5.50±2.12	active
NCGC00454170-01		3.26± 4.51	-36.04± 0.45	-2.00±0.00	active
NCGC00398802-01		NaN± NA	-10.47± 14.81	0.00±0.00	inactive
NCGC00241734-01		NaN± NA	10.44± 14.76	0.00±0.00	inactive
NCGC00241082-02		NaN± NA	5.01± 7.08	0.00±0.00	inactive
NCGC00479274-01		0.00± NA	13.46± 19.04	1.50±2.12	inconclusive
NCGC00161823-02	L-803,087 trifluoroacetate	NaN± NA	2.82± 19.90	0.00±0.00	inactive
NCGC00379899-01		NaN± NA	13.70± 6.07	0.00±0.00	inactive
NCGC00674675-01		NaN± NA	-10.76± 6.44	0.00±0.00	inactive
NCGC00408842-01	Glaziovine	10.49± 3.36	-35.24± 21.36	-2.00±0.00	active
NCGC00610492-01		NaN± NA	0.00± 0.00	0.00±0.00	inactive
NCGC00678290-01		3.23± NA	-37.95± 19.80	-1.00±1.41	active
NCGC00418129-01		17.64± 1.43	-90.96± 1.40	-4.50±3.54	active
NCGC00343704-01		14.54± 2.36	-107.49± 78.72	-4.50±3.54	active
NCGC00501110-01		NaN± NA	8.57± 29.61	0.00±0.00	inactive
NCGC00250276-01		NaN± NA	11.66± 5.89	0.00±0.00	inactive
NCGC00181478-01		10.66± 7.83	-39.45± 1.70	-3.00±1.41	active
NCGC00378383-01		5.51± 1.33	-66.44± 6.98	-7.00±2.83	active
NCGC00413948-01		0.06± NA	-14.05± 53.84	-1.00±1.41	active
NCGC00014952-02		3.82± 4.83	-121.90± 50.78	-8.00±1.41	active
NCGC00411677-01		NaN± NA	-31.47± 12.91	0.00±0.00	inactive
NCGC00318955-01		12.95± 2.10	-145.28± 8.39	-7.00±0.00	active
NCGC00524815-01		NaN± NA	12.24± 17.31	0.00±0.00	inactive

NCGC00505692-01		NaN± NA	-7.68± 10.87	0.00±0.00	inactive
NCGC00418072-01		15.93± 3.85	-87.42± 21.30	-4.50±3.54	active
NCGC00411658-01		NaN± NA	-23.71± 5.25	0.00±0.00	inactive
NCGC00418149-01		NaN± NA	-17.13± 8.60	0.00±0.00	inactive
NCGC00622061-01		2.04± NA	-29.50± 65.22	-2.50±3.54	active
NCGC00620572-01		NaN± NA	-12.84± 8.16	0.00±0.00	inactive
NCGC00500237-01		0.00± NA	11.67± 16.50	1.50±2.12	inconclusive
NCGC00499813-01		NaN± NA	21.58± 7.09	0.00±0.00	inactive
NCGC00500217-01		7.43± NA	-34.03± 22.84	-1.50±2.12	active
NCGC00521797-01		NaN± NA	14.27± 3.24	0.00±0.00	inactive
NCGC00476420-01		NaN± NA	-17.89± 11.41	0.00±0.00	inactive
NCGC00242591-01		NaN± NA	13.46± 0.15	0.00±0.00	inactive
NCGC00501143-01		5.12± NA	-28.70± 0.81	-1.00±1.41	active
NCGC00355903-01		NaN± NA	-9.51± 13.45	0.00±0.00	inactive
NCGC00505679-01		7.24± NA	-36.43± 1.50	-1.00±1.41	active
NCGC00418057-01		17.34±12.74	-65.10± 6.77	-3.50±2.12	active
NCGC00476458-01		NaN± NA	0.00± 0.00	0.00±0.00	inactive
NCGC00529685-01		NaN± NA	0.00± 0.00	0.00±0.00	inactive
NCGC00411681-01		5.90± NA	-23.04± 6.22	-1.00±1.41	active
NCGC00641177-01		14.54± 2.36	-121.69±100.45	-4.50±3.54	active
NCGC00505676-01		NaN± NA	5.36± 7.58	0.00±0.00	inactive
NCGC00434959-01		NaN± NA	3.01± 4.26	0.00±0.00	inactive
NCGC00476475-01		NaN± NA	-14.06± 19.89	0.00±0.00	inactive
NCGC00678304-01		NaN± NA	-17.29± 6.71	0.00±0.00	inactive
NCGC00678294-01		NaN± NA	-23.99± 18.55	0.00±0.00	inactive
NCGC00639442-01		NaN± NA	9.96± 14.09	0.00±0.00	inactive
NCGC00496825-01		NaN± NA	-7.51± 10.62	0.00±0.00	inactive
NCGC00244240-01		NaN± NA	11.03± 0.49	0.00±0.00	inactive
NCGC00384300-01		0.01± NA	22.92± 6.26	1.50±2.12	inconclusive
NCGC00379268-01		0.06± NA	-18.42± 26.05	-1.50±2.12	active

NCGC00432889-01		NaN± NA	4.22± 5.97	0.00±0.00	inactive
NCGC00391999-01		NaN± NA	-5.85± 8.27	0.00±0.00	inactive
NCGC00188935-01		NaN± NA	7.93± 11.22	0.00±0.00	inactive
NCGC00506066-01		0.64± NA	-30.64± 43.33	-2.50±3.54	active
NCGC00506266-01		NaN± NA	19.84± 18.10	0.00±0.00	inactive
NCGC00411666-01		NaN± NA	-17.50± 0.71	0.00±0.00	inactive
NCGC00412201-01		14.82± NA	-28.24± 17.21	-1.00±1.41	active
NCGC00594390-01		1.82± 0.00	-49.67± 10.78	-2.00±0.00	active
NCGC00411649-01		NaN± NA	-6.43± 9.10	0.00±0.00	inactive
NCGC00524752-01		NaN± NA	7.91± 23.19	0.00±0.00	inactive
NCGC00371362-01		13.65± 1.11	-50.37± 12.97	-4.50±0.71	active
NCGC00622266-01		2.57± NA	-28.67± 9.80	-3.00±4.24	active
NCGC00517371-01		NaN± NA	-5.62± 7.95	0.00±0.00	inactive
